# Depletion of *Arabidopsis* SC35 and SC35-like serine/arginine-rich proteins affects the transcription and splicing of a subset of genes

**DOI:** 10.1371/journal.pgen.1006663

**Published:** 2017-03-08

**Authors:** Qingqing Yan, Xi Xia, Zhenfei Sun, Yuda Fang

**Affiliations:** National key Laboratory of Plant Molecular Genetics, Chinese Academy of Sciences Center for Excellence in Molecular Plant Sciences, Institute of Plant Physiology and Ecology, Chinese Academy of Sciences; University of Chinese Academy of Sciences, Shanghai, China; Peking University, CHINA

## Abstract

Serine/arginine-rich (SR) proteins are important splicing factors which play significant roles in spliceosome assembly and splicing regulation. However, little is known regarding their biological functions in plants. Here, we analyzed the phenotypes of mutants upon depleting different subfamilies of *Arabidopsis* SR proteins. We found that loss of the functions of SC35 and SC35-like (SCL) proteins cause pleiotropic changes in plant morphology and development, including serrated leaves, late flowering, shorter roots and abnormal silique phyllotaxy. Using RNA-seq, we found that SC35 and SCL proteins play roles in the pre-mRNA splicing. Motif analysis revealed that SC35 and SCL proteins preferentially bind to a specific RNA sequence containing the AGAAGA motif. In addition, the transcriptions of a subset of genes are affected by the deletion of SC35 and SCL proteins which interact with NRPB4, a specific subunit of RNA polymerase II. The splicing of *FLOWERING LOCUS C* (*FLC*) intron1 and transcription of *FLC* were significantly regulated by SC35 and SCL proteins to control *Arabidopsis* flowering. Therefore, our findings provide mechanistic insight into the functions of plant SC35 and SCL proteins in the regulation of splicing and transcription in a direct or indirect manner to maintain the proper expression of genes and development.

## Author summary

SR proteins were identified to be important splicing factors. This work generated mutants of different subfamilies of the classic *Arabidopsis* SR proteins. Genetic analysis revealed that loss of the function of SC35/SCL proteins influences the plant development. This study revealed SC35/SCL proteins regulate alternative splicing, preferentially bind a specific RNA motif, interact with NRPB4, and affect the transcription of a subset of genes. This study further revealed that SC35/SCL proteins control flowering by regulating the splicing and transcription of *FLC*. These results shed light on the functions of SR proteins in plants.

## Introduction

Alternative splicing (AS) is an important mechanism in the regulation of gene expression by excising introns and ligating different exons to produce multiple mRNA isoforms from a single gene. This post-transcriptional process greatly enhances transcriptome and proteome complexity [1,2]. In humans, pre-mRNAs from >95% protein-coding genes are alternatively spliced to produce mature mRNAs [3,4]. Of the total intron-containing genes, >60% and >48% undergo AS in *Arabidopsis* and rice, respectively [5,6]. In addition, AS plays a key role in the life process by modulating the gene expression in development [7–11]. Mutations in AS may result in a wide range of diseases in humans [1,12]. In plants, aberrant AS may affect their growths and defense responses [13–16].

There are five different types of AS, including exon skipping, intron retaining, mutually exclusive exons, alternative 5' splice site and 3' splice site selection [3,6]. In vertebrates, exon skipping is the most frequent type, whereas intron retention is the most common event in plants [17]. Two elements are necessary for AS: 1) cis-acting elements, a specific RNA sequence often found in exons or introns (ESE/ESS, exon splicing enhancer/silencer; ISE/ISS, intron splicing enhancer/silencer), and 2) the trans-acting elements [7,10], proteins which promote the joining of exons.

Pre-mRNA splicing takes place in a large RNA-protein complex known as spliceosome, composed of five small nuclear ribonucleoprote in particles (U1,U2,U4/U6,U5 snRNPs) and a large number of non-snRNP proteins, including serine/arginine-rich (SR) proteins [18–20]. The interactions between SR proteins and snRNPs are important for splicing. In mammals, it is accepted that SC35 and SF2/ASF interact with both U1-70K and U2AF35, the subunits of U1 and U2 snRNPs respectively, thereby playing a role in the selection of 5' and 3' splice sites [21]. In *Arabidopsis*, SRZ21, SRZ22 and SCL33 were also found to interact with U1-70K [22,23]. SR proteins contain one or two RNA recognition motifs (RRM) at the N-terminal domains and serine/arginine-rich (RS) domain at the C-terminal [8,24–30]. The two different domains have disparate functions, with the RRM domain interacting with pre-mRNAs and the RS domain regulating the protein-protein interactions [24,29,31,32]. The RS domain can influence protein subcellular localization by modulating its phosphorylation [33]. The SR proteins are dynamic [34], and they assemble in nuclear speckles at the subcellular level [34–38]. SR proteins participate in many processes, including mRNA export [39,40], maintenance of genome stability [41,42], microRNA processing [43] and transcription [44]. There are seven SR proteins in humans (SF2/ASF, SC35, SRp20, SRp75, SRp40, SRp55 and 9G8) [27,45]. *In vivo*, transcription and RNA processing are coupled [46–49], and SC35 was found to participate in transcription in animals, and influences transcriptional elongation by regulating the functions of RNA polymeraseII (RNAPII) [44,50,51]. Deletion of the ASF/SF2 or SRp20 resulted in cell and embryo lethality [41,52,53]. Mutation of ASF/SF2 may lead to cancer [54,55], and disruption of SC35 leads to a heart disease [56]. Plants have a much higher number of SR proteins with 18 in *Arabidopsis* and 24 in rice [15,57]. In *Arabidopsis*, the total SR proteins are divided into six subfamilies: SR (ASF/SF2-like, SR34, SR34a, SR34b, and SR30), RSZ (9G8-like, RSZ21, RSZ22, and RSZ22a), SC (ortholog of SC35), SCL (SC35-like, SCL28, SCL30, SCL30a, and SCL33), RS (RSp31, RSp31a, RSp40, and RSp41), and RS2Z (RS2Z32, and RS2Z33). Among these six subfamilies, the latter three are specific to plants [15]. SR proteins play important roles in plant development. Overexpression of *atSRp30* affects the splicing and growth of plants, resulting in late flowering, reduced apical dominance, and larger flowers and rosette leaves. Overexpression of *atRS2Z33* leads to an increased number of embryos, thicker hypocotyl and cotyledons, altered shapes of root hairs and trichromes, and elevated cell size [58,59]. SR45, which contains one RRM in the middle and two RS domains with each in the N-terminal and C-terminal respectively, is therefore not a classical SR protein. *Sr45-1*, a transfer DNA (T-DNA) insertion mutant, exhibits abnormal phenotypes, including late flowering time, narrow leaves, reduced root growth and an altered number of petals and stamens [60]. Recently, SR45 was found to be involved in RNA-direct DNA methylation (RdDM), however, the detailed mechanism is not clear [61]. Compared with the studies on SR proteins in vertebrates, the functions of SR proteins in plants remain poorly understood.

Here we analyzed the functions of six subfamilies of *Arabidopsis* SR proteins using a genetic approach. We addressed the molecular basis for the functions of SC35 and SC35-like proteins. *Arabidopsis* SC35 is an ortholog of human SC35 splicing regulator, containing a RRM domain and a RS domain in its N- and C-terminal, respectively [62]. The four SCL proteins in *Arabidopsis* all contain a RRM domain and a RS domain in the N- and C-terminal, respectively. In addition, SCL proteins contain an N-terminal domain rich in arginine, proline, serine, glycine and tyrosine [15,62]. We identified polytropic defects of the *sc35-scl* mutant (*scl28 scl30 scl30a scl33 sc35*) in plant development, including delayed flowering time, serrated leaves, shorter root length and abnormal silique phyllotaxy. In the *sc35-scl* mutant, 213 genes were found to show significant changes in AS, including all the common AS patterns. In addition, the expression levels of 1249 genes were altered in the *sc35-scl* mutant. Therefore, our findings demonstrated that SC35 and SCL proteins regulate plant development in a redundant manner by modulating splicing and transcription of a subset of genes.

## Results

### SC35 and SCL proteins are required for plant development

To study the function of SR proteins, we identified single null mutants of 17 SR protein genes: *scl28*, *scl30*, *scl30a*, *scl33*, *sc35*, *sr30*, *sr34*, *sr34a*, *sr34b*, *rs31*, *rs31a*, *rs40*, *rs41*, *rsz21*, *rsz22*, *rs2z32*, and *rs2z33*. Under a long-day condition, we observed no obvious morphological alterations of these single null mutants. To study the functional relationships between these SR proteins, we generated multiple mutants of different families of SR proteins according to their phylogenetics and structures by crossing the related single T-DNA insertion mutant in addition to clustered regularly interspaced short palindromic repeats/ CRISPR associated proteins 9 (CRISPR/Cas9)-mediated mutagenesis [63]. We first generated *sr* (*sr34 sr34a sr34b sr30*) and *rs* quadruple mutant (*rs31 rs31a rs40 rs41*). Given that RSZ and RS2Z subfamilies containing one or two Zn knuckles in the middle of the RS and RRM domains are evolutionarily related [25], and that SCL proteins have a similar structure to that of SC35 [15,62] (S1 Fig), we generated the *rsz-rs2z* quintuple mutant (*rsz21 rsz22 rsz22a rs2z32 rs2z33*) and the *sc35-scl* quintuple mutant (S2 Fig) to analyze the functions of these subfamilies. Apart from the *sc35-scl* quintuple mutant (*scl28 scl30 scl30a scl33 sc35*), we observed no visible phenotypes of the *sr* quadruple mutant, *rs* quadruple mutant, and *rsz-rs2z* quintuple mutant (S3 Fig).

**Fig 1:**
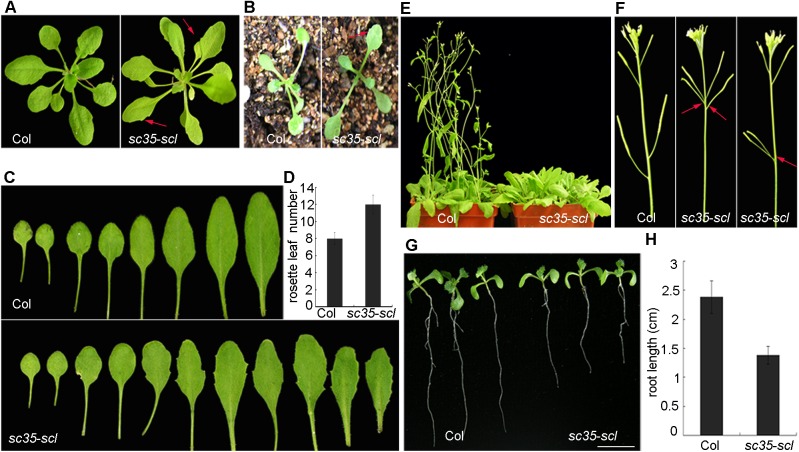
Phenotypes of Plants with Loss-of-function of SC35 and SCL Proteins Compared with Those of WT. (**A**) Phenotypes of WT and *sc35-scl* quintuple mutant plants grown 20 d after vernalization. (**B**) Phenotypes of WT and *sc35-scl* quintuple mutant plants grown 12 d after vernalization. (**C**) Rosette leaves of WT and *sc35-scl* quintuple mutant plants. (**D**) The rosette leaf numbers of WT and *sc35-scl* quintuple mutant plants began bolting. Numbers are shown as means ± SD (n = 60). (**E**) Phenotypes of WT and *sc35-scl* quintuple mutant plants grown for 30 d after vernalization under a photoperiod (16 hours light, 8 hours dark). (**F**) Silique phyllotaxy of WT and *sc35-scl* quintuple mutant. (**G**) Phenotypes of WT and *sc35-scl* quintuple mutant seedlings grown vertically on the plate for 10 d. Bar = 0.5cm. (**H**) Statistics of root lengths of WT and *sc35-scl* quintuple mutant plants grown vertically on a plate for 10 d. Error bars represent SDs (n = 70).

Compared with the wild-type (Col-0; WT), the *sc35-scl* mutant is characterized by serrate rosette leaves, appearing as early as at the first euphylla (Fig 1A–1C). In addition, several smaller rosette leaves were observed during the late vegetative stage. Under a long-day (16h: 8h light: dark) condition, the *sc35-scl* mutant displayed a delayed flowering phenotype (Fig 1E), with approximately four more rosette leaves than WT (Fig 1C and 1D), and an altered phyllotaxis arrangement (Fig 1F). The roots of the quintuple mutant are shorter in comparison to those of WT seedlings (Fig 1G and 1H). To generate the *sc35-scl* mutant, we obtained several double, triple and quadruple mutants of the *SC35* and *SCL* genes. These double (*scl28 scl30*, *scl28 scl33*, *scl28 sc35*, *scl30 scl33*, *scl30 sc35*, *scl30a scl33*, *scl30a sc35*, and *scl33 sc35*) and triple mutants (*scl28 scl30 scl30a* and *scl30 scl30a scl33*) have no obvious phenotypic alteration, only the quadruple mutant (*scl28 scl30 scl30a scl33)* mutant shows mild phenotypic changes, including serrated rosette leaves and late-flowering (S4 Fig). Considered together, these results suggested that the SC35 and SCL proteins play a redundant role in plant development.

### SC35 and SCL proteins colocalize in nuclear speckles and interact with U1-70K and U2AF65a

**Fig 2:**
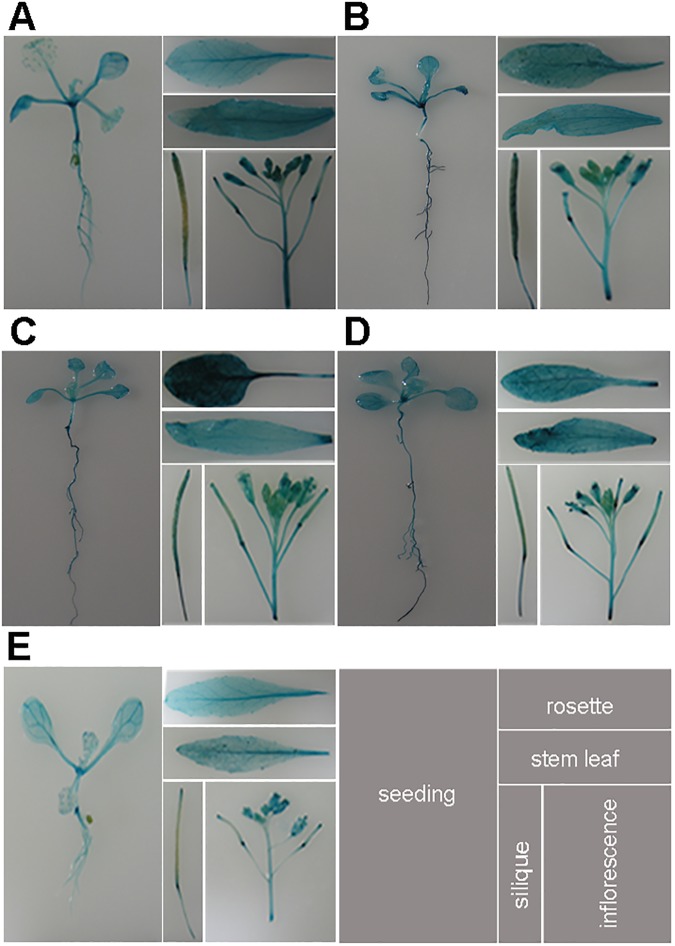
The Expression Patterns of SC35 and SCL Proteins in the Seedling and Tissues. Illustrated are GUS-staining of 12 d seedlings, rosette leaves, stem leaves, siliques, and inflorescences. (**A**) Histochemical localization of *SCL28* promoter-*GUS* activity in transgenic plants. (**B**) Histochemical localization of *SCL30* promoter-*GUS* activity in transgenic plants. (**C**) Histochemical localization of *SCL30a* promoter-*GUS* activity in transgenic plants. (**D**) Histochemical localization of *SCL33* promoter-*GUS* activity in transgenic plants. (**E**) Histochemical localization of *SC35* promoter-*GUS* activity in transgenic plants.

To elucidate the expression patterns of SC35 and SCL proteins, we fused the upstream regulatory sequences of *SC35*, *SCL28*, *SCL30*, *SCL30a* and *SCL33* to the β-glucuronidase (*GUS*) reporter gene and transformed these fusions into *Arabidopsis* plants. For each fusion protein, three independent transgenic lines were selected to analyze the GUS staining in seedlings and different tissues of adult plants. We observed overlapping expressions of the five SR proteins, which widespread in seedlings, rosettes, stem leaves, siliques and flowers (Fig 2), implicating that the five SR proteins have ubiquitous functions in plant growth.

**Fig 3:**
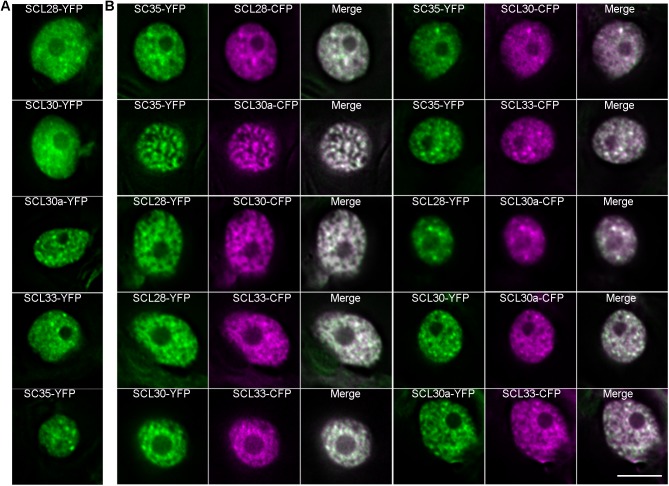
SC35 and SCL Proteins Co-localize with Each Other In Nuclear Speckles. (**A**) Distributions of SCL28-YFP, SCL30-YFP, SCL30a-YFP, SCL33-YFP, and SC35-YFP in nuclear speckles. (**B**) Co-localization of SCL28, SCL30, SCL30a, SCL33 and SC35 in nuclear speckles. Bar = 10μm.

To address the subcellular distributions of SC35 and SCL proteins, these proteins were fused to yellow fluorescent protein/cyan fluorescent protein (YFP/CFP), and transiently expressed or coexpressed in tobacco leaves. SC35 and SCL proteins were observed to form nuclear speckles (Fig 3A) and colocalize in these subnuclear domains (Fig 3B).

**Fig 4:**
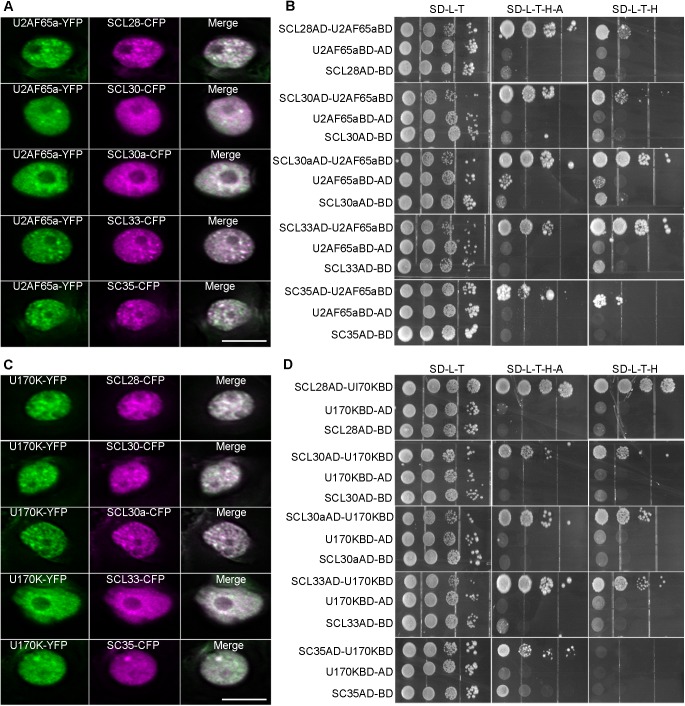
Colocalizations and Interactions between U2AF65a/U1-70K and SC35 /SCL Proteins. (**A**) Colocalizations between U2AF65a and SC35/SCL proteins in nuclear speckles. Bar = 10μm (**B**) Yeast-two hybrid assays indicated the interactions between U2AF65a and SC35/SCL proteins on the selective medium SD-Trp-Leu, SD-Trp-Leu-His-Ade and SD-Trp-Leu-His+ 5mM 3-AT (3-amino-1, 2, 4-triazole). (**C**) Colocalizations between U1-70K and SC35/SCL proteins in nuclear speckles. Bar = 10μm (**D**) Yeast-two hybrid assays indicated the interactions between U1-70K and SC35/SCL proteins on the selective medium SD-Trp-Leu, SD-Trp-Leu-His-Ade and SD-Trp-Leu-His+ 5mM 3-AT (3-amino-1, 2, 4-triazole).

To elucidate the relationships between U2 snRNP and SC35/SCL proteins, YFP-tagged U2AF65a and CFP-fused SC35/SCL proteins were transiently coexpressed in tobacco leaves. Colocalization between U2AF65a and SC35/SCL proteins was observed in nuclear speckles (Fig 4A). In addition, yeast two-hybrid assays revealed the interactions between U2AF65a and SC35/SCL proteins (Fig 4B). To study the relationships between U1 snRNP and SC35/SCL proteins, YFP-tagged U1-70K [64] and CFP-fused SC35/SCL proteins were transiently coexpressed in tobacco leaves. Colocalization between U1-70K and SC35/SCL proteins was observed in nuclear speckles (Fig 4C). Similarly, we observed the interactions between U1-70K and SC35/SCL proteins as indicated by yeast two-hybrid assays (Fig 4D).

### Depletion of SC35 and SCL proteins affects the splicing patterns of a population of genes

**Fig 5:**
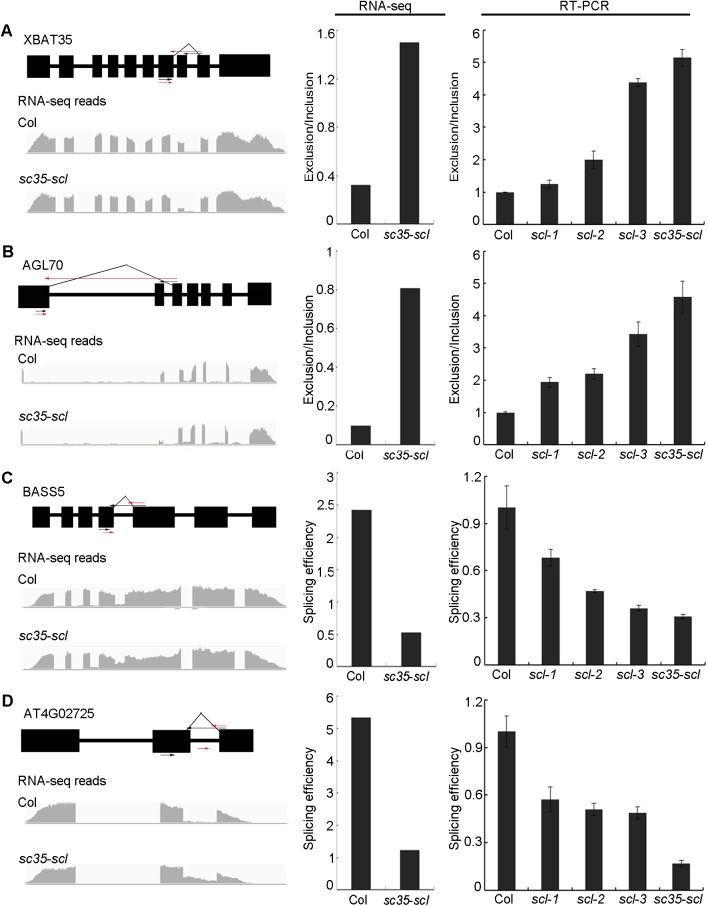
SC35 and SCL Proteins Regulate the Alternative Splicing of *XBAT35*, *AGL70*, *BASS5*, and *AT4G02725*. (**A**)SC35 and SCL proteins regulate the exon skipping splicing event of *XBAT35*. (**B**) SC35 and SCL proteins regulate the exon skipping splicing event of *AGL70*. (**C**)SC35 and SCL proteins regulate the intron retained splicing event of *BASS5*. (**D**)SC35 and SCL proteins regulate the intron retained splicing event of *AT4G02725*. The diagram shows the gene structures and the RNA-seq reads on the left. Black boxes represent exons. The positions of arrows represent the primers used to analyze the splicing efficiency. In (**A**) and (**B**), the black arrows represent primers used to analyze the normal splicing pattern (unskipped exon), the red arrows represent the primers used to detect the exon skipping events, and the 3’ primers were designed in the different exon-exon junctions. In (**C**) and (**D**), The black arrows represent primers used to analyze the normal splicing pattern and the red arrows represent primers to detect the unspliced pattern (intron-retained), the 3’ primers were designed in the exon-intron junctions. The right panel shows the splicing efficiencies of the corresponding genes. The bar charts show the statistical data from RNA-sequencing (left) and RT-PCR results (right). *Scl-1*: *scl33 scl30a* double mutant; *scl-2*: *scl33 scl30a scl30* triple mutant; *scl-3*: *scl33 scl30a scl30 scl28* quadruple mutant. The splicing efficiencies were calculated as the level of spliced events normalized to unspliced events and the Exclusion/Inclusion (exclusion/inclusion ratio) calculated as the level of exon-skipped events normalized to exon-unskipped events. Values are shown as mean± SEM from three biological repeats.

As SC35 and SCL proteins interact with subunits of U1 and U2 snRNPs, we then investigated the global AS of the *sc35-scl* quintuple mutant. To this end, we applied high-throughput sequencing to analyze mRNAs in 12 d old plants of the mutant using WT as a control. Two distinct groups of RNA-seq data from three biological repeats were formed in the hierarchical clustering (S5A and S5B Fig). The reads mapped to the genome and aligned against splice junctions were summarized in S1 Table. Compared to WT, the 10114 splicing events were found to be changed in the *sc35-scl* quintuple mutant, including exon skipping, intron retained, alternative 3’ splice site, alternative 5’ splice site, alternative start, and alternative end (S6B Fig). Among these genes with altered splicing, 213 genes have the significant alternation (p<0.05) of the splicing pattern. Real-time reverse transcription polymerase chain reactions (RT-PCRs) of several genes (Fig 5) were applied to validate sequencing data by comparing the exclusion/inclusion ratio (the ratio of skipped events to unskipped events) and splicing efficiencies between WT and *sc35-scl* mutant (the ratios of spliced RNA to unspliced RNA) [65,66]. For these genes, the splicing results revealed by RT-PCRs are consistent with those from RNA-sequencing data (Fig 5; S7 Fig). The results of semi-quantitative reverse transcription and polymerase chain reaction (qRT-PCR) were observed to be consistent with the RNA-sequencing data (S8 Fig). In addition, the alternative splicings of representative genes also change in double, triple, and quadruple mutants of SC35/SCL proteins, however, the changing rates increase from double, triple, quadruple to quintuple mutants of *SC35* and *SCL* genes (Fig 5; S7 Fig), supporting that the proper splicing of these genes depend on the interactive roles of the five SC35/SCL proteins.

**Fig 6:**
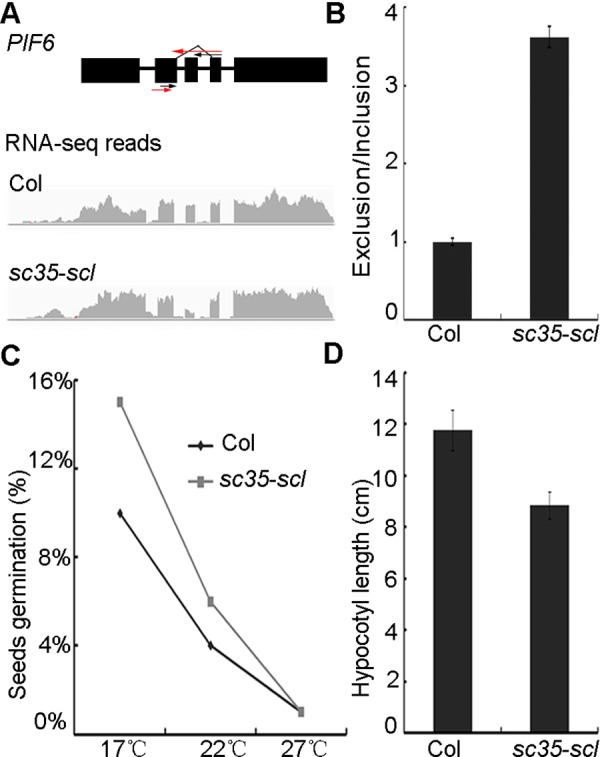
SC35 and SCL Proteins Regulate the Alternative Splicing of *PIF6*. (**A**) The structure and RNA-sequencing reads of *PIF6* in WT and *sc35-scl* mutant. The black arrows represent primers used to analyze the normal splicing pattern and the red arrows represent the primers used to detect the exon skipping events. (**B**) The splicing of *PIF6* in WT and *sc35-scl* mutant. (**C**) The germination rates of freshly harvested seeds of WT and *sc35-scl* mutant at three ambient temperatures. Statistical data were obtained 7 d after sowing on the plates. Data are given as means ± SD (n = 1,100). (**D**) Statistical data of hypocotyl lengths of WT and *sc35-scl* mutant seedlings grown under red-light for 4 d after vernalization. Data are shown as means ± SD (n = 70).

The different isoforms of a specific gene may play disparate functions in special development stages or different tissues [65,67–69]. We investigated the functions of different splicing isoforms of the *PIF6* gene to further validate the RNA-sequencing data. PIF6 is a transcription factor containing an active photochromo-binding motif and a bHLH-heterodimerization domain, and plays a role in seed germination [67,70]. PIF6 has two distinct splicing isoforms: PIF6-α and PIF6-β. PIF6-β lacks the bHLH-heterodimerization domain, and therefore loses the ability to interact with DNA or other proteins. Overexpression of PIF6-β influences seed dormancy and hypocotyl length under red light, and results in an increased rate of seed germination and decreased hypocotyl growth under red light [67]. The real-time RT-PCR indicated that efficiency of the third exon skipping increases approximately 3.5 fold in the mutant compared with that of WT (Fig 6A and 6B), resulting in the elevation of the PIF6-β isoform. Accordingly, the seed germination rate of the *sc35-scl* mutant is higher (Fig 6C), and hypocotyl length of the quintuple mutant is shorter than that of WT under red light (Fig 6D).

### SCL30 binds to a specific RNA motif

**Fig 7:**
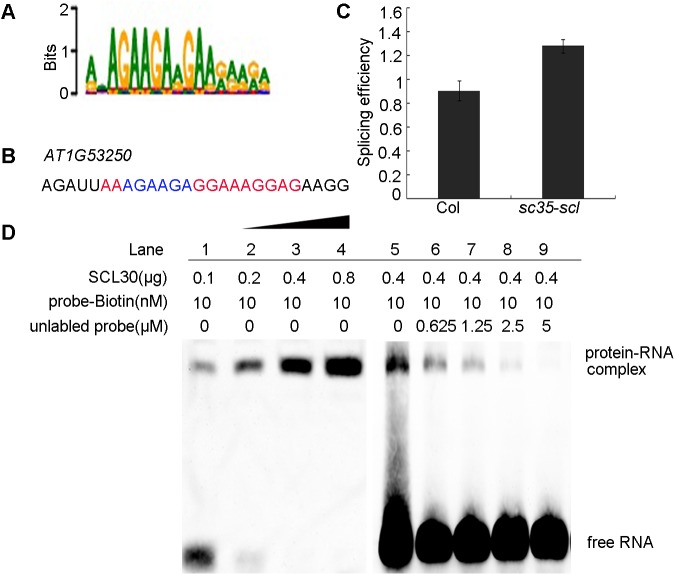
SCL30 Binds to a Specific RNA Sequence. (**A**) The sequence of a specific motif obtained by XX motif analysis. (**B**) The RNA motif in *AT1G53250*. (**C**) Splicing efficiency of *AT1G53250* in WT and *sc35-scl* mutant. (**D**) RNA EMSA assay indicated the binding of SCL30 to the RNA motif in (**B**).

To clarify the molecular mechanism for the role of SC35 and SCL proteins in the regulation of splicing, using the exhaustive evaluation of the matriX motifs (XX motif) method, we identified a specific SC35/SCL protein-binding sequence containing a short AGAAGA motif (Fig 7A), and the splicings of several genes with this motif were confirmed by real-time RT-PCR, consistent to RNA-seq data (S9 Fig). To investigate whether SC35 and SCL proteins directly interact with this motif, we used RNA electrophoretic mobility shift assays (EMSAs) to test the interaction between a purified SCL protein and the Biotin-labeled RNA motif. We examined the binding of SCL30 to the RNA fragment of *AT1G53250* locus, which was randomly selected from genes containing this short AGAAGA motif (Fig 7B). The AS pattern of *AT1G53250* was confirmed by real-time RT-PCR (Fig 7C; S9 Fig). As shown in Fig 7D, the amount of RNA-protein complex increases proportionally to the increase of purified SCL30. In contrast, the amount of RNA-protein complex decreases as the unlabeled probe increases (Fig 7D). Finally, the unlabeled probe completely abolished the binding of labeled probes (Fig 7D, lane 9), indicating that SCL30 binds directly to this specific RNA sequence. To further verify the specificity of the binding of SCL30 to this RNA sequence, we mutated the AGAAGA to UCUUCU (S10A Fig), and performed a competition assay. The results showed that the mutated probe failed to compete the binding of SCL30 to the un-mutated probe (S10B Fig), further supporting that the binding of SCL30 to the RNA sequence is specific.

### Depletion of SC35 and SCL proteins affects the transcription of a subset of genes

**Fig 8:**
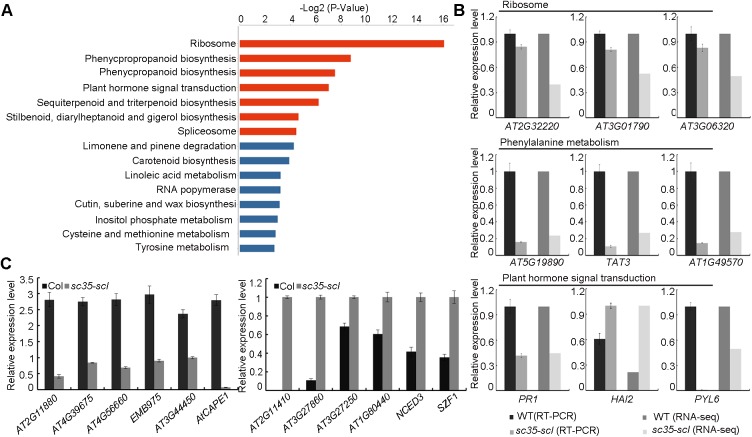
Loss-of-function of SC35 and SCL Proteins Affects Gene Expression. (**A**) Pathways of the differential genes between WT and *sc35-scl* mutant (p<0.05). (**B**) Validation of the selected genes with altered expression in the *sc35-scl* mutant by RT-PCR. The bar charts show the statistics data from RT-PCR results and RNA-sequencing. Values are shown as the mean± SEM from three biological repeats. (**C**) Validation of the selected genes without introns in their coding regions and with altered expression in the *sc35-scl* mutant by RT-PCR. Values are shown as the mean± SEM from three biological repeats.

By comparing the RNA-seq data of WT and *sc35-scl* mutant, we noticed that there were 1249 differentially expressed genes (fold change>1.5, p<0.05); the expressions of 720 and 529 genes increased and decreased, respectively (S2 Table). These genes of altered expression are involved in different biological processes, including genes involved in the ribosome, phenylalanine metabolism, plant hormone signal transduction, and spliceosome (Fig 8A). We verified the expressions of key genes involved in the ribosome, phenylalanine metabolism, and plant hormone signal transduction by real-time RT-PCRs in seedlings of WT and *sc35-scl* mutant (Fig 8B). Importantly, the expressions of a group of genes without intron also changed (Fig 8C), raising a possibility that SC35 and SCL proteins might play a role in the regulation of transcription. As both transcription and degradation of mRNA affect the accumulation level of the mRNA, we compared the transcript decay rates of several genes without intron between WT and *sc35-scl* to uncover the role of SC35/SCL proteins in the transcriptional regulation. To this end, cordycepin, a drug which is structurally analogous to adenosine, was used to inhibit the transcription and followed by the evaluation of mRNA decay [71–73] using *EIF-4A* transcript, which is stable and has a prolonged half life, as a control [73,74] (S11 Fig). The results showed that the mRNA decay efficiencies of some genes in *sc35-scl* were similar to those in WT (S12 Fig), supporting a role of SC35 and SCL proteins in the transcriptional regulation of a population of genes in a direct or indirect manner.

### SC35 and SCL proteins interact with a subunit of RNA polymerase II

**Fig 9:**
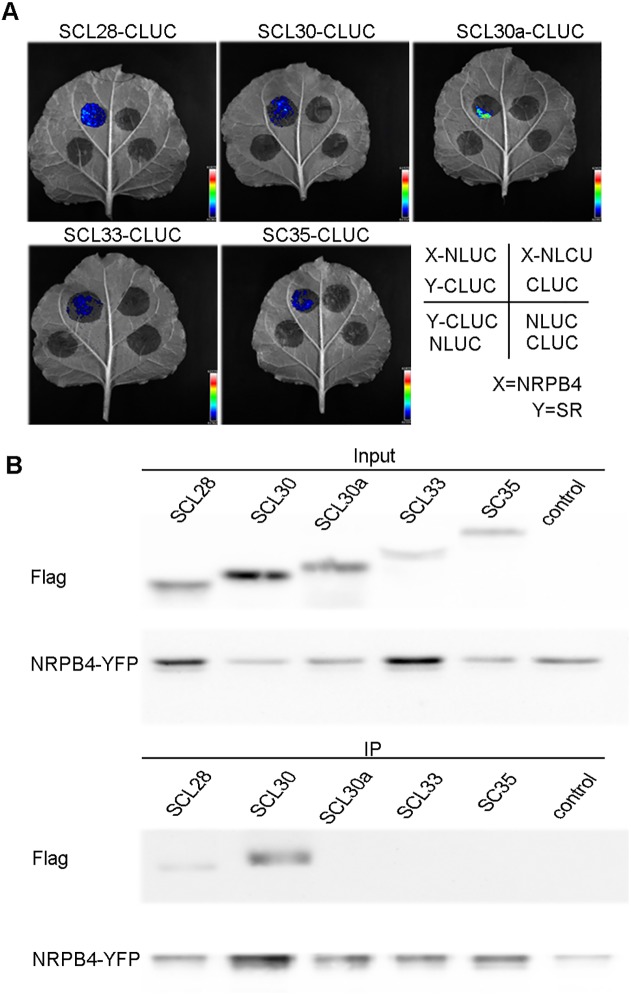
SC35 and SCL Proteins Interact with NRPB4. (**A**) Firefly luciferase complementation imaging assay shows the interactions between NRPB4 and SC35/SCL proteins. The NRPB4-NLUC and SR-CLUC were co-expressed in the tobacco leaves and the fluorescence signals were visualized 48 h after inoculation. (**B**) Co-IP assays show the interactions between NRPB4 and SCL28/SCL30 proteins. IP, immunoprecipitation.

In animals, it was known that SR proteins interact with RNA polymerase II (RNAP II) [75–77], and that deletion of the SR proteins attenuates the production of nascent RNAs [44]. Firefly luciferase complementation imaging assays were performed to address the potential interactions between plant SC35/SCL proteins and NRPB4, a specific subunit of RNAP II [69]. NRPB4 was fused to CLUC, and the five SR proteins were fused to NLUC. CLUC/NLUC pairs of constructs were transiently coexpressed in tobacco epidermal leaf cells, and complemented luciferase signals were observed between NRPB4 and SC35/SCL proteins (Fig 9A), indicating that SC35 and SCL proteins interact with NRPB4. These interactions between NRPB4 and SC35/SCL proteins were further tested by the Co-IP assays. Flag-fused SCL28 and SCL30 were observed to co-immunoprecipate with YFP-fused NRPB4 (Fig 9B), but not with SCL30a, SCL33, and SC35, possibly due to weak interactions between NRPB4 and them.

### SC35 and SCL proteins regulate flowering by modulating splicing and transcription of *FLC*

**Fig 10:**
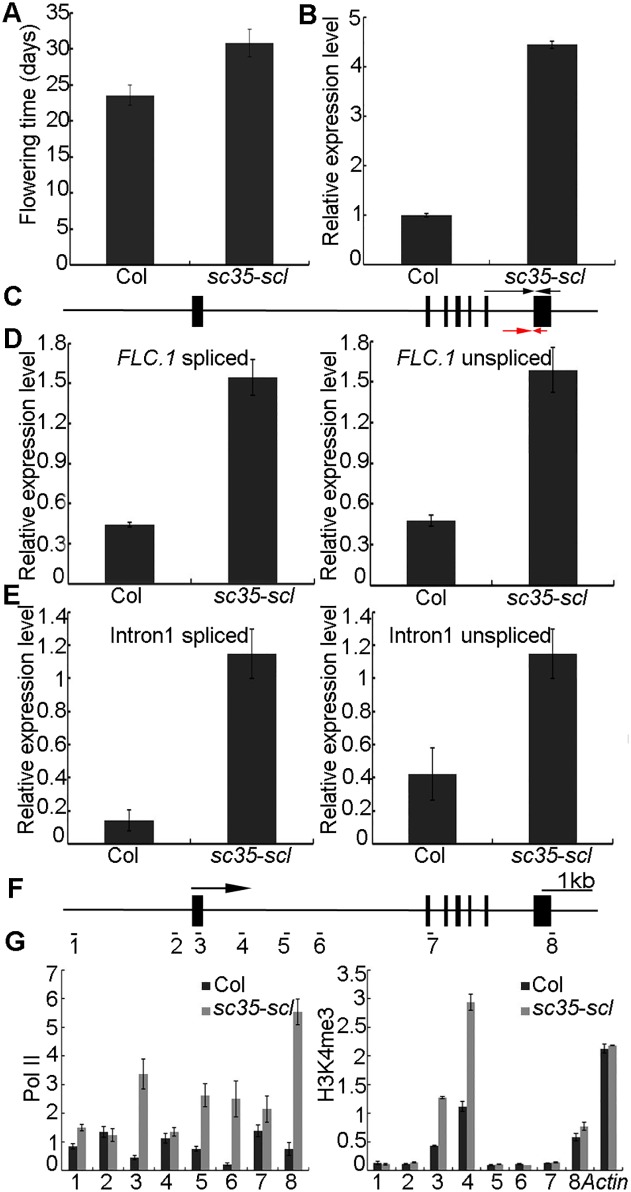
SC35 and SCL Proteins Regulate *FLC* Splicing and Transcription. (**A**) The flowering time of WT and *sc35-scl* mutant plants under long-day condition. Error bars represent SD (n = 60). (**B**) The level of *FLC* mRNA of WT and *sc35-scl* mutant as examined by real-time RT-PCR. Values are shown as the mean± SEM from three biological repeats. (**C**) Schematic representation of the structure of *FLC*. Black boxes represent exons. The arrows represent the positions of primers used to analyze the splicing efficiency. The black arrows are primer sused to analyze the splicing pattern and the red arrows are the primers to investigate the unsplicing event. The forward primers were designed on the different exon-exon junctions. (**D**) The splicing events of the last intron of the *FLC* gene in WT and *sc35-scl* mutant as examined by real-time RT-PCR. The bar graphs represent the spliced *FLC*.*1* in the left panel, and the unspliced *FLC*.*1* on the right panel. Values are shown as the mean± SEM from three biological repeats. (**E**) SC35/SCL proteins repress the efficient splicing of *FLC* intron 1. The bar graphs represent the spliced intron 1 on the left panel and unspliced intron 1 on the right panel. Values mean± SEM from three biological repeats. (**F**) Schematic representation of the structure of *FLC*. Black boxes represent exons. The numbers indicate the positions of primer pairs used for CHIP-PCR. The arrow shows the transcription start site. (**G**) Quantification data of the ChIP results. ChIP-PCR assays were used to analyze the Pol II enrichment at *FLC* which was presented as ratio of (Pol II *FLC*/input *FLC*) to (Pol II *Actin*/input *Actin*) and H3K4me3 enrichment at *FLC* given as ratio of (H3K4me3 *FLC*/input *FLC*) to (H3 *FLC*/input *FLC*), *actin* was used as an internal control for the CHIP experiments. Values mean± SEM from three technical repeats. ChIP assays were repeated three times with similar results.

An obvious phenotype of the *sc35-scl* quintuple mutant is late-flowering. Under the long-day condition, plants of the *sc35-scl* mutant bolt at approximately 30 d after sowing, whereas WT plants at approximately 23 d (Fig 1E; Fig 10A). In the quintuple mutant from the RNA-seq data, we found that the expression of *Flower Locus C* (*FLC*), which encodes a MADS-box DNA binding protein, a key regulator of flowering time in *Arabidopsis* [78,79], increases significantly compared with that of the WT (S2 Table). Real-time RT-PCR further confirmed that loss of functions of SC35 and SCL proteins resulted in a sharp increase of *FLC* (Fig 10B). In *Arabidopsis*, there are four different splicing isoforms of *FLC*: *FLC*.*1*, *FLC*.*2*, *FLC*.*3* and *FLC*.*4*. Among them, the isoform of *FLC*.*1* encodes a functional FLC protein and has an abundant expression in *Arabidopsis* [66]. We first investigated the splicing of the last intron by real-time RT-PCR and found that the proportions of spliced to unspliced introns were identical in WT and *sc35-scl* mutant (the splicing efficiencies are 0.917 and 0.973 in WT and *sc35-scl*, respectively) (Fig 10C and 10D), indicating that the SC35 and SCL proteins have no effect on AS of *FLC* transcripts, consistent with the RNA-seq data (S3 Table). We then addressed whether the constitutive splicing of *FLC* has changed. To this end, we examined the splicing of the first intron of *FLC* by real-time RT-PCR and found that the proportion of spliced to unspliced transcript increases in the *sc35-scl* quintuple mutant compare with that in WT (the splicing efficiency is 0.331 and 1 in WT and *sc35-scl*, respectively) (Fig 10E), suggesting that SC35 and SCL proteins regulate the splicing efficiency of the first intron of *FLC*.

The level of unspliced *FLC* transcript is higher in *sc35-scl* mutant than in WT (Fig 10D), we then asked if the elevated expression level of *FLC* is caused by not only the splicing, but also by the transcriptional regulation. To this end, we examined levels of Pol II of the *FLC* gene by chromatin immunoprecipitation (ChIP). Eight primers on different regions of the *FLC* gene were designed for the real-time RT-PCR of the immunoprecipitated samples (Fig 10F). The results showed that Pol II levels were higher in *sc35-scl* (Fig 10G). We also tested the levels of the transcription-activating mark (H3K4me3) and transcription-repressing mark (H3K27me3) at *FLC* locus by ChIP-PCR assay, the results showed that the level of H3K4me3 increases (Fig 10G), whereas the level of H3K27me3 decreases in *FLC* chromatin of the *sc35-scl* mutant compared with those in WT (S13 Fig).

## Discussion

Splicing is an important mechanism in eukaryotes, and is thought to regulate gene expression at the co- and post-transcriptional levels [80,81]. Cis-acting elements and trans-acting elements interact to regulate this process. The SR proteins are important regulators belonging to the trans-acting elements [19]. Plants have a greater number of SR proteins than animals, with *Arabidopsis* encoding 18 SRs and rice encoding 22 SRs. However, compared to the extensive studies on SR proteins in animals, studies on SR proteins in plants were very limited. In previously studies, gain-of-function has been the main tool to study the functions of plant SR proteins [58,59]. However, due to the functional redundancy and dosage-dependence of SR proteins, the loss-of-function approach is necessary to address the roles of SR proteins in development and their molecular mechanisms. Here we systematically studied different subfamilies of the classical SR proteins in *Arabidopsis* using the genetic approach. The 18 classical SR proteins in *Arabidopsis* were crossed to generate four mutants, including *sr* quadruple mutant (*sr34 sr34a sr34b sr30*), *rs* quadruple mutant (*rs31 rs31a rs40 rs41*), *rsz-rs2z* quintuple mutant (*rsz21 rsz22a rsz22 rs2z32 rs2z33*), and *sc35-scl* quintuple mutant (*scl28 scl30 scl30a scl33 sc35*). Interestingly, no visible phenotypes were observed in the *sr* quadruple mutant, *rs* quadruple mutant, and *rsz-rs2z* quintuple mutant. These observations are different from those in animals in which deletion of ASF/SF2 arrests the cell growth [52], and loss-of-function of SRp20 in the mouse leads to the embryonic lethality with the embryo failing to grow as early as in the morula stage [53]. Our results indicated that the functions of SR proteins are redundant in *Arabidopsis*. Importantly, the redundancy not only appears in the members of SR proteins of the same subfamily, but also to some degree among members from different subfamilies of SR proteins.

The loss of functions of SC35 and SCL (SC35-like, SCL28, SCL30, SCL30a and SCL33) results in pleiotropic changes in development, including serrate rosettes, late flowering, shorter roots, and anomalous phyllotaxis arrangement. At the subcellular level, SC35 and four SCL proteins co-localize in nuclear speckles. SC35 and SCL proteins were found to interact with the U1-70K and U2AF65a. In the *sc35-scl* quintuple mutant, pre-mRNA splicing of a population of genes are affected, including all events of AS with the alternative 3’ splice site the main target. In *sc35-scl*, the changed splicing isoform of *PIF6* might affect the seeds dormancy and hypocotyl elongation as we have not detected obvious changes of the expression levels of several known genes related to seeds germination (*PIF1*, *SPT*, *RBCS*, *CAB1*, *APL3*, *CH3*, *ABI1*, *ABI2*, *ABI4* and *ABI5*) [82–84] and hypocotyl elongation (*PIF3*, *PIF4* and *PIF5*) [85–88] (S14 Fig), however we cannot rule out other unknown factors which may contribute to these phenotypes. It was known that the RRM domain(s) of SR proteins have the ability to bind to the target pre-mRNA, and preferentially to specific RNA sequences [87,89–91]. We found that SCL30 binds to the AGAAGA motif, in comparison to previous studies showed that the GAAG repeats function as the splicing enhancers [92–94] in animals, and RZ-1C and SR45 proteins bind to the AG-riched motif in plant [66,95], suggesting that the AG-rich sequence might play conservative and important role in splicing both in plant and animals. It was known that most exons are bound by at least one SR protein [87,91,96,97], we thought SR45 and SC35/SCL proteins may bind to specific motifs to play redundant and cooperative roles in splicing. RZ-1C, an RNA-binding protein, might also participate in this process together with these SR proteins. We also found that the AS patterns of a set of genes without the AGAAGA motif changed in the *sc35-scl* mutant. We speculated that the AS of different genes may depend on different motifs. Alternatively, the loss of SC35 and SCL proteins may influence the functions of other SR proteins.

Among 1249 genes, of which the expression levels changed in the *sc35-scl* mutant, only 12 genes were found to have changed their splicing patterns as indicated in the sequencing data (S4 Table). The expression levels of genes can be regulated by their constitutive splicing as previously reported [66,98]; however, we found that the expression levels of many genes without introns also change with the degradation of some transcripts not changed in s*c35-scl*, suggesting a direct or indirect role of SC35 and SCL proteins in the transcriptional regulation in addition to their functions in splicing. In mammals, many studies have proved that splicing factors interact with C-terminal domain (CTD) of NRPB1 in RNAP II, or other components of transcription machinery to influence the transcription process [99,100]. We tested the potential interactions between SC35/SCL proteins and CTD of NRPB1, full-length of NRPB1 and NRPB4, two RNAP II-specific subunits, luciferase complementary signals can be only detected between SC35/SCL proteins and NRPB4 (Fig 9A). In addition, Co-IP also confirmed the interactions between NRPB4 and SC35/SCL, however no clear positive results between NRPB4 and SCL30a/SCL33/SC35 were observed in the experiment (Fig 9B). We thought the interactions between them maybe too weak to be detected under the experimental condition. We also found that SC35/SCL co-localize partially with the biggest subunit RNAP II (NRPB1) in nuclear speckles (S15 Fig), implicating that these SR proteins may exist in the large complex containing RNAP II to regulate gene expression at transcriptional and post-transcriptional levels.

The result that SC35/SCL proteins regulate both the splicing and transcription was further supported by our analysis of the expression level of *FLC* gene which increases significantly in the *sc35-scl* mutant with a later flowering phenotype. Several genes (*FPA*, *FVE*, *FY*, *VIL2*, *VRN1*, *VRN2*, and *VRN5*) involved in the regulation of *FLC* expression by the autonomous or vernalization pathway [101–104] have no obvious changes in their AS patterns, and only mild increases in their expression levels in the *sc35-scl* mutant (S2 Table; S3 Table; S16 Fig). It was known that the increases of these regulators lead to earlier flowering. We suspected that the elevated expression of *FLC* due to losses of functions of SC35 and SCL proteins might play a predominant role in the control of the later flowering phenotype of *sc35-scl* mutant. The splicing efficiency of *FLC*.*1* did not change in the quintuple mutant (Fig 10C and 10D). However, SC35 and SCL proteins suppress the constitutive splicing of the first intron of *FLC* (Fig 10E), thereby resulting in a higher expression of *FLC* in *sc35-scl*. In addition, the result that the level of unspliced *FLC* increases in *sc35-scl* suggested the regulation may occur at the level of transcription. The repression of SC35 and SCL proteins on the *FLC* transcription was further supported by the chromatin status in this locus with a decrease in H3K27me3 and increases in Pol II and H3K4me3 occupancies in the *sc35-scl* mutant.

It has been known that the *FLC* antisense RNA *COOLAIR* and chromatin modifications influence the *FLC* expression [105–107]. We found that SC35 and SCL splicing factors regulate the splicing efficiency of the first intron of *FLC* and histone methylation status at *FLC* locus, thus revealed a novel regulatory pathway in the control of expression level of *FLC* and flowering. In animals, it was found that SR proteins regulate the chromatin structure and dynamics through interaction with histones [108,109], and yeast SR-like protein Npl3 was also found to play a role in chromatin remodeling and histone modification [110]. It is of interest to address if SC35 and SCL or other plant SR proteins have roles in histone modifications or not. Future studies will focus on investigating the potential interactions between histones and SR proteins, and uncovering more functions of SR proteins in plants.

## Materials and methods

### Plant materials and growth conditions

*Arabidopsis thaliana* (ecotype Col-0), T-DNA insertion mutants *scl28* (CS853758), *scl30* (CS805508), *scl30a* (Salk_056672), *scl33* (Salk_058566), *sc35* (Salk_033824C), *sr30* (Salk_116747C), *sr34* (CS878689), *sr34a* (Salk_087841C), *sr34b* (Salk_055412), *rs31* (Salk_014656C), *rs31a* (CS834516), *rs40* (Salk_118875), *rs41* (CS803022), *rsz21* (Salk_114234), *rsz22* (CS809215), *rs2z32* (Salk_031147C), *rs2z33* (Salk_051525) were obtained from TAIR. All the mutants were confirmed by PCR (primers were listed in S5 Table).

*SR34* and *RSZ22a* were mutated by CRISPR/Cas9 [63]. T_0_ seeds were selected in Murashige and Skoog (MS) medium containing 50mg/L Hygromycin B. Homozygous transgenic lines were confirmed by PCR and sequencing.

All seeds were germinated in MS medium (1% sucrose and 0.8% agar), after vernalizing at 4°C for 3 d or 4 d. Plants were grown in a green house under a 16 h light/8 h dark photoperiod. The multiple mutants including quintuple mutant (*scl28 scl30 scl30a scl33 sc35*) and quadruple mutants (*sr34 sr34a sr34b sr30*, *rsz21 rsz22 rs2z32 rs2z33*, and *rs31 rs31a rs40 rs41*) were generated by crossing between corresponding single mutants in combination with the CRISPR/Cas9 method.

### Seed germination and flowering time measurement

A seed germination assay was performed according to the previous report [67]. Freshly harvested seeds from brown siliques were sown on the 0.8% MS plates and placed in a growth chamber under a 12 h light/12 h dark photoperiod at different temperatures. The germination rates were scored after 7 d according to the standard [67].

For the measurement of flowering times, the seeds of Col-0 and *sc35-scl* were vernalized in 4°C for 4 d and sown in the soil under LD (16 h light/8 h dark). The bolting time and the numbers of rosette leaves were scored.

### Constructs and transient expression

The cDNAs of *SC35*, *SCL28*, *SCL30*, *SCL30a*, *SCL33*, *U170K* and *U2AF65a* were amplified by PCR from Col-0 cDNAs (primers used were shown in the S5 Table), then subcloned into the plasmids pCambia1300-35S-N1-YFP and pCambia2300-35S-N1-CFP. The constructs were confirmed by sequencing and introduced into *Agrobacterium tumefaciens* (GV3101) by electroporation.

Transient expression and image processing were conducted according to the protocol [111]. For colocalization analysis, plasmid pairs SCL28-CFP/U170K-YFP, SCL28-CFP/U170K-YFP, SCL28-CFP-SC35-YFP, SCL30-CFP/U170K-YFP, SCL30-CFP/U2AF65a-YFP, SCL30-CFP/SC35-YFP, SCL30-CFP/SCL28-YFP, SCL30-YFP/SCL30a-CFP, SCL30-YFP/SCL33-CFP, SCL30a-CFP/U170K-YFP, SCL30a-CFP/U2AF65a-YFP, SCL30a-CFP/SC35-YFP, SCL30a-YFP/SCL33-CFP, SCL30a-CFP/SCL28-YFP, SCL33-CFP/U170K-YFP, SCL33-CFP/U2AF65a-YFP, SCL33-CFP/SC35-YFP, SCL33-CFP/SCL28-YFP, SC35-CFP/U170K-YFP and SC35-CFP/U2AF65a-YFP were co-expressed in tobacco leaves. After 48 h, inoculated leaf discs were visualized under a DeltaVision PersonalDV system (Applied Precision) using the Olympus UPLANAPO water immersion objective lens (60 ×/1.20 numerical apertures). The filters used for YFP were exciter (492/18 nm/nm), emitter (535/30 nm/nm), for CFP were exciter (430/24 nm/nm) and emitter (470/24 nm/nm).

### Yeast two-hybrids

For yeast two-hybrid assays, the coding regions of *SCL28*, *SCL30*, *SCL30a*, *SCL33* and *SC35* were cloned in pGBKT7 and pGADT7 plasmids (primers used were shown in S5 Table).The coding regions of *U1-70K* and *U2AF65a* were cloned in pGADT7 plasmid (primers used were shown in S5 Table). These constructs were confirmed by sequencing and cotransformed pairwisely into yeast strain AH109 according to the Pro-Quest Two-Hybrid System Manual (Matchmaker user’s manual, Invitrogen). Transformants were cultivated on the SD-Leu-Trp medium at 30°C in an incubator for approximately 3 d, and tested in selection plates: SD-Leu-Trp-His-Ala medium and SD-Leu-Trp-His medium were supplemented with 1mM or 5mM 3-amino-1, 2, 4-triazole (3-AT), respectively. The results were tested after 3–6 d of growth at 30°C.

### RNA sequencing and bioinformatics analysis

Total RNAs were extracted from 12 d seedlings of WT and quintuple mutant (*scl28 scl30 scl30a scl33 sc35*) using the RNeasy plant mini kit (Qiagen). The total RNAs were treated with DNase I followed by mRNA isolation using magnetic beads with Oligo (dT). Fragmented mRNAs were used as templates for PCR amplification and the construction of the RNA-seq library. Agilent 2100 Bioanalyzer and the ABI Step One Plus Real-time PCR System were used for the quantification and qualification of the sample library. Finally, the library was sequenced using Illumina HiSeq TM 2000.

The sequencing data termed raw reads were subjected to quality control (QC). After QC, raw reads were filtered into clean reads, and aligned to the reference sequences with SOAP aligner/SOAP2. The reads with strand direction were aligned to the TAIR10 genome using SOAP aligner/SOAP2, allowing no more than five mismatches. The ASD (AS detector) software (http://www.novelbio.com/asd/ASD/html) was used for the detection of AS events. To calculate the p-value of AS events, first count the number of junction reads that align either to the inclusion or exclusion isoforms in both the WT and *sc35-scl* quintuple mutant, and calculate a p-value using junction read-counts between WT and *sc35-scl* quintuple mutant by Fisher exact test. Then calculate read coverage for the alternative exon and its corresponding gene in both WT and *sc35-scl* quintuple mutant, and calculate a second p-value by Fisher exact test according to the alternative exon read coverage relative to its gene reads coverage between WT and *sc35-scl* quintuple mutant. Finally combine the above two p-values to obtain an adjusted p-value using a weighted arithmetic equation for assessing the statistical difference of AS between WT and *sc35-scl* quintuple mutant [112,113]. The AS events with p-value<0.05 were considered as the significant change. Using the EB-seq algorithm to analyze the differential expression genes, the standard was a fold change>1.5 or<0.667, p<0.05. Pathway analysis was used to identify the significant pathway of the differential genes according to the Kyoto Encyclopedia of Genes and Genomes (KEGG) database. We used the Fisher’s exact test to select the significant pathway. The threshold of significance was defined by the p-value and false discovery rate (FDR) [114–117].

### Histochemical GUS staining

The promoters of *SCL28*, *SCL30*, *SCL30a*, *SCL33* and *SC35* were cloned into the pBI101 plasmid (primers used were shown in S5 Table). The transgenic *Arabidopsis* plants generated using the flower dip method [118] were selected on hygromycin (50mg/L) and confirmed by PCR. At least three T_2_ independent lines were analyzed. GUS staining was performed according to published literature [119] with a modified buffer (1mg/ml 5-bromo-4-chloro-3-indolyl-b-D-glucuronic acid cyclohexylammonium salt, 50mM Na_3_PO_4_, pH 7.0, 0.1% Triton X-100, 2mM K_4_Fe(CN)_6_·3H_2_O, 2mM K_3_Fe(CN)_6_, and 10mM EDTA). The seedings and plant tissues (rosette, stem leaf, silique and inflorescence) were immersed in the GUS buffer overnight at 37°C in dark, and then cleared with 75% ethanol [120].

### RNA extraction and real-time quantitative PCR

The total RNAs were extracted from 12 d *Arabidopsis* seedlings using the RNeasy Plant Mini Kit (Qiagen). RNAs were treated with master mix reagents (Toyobo) to remove the genomic DNA, and the reverse transcription was conducted using the master mix reagents to generate the cDNAs.

The cDNAs were diluted approximately five-fold and used as a template for quantitative PCR using a SYBR Green Master Mix (Takara). Quantitative real-time PCR was performed in a Bio-Rad CFX Real-time System (primers used were shown in S5 Table). The *ACTIN2* gene was used as an internal control and for data normalization. The data obtained were analyzed through a Bio-Rad iCycleriQ Real-time Detection System, and three biological repeats were performed.

### Measurement of splicing efficiency

The splicing efficiency was measured as previously reported [66,112,121]. The splicing efficiency was calculated by determining the level of spliced RNA normalized to the level of unspliced RNA in the intron-retained splicing events, and in the exon-skipped splicing event, the exclusion/inclusion ratio was calculated by determining the level of skipped RNA normalized to the level of unskipped RNA [112]. The 3’ unspliced primers were designed crossing the intron-exon junction, and the 5’ primers were designed on the intron or the next exon. The 3’ spliced primers were designed to span the exon-exon junction. The primers related to unskipped RNA were designed to span the exon-exon junction, whereas the skipped primers were designed to span the exon with the exon after the next. These primers were then used for real-time RT-PCR. The data of the splicing efficiency were derived from three biological repeats.

### Analysis of the mRNA degradation

The seedings of WT and *sc35-scl* mutant were incubated in cordycepin, the samples were then collected at one hour intervals. Total RNA were extracted from the samples using RNeasy Plant Mini Kit (Qiagen), then cDNAs were obtained by inverse transcription. Real-time PCR was used to test the expression levels of related genes which were normalized to the expression level of corresponding genes at 0 h to evaluate the mRNA decay efficiencies.

### Motif analysis and RNA electrophoretic mobility shift assay (RNA EMSA)

The software of the XX motif was used to analyze the RNA motif [122] which is extracted from proximate the splice site of 100bp in the AS genes (p<0.05). The parameters used were described as follows: expected occurrences of motifs per sequence: zero, one or multiple occurrences; order of background model: 2; similarity threshold for merging motifs/PWMs: low; pseudocounts: 10%; number of gaps n fivemer seed: 0; start search with these seed patterns: includes 5-mer nucleotides, palindromes and tandem repeats.

We selected one sequence of approximately 26 nt (AGAUUAAAGAAGAGGAAAGGAGAAGG) from *AT1G53250*, containing the motif. The sequence was synthesized in vitro (Takara), and the 5’ end was labeled with biotin. SCL30 was cloned in the plasmid of pET28a, fused with His-tag. The constructs were expressed in *E*.*coli* (transetta) and cultured at 37°C, and the expressions of SR proteins were induced by 0.4M isopropyl β-D-1-thiogalactopyranoside (IPTG). The Ni-NTA agarose beads were used to purify SR proteins. The binding assays were performed according to the manual (Light Shit Chemiluminescent RNA EMSA Kit, Thermo Pierce). The 20μl reaction system contained 2μg tRNA, 10nM of labeled RNA and the purified individual SR protein of different concentrations. The RNA-protein mixtures were incubated for approximately 30min at room temperature and fractioned on a 6% native polyacrylamide gel under 100V for approximately 60min in 0.5x TBE buffer, then transferred to a nylon membrane (GE Healthcare). The biotin-labeled RNAs on the nylon membrane were detected using a chemiluminescent nucleic acid module (Thermo Pierce) [123]. For the competition assay, purified SR proteins, 2μg tRNA and the biotin-labeled 10nM specific RNA was added in all lanes, and unlabeled RNA was added to lanes 6–9. Lane 5, 0.4μg SR protein; lane 6, 0.4μg SR protein+0.625μM unlabeled RNA; lane 7, 0.4μg SR protein+1.25μM unlabeled RNA; lane 8, 0.4μg SR protein+2.5μM unlabeled RNA; lane 9, 0.4μg SR protein+5μM unlabeled RNA. The process was the same as that employed in the binding assay.

### Firefly luciferase (LUC) complementation image assay

The luciferase (LUC) complementation imaging assay was performed as previously described [124]. SR and NRPB4 proteins were fused to the C-terminal and N-terminal fragment of firefly luciferase, respectively. The NRPB4-NLUC and CLUC-SR were transferred into Agrobacteria strain GV3101, and then co-infiltrated into the tobacco leaves using an injection syringe. At approximately 48 h, the leaves were injected with 100mM luciferin (Sangon Biotech) in 0.1% Triton X-100, and fluorescence was quenched in the dark for several minutes, a Chemiluminescence Imaging System (Tanon) was then used to observe the luciferase signals.

### Coimmunoprecipitation (Co-IP) assay

The plasmids pairs 35S::SC35/SCL-Flag and 35S::NRPB4-YFP were co-infiltrated into the tobacco leaves using an injection syringe. At approximately 48 h, the leaves were collected and ground in liquid nitrogen. The cell debris were treated with the three volumes of extraction buffer (50mM Tris-HCl at pH 8.0, 150mM NaCl, 0.5% Triton X-100, 0.2% 2-mercaptoethanol, 5% glycerol) containing one proteinase inhibitor cocktail tablet/50 ml (Roche), centrifuged for 20 min at 8,000g [125]. The total proteins incubated with the GFP agarose beads (MBL) about 3–4 h at 4°C. The columns were washed 5 times with washing buffer (50mM Tris-HCl at pH 7.5, 100mM NaCl, 10% Glycerol, 0.05% Triton X-100, 1mM EDTA) and proteins were released by boiling the beads in SDS-PAGE loading buffer at 100°C for 10 min. The proteins were resolved by SDS/PAGE, and then the anti-Flag (Sigma) and anti-GFP (Sigma) antibodies were used to detect SC35/SCL-Flag and NRPB4-YFP, respectively.

### Chromatin immunoprecipitation (ChIP)

The ChIP assay was performed as previously described [126] using 12 d seedlings of WT and *sc35-scl* mutant. The seedlings of approximately 2.5g were harvested in cross-linking buffer (0.4M sucrose, 10mM Tris-HCl (pH8.0), 1mM PMSF, ImM EDTA, 1% formaldehyde) for 10 min using vacuum infiltration and then halted in 2M glycine. After the addition of 5μg H3K27me3, H3K4me3 (Milipore) and Pol II antibodies (Abcam) to the chromatin and incubation at 4°C overnight, the agarose beads of protein A and protein G were added and maintained at 4°C for approximately 2 h. After reverse cross-linking, DNA was purified and dissolved in 30μl water. The immunoprecipitated DNA was diluted and then quantified by real-time PCR. Real-time PCR data of Pol II were normalized to *Actin*, and H3K4me3 normalized to H3. The Pol II enrichment at *FLC* was given as ratio of (Pol II *FLC*/input *FLC*) to (Pol II *Actin*/input *Actin*) and H3K4me3 enrichment as ratio of (H3K4me3 *FLC*/input *FLC*) [106]. Primers used for real-time PCR are listed in S5 Table.

## Supporting information

S1 Fig The structures and sequence alignment of SC35 and SCL proteins in *Arabidopsis*. (A) The domains illustrating SC35 and SCL proteins. RRM, RNA Recognition Motif; RS, Serine/Arginine-rich Domain. (B) The sequence alignment of SC35 and SCL proteins.
(TIF)

S2 Fig The characterizations of *SCL28*, *SCL30*, *SCL30a*, *SCL33* and *SC35* expressions in the *sc35-scl* mutant. (A) Diagrams showing the T-DNA insertion sites of *SCL28*, *SCL30*, *SCL30a*, *SCL33* and *SC35* T-DNA insertion lines. Black boxes represent the exons. (B) The transcription levels of *SC35* and *SCL* genes in 12 d seedlings of WT and *sc35-scl* mutant. Data are shown as means ± SEM from three biological repeats of RT-PCR (left) and RNA-sequencing (right).
(TIF)

S3 Fig Phenotypes of *sr*, *rs* and *rsz-rs2z* mutants. (A) Phenotypes of 12 d seedlings of the *sr*, *rs*, and *rsz-rs2z* mutants. (B) Phenotype of 25 d plants of the *sr*, *rs*, and *rsz-rs2z* mutants.
(TIF)

S4 Fig Phenotypes of single, double, triple and quadruple mutants of SC35 and SCL proteins. (A) The phenotypes of single, double, triple and quadruple mutants of SC35 and SCL proteins compared with that of WT. No obvious visible phenotypes were observed for the single, double and triple mutants. Mildly serrated rosette leaves were observed for the *scl28 scl30 scl30a scl33* quadruple mutant. (B) The rosette leaves of WT and mutants. (C) Plants of WT and *scl33 scl28 scl30 scl30a* quadruple mutant grown for 35 d under a long day condition. The flowering time of the mutant was slightly delayed compared with that of WT.
(TIF)

S5 Fig Global evaluation of the RNA-seq data from three biological repeats. (A) Heatmap of Pearson Correlation between WT and *sc35-scl* mutant samples. (B) Hierarchical clustering between samples of WT and *sc35-scl* mutant.
(TIF)

S6 Fig Alternative splicing patterns and the splicing events affected by SC35 and SCL proteins. (A) Schematic diagrams showing the alternative splicing patterns. Black boxes represent the exons. (B) The splicing events affected by SC35 and SC35-like proteins. A total of 213 genes (p-value<0.05) with changed splicing patterns were observed from RNA-sequencing data.
(TIF)

S7 Fig SC35 and SCL proteins regulate the alternative splicing of *AtSEN1*, *AT5G47455*, *UMAMIT47* and *AT2G46915*. (A) Statistical data of the intron retained splicing of *AtSEN1* and *AT5G47455*affected by SC35 and SCL proteins. Data are from RNA-sequencing. (B) Statistical data of the intron retained splicing of *AtSEN1* and *AT5G47455* affected by SC35 and SCL proteins.*Scl-1*: *scl33 scl30a* double mutant; *scl-2*: *scl33 scl30a scl30* triple mutant; *scl-3*: *scl33 scl30a scl30 scl28* quadruple mutant. Data are from RT-PCR, Values are shown as mean± SEM from three biological repeats. (C) Statistical data of the exon skipping splicing of *UMAMIT47* and *AT2G46915* affected by SC35 and SCL proteins. Data are from RNA-sequencing. (D) Statistical data of the exon skipping splicing of *UMAMIT47* and *AT2G46915* affected by SC35 and SCL proteins.*Scl-1*: *scl33 scl30a* double mutant; *scl-2*: *scl33 scl30a scl30* triple mutant; *scl-3*: *scl33 scl30a scl30 scl28* quadruple mutant. Data are from RT-PCR, Values are shown as mean± SEM from three biological repeats.
(TIF)

S8 Fig Validation of SC35/SCL proteins-affected splicing events by qRT-PCR. (A) SC35/SCL proteins repress the exon skipping event. (B) SC35/SCL proteins repress the intron retained event. The diagram shows the gene structures of individual gene, black boxes represent exons. Arrows represent RT-PCR primers used. Quantification of the PCR products was measured using the software GIS (Gel Image System), as shown in the histogram. Values are shown as mean± SEM from three biological repeats.
(TIF)

S9 Fig SC35 and SCL proteins regulate the alternative splicing of genes containing the AGAAGA motif. (A) The splicing efficiencies of genes with the AGAAGA motif in WT and *sc35-scl* mutant as examined by RT-PCR. (B) The splicing efficiencies of genes with the AGAAGA motif in WT and *sc35-scl* mutant as examined by RNA-seq. Values were shown as the mean± SEM from three biological repeats.
(TIF)

S10 Fig The binding of SCL30 to AGAAGA motif is specific. (A) The mutated RNA probe (AGAAGA to UCUUCU). (B) The binding of SCL30 to the specific RNA sequence cannot be competed by the mutated probe in RNA EMSA assay.
(TIF)

S11 Fig The expression of *eIF-4A* in WT and *sc35-scl* mutant. (TIF)

S12 Fig Comparisons of mRNA decay rates between WT and *sc35-scl*. (A) The expression level of *AT1G56660* and *AT3G44450* upon cordycepin treatment for 0, 1, 2, 3 hours in WT and *sc35-scl* (left). The decay efficiencies of *AT1G56660* and *AT3G44450* were shown by normalization of the expression level to that at 0 h (right). (B) The expression level of *AT3G27250* and *SZF1* upon cordycepin treatment for 0, 1, 2, 3 hours in WT and *sc35-scl* (left). The decay efficiencies of *AT3G27250* and *SZF1* were shown by normalization of the expression level to that at 0 h. The level of *eIF-4A* transcript was used as a control. Values were shown as mean± SEM from three biological repeats.
(TIF)

S13 Fig Quantification data of H3K27me3 ChIP-PCR results. ChIP-PCR assay was used to analyze H3K27me3 enrichments at different positions of *FLC*, and shown as ratio of (H3K27me3 *FLC*/input *FLC*) to (H3 *FLC*/input *FLC*). *Agarmous* was used as an internal control for the ChIP experiments. Values were shown mean± SEM from three technical repeats. ChIP assays were repeated three times with similar results.
(TIF)

S14 Fig The expression levels of genes involved in seed germination and hypocotyl elongation. The transcription levels of key genes related to seed germination and hypocotyl elongation as revealed by RT-PCR. Values were shown mean± SEM from three biological repeats.
(TIF)

S15 Fig Partial colocalizations between NRPB1 and SC35/SCL proteins in nuclear speckles. Bar = 10μm.
(TIF)

S16 Fig The transcription levels of key genes involved in flowering in the autonomous and vernalization pathways as revealed by RT-PCR. Values were shown mean± SEM from three biological repeats.
(TIF)

S1 Table Summary of RNA-seq data. (DOCX)

S2 Table The genes differentially expressed in RNA-seq data. The differentially expressed genes between WT and *sc35-scl* quintuple mutant (>1.5 fold, p<0.05). Data were analyzed using the EB-seq algorithm.
(DOCX)

S3 Table The alternatively spliced genes between WT and *sc35-scl* quintuple mutantin RNA-seq data. Data were analyzed using the ASD software.
(DOCX)

S4 Table Splicing genes overlapped to the differentially expressed genes. The alternatively spliced genes (213 (p<0.05)) overlapped to the differentially expressed genes (1249). Data were analyzed using the formula VLOOKUP.
(DOCX)

S5 Table Primers used in this study. (DOCX)
