# Supplementary figures and images for: Depletion of Arabidopsis SC35 and SC35-like serine/arginine-rich proteins affects the transcription and splicing of a subset of genes
Source: PLoS Genet. 2017 Mar 8;13(3):e1006663. doi: 10.1371/journal.pgen.1006663 (PMC5362245; doi:10.1371/journal.pgen.1006663)

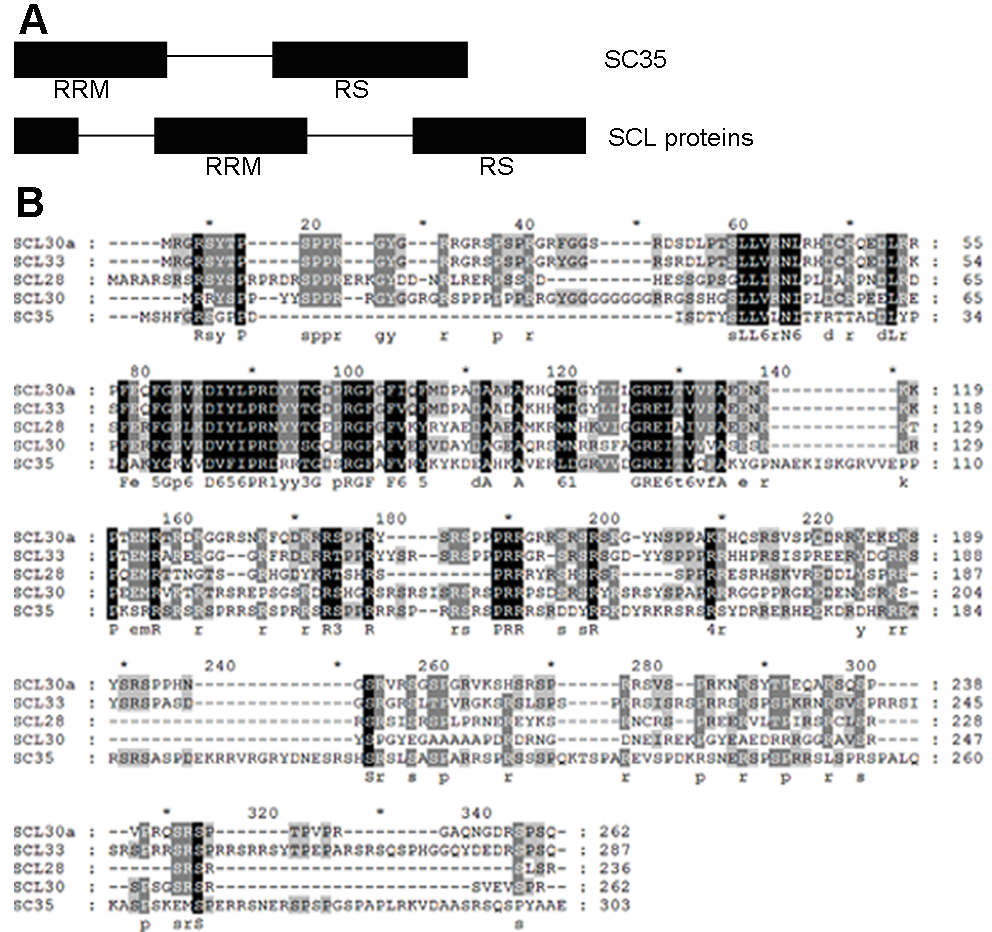

Supplement: S1 Fig — (A) The domains illustrating SC35 and SCL proteins. RRM, RNA Recognition Motif; RS, Serine/Arginine-rich Domain. (B) The sequence alignment of SC35 and SCL proteins. (TIF) [file pgen.1006663.s001.tif]

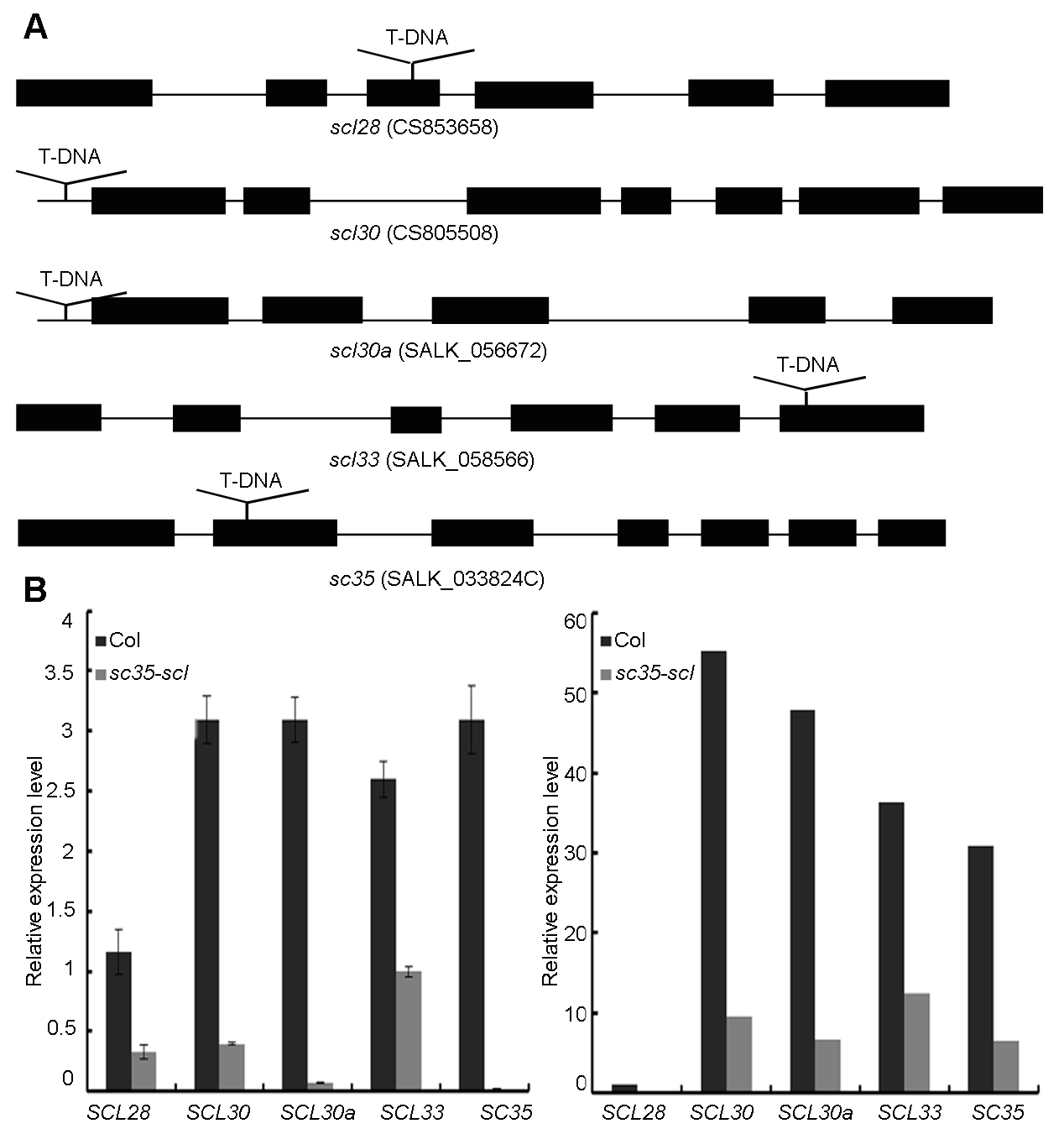

Supplement: S2 Fig — (A) Diagrams showing the T-DNA insertion sites of SCL28, SCL30, SCL30a, SCL33 and SC35 T-DNA insertion lines. Black boxes represent the exons. (B) The transcription levels of SC35 and SCL genes in 12 d seedlings of WT and sc35-scl mutant. Data are shown as means ± SEM from three biological repeats of RT-PCR (left) and RNA-sequencing (right). (TIF) [file pgen.1006663.s002.tif]

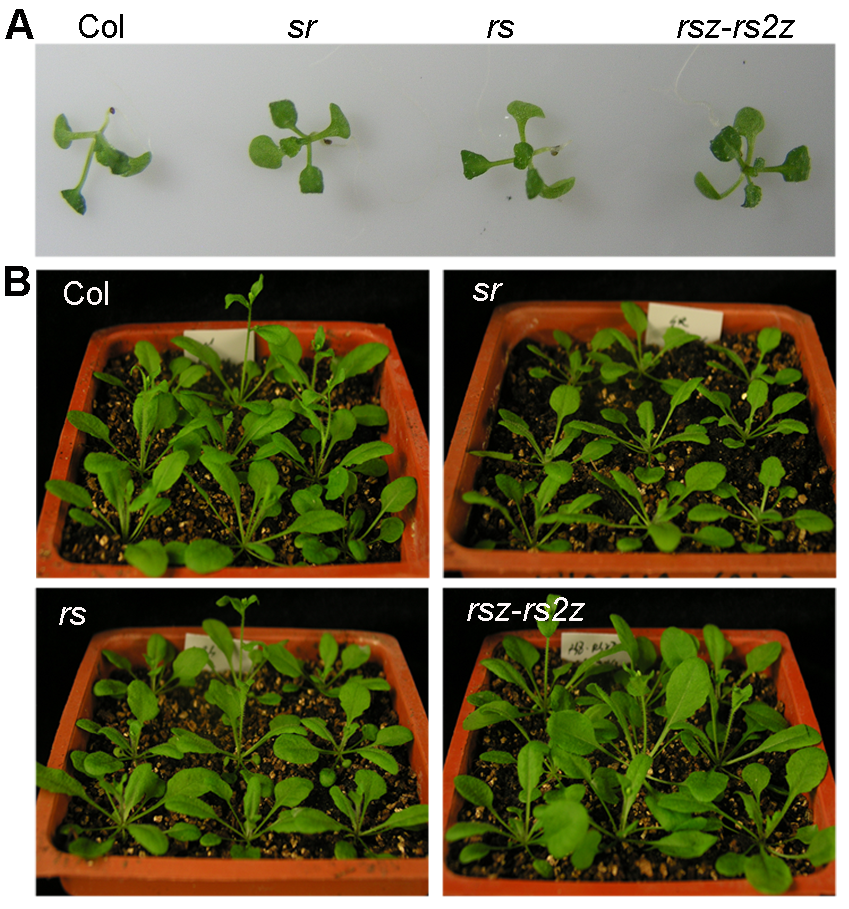

Supplement: S3 Fig — (A) Phenotypes of 12 d seedlings of the sr, rs, and rsz-rs2z mutants. (B) Phenotype of 25 d plants of the sr, rs, and rsz-rs2z mutants. (TIF) [file pgen.1006663.s003.tif]

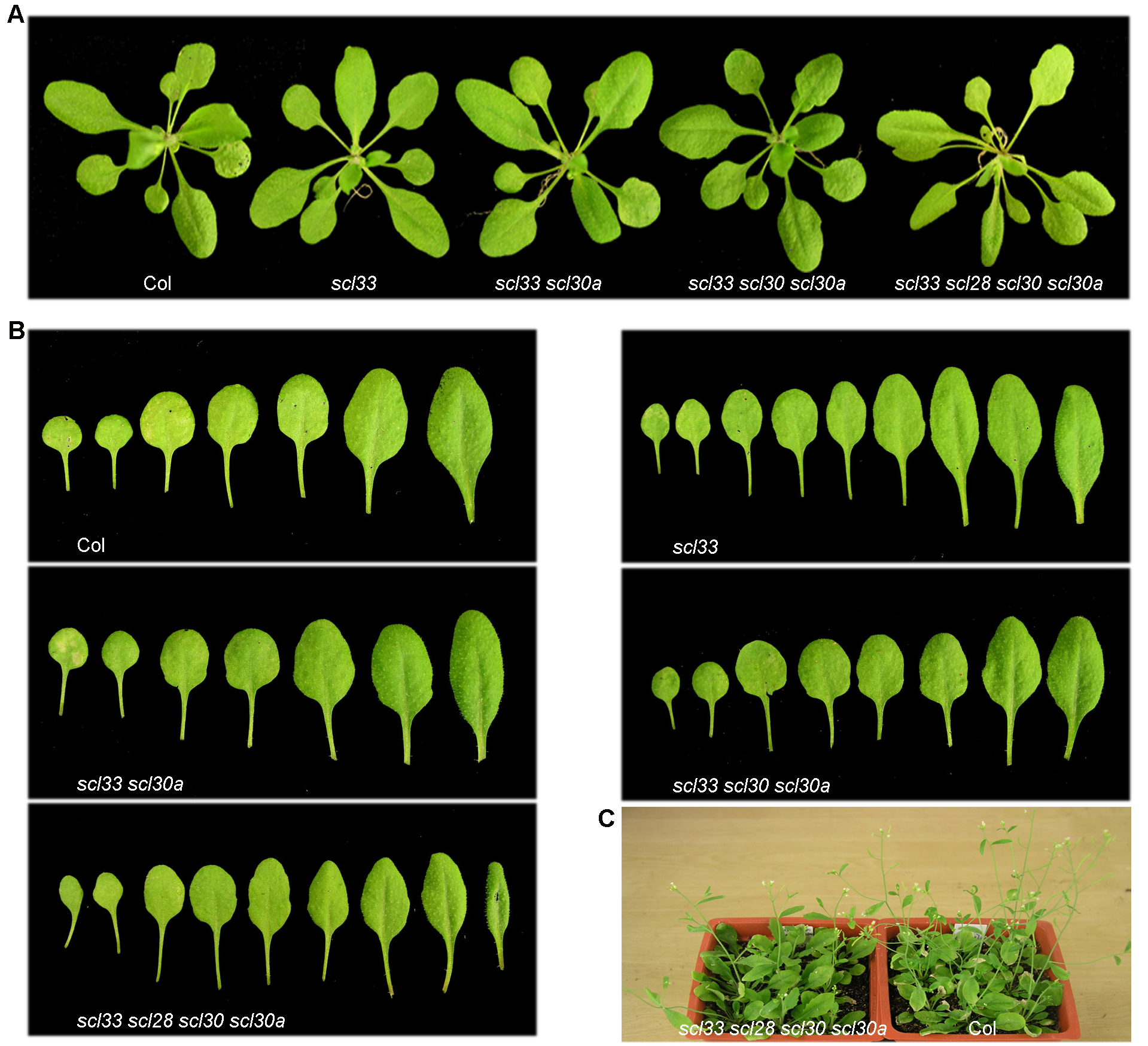

Supplement: S4 Fig — (A) The phenotypes of single, double, triple and quadruple mutants of SC35 and SCL proteins compared with that of WT. No obvious visible phenotypes were observed for the single, double and triple mutants. Mildly serrated rosette leaves were observed for the scl28 scl30 scl30a scl33 quadruple mutant. (B) The rosette leaves of WT and mutants. (C) Plants of WT and scl33 scl28 scl30 scl30a quadruple mutant grown for 35 d under a long day condition. The flowering time of the mutant was slightly delayed compared with that of WT. (TIF) [file pgen.1006663.s004.tif]

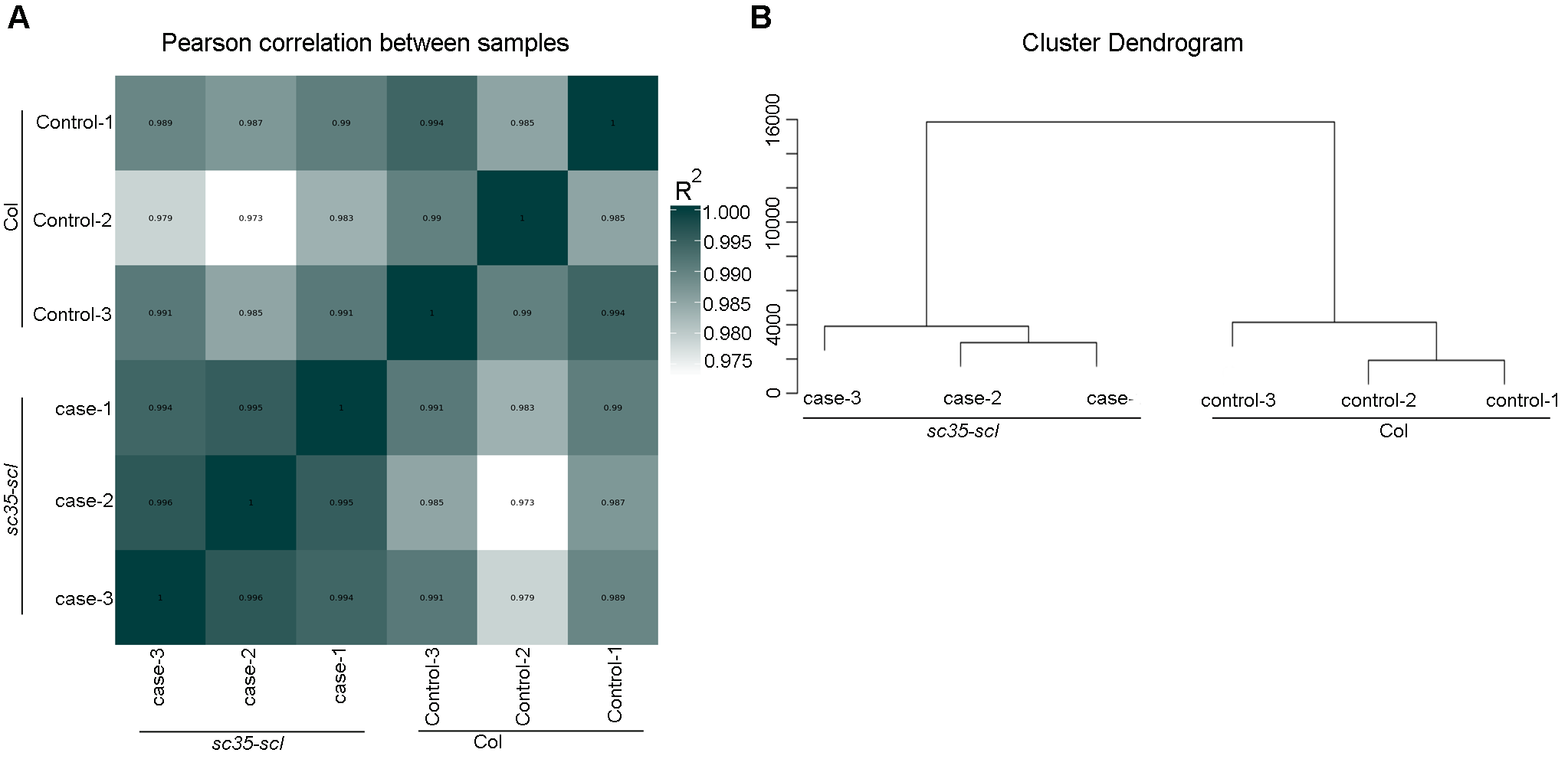

Supplement: S5 Fig — (A) Heatmap of Pearson Correlation between WT and sc35-scl mutant samples. (B) Hierarchical clustering between samples of WT and sc35-scl mutant. (TIF) [file pgen.1006663.s005.tif]

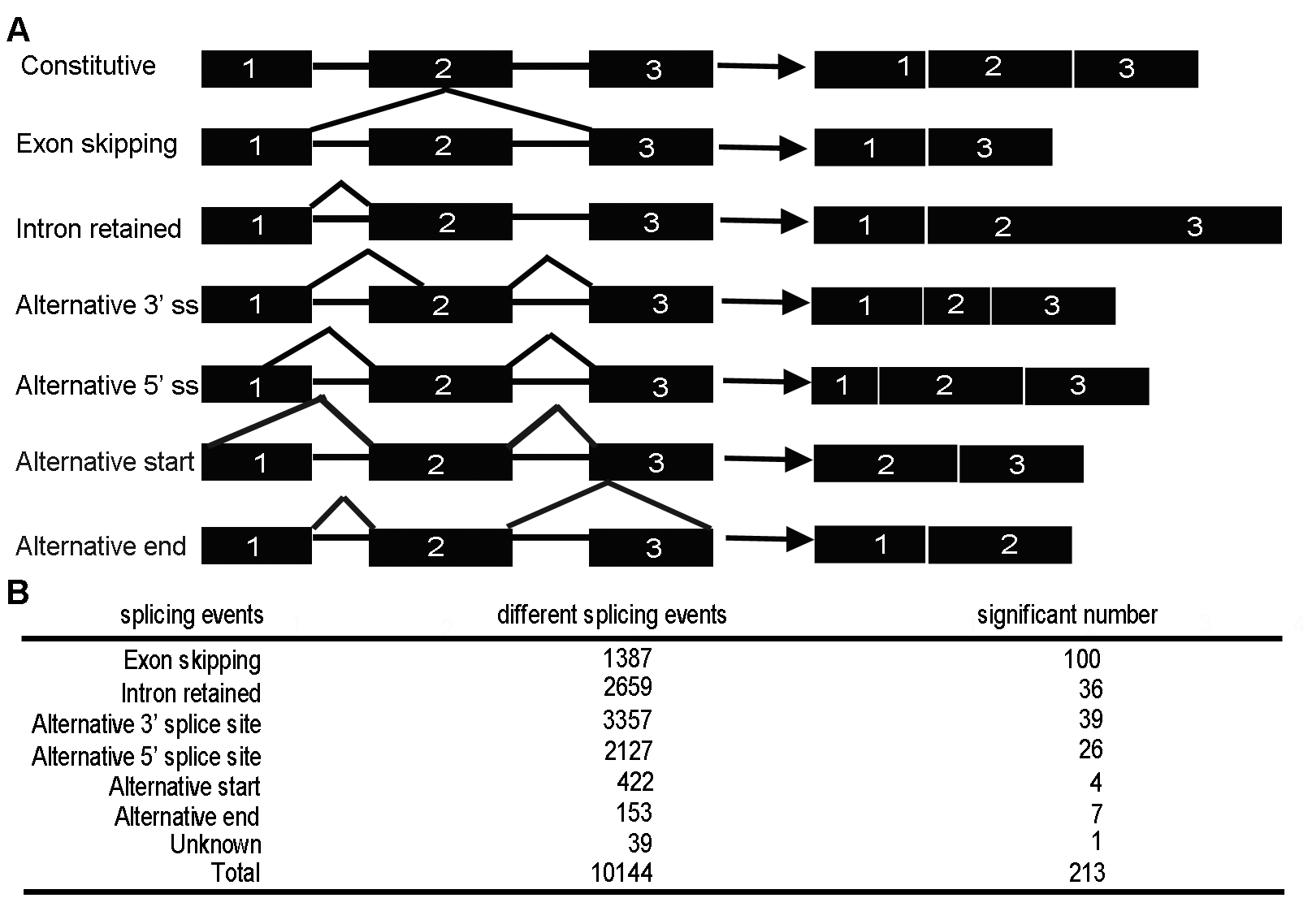

Supplement: S6 Fig — (A) Schematic diagrams showing the alternative splicing patterns. Black boxes represent the exons. (B) The splicing events affected by SC35 and SC35-like proteins. A total of 213 genes (p-value<0.05) with changed splicing patterns were observed from RNA-sequencing data. (TIF) [file pgen.1006663.s006.tif]

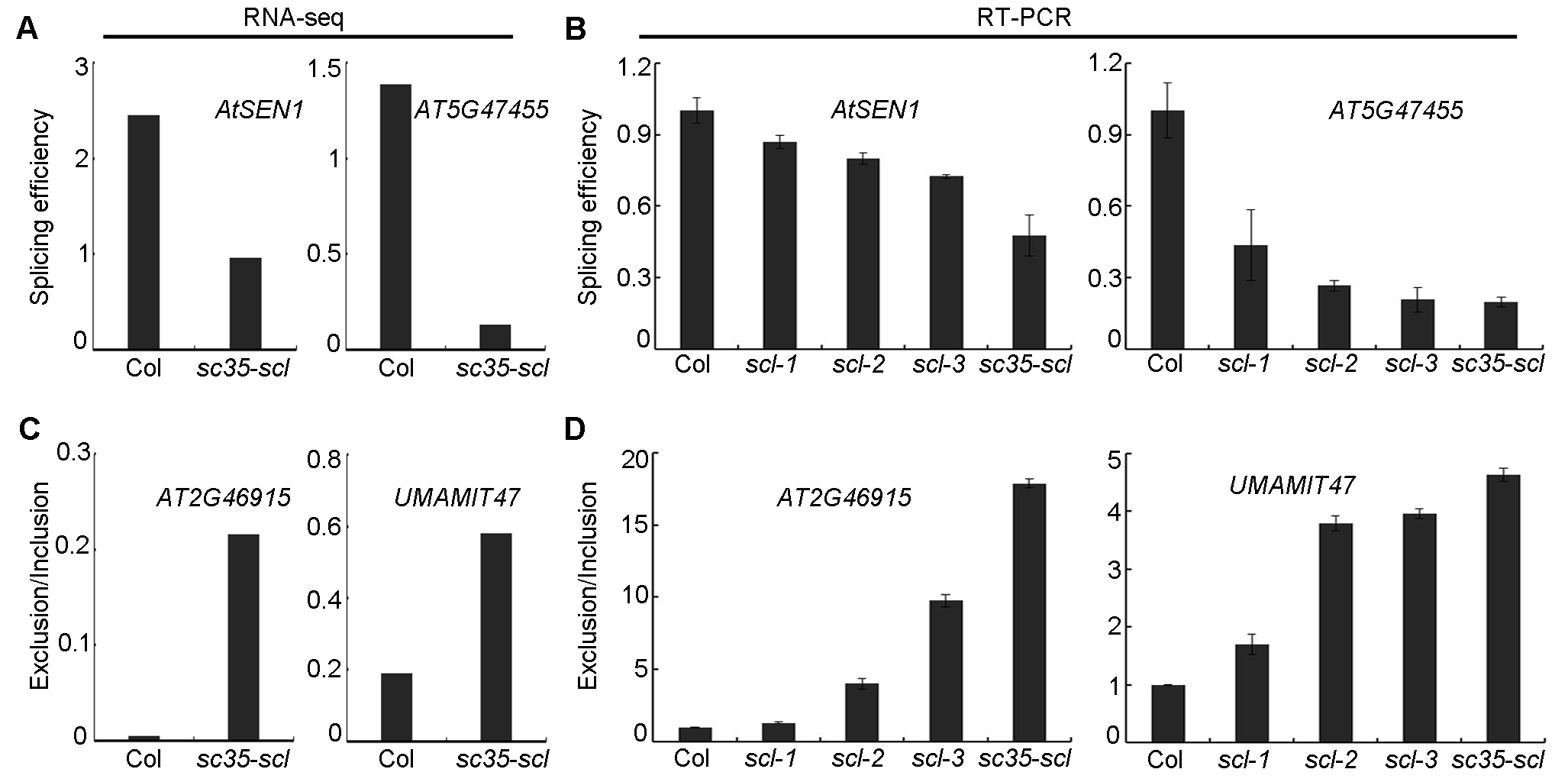

Supplement: S7 Fig — (A) Statistical data of the intron retained splicing of AtSEN1 and AT5G47455affected by SC35 and SCL proteins. Data are from RNA-sequencing. (B) Statistical data of the intron retained splicing of AtSEN1 and AT5G47455 affected by SC35 and SCL proteins.Scl-1: scl33 scl30a double mutant; scl-2: scl33 scl30a scl30 triple mutant; scl-3: scl33 scl30a scl30 scl28 quadruple mutant. Data are from RT-PCR, Values are shown as mean± SEM from three biological repeats. (C) Statistical data of the exon skipping splicing of UMAMIT47 and AT2G46915 affected by SC35 and SCL proteins. Data are from RNA-sequencing. (D) Statistical data of the exon skipping splicing of UMAMIT47 and AT2G46915 affected by SC35 and SCL proteins.Scl-1: scl33 scl30a double mutant; scl-2: scl33 scl30a scl30 triple mutant; scl-3: scl33 scl30a scl30 scl28 quadruple mutant. Data are from RT-PCR, Values are shown as mean± SEM from three biological repeats. (TIF) [file pgen.1006663.s007.tif]

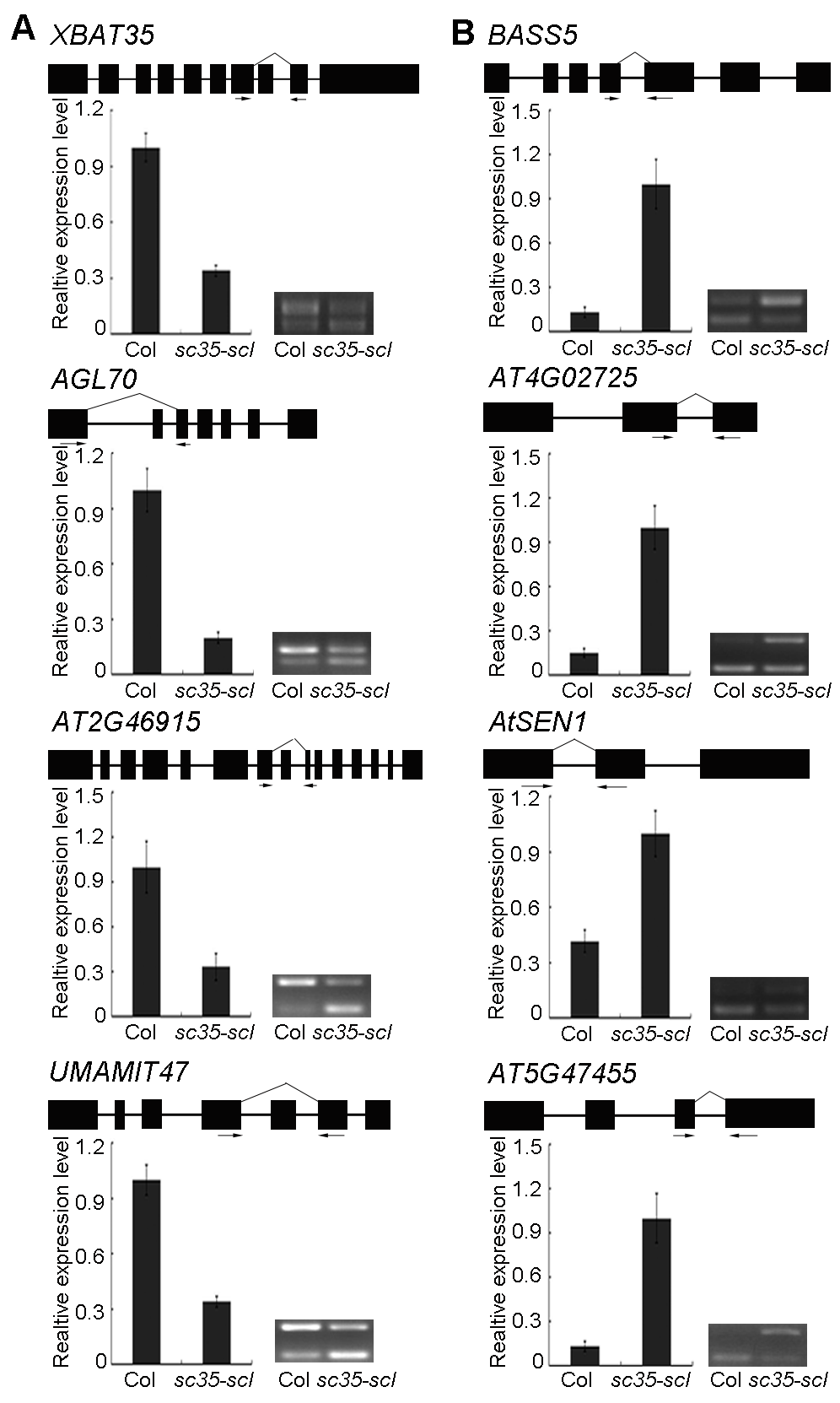

Supplement: S8 Fig — (A) SC35/SCL proteins repress the exon skipping event. (B) SC35/SCL proteins repress the intron retained event. The diagram shows the gene structures of individual gene, black boxes represent exons. Arrows represent RT-PCR primers used. Quantification of the PCR products was measured using the software GIS (Gel Image System), as shown in the histogram. Values are shown as mean± SEM from three biological repeats. (TIF) [file pgen.1006663.s008.tif]

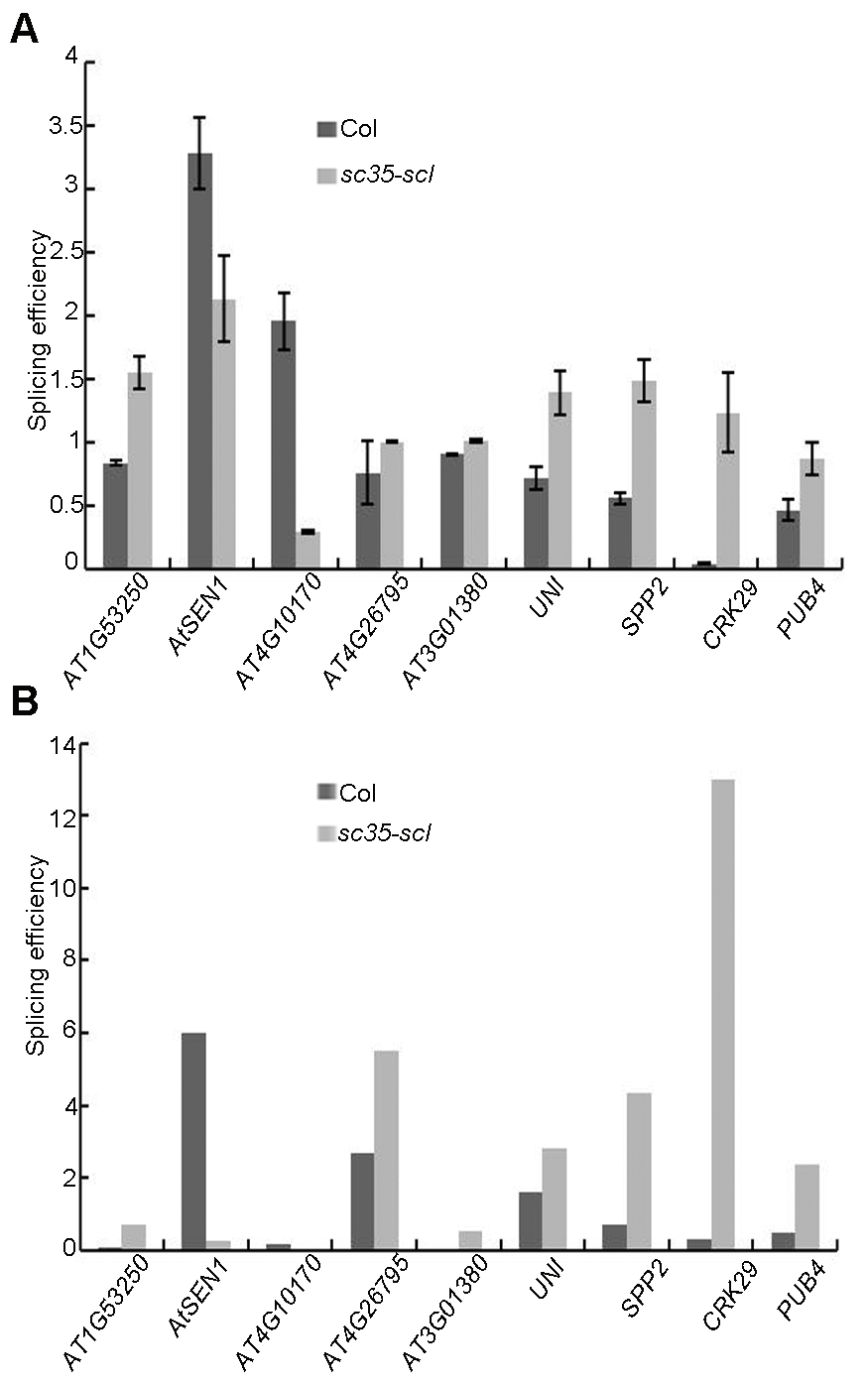

Supplement: S9 Fig — (A) The splicing efficiencies of genes with the AGAAGA motif in WT and sc35-scl mutant as examined by RT-PCR. (B) The splicing efficiencies of genes with the AGAAGA motif in WT and sc35-scl mutant as examined by RNA-seq. Values were shown as the mean± SEM from three biological repeats. (TIF) [file pgen.1006663.s009.tif]

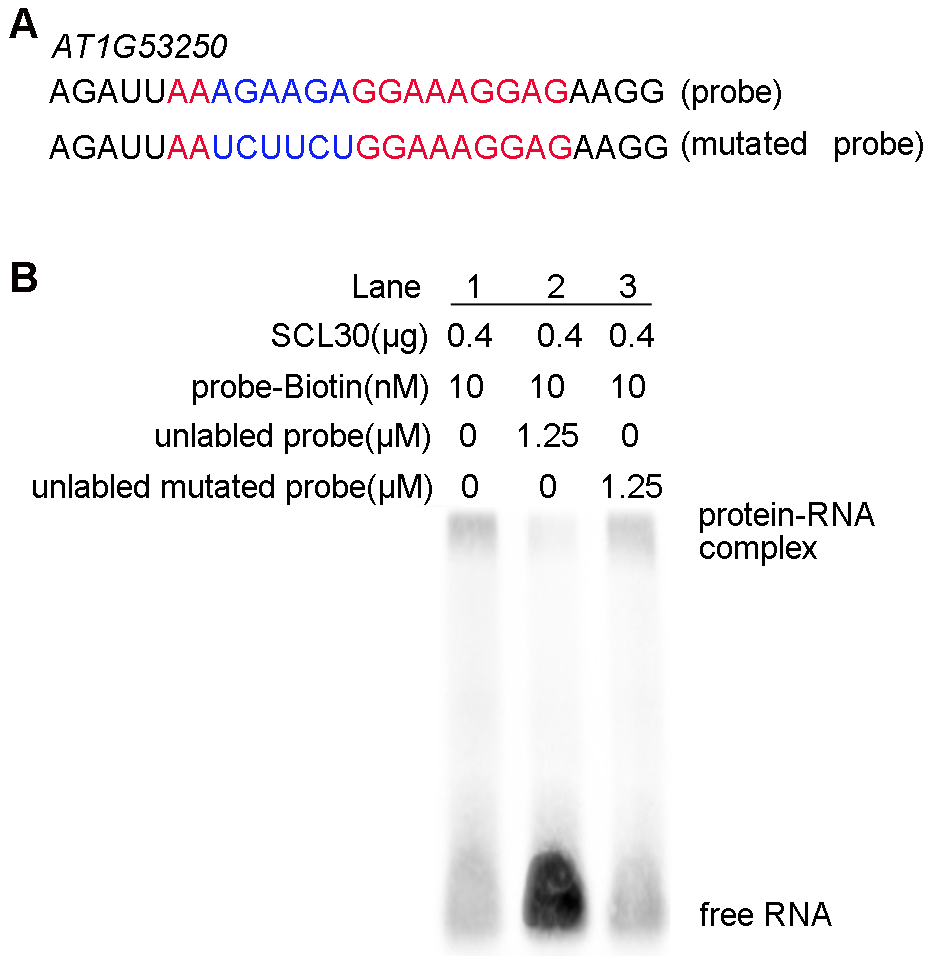

Supplement: S10 Fig — (A) The mutated RNA probe (AGAAGA to UCUUCU). (B) The binding of SCL30 to the specific RNA sequence cannot be competed by the mutated probe in RNA EMSA assay. (TIF) [file pgen.1006663.s010.tif]

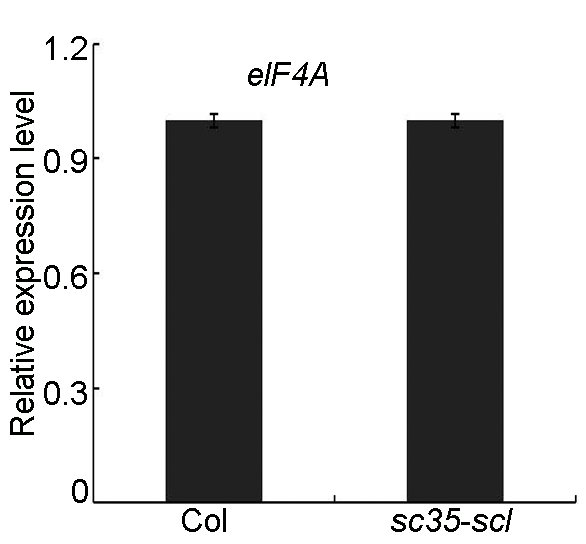

Supplement: S11 Fig — (TIF) [file pgen.1006663.s011.tif]

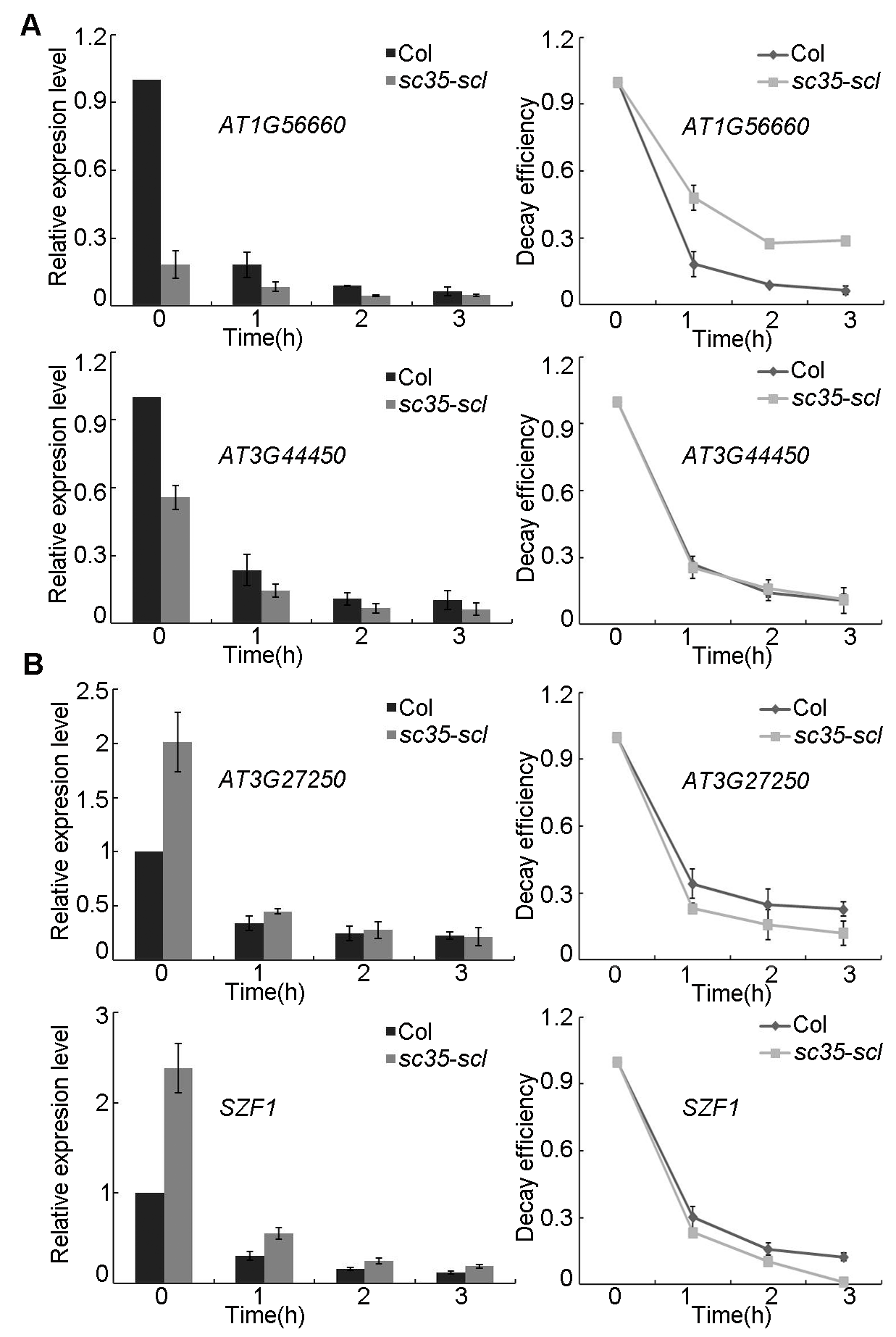

Supplement: S12 Fig — (A) The expression level of AT1G56660 and AT3G44450 upon cordycepin treatment for 0, 1, 2, 3 hours in WT and sc35-scl (left). The decay efficiencies of AT1G56660 and AT3G44450 were shown by normalization of the expression level to that at 0 h (right). (B) The expression level of AT3G27250 and SZF1 upon cordycepin treatment for 0, 1, 2, 3 hours in WT and sc35-scl (left). The decay efficiencies of AT3G27250 and SZF1 were shown by normalization of the expression level to that at 0 h. The level of eIF-4A transcript was used as a control. Values were shown as mean± SEM from three biological repeats. (TIF) [file pgen.1006663.s012.tif]

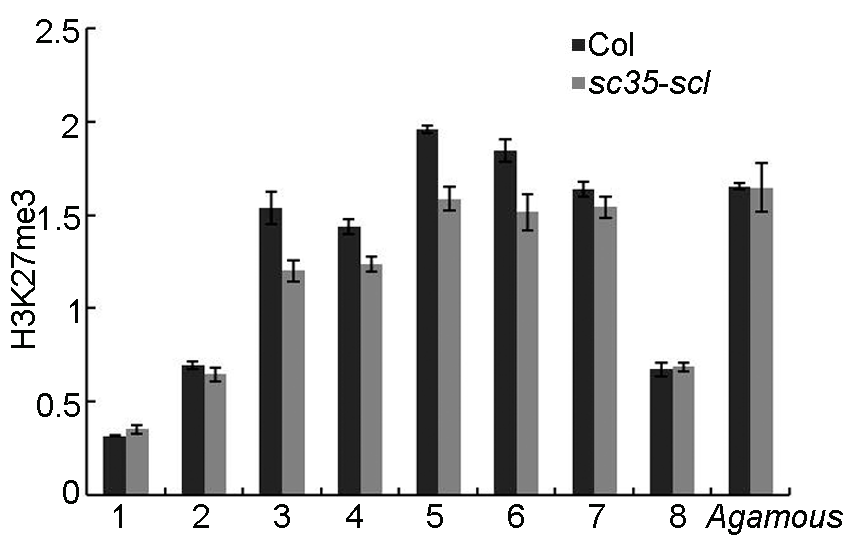

Supplement: S13 Fig — ChIP-PCR assay was used to analyze H3K27me3 enrichments at different positions of FLC, and shown as ratio of (H3K27me3 FLC/input FLC) to (H3 FLC/input FLC). Agarmous was used as an internal control for the ChIP experiments. Values were shown mean± SEM from three technical repeats. ChIP assays were repeated three times with similar results. (TIF) [file pgen.1006663.s013.tif]

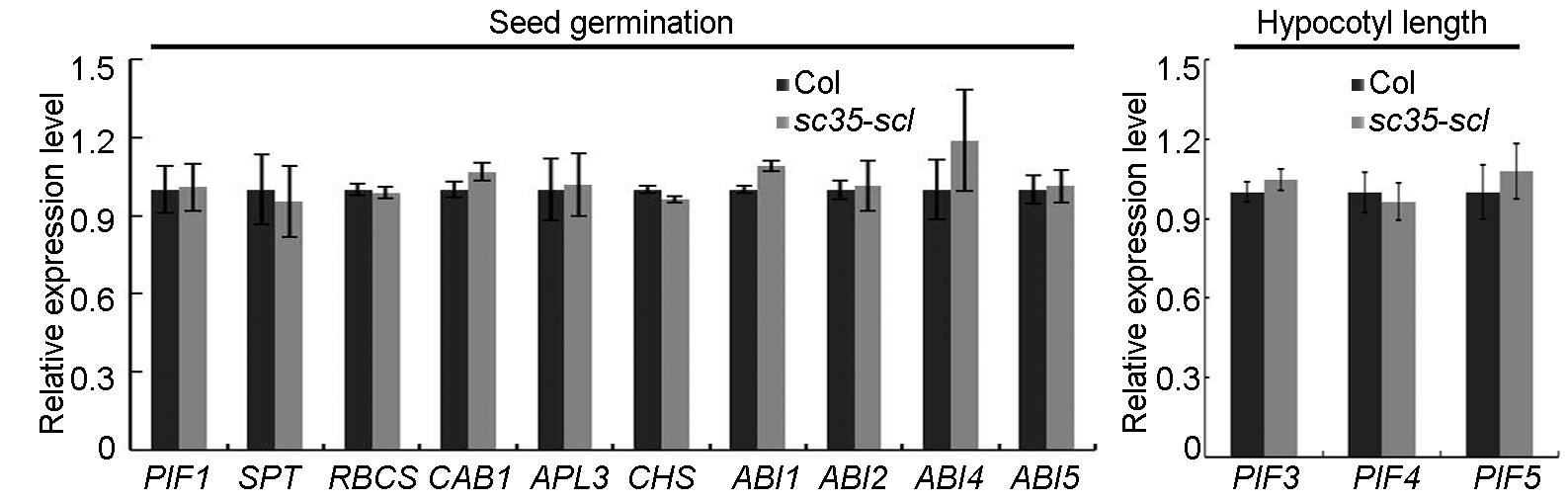

Supplement: S14 Fig — The transcription levels of key genes related to seed germination and hypocotyl elongation as revealed by RT-PCR. Values were shown mean± SEM from three biological repeats. (TIF) [file pgen.1006663.s014.tif]

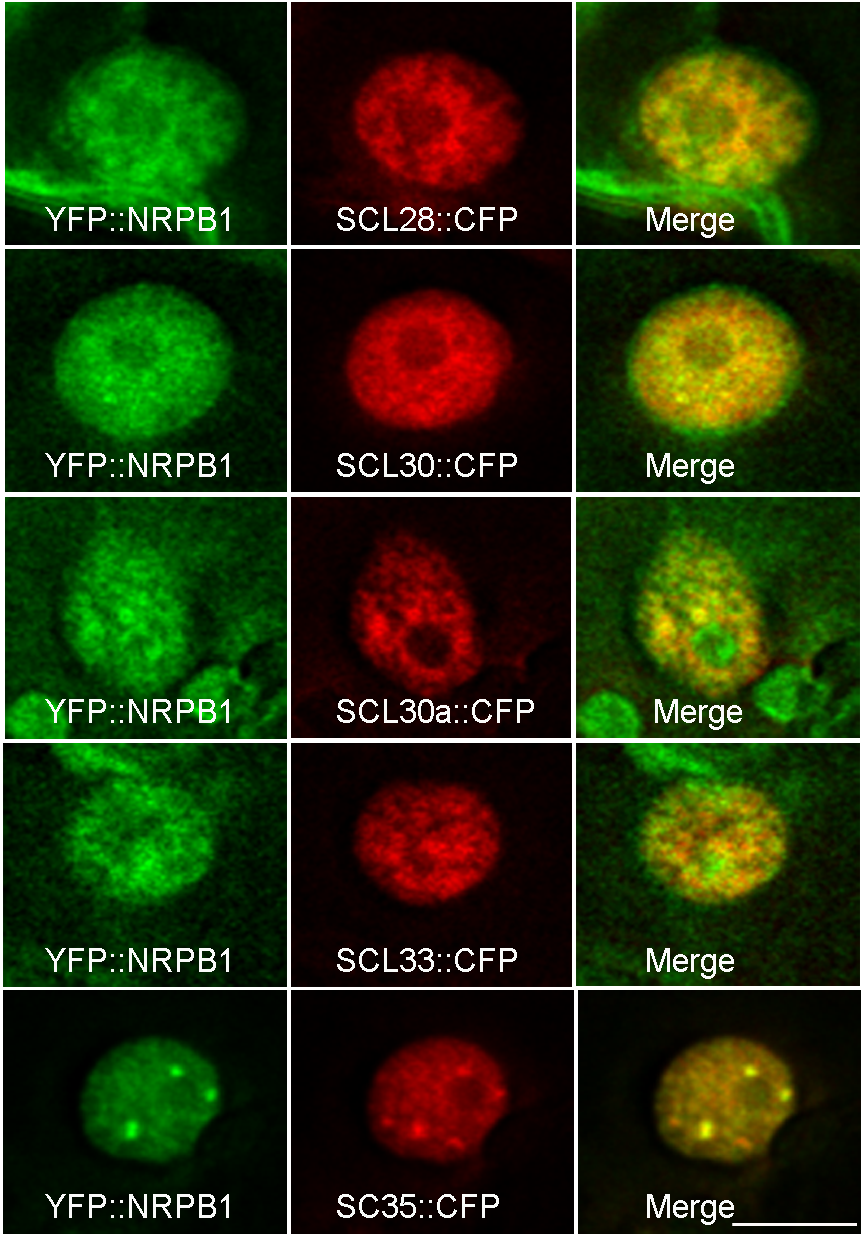

Supplement: S15 Fig — Bar = 10μm. (TIF) [file pgen.1006663.s015.tif]

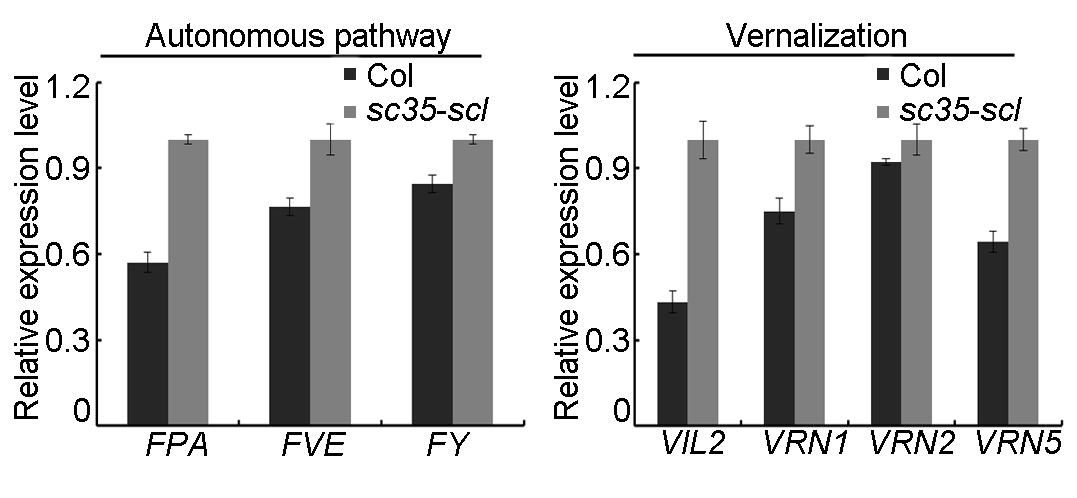

Supplement: S16 Fig — Values were shown mean± SEM from three biological repeats. (TIF) [file pgen.1006663.s016.tif]
