# Supplementary material for: Depletion of Arabidopsis SC35 and SC35-like serine/arginine-rich proteins affects the transcription and splicing of a subset of genes
Source: PLoS Genet. 2017 Mar 8;13(3):e1006663. doi: 10.1371/journal.pgen.1006663 (PMC5362245; doi:10.1371/journal.pgen.1006663)
Supplement: S1 Table — (DOCX) [file pgen.1006663.s017.docx]

**Table 1. Summary of RNA-seq data**

| ColA | | sc35-sclA | |
| --- | --- | --- | --- |
| Statistics | Result | Statistics | Result |
| All | 43344730 | All | 43090624 |
| UnMapped | 339986 | UnMapped | 477352 |
| Mapped | 43004744 | Mapped | 42613272 |
| MappedRate | 0.992 | MappedRate | 0.989 |
| UniqueMapped | 41032316 | UniqueMapped | 40719893 |
| UniqueMappedRate | 0.947 | UniqueMappedRate | 0.945 |
| RepeatMapped | 1972428 | RepeatMapped | 1893379 |
| JunctionAllMapped | 12702725 | JunctionAllMapped | 12658193 |
| JunctionUniqueMapped | 11849849 | JunctionUniqueMapped | 11805931 |
| AllBase | 4330492602 | AllBase | 4306446458 |
| UnMappedBase | 33844590 | UnMappedBase | 47582940 |
| MappedBase | 4296648012 | MappedBase | 4258863518 |
| UniqueMappedBase | 4099582435 | UniqueMappedBase | 4069635062 |
| RepeatMappedBase | 197065577 | RepeatMappedBase | 189228456 |
|  |  |  |  |
| ColB | | sc35-sclB | |
| Statistics | Result | Statistics | Result |
| All | 43793538 | All | 42761810 |
| UnMapped | 340748 | UnMapped | 441380 |
| Mapped | 43452790 | Mapped | 42320430 |
| MappedRate | 0.992 | MappedRate | 0.99 |
| UniqueMapped | 41024489 | UniqueMapped | 40408418 |
| UniqueMappedRate | 0.937 | UniqueMappedRate | 0.945 |
| RepeatMapped | 2428301 | RepeatMapped | 1912012 |
| JunctionAllMapped | 12796567 | JunctionAllMapped | 12639412 |
| JunctionUniqueMapped | 11950795 | JunctionUniqueMapped | 11779527 |
| AllBase | 4375078784 | AllBase | 4273551297 |
| UnMappedBase | 33930669 | UnMappedBase | 43999627 |
| MappedBase | 4341148115 | MappedBase | 4229551670 |
| UniqueMappedBase | 4098542007 | UniqueMappedBase | 4038468142 |
| RepeatMappedBase | 242606108 | RepeatMappedBase | 191083528 |
|  |  |  |  |
| ColC | | sc35-sclC | |
| Statistics | Result | Statistics | Result |
| All | 43482104 | All | 43722652 |
| UnMapped | 365629 | UnMapped | 462372 |
| Mapped | 43116475 | Mapped | 43260280 |
| MappedRate | 0.992 | MappedRate | 0.989 |
| UniqueMapped | 41115298 | UniqueMapped | 41260420 |
| UniqueMappedRate | 0.946 | UniqueMappedRate | 0.944 |
| RepeatMapped | 2001177 | RepeatMapped | 1999860 |
| JunctionAllMapped | 12750336 | JunctionAllMapped | 12709255 |
| JunctionUniqueMapped | 11882757 | JunctionUniqueMapped | 11863840 |
| AllBase | 4344469013 | AllBase | 4369704375 |
| UnMappedBase | 36403242 | UnMappedBase | 46086632 |
| MappedBase | 4308065771 | MappedBase | 4323617743 |
| UniqueMappedBase | 4108114703 | UniqueMappedBase | 4123750723 |
| RepeatMappedBase | 199951068 | RepeatMappedBase | 199867020 |

epeatMappedBase 199867020
