# Supplementary material for: Depletion of Arabidopsis SC35 and SC35-like serine/arginine-rich proteins affects the transcription and splicing of a subset of genes
Source: PLoS Genet. 2017 Mar 8;13(3):e1006663. doi: 10.1371/journal.pgen.1006663 (PMC5362245; doi:10.1371/journal.pgen.1006663)
Supplement: S2 Table — The differentially expressed genes between WT and sc35-scl quintuple mutant (>1.5 fold, p<0.05). Data were analyzed using the EB-seq algorithm. (DOCX) [file pgen.1006663.s018.docx]

**Table S2 The genes differentially expressed between WT and *sc35-scl* mutant in RNA-seq data**

| AccID | Log2FC | FDR | Style | SC35-SCL | Col |
| --- | --- | --- | --- | --- | --- |
| AT2G11410 | 20 | 0 | up | 513 | 0 |
| AT1G53480 | 5.288759679 | 0 | up | 386 | 10 |
| AT2G42540 | 3.222997488 | 0 | up | 461 | 50 |
| AT1G53490 | 3.14407467 | 0 | up | 419 | 48 |
| AT5G52300 | 3.056098923 | 0 | up | 386 | 47 |
| AT4G08093 | 2.920534063 | 0 | up | 157 | 21 |
| AT3G27860 | 2.806726632 | 0 | up | 228 | 33 |
| AT2G37870 | 2.63714057 | 0 | up | 344 | 56 |
| AT1G60190 | 2.599431319 | 0 | up | 383 | 64 |
| AT3G14440 | 2.303080728 | 0 | up | 653 | 134 |
| AT5G35777 | 2.295070942 | 0 | up | 252 | 52 |
| AT2G16367 | 2.14265619 | 0 | up | 1186 | 272 |
| AT3G48360 | 2.134975701 | 0 | up | 759 | 175 |
| AT2G40000 | 2.127101254 | 0 | up | 4430 | 1027 |
| AT3G27250 | 2.121978662 | 0 | up | 490 | 114 |
| AT5G59320 | 2.083274269 | 0 | up | 431 | 103 |
| AT5G56550 | 2.039625519 | 0 | up | 1019 | 251 |
| AT3G55980 | 2.010289939 | 0 | up | 1993 | 501 |
| AT4G33550 | 1.991375245 | 0 | up | 958 | 244 |
| AT2G39800 | 1.945003385 | 0 | up | 25755 | 6774 |
| AT4G11280 | 1.929843743 | 0 | up | 1377 | 366 |
| AT1G80440 | 1.925219773 | 0 | up | 3664 | 977 |
| AT1G23390 | 1.89836732 | 0 | up | 4421 | 1201 |
| AT2G20670 | 1.894140389 | 0 | up | 5344 | 1456 |
| AT1G02205 | 1.87931314 | 0 | up | 2503 | 689 |
| AT2G44130 | 1.862169427 | 0 | up | 639 | 178 |
| AT2G41190 | 1.706462508 | 0 | up | 912 | 283 |
| AT3G46600 | 1.673363411 | 0 | up | 2633 | 836 |
| AT3G15450 | 1.616935551 | 0 | up | 3059 | 1010 |
| AT5G35190 | 1.575108491 | 0 | up | 1577 | 536 |
| AT5G21940 | 1.555121845 | 0 | up | 3099 | 1068 |
| AT4G12480 | -1.63037005 | 0 | down | 340 | 1066 |
| AT2G16060 | -1.668872825 | 0 | down | 418 | 1346 |
| AT3G05730 | -1.700174285 | 0 | down | 884 | 2909 |
| AT1G65190 | -1.758951957 | 0 | down | 283 | 970 |
| AT4G02970 | -1.793267879 | 0 | down | 298 | 1046 |
| AT5G64200 | -2.226735376 | 0 | down | 231 | 1095 |
| AT2G21640 | -2.473622359 | 0 | down | 40 | 225 |
| AT3G55460 | -2.500180571 | 0 | down | 244 | 1398 |
| AT3G16670 | -2.529765437 | 0 | down | 336 | 1965 |
| AT3G13570 | -2.806935461 | 0 | down | 184 | 1304 |
| AT2G01422 | -4.894658599 | 0 | down | 8 | 241 |
| AT5G09570 | -2.028243693 | 1.11E-16 | down | 84 | 347 |
| AT5G51190 | 2.109378625 | 2.22E-16 | up | 294 | 69 |
| AT2G38470 | 1.565629825 | 4.44E-16 | up | 1251 | 428 |
| AT5G15500 | 2.326675603 | 7.77E-16 | up | 213 | 43 |
| AT2G11405 | 20 | 9.99E-16 | up | 60 | 0 |
| AT5G13170 | 1.685790324 | 1.22E-15 | up | 629 | 198 |
| AT2G25900 | 1.453831062 | 1.22E-15 | up | 3062 | 1132 |
| AT3G06435 | -2.010338415 | 1.78E-15 | down | 75 | 306 |
| AT4G12490 | -2.310551029 | 1.78E-15 | down | 42 | 211 |
| AT1G56660 | -1.688163158 | 2.22E-15 | down | 186 | 607 |
| AT5G02020 | 1.514383971 | 2.44E-15 | up | 1323 | 469 |
| AT3G50480 | -1.824227986 | 2.89E-15 | down | 116 | 416 |
| AT1G72910 | 1.624051843 | 1.31E-14 | up | 627 | 206 |
| AT1G55310 | -1.519242838 | 1.41E-14 | down | 350 | 1016 |
| AT3G19620 | 1.724169436 | 1.48E-14 | up | 460 | 141 |
| AT3G18773 | 1.785958585 | 1.62E-14 | up | 395 | 116 |
| AT5G24850 | -1.612901945 | 2.24E-14 | down | 205 | 635 |
| AT4G39675 | -1.659841168 | 2.26E-14 | down | 170 | 544 |
| AT5G37260 | 1.482177837 | 7.97E-14 | up | 960 | 348 |
| AT5G63160 | 1.40849255 | 1.06E-13 | up | 1578 | 602 |
| AT3G28220 | 1.360765522 | 1.20E-13 | up | 2891 | 1140 |
| AT2G18700 | 1.394274105 | 1.27E-13 | up | 1752 | 675 |
| AT5G52310 | 1.399013129 | 1.86E-13 | up | 1526 | 586 |
| AT1G18710 | 1.528874917 | 2.05E-13 | up | 681 | 239 |
| AT2G11880 | -2.711121673 | 2.08E-13 | down | 19 | 126 |
| AT1G67105 | -1.706762215 | 2.23E-13 | down | 121 | 400 |
| AT3G10020 | 1.455835993 | 3.58E-13 | up | 902 | 333 |
| AT5G49080 | 1.439168838 | 3.96E-13 | up | 980 | 366 |
| AT1G60590 | 1.331422885 | 6.31E-13 | up | 2552 | 1027 |
| AT3G07350 | 1.378160935 | 6.69E-13 | up | 1404 | 547 |
| AT2G30770 | 2.447074036 | 7.49E-13 | up | 140 | 26 |
| AT5G47450 | 1.574792812 | 8.04E-13 | up | 503 | 171 |
| AT1G72920 | 1.867325167 | 8.30E-13 | up | 263 | 73 |
| AT3G26510 | 1.296632281 | 1.06E-12 | up | 4587 | 1891 |
| AT4G33720 | -3.682208981 | 1.09E-12 | down | 6 | 78 |
| AT5G59220 | 1.606438897 | 1.11E-12 | up | 445 | 148 |
| AT4G02280 | 1.433268236 | 1.43E-12 | up | 840 | 315 |
| AT5G24150 | 1.285480129 | 2.39E-12 | up | 3702 | 1538 |
| AT5G17890 | -1.353184282 | 2.43E-12 | down | 533 | 1379 |
| AT3G16530 | -1.341067117 | 3.26E-12 | down | 564 | 1447 |
| AT2G01008 | 1.882416882 | 4.01E-12 | up | 233 | 64 |
| AT2G43140 | -3.135574599 | 4.56E-12 | down | 10 | 89 |
| AT1G80840 | 1.862579867 | 4.93E-12 | up | 237 | 66 |
| AT1G27020 | -1.625625453 | 6.00E-12 | down | 120 | 375 |
| AT1G69480 | 1.706815869 | 7.18E-12 | up | 303 | 94 |
| AT3G26740 | 1.285557439 | 8.17E-12 | up | 2087 | 867 |
| AT4G27280 | 1.369970883 | 8.45E-12 | up | 929 | 364 |
| AT5G60250 | 1.913261177 | 9.08E-12 | up | 212 | 57 |
| AT2G40330 | -3.303697358 | 1.16E-11 | down | 8 | 80 |
| AT3G01420 | 1.250268097 | 1.17E-11 | up | 3702 | 1576 |
| AT5G57560 | 1.402340408 | 1.34E-11 | up | 723 | 277 |
| AT1G69490 | 1.302575649 | 1.74E-11 | up | 1364 | 560 |
| AT3G44450 | -2.244803669 | 1.78E-11 | down | 30 | 144 |
| AT2G01860 | -2.161092962 | 2.19E-11 | down | 34 | 154 |
| AT1G52000 | 1.21733619 | 3.09E-11 | up | 6043 | 2632 |
| AT2G15090 | 1.313326352 | 3.11E-11 | up | 1092 | 445 |
| AT1G16090 | -2.292970951 | 3.15E-11 | down | 27 | 134 |
| AT5G09930 | 1.806726632 | 3.37E-11 | up | 228 | 66 |
| AT4G36040 | 1.236558185 | 3.92E-11 | up | 2599 | 1117 |
| AT2G27830 | 1.329901728 | 4.33E-11 | up | 911 | 367 |
| AT5G17300 | 1.222516109 | 4.43E-11 | up | 3431 | 1489 |
| AT3G54580 | 1.195399852 | 6.60E-11 | up | 10981 | 4856 |
| AT1G13260 | 1.301020438 | 7.76E-11 | up | 1000 | 411 |
| AT5G66400 | 1.354312926 | 7.92E-11 | up | 717 | 284 |
| AT4G37610 | 1.240083531 | 8.63E-11 | up | 1782 | 764 |
| AT5G61600 | 1.507333222 | 9.38E-11 | up | 393 | 140 |
| AT1G72360 | -1.550612098 | 9.69E-11 | down | 120 | 356 |
| AT1G69260 | 1.455636049 | 1.06E-10 | up | 455 | 168 |
| AT3G21720 | -1.374369373 | 1.07E-10 | down | 243 | 638 |
| AT1G77120 | -1.194221817 | 1.15E-10 | down | 2039 | 4725 |
| AT4G04760 | 2.574624086 | 1.21E-10 | up | 100 | 17 |
| AT1G26390 | 4.21462795 | 1.34E-10 | up | 55 | 3 |
| AT2G44180 | -1.348886132 | 1.61E-10 | down | 264 | 681 |
| AT1G31290 | 2.210875815 | 1.91E-10 | up | 128 | 28 |
| AT2G42530 | 2.340158832 | 1.91E-10 | up | 115 | 23 |
| AT1G62440 | 1.440852832 | 2.06E-10 | up | 445 | 166 |
| AT2G30766 | 2.103119635 | 2.33E-10 | up | 140 | 33 |
| AT1G54040 | 1.449159609 | 2.41E-10 | up | 426 | 158 |
| AT1G75750 | -1.175402691 | 2.91E-10 | down | 1824 | 4172 |
| AT2G33380 | 1.24642878 | 3.05E-10 | up | 1162 | 496 |
| AT5G05440 | -1.500879216 | 3.11E-10 | down | 127 | 364 |
| AT2G39510 | -1.37475352 | 3.72E-10 | down | 206 | 541 |
| AT2G38310 | -1.209552822 | 3.80E-10 | down | 725 | 1698 |
| AT3G54590 | 1.151817794 | 5.76E-10 | up | 5518 | 2515 |
| AT2G19590 | -1.215230553 | 6.32E-10 | down | 575 | 1352 |
| AT1G49450 | 2.212608782 | 6.62E-10 | up | 119 | 26 |
| AT3G12580 | 1.144992483 | 7.11E-10 | up | 6396 | 2929 |
| AT4G21650 | 1.16194747 | 7.22E-10 | up | 2932 | 1327 |
| AT5G46900 | 1.300169101 | 7.57E-10 | up | 676 | 278 |
| AT1G79520 | 1.214280808 | 8.67E-10 | up | 1212 | 529 |
| AT1G11440 | -1.521073047 | 9.60E-10 | down | 107 | 311 |
| AT1G18870 | 1.425888706 | 1.09E-09 | up | 390 | 147 |
| AT5G15970 | 1.154241798 | 1.27E-09 | up | 2512 | 1143 |
| AT4G12550 | -1.1601406 | 1.38E-09 | down | 984 | 2227 |
| AT1G49860 | -2.229696776 | 1.45E-09 | down | 24 | 114 |
| AT2G17850 | -1.572186194 | 1.60E-09 | down | 88 | 265 |
| AT2G18660 | -2.46416203 | 1.65E-09 | down | 17 | 95 |
| AT5G57760 | -1.498987157 | 1.65E-09 | down | 109 | 312 |
| AT1G15380 | -1.506514241 | 1.73E-09 | down | 106 | 305 |
| AT5G62020 | 1.282620423 | 1.94E-09 | up | 639 | 266 |
| AT3G56880 | 1.21189546 | 1.97E-09 | up | 1011 | 442 |
| AT5G35940 | -1.331986402 | 2.09E-09 | down | 202 | 515 |
| AT1G33960 | -3.174414341 | 3.24E-09 | down | 7 | 64 |
| AT3G53980 | 1.152465313 | 3.73E-09 | up | 1587 | 723 |
| AT3G59940 | 1.117012356 | 3.86E-09 | up | 3279 | 1531 |
| AT4G03230 | 1.231662291 | 4.19E-09 | up | 749 | 323 |
| AT5G01520 | 1.286719573 | 5.85E-09 | up | 530 | 220 |
| AT3G28550 | 1.091406653 | 6.08E-09 | up | 13163 | 6256 |
| AT1G73480 | 1.145250359 | 1.03E-08 | up | 1234 | 565 |
| AT2G33790 | 1.235822172 | 1.29E-08 | up | 600 | 258 |
| AT3G16660 | -1.162533497 | 1.31E-08 | down | 442 | 1002 |
| AT2G32800 | 1.210875815 | 1.31E-08 | up | 688 | 301 |
| AT5G04120 | 1.903588171 | 1.39E-08 | up | 133 | 36 |
| AT5G25280 | 1.074545882 | 1.40E-08 | up | 5590 | 2688 |
| AT3G30775 | -1.104817807 | 1.44E-08 | down | 921 | 2006 |
| AT1G27730 | 1.43841154 | 1.82E-08 | up | 281 | 105 |
| AT1G65970 | -1.17291075 | 2.13E-08 | down | 360 | 822 |
| AT1G70290 | 1.058482905 | 2.91E-08 | up | 4652 | 2262 |
| AT3G62680 | 1.097777536 | 3.51E-08 | up | 1454 | 688 |
| AT1G26800 | 1.320530025 | 3.61E-08 | up | 365 | 148 |
| AT5G59310 | 2.825585659 | 4.27E-08 | up | 63 | 9 |
| AT1G13609 | 2.925121333 | 4.50E-08 | up | 60 | 8 |
| AT3G09940 | -1.282686865 | 4.87E-08 | down | 166 | 409 |
| AT5G46890 | 1.16582014 | 5.48E-08 | up | 689 | 311 |
| AT3G62070 | 1.533819535 | 6.20E-08 | up | 203 | 71 |
| AT3G24460 | 1.149936416 | 6.26E-08 | up | 745 | 340 |
| AT3G15310 | -1.525509101 | 7.23E-08 | down | 71 | 207 |
| AT1G13430 | -1.559314292 | 7.38E-08 | down | 65 | 194 |
| AT1G53830 | 1.170497072 | 8.89E-08 | up | 609 | 274 |
| AT2G41730 | -2.20957518 | 9.08E-08 | down | 19 | 89 |
| AT1G68570 | 1.054042804 | 9.33E-08 | up | 1917 | 935 |
| AT5G64190 | 1.429299334 | 9.69E-08 | up | 242 | 91 |
| AT1G36180 | 1.119079743 | 1.33E-07 | up | 785 | 366 |
| AT4G21870 | -1.272586825 | 1.42E-07 | down | 150 | 367 |
| AT1G23720 | 1.012649008 | 1.46E-07 | up | 7997 | 4014 |
| AT5G05640 | -1.715375845 | 1.48E-07 | down | 43 | 143 |
| AT4G19060 | -1.291097321 | 1.51E-07 | down | 138 | 342 |
| AT3G56360 | 1.051174836 | 1.67E-07 | up | 1506 | 736 |
| AT5G02590 | -1.325356239 | 1.76E-07 | down | 119 | 302 |
| AT5G66270 | -1.767264751 | 1.88E-07 | down | 38 | 131 |
| AT5G42060 | -1.361357926 | 2.09E-07 | down | 103 | 268 |
| AT3G44900 | 2.139246138 | 2.21E-07 | up | 87 | 20 |
| AT3G49970 | -1.882233589 | 2.46E-07 | down | 30 | 112 |
| AT4G07510 | 4.227684103 | 2.49E-07 | up | 37 | 2 |
| AT1G19530 | -1.171146627 | 2.78E-07 | down | 221 | 504 |
| AT2G41090 | -0.997735568 | 3.00E-07 | down | 2561 | 5179 |
| AT5G55450 | -1.542069709 | 3.34E-07 | down | 59 | 174 |
| AT1G75335 | -1.715123604 | 3.54E-07 | down | 40 | 133 |
| AT1G12040 | 1.056591211 | 3.76E-07 | up | 1029 | 501 |
| AT4G29990 | -1.749595821 | 3.95E-07 | down | 37 | 126 |
| AT5G66985 | -1.189526338 | 4.65E-07 | down | 184 | 425 |
| AT2G18328 | -1.237424138 | 4.82E-07 | down | 147 | 351 |
| AT4G01950 | 1.024462691 | 4.94E-07 | up | 1392 | 693 |
| AT5G14920 | -1.035585098 | 4.95E-07 | down | 592 | 1229 |
| AT2G34390 | -1.39035174 | 5.73E-07 | down | 84 | 223 |
| AT3G04720 | -0.980003058 | 5.90E-07 | down | 2452 | 4898 |
| AT5G19120 | 0.978007168 | 6.17E-07 | up | 4598 | 2364 |
| AT1G49570 | -2.048883459 | 6.70E-07 | down | 21 | 88 |
| AT4G19100 | -1.175410333 | 7.93E-07 | down | 181 | 414 |
| AT4G31870 | 1.443536572 | 8.26E-07 | up | 188 | 70 |
| AT4G10270 | -1.215259393 | 8.87E-07 | down | 148 | 348 |
| AT4G15910 | -0.986454428 | 9.00E-07 | down | 1076 | 2159 |
| AT4G19680 | 1.846750269 | 9.16E-07 | up | 103 | 29 |
| AT5G43840 | 2.133707954 | 1.01E-06 | up | 78 | 18 |
| AT4G08410 | 1.062749404 | 1.04E-06 | up | 724 | 351 |
| AT5G23750 | 1.090318352 | 1.06E-06 | up | 595 | 283 |
| AT1G69760 | -1.099338859 | 1.06E-06 | down | 265 | 575 |
| AT2G39681 | -1.190011196 | 1.07E-06 | down | 161 | 372 |
| AT3G29970 | -3.374086686 | 1.13E-06 | down | 4 | 42 |
| AT3G10340 | 1.006178077 | 1.29E-06 | up | 1192 | 601 |
| AT1G54970 | 1.094466323 | 1.43E-06 | up | 544 | 258 |
| AT1G21320 | -2.282938798 | 1.44E-06 | down | 14 | 69 |
| AT4G20820 | 1.136493619 | 1.52E-06 | up | 432 | 199 |
| AT3G03270 | -0.975469275 | 1.65E-06 | down | 918 | 1828 |
| AT1G36370 | 0.958827532 | 1.65E-06 | up | 2831 | 1475 |
| AT5G52780 | -1.030560702 | 1.75E-06 | down | 407 | 842 |
| AT1G02820 | 1.069026589 | 1.79E-06 | up | 607 | 293 |
| AT5G42530 | -0.943925912 | 2.10E-06 | down | 2781 | 5418 |
| AT5G55930 | -1.188549485 | 2.10E-06 | down | 146 | 337 |
| AT2G47770 | 2.317791019 | 2.26E-06 | up | 64 | 13 |
| AT4G11460 | -1.611819653 | 2.26E-06 | down | 42 | 130 |
| AT1G49210 | 4.876211732 | 2.30E-06 | up | 29 | 1 |
| AT3G26200 | -1.714485384 | 2.30E-06 | down | 34 | 113 |
| AT5G64120 | -0.994662066 | 2.30E-06 | down | 557 | 1124 |
| AT2G25200 | 1.242398783 | 2.42E-06 | up | 271 | 116 |
| AT2G42610 | -1.169169231 | 2.49E-06 | down | 155 | 353 |
| AT1G02660 | 1.210182045 | 2.61E-06 | up | 297 | 130 |
| AT4G05070 | 0.985865211 | 2.70E-06 | up | 1146 | 586 |
| AT2G22470 | 0.980262887 | 2.90E-06 | up | 1200 | 616 |
| AT1G32920 | 1.037829915 | 2.97E-06 | up | 667 | 329 |
| AT3G09220 | 0.999694786 | 3.05E-06 | up | 928 | 470 |
| AT2G23000 | 0.975980217 | 3.06E-06 | up | 1245 | 641 |
| AT1G06100 | 1.750534954 | 3.26E-06 | up | 103 | 31 |
| AT5G28300 | -0.974760204 | 3.47E-06 | down | 619 | 1232 |
| AT1G18400 | -1.434736218 | 3.48E-06 | down | 61 | 167 |
| AT5G06640 | 0.946225023 | 3.74E-06 | up | 1954 | 1027 |
| AT3G42806 | -1.303697358 | 3.78E-06 | down | 88 | 220 |
| AT1G14200 | 1.103119635 | 4.20E-06 | up | 420 | 198 |
| AT5G03190 | 0.964938496 | 4.30E-06 | up | 1249 | 648 |
| AT3G28270 | 0.937787745 | 4.42E-06 | up | 2145 | 1134 |
| AT1G28660 | -0.958205791 | 4.94E-06 | down | 679 | 1336 |
| AT2G47800 | 0.925121333 | 5.26E-06 | up | 2805 | 1496 |
| AT4G21440 | 2.548745454 | 5.30E-06 | up | 52 | 9 |
| AT2G21660 | -0.91738636 | 5.59E-06 | down | 2337 | 4470 |
| AT4G21930 | 1.436374147 | 5.73E-06 | up | 155 | 58 |
| AT3G13277 | 1.093518864 | 5.84E-06 | up | 413 | 196 |
| AT4G34250 | 0.997197037 | 6.31E-06 | up | 749 | 380 |
| AT4G24700 | -1.21333333 | 6.44E-06 | down | 112 | 263 |
| AT5G66650 | 1.295340328 | 6.56E-06 | up | 206 | 85 |
| AT2G46680 | 0.936880792 | 6.79E-06 | up | 1586 | 839 |
| AT5G10040 | -1.632023224 | 7.19E-06 | down | 36 | 113 |
| AT4G33560 | -0.987155809 | 7.24E-06 | down | 401 | 805 |
| AT5G02502 | -1.168854816 | 7.69E-06 | down | 130 | 296 |
| AT4G13390 | 0.970821377 | 7.91E-06 | up | 898 | 464 |
| AT1G62510 | 0.907212908 | 7.91E-06 | up | 3963 | 2140 |
| AT2G47520 | -1.39976008 | 7.93E-06 | down | 61 | 163 |
| AT1G20430 | -1.033154649 | 8.41E-06 | down | 262 | 543 |
| AT2G29630 | 0.904848938 | 8.44E-06 | up | 4134 | 2236 |
| AT4G13310 | -2.09029372 | 8.56E-06 | down | 16 | 69 |
| AT2G46390 | -0.975011897 | 8.69E-06 | down | 428 | 852 |
| AT1G21250 | -0.966104668 | 9.22E-06 | down | 463 | 916 |
| AT1G58360 | 0.908451696 | 9.33E-06 | up | 2732 | 1474 |
| AT2G16650 | -1.031237939 | 9.49E-06 | down | 258 | 534 |
| AT2G04032 | 2.291249231 | 9.49E-06 | up | 58 | 12 |
| AT3G22235 | -1.035787801 | 9.66E-06 | down | 249 | 517 |
| AT5G25610 | 0.898122071 | 9.98E-06 | up | 10574 | 5746 |
| AT2G20750 | 1.004246508 | 1.02E-05 | up | 616 | 311 |
| AT1G49390 | -2.279449812 | 1.04E-05 | down | 12 | 59 |
| AT1G07135 | 1.515062328 | 1.04E-05 | up | 127 | 45 |
| AT2G28470 | 0.896124942 | 1.06E-05 | up | 5412 | 2945 |
| AT3G28345 | 0.917653782 | 1.16E-05 | up | 1662 | 891 |
| AT2G44230 | 0.939263903 | 1.23E-05 | up | 1102 | 582 |
| AT5G05400 | -1.926627709 | 1.24E-05 | down | 20 | 77 |
| AT3G62550 | 1.186736461 | 1.27E-05 | up | 254 | 113 |
| AT3G45140 | 0.890167758 | 1.30E-05 | up | 5408 | 2955 |
| AT1G15405 | -1.893232588 | 1.33E-05 | down | 21 | 79 |
| AT2G05510 | -0.903435789 | 1.33E-05 | down | 1211 | 2294 |
| AT5G45630 | 4.718670455 | 1.36E-05 | up | 26 | 1 |
| AT3G23800 | -1.159556382 | 1.47E-05 | down | 122 | 276 |
| AT4G28800 | 3.477662356 | 1.52E-05 | up | 33 | 3 |
| AT2G01023 | 2.39674236 | 1.54E-05 | up | 52 | 10 |
| AT4G29180 | 1.14947527 | 1.63E-05 | up | 276 | 126 |
| AT5G41020 | -0.961331516 | 1.71E-05 | down | 391 | 771 |
| AT2G43050 | -1.196135544 | 1.78E-05 | down | 103 | 239 |
| AT4G31351 | 1.531043452 | 1.78E-05 | up | 117 | 41 |
| AT5G65207 | -0.977377494 | 1.83E-05 | down | 329 | 656 |
| AT5G40800 | 1.6320481 | 1.86E-05 | up | 101 | 33 |
| AT4G02380 | -0.907382211 | 1.96E-05 | down | 786 | 1493 |
| AT2G43920 | -1.034962202 | 1.98E-05 | down | 213 | 442 |
| AT4G36648 | -0.893806325 | 2.02E-05 | down | 1082 | 2036 |
| AT4G39260 | -0.877509131 | 2.04E-05 | down | 3299 | 6138 |
| AT2G40140 | 0.910997514 | 2.10E-05 | up | 1296 | 698 |
| AT1G07430 | 2.240623158 | 2.12E-05 | up | 56 | 12 |
| AT4G02520 | -0.875347204 | 2.18E-05 | down | 4050 | 7524 |
| AT3G49320 | -1.295660039 | 2.24E-05 | down | 72 | 179 |
| AT3G56080 | 0.92313502 | 2.45E-05 | up | 998 | 533 |
| AT1G43800 | -0.87893957 | 2.48E-05 | down | 1519 | 2829 |
| AT5G50915 | 1.201452561 | 2.67E-05 | up | 218 | 96 |
| AT1G15125 | -1.22028135 | 2.72E-05 | down | 89 | 210 |
| AT2G27775 | -1.158896951 | 2.73E-05 | down | 111 | 251 |
| ATCG00170 | 1.298338656 | 2.79E-05 | up | 170 | 70 |
| AT2G22100 | -1.206400156 | 3.00E-05 | down | 92 | 215 |
| AT3G50840 | 0.955353534 | 3.16E-05 | up | 651 | 340 |
| AT3G62700 | 0.863433792 | 3.18E-05 | up | 5262 | 2929 |
| AT5G18700 | 0.912756068 | 3.27E-05 | up | 1002 | 539 |
| AT3G11410 | 0.882169188 | 3.32E-05 | up | 1729 | 950 |
| AT2G24850 | -1.869294534 | 3.37E-05 | down | 20 | 74 |
| AT1G65500 | -1.494581978 | 3.45E-05 | down | 41 | 117 |
| AT5G17270 | -0.949779149 | 3.61E-05 | down | 342 | 669 |
| AT3G05370 | -1.11105228 | 3.76E-05 | down | 128 | 280 |
| AT1G21460 | -0.96046575 | 3.77E-05 | down | 307 | 605 |
| AT1G13820 | -0.929301843 | 3.97E-05 | down | 406 | 783 |
| AT3G50060 | 1.081495631 | 4.09E-05 | up | 303 | 145 |
| AT4G36570 | -1.730230496 | 4.09E-05 | down | 25 | 84 |
| AT2G22200 | 1.232969586 | 4.17E-05 | up | 188 | 81 |
| AT2G31230 | -1.195073811 | 4.22E-05 | down | 91 | 211 |
| AT2G27385 | 0.865266625 | 4.56E-05 | up | 2101 | 1168 |
| AT3G01790 | -0.913903233 | 4.60E-05 | down | 457 | 872 |
| AT3G28950 | -0.998071075 | 4.70E-05 | down | 220 | 445 |
| AT4G18550 | 1.471403365 | 4.78E-05 | up | 115 | 42 |
| AT2G24980 | 0.88387835 | 4.97E-05 | up | 1250 | 686 |
| AT5G13630 | 0.874432289 | 5.16E-05 | up | 100725 | #### |
| AT4G17250 | 0.979114607 | 5.36E-05 | up | 473 | 243 |
| AT1G18810 | 0.963987692 | 5.37E-05 | up | 522 | 271 |
| AT5G22320 | -0.962803985 | 5.68E-05 | down | 268 | 529 |
| AT4G15210 | -1.669825257 | 5.74E-05 | down | 27 | 87 |
| AT3G22910 | 1.761455322 | 5.79E-05 | up | 77 | 23 |
| AT2G40765 | -0.904978009 | 5.79E-05 | down | 463 | 878 |
| AT2G19900 | 1.603193238 | 5.79E-05 | up | 93 | 31 |
| AT4G08400 | 1.010813266 | 5.81E-05 | up | 388 | 195 |
| AT2G41150 | -1.147017777 | 5.84E-05 | down | 103 | 231 |
| AT1G06160 | -1.899307103 | 5.90E-05 | down | 18 | 68 |
| AT5G24200 | -20 | 5.93E-05 | down | 0 | 19 |
| AT2G27400 | -1.056247479 | 6.01E-05 | down | 151 | 318 |
| AT1G19050 | -1.081304936 | 6.34E-05 | down | 133 | 285 |
| AT4G38840 | -0.892915872 | 6.40E-05 | down | 519 | 976 |
| AT1G79770 | -1.339960902 | 6.89E-05 | down | 55 | 141 |
| AT1G59960 | -0.917280536 | 7.43E-05 | down | 366 | 700 |
| AT2G17880 | 1.006735098 | 7.44E-05 | up | 375 | 189 |
| AT1G65450 | 1.374374547 | 7.76E-05 | up | 128 | 50 |
| AT3G26210 | -0.996490769 | 8.20E-05 | down | 195 | 394 |
| AT4G29780 | 1.058588475 | 8.42E-05 | up | 290 | 141 |
| AT2G43510 | -1.1121659 | 8.43E-05 | down | 111 | 243 |
| AT3G01820 | -0.889277583 | 8.93E-05 | down | 467 | 876 |
| AT1G48090 | 0.831007947 | 9.03E-05 | up | 5398 | 3073 |
| AT3G43510 | -1.051190664 | 9.36E-05 | down | 142 | 298 |
| ATMG00030 | -1.226572932 | 9.40E-05 | down | 73 | 173 |
| AT3G55710 | 1.06623611 | 9.53E-05 | up | 275 | 133 |
| AT5G47230 | 1.125145941 | 9.63E-05 | up | 224 | 104 |
| AT5G06320 | 0.87649383 | 9.90E-05 | up | 959 | 529 |
| AT2G46440 | -1.010831729 | 9.98E-05 | down | 172 | 351 |
| AT5G26800 | -0.968593874 | 1.03E-04 | down | 220 | 436 |
| AT5G65610 | -1.135243744 | 1.04E-04 | down | 98 | 218 |
| AT3G58980 | -1.23956702 | 1.05E-04 | down | 69 | 165 |
| AT4G33070 | -0.830676674 | 1.06E-04 | down | 1760 | 3170 |
| AT5G60680 | 0.845482062 | 1.09E-04 | up | 1588 | 895 |
| AT1G35240 | 1.723487472 | 1.09E-04 | up | 75 | 23 |
| AT2G32487 | -1.422341854 | 1.14E-04 | down | 42 | 114 |
| AT5G46330 | -0.918162609 | 1.14E-04 | down | 313 | 599 |
| AT5G55970 | 0.884857462 | 1.19E-04 | up | 795 | 436 |
| AT3G45160 | -0.860709877 | 1.19E-04 | down | 615 | 1131 |
| AT2G39310 | 0.824941272 | 1.21E-04 | up | 12311 | 7038 |
| AT1G14640 | 1.132563413 | 1.24E-04 | up | 210 | 97 |
| AT4G13575 | -0.87873423 | 1.26E-04 | down | 457 | 851 |
| AT2G26440 | -1.10559785 | 1.29E-04 | down | 106 | 231 |
| AT2G32220 | -1.308934006 | 1.29E-04 | down | 55 | 138 |
| AT3G18170 | 1.056270098 | 1.31E-04 | up | 269 | 131 |
| AT5G03350 | -0.876297717 | 1.33E-04 | down | 461 | 857 |
| AT1G58590 | -1.522337644 | 1.33E-04 | down | 33 | 96 |
| AT1G75580 | -1.638881549 | 1.38E-04 | down | 26 | 82 |
| AT1G64490 | -1.01876347 | 1.38E-04 | down | 154 | 316 |
| AT1G19510 | -2.166193834 | 1.42E-04 | down | 11 | 50 |
| AT4G15393 | -0.867257382 | 1.45E-04 | down | 498 | 920 |
| AT3G25670 | 1.456803751 | 1.46E-04 | up | 103 | 38 |
| AT4G31354 | 2.642721602 | 1.46E-04 | up | 37 | 6 |
| AT2G05380 | -0.820108904 | 1.47E-04 | down | 7278 | #### |
| AT5G09820 | -0.981769263 | 1.50E-04 | down | 185 | 370 |
| AT3G16770 | -0.820374421 | 1.52E-04 | down | 8442 | #### |
| AT4G12735 | -3.682208981 | 1.55E-04 | down | 2 | 26 |
| AT4G13280 | 1.064773323 | 1.57E-04 | up | 252 | 122 |
| AT1G51140 | 0.845510201 | 1.57E-04 | up | 1203 | 678 |
| AT5G38340 | -1.351003073 | 1.57E-04 | down | 48 | 124 |
| AT5G54880 | -1.351003073 | 1.57E-04 | down | 48 | 124 |
| AT1G21520 | -1.298441632 | 1.59E-04 | down | 55 | 137 |
| AT3G44990 | -0.815112631 | 1.60E-04 | down | 2177 | 3879 |
| AT2G14510 | -2.303697358 | 1.61E-04 | down | 9 | 45 |
| AT2G43590 | -0.829602422 | 1.61E-04 | down | 979 | 1762 |
| AT4G19550 | -1.481842866 | 1.68E-04 | down | 35 | 99 |
| AT3G21500 | -2.496342436 | 1.68E-04 | down | 7 | 40 |
| AT3G45680 | 0.946244624 | 1.69E-04 | up | 430 | 226 |
| AT1G05340 | 1.279190815 | 1.70E-04 | up | 139 | 58 |
| AT5G10140 | 1.358459649 | 1.71E-04 | up | 119 | 47 |
| AT1G32960 | -3.254787757 | 1.73E-04 | down | 3 | 29 |
| AT1G02340 | 0.838409699 | 1.82E-04 | up | 1243 | 704 |
| AT2G20520 | 1.292153459 | 1.92E-04 | up | 133 | 55 |
| AT1G72940 | 0.948857034 | 1.98E-04 | up | 406 | 213 |
| AT4G10910 | 2.143761619 | 2.10E-04 | up | 48 | 11 |
| AT1G24530 | -0.977641377 | 2.10E-04 | down | 175 | 349 |
| AT1G75830 | -20 | 2.12E-04 | down | 0 | 17 |
| AT5G45950 | 0.832274913 | 2.16E-04 | up | 1243 | 707 |
| AT3G21040 | 3.240623158 | 2.18E-04 | up | 28 | 3 |
| AT5G15960 | 0.947496226 | 2.20E-04 | up | 398 | 209 |
| AT2G03760 | -0.91624569 | 2.20E-04 | down | 259 | 495 |
| AT1G56600 | 0.848650507 | 2.22E-04 | up | 930 | 523 |
| AT3G07860 | -1.090703634 | 2.24E-04 | down | 102 | 220 |
| AT1G22890 | 1.085344933 | 2.24E-04 | up | 220 | 105 |
| AT3G20380 | -0.969696431 | 2.26E-04 | down | 180 | 357 |
| AT4G31290 | -0.805960079 | 2.30E-04 | down | 1643 | 2909 |
| AT3G13224 | -0.887771773 | 2.31E-04 | down | 325 | 609 |
| AT1G24260 | -1.519426049 | 2.34E-04 | down | 31 | 90 |
| AT1G60660 | -0.962246705 | 2.37E-04 | down | 186 | 367 |
| AT1G64660 | 0.819431333 | 2.52E-04 | up | 1462 | 839 |
| AT1G73500 | 0.975417654 | 2.53E-04 | up | 332 | 171 |
| AT4G13900 | 0.907527273 | 2.63E-04 | up | 489 | 264 |
| AT4G27520 | 0.797365785 | 2.64E-04 | up | 7695 | 4484 |
| AT1G32460 | -0.886191603 | 2.70E-04 | down | 312 | 584 |
| AT2G28780 | -0.85243604 | 2.71E-04 | down | 449 | 821 |
| AT5G21020 | -0.797276238 | 2.79E-04 | down | 5269 | 9273 |
| AT5G45340 | 1.147513754 | 2.91E-04 | up | 175 | 80 |
| AT2G18390 | -0.90454019 | 2.97E-04 | down | 259 | 491 |
| AT3G15357 | -1.113366146 | 2.99E-04 | down | 89 | 195 |
| AT1G71400 | -1.162341509 | 3.03E-04 | down | 75 | 170 |
| AT3G04210 | -0.898991587 | 3.03E-04 | down | 269 | 508 |
| AT5G19890 | -1.825043759 | 3.11E-04 | down | 17 | 61 |
| AT2G32300 | 0.921682249 | 3.23E-04 | up | 419 | 224 |
| AT4G10600 | -2.566731764 | 3.26E-04 | down | 6 | 36 |
| AT3G43190 | -0.790403737 | 3.31E-04 | down | 2415 | 4230 |
| AT1G72140 | 0.931643052 | 3.44E-04 | up | 388 | 206 |
| AT3G44970 | -1.151694264 | 3.47E-04 | down | 76 | 171 |
| AT1G03410 | -0.891485903 | 3.54E-04 | down | 272 | 511 |
| AT3G49570 | 1.140714744 | 3.62E-04 | up | 172 | 79 |
| AT1G06380 | -1.029074978 | 3.69E-04 | down | 120 | 248 |
| AT2G19810 | 0.857536683 | 3.69E-04 | up | 662 | 370 |
| AT4G11521 | -1.357636165 | 3.74E-04 | down | 42 | 109 |
| AT4G16500 | -0.870020905 | 3.77E-04 | down | 322 | 596 |
| AT4G34150 | 0.793751988 | 3.81E-04 | up | 2073 | 1211 |
| AT3G58850 | -1.09724648 | 3.94E-04 | down | 90 | 195 |
| AT1G58400 | -1.054748708 | 3.98E-04 | down | 106 | 223 |
| AT2G27402 | -0.821601808 | 4.03E-04 | down | 571 | 1022 |
| AT5G24140 | 1.271987329 | 4.18E-04 | up | 124 | 52 |
| AT1G02390 | 1.132302504 | 4.29E-04 | up | 171 | 79 |
| AT1G68650 | -0.932082139 | 4.33E-04 | down | 192 | 371 |
| AT5G60670 | -0.796123326 | 4.40E-04 | down | 940 | 1653 |
| AT4G03180 | -0.92605983 | 4.45E-04 | down | 198 | 381 |
| AT1G03060 | 0.779988793 | 4.46E-04 | up | 3815 | 2250 |
| AT3G23830 | -0.80763201 | 4.52E-04 | down | 686 | 1216 |
| AT4G31877 | 0.987203841 | 4.53E-04 | up | 276 | 141 |
| AT3G13940 | -0.876416263 | 4.55E-04 | down | 284 | 528 |
| AT5G42200 | 1.281265143 | 4.61E-04 | up | 120 | 50 |
| AT1G04700 | 1.052178069 | 4.62E-04 | up | 215 | 105 |
| AT2G43520 | -1.422341854 | 4.69E-04 | down | 35 | 95 |
| AT2G35950 | 1.368727984 | 4.69E-04 | up | 102 | 40 |
| AT2G19800 | -0.903057287 | 4.84E-04 | down | 226 | 428 |
| AT4G31398 | 2.018230737 | 4.97E-04 | up | 48 | 12 |
| AT3G54880 | -0.855452813 | 4.98E-04 | down | 334 | 612 |
| AT3G05727 | -0.779264472 | 5.03E-04 | down | 1657 | 2880 |
| AT1G24265 | -1.303697358 | 5.12E-04 | down | 46 | 115 |
| AT5G10800 | -1.070125137 | 5.15E-04 | down | 95 | 202 |
| AT1G76960 | 1.233243628 | 5.22E-04 | up | 130 | 56 |
| AT5G09970 | -1.470516448 | 5.26E-04 | down | 31 | 87 |
| AT1G18570 | 0.924617686 | 5.41E-04 | up | 358 | 191 |
| AT3G60330 | 0.825128604 | 5.59E-04 | up | 789 | 451 |
| AT1G26380 | 1.486379573 | 5.60E-04 | up | 83 | 30 |
| AT3G51600 | -0.772349184 | 5.61E-04 | down | 3386 | 5857 |
| AT5G55400 | 0.846750269 | 5.67E-04 | up | 618 | 348 |
| AT3G56070 | -0.799076965 | 5.76E-04 | down | 681 | 1200 |
| AT5G43720 | -0.860207283 | 6.02E-04 | down | 297 | 546 |
| AT4G28780 | 0.802502046 | 6.06E-04 | up | 1054 | 612 |
| AT1G34510 | 1.03869484 | 6.15E-04 | up | 213 | 105 |
| AT2G01870 | -1.086885969 | 6.29E-04 | down | 86 | 185 |
| AT1G31690 | 1.433268236 | 6.47E-04 | up | 88 | 33 |
| AT5G27830 | -0.999399157 | 6.52E-04 | down | 122 | 247 |
| AT5G37300 | 1.180502166 | 6.61E-04 | up | 141 | 63 |
| AT4G24570 | 1.046799889 | 6.67E-04 | up | 204 | 100 |
| AT5G15310 | 0.842459478 | 6.70E-04 | up | 602 | 340 |
| AT5G58240 | -0.887399312 | 6.76E-04 | down | 229 | 429 |
| AT3G10185 | 2.050652215 | 6.95E-04 | up | 45 | 11 |
| AT3G21340 | 2.050652215 | 6.95E-04 | up | 45 | 11 |
| AT4G12520 | 0.808068605 | 7.00E-04 | up | 880 | 509 |
| AT3G20470 | -0.77985945 | 7.08E-04 | down | 915 | 1591 |
| AT2G07709 | -0.929930331 | 7.28E-04 | down | 170 | 328 |
| AT5G02760 | -0.771683737 | 7.36E-04 | down | 1177 | 2035 |
| AT1G08580 | -0.824863208 | 7.43E-04 | down | 393 | 705 |
| AT3G59140 | 0.771096044 | 7.55E-04 | up | 1916 | 1137 |
| AT3G46560 | -0.826405201 | 7.60E-04 | down | 382 | 686 |
| AT4G37520 | -0.782172764 | 8.20E-04 | down | 743 | 1294 |
| AT3G53230 | 0.788042515 | 8.48E-04 | up | 1081 | 634 |
| AT2G43120 | 0.973492422 | 8.54E-04 | up | 254 | 131 |
| AT4G27380 | -0.866776741 | 8.62E-04 | down | 248 | 458 |
| AT5G44580 | -0.75868392 | 8.70E-04 | down | 1909 | 3271 |
| AT1G70320 | 0.760837213 | 8.75E-04 | up | 11617 | 6943 |
| AT1G11700 | -0.819764777 | 8.79E-04 | down | 386 | 690 |
| AT5G18670 | 0.757078856 | 8.85E-04 | up | 7413 | 4442 |
| AT5G42110 | -0.994047593 | 8.89E-04 | down | 117 | 236 |
| AT4G34710 | 0.76303174 | 8.93E-04 | up | 15447 | 9218 |
| AT3G20395 | -1.000794089 | 9.09E-04 | down | 113 | 229 |
| AT2G43800 | 0.774996574 | 9.17E-04 | up | 1345 | 796 |
| AT5G10930 | 0.78849634 | 9.25E-04 | up | 1008 | 591 |
| AT5G22250 | -1.449918099 | 9.26E-04 | down | 30 | 83 |
| AT4G02725 | -0.844512755 | 9.28E-04 | down | 292 | 531 |
| AT1G04900 | -0.897556259 | 9.35E-04 | down | 194 | 366 |
| AT2G27840 | -0.809924507 | 9.35E-04 | down | 423 | 751 |
| AT5G54190 | 1.011536085 | 9.41E-04 | up | 215 | 108 |
| AT1G66940 | -0.798746371 | 9.48E-04 | down | 491 | 865 |
| AT1G51860 | 0.900233528 | 9.53E-04 | up | 352 | 191 |
| AT3G48460 | 0.796074959 | 9.60E-04 | up | 871 | 508 |
| AT1G23205 | 0.831893912 | 9.66E-04 | up | 573 | 326 |
| ATCG00180 | 1.067699413 | 9.67E-04 | up | 178 | 86 |
| AT2G41210 | 0.939697335 | 9.70E-04 | up | 286 | 151 |
| AT1G70460 | 0.920534063 | 9.71E-04 | up | 314 | 168 |
| AT2G25000 | -0.919993065 | 9.76E-04 | down | 167 | 320 |
| AT2G07671 | -2.344339342 | 9.78E-04 | down | 7 | 36 |
| AT3G46700 | 0.784885371 | 9.81E-04 | up | 1031 | 606 |
| AT2G29090 | 1.032877513 | 9.84E-04 | up | 198 | 98 |
| AT3G44205 | -1.478195089 | 9.87E-04 | down | 28 | 79 |
| AT4G25630 | -0.763202726 | 9.92E-04 | down | 1067 | 1834 |
| AT3G57730 | -1.265562229 | 9.95E-04 | down | 46 | 112 |
| AT3G02260 | 0.754541442 | 0.001003 | up | 9544 | 5729 |
| AT1G72750 | -0.770452399 | 0.001024 | down | 811 | 1401 |
| AT5G38720 | -0.873853924 | 0.001029 | down | 222 | 412 |
| AT4G39800 | 0.754162613 | 0.001032 | up | 10231 | 6143 |
| AT5G43370 | 1.258057752 | 0.001045 | up | 111 | 47 |
| AT1G51080 | -1.103232775 | 0.00105 | down | 74 | 161 |
| AT1G78820 | 0.774171466 | 0.001063 | up | 1199 | 710 |
| AT1G66390 | -1.325723664 | 0.001079 | down | 39 | 99 |
| AT3G01260 | -1.090022154 | 0.001084 | down | 77 | 166 |
| AT2G25210 | -0.76454851 | 0.001085 | down | 905 | 1557 |
| AT1G18730 | -0.768077743 | 0.001107 | down | 799 | 1378 |
| AT2G19970 | -0.975795362 | 0.001118 | down | 121 | 241 |
| AT5G48412 | -0.774327183 | 0.001135 | down | 672 | 1164 |
| AT3G09450 | -0.92155174 | 0.00114 | down | 159 | 305 |
| AT4G27950 | -1.10646501 | 0.00115 | down | 72 | 157 |
| AT1G06830 | -1.162341509 | 0.001172 | down | 60 | 136 |
| AT3G47720 | -1.802799122 | 0.001177 | down | 15 | 53 |
| AT2G35420 | -1.802799122 | 0.001177 | down | 15 | 53 |
| AT3G25717 | -0.80698085 | 0.001185 | down | 390 | 691 |
| AT3G10710 | 0.911315533 | 0.001191 | up | 312 | 168 |
| AT3G06320 | -0.981769263 | 0.001195 | down | 116 | 232 |
| AT5G38780 | -1.727196436 | 0.001202 | down | 17 | 57 |
| ATMG01080 | -1.727196436 | 0.001202 | down | 17 | 57 |
| AT4G37070 | 0.79104153 | 0.001216 | up | 815 | 477 |
| AT2G44670 | -0.756359378 | 0.001218 | down | 1037 | 1774 |
| AT1G66660 | -1.081304936 | 0.00128 | down | 77 | 165 |
| AT1G12805 | -0.909619477 | 0.001315 | down | 164 | 312 |
| AT4G12510 | 0.969732219 | 0.001317 | up | 234 | 121 |
| AT3G05640 | 0.901015123 | 0.001341 | up | 319 | 173 |
| AT3G28900 | -0.757420645 | 0.00137 | down | 861 | 1474 |
| AT2G22122 | -1.131516382 | 0.001405 | down | 64 | 142 |
| AT5G15230 | -0.741470501 | 0.001419 | down | 4344 | 7355 |
| AT2G26190 | 0.777967639 | 0.001436 | up | 894 | 528 |
| AT3G26483 | -20 | 0.001459 | down | 0 | 14 |
| AT4G30930 | -0.802360012 | 0.001464 | down | 372 | 657 |
| AT3G32030 | 1.025966386 | 0.001465 | up | 187 | 93 |
| AT5G05340 | -0.975427736 | 0.001472 | down | 114 | 227 |
| AT4G15230 | 0.82848555 | 0.001488 | up | 498 | 284 |
| AT4G32480 | 0.738965458 | 0.001488 | up | 3123 | 1895 |
| AT2G33480 | -0.851708722 | 0.001498 | down | 232 | 424 |
| AT4G37220 | 1.652946273 | 0.0015 | up | 59 | 19 |
| AT1G65570 | 1.652946273 | 0.0015 | up | 59 | 19 |
| AT2G39855 | 2.773118239 | 0.001522 | up | 27 | 4 |
| AT2G15970 | 0.7355057 | 0.00158 | up | 4730 | 2877 |
| AT3G10570 | 0.936392445 | 0.001599 | up | 257 | 136 |
| AT2G20800 | -1.640732345 | 0.001647 | down | 19 | 60 |
| AT1G31200 | -1.775318385 | 0.001667 | down | 15 | 52 |
| AT2G31490 | -0.759920742 | 0.001677 | down | 663 | 1137 |
| AT2G44110 | 0.871126443 | 0.001681 | up | 354 | 196 |
| AT1G05135 | -0.773685454 | 0.001686 | down | 510 | 883 |
| AT1G58370 | -0.909256763 | 0.001723 | down | 153 | 291 |
| AT1G05900 | -0.855628233 | 0.001727 | down | 215 | 394 |
| AT4G11210 | 1.236135767 | 0.001727 | up | 107 | 46 |
| AT1G52240 | -1.163099028 | 0.00173 | down | 56 | 127 |
| AT1G23090 | -0.798255197 | 0.001733 | down | 360 | 634 |
| AT2G14900 | -0.975196609 | 0.001736 | down | 110 | 219 |
| AT2G14878 | -0.735020128 | 0.00177 | down | 1568 | 2643 |
| AT5G20790 | -0.732409794 | 0.001786 | down | 2079 | 3498 |
| AT1G10990 | -1.255944226 | 0.001788 | down | 43 | 104 |
| AT2G21140 | 0.74681342 | 0.001795 | up | 1430 | 863 |
| AT5G47640 | 0.740944669 | 0.001797 | up | 1746 | 1058 |
| AT2G33060 | 0.882347309 | 0.001823 | up | 324 | 178 |
| AT5G49560 | -1.107300145 | 0.001829 | down | 66 | 144 |
| AT5G61220 | -0.886421386 | 0.001832 | down | 172 | 322 |
| AT3G22231 | -0.919484985 | 0.001852 | down | 142 | 272 |
| AT4G31790 | -0.818886497 | 0.001887 | down | 281 | 502 |
| AT1G68238 | -1.012796159 | 0.001907 | down | 92 | 188 |
| AT3G08520 | -0.745136948 | 0.001968 | down | 823 | 1397 |
| AT5G24770 | 0.773003817 | 0.00198 | up | 788 | 467 |
| AT5G23940 | 0.734177041 | 0.001995 | up | 1976 | 1203 |
| AT2G47690 | -0.777628546 | 0.002011 | down | 432 | 750 |
| AT1G66930 | -2.017393173 | 0.002045 | down | 10 | 41 |
| AT1G62380 | -0.73968632 | 0.002052 | down | 12756 | #### |
| AT3G51895 | 0.728106721 | 0.002059 | up | 2761 | 1688 |
| AT3G48350 | 0.870553569 | 0.002064 | up | 334 | 185 |
| AT2G47460 | -1.022641544 | 0.002072 | down | 87 | 179 |
| AT2G10410 | -0.727342578 | 0.00208 | down | 1905 | 3194 |
| AT3G21010 | 1.222589236 | 0.002154 | up | 106 | 46 |
| AT4G25250 | 1.135587688 | 0.002177 | up | 128 | 59 |
| AT1G09090 | -0.854279528 | 0.002195 | down | 201 | 368 |
| AT4G22666 | 0.891059497 | 0.002211 | up | 293 | 160 |
| AT1G14250 | 0.916097952 | 0.002232 | up | 259 | 139 |
| AT1G55010 | -1.888659859 | 0.002245 | down | 12 | 45 |
| AT3G21150 | 0.822361758 | 0.002258 | up | 447 | 256 |
| AT3G44590 | -0.765651075 | 0.002277 | down | 478 | 823 |
| AT5G28510 | -2.981769263 | 0.002296 | down | 3 | 24 |
| AT1G10640 | 0.740303863 | 0.00233 | up | 1285 | 779 |
| AT2G34690 | -0.748510108 | 0.002367 | down | 633 | 1077 |
| AT5G20160 | -0.746529962 | 0.002379 | down | 658 | 1118 |
| ATCG00630 | -0.981769263 | 0.002454 | down | 99 | 198 |
| AT5G42600 | 0.753618753 | 0.002499 | up | 914 | 549 |
| AT2G17930 | 0.718559733 | 0.002534 | up | 4886 | 3007 |
| AT5G40380 | 0.779871567 | 0.002552 | up | 629 | 371 |
| AT3G45700 | 1.160675002 | 0.002603 | up | 117 | 53 |
| AT4G01985 | 2.255269934 | 0.002662 | up | 33 | 7 |
| AT3G13784 | 2.255269934 | 0.002662 | up | 33 | 7 |
| AT3G21030 | 2.255269934 | 0.002662 | up | 33 | 7 |
| AT3G02780 | -0.72072341 | 0.002664 | down | 1450 | 2420 |
| AT1G14210 | -0.756435566 | 0.002735 | down | 491 | 840 |
| AT2G29980 | -0.717748062 | 0.002736 | down | 1755 | 2923 |
| AT5G20710 | 1.578945692 | 0.00275 | up | 59 | 20 |
| AT5G18250 | -1.025623553 | 0.002774 | down | 81 | 167 |
| AT5G23740 | -0.715719271 | 0.002797 | down | 2203 | 3664 |
| AT1G16970 | -0.804891501 | 0.002797 | down | 273 | 483 |
| AT1G49520 | -0.967555404 | 0.002818 | down | 102 | 202 |
| AT4G39950 | 0.713823228 | 0.002875 | up | 4457 | 2752 |
| AT2G32060 | -0.715332638 | 0.002884 | down | 1868 | 3106 |
| AT2G40510 | -0.723457267 | 0.002903 | down | 1037 | 1734 |
| AT3G10720 | 0.722340503 | 0.002926 | up | 1722 | 1057 |
| AT1G52930 | -0.756304008 | 0.002934 | down | 470 | 804 |
| AT5G63000 | -0.890621375 | 0.002949 | down | 147 | 276 |
| AT2G02100 | -0.724555272 | 0.003009 | down | 940 | 1573 |
| AT2G31141 | -1.981769263 | 0.003021 | down | 10 | 40 |
| AT5G45820 | -0.937179112 | 0.003033 | down | 115 | 223 |
| AT4G32460 | -0.718734857 | 0.003053 | down | 1182 | 1970 |
| AT1G07830 | -0.788452696 | 0.003084 | down | 307 | 537 |
| AT1G10760 | 0.712173122 | 0.003136 | up | 2797 | 1729 |
| AT3G25940 | -1.063069365 | 0.003154 | down | 69 | 146 |
| AT2G45860 | -0.873457452 | 0.003171 | down | 159 | 295 |
| AT3G59650 | -0.892718075 | 0.003209 | down | 142 | 267 |
| AT5G08185 | -0.879199529 | 0.003225 | down | 153 | 285 |
| AT2G04845 | -1.027283922 | 0.00323 | down | 78 | 161 |
| AT5G55720 | 1.494668781 | 0.003236 | up | 64 | 23 |
| AT2G30210 | 0.812007253 | 0.003238 | up | 423 | 244 |
| AT5G05250 | 1.047574699 | 0.003265 | up | 149 | 73 |
| AT1G63310 | -0.941876536 | 0.003331 | down | 110 | 214 |
| AT2G44140 | 0.723556252 | 0.003341 | up | 1368 | 839 |
| AT2G32100 | 0.783494326 | 0.003349 | up | 532 | 313 |
| AT5G11260 | -0.800831269 | 0.003362 | down | 263 | 464 |
| AT2G25720 | -1.039102438 | 0.003384 | down | 74 | 154 |
| AT4G24110 | -0.837183641 | 0.003541 | down | 194 | 351 |
| AT5G62930 | -0.754854283 | 0.003558 | down | 426 | 728 |
| AT5G07322 | -1.017838518 | 0.003595 | down | 79 | 162 |
| AT3G22750 | -0.768130677 | 0.003609 | down | 356 | 614 |
| AT3G15500 | 1.426315476 | 0.003648 | up | 69 | 26 |
| AT1G08890 | 0.899586241 | 0.003688 | up | 245 | 133 |
| AT3G25730 | -1.116928846 | 0.003752 | down | 56 | 123 |
| AT1G21670 | 0.770436209 | 0.00381 | up | 571 | 339 |
| AT1G19610 | -2.920368718 | 0.003824 | down | 3 | 23 |
| AT1G61570 | -0.773776221 | 0.003835 | down | 324 | 561 |
| AT5G05370 | -0.800619824 | 0.003837 | down | 250 | 441 |
| AT1G34315 | -1.190355885 | 0.003852 | down | 45 | 104 |
| AT2G14610 | -1.13931054 | 0.003882 | down | 52 | 116 |
| AT3G46020 | -1.2241301 | 0.003898 | down | 41 | 97 |
| AT2G33850 | 0.720103549 | 0.003901 | up | 1259 | 774 |
| AT4G22990 | 0.800639302 | 0.003901 | up | 430 | 250 |
| AT4G22070 | 1.135800333 | 0.003901 | up | 115 | 53 |
| AT5G48010 | 0.702287653 | 0.003919 | up | 4203 | 2616 |
| AT3G57710 | -0.854657345 | 0.004032 | down | 166 | 304 |
| AT1G11600 | 0.765996863 | 0.004047 | up | 581 | 346 |
| AT4G25810 | 0.952476292 | 0.004058 | up | 193 | 101 |
| AT3G46330 | -3.303697358 | 0.004093 | down | 2 | 20 |
| AT2G20870 | 2.662086927 | 0.004098 | up | 25 | 4 |
| AT4G25470 | 2.662086927 | 0.004098 | up | 25 | 4 |
| AT2G07711 | -1.27127588 | 0.004121 | down | 36 | 88 |
| AT5G04370 | 1.102764247 | 0.004134 | up | 123 | 58 |
| AT3G29034 | -0.990230842 | 0.004185 | down | 85 | 171 |
| AT5G59613 | -0.703587239 | 0.004198 | down | 1417 | 2337 |
| AT5G66740 | 1.061299459 | 0.004257 | up | 136 | 66 |
| AT1G77885 | -0.83060519 | 0.004262 | down | 191 | 344 |
| AT1G43790 | -0.858710867 | 0.004284 | down | 159 | 292 |
| AT1G58380 | -0.725298967 | 0.004344 | down | 614 | 1028 |
| AT1G69880 | -1.145267995 | 0.004361 | down | 50 | 112 |
| AT5G62210 | 0.925121333 | 0.004409 | up | 210 | 112 |
| AT1G63360 | -0.736222284 | 0.004427 | down | 492 | 830 |
| AT2G19990 | -1.009249999 | 0.004438 | down | 78 | 159 |
| ATMG01380 | -1.945243387 | 0.004448 | down | 10 | 39 |
| AT3G02850 | 1.161188691 | 0.004466 | up | 106 | 48 |
| AT1G02800 | 1.018230737 | 0.004479 | up | 152 | 76 |
| AT3G14067 | 0.70125175 | 0.004538 | up | 9169 | 5711 |
| AT5G27100 | -0.966001947 | 0.00455 | down | 92 | 182 |
| AT1G14060 | -0.800577702 | 0.004604 | down | 233 | 411 |
| AT1G68840 | 0.69657738 | 0.004624 | up | 3151 | 1969 |
| AT5G60730 | -1.148419132 | 0.004626 | down | 49 | 110 |
| AT5G51220 | -0.789124185 | 0.004647 | down | 256 | 448 |
| AT3G10860 | -0.698763783 | 0.00466 | down | 1513 | 2487 |
| AT3G03770 | 0.700040376 | 0.004683 | up | 2087 | 1301 |
| AT1G26410 | 1.734437771 | 0.004709 | up | 46 | 14 |
| AT3G45100 | -0.782263271 | 0.004711 | down | 271 | 472 |
| AT2G45210 | -0.965827719 | 0.004786 | down | 91 | 180 |
| AT5G18540 | -1.294927148 | 0.004891 | down | 33 | 82 |
| AT4G28480 | -0.732353668 | 0.004948 | down | 485 | 816 |
| AT4G33610 | 0.743241198 | 0.004959 | up | 681 | 412 |
| AT5G65890 | 0.794823405 | 0.005073 | up | 406 | 237 |
| AT1G49310 | -1.10952481 | 0.005108 | down | 54 | 118 |
| AT4G08770 | -0.745991076 | 0.00512 | down | 388 | 659 |
| AT1G63880 | -0.727741176 | 0.005132 | down | 511 | 857 |
| AT2G21770 | 1.418768667 | 0.005145 | up | 66 | 25 |
| AT4G13520 | -0.725889186 | 0.0052 | down | 523 | 876 |
| AT5G35732 | -1.074878667 | 0.005214 | down | 60 | 128 |
| AT1G27370 | -0.770526966 | 0.005226 | down | 290 | 501 |
| AT3G56020 | -0.703604368 | 0.005243 | down | 901 | 1486 |
| AT1G73680 | 0.722478363 | 0.005258 | up | 901 | 553 |
| AT2G23600 | -0.700443708 | 0.005262 | down | 1016 | 1672 |
| AT4G12600 | -0.697138312 | 0.005267 | down | 1198 | 1967 |
| AT5G11910 | -0.822892088 | 0.005323 | down | 187 | 335 |
| AT5G53980 | -0.973357023 | 0.005354 | down | 86 | 171 |
| AT4G02410 | 0.735875721 | 0.005363 | up | 717 | 436 |
| AT5G63600 | -0.70632607 | 0.005384 | down | 794 | 1312 |
| AT1G53700 | -0.93139574 | 0.005523 | down | 102 | 197 |
| AT1G44830 | -1.517822163 | 0.005552 | down | 20 | 58 |
| AT1G20160 | 0.708375474 | 0.005591 | up | 1152 | 714 |
| AT5G18840 | -0.891003799 | 0.005644 | down | 123 | 231 |
| ATMG00020 | -0.735768054 | 0.005649 | down | 421 | 710 |
| AT2G36170 | -0.694445654 | 0.005684 | down | 1174 | 1924 |
| AT1G21240 | 3.266158251 | 0.005737 | up | 19 | 2 |
| AT4G21585 | -1.114219559 | 0.005761 | down | 52 | 114 |
| AT4G33150 | 0.698006501 | 0.005767 | up | 1525 | 952 |
| AT3G16720 | 0.842131968 | 0.005884 | up | 285 | 161 |
| AT2G25510 | -0.687171006 | 0.005934 | down | 2255 | 3677 |
| AT1G07090 | -0.810002915 | 0.006047 | down | 196 | 348 |
| AT5G51060 | 0.735208153 | 0.006079 | up | 669 | 407 |
| AT1G12160 | -0.85992814 | 0.006126 | down | 142 | 261 |
| AT5G41010 | -0.811376992 | 0.006143 | down | 193 | 343 |
| AT4G27410 | 0.803405424 | 0.006157 | up | 355 | 206 |
| AT5G37410 | -1.59474614 | 0.006178 | down | 17 | 52 |
| AT4G21610 | -0.964895444 | 0.006205 | down | 86 | 170 |
| AT3G10110 | -1.022991926 | 0.006213 | down | 69 | 142 |
| AT2G30750 | 0.982161482 | 0.006227 | up | 158 | 81 |
| AT2G31083 | 1.320793507 | 0.006316 | up | 74 | 30 |
| AT3G27831 | 2.165072125 | 0.006336 | up | 31 | 7 |
| AT3G45440 | 1.383880209 | 0.006375 | up | 67 | 26 |
| AT5G47990 | 0.683585534 | 0.006412 | up | 4179 | 2635 |
| AT5G20080 | -0.692500695 | 0.006424 | down | 1010 | 1653 |
| AT4G37260 | 0.764474145 | 0.006577 | up | 468 | 279 |
| AT3G53160 | -1.697976297 | 0.006626 | down | 14 | 46 |
| AT5G48240 | -0.769398761 | 0.006661 | down | 263 | 454 |
| AT4G27260 | 0.683111709 | 0.006679 | up | 2700 | 1703 |
| AT3G19580 | 0.934017733 | 0.006703 | up | 183 | 97 |
| AT5G57340 | 0.741312377 | 0.006712 | up | 586 | 355 |
| AT2G37450 | -0.740393738 | 0.00672 | down | 357 | 604 |
| AT5G09978 | -1.144707834 | 0.006824 | down | 46 | 103 |
| AT2G23180 | -0.831743819 | 0.006894 | down | 162 | 292 |
| AT3G54530 | -3.229696776 | 0.007012 | down | 2 | 19 |
| AT5G44980 | -3.229696776 | 0.007012 | down | 2 | 19 |
| AT4G13495 | -0.681030405 | 0.007031 | down | 1884 | 3059 |
| AT4G31985 | -0.685042366 | 0.007082 | down | 1213 | 1975 |
| AT3G44750 | -0.690943791 | 0.007086 | down | 912 | 1491 |
| AT4G32720 | -0.687947073 | 0.007131 | down | 1023 | 1669 |
| AT1G75670 | -0.878458546 | 0.007133 | down | 123 | 229 |
| AT1G59660 | 0.769774796 | 0.007142 | up | 431 | 256 |
| AT5G57050 | 0.746235464 | 0.007196 | up | 535 | 323 |
| AT3G02170 | 0.679177188 | 0.007198 | up | 3333 | 2108 |
| AT4G04223 | 1.136067227 | 0.007298 | up | 102 | 47 |
| AT1G66090 | 1.525190726 | 0.0074 | up | 54 | 19 |
| AT2G23940 | -0.783543153 | 0.007451 | down | 222 | 387 |
| AT3G43980 | -0.749822535 | 0.00747 | down | 303 | 516 |
| AT3G19508 | -1.251858426 | 0.007484 | down | 34 | 82 |
| AT4G08780 | -1.084862756 | 0.007493 | down | 54 | 116 |
| AT1G15870 | -1.492731182 | 0.007501 | down | 20 | 57 |
| AT5G37500 | 0.819546743 | 0.00761 | up | 298 | 171 |
| AT5G59950 | -0.69435295 | 0.007704 | down | 728 | 1193 |
| AT3G03010 | -0.858194578 | 0.007711 | down | 134 | 246 |
| AT4G16260 | -0.681656753 | 0.007715 | down | 1206 | 1959 |
| AT4G26950 | -1.382307193 | 0.007724 | down | 25 | 66 |
| AT3G59900 | -0.828757644 | 0.007742 | down | 159 | 286 |
| AT1G13145 | -1.151694264 | 0.007748 | down | 44 | 99 |
| AT1G17870 | 0.752218631 | 0.007808 | up | 484 | 291 |
| AT1G26970 | 1.350806076 | 0.007816 | up | 68 | 27 |
| AT4G11880 | -1.106097398 | 0.007942 | down | 50 | 109 |
| AT1G56630 | 1.188155739 | 0.007953 | up | 90 | 40 |
| AT5G64000 | -0.784323199 | 0.007956 | down | 215 | 375 |
| AT4G22305 | -1.069232104 | 0.007983 | down | 56 | 119 |
| AT5G23110 | 0.675268942 | 0.008014 | up | 2996 | 1900 |
| AT3G50520 | -0.785230269 | 0.008098 | down | 212 | 370 |
| AT3G24480 | 0.674381291 | 0.008105 | up | 3749 | 2379 |
| AT5G55960 | 0.685594608 | 0.008203 | up | 1396 | 879 |
| AT3G49400 | 0.895082506 | 0.008203 | up | 202 | 110 |
| AT1G47580 | -0.772219147 | 0.008342 | down | 233 | 403 |
| AT2G39675 | -0.879588868 | 0.008362 | down | 117 | 218 |
| AT2G38080 | 0.768252484 | 0.008411 | up | 407 | 242 |
| AT2G17845 | -2.566731764 | 0.008424 | down | 4 | 24 |
| AT5G06550 | -0.793324174 | 0.008466 | down | 196 | 344 |
| AT1G22900 | -1.108526405 | 0.008466 | down | 49 | 107 |
| AT1G79075 | -0.796657858 | 0.0085 | down | 191 | 336 |
| AT3G52060 | 0.672500609 | 0.008534 | up | 4232 | 2689 |
| AT5G11460 | -0.920368718 | 0.00855 | down | 96 | 184 |
| AT1G16000 | -0.828724888 | 0.008555 | down | 154 | 277 |
| AT2G27330 | -0.859285256 | 0.008602 | down | 129 | 237 |
| AT1G55240 | 1.084726149 | 0.008621 | up | 111 | 53 |
| AT5G27990 | -0.741189708 | 0.00881 | down | 306 | 518 |
| AT4G23700 | 0.725006677 | 0.009054 | up | 599 | 367 |
| AT3G13437 | -1.629467519 | 0.009075 | down | 15 | 47 |
| AT1G60505 | -0.925812857 | 0.009205 | down | 92 | 177 |
| AT3G50800 | 1.228797723 | 0.009214 | up | 81 | 35 |
| AT5G23155 | -1.941127278 | 0.009295 | down | 9 | 35 |
| AT4G15290 | 0.934814729 | 0.009359 | up | 168 | 89 |
| AT4G26790 | 0.871605823 | 0.009386 | up | 215 | 119 |
| AT5G36910 | 0.741437555 | 0.009538 | up | 487 | 295 |
| AT1G26440 | -0.747923325 | 0.009563 | down | 274 | 466 |
| AT5G39680 | -1.754358767 | 0.009603 | down | 12 | 41 |
| AT2G39040 | 1.124025401 | 0.009691 | up | 99 | 46 |
| AT5G52640 | 0.689240978 | 0.009724 | up | 1019 | 640 |
| AT5G65990 | 0.693467341 | 0.009883 | up | 915 | 573 |
| AT1G54450 | -1.015192264 | 0.009915 | down | 64 | 131 |
| AT5G23380 | -0.781928684 | 0.009928 | down | 201 | 350 |
| AT4G34760 | -0.741915227 | 0.010064 | down | 284 | 481 |
| AT3G02790 | -0.731421956 | 0.010132 | down | 317 | 533 |
| AT3G06700 | -0.665916015 | 0.010178 | down | 2281 | 3665 |
| AT4G26230 | -0.66725864 | 0.010204 | down | 1552 | 2496 |
| AT1G61960 | -0.962910236 | 0.010235 | down | 77 | 152 |
| AT2G11140 | -1.058390545 | 0.010243 | down | 55 | 116 |
| AT4G34750 | -0.924544394 | 0.010254 | down | 90 | 173 |
| AT1G25260 | -0.672380364 | 0.010333 | down | 1031 | 1664 |
| AT5G36220 | -0.752682189 | 0.010382 | down | 252 | 430 |
| AT4G27450 | -0.665040581 | 0.010436 | down | 2562 | 4114 |
| AT1G29395 | 0.786415062 | 0.010473 | up | 327 | 192 |
| AT1G69930 | -2.789124185 | 0.010486 | down | 3 | 21 |
| AT1G19240 | -0.93114319 | 0.010497 | down | 87 | 168 |
| AT1G32780 | -1.204161684 | 0.010505 | down | 36 | 84 |
| AT5G55200 | -0.765831864 | 0.010548 | down | 223 | 384 |
| AT5G46790 | -0.734144296 | 0.010758 | down | 298 | 502 |
| AT5G43190 | 0.710108442 | 0.01077 | up | 651 | 403 |
| AT3G15395 | -0.812414835 | 0.010775 | down | 158 | 281 |
| AT1G22190 | 0.677632071 | 0.01088 | up | 1213 | 768 |
| AT5G39890 | -0.728807666 | 0.010895 | down | 314 | 527 |
| AT1G43910 | -0.843164025 | 0.010964 | down | 131 | 238 |
| AT5G06730 | -1.119272787 | 0.011006 | down | 45 | 99 |
| AT5G52380 | -0.857321892 | 0.011084 | down | 121 | 222 |
| AT3G17520 | 1.416780114 | 0.011144 | up | 58 | 22 |
| AT5G50800 | 0.82143003 | 0.011155 | up | 260 | 149 |
| AT4G15770 | -0.691068114 | 0.01119 | down | 537 | 878 |
| AT3G50460 | -3.888659859 | 0.011203 | down | 1 | 15 |
| AT1G32630 | -1.016257639 | 0.011231 | down | 62 | 127 |
| AT1G49160 | 0.84620727 | 0.011242 | up | 229 | 129 |
| AT5G57530 | 0.892699855 | 0.011319 | up | 187 | 102 |
| AT5G53120 | 0.666804132 | 0.011426 | up | 1704 | 1087 |
| AT4G38100 | -0.723153528 | 0.011461 | down | 326 | 545 |
| AT5G18810 | -2.204161684 | 0.01156 | down | 6 | 28 |
| AT2G26400 | -2.204161684 | 0.01156 | down | 6 | 28 |
| AT2G39030 | -2.204161684 | 0.01156 | down | 6 | 28 |
| AT2G44798 | -1.147779214 | 0.011685 | down | 41 | 92 |
| AT5G14690 | -0.864199667 | 0.011686 | down | 115 | 212 |
| AT2G40800 | -0.702270256 | 0.01182 | down | 423 | 697 |
| AT2G01940 | 0.79042482 | 0.011901 | up | 304 | 178 |
| AT5G41765 | -3.151694264 | 0.011958 | down | 2 | 18 |
| AT4G21830 | -0.816710017 | 0.012098 | down | 148 | 264 |
| AT3G17609 | -0.704343765 | 0.012118 | down | 403 | 665 |
| AT5G33370 | 0.721759372 | 0.012124 | up | 526 | 323 |
| AT3G61890 | 0.693664829 | 0.012135 | up | 765 | 479 |
| AT4G01390 | -0.701415381 | 0.012203 | down | 419 | 690 |
| AT3G10520 | -0.789124185 | 0.0123 | down | 176 | 308 |
| AT2G20450 | -0.668686407 | 0.01232 | down | 874 | 1407 |
| AT5G01180 | -1.0458996 | 0.012324 | down | 55 | 115 |
| AT1G61420 | -1.0458996 | 0.012324 | down | 55 | 115 |
| AT2G29010 | 0.846862319 | 0.012332 | up | 222 | 125 |
| AT5G42580 | 0.698828517 | 0.012594 | up | 686 | 428 |
| AT3G23550 | -1.598440623 | 0.012603 | down | 15 | 46 |
| AT5G62920 | -0.790315019 | 0.012606 | down | 173 | 303 |
| AT3G62150 | -0.732069496 | 0.012682 | down | 280 | 471 |
| AT3G07910 | -0.728684517 | 0.012776 | down | 289 | 485 |
| AT3G49510 | -1.63384596 | 0.013029 | down | 14 | 44 |
| AT4G28300 | 0.656099901 | 0.013096 | up | 2555 | 1642 |
| AT5G23330 | -0.768365625 | 0.013099 | down | 200 | 345 |
| AT4G01897 | -0.814312517 | 0.013169 | down | 146 | 260 |
| AT5G04140 | 0.685652021 | 0.013372 | up | 50258 | #### |
| AT5G46500 | -1.673646968 | 0.013392 | down | 13 | 42 |
| AT1G67148 | -1.104626011 | 0.013503 | down | 45 | 98 |
| AT3G28750 | -2.505331219 | 0.01351 | down | 4 | 23 |
| AT3G28650 | -2.505331219 | 0.01351 | down | 4 | 23 |
| AT4G13340 | 0.655660658 | 0.013515 | up | 2282 | 1467 |
| AT3G19130 | -0.663202482 | 0.013654 | down | 911 | 1461 |
| AT5G07040 | -1.899307103 | 0.013675 | down | 9 | 34 |
| AT4G28290 | 0.790820241 | 0.013857 | up | 287 | 168 |
| AT5G48580 | -0.67239354 | 0.01391 | down | 658 | 1062 |
| AT5G57770 | -0.818270531 | 0.013919 | down | 140 | 250 |
| AT3G47010 | -0.913865198 | 0.013939 | down | 87 | 166 |
| AT4G29480 | -0.660390871 | 0.014 | down | 984 | 1575 |
| AT1G69270 | 0.700979129 | 0.01403 | up | 618 | 385 |
| AT3G28160 | -0.683294449 | 0.014047 | down | 511 | 831 |
| AT2G24240 | 0.966515701 | 0.014074 | up | 137 | 71 |
| AT1G20100 | -0.664695017 | 0.014092 | down | 816 | 1310 |
| AT5G65740 | -0.851708722 | 0.014097 | down | 116 | 212 |
| AT2G29620 | 1.075946235 | 0.014181 | up | 102 | 49 |
| AT4G32800 | -1.461762204 | 0.014318 | down | 19 | 53 |
| AT1G22270 | -0.738379386 | 0.014327 | down | 248 | 419 |
| AT4G05100 | 0.860439491 | 0.014532 | up | 199 | 111 |
| AT5G20190 | -0.652648707 | 0.014635 | down | 1483 | 2361 |
| AT5G27400 | -0.774408455 | 0.014636 | down | 183 | 317 |
| AT4G18670 | 0.650558949 | 0.014659 | up | 3607 | 2327 |
| AT4G38780 | 0.975162015 | 0.014733 | up | 132 | 68 |
| AT4G04614 | -0.761170123 | 0.014852 | down | 201 | 345 |
| AT1G77750 | -0.751599491 | 0.014877 | down | 217 | 370 |
| AT4G26080 | 0.651383925 | 0.01495 | up | 2283 | 1472 |
| AT2G23430 | -0.728428253 | 0.014999 | down | 267 | 448 |
| AT3G50190 | -1.484269603 | 0.015097 | down | 18 | 51 |
| AT1G68550 | -0.695107798 | 0.015236 | down | 394 | 646 |
| AT4G29310 | -0.714945767 | 0.015292 | down | 305 | 507 |
| AT5G38840 | -0.690357595 | 0.015353 | down | 421 | 688 |
| AT1G52710 | -0.887647086 | 0.015428 | down | 95 | 178 |
| AT1G56045 | -0.652645667 | 0.015441 | down | 1206 | 1920 |
| AT3G22210 | -0.682912678 | 0.015503 | down | 473 | 769 |
| AT5G56940 | -0.72289548 | 0.015585 | down | 277 | 463 |
| AT1G25275 | -0.676914681 | 0.01566 | down | 525 | 850 |
| AT1G53760 | -0.779506182 | 0.015664 | down | 172 | 299 |
| AT4G01430 | -0.942775131 | 0.015664 | down | 75 | 146 |
| AT1G19380 | -1.034236683 | 0.01583 | down | 54 | 112 |
| AT1G23290 | -0.647843455 | 0.015853 | down | 2098 | 3329 |
| AT4G35560 | 0.680880648 | 0.015888 | up | 763 | 482 |
| AT2G29940 | 0.784273564 | 0.015894 | up | 284 | 167 |
| AT1G56570 | -1.050810907 | 0.01611 | down | 51 | 107 |
| AT5G03552 | -0.918838007 | 0.016117 | down | 82 | 157 |
| AT1G61370 | -0.951395614 | 0.016128 | down | 72 | 141 |
| AT2G26530 | 0.79352445 | 0.016228 | up | 267 | 156 |
| AT4G08115 | -1.204161684 | 0.016278 | down | 33 | 77 |
| AT4G02075 | 0.857376726 | 0.01628 | up | 195 | 109 |
| AT3G61620 | -0.744613489 | 0.016324 | down | 221 | 375 |
| AT1G67030 | -0.724148353 | 0.01645 | down | 266 | 445 |
| AT1G21270 | -0.670520867 | 0.016477 | down | 572 | 922 |
| AT1G70260 | 0.900049311 | 0.01657 | up | 164 | 89 |
| AT2G37040 | 0.651239903 | 0.016604 | up | 8472 | 5463 |
| AT3G49790 | 0.705072852 | 0.016649 | up | 528 | 328 |
| AT4G16960 | -0.880622539 | 0.016666 | down | 96 | 179 |
| AT1G27435 | -0.721937289 | 0.016691 | down | 270 | 451 |
| AT5G45600 | -0.750143562 | 0.016692 | down | 209 | 356 |
| AT1G29430 | -0.744966399 | 0.016723 | down | 218 | 370 |
| AT1G17450 | 0.67327951 | 0.016837 | up | 833 | 529 |
| AT4G28420 | 3.825585659 | 0.017036 | up | 14 | 1 |
| AT5G58760 | -0.718734857 | 0.017048 | down | 276 | 460 |
| AT3G25120 | -0.762570766 | 0.017052 | down | 188 | 323 |
| AT4G26690 | 0.643981352 | 0.01719 | up | 3103 | 2011 |
| AT4G08100 | -1.315192997 | 0.017295 | down | 25 | 63 |
| AT2G24110 | -1.566731764 | 0.017422 | down | 15 | 45 |
| AT5G14105 | -0.820973051 | 0.017463 | down | 128 | 229 |
| AT5G35935 | 0.725589869 | 0.01751 | up | 418 | 256 |
| AT2G45600 | 0.795036596 | 0.017651 | up | 257 | 150 |
| AT3G55700 | -2.151694264 | 0.017715 | down | 6 | 27 |
| AT2G23810 | 0.64964216 | 0.017853 | up | 1515 | 978 |
| AT1G55860 | 0.644708993 | 0.017974 | up | 5993 | 3882 |
| AT5G54370 | 0.671580213 | 0.017976 | up | 810 | 515 |
| AT2G01730 | -1.41472867 | 0.018118 | down | 20 | 54 |
| AT2G33847 | -1.600679096 | 0.018144 | down | 14 | 43 |
| AT4G28040 | 0.666802626 | 0.018188 | up | 881 | 562 |
| AT5G23360 | -1.261877182 | 0.018189 | down | 28 | 68 |
| AT5G47980 | 0.7089553 | 0.01819 | up | 481 | 298 |
| AT1G74270 | -0.651111684 | 0.018197 | down | 891 | 1417 |
| AT2G36160 | -0.642099586 | 0.018199 | down | 2250 | 3556 |
| AT4G13800 | 1.005739793 | 0.018246 | up | 115 | 58 |
| AT3G55170 | -0.682745 | 0.018419 | down | 414 | 673 |
| AT3G19030 | 0.681017914 | 0.018435 | up | 676 | 427 |
| AT5G18920 | -1.3275441 | 0.018497 | down | 24 | 61 |
| AT2G44860 | -0.650723272 | 0.01856 | down | 873 | 1388 |
| AT4G26670 | -0.909619477 | 0.018615 | down | 82 | 156 |
| AT3G13610 | -0.691813558 | 0.018869 | down | 357 | 584 |
| AT4G18250 | -2.032395336 | 0.018936 | down | 7 | 29 |
| AT3G25882 | -2.032395336 | 0.018936 | down | 7 | 29 |
| AT4G04700 | -0.993943977 | 0.0193 | down | 59 | 119 |
| AT2G33370 | -0.641597919 | 0.01939 | down | 1333 | 2106 |
| AT4G12090 | -0.824457258 | 0.019579 | down | 121 | 217 |
| AT1G69730 | -0.667967058 | 0.019608 | down | 509 | 819 |
| AT3G23880 | 0.711093049 | 0.019669 | up | 451 | 279 |
| AT1G77570 | -1.73179101 | 0.01978 | down | 11 | 37 |
| AT5G50790 | -3.789124185 | 0.019873 | down | 1 | 14 |
| AT5G44710 | -0.755493407 | 0.019947 | down | 186 | 318 |
| AT3G23920 | 0.639192006 | 0.019948 | up | 5052 | 3285 |
| AT1G50400 | -1.856238381 | 0.020001 | down | 9 | 33 |
| AT2G35480 | -0.981769263 | 0.020041 | down | 61 | 122 |
| AT3G52420 | -0.981769263 | 0.020041 | down | 61 | 122 |
| AT5G14730 | -0.9405466 | 0.020063 | down | 71 | 138 |
| AT1G52100 | 0.655348825 | 0.020151 | up | 1028 | 661 |
| AT5G43740 | -0.70483404 | 0.020224 | down | 292 | 482 |
| AT3G50810 | -1.022033133 | 0.020355 | down | 53 | 109 |
| AT5G43540 | -1.455700451 | 0.020386 | down | 18 | 50 |
| AT5G42700 | -1.455700451 | 0.020386 | down | 18 | 50 |
| AT3G07070 | 0.953135709 | 0.020466 | up | 130 | 68 |
| AT3G30725 | -1.185302657 | 0.020538 | down | 33 | 76 |
| AT2G44360 | -0.762827441 | 0.020602 | down | 174 | 299 |
| AT4G00380 | 0.746426122 | 0.02069 | up | 328 | 198 |
| AT4G00310 | -0.970273624 | 0.020761 | down | 63 | 125 |
| AT1G21900 | -0.676104292 | 0.020801 | down | 419 | 678 |
| AT5G67510 | -0.656061052 | 0.020903 | down | 621 | 991 |
| AT2G28290 | 0.638638519 | 0.020906 | up | 6057 | 3940 |
| AT1G63530 | 0.873032822 | 0.020946 | up | 170 | 94 |
| AT3G45210 | -0.681621684 | 0.021347 | down | 378 | 614 |
| AT4G22380 | -0.713153825 | 0.021461 | down | 259 | 430 |
| AT4G16680 | -0.77710722 | 0.021593 | down | 155 | 269 |
| AT4G12545 | -0.655648155 | 0.021626 | down | 603 | 962 |
| AT1G15350 | -0.685663035 | 0.021728 | down | 353 | 575 |
| AT5G57120 | -0.638881549 | 0.021882 | down | 1092 | 1722 |
| AT3G55240 | -0.678219697 | 0.021903 | down | 390 | 632 |
| AT1G50180 | -0.844952573 | 0.021917 | down | 105 | 191 |
| AT3G19380 | 0.687841063 | 0.022018 | up | 544 | 342 |
| AT3G46210 | -0.740108508 | 0.022024 | down | 201 | 340 |
| AT1G47278 | -0.733629072 | 0.022028 | down | 212 | 357 |
| AT3G60490 | -0.949103074 | 0.022133 | down | 67 | 131 |
| AT1G51500 | 0.634433005 | 0.022142 | up | 4715 | 3076 |
| AT4G34770 | -1.156266994 | 0.022255 | down | 35 | 79 |
| AT1G58270 | 0.633522848 | 0.0223 | up | 4182 | 2730 |
| AT1G32870 | -0.688878873 | 0.022378 | down | 332 | 542 |
| AT1G52450 | -1.292109384 | 0.022468 | down | 25 | 62 |
| AT5G28630 | -1.370811554 | 0.022583 | down | 21 | 55 |
| AT1G31320 | -0.784853748 | 0.022623 | down | 145 | 253 |
| AT5G27850 | -0.637404762 | 0.02268 | down | 4601 | 7248 |
| AT5G53450 | 0.639951788 | 0.022719 | up | 1431 | 930 |
| AT5G06860 | 0.636740731 | 0.022811 | up | 1675 | 1091 |
| AT1G67550 | -0.683151816 | 0.022846 | down | 353 | 574 |
| AT2G14890 | 0.634535428 | 0.022866 | up | 5770 | 3764 |
| AT5G40690 | -0.701297165 | 0.0229 | down | 283 | 466 |
| AT1G33230 | -0.667486202 | 0.022926 | down | 447 | 719 |
| AT4G22212 | -0.752011012 | 0.023006 | down | 180 | 307 |
| AT1G34540 | 2.68119575 | 0.023306 | up | 19 | 3 |
| AT5G06630 | 0.659922685 | 0.023372 | up | 791 | 507 |
| AT3G13760 | -0.90582041 | 0.023688 | down | 78 | 148 |
| AT1G55810 | -0.654914487 | 0.023872 | down | 550 | 877 |
| AT1G10682 | -0.633202378 | 0.023986 | down | 1223 | 1921 |
| AT5G65050 | -0.66934683 | 0.024095 | down | 416 | 670 |
| AT5G61160 | -1.387761623 | 0.024108 | down | 20 | 53 |
| AT2G26110 | -0.658406364 | 0.024252 | down | 503 | 804 |
| AT1G30110 | -0.755176853 | 0.024302 | down | 172 | 294 |
| AT1G62570 | 0.641404396 | 0.024439 | up | 1186 | 770 |
| AT3G17210 | -0.637846246 | 0.024511 | down | 884 | 1393 |
| AT2G47380 | -0.650842718 | 0.024656 | down | 583 | 927 |
| AT1G12020 | -0.781899858 | 0.024719 | down | 143 | 249 |
| AT1G73965 | -2.244803669 | 0.024733 | down | 5 | 24 |
| AT4G33730 | 1.130705466 | 0.024755 | up | 80 | 37 |
| AT2G37990 | -0.643329457 | 0.024961 | down | 703 | 1112 |
| AT3G17715 | -0.929301843 | 0.024966 | down | 70 | 135 |
| AT3G60900 | 0.764129456 | 0.02508 | up | 270 | 161 |
| AT4G36060 | -1.081304936 | 0.025111 | down | 42 | 90 |
| AT1G68880 | -1.081304936 | 0.025111 | down | 42 | 90 |
| AT5G37540 | 0.703825601 | 0.025244 | up | 423 | 263 |
| AT5G03120 | -0.800439498 | 0.025288 | down | 127 | 224 |
| AT5G02490 | 0.649775374 | 0.025388 | up | 897 | 579 |
| AT5G12190 | -0.733161928 | 0.025559 | down | 199 | 335 |
| AT1G49500 | 0.631460963 | 0.025621 | up | 1675 | 1095 |
| AT5G19190 | 0.628320138 | 0.025784 | up | 2114 | 1385 |
| AT2G29460 | -1.041890255 | 0.025831 | down | 47 | 98 |
| AT5G15120 | -0.679915608 | 0.025861 | down | 339 | 550 |
| AT1G49430 | 0.632132122 | 0.02592 | up | 1561 | 1020 |
| AT4G35800 | 0.630584587 | 0.025968 | up | 6673 | 4365 |
| AT3G15760 | 0.781372954 | 0.026055 | up | 241 | 142 |
| AT3G21050 | 1.340158832 | 0.026094 | up | 55 | 22 |
| AT4G34580 | 0.704731264 | 0.026102 | up | 412 | 256 |
| AT1G11210 | 1.193317444 | 0.026166 | up | 70 | 31 |
| AT1G30760 | 0.764298538 | 0.026246 | up | 265 | 158 |
| AT2G30020 | 0.734437771 | 0.026329 | up | 322 | 196 |
| AT1G05540 | 0.745546161 | 0.026336 | up | 298 | 180 |
| AT1G55990 | 0.744055774 | 0.026338 | up | 301 | 182 |
| AT3G08990 | -1.061496455 | 0.026477 | down | 44 | 93 |
| AT4G28720 | -0.928527242 | 0.026631 | down | 69 | 133 |
| AT3G28940 | -0.625511175 | 0.026788 | down | 2020 | 3156 |
| AT3G06145 | -0.844265739 | 0.026799 | down | 99 | 180 |
| AT1G25560 | 0.629990485 | 0.026877 | up | 1603 | 1049 |
| AT4G06534 | 1.0045558 | 0.026879 | up | 105 | 53 |
| AT5G50335 | -0.720063918 | 0.026881 | down | 217 | 362 |
| AT1G17330 | -0.767264751 | 0.02695 | down | 152 | 262 |
| AT5G57280 | -0.714882532 | 0.027426 | down | 225 | 374 |
| AT3G48390 | 0.948968075 | 0.027442 | up | 122 | 64 |
| AT5G02170 | 0.901191316 | 0.027511 | up | 142 | 77 |
| AT2G17500 | 0.623951798 | 0.02757 | up | 2695 | 1771 |
| AT5G44730 | -0.697737776 | 0.027931 | down | 263 | 432 |
| AT1G53990 | -1.981769263 | 0.028295 | down | 7 | 28 |
| AT3G41762 | 1.353833769 | 0.028329 | up | 53 | 21 |
| AT3G11120 | -0.633518855 | 0.028339 | down | 802 | 1260 |
| AT4G19170 | 0.629917514 | 0.028375 | up | 8745 | 5723 |
| AT5G53650 | -0.739311219 | 0.02843 | down | 181 | 306 |
| AT2G22425 | -0.672665208 | 0.028437 | down | 350 | 565 |
| AT3G02910 | -0.638058211 | 0.028447 | down | 684 | 1078 |
| AT3G18950 | 0.726341469 | 0.028458 | up | 330 | 202 |
| AT2G43150 | 0.644837483 | 0.02851 | up | 24479 | #### |
| AT2G28630 | 0.674115024 | 0.028586 | up | 542 | 344 |
| AT3G20760 | -1.888659859 | 0.028946 | down | 8 | 30 |
| AT1G10050 | 0.666170671 | 0.028947 | up | 597 | 381 |
| AT3G13435 | -1.086105923 | 0.029145 | down | 40 | 86 |
| AT2G37120 | -0.69822226 | 0.029276 | down | 255 | 419 |
| AT3G27290 | -1.21880846 | 0.029784 | down | 28 | 66 |
| AT4G37660 | -0.743825838 | 0.03004 | down | 171 | 290 |
| AT1G76780 | -1.177320072 | 0.030051 | down | 31 | 71 |
| AT1G67140 | 0.619958867 | 0.030325 | up | 2510 | 1654 |
| AT3G53590 | 3.718670455 | 0.030362 | up | 13 | 1 |
| AT3G01050 | -0.700812949 | 0.030521 | down | 243 | 400 |
| AT1G70300 | 0.625041637 | 0.030694 | up | 1497 | 983 |
| AT1G07985 | 1.204643861 | 0.031136 | up | 66 | 29 |
| AT4G27900 | -0.664773464 | 0.031274 | down | 365 | 586 |
| AT4G38850 | -1.279449812 | 0.031292 | down | 24 | 59 |
| AT3G44010 | -0.618364532 | 0.031631 | down | 1729 | 2688 |
| AT5G23930 | -1.360280886 | 0.031917 | down | 20 | 52 |
| AT5G47060 | -0.659841168 | 0.032094 | down | 385 | 616 |
| AT5G45790 | -1.226881761 | 0.032129 | down | 27 | 64 |
| AT5G05060 | -0.68501072 | 0.032341 | down | 277 | 451 |
| AT3G22230 | -0.617467257 | 0.032348 | down | 1667 | 2590 |
| AT3G49410 | -1.010915609 | 0.032506 | down | 49 | 100 |
| AT3G14270 | 0.616163314 | 0.032989 | up | 2514 | 1661 |
| AT2G40460 | 0.623179854 | 0.033042 | up | 1381 | 908 |
| AT3G21370 | 0.8502861 | 0.033075 | up | 162 | 91 |
| AT3G47836 | -0.764412431 | 0.033106 | down | 143 | 246 |
| AT4G37040 | -0.636301611 | 0.033112 | down | 587 | 924 |
| AT4G24920 | -0.647556093 | 0.033416 | down | 457 | 725 |
| AT1G14860 | -0.838178409 | 0.033524 | down | 95 | 172 |
| AT2G02955 | -0.776578932 | 0.03353 | down | 132 | 229 |
| AT1G64110 | 0.688082135 | 0.033603 | up | 420 | 264 |
| AT4G12620 | -0.794310632 | 0.03363 | down | 119 | 209 |
| AT2G04050 | -0.884907724 | 0.033632 | down | 77 | 144 |
| AT4G01975 | -2.981769263 | 0.034006 | down | 2 | 16 |
| AT2G24550 | 0.622433122 | 0.034262 | up | 1321 | 869 |
| AT1G23200 | 0.945474395 | 0.034436 | up | 116 | 61 |
| AT1G11410 | 0.632844044 | 0.03447 | up | 934 | 610 |
| AT2G21220 | -1.531966345 | 0.034579 | down | 14 | 41 |
| AT4G35490 | -0.638548924 | 0.034603 | down | 529 | 834 |
| AT2G14210 | -0.715832049 | 0.034738 | down | 199 | 331 |
| AT1G33480 | 0.720394449 | 0.034873 | up | 314 | 193 |
| AT1G22640 | 0.635348476 | 0.035042 | up | 862 | 562 |
| AT5G09290 | -1.189877458 | 0.035045 | down | 29 | 67 |
| AT2G39330 | -0.841911599 | 0.03507 | down | 92 | 167 |
| AT1G11475 | -0.674340738 | 0.035202 | down | 297 | 480 |
| AT1G52040 | 0.680649378 | 0.035257 | up | 440 | 278 |
| AT5G27770 | -0.613449616 | 0.035263 | down | 1742 | 2699 |
| AT5G49740 | 0.614362952 | 0.035311 | up | 4603 | 3045 |
| AT1G35320 | -0.679811813 | 0.035381 | down | 278 | 451 |
| AT3G58020 | -0.752950572 | 0.035416 | down | 150 | 256 |
| AT5G02050 | -0.630099779 | 0.035527 | down | 631 | 989 |
| AT4G18930 | -0.694281813 | 0.035699 | down | 238 | 390 |
| AT3G59540 | -0.612635972 | 0.035858 | down | 1794 | 2778 |
| AT4G01380 | -0.968593874 | 0.035934 | down | 55 | 109 |
| AT2G40205 | -0.667171665 | 0.036103 | down | 319 | 513 |
| AT5G16950 | -0.996124556 | 0.036256 | down | 50 | 101 |
| AT5G12050 | -0.627644252 | 0.03629 | down | 657 | 1028 |
| AT3G47340 | 0.631395379 | 0.036396 | up | 904 | 591 |
| AT1G62420 | -1.396806762 | 0.036592 | down | 18 | 48 |
| ATMG01370 | -1.396806762 | 0.036592 | down | 18 | 48 |
| AT1G53200 | -0.720277022 | 0.036729 | down | 187 | 312 |
| AT1G74810 | 0.770303224 | 0.037242 | up | 224 | 133 |
| AT1G13810 | -0.746749233 | 0.037353 | down | 153 | 260 |
| AT5G23030 | 1.326353032 | 0.037393 | up | 52 | 21 |
| AT4G01790 | -1.244803669 | 0.037425 | down | 25 | 60 |
| AT1G54380 | -0.677375827 | 0.037431 | down | 276 | 447 |
| AT3G52670 | -1.048883459 | 0.037462 | down | 42 | 88 |
| AT1G32450 | -0.620541678 | 0.037762 | down | 6675 | #### |
| AT2G43530 | -0.707430627 | 0.037802 | down | 205 | 339 |
| AT1G08180 | -0.856238381 | 0.038006 | down | 84 | 154 |
| AT3G51860 | 0.622513848 | 0.038011 | up | 1090 | 717 |
| AT1G51820 | -1.606260128 | 0.038072 | down | 12 | 37 |
| AT4G22830 | -0.77444429 | 0.03846 | down | 127 | 220 |
| AT5G09530 | 0.610186441 | 0.038501 | up | 2068 | 1372 |
| AT2G47990 | -0.649605946 | 0.03858 | down | 389 | 618 |
| AT2G15880 | -1.071406475 | 0.0386 | down | 39 | 83 |
| AT2G36930 | -0.629097645 | 0.038604 | down | 581 | 910 |
| AT4G14270 | 0.652618455 | 0.038645 | up | 579 | 373 |
| AT3G55340 | -0.683082548 | 0.038828 | down | 254 | 413 |
| AT3G14810 | 0.626203216 | 0.038845 | up | 948 | 622 |
| AT3G57860 | -0.844265739 | 0.038956 | down | 88 | 160 |
| AT3G59840 | -0.725315006 | 0.038999 | down | 175 | 293 |
| AT3G47370 | -0.610538248 | 0.039088 | down | 1265 | 1956 |
| AT1G14790 | 0.654232671 | 0.039101 | up | 561 | 361 |
| AT5G49270 | 0.734057564 | 0.03931 | up | 271 | 165 |
| AT4G16670 | -0.720328115 | 0.039378 | down | 181 | 302 |
| AT4G04780 | -0.760254127 | 0.039516 | down | 137 | 235 |
| AT1G64400 | 0.617548531 | 0.039535 | up | 1212 | 800 |
| AT1G04290 | -0.736656765 | 0.039628 | down | 160 | 270 |
| AT5G57800 | 0.607401394 | 0.039807 | up | 3090 | 2054 |
| AT1G54000 | -0.607790937 | 0.039927 | down | 2113 | 3261 |
| AT1G13420 | 0.719889463 | 0.039939 | up | 296 | 182 |
| AT2G43870 | -0.752635264 | 0.040073 | down | 143 | 244 |
| AT5G27420 | 0.94294261 | 0.040195 | up | 112 | 59 |
| AT4G03210 | -0.620843451 | 0.040371 | down | 689 | 1073 |
| AT3G24715 | 0.697129639 | 0.04067 | up | 349 | 218 |
| AT1G14980 | -0.607586536 | 0.040986 | down | 1381 | 2131 |
| AT3G24780 | 1.276028495 | 0.041085 | up | 55 | 23 |
| AT3G60910 | -0.660408154 | 0.041107 | down | 318 | 509 |
| AT3G49100 | -0.761170123 | 0.041206 | down | 134 | 230 |
| AT1G64563 | 0.914726161 | 0.041339 | up | 121 | 65 |
| AT5G61550 | 0.771013037 | 0.041591 | up | 214 | 127 |
| AT3G16080 | -0.608159316 | 0.041667 | down | 1190 | 1837 |
| AT1G04840 | -1.441200882 | 0.041743 | down | 16 | 44 |
| AT4G24275 | -0.753353054 | 0.041756 | down | 140 | 239 |
| AT5G57290 | -0.605581504 | 0.041821 | down | 1954 | 3011 |
| AT5G09585 | -1.929301843 | 0.041888 | down | 7 | 27 |
| AT3G08860 | 0.654157694 | 0.041904 | up | 533 | 343 |
| AT3G28150 | 1.034909478 | 0.041984 | up | 87 | 43 |
| AT4G21940 | -0.895612619 | 0.042045 | down | 69 | 130 |
| AT3G57720 | -1.031237939 | 0.042167 | down | 43 | 89 |
| AT1G15550 | 0.803831106 | 0.042662 | up | 181 | 105 |
| AT3G50845 | -1.100413759 | 0.042835 | down | 35 | 76 |
| AT1G19900 | 0.852644671 | 0.042861 | up | 148 | 83 |
| AT1G02730 | 0.609034116 | 0.043 | up | 1485 | 986 |
| AT3G23150 | -0.634708557 | 0.043007 | down | 456 | 717 |
| AT3G15356 | -0.63039739 | 0.04317 | down | 495 | 776 |
| AT4G17770 | 0.605098792 | 0.043588 | up | 1894 | 1261 |
| AT1G56150 | -1.265562229 | 0.043635 | down | 23 | 56 |
| AT5G06400 | -1.265562229 | 0.043635 | down | 23 | 56 |
| AT1G78070 | 0.61208233 | 0.043954 | up | 1221 | 809 |
| AT3G02550 | -0.613852054 | 0.044131 | down | 753 | 1167 |
| AT2G43200 | -0.744374678 | 0.044184 | down | 145 | 246 |
| AT1G57860 | -0.614417852 | 0.044251 | down | 734 | 1138 |
| AT3G22100 | 1.355265724 | 0.044366 | up | 48 | 19 |
| AT5G67400 | 0.629398308 | 0.044477 | up | 750 | 491 |
| AT5G61820 | 0.601930485 | 0.04481 | up | 2855 | 1905 |
| AT1G79150 | -0.618382394 | 0.044935 | down | 631 | 981 |
| AT1G55020 | 0.601631034 | 0.045109 | up | 2769 | 1848 |
| AT2G18860 | -0.660446198 | 0.045131 | down | 298 | 477 |
| AT1G72210 | -0.99703602 | 0.045295 | down | 47 | 95 |
| AT2G35980 | 1.098401086 | 0.045627 | up | 74 | 35 |
| AT4G25315 | -0.78364212 | 0.045715 | down | 113 | 197 |
| AT5G34871 | -0.78364212 | 0.045715 | down | 113 | 197 |
| AT3G25890 | -0.634764971 | 0.045865 | down | 428 | 673 |
| AT2G23985 | -0.776740461 | 0.046178 | down | 117 | 203 |
| AT2G35736 | -0.877933452 | 0.046301 | down | 72 | 134 |
| AT3G59390 | -0.877933452 | 0.046301 | down | 72 | 134 |
| AT2G21290 | -0.649514808 | 0.047071 | down | 333 | 529 |
| AT2G27690 | -1.277225146 | 0.047123 | down | 22 | 54 |
| AT4G17490 | 1.075563912 | 0.047227 | up | 77 | 37 |
| AT5G41690 | -1.053919049 | 0.047228 | down | 39 | 82 |
| AT3G16700 | -1.053919049 | 0.047228 | down | 39 | 82 |
| AT1G18850 | -0.658613865 | 0.047569 | down | 294 | 470 |
| AT4G25760 | -0.789124185 | 0.047681 | down | 108 | 189 |
| AT4G25220 | 0.920383414 | 0.047803 | up | 114 | 61 |
| AT1G08930 | -0.608413843 | 0.047841 | down | 796 | 1229 |
| AT1G50055 | -0.822702777 | 0.047862 | down | 91 | 163 |
| AT1G30250 | 2.155734261 | 0.047898 | up | 22 | 5 |
| AT1G49700 | -1.174414341 | 0.04793 | down | 28 | 64 |
| AT3G01600 | -0.967555404 | 0.048022 | down | 51 | 101 |
| AT5G51510 | -0.674487944 | 0.048023 | down | 245 | 396 |
| AT2G47880 | -0.856238381 | 0.048066 | down | 78 | 143 |
| AT5G53870 | 0.90275352 | 0.048359 | up | 120 | 65 |
| AT5G38020 | 0.601855551 | 0.048442 | up | 1617 | 1079 |
| AT5G65060 | -0.738498112 | 0.048464 | down | 145 | 245 |
| AT1G26470 | -0.696708551 | 0.048572 | down | 198 | 325 |
| AT5G60660 | 0.696302642 | 0.04875 | up | 320 | 200 |
| AT5G27250 | -1.078630802 | 0.048815 | down | 36 | 77 |
| AT1G48100 | 0.619922876 | 0.04886 | up | 824 | 543 |
| AT3G09735 | -0.639445947 | 0.048898 | down | 374 | 590 |
| AT1G51650 | -0.599160913 | 0.048916 | down | 1393 | 2137 |
| AT5G43970 | -0.635163489 | 0.049262 | down | 398 | 626 |
| AT5G27660 | -0.677458582 | 0.049366 | down | 234 | 379 |
| AT1G35250 | 0.742865529 | 0.049462 | up | 233 | 141 |
| AT5G05900 | 2.018230737 | 0.049608 | up | 24 | 6 |
| AT1G61210 | 0.598965275 | 0.049614 | up | 1874 | 1253 |
| AT1G80820 | 0.711677868 | 0.049734 | up | 283 | 175 |
| AT1G57660 | -0.59752127 | 0.049886 | down | 1638 | 2510 |
| AT5G51810 | 1.100692897 | 0.049905 | up | 72 | 34 |
