# Supplementary material for: Depletion of Arabidopsis SC35 and SC35-like serine/arginine-rich proteins affects the transcription and splicing of a subset of genes
Source: PLoS Genet. 2017 Mar 8;13(3):e1006663. doi: 10.1371/journal.pgen.1006663 (PMC5362245; doi:10.1371/journal.pgen.1006663)
Supplement: S3 Table — Data were analyzed using the ASD software. (DOCX) [file pgen.1006663.s019.docx]

**Table S3 The genes alternatively spliced between WT and *sc35-scl* mutant in RNA-seq data**

AccID Location EN case_Skip control_Skip caseExp controlExp _PValue FDR SplicingType

AT5G36170 chr5:14237125-14237264 4 69::113 0::23 157::312 276::349 0 0 A3SS

AT2G38870 chr2:16237032-16237106 1 1098::973 1344::125 774::1207 1040::817 1.11257E-14 5.64295E-11 A5SS

AT5G64170 chr5:25675911-25676040 2 153::54 80::177 35::245 105::227 7.14466E-11 2.41585E-07 Cassette

AT4G16510 chr4:9303438-9303522 3 45::105 0::268 60::101 149::141 6.47717E-10 1.64261E-06 Cassette

AT1G08520 chr1:2698111-2698203 6 218::904 4::1012 483::613 497::479 9.868E-10 2.00202E-06 Cassette

AT3G29130 chr3:11103191-11103335 4 42::63 0::220 52::99 147::124 1.48299E-09 2.50725E-06 Cassette_multi

AT3G53830 chr3:19943017-19943077 9-10 174::105 95::449 70::245 245::325 6.55207E-09 9.49489E-06 Cassette

AT5G13800 chr5:4454310-4454360 2 121::89 20::162 47::209 80::182 9.1371E-09 1.15858E-05 Cassette

AT1G02840 chr1:628777-628856 10-11 989::454 1591::387 253::796 236::1037 4.47711E-08 5.0462E-05 Cassette

AT2G43010 chr2:17888114-17888179 5 83::832 1::764 422::906 426::810 1.01744E-07 0.000103209 Cassette

AT4G34265 chr4:16402958-16403022 3 1107::649 1033::1069 296::805 537::906 3.11561E-07 0.000287316 Cassette

AT5G36170 chr5:14237341-14237561 3 101::212 0::436 146::301 261::272 1.98401E-06 0.001677154 Cassette

AT1G55310 chr1:20631561-20631722 4 140::1 185::86 16::224 153::480 3.24411E-06 0.002531404 Cassette

AT2G04378 chr2:1525785-1526156 1 34::173 58::11 402::834 622::813 6.83656E-06 0.004399468 A5SS

AT1G07820 chr1:2421783-2421903 1 296::801 554::558 501::1429 338::1670 6.85601E-06 0.004399468 IR

AT4G33770 chr4:16195006-16195038 3 28::262 2::566 134::166 290::331 6.93922E-06 0.004399468 Cassette

AT3G51420 chr3:19084952-19085017 3 412::103 614::42 509::734 683::760 9.07611E-06 0.005415768 A3SS

AT4G35770 chr4:16945828-16945909 4 541::418 505::102 270::298 82::213 2.81217E-05 0.015848115 IR

AT3G10420 chr3:3241124-3241244 6 100::884 3::1148 755::775 951::803 3.43134E-05 0.01831977 A5SS

AT3G22121 chr3:7795887-7795966 1 155::9094 0::10314 4556::7801 5060::10317 4.76888E-05 0.02339277 IR

AT2G04378 chr2:1525785-1526037 1 173::39 11::69 381::508 621::575 5.02598E-05 0.02339277 Cassette

AT5G56140 chr5:22726610-22726755 3 77::778 152::564 451::534 315::514 5.07335E-05 0.02339277 Cassette

AT1G04830 chr1:1360225-1360353 6 48::52 1::191 33::115 97::140 6.49599E-05 0.02865013 Cassette

AT5G62575 chr5:25118054-25118156 3 52::768 3::1051 459::286 637::309 7.14003E-05 0.029713778 Cassette

AT5G11170 chr5:3554939-3555538 6 1101::26 1046::90 160::1007 297::1023 7.37094E-05 0.029713778 A5SS

AT5G18255 chr5:6036230-6036292 2 36::67 5::139 117::168 161::218 7.62233E-05 0.029713778 Cassette

AT1G51110 chr1:18935743-18935796 2 45::319 1::539 173::191 309::207 8.05996E-05 0.029713778 Cassette

AT4G13500 chr4:7857578-7857647 2 61::2293 1::2476 997::651 1105::677 8.20175E-05 0.029713778 Cassette

AT5G14550 chr5:4691318-4691359 11 110::97 39::270 2242::987 1792::850 0.000105101 0.035885585 Cassette

AT4G08390 chr4:5317179-5317456 11 43::791 12::1350 387::649 745::799 0.000106129 0.035885585 AltEnd

AT3G19720 chr3:6851209-6851318 15 174::496 93::894 285::425 500::548 0.000151133 0.049348638 IR

AT5G58800 chr5:23746870-23746943 2 49::524 0::503 278::416 271::410 0.000158705 0.049348638 Cassette

AT2G26980 chr2:11516427-11516480 10-11 186::48 207::203 42::226 112::311 0.000169914 0.049348638 Cassette

AT2G39730 chr2:16571066-16571191 6 52496::58491 62832::66411 9502::74159 11236::79332 0.000170625 0.049348638 IR

AT1G07110 chr1:2179108-2179137 19 308::33 474::1 334::401 506::570 0.000172109 0.049348638 A5SS

AT1G73480 chr1:27631319-27631365 3-4 190::28 76::51 28::232 37::119 0.000175133 0.049348638 Cassette

AT1G19350 chr1:6688775-6688834 3 768::499 817::187 1989::2966 1851::2821 0.000204563 0.056083474 A3SS

AT5G66570 chr5:26569323-26569402 2 25::88667 11::74934 48576::49600 39914::44366 0.000224112 0.059826125 IR

AT5G53860 chr5:21866783-21866842 5 121::983 31::1098 457::627 487::617 0.000239356 0.06225711 Cassette

AT1G11840 chr1:3996016-3996041 2 146::1561 251::1198 400::2518 457::2083 0.000292108 0.074078498 A3SS

AT3G05160 chr3:1455119-1455183 8 0::136 28::70 70::83 45::89 0.000352328 0.085067994 Cassette

AT3G49645 chr3:18404366-18404443 2 23::7 0::35 5::34 26::30 0.000354871 0.085067994 Cassette

AT1G34470 chr1:12605070-12605093 2 73::3 36::31 8::83 35::73 0.000363475 0.085067994 A5SS

AT5G37055 chr5:14642105-14642210 2 35::101 0::200 83::98 118::118 0.00037185 0.085067994 Cassette

AT2G20890 chr2:8988195-8988348 2 171::4121 10::3985 4407::5088 4253::4268 0.000377372 0.085067994 A5SS

AT1G55130 chr1:20573329-20573418 8 44::557 4::681 270::327 330::320 0.000520427 0.114765367 Cassette

AT5G60390 chr5:24290464-24290514 3 0::40033 0::47341 21811::15555 26011::16417 0.000554925 0.119204132 IR

AT3G18500 chr3:6353271-6353336 3 20::39 9::163 30::64 99::92 0.000564057 0.119204132 Cassette

AT1G69460 chr1:26112271-26112347 3 46::863 5::1183 463::524 631::666 0.000591941 0.122543966 Cassette

AT3G47460 chr3:17493860-17494077 21 3::169140 3::166707 85039::88393 84330::83813 0.000626409 0.126762863 IR

AT1G18735 chr1:6460896-6461073 3 135::1374 42::1904 762::860 952::1074 0.000647567 0.126762863 Cassette

AT4G08280 chr4:5230964-5231053 2 53::1044 5::1276 567::575 667::589 0.00064981 0.126762863 Cassette

AT2G18440 chr2:7995892-7995974 3 17::32 0::113 479::978 744::1285 0.000778898 0.145274127 Cassette

AT2G42500 chr2:17698917-17699049 8 756::7 618::54 9::810 37::778 0.000782545 0.145274127 Cassette

AT1G69530 chr1:26142917-26143008 4 392::12222 769::15089 6535::5925 7958::7243 0.000787665 0.145274127 IR

AT1G50000 chr1:18515597-18515658 7 42::3 27::49 19::84 85::125 0.000805008 0.14582152 A3SS

AT1G64430 chr1:23935734-23935821 9 19::343 0::737 195::232 366::433 0.000873571 0.155464976 Cassette

AT5G18260 chr5:6036202-6036292 4 36::52 5::97 100::159 137::199 0.000944609 0.165208934 AltEnd

AT3G49290 chr3:18273343-18273389 2 19::32 55::2 20::68 60::73 0.000978132 0.168172456 A3SS

AT1G19120 chr1:6608722-6608768 3 50::309 12::390 280::319 354::377 0.00109198 0.18461749 Cassette

AT1G37130 chr1:14161410-14161474 3 0::20252 28::25573 10080::6062 12892::6541 0.001134259 0.188271653 IR

AT1G23360 chr1:8296242-8296254 4 40::86 145::83 64::149 137::181 0.001150714 0.188271653 A5SS

AT5G14550 chr5:4691190-4691318 11 39::3714 140::2741 1966::2008 1452::1544 0.00120521 0.19102783 IR

AT2G28550 chr2:12226969-12227013 6 559::25 588::119 22::598 127::798 0.001205223 0.19102783 Cassette

AT3G52340 chr3:19407205-19407274 2 125::34 73::77 38::162 61::151 0.001304402 0.200758576 Cassette

AT4G22670 chr4:11920058-11920083 8 224::3000 0::3242 1479::1974 1658::2237 0.001317085 0.200758576 IR

AT3G13470 chr3:4392611-4392801 15 76::2011 29::2743 946::1105 1276::957 0.001325988 0.200758576 AltEnd

AT4G00710 chr4:291743-291848 6 22::236 0::469 171::210 264::295 0.001456729 0.215983597 Cassette

AT2G04690 chr2:1645510-1645671 3 137::280 55::438 314::437 405::457 0.001469131 0.215983597 A5SS

AT4G09760 chr4:6149586-6149636 4-5 188::37 127::82 30::244 58::224 0.001547258 0.224219835 Cassette

AT3G63340 chr3:23393582-23393669 13 16::88 0::259 66::75 158::139 0.001752935 0.250447492 Cassette

AT1G78790 chr1:29623760-29623812 8 32::15 154::0 40::21 26::68 0.001788965 0.252045336 A3SS

AT4G38330 chr4:17951752-17951818 2 65::9 15::38 8::74 29::49 0.001896191 0.263492622 Cassette

AT5G20510 chr5:6939178-6939251 6 84::2658 18::3209 1349::1499 1664::1729 0.002043376 0.280108194 Cassette

AT1G26670 chr1:9216860-9216918 2 33::1401 0::1552 645::631 723::672 0.002107024 0.284981986 Cassette

AT1G52220 chr1:19454187-19454273 2 378::5928 171::7336 3082::2631 3756::2823 0.002305627 0.307740594 Cassette

AT5G58100 chr5:23510795-23510865 14 24::113 0::190 62::78 100::87 0.00248369 0.32720194 Cassette

AT5G54270 chr5:22039153-22039347 3 0::138174 17::154640 78870::64977 89948::63965 0.002668336 0.347020505 IR

AT3G52050 chr3:19307082-19307135 7 12::251 36::155 115::157 86::142 0.002749902 0.351334581 Cassette

AT5G23020 chr5:7721288-7721362 9 98::785 83::1521 360::310 702::554 0.002770777 0.351334581 Cassette

AT5G27390 chr5:9675000-9675023 5 378::29 486::2 400::446 492::523 0.003090007 0.386975653 A5SS

AT3G24520 chr3:8941712-8941767 2 23::163 65::78 37::173 78::135 0.003141029 0.388568264 A3SS

AT4G02725 chr4:1206994-1207207 2 165::160 442::94 74::215 60::334 0.00341697 0.415813015 IR

AT3G05160 chr3:1454713-1454793 10 28::157 3::226 78::87 118::95 0.003443247 0.415813015 Cassette

AT1G76280 chr1:28620268-28620363 8 16::8 8::88 9::33 50::45 0.003578758 0.427093169 Cassette

AT1G28395 chr1:9970113-9970153 3 270::435 284::743 221::234 357::313 0.004033354 0.470645392 Cassette

AT4G34710 chr4:16562465-16562563 1 2::3079 2::1612 1845::3521 947::2327 0.004036489 0.470645392 IR

AT3G28130 chr3:10468077-10468238 5 39::151 10::262 96::155 157::191 0.00421113 0.485428452 Cassette

AT5G51960 chr5:21110703-21110913 2 37::354 0::528 212::159 309::198 0.004277046 0.487487077 Cassette

AT1G14710 chr1:5063823-5063930 3 32::298 1::349 135::154 177::148 0.004607589 0.519326503 Cassette

AT4G05320 chr4:2719241-2719291 3 0::288 280::61 16880::16758 17351::19359 0.004671392 0.520731857 A3SS

AT2G45070 chr2:18587471-18587493 3 168::394 221::204 1118::2531 988::2873 0.005016727 0.553148717 A3SS

AT5G05435 chr5:1606960-1607060 2 12::33 3::123 28::33 76::56 0.00514303 0.560977379 Cassette

AT3G53890 chr3:19956062-19956080 2 121::2585 48::2844 2343::3975 2577::4343 0.005299678 0.571914137 A3SS

AT1G12370 chr1:4207435-4207525 4 42::35 13::81 76::121 106::104 0.005747756 0.61373935 A3SS

AT5G05270 chr5:1563492-1563543 1 308::11 172::45 30::99 32::51 0.005862305 0.619450195 A5SS

AT2G45140 chr2:18611959-18612009 6 41::958 7::1023 420::533 444::536 0.006019917 0.624939007 Cassette

AT4G31810 chr4:15387600-15387641 13 18::373 0::640 169::157 299::242 0.006037463 0.624939007 Cassette

AT1G05230 chr1:1518181-1518226 2 28::6 2::22 10::44 16::41 0.006667184 0.676042484 Cassette_multi

AT4G08460 chr4:5378698-5378832 2 531::291 430::403 147::624 209::652 0.006688046 0.676042484 Cassette

AT3G66654 chr3:2088183-2088505 1 157::1 120::20 11::39 29::28 0.006731101 0.676042484 A5SS

AT1G05900 chr1:1787786-1787910 5 35::42 17::122 41::88 90::110 0.00680455 0.676719195 Cassette

AT4G18440 chr4:10186692-10186760 9 28::549 0::887 265::237 418::398 0.007284304 0.717397872 Cassette

AT4G05320 chr4:2719062-2719291 2 2201::28658 2115::27384 15754::13663 15290::14502 0.007424863 0.719112797 IR

AT2G46915 chr2:19275238-19275378 8 88::104 17::212 61::136 103::129 0.007443498 0.719112797 Cassette

AT5G26110 chr5:9119105-9119218 3 35::253 1::295 129::181 150::189 0.007591001 0.725271728 Cassette_multi

AT3G26570 chr3:9756336-9756414 1 109::2948 72::2203 1695::2456 1230::2195 0.007650244 0.725271728 IR

AT4G31720 chr4:15355612-15355683 2 639::129 869::47 599::796 852::992 0.007854806 0.737769904 A5SS

AT5G04430 chr5:1251501-1251564 6 706::434 871::332 557::1237 584::1418 0.008261247 0.768826489 A3SS

AT5G52030 chr5:21128784-21128805 2 5::87 34::54 21::142 50::128 0.008462684 0.777481265 A3SS

AT3G61420 chr3:22729093-22729152 12 16::17 61::4 47::73 90::104 0.008507534 0.777481265 A5SS

AT4G38520 chr4:18017585-18017682 2 61::338 0::430 444::840 546::992 0.008666368 0.784925364 A3SS

AT2G43235 chr2:17969932-17969973 1-2 137::10 133::47 10::152 33::170 0.009121184 0.818807891 Cassette

AT3G56160 chr3:20839375-20839488 8 75::241 16::353 170::239 226::235 0.010203227 0.907908226 Cassette

AT5G55580 chr5:22515958-22516030 2 22::81 0::140 48::63 78::71 0.010514674 0.92748566 Cassette

AT1G02840 chr1:628935-629288 11 220::962 182::1583 226::1015 269::1525 0.010867428 0.950337813 A3SS

AT2G21330 chr2:9128694-9128777 5 39925::811 38129::1543 3018::28648 3270::27871 0.01126819 0.964521884 IR

AT1G73490 chr1:27634522-27634598 3 20::363 0::517 191::297 282::371 0.011281904 0.964521884 Cassette

AT3G56210 chr3:20853100-20853418 7 512::183 261::233 15::210 20::194 0.01137499 0.964521884 Cassette

AT2G19480 chr2:8440872-8440889 11 11::3426 88::4071 1421::940 1693::1167 0.011409959 0.964521884 Cassette

AT4G13495 chr4:7843248-7843376 2 369::782 418::1561 407::879 828::1538 0.011522566 0.965990965 IR

AT2G39760 chr2:16585358-16585406 3-4 229::49 376::22 20::198 21::296 0.011756608 0.973172373 Cassette

AT3G26510 chr3:9711134-9711338 4 6::17 0::10 46::1229 73::528 0.011800099 0.973172373 AltEnd

AT1G14710 chr1:5064811-5064972 1 33::113 28::33 59::156 14::160 0.01330653 1 IR

AT4G33770 chr4:16194902-16194962 3-4 28::11 2::26 16::166 64::331 0.014079739 1 Cassette

AT1G54390 chr1:20306153-20306216 4 19::112 5::239 58::91 124::157 0.014096818 1 Cassette

AT1G31160 chr1:11123479-11123551 4 31::476 1::576 452::447 567::572 0.014183933 1 A3SS

AT3G17300 chr3:5907796-5907804 3 52::384 21::484 258::418 318::479 0.014316415 1 A5SS

AT3G08940 chr3:2718663-2718702 1 189::58774 154::60280 30737::48563 31742::39338 0.014446604 1 IR

AT4G27040 chr4:13574622-13574673 5 120::50 184::22 100::226 53::262 0.014543882 1 A3SS

AT2G05100 chr2:1823672-1823715 2 79::200493 525::194166 79713::47451 82577::44282 0.014559869 1 IR

AT5G55896 chr5:22632678-22632769 1-2 43::13 28::42 6::43 32::43 0.015431473 1 Cassette

AT3G01500 chr3:195406-195463 8 28061::512 22260::12 29587::27922 23153::20732 0.016393892 1 A5SS

AT3G15770 chr3:5340486-5340641 2 40::88 82::0 155::238 243::284 0.017038827 1 A3SS

AT5G21105 chr5:7177256-7177640 5 0::756 0::1210 464::498 790::575 0.017113948 1 IR

AT5G01650 chr5:243034-243123 3 1923::56 1965::129 53::1401 109::1457 0.017135203 1 Cassette

AT5G41670 chr5:16665543-16665620 1 119::357 175::533 208::397 341::415 0.017633238 1 IR

AT4G19095 chr4:10453634-10453714 before first 118::26 167::3 38::44 37::62 0.017959614 1 AltStart

AT3G26900 chr3:9912290-9912382 10 25::869 0::987 409::411 496::366 0.018280058 1 Cassette

AT3G09600 chr3:2946925-2946982 2 20::544 0::779 297::478 434::613 0.018296146 1 Cassette

AT1G73650 chr1:27688457-27688561 9 106::573 261::534 323::208 296::296 0.018374062 1 IR

AT1G07119 chr1:2186873-2186913 6 35::42 10::89 25::104 43::99 0.018539812 1 Cassette

AT2G30860 chr2:13139870-13140040 3 0::41343 0::46102 22949::14719 25234::14973 0.019038567 1 IR

AT5G02500 chr5:554285-554388 2 0::60381 777::94508 32202::19564 50703::27742 0.019120385 1 IR

AT3G55460 chr3:20562312-20562392 3-4 241::13 1075::20 11::181 26::821 0.019302886 1 AltStart

AT2G39780 chr2:16592241-16592244 5 6::27 31::12 9::605 20::509 0.019449219 1 Cassette

AT1G55690 chr1:20812173-20812296 2 20::30 0::71 22::34 41::37 0.019850581 1 Cassette

AT3G17950 chr3:6146603-6146629 2 123::22 58::38 159::210 94::241 0.020128688 1 A3SS

AT3G13480 chr3:4392611-4392691 3 76::2011 29::2743 1559::814 2117::857 0.021351882 1 AltStart

AT3G01690 chr3:257457-257532 3 686::66 911::3 764::1055 925::1200 0.021418093 1 A3SS

AT3G26020 chr3:9514038-9514192 2 23::6 0::21 10::44 16::48 0.022949085 1 Cassette

AT3G28950 chr3:10976917-10976958 3 116::25 203::7 116::154 188::197 0.023868112 1 A5SS

AT5G26180 chr5:9152754-9152816 14 16::25 57::12 26::69 62::79 0.024048724 1 A3SS

AT5G09225 chr5:2870368-2870407 3 153::216 135::389 222::424 408::625 0.024499259 1 A3SS

AT4G03415 chr4:1505834-1505937 2 117::14 94::43 10::214 37::205 0.024585075 1 Cassette

AT3G18890 chr3:6512178-6512183 5 57::363 96::261 259::347 241::326 0.025172864 1 A3SS

AT1G29040 chr1:10134536-10134581 2 38::356 7::412 332::353 355::341 0.025568854 1 A5SS

AT3G10230 chr3:3165768-3165983 1 1::1016 0::797 573::722 415::755 0.025592581 1 IR

AT1G79600 chr1:29951735-29951763 3 8::628 12::380 693::754 476::736 0.026037221 1 A3SS

AT4G24800 chr4:12782238-12782310 3 200::17 126::45 15::191 32::142 0.026172337 1 Cassette

AT2G06025 chr2:2350897-2350933 6 7::499 34::429 219::242 218::254 0.026690659 1 Cassette

AT5G53850 chr5:21863802-21864089 2 9::263 36::140 15::299 33::188 0.02671608 1 A5SS

AT3G17609 chr3:6024108-6024153 2 209::212 308::162 279::566 438::777 0.027007977 1 A3SS

AT1G36160 chr1:13538827-13538916 23 32::70 0::99 65::94 90::81 0.027176485 1 A5SS

AT1G70490 chr1:26565318-26565416 2 3034::6 2520::43 11::2563 36::2146 0.027214842 1 Cassette

AT5G17300 chr5:5691551-5691612 4 129::1123 18::500 505::884 216::326 0.027496264 1 Cassette

AT5G53048 chr5:21511543-21511670 5 145::603 174::1640 315::377 842::739 0.028030081 1 IR

AT5G53048 chr5:21510781-21510838 3 101::188 164::181 267::230 552::303 0.028098263 1 A3SS

AT2G05310 chr2:1933778-1933848 2 111::2300 21::2413 502::2801 520::2956 0.02846722 1 A5SS

AT1G49590 chr1:18355747-18355796 5 172::61 113::96 50::217 68::196 0.028799525 1 Cassette

AT4G26650 chr4:13445416-13445563 2 83::248 10::247 103::175 96::126 0.028873531 1 Cassette

AT1G23310 chr1:8270149-8270233 7 21::31646 61::26866 13805::13300 11709::10988 0.028938225 1 IR

AT3G61420 chr3:22728909-22729044 13 15::17 55::4 43::69 35::104 0.029095919 1 A3SS

AT1G07010 chr1:2154047-2154052 6 144::586 234::527 548::946 586::947 0.029105096 1 A3SS

AT1G36390 chr1:13702788-13703103 2 251::37 406::3 162::221 219::193 0.030127816 1 A5SS

AT1G02305 chr1:456757-456761 5 377::2 167::11 3::566 8::358 0.030827901 1 Cassette

AT3G16230 chr3:5502603-5502867 12 30::222 0::321 118::138 159::163 0.030853385 1 Cassette_multi

AT1G19396 chr1:6710468-6710548 2-3 32::14 77::2 39::47 27::85 0.030864672 1 AltEnd

AT1G01790 chr1:286925-286997 5 9::240 37::200 22::305 56::281 0.03206637 1 A3SS

AT3G45050 chr3:16477128-16477300 4 68::252 193::235 118::185 106::278 0.034325152 1 IR

AT2G43160 chr2:17948798-17948827 2 176::53 87::72 158::244 85::165 0.034635099 1 A3SS

AT1G69252 chr1:26037066-26037276 2 144::32 6::41 1997::2334 1562::1856 0.034799916 1 A5SS

AT5G38480 chr5:15410069-15410149 1-2 914::0 769::19 5::1828 32::1755 0.034969834 1 AltStart

AT1G80940 chr1:30411603-30411680 2 163::201 40::395 330::482 471::541 0.035361636 1 A5SS

AT4G14385 chr4:8287517-8287544 4 44::393 22::583 224::259 326::333 0.035382557 1 Cassette

AT1G19350 chr1:6688644-6688738 1 1246::34 923::99 231::863 236::745 0.035497787 1 A5SS

AT1G20810 chr1:7233178-7233205 5 333::17 433::71 76::458 142::543 0.035869973 1 A5SS

AT5G01600 chr5:227953-229197 2 35::3481 10::4847 1784::1831 2937::2308 0.036569998 1 AltEnd

AT2G35500 chr2:14914732-14914737 3 6::299 44::282 88::289 103::332 0.036798799 1 A3SS

AT4G00420 chr4:181903-181952 5 36::119 16::207 137::189 235::245 0.037522449 1 A3SS

AT4G13940 chr4:8056139-8056707 3 0::30478 0::37738 21143::7139 26808::8366 0.03756324 1 IR

AT2G21240 chr2:9102501-9102591 2 58::152 14::158 164::259 227::272 0.039645818 1 A3SS

AT1G47530 chr1:17453099-17453116 4 18::878 42::830 71::1021 173::1093 0.039704485 1 A3SS

AT2G02390 chr2:629518-629539 4 69::201 24::211 95::285 55::240 0.039930641 1 A3SS

AT4G20850 chr4:11164063-11164180 22 21::477 0::629 250::222 298::287 0.040389582 1 Cassette

AT1G66980 chr1:24999394-24999580 7 18::27 0::59 24::49 61::75 0.040701703 1 Cassette

AT3G46130 chr3:16945550-16945621 1 18::56 13::165 26::44 92::78 0.041438068 1 IR

AT1G47490 chr1:17426096-17426210 3 382::31 425::137 37::329 83::469 0.043047906 1 IR

AT3G13920 chr3:4592664-4592705 5 0::13639 0::16545 7071::5016 8506::5455 0.043056133 1 IR

AT4G04880 chr4:2466170-2466382 5 28::99 0::127 54::69 54::64 0.043291279 1 Cassette

AT3G61420 chr3:22727539-22727618 19 83::24 139::3 30::107 30::192 0.043904861 1 A3SS

AT5G12240 chr5:3959632-3959672 4 30::1041 71::1082 451::516 472::554 0.04395902 1 Cassette

AT1G76970 chr1:28924571-28924900 2 33::23 1::44 27::32 26::22 0.04401697 1 Cassette_multi

AT3G28080 chr3:10454444-10454605 5 41::81 22::151 40::119 90::140 0.044108378 1 Cassette

AT5G53850 chr5:21863653-21863700 3 85::341 93::171 176::298 92::205 0.044295179 1 Cassette

AT3G25680 chr3:9351344-9351349 9 5::47 37::33 28::73 40::92 0.045539562 1 A3SS

AT3G49430 chr3:18332248-18332492 2 789::75 701::156 37::420 63::441 0.045549453 1 Cassette

AT3G54790 chr3:20284138-20284375 2 35::6 0::18 16::58 30::56 0.046249593 1 A3SS

AT1G33230 chr1:12047281-12047375 3 15::116 0::217 73::99 113::164 0.046832921 1 Cassette

AT2G34410 chr2:14523214-14525352 17 85::72 79::83 41083::7799 33843::8990 0.047824864 1 AltEnd

AT4G35785 chr4:16953854-16953890 7 307::67 336::179 63::390 174::514 0.048650988 1 Cassette

AT1G72510 chr1:27303520-27303739 1 7::460 43::325 151::324 94::269 0.048722164 1 IR

AT1G28330 chr1:9934288-9934451 3 307::193 464::91 126::405 60::376 0.050149103 1 IR

AT4G29020 chr4:14305327-14305407 1-2 57::0 52::179 5546::2516 4800::1933 0.050998542 1 AltEnd

AT1G25098 chr1:8813245-8813920 2 3014::1081 3418::1904 3::6 3::5 0.052039519 1 IR

AT3G01500 chr3:194891-195179 9 3446::15436 3836::12313 4902::14408 3987::11967 0.052398907 1 IR

AT2G46270 chr2:19002161-19002230 9 65::169 91::90 178::242 87::197 0.052601615 1 A5SS

AT5G06980 chr5:2168216-2168242 3 446::191 697::146 527::1008 815::1325 0.054865755 1 A3SS

AT4G05530 chr4:2817925-2817960 6 465::120 729::18 423::297 680::368 0.057485143 1 A3SS

AT1G72180 chr1:27169330-27169468 5 13::25 2::67 16::36 36::45 0.057679986 1 Cassette

AT5G05610 chr5:1678019-1678072 3-4 324::39 387::18 22::454 14::455 0.058097193 1 Cassette

AT3G16770 chr3:5706305-5706392 2 5::21653 11::33442 11190::6337 17279::10557 0.05834084 1 IR

AT5G53048 chr5:21510944-21511045 4 119::344 131::855 192::335 454::686 0.058548135 1 Cassette

AT4G24100 chr4:12517327-12517395 10 16::32 0::65 20::46 38::52 0.058943432 1 Cassette

AT2G22720 chr2:9658140-9658147 3 85::109 46::139 142::194 121::181 0.059120757 1 A3SS

AT5G45310 chr5:18360061-18360240 3 20::9 0::28 12::32 15::22 0.059418522 1 Cassette

AT3G55330 chr3:20514854-20514863 3 85::906 124::724 668::1223 609::1206 0.059519346 1 A3SS

AT4G11830 chr4:7118727-7119481 3 32::97 15::140 63::134 140::134 0.059532667 1 Cassette

AT2G24270 chr2:10329618-10329722 1 164::5666 144::3033 3065::2828 1640::1720 0.059664192 1 IR

AT3G53970 chr3:19986758-19986880 7 425::27 435::92 51::497 78::555 0.059695767 1 IR

AT1G22750 chr1:8052610-8052663 9 119::470 259::467 289::180 572::245 0.060108781 1 A3SS

AT5G25560 chr5:8898022-8898983 1 67::45 10::172 99::94 178::99 0.06015091 1 A5SS

AT1G72640 chr1:27347489-27347498 4 131::48 147::133 71::241 129::314 0.062091378 1 A3SS

AT3G13040 chr3:4175031-4175055 2 418::48 419::9 377::207 369::164 0.063020823 1 A5SS

AT1G32610 chr1:11796966-11797053 1 0::47 0::16 27::17 13::26 0.063610007 1 IR

AT5G52570 chr5:21336372-21336431 2 54::1176 8::900 521::503 397::380 0.063642867 1 Cassette

AT2G43680 chr2:18108630-18108719 2 375::12 380::43 10::311 24::302 0.063975936 1 Cassette

AT3G29100 chr3:11076811-11077022 3 21::7 3::17 5::30 22::25 0.064251556 1 Cassette

AT5G25560 chr5:8899094-8899426 2 45::68 172::12 144::217 243::298 0.064498061 1 A3SS

AT3G15450 chr3:5213710-5213808 2 1604::526 705::105 382::1709 102::619 0.064988903 1 IR

AT5G53900 chr5:21882580-21882734 3 42::226 0::371 147::245 209::239 0.065194587 1 Cassette

AT2G26500 chr2:11270595-11270692 2 15::32331 14::35863 16309::12090 18374::12778 0.067120029 1 IR

AT1G61040 chr1:22483354-22483597 1 73::4 70::13 14::50 29::38 0.067604768 1 A5SS

AT3G48200 chr3:17836122-17836285 5 40::154 0::202 187::279 228::228 0.068746746 1 A3SS

AT3G15000 chr3:5051717-5051748 4 234::3766 0::3971 1962::1616 2073::1915 0.070522624 1 IR

AT1G01060 chr1:35212-35282 9 89::3113 11::2955 1612::1924 1555::1947 0.07066824 1 IR

AT1G76930 chr1:28895901-28895950 2 429::4209 532::4972 2191::2414 2594::3366 0.070749233 1 IR

AT4G33530 chr4:16128238-16128280 7 8::160 31::123 52::190 63::155 0.0709586 1 A5SS

AT5G67385 chr5:26884654-26884809 1 41::291 3::231 304::222 242::151 0.072553977 1 Cassette

AT5G55530 chr5:22493324-22493524 2 16::145 30::65 53::242 28::237 0.073347333 1 AltStart

AT2G20610 chr2:8878325-8878406 6 1178::74 1678::223 134::685 277::987 0.074592542 1 IR

AT1G02780 chr1:607970-608060 5 15::8527 0::9125 3928::3703 4057::4275 0.074786566 1 IR

AT5G14590 chr5:4704739-4704807 10 24::356 1::420 179::241 203::271 0.075099121 1 Cassette

AT3G53460 chr3:19820264-19820289 3 113::9481 58::9535 5055::4869 5062::5408 0.076274244 1 IR

AT1G24360 chr1:8642390-8642422 7 27::2036 0::2049 889::1350 873::1263 0.07785144 1 Cassette

AT5G65050 chr5:25984681-25984738 2 24::240 11::426 123::90 222::139 0.078139266 1 Cassette

AT4G23710 chr4:12350551-12350652 2 25::1267 2::1325 697::624 778::613 0.078328319 1 Cassette

AT5G48220 chr5:19550132-19550226 2 153::31 129::72 30::178 49::166 0.078883064 1 Cassette_multi

AT5G45350 chr5:18383145-18383190 2 15::36 39::19 36::84 45::57 0.080096514 1 A5SS

AT4G21720 chr4:11543744-11543796 3 0::65 20::53 7::63 30::73 0.080598476 1 A3SS

AT5G01720 chr5:269137-269351 2 6::108 45::105 11::103 42::134 0.081523309 1 A5SS

AT2G29340 chr2:12598016-12598115 4 1046::221 1094::113 156::801 125::828 0.081725049 1 IR

AT5G52110 chr5:21171016-21171027 8 121::177 199::137 170::327 162::394 0.082291828 1 A3SS

AT1G77770 chr1:29246390-29246489 2 85::123 84::215 77::148 143::145 0.082473534 1 IR

AT4G27040 chr4:13574622-13574742 4 120::116 184::62 62::181 40::215 0.083421959 1 IR

AT3G56940 chr3:21076631-21076709 1 0::45357 0::26413 25782::17219 15055::10759 0.083715911 1 IR

AT4G13495 chr4:7843248-7843302 2 373::82 425::190 421::624 905::1075 0.084582607 1 A5SS

AT2G01270 chr2:139716-139770 1-2 171::39 204::11 40::214 31::203 0.084717295 1 Cassette

AT4G36648 chr4:17281099-17281897 2 234::277 106::717 168::247 358::387 0.084837654 1 IR

AT5G64240 chr5:25698602-25698743 6 29::224 2::275 247::318 249::290 0.085123519 1 A5SS

AT4G34570 chr4:16514106-16514147 2 8::144 18::66 68::46 32::34 0.085511506 1 Cassette

AT2G37340 chr2:15671420-15671561 3 1744::55 2085::187 115::710 193::915 0.086067916 1 IR

AT4G31990 chr4:15473547-15473672 1 310::54 327::14 50::212 20::207 0.086776309 1 IR

AT4G21910 chr4:11625742-11625822 8 196::141 311::74 86::96 58::123 0.087299848 1 IR

AT2G22670 chr2:9636808-9636897 2 1564::5 1249::28 10::1905 23::1612 0.088071086 1 Cassette

AT1G44575 chr1:16872480-16872947 3 35263::78 32863::169 190::19961 227::18206 0.088084066 1 IR

AT2G24420 chr2:10383059-10383163 14 48::461 5::493 233::145 222::159 0.088786106 1 IR

AT1G22630 chr1:8003703-8003717 2 87::1078 58::1428 164::1379 165::1651 0.08947787 1 A3SS

AT5G46690 chr5:18946315-18946504 2 87::62 97::8 124::215 147::222 0.089726364 1 A3SS

AT4G28025 chr4:13936452-13936536 2 27::2094 2::2560 1105::1112 1324::1335 0.089796465 1 Cassette

AT5G53048 chr5:21509922-21510421 1 234::163 421::151 99::212 149::403 0.090562875 1 A5SS

AT4G09012 chr4:5781045-5781106 3 14::8 40::3 12::28 18::54 0.091128768 1 Cassette

AT1G26100 chr1:9023666-9023682 3 12::133 31::84 124::172 87::126 0.092264401 1 A3SS

AT2G20180 chr2:8704530-8704608 8 22::280 1::298 188::173 205::195 0.092429274 1 Cassette

AT5G19000 chr5:6343644-6343725 3 153::102 245::78 66::269 59::357 0.093071223 1 Cassette

AT1G58280 chr1:21621261-21621337 6 365::77 424::194 81::420 154::526 0.093396806 1 IR

AT4G26790 chr4:13488333-13488444 3 91::8 33::28 92::109 44::82 0.094870363 1 A3SS

AT1G76630 chr1:28762015-28762029 11 58::2 27::19 46::55 30::41 0.095075129 1 A3SS

AT3G54890 chr3:20339848-20339940 3 79686::1152 91104::1418 4052::56678 4664::59944 0.09520889 1 IR

AT1G10890 chr1:3628921-3628972 2-3 69::10 39::25 58::144 110::167 0.09559892 1 Cassette

AT4G30200 chr4:14787915-14787951 5 143::179 156::121 221::370 165::439 0.097591415 1 A3SS

AT4G02890 chr4:1279312-1279388 3 0::598 39::409 4605::4165 4149::4501 0.09833967 1 Cassette

AT3G06500 chr3:2013384-2013429 1-2 33::0 34::20 4::30 30::44 0.099715856 1 Cassette

AT5G10070 chr5:3148901-3148907 9 46::105 126::112 43::189 93::256 0.100828475 1 A5SS

AT4G14960 chr4:8548878-8548964 2 2::15887 5::16986 8259::6191 8893::5963 0.101162783 1 IR

AT2G05440 chr2:1993847-1993886 3 268::147 597::27 1841::1812 2240::2494 0.101719268 1 A3SS

AT4G01915 chr4:828128-828212 3 108::84 64::154 57::98 92::96 0.102553935 1 IR

AT5G46470 chr5:18849609-18849687 11 4::60 24::14 30::74 13::44 0.102809685 1 IR

AT1G77080 chr1:28958311-28958501 2 50::51 109::43 51::151 188::271 0.102813114 1 MXE

AT3G23280 chr3:8323099-8323170 8 149::133 73::206 79::228 129::186 0.103007468 1 Cassette

AT1G12750 chr1:4345407-4345459 7 13::203 38::178 203::164 190::227 0.106054424 1 A3SS

AT4G36640 chr4:17278439-17278484 2 18::38 0::47 38::64 57::70 0.106800299 1 A3SS

AT3G13470 chr3:4392487-4392511 14 45::2011 15::2745 1989::1920 2718::2461 0.106811693 1 A5SS

AT4G38510 chr4:18014903-18015001 2 264::351 134::431 81::252 117::207 0.106982312 1 Cassette

AT4G33470 chr4:16103877-16103897 6 89::127 124::99 77::204 105::195 0.107579284 1 A5SS

AT1G28330 chr1:9934451-9934566 3 481::629 409::797 356::584 375::521 0.108317499 1 Cassette

AT4G02430 chr4:1069010-1069081 1 6::4 44::1 6::5 6::23 0.111437493 1 A5SS

AT5G42765 chr5:17150906-17151006 3 18::649 1::801 298::462 360::495 0.111646033 1 Cassette

AT2G35830 chr2:15052667-15052682 2 1131::752 841::797 1241::2060 975::1878 0.112064396 1 A3SS

AT2G01460 chr2:208994-209007 13 21::0 12::21 28::34 24::40 0.112434908 1 A3SS

AT4G36190 chr4:17125459-17125478 7 93::7 82::32 132::157 201::267 0.115007582 1 A3SS

AT4G02890 chr4:1279083-1279312 3 570::9059 395::8911 4787::4288 4678::4833 0.115809911 1 IR

AT5G54430 chr5:22097791-22097882 1-2 952::13 1043::49 13::760 53::750 0.115829658 1 Cassette

AT2G34410 chr2:14525272-14525352 17 85::97 79::49 34490::7799 25197::8990 0.11610912 1 AltEnd

AT5G46800 chr5:18990316-18990396 1-2 1832::19 1231::32 43::1906 42::1531 0.117361348 1 AltStart

AT5G06160 chr5:1865988-1866033 1-2 83::1 65::19 4::68 14::71 0.120145126 1 Cassette

AT3G56160 chr3:20838394-20838692 11 233::60 258::12 95::345 72::385 0.120408855 1 A3SS

AT4G00420 chr4:182576-182695 3 20::150 3::196 76::143 115::150 0.121214794 1 Cassette

AT1G73350 chr1:27576339-27576345 4 22::59 11::113 118::105 175::141 0.121489177 1 A3SS

AT2G29210 chr2:12559242-12559292 6 18::63 38::37 27::119 42::125 0.123184387 1 A3SS

AT2G34410 chr2:14522806-14522812 17 33::17 63::0 176855::7799 213055::8990 0.123743076 1 MXE

AT5G18240 chr5:6029602-6029623 6 14::23 41::16 19::48 46::78 0.123885697 1 A5SS

AT5G27390 chr5:9674820-9674892 6 15::829 0::1056 420::312 577::406 0.124280212 1 Cassette

AT2G36630 chr2:15353336-15353441 8 32::822 13::1268 474::458 703::694 0.124304541 1 IR

AT4G26140 chr4:13245233-13245253 9 8::21 33::10 13::39 36::61 0.12460671 1 A5SS

AT5G53460 chr5:21701958-21702080 5-6 180::46 84::56 60::233 58::173 0.125857299 1 Cassette_multi

AT2G41840 chr2:17461283-17461317 1 1::23217 317::21996 12252::8365 11417::8320 0.130578695 1 IR

AT1G07890 chr1:2437678-2437833 2 5297::64 4851::154 57::3056 111::2887 0.131156982 1 Cassette

AT4G03200 chr4:1408987-1409008 3 19::90 2::101 32::40 41::49 0.131530312 1 Cassette

AT1G33290 chr1:12074659-12074664 4 22::229 67::202 132::260 155::303 0.131550881 1 A3SS

AT4G28910 chr4:14266023-14266144 2 249::170 206::251 63::356 82::390 0.131629285 1 Cassette

AT5G32440 chr5:12078034-12078076 4 476::16 698::0 478::592 674::731 0.131838555 1 A3SS

AT1G19350 chr1:6689538-6689622 4 18::6062 5::6167 3241::2994 3270::3440 0.132217338 1 IR

AT3G15240 chr3:5134630-5134641 3 78::0 56::23 40::123 45::99 0.132695449 1 A5SS

AT3G52030 chr3:19302406-19302416 3 49::12 34::27 64::73 51::61 0.133753166 1 A3SS

AT5G45800 chr5:18576743-18576823 1-2 233::31 221::5 36::250 17::262 0.134042594 1 AltEnd

AT5G53050 chr5:21510163-21510421 11 234::163 414::151 160::292 219::432 0.13432854 1 AltEnd

AT5G51300 chr5:20849158-20849263 2 27::128 9::226 177::123 374::180 0.135697868 1 A3SS

AT1G12250 chr1:4159565-4159570 2 439::83 398::135 372::697 360::785 0.137645485 1 A3SS

AT3G61600 chr3:22797937-22798054 6 5::646 56::642 339::551 349::549 0.137846561 1 IR

AT2G32850 chr2:13935641-13935702 8 41::363 81::342 198::173 184::251 0.137851583 1 IR

AT4G34600 chr4:16529426-16529551 1-2 390::14 466::56 35::335 69::444 0.137920597 1 Cassette

AT3G15520 chr3:5252107-5252112 2 167::16 151::50 99::204 126::201 0.138129446 1 A3SS

AT2G05440 chr2:1993744-1993766 2 250::4995 635::5666 1770::4572 1792::5430 0.139300652 1 IR

AT4G25080 chr4:12876973-12877074 1 33::6490 36::5622 3829::4789 3230::4489 0.14140046 1 IR

AT1G19396 chr1:6711414-6711420 3 28::7 79::0 36::66 79::141 0.141798804 1 A3SS

AT4G37553 chr4:17644465-17644545 5 0::402 18::355 390::310 379::351 0.141848902 1 AltEnd

AT2G14045 chr2:5906998-5907124 2 97::16 171::3 168::197 245::274 0.144304792 1 A3SS

AT5G65050 chr5:25985654-25985925 6 14::326 1::632 25::102 42::160 0.145005101 1 Cassette

AT3G52340 chr3:19408045-19408106 5 203::46 207::11 336::415 366::385 0.145142058 1 A3SS

AT3G02300 chr3:462786-462954 6 131::40 94::68 82::200 137::193 0.145463537 1 A5SS

AT2G39280 chr2:16402820-16402829 14 15::52 7::124 63::67 124::123 0.146057354 1 A3SS

AT4G26140 chr4:13245145-13245160 10 8::21 33::10 20::38 30::48 0.146389046 1 A3SS

AT1G57680 chr1:21364018-21364116 2 23::224 8::190 287::548 282::701 0.148874268 1 A3SS

AT4G18975 chr4:10393673-10393695 2 47::31 16::36 45::84 47::61 0.149252352 1 A3SS

AT3G53270 chr3:19752428-19752580 3 31::101 5::110 44::83 57::83 0.149479837 1 Cassette_multi

AT1G02100 chr1:392072-392170 10 293::5 399::35 21::280 66::401 0.149583927 1 A5SS

AT3G55400 chr3:20536212-20536326 9 313::26 292::78 34::324 57::351 0.150331499 1 IR

AT2G43330 chr2:18002476-18002556 3-4 137::11 117::30 14::239 42::237 0.151132968 1 AltEnd

AT2G04790 chr2:1679886-1679909 2 216::68 163::105 131::337 180::344 0.152261378 1 A5SS

AT1G19680 chr1:6809239-6809250 6 17::22 3::44 33::37 58::47 0.152525132 1 A5SS

AT3G42150 chr3:14310533-14310649 3 110::483 58::627 204::292 276::343 0.152794093 1 Cassette

AT3G13440 chr3:4378334-4378414 2 81::130 57::171 78::230 102::265 0.152900332 1 Cassette

AT1G22750 chr1:8051455-8051556 4 18::905 0::968 428::505 466::516 0.153100581 1 Cassette

AT1G19715 chr1:6819412-6819818 1 58::114 38::229 36::159 91::213 0.15388715 1 Cassette

AT1G65370 chr1:24285757-24285873 2 19::406 2::665 194::213 317::292 0.154834572 1 IR

AT3G17365 chr3:5947793-5947809 6 20::12 44::3 23::38 34::55 0.154883148 1 A3SS

AT3G61870 chr3:22903436-22903717 2 2553::90 2741::201 118::1771 168::1852 0.156408615 1 IR

AT3G52560 chr3:19495898-19495921 1 9::1321 22::1365 11::575 33::590 0.156538242 1 A5SS

AT4G15000 chr4:8572265-8572362 1 1::9388 1::10886 5418::7351 6336::7813 0.156801749 1 IR

AT4G38770 chr4:18097496-18097587 2 770::12217 352::6913 5890::4520 3450::2941 0.156942413 1 IR

AT3G54500 chr3:20178021-20178064 4 23::1253 89::1654 1225::1236 1544::1583 0.157314232 1 A3SS

AT3G14420 chr3:4821638-4821788 1 7319::55 4461::101 502::8868 345::5800 0.158519009 1 IR

AT4G27960 chr4:13917240-13917343 1 773::311 598::354 211::1105 227::1049 0.159082094 1 IR

AT3G24520 chr3:8941626-8941682 1 36::156 69::78 187::81 94::57 0.159854263 1 A5SS

AT5G56190 chr5:22742575-22742685 2 30::10 27::52 9::45 34::60 0.160581812 1 IR

AT3G54760 chr3:20270980-20271074 2 8::177 17::111 112::130 59::140 0.161603405 1 IR

AT3G56130 chr3:20827693-20827901 3 766::56 711::127 52::808 71::803 0.161811082 1 IR

AT1G80860 chr1:30388302-30388379 2 52::190 116::149 122::218 90::238 0.161875278 1 IR

AT4G26795 chr4:13488956-13489036 1-2 12::120 16::42 171::65 66::49 0.162163167 1 AltStart

AT1G47240 chr1:17310307-17310366 2 22::42 1::59 58::66 76::87 0.164083926 1 A3SS

AT5G01470 chr5:192215-192227 6 175::11 117::26 76::161 72::136 0.164266296 1 A5SS

AT1G17550 chr1:6035811-6035859 2 53::141 24::183 92::225 61::238 0.164832879 1 A3SS

AT3G01150 chr3:52645-52746 2-3 99::23 82::52 13::156 24::130 0.164888649 1 Cassette

AT2G45770 chr2:18852776-18852795 8 316::2 258::18 273::279 216::263 0.166444641 1 A3SS

AT2G35390 chr2:14897107-14897438 1 289::41 249::102 26::282 59::310 0.168118919 1 IR

AT2G15530 chr2:6773255-6773392 2 24::6 15::27 7::38 15::26 0.168239764 1 Cassette

AT3G03040 chr3:685713-685778 3 45::4 48::30 89::118 149::227 0.168499321 1 A3SS

AT1G07650 chr1:2361682-2361761 18 14::99 7::213 47::78 115::112 0.168629258 1 IR

AT5G63120 chr5:25319643-25319829 9 83::22 90::57 19::131 52::140 0.169256892 1 Cassette

AT1G01060 chr1:33858-33981 10 61::1134 31::1646 526::1228 779::1496 0.171304573 1 IR

AT2G33340 chr2:14126492-14126577 18 82::1723 44::2306 946::647 1291::807 0.172376323 1 IR

AT1G52000 chr1:19335898-19335978 1-2 273::10 57::8 14::1021 4::421 0.172958114 1 AltStart

AT2G26770 chr2:11407823-11407884 2 135::89 78::103 46::206 40::153 0.173134783 1 Cassette

AT3G06190 chr3:1875381-1875463 3-4 317::19 264::41 22::335 34::345 0.173526226 1 Cassette

AT1G07110 chr1:2181582-2181606 10 214::30 222::67 65::245 115::319 0.175721346 1 A3SS

AT1G02160 chr1:411253-411336 2 447::46 462::118 59::364 103::416 0.175885153 1 IR

AT1G79870 chr1:30045333-30045367 2 732::5 564::10 716::768 553::778 0.176336831 1 A3SS

AT5G17170 chr5:5650435-5650520 4 76::3883 19::4265 1914::2234 2049::2310 0.176522854 1 Cassette

AT5G42540 chr5:17013408-17013439 19 14::91 4::153 102::72 167::112 0.177238966 1 A5SS

AT2G33800 chr2:14301691-14301712 1 4205::11 4211::16 4270::4104 4238::3185 0.177254161 1 A5SS

AT3G02600 chr3:551705-551718 3 59::6 91::30 26::131 54::183 0.177512157 1 Cassette

AT5G12130 chr5:3920090-3920095 3 15::185 44::147 90::207 94::201 0.178608581 1 A3SS

AT1G19720 chr1:6819430-6819818 2 58::114 38::229 37::103 94::147 0.179985027 1 Cassette

AT4G33520 chr4:16122210-16122242 8 33::13 14::29 17::44 38::43 0.181993913 1 A5SS

AT3G06125 chr3:1849036-1849141 1 435::2644 321::2785 1384::1180 1415::1182 0.182894911 1 IR

AT2G18280 chr2:7946689-7946721 2 43::787 92::715 147::849 200::900 0.182992647 1 A3SS

AT5G53050 chr5:21511319-21511426 8 170::119 442::131 325::521 664::1028 0.183751066 1 A5SS

AT2G26030 chr2:11092194-11092927 2 32::3 3::22 22::116 60::116 0.184383632 1 A3SS

AT5G38430 chr5:15384568-15384589 4 43::15942 0::16454 3167::8909 3100::11119 0.185212742 1 A3SS

AT5G53180 chr5:21570834-21570894 4 176::65 130::127 38::203 74::192 0.187874361 1 Cassette

AT1G22430 chr1:7920423-7920430 5 441::29 491::7 327::341 385::422 0.1886952 1 A3SS

AT4G18970 chr4:10389524-10389531 5 26::2058 23::1548 1294::1344 978::1229 0.189237829 1 A3SS

AT1G13460 chr1:4615978-4616059 1 36::9 30::10 7::31 19::30 0.189918353 1 A5SS

AT2G47020 chr2:19319816-19319851 11 64::6 41::24 283::282 397::350 0.190432112 1 A3SS

AT5G57565 chr5:23311437-23311564 4 14::4 5::18 6::19 9::23 0.19054454 1 IR

AT5G47435 chr5:19242564-19242723 4 16::796 1::1033 405::439 483::557 0.192343073 1 Cassette

AT4G02260 chr4:990617-990662 21 26::190 9::286 182::221 300::257 0.19289515 1 A5SS

AT2G30695 chr2:13079926-13079939 7 5::156 16::89 98::268 75::256 0.193187174 1 Cassette

AT1G48315 chr1:17855368-17855452 3 308::67 312::122 58::277 93::238 0.193333868 1 IR

AT3G28970 chr3:10989359-10989485 2 19::92 7::141 49::57 65::59 0.193985706 1 Cassette

AT4G12780 chr4:7513910-7513992 2 14::190 20::75 103::139 41::117 0.194318904 1 IR

AT3G56210 chr3:20855272-20855283 2 1::242 22::262 217::219 241::258 0.197243929 1 A3SS

AT4G11830 chr4:7119578-7120374 2 18::32 37::16 29::174 65::163 0.199226629 1 A5SS

AT1G17520 chr1:6025505-6025523 4 63::34 91::15 69::103 88::105 0.200640246 1 A5SS

AT5G25520 chr5:8888669-8888699 6 16::114 3::105 33::149 15::198 0.200802382 1 A3SS

AT5G55580 chr5:22515794-22515879 1 32::41 3::64 66::59 109::60 0.20122164 1 A5SS

AT2G27730 chr2:11821607-11821646 1-2 1565::36 1942::24 31::1330 23::1611 0.201498715 1 Cassette

AT3G11940 chr3:3779356-3779442 1 8491::120 8920::65 448::7781 431::8383 0.203096423 1 IR

AT1G70850 chr1:26716231-26716400 2 269::9132 182::5708 9438::7630 6018::6233 0.206718618 1 Cassette

AT1G10870 chr1:3617929-3617944 17 82::23 89::1 48::124 33::94 0.20677051 1 A3SS

AT3G46020 chr3:16913214-16913221 1 53::1 59::28 30::17 31::23 0.207570123 1 A5SS

AT5G02240 chr5:451707-451833 2 1313::28 1074::0 1395::1396 1131::1285 0.209300323 1 A3SS

AT5G53486 chr5:21721957-21722013 2 138::40 193::25 21::184 17::190 0.209616306 1 Cassette

AT3G13000 chr3:4160876-4160961 2 98::50 75::8 32::69 12::57 0.210408764 1 IR

AT4G13040 chr4:7614615-7614668 5 7::129 31::135 139::137 135::157 0.211218139 1 A5SS

AT1G09140 chr1:2943226-2943564 11 257::348 462::443 288::569 353::897 0.212072882 1 A3SS

AT3G52720 chr3:19541256-19541284 1 23::211 25::135 89::1202 57::1016 0.212168261 1 AltStart

AT2G01260 chr2:137076-137164 2 28::88 8::141 84::123 133::154 0.213173128 1 A3SS

AT2G28910 chr2:12417126-12417207 1-2 2447::49 2263::18 27::2159 10::2028 0.215803072 1 Cassette

AT1G61520 chr1:22700295-22700385 2 62625::217 48854::240 1534::45485 1234::41643 0.217144072 1 IR

AT2G26210 chr2:11157616-11157688 2 105::113 127::71 166::317 206::296 0.217487913 1 A3SS

AT4G08980 chr4:5758484-5758982 2 184::98 217::75 57::436 48::542 0.218009896 1 IR

AT1G49600 chr1:18357545-18357625 5-6 1544::23 2212::15 16::1026 21::1280 0.218604493 1 AltStart

AT5G18240 chr5:6030664-6030790 1 14::5 10::33 16::19 15::53 0.218919239 1 A5SS

AT1G05710 chr1:1716196-1716212 4 66::38 75::12 92::162 107::159 0.219749783 1 A3SS

AT1G72640 chr1:27346899-27346919 7 56::203 93::158 262::345 233::453 0.221132014 1 A3SS

AT4G36890 chr4:17379522-17379717 3 40::205 2::267 80::93 108::94 0.222372252 1 IR

AT1G07119 chr1:2185391-2185436 3-4 13::34 27::20 25::51 17::45 0.223268212 1 Cassette

AT1G54730 chr1:20429773-20429854 16 74::16 89::89 15::30 50::47 0.223500204 1 IR

AT1G02100 chr1:392495-392576 12 189::368 326::356 218::281 205::384 0.224206957 1 IR

AT1G16010 chr1:5497437-5497527 1 163::16 171::37 28::189 39::160 0.225009323 1 IR

AT1G70830 chr1:26710568-26710884 2 5082::335 4943::467 5266::4135 4771::3106 0.225727638 1 A5SS

AT2G47250 chr2:19400816-19400925 4 192::19 179::27 61::310 144::385 0.226048864 1 A3SS

AT5G20140 chr5:6799091-6799161 8 215::55 344::37 73::160 69::230 0.226336666 1 A3SS

AT2G04540 chr2:1582178-1582220 11 91::4 114::21 16::90 43::141 0.226822702 1 A3SS

AT2G26280 chr2:11191096-11191159 1-2 46::161 69::136 155::118 113::100 0.228211658 1 Cassette

AT2G05520 chr2:2026650-2026671 3 328::999 523::1346 6191::8478 7958::12867 0.228944836 1 A3SS

AT1G25054 chr1:8799542-8799622 3-4 540::3414 835::3789 9::5 10::5 0.229448967 1 AltEnd

AT5G67140 chr5:26794495-26794630 3 43::111 26::156 76::139 105::116 0.22969697 1 Cassette

AT5G19221 chr5:6465490-6465501 4 66::758 105::688 566::774 550::747 0.232024301 1 A5SS

AT1G20620 chr1:7145780-7145880 5 12772::142 14903::92 684::12808 747::15313 0.234698085 1 IR

AT4G00430 chr4:186473-186602 2 6941::62 6077::142 343::7351 343::6165 0.236218309 1 IR

AT3G57880 chr3:21433637-21433689 2 14::163 6::72 84::185 39::169 0.237172401 1 Cassette

AT1G68310 chr1:25600988-25601033 6 39::679 17::806 317::225 377::274 0.23721822 1 Cassette

AT4G12560 chr4:7443193-7443376 2 149::24 159::75 79::213 131::243 0.237802478 1 A5SS

AT3G61010 chr3:22574724-22574792 2 18::43 10::84 47::50 105::124 0.239265495 1 A3SS

AT5G53048 chr5:21510708-21510730 2 164::188 298::181 268::468 421::769 0.239505894 1 A5SS

AT3G23900 chr3:8631915-8631922 7 36::14 76::14 46::49 87::110 0.240659134 1 A5SS

AT5G43320 chr5:17386658-17386704 12-13 570::1 634::22 36::573 46::650 0.24132829 1 Cassette

AT3G51390 chr3:19076672-19076687 5 52::27 76::8 76::112 98::113 0.241557598 1 A3SS

AT1G80190 chr1:30159245-30159332 1 1::51 32::48 46::17 54::40 0.242375586 1 A5SS

AT1G25098 chr1:8813920-8814342 3 470::3498 881::4196 3::20 4::17 0.243022886 1 Cassette

AT5G51720 chr5:21009812-21009868 1 74::2828 27::4037 2987::2159 4145::2746 0.245006365 1 A5SS

AT4G04223 chr4:2036892-2036933 2 392::507 167::133 246::248 68::66 0.246350708 1 Cassette

AT4G27700 chr4:13826932-13826974 4 2305::48 2264::16 2135::2110 2124::2064 0.246599966 1 A5SS

AT5G19220 chr5:6465044-6465316 8 26::1397 1::1445 614::745 668::819 0.246670244 1 Cassette_multi

AT4G15030 chr4:8580243-8580248 7 15::2 7::13 15::21 16::27 0.247224294 1 A3SS

AT4G39260 chr4:18274693-18274773 1-2 3069::312 5610::368 754::2454 1355::4476 0.24729261 1 AltStart

AT5G01920 chr5:361627-361726 5 66::118 32::280 69::113 151::187 0.247859593 1 IR

AT3G23090 chr3:8214851-8214863 6 3::114 24::99 48::108 58::113 0.248431247 1 A5SS

AT5G37475 chr5:14868234-14868310 1-2 479::27 513::9 21::308 9::320 0.248461151 1 Cassette

AT4G07990 chr4:4827486-4827527 3 101::14 133::50 9::96 26::112 0.248725856 1 Cassette

AT1G34270 chr1:12493690-12493723 2 32::18 10::23 22::46 31::50 0.248874451 1 A3SS

AT5G53580 chr5:21765999-21766009 4 403::30 448::73 293::566 339::634 0.248936122 1 A5SS

AT2G43160 chr2:17952780-17952909 14 788::0 826::0 969::720 1009::912 0.250683292 1 A3SS

AT2G45170 chr2:18624378-18624537 1 363::93 376::44 70::272 42::266 0.251762548 1 IR

AT4G01897 chr4:821095-821169 2 20::371 9::514 171::146 245::190 0.252230544 1 Cassette

AT1G19080 chr1:6584426-6584556 2 53::3 31::19 10::50 26::45 0.252606613 1 Cassette

AT1G01830 chr1:302048-302054 1 16::13 4::31 13::12 8::14 0.253067182 1 A5SS

AT1G05710 chr1:1716559-1716624 6 24::330 7::393 170::201 226::234 0.253315214 1 Cassette

AT4G15160 chr4:8646305-8646552 1 0::423 0::465 250::244 242::343 0.253663051 1 IR

AT2G01270 chr2:139620-139650 1 33::171 8::204 233::214 262::197 0.253755994 1 A5SS

AT4G00560 chr4:241247-241370 4 109::263 119::468 147::246 245::388 0.254600282 1 Cassette

AT2G01520 chr2:236613-236627 2 14429::134 7509::0 13227::7594 6976::4758 0.25561974 1 A3SS

AT1G77080 chr1:28958663-28958745 4 160::33 161::127 29::184 78::295 0.256062625 1 IR

AT2G26030 chr2:11092194-11093097 1 32::87 2::126 22::85 56::83 0.256629725 1 IR

AT2G01220 chr2:125274-125383 9 20::373 13::603 185::244 303::349 0.257034698 1 Cassette

AT2G20180 chr2:8706315-8706352 3 40::28 6::52 38::201 59::208 0.257148322 1 A3SS

AT5G65440 chr5:26156500-26156584 19 67::17 58::60 20::83 41::118 0.257157457 1 IR

AT1G80500 chr1:30271720-30271746 2-3 580::24 764::17 20::536 25::709 0.259631335 1 Cassette

AT5G18940 chr5:6327326-6327391 7 16::307 0::328 166::170 175::179 0.260163461 1 Cassette

AT1G17100 chr1:5845110-5845129 2 2532::14 1871::15 2423::1971 1850::1872 0.260846995 1 A3SS

AT2G45245 chr2:18659991-18660105 3 74::7 66::14 3::71 13::67 0.261698763 1 Cassette

AT5G35170 chr5:13421479-13421489 8 12::233 30::148 184::359 251::373 0.263080701 1 A3SS

AT4G33000 chr4:15926141-15926157 9 49::17 95::7 144::216 202::263 0.263893589 1 A3SS

AT1G50440 chr1:18689702-18689713 8 17::379 34::283 99::365 93::267 0.265466468 1 A3SS

AT2G22980 chr2:9782891-9782998 13 27::48 80::37 18::20 24::45 0.265658486 1 IR

AT2G39930 chr2:16669274-16669290 9 125::0 111::19 23::133 39::124 0.267442667 1 A5SS

AT4G03110 chr4:1376760-1376856 8 333::22 405::81 28::259 66::321 0.267727686 1 IR

AT5G09660 chr5:2993904-2993978 6 9727::311 9524::457 1138::9891 1201::9803 0.267988763 1 IR

AT1G21310 chr1:7454024-7454182 1 47::0 61::97 3101::6572 3383::8517 0.2689902 1 A3SS

AT2G36410 chr2:15279590-15279628 1-2 272::16 268::0 5::250 6::217 0.270303564 1 Cassette

AT5G09880 chr5:3083405-3083485 4-5 21::27 14::59 93::175 133::294 0.270336364 1 Cassette

AT3G10350 chr3:3209053-3209064 4 13::277 29::225 112::267 107::223 0.270949759 1 A3SS

AT1G27920 chr1:9728488-9728520 5 19::6 51::2 10::30 8::55 0.271036987 1 A5SS

AT2G28320 chr2:12097040-12097045 11 80::8 61::27 50::97 41::78 0.27116862 1 A5SS

AT1G52500 chr1:19561477-19561527 6 22::64 17::162 38::311 82::439 0.271523029 1 Cassette

AT5G18240 chr5:6030581-6030596 2 10::6 3::36 27::38 46::69 0.271658003 1 A3SS

AT3G43540 chr3:15431017-15431095 1 149::56 113::113 50::167 74::173 0.272533366 1 IR

AT1G19270 chr1:6664583-6664618 7 23::11 13::16 26::38 19::48 0.273809986 1 A3SS

AT3G19170 chr3:6630137-6630257 6 315::0 151::0 320::567 157::463 0.27418076 1 MXE

AT5G14200 chr5:4577950-4578037 8 2634::6 4426::75 246::1613 440::2690 0.274196759 1 IR

AT4G25080 chr4:12878339-12878382 4 1550::669 1748::1047 1472::1052 1689::1405 0.27557615 1 A3SS

AT4G30993 chr4:15099410-15099504 6 489::55 549::115 67::556 101::623 0.275846176 1 IR

AT4G24810 chr4:12787436-12787486 9 94::18 177::18 144::145 207::183 0.276321759 1 A5SS

AT2G04380 chr2:1525695-1525742 1 207::0 69::0 340::547 609::656 0.277030427 1 A5SS

AT4G28060 chr4:13944154-13944185 3 1636::7 1877::30 1720::2110 1967::2329 0.277076823 1 A5SS

AT3G48420 chr3:17930741-17930755 4 818::32 824::64 290::825 325::815 0.277209614 1 A5SS

AT5G58005 chr5:23482942-23482963 4 26::243 9::371 58::130 67::171 0.277324402 1 A3SS

AT1G17550 chr1:6034399-6034608 1 6::98 9::39 67::155 27::132 0.27759103 1 IR

AT1G06700 chr1:2055126-2055143 1 73::316 123::253 140::95 174::97 0.279322824 1 A5SS

AT5G44030 chr5:17716408-17716425 7 3::32 12::9 7::32 19::35 0.28059217 1 A3SS

AT5G59950 chr5:24140405-24140602 1 592::243 987::249 130::416 150::636 0.280703369 1 IR

AT1G53233 chr1:19850221-19850286 2 20::0 22::0 259::256 414::275 0.280886528 1 A3SS

AT5G48545 chr5:19676958-19676968 4 147::195 217::195 293::482 374::584 0.281445986 1 A3SS

AT4G37550 chr4:17644396-17644545 3 0::402 18::355 417::364 395::350 0.282730715 1 A5SS

AT1G21310 chr1:7453831-7453916 1 539::12116 1749::14590 6019::3048 6916::3376 0.283474031 1 IR

AT3G07670 chr3:2452128-2452196 2 9::172 23::125 21::183 32::139 0.284295952 1 A3SS

AT1G19660 chr1:6802247-6802325 2 722::0 383::5 31::745 45::500 0.285150911 1 A3SS

AT2G44065 chr2:18228670-18228715 2 240::58 269::36 28::233 17::250 0.285568132 1 Cassette

AT5G18380 chr5:6090206-6090353 1 0::25966 0::27863 13161::16255 14315::16719 0.28562297 1 IR

AT2G16940 chr2:7345946-7346177 4 225::52 207::87 58::318 92::305 0.285867771 1 Cassette_multi

AT3G24430 chr3:8870911-8870924 5 495::23 317::37 56::667 61::474 0.286036971 1 A3SS

AT2G46820 chr2:19243589-19243700 1 115::6663 104::5157 2444::11765 1838::10125 0.286234948 1 IR

AT2G46910 chr2:19272858-19272869 4 18::421 35::387 79::371 100::406 0.286770839 1 A3SS

AT3G25840 chr3:9456874-9456929 2 31::28 33::6 19::56 7::49 0.287148612 1 Cassette

AT5G17440 chr5:5751858-5751904 7-8 61::15 140::12 34::109 31::164 0.287707378 1 Cassette

AT3G60300 chr3:22286957-22286965 8 671::16 785::12 650::525 824::748 0.288310907 1 A3SS

AT2G46830 chr2:19246681-19246751 5 331::6 261::13 147::753 183::745 0.288615388 1 AltStart

AT5G59880 chr5:24121496-24121542 3 0::8416 0::9521 4219::2306 4732::2802 0.288666958 1 IR

AT4G05040 chr4:2579601-2579722 3 16::35 1::42 29::49 30::64 0.288682656 1 Cassette

AT1G52100 chr1:19385233-19385480 1 6::65 16::23 8::18 12::8 0.289606634 1 A5SS

AT1G01060 chr1:33666-33858 10-11 4::61 5::31 241::1472 334::1455 0.289918 1 AltEnd

AT1G05000 chr1:1426235-1426330 3 304::24 375::52 19::345 35::453 0.290455077 1 Cassette

AT3G49100 chr3:18197822-18197841 3 234::15 315::2 239::281 335::362 0.290655712 1 A3SS

AT1G61150 chr1:22542863-22542958 3 61::109 75::84 86::157 120::172 0.290921837 1 A3SS

AT1G43170 chr1:16268680-16268745 6 0::4107 0::4673 1870::5715 2101::6982 0.291451563 1 IR

AT2G26340 chr2:11216092-11216101 5 844::41 905::17 536::975 587::996 0.291527159 1 A3SS

AT4G37550 chr4:17644602-17644641 4 0::402 21::355 42::446 50::463 0.291736309 1 A3SS

AT1G52500 chr1:19560978-19561046 4 22::67 17::110 40::311 57::439 0.292192197 1 Cassette

AT5G61960 chr5:24878830-24878979 15 31::344 2::569 141::165 225::253 0.29312454 1 IR

AT5G49840 chr5:20257876-20257910 10 60::7 90::9 31::77 25::104 0.293190678 1 A5SS

AT5G65720 chr5:26296502-26296580 1 2::1099 1::867 572::652 449::647 0.293796424 1 IR

AT4G26790 chr4:13488192-13488230 1 3::91 17::33 121::66 61::47 0.293873834 1 A5SS

AT4G32660 chr4:15757684-15757798 7 19::223 9::382 144::162 230::251 0.294235577 1 Cassette

AT3G02600 chr3:551800-551847 3-4 8::30 12::25 50::290 69::383 0.294473936 1 MXE

AT1G76930 chr1:28896371-28896684 1 0::1361 18::1028 405::570 297::322 0.29453737 1 IR

AT1G02280 chr1:450251-450258 2 62::533 87::447 518::754 484::799 0.294640259 1 A3SS

AT5G50565 chr5:20580493-20580565 1 11::49 41::40 3::3 3::3 0.294757003 1 A5SS

AT1G72500 chr1:27297897-27298055 2 32::173 6::153 65::111 68::98 0.295246216 1 Cassette

AT5G65060 chr5:25989671-25989728 2 43::33 57::86 18::59 46::98 0.295504416 1 Cassette

AT4G16990 chr4:9564677-9564692 9 32::161 34::311 169::304 281::674 0.295727981 1 A3SS

AT1G65486 chr1:24349740-24349870 2 192::6 251::39 6::204 26::280 0.297381015 1 AltStart

AT3G53100 chr3:19685574-19685590 4 36::196 69::157 220::363 222::353 0.298286789 1 A3SS

AT1G08570 chr1:2712985-2713096 1 24::2191 30::1236 1199::1062 635::689 0.298661049 1 IR

AT5G65685 chr5:26275481-26275514 3 14::132 5::191 68::80 109::102 0.29955413 1 Cassette_multi

AT3G55770 chr3:20704752-20704876 7 2::51 8::146 28::1249 68::1506 0.299631046 1 IR

AT5G11860 chr5:3824070-3824193 1 17::73 2::63 23::30 9::28 0.299658539 1 A5SS

AT2G05440 chr2:1993831-1993846 3 7::52 43::42 1121::3662 1359::4448 0.29996283 1 Cassette

AT4G08930 chr4:5730166-5730171 3 207::28 254::11 32::237 31::289 0.300481152 1 A5SS

AT3G61420 chr3:22730924-22730934 6 14::27 9::63 24::48 48::79 0.300774364 1 A3SS

AT2G40008 chr2:16710081-16710095 3 62::123 104::144 120::166 151::204 0.300787357 1 A5SS

AT4G10070 chr4:6297466-6297606 6-7 24::69 16::96 42::128 62::157 0.300834085 1 Cassette

AT1G08520 chr1:2699467-2699474 10 900::0 955::16 557::935 601::1001 0.301626995 1 A3SS

AT5G52040 chr5:21131562-21131887 3 508::53 477::69 60::626 117::671 0.302262511 1 Cassette

AT2G22570 chr2:9589982-9590081 3 66::3 49::25 7::69 20::87 0.302515804 1 IR

AT1G16460 chr1:5620312-5620331 10 580::2 626::21 506::557 550::596 0.302813811 1 A5SS

AT1G67300 chr1:25194740-25194778 10 25::58 25::29 98::100 82::125 0.302935073 1 A3SS

AT1G14170 chr1:4845150-4845226 2 5::7 3::28 6::23 19::30 0.303203942 1 IR

AT3G47630 chr3:17565340-17565422 1 6::30 7::42 10::33 6::26 0.303354419 1 A5SS

AT5G23410 chr5:7886163-7886178 2 1::66 23::69 14::39 33::59 0.303616922 1 A5SS

AT4G16143 chr4:9134324-9134448 9 695::473 973::447 274::636 284::806 0.303943176 1 IR

AT2G34080 chr2:14394014-14394033 2 15::9 9::12 30::65 101::89 0.305175577 1 A3SS

AT4G01450 chr4:610307-610361 6 270::1 237::3 286::165 274::207 0.305899563 1 A3SS

AT5G44290 chr5:17843433-17843493 2 21::38 5::42 54::84 48::84 0.306581668 1 Cassette

AT1G76930 chr1:28896046-28896094 1 73::45 71::22 1453::655 1378::503 0.307309212 1 A5SS

AT4G14410 chr4:8300299-8300456 2 4::308 21::252 22::366 53::394 0.30796609 1 A3SS

AT1G49670 chr1:18384473-18384560 6 21::112 6::165 66::66 92::91 0.308125015 1 Cassette

AT1G12520 chr1:4268447-4268529 2 56::73 51::165 39::87 86::126 0.309247838 1 IR

AT5G57270 chr5:23203375-23203390 2 24::13 34::0 21::27 15::24 0.309554502 1 A3SS

AT4G18710 chr4:10298368-10299362 8 0::968 8::794 801::670 822::546 0.30978802 1 AltEnd

AT1G05870 chr1:1771814-1771892 4 68::100 66::237 64::131 143::190 0.310659426 1 IR

AT5G23150 chr5:7792087-7792208 11 18::182 1::262 85::61 107::79 0.310813076 1 IR

AT4G13575 chr4:7893244-7893289 3 83::137 207::200 92::247 234::492 0.311122589 1 A5SS

AT4G24440 chr4:12633289-12633328 1 137::498 110::653 591::414 725::432 0.311550215 1 A5SS

AT1G59520 chr1:21865663-21865768 11 51::26 78::8 76::92 131::149 0.313542478 1 A3SS

AT5G17010 chr5:5587752-5587844 14 157::125 238::91 72::124 48::156 0.314036833 1 IR

AT1G48550 chr1:17951839-17951855 6 6::69 28::59 70::95 77::124 0.314116434 1 A3SS

AT5G16610 chr5:5447351-5447583 4 0::31 0::59 31::18 34::37 0.314529031 1 Cassette

AT5G55550 chr5:22501918-22502046 5 0::10 0::54 6::8 36::11 0.315619468 1 IR

AT1G72650 chr1:27351246-27351264 4 56::0 48::14 19::69 42::74 0.317656643 1 A5SS

AT1G67060 chr1:25037148-25037198 2 169::19 157::3 32::185 23::198 0.318263203 1 A5SS

AT3G06510 chr3:2018188-2018291 6 660::164 645::247 132::826 162::841 0.319149388 1 IR

AT1G69250 chr1:26034711-26034810 6 271::19 254::47 26::242 37::257 0.319155546 1 IR

AT2G43010 chr2:17887985-17887996 4 482::54 315::66 253::650 213::493 0.31994097 1 A5SS

AT1G80320 chr1:30197639-30197672 3 11::7 8::35 19::26 20::55 0.320031043 1 A3SS

AT3G62750 chr3:23215854-23215864 9 90::27 201::11 99::184 164::280 0.32162394 1 A3SS

AT2G20210 chr2:8724287-8724304 5 10::4 8::12 10::18 15::27 0.32177091 1 A3SS

AT3G27831 chr3:10319077-10319152 3 830::93 1055::105 709::450 969::448 0.321880234 1 A3SS

AT3G59210 chr3:21889695-21889781 1 16::22 7::37 14::45 21::55 0.321986117 1 IR

AT1G67790 chr1:25418000-25418098 4 17::21 26::19 15::35 13::42 0.322003626 1 IR

AT2G47910 chr2:19615006-19615015 2 21::762 16::620 416::729 316::735 0.322641799 1 A3SS

AT1G79040 chr1:29736166-29736213 1 16::219305 5::199334 113101::67699 102333::58865 0.323309839 1 IR

AT5G45510 chr5:18448938-18449021 3 61::248 61::520 97::222 124::383 0.323471857 1 A3SS

AT5G11380 chr5:3632733-3632738 9 4::110 27::86 80::157 105::204 0.324667493 1 A3SS

AT2G02910 chr2:848125-848130 6 42::13 28::33 19::69 17::81 0.325425594 1 A5SS

AT5G40550 chr5:16242035-16242044 4 9::4 5::8 9::18 8::25 0.325469635 1 A3SS

AT3G50950 chr3:18936110-18936116 2 29::36 9::39 42::116 25::152 0.326711067 1 A3SS

AT5G26980 chr5:9488446-9488705 8 5::15 24::1 35::72 58::74 0.32773234 1 A5SS

AT5G53450 chr5:21689190-21689269 2 133::11 53::22 169::187 61::85 0.328551936 1 A3SS

AT1G80030 chr1:30105250-30105377 11 78::414 133::411 173::145 180::217 0.328831624 1 IR

AT1G13650 chr1:4681946-4682066 1 6::115 25::83 84::43 61::41 0.329469859 1 IR

AT4G03250 chr4:1426446-1426511 4 9::18 11::23 6::37 20::36 0.330363784 1 A3SS

AT3G24190 chr3:8745051-8745056 6 458::16 280::31 208::464 145::303 0.33059913 1 A5SS

AT3G58640 chr3:21692693-21692792 2 145::15 127::43 17::180 30::145 0.331325991 1 IR

AT3G12570 chr3:3989180-3989266 2 5::258 10::184 206::294 191::356 0.331394309 1 Cassette

AT1G12650 chr1:4306149-4306169 2 1::109 8::64 13::74 17::57 0.331488046 1 A3SS

AT2G35820 chr2:15051084-15051089 1 627::5 660::29 421::571 476::597 0.331724138 1 A5SS

AT3G55770 chr3:20704674-20704775 7 1913::12 2146::24 41::1792 107::2102 0.331782858 1 Cassette

AT3G56860 chr3:21050142-21050491 2 223::221 186::361 59::262 112::361 0.333287486 1 AltEnd

AT4G11830 chr4:7116967-7116999 9 75::18 149::16 73::66 170::201 0.333828621 1 A5SS

AT2G16600 chr2:7201012-7201079 1 0::11738 0::11674 6247::4953 6167::5226 0.333835588 1 IR

AT2G30530 chr2:13010219-13010224 4 173::24 198::63 105::201 118::261 0.334367167 1 A5SS

AT1G49180 chr1:18185655-18185734 9 9::27 48::32 21::38 35::76 0.334426309 1 A3SS

AT1G18420 chr1:6345051-6345240 7 10::27 7::10 17::30 9::43 0.33450448 1 IR

AT1G70830 chr1:26710310-26710480 3 365::5084 467::4937 4721::2458 4972::3225 0.336300735 1 A3SS

AT1G59218 chr1:21832476-21832708 6 105::37 138::107 3::3 5::3 0.338370039 1 AltEnd

AT1G14660 chr1:5034008-5034028 14 10::5 9::29 10::17 22::25 0.339358878 1 A3SS

AT2G05520 chr2:2026671-2026713 3 999::119 1346::225 4312::8911 5722::13609 0.339681499 1 A3SS

AT5G54940 chr5:22309216-22309280 2 2407::0 1812::17 76::2498 54::2272 0.341540486 1 Cassette

AT4G24960 chr4:12828253-12828304 2 778::1 619::14 48::704 46::582 0.341570414 1 A5SS

AT5G14260 chr5:4604042-4604127 12 670::10 724::31 68::811 96::892 0.341991923 1 A5SS

AT4G03200 chr4:1408786-1409008 2 14::84 2::117 46::40 50::49 0.342113534 1 Cassette_multi

AT2G46790 chr2:19233322-19233330 2 39::13 30::35 35::57 55::71 0.342134116 1 A5SS

AT2G37690 chr2:15807258-15807271 5 0::73 16::79 3::73 19::95 0.342868599 1 A3SS

AT1G73090 chr1:27489560-27489574 4 148::2 118::22 65::98 69::95 0.344312564 1 A5SS

AT4G00590 chr4:254369-254376 8 32::16 50::5 39::63 57::64 0.345156687 1 A3SS

AT5G53050 chr5:21510421-21510501 10 163::300 151::524 91::266 142::419 0.345554606 1 IR

AT4G13040 chr4:7614072-7614142 3 37::105 22::167 64::129 103::138 0.34666776 1 Cassette

AT2G04790 chr2:1680176-1680269 3 225::37 231::92 34::182 59::201 0.347225435 1 IR

AT1G52500 chr1:19561140-19561265 5 22::66 17::125 52::311 62::439 0.347664845 1 Cassette

AT4G21980 chr4:11655775-11655896 1 382::46 485::18 44::182 39::228 0.347906885 1 IR

AT3G20320 chr3:7088580-7088591 4 263::25 282::57 57::310 79::359 0.348298479 1 A3SS

AT3G61860 chr3:22901255-22901768 3 250::23 264::70 47::354 89::442 0.348523436 1 A3SS

AT2G43150 chr2:17946268-17946317 1 541::5065 670::6339 2444::2060 3139::2853 0.348763251 1 IR

AT1G15215 chr1:5239715-5239726 9 2::34 7::49 8::39 37::53 0.348791354 1 A3SS

AT3G28080 chr3:10451665-10451763 1 78::57 110::33 61::91 45::95 0.349001717 1 A5SS

AT2G17320 chr2:7533688-7533713 11 129::15 155::3 145::184 178::219 0.349365983 1 A5SS

AT5G25520 chr5:8888098-8888304 4 88::22 78::54 22::125 54::146 0.349469043 1 Cassette

AT2G05520 chr2:2026649-2026671 2 833::13061 1011::16995 6240::9419 8010::13449 0.349670647 1 IR

AT3G15660 chr3:5309449-5309527 1 37::471 49::353 244::441 188::409 0.350179466 1 IR

AT5G58140 chr5:23528237-23528247 15 58::821 97::796 282::909 307::964 0.351473516 1 A3SS

AT4G04850 chr4:2454818-2454832 7 395::1 329::16 117::398 111::327 0.351786132 1 A5SS

AT1G07280 chr1:2238390-2238395 3 159::0 155::0 142::293 136::273 0.351813222 1 A3SS

AT1G60230 chr1:22213448-22213458 5 117::11 116::36 74::150 91::156 0.351834714 1 A3SS

AT4G19830 chr4:10773408-10773530 2 19::357 7::470 172::178 222::206 0.352213847 1 Cassette

AT5G54980 chr5:22316306-22316313 3 404::8 248::18 234::201 155::151 0.352428887 1 A3SS

AT2G30260 chr2:12906987-12907022 2 16::221 9::304 245::343 365::402 0.35248162 1 A3SS

AT5G05080 chr5:1500597-1500605 1 461::40 500::20 344::269 341::218 0.352497617 1 A5SS

AT1G33110 chr1:12008023-12008128 5 457::41 546::105 44::562 78::654 0.353303627 1 IR

AT5G53620 chr5:21781075-21781174 1 140::20 69::29 17::138 16::97 0.353379035 1 IR

AT5G24310 chr5:8274586-8274647 2 9::11 21::5 24::56 29::41 0.353964154 1 A3SS

AT4G05040 chr4:2579722-2579881 3 10::39 6::36 18::48 12::63 0.35410123 1 IR

AT2G43560 chr2:18074401-18074448 5 1280::29 1674::25 132::1381 194::1793 0.354656737 1 A3SS

AT1G27920 chr1:9729487-9729526 10 11::7 18::11 15::29 30::44 0.354659481 1 A5SS

AT1G44446 chr1:16849850-16849891 5 61::2293 61::1513 2437::2623 1665::1851 0.355750738 1 A3SS

AT4G26400 chr4:13346388-13346405 1 124::22 107::44 143::104 122::97 0.35615838 1 A5SS

AT5G22720 chr5:7548817-7548921 5 14::45 8::95 72::66 91::84 0.35633267 1 Cassette_multi

AT2G05440 chr2:1993766-1993787 3 429::530 999::637 1226::872 1343::1055 0.356523385 1 A3SS

AT3G59780 chr3:22089133-22089138 8 773::65 906::111 432::861 520::1029 0.358721863 1 A3SS

AT4G27800 chr4:13852298-13852383 9 737::82 789::150 107::682 146::763 0.359018956 1 IR

AT1G80360 chr1:30210762-30210803 1 21::109 31::85 88::511 76::569 0.35902593 1 AltStart

AT3G52155 chr3:19344553-19344561 4 12::406 5::486 312::496 372::516 0.359506882 1 A5SS

AT5G51050 chr5:20754351-20754365 2 2::34 32::45 16::60 37::77 0.360560068 1 A3SS

AT1G67870 chr1:25449681-25449730 2 496::11486 577::12742 5853::3983 6576::4097 0.361469644 1 IR

AT5G03040 chr5:710283-710379 6 19::538 10::638 333::867 399::959 0.361491032 1 IR

AT2G28290 chr2:12070737-12070839 34 28::11 28::15 26::46 19::55 0.361651462 1 A5SS

AT4G17240 chr4:9667798-9667894 1 59::0 22::0 77::48 29::36 0.361815306 1 A5SS

AT3G63290 chr3:23381949-23382085 1 31::19 41::22 11::52 16::54 0.361918354 1 IR

AT5G39830 chr5:15945002-15945044 10 174::11 307::13 155::179 289::296 0.362475645 1 A3SS

AT1G28680 chr1:10079363-10079397 2 1::301 21::388 323::298 417::292 0.363340911 1 A5SS

AT1G61150 chr1:22542810-22542862 2 111::118 117::84 46::114 32::113 0.363544623 1 Cassette

AT3G56590 chr3:20967625-20967699 4 182::31 274::114 42::228 98::317 0.364494262 1 IR

AT2G45810 chr2:18861635-18861708 6 333::14 348::2 397::450 421::544 0.364509573 1 A3SS

AT2G43080 chr2:17917826-17917831 9 16::94 31::182 6::124 13::196 0.364816491 1 A3SS

AT4G32850 chr4:15854241-15854327 13 62::93 63::170 29::48 75::62 0.365596075 1 IR

AT3G17310 chr3:5913038-5913149 1 34::78 18::63 43::56 37::30 0.366037348 1 IR

AT2G47510 chr2:19498590-19498602 2 546::160 572::108 593::810 607::808 0.366147586 1 A3SS

AT3G22600 chr3:8006988-8006993 2 26::631 0::460 350::650 298::493 0.367700955 1 A3SS

AT5G64050 chr5:25632077-25632087 5 240::9 227::27 49::280 58::276 0.367819871 1 A3SS

AT2G30170 chr2:12880680-12880688 5 6::279 27::325 204::298 273::402 0.367851849 1 A3SS

AT4G39260 chr4:18274320-18274393 2 8::6733 22::12491 3438::4142 6437::7578 0.368525386 1 IR

AT1G33265 chr1:12066990-12067054 2 21::465 48::503 223::244 258::286 0.368847476 1 Cassette

AT2G26430 chr2:11245268-11245353 2 235::5 169::32 23::237 34::237 0.368950601 1 IR

AT3G22170 chr3:7822701-7822707 6 30::161 24::212 156::210 192::255 0.369015556 1 A3SS

AT3G07600 chr3:2423454-2423526 4 320::56 324::102 39::372 73::400 0.369095359 1 Cassette

AT2G36810 chr2:15437863-15437871 7 9::17 10::8 19::36 11::26 0.369924267 1 A3SS

AT5G47900 chr5:19393812-19393820 9 24::33 49::24 37::80 41::78 0.370180698 1 A3SS

AT4G02120 chr4:943015-943022 15 149::19 206::10 88::141 124::214 0.371744902 1 A3SS

AT5G04920 chr5:1439804-1439809 6 19::186 44::295 137::224 188::294 0.37232905 1 A3SS

AT5G62760 chr5:25207372-25207478 6 13::17 6::28 9::32 15::31 0.373303411 1 Cassette

AT2G47640 chr2:19537255-19537260 1 306::95 383::128 260::133 339::174 0.373770114 1 A5SS

AT4G32570 chr4:15717244-15717500 3 38::984 17::1011 558::624 588::581 0.374400503 1 Cassette

AT4G17680 chr4:9843750-9843830 2 31::18 30::71 12::55 38::87 0.374723012 1 IR

AT5G63310 chr5:25373130-25373216 3 27::3351 9::3742 1481::1936 1741::2014 0.376514747 1 Cassette

AT1G08465 chr1:2678679-2678694 5 232::18 262::5 191::270 218::296 0.377516591 1 A3SS

AT4G39270 chr4:18279088-18279163 3 46::121 49::54 65::92 31::98 0.377652304 1 IR

AT3G28956 chr3:10981462-10981593 2 12::24 5::69 26::29 42::38 0.378392021 1 Cassette

AT5G49555 chr5:20110125-20110286 2 25::195 0::150 92::137 76::99 0.378907485 1 Cassette

AT5G01580 chr5:223340-223399 3 6::5 8::15 8::28 24::24 0.379426255 1 A3SS

AT3G14930 chr3:5022583-5022666 6 474::933 740::1083 560::760 657::1030 0.379945148 1 IR

AT2G15530 chr2:6773964-6773983 3 7::19 21::10 19::24 20::37 0.380130528 1 A5SS

AT5G25060 chr5:8639442-8639487 3 6::67 16::55 31::47 28::38 0.380453742 1 Cassette

AT3G48050 chr3:17733983-17734064 4 195::118 328::109 81::176 88::264 0.380662746 1 IR

AT5G52070 chr5:21163579-21163648 3 51::20 30::25 13::85 13::65 0.38172883 1 Cassette

AT1G06040 chr1:1828904-1828987 2 4842::116 5005::238 408::3195 485::3415 0.38234873 1 IR

AT1G71810 chr1:27006135-27006155 7 46::46 31::62 44::73 58::73 0.382710223 1 A3SS

AT5G01510 chr5:202490-202523 4 16::10 5::18 15::32 31::47 0.382910058 1 A5SS

AT3G62190 chr3:23021480-23021497 2 513::13 513::8 441::423 436::497 0.382955367 1 A3SS

AT1G77350 chr1:29070482-29070493 2 231::52 282::33 241::269 294::301 0.38362711 1 A3SS

AT1G71696 chr1:26967556-26967699 3 13::44 11::25 14::32 10::30 0.384729825 1 IR

AT3G26890 chr3:9911368-9911415 2 2::61 14::29 23::36 36::38 0.38481594 1 A5SS

AT4G04890 chr4:2481289-2481369 1-2 111::11 68::12 10::318 8::322 0.385965178 1 AltStart

AT4G21910 chr4:11628921-11629001 2 136::156 52::148 191::261 186::246 0.387264809 1 AltStart

AT4G26480 chr4:13374133-13374272 3 82::868 125::797 454::552 412::584 0.387393557 1 Cassette

AT3G17650 chr3:6035620-6035668 5 48::187 56::176 67::315 113::362 0.387438872 1 A3SS

AT5G64816 chr5:25913433-25913457 2 108::201 59::216 153::291 128::346 0.38748674 1 A3SS

AT5G20020 chr5:6763404-6763412 3 15::1749 1::1683 1243::1547 1256::1608 0.387593775 1 A5SS

AT2G05440 chr2:1993624-1993671 2 17::69 3::74 15047::16379 17148::19434 0.387665638 1 A5SS

AT3G43740 chr3:15644750-15644893 3 22::498 2::504 304::320 298::319 0.388633506 1 Cassette

AT5G35180 chr5:13430158-13430224 18 15::241 8::393 120::176 180::218 0.388704336 1 Cassette

AT4G17300 chr4:9683610-9683617 9 11::190 36::205 109::167 116::210 0.3888413 1 A5SS

AT1G80940 chr1:30411706-30411749 3 21::343 2::433 111::178 98::214 0.388909425 1 A3SS

AT3G53830 chr3:19941685-19941694 5 120::12 73::19 104::137 84::170 0.389209739 1 A3SS

AT5G37510 chr5:14900348-14900435 5 0::2065 0::2685 1182::1123 1571::1268 0.389278513 1 IR

AT2G01220 chr2:125578-125592 10 9::168 30::284 55::266 101::385 0.389318098 1 A3SS

AT5G35330 chr5:13525621-13525642 1 96::84 75::115 122::117 116::123 0.389414379 1 A5SS

AT1G28290 chr1:9890397-9890491 1 638::82 476::39 1490::1999 1385::2165 0.390736692 1 Cassette

AT4G21060 chr4:11241844-11242088 2 0::24 0::34 18::25 25::22 0.392370117 1 IR

AT3G16470 chr3:5597269-5597431 4 11::724 10::593 815::1297 637::1233 0.392529603 1 Cassette

AT5G67540 chr5:26945699-26945782 2 19::0 13::7 4::59 12::62 0.392784097 1 A3SS

AT5G22840 chr5:7629036-7629053 5 252::20 208::33 77::232 79::225 0.393346644 1 A3SS

AT2G38710 chr2:16186823-16187119 1 282::6 183::22 12::436 12::348 0.393460312 1 IR

AT1G55680 chr1:20807712-20807731 1 21::362 6::240 25::240 13::149 0.393639409 1 A5SS

AT4G13575 chr4:7893146-7893189 4 85::138 211::210 153::253 204::428 0.393807473 1 A3SS

AT4G11980 chr4:7184839-7184846 4 21::81 14::72 69::98 66::126 0.39406304 1 A3SS

AT4G13590 chr4:7902264-7902274 5 32::591 50::539 542::617 500::596 0.394179319 1 A3SS

AT5G11860 chr5:3823265-3823402 4 9::182 16::166 155::156 139::183 0.394220822 1 A3SS

AT2G41600 chr2:17345988-17345993 2 29::164 18::265 140::214 226::269 0.394230757 1 A5SS

AT5G01920 chr5:360520-360528 4 1500::22 1311::47 822::665 736::729 0.394311002 1 A3SS

AT1G50440 chr1:18686061-18686144 2 13::23 48::12 47::127 103::195 0.39493626 1 A3SS

AT4G31115 chr4:15130942-15130947 3 20::130 38::113 60::173 61::185 0.395880968 1 A3SS

AT4G10060 chr4:6291874-6291885 8 135::8 53::12 131::177 82::109 0.396702739 1 A3SS

AT5G66810 chr5:26673993-26674038 4 22::39 5::33 30::79 8::56 0.397708861 1 A3SS

AT1G21780 chr1:7651926-7651998 1 1204::130 1521::254 163::620 269::750 0.397808165 1 IR

AT3G11470 chr3:3611402-3611407 4 50::41 25::25 30::88 16::49 0.399582813 1 A3SS

AT2G10940 chr2:4310782-4311137 1 9818::72 12269::155 181::10158 239::10298 0.401152628 1 IR

AT5G46470 chr5:18844142-18844225 2 4::30 23::46 15::36 32::46 0.401400561 1 IR

AT4G21990 chr4:11657597-11657750 5 0::1736 0::1522 1023::643 869::645 0.401701275 1 IR

AT4G34020 chr4:16299998-16300011 4 104::44 57::70 74::176 79::155 0.401902799 1 A3SS

AT5G14550 chr5:4691190-4691216 11 149::31 179::78 1716::2005 1300::1571 0.401981944 1 A3SS

AT4G35090 chr4:16700996-16701111 7 21032::306 22632::473 1471::6247 1631::6603 0.402304017 1 IR

AT3G61010 chr3:22573610-22573637 6 37::29 70::36 44::66 76::95 0.402468153 1 A5SS

AT3G07510 chr3:2394568-2394574 1 72::130 51::139 124::114 110::101 0.402894823 1 A5SS

AT1G28320 chr1:9922308-9922321 11 104::9 50::25 47::99 76::146 0.402944023 1 A3SS

AT4G36195 chr4:17129672-17129705 13 649::27 960::16 615::293 948::426 0.403048925 1 A3SS

AT3G10270 chr3:3179088-3179229 2 29::153 9::177 89::62 95::64 0.403101046 1 Cassette

AT3G55020 chr3:20389482-20389508 17 19::243 17::414 260::318 432::510 0.40327708 1 A3SS

AT3G12587 chr3:3995343-3995423 1-2 722::11 856::4 11::346 4::393 0.403575988 1 AltStart

AT1G20880 chr1:7263560-7263616 3-4 71::7 49::14 5::86 18::71 0.404375004 1 Cassette

AT1G31600 chr1:11314437-11314443 4 18::70 15::152 70::87 140::171 0.405258402 1 A3SS

AT1G79040 chr1:29736166-29736210 1 16::3 10::23 112689::58578 101804::49500 0.405290245 1 A5SS

AT2G41070 chr2:17131096-17131157 1 24::7 43::12 31::31 37::60 0.405343364 1 A5SS

AT1G14900 chr1:5139182-5139195 2 9::1310 7::976 1268::1208 978::1117 0.406272254 1 A3SS

AT3G63510 chr3:23451725-23451730 7 12::76 11::124 49::91 83::184 0.406382911 1 A3SS

AT1G65270 chr1:24244536-24244795 8 87::354 202::393 150::326 158::445 0.407167271 1 IR

AT5G47455 chr5:19251658-19251704 3 25::199 74::278 134::109 178::191 0.407436668 1 Cassette

AT1G54370 chr1:20294825-20294885 13 11::207 11::194 133::123 137::178 0.407652243 1 Cassette

AT4G13810 chr4:8006952-8007057 1 41::21 40::11 13::46 12::31 0.407743075 1 IR

AT2G21540 chr2:9224154-9224337 1 27::0 28::6 14::3 14::5 0.407847249 1 A5SS

AT1G71720 chr1:26984915-26984941 3 218::26 203::52 71::209 84::202 0.40786855 1 A5SS

AT4G37550 chr4:17645228-17645342 5 378::41 468::74 44::469 62::576 0.408517219 1 IR

AT4G01050 chr4:457756-457808 7 324::10481 0::10811 5346::5356 5526::5658 0.408526988 1 IR

AT3G07300 chr3:2326719-2326725 4 60::99 26::123 44::182 40::161 0.408921371 1 A5SS

AT1G15110 chr1:5200586-5200602 7 21::49 13::77 66::82 95::96 0.409107966 1 A5SS

AT1G21310 chr1:7454687-7454697 1 0::39 98::33 1684::1573 1501::1141 0.409282528 1 A5SS

AT1G80150 chr1:30149665-30149761 1 6::46 11::103 19::32 53::46 0.409632131 1 IR

AT1G80325 chr1:30197559-30197594 2 11::7 8::35 15::26 52::43 0.410632477 1 A3SS

AT4G02120 chr4:943579-943627 18 15::339 4::482 166::188 267::254 0.410990215 1 Cassette

AT1G53785 chr1:20077798-20077988 2 49::76 62::46 48::174 43::196 0.411020802 1 AltEnd

AT5G54840 chr5:22276994-22277016 5 13::81 23::82 78::105 79::131 0.411208358 1 A3SS

AT5G61310 chr5:24653752-24653761 2 236::185 253::128 331::404 301::463 0.411764562 1 A3SS

AT5G36170 chr5:14236975-14236980 5 294::44 266::77 249::374 266::404 0.4118132 1 A3SS

AT2G23985 chr2:10205450-10205462 2 57::39 60::10 58::95 57::70 0.413101843 1 A3SS

AT5G53550 chr5:21757040-21757056 5 4::186 13::193 177::215 180::237 0.415001665 1 A5SS

AT3G26950 chr3:9942827-9942837 2 18::89 47::87 54::147 83::149 0.415397153 1 A5SS

AT4G05400 chr4:2742158-2742199 2 125::19 176::53 15::166 28::210 0.416166466 1 Cassette

AT1G55340 chr1:20652902-20652932 4 127::14 129::50 184::222 174::246 0.416420194 1 A3SS

AT5G20990 chr5:7132245-7132325 3-4 78::1 106::21 6::156 31::199 0.416437345 1 AltStart

AT1G65430 chr1:24302698-24302726 11 5::122 21::108 23::121 37::119 0.417064268 1 A5SS

AT2G30600 chr2:13037052-13037114 2 8::9 5::9 14::71 9::126 0.41818129 1 AltStart

AT3G62750 chr3:23214927-23214944 4 10::110 13::134 78::100 82::112 0.418280066 1 A5SS

AT3G20240 chr3:7058137-7058142 4 132::23 141::6 93::137 96::176 0.418639186 1 A3SS

AT5G39250 chr5:15724847-15724903 2 35::24 58::0 29::63 58::90 0.418685489 1 A3SS

AT1G02850 chr1:631858-632009 7 132::68 105::92 43::195 60::129 0.418694041 1 IR

AT1G63800 chr1:23668218-23668226 5 293::51 411::60 112::416 150::510 0.418868984 1 A3SS

AT2G36670 chr2:15367400-15367414 2 18::56 19::62 22::66 29::71 0.420230469 1 Cassette

AT5G46730 chr5:18964588-18964721 1 0::317 0::343 165::356 180::348 0.420852797 1 IR

AT3G12130 chr3:3865587-3865647 2 89::726 52::758 367::300 360::326 0.421022474 1 Cassette

AT2G32010 chr2:13628603-13628637 11 41::8 68::0 56::79 74::81 0.421575818 1 A3SS

AT2G47940 chr2:19620115-19620123 11 37::707 62::682 560::735 535::701 0.422457734 1 A3SS

AT1G07120 chr1:2185399-2185436 3 13::23 27::13 26::39 19::34 0.42278141 1 Cassette

AT2G05520 chr2:2026646-2026649 2-3 501::902 877::1085 7257::9534 9106::13635 0.423144206 1 Cassette

AT5G13280 chr5:4251402-4251416 7 246::6 294::24 71::346 99::374 0.426088612 1 A5SS

AT5G38290 chr5:15304078-15304083 4 8::143 25::117 62::146 69::154 0.426193734 1 A3SS

AT3G23890 chr3:8630083-8630187 17 9::184 8::158 89::85 81::85 0.426478521 1 Cassette

AT3G56040 chr3:20795552-20795572 6 9::413 13::438 363::493 409::551 0.427137227 1 A3SS

AT5G58200 chr5:23549903-23550032 3 109::101 126::209 58::188 93::265 0.42805105 1 IR

AT4G04850 chr4:2455399-2455431 10 3::435 19::386 58::469 66::423 0.428485003 1 A3SS

AT3G11820 chr3:3730848-3730966 1 77::134 88::113 77::205 58::225 0.428868742 1 IR

AT1G09995 chr1:3262908-3263018 2 67::15 85::2 26::95 17::119 0.428933236 1 A3SS

AT4G16765 chr4:9430388-9430471 8 67::23 85::29 14::53 24::66 0.429709675 1 IR

AT4G00810 chr4:347083-347169 1 409::941 463::862 258::217 273::204 0.429882236 1 A5SS

AT5G21170 chr5:7207224-7207334 2 403::35 354::9 40::363 16::287 0.430791345 1 Cassette

AT5G10690 chr5:3376598-3376648 3 182::14 150::44 40::280 58::255 0.431297761 1 A3SS

AT1G78870 chr1:29651302-29651316 4 14::410 6::532 122::383 133::475 0.432267196 1 A3SS

AT5G27360 chr5:9661658-9661673 16 26::107 16::151 109::145 133::160 0.433207136 1 A5SS

AT4G24740 chr4:12756745-12756934 2 68::288 68::414 147::142 228::198 0.433804063 1 Cassette

AT3G07550 chr3:2409820-2409931 1 11::18 16::32 14::56 19::69 0.434094919 1 IR

AT4G22570 chr4:11883729-11883809 2-3 1314::20 1087::2 26::969 12::821 0.434096871 1 AltEnd

AT3G56240 chr3:20863537-20863580 3 41::32783 84::32754 18584::9585 18604::9115 0.434385575 1 IR

AT1G78140 chr1:29402592-29402706 5 338::24 364::6 309::288 284::262 0.435243634 1 A5SS

AT5G18100 chr5:5988423-5988464 6 517::1 540::0 14::557 56::606 0.435546589 1 AltEnd

AT2G16930 chr2:7341210-7341247 1 468::20 480::34 42::248 84::260 0.436132638 1 A5SS

AT1G18335 chr1:6309954-6309961 5 4::20 18::41 4::22 14::69 0.436199639 1 A5SS

AT3G17040 chr3:5812397-5812500 1 31::619 65::496 355::387 278::344 0.437007949 1 IR

AT1G60270 chr1:22224054-22224061 2 1::47 24::52 52::55 82::81 0.437097114 1 A3SS

AT3G53570 chr3:19864153-19864363 2 25::18 14::40 10::46 26::51 0.437238469 1 IR

AT1G49975 chr1:18505297-18505597 3 16::721 0::958 279::573 357::623 0.437504002 1 AltEnd

AT5G28020 chr5:10026746-10026755 9 33::720 66::1169 280::687 456::1136 0.438959616 1 A5SS

AT5G16520 chr5:5395480-5395515 6 12::40 4::71 48::57 84::105 0.439077904 1 A3SS

AT4G24930 chr4:12821402-12821527 5 27::674 3::723 334::480 380::524 0.439080701 1 IR

AT4G27760 chr4:13845912-13845995 5 209::11 246::15 32::253 52::285 0.439112984 1 A5SS

AT1G25420 chr1:8916821-8916936 4 5::225 21::181 222::203 199::209 0.439422191 1 A5SS

AT1G80780 chr1:30358295-30358379 1 449::425 414::609 252::778 341::920 0.439955192 1 IR

AT2G41060 chr2:17131016-17131096 4 27::24 11::43 55::98 89::129 0.439996139 1 AltStart

AT5G50665 chr5:20613835-20613907 1 17::54 49::44 3::3 3::3 0.441116345 1 A5SS

AT4G26700 chr4:13466904-13466928 13 119::12 198::4 43::155 73::239 0.441642752 1 A5SS

AT4G14965 chr4:8552747-8552769 6 34::276 18::305 240::267 259::271 0.44201434 1 A5SS

AT4G35410 chr4:16833428-16833540 4 104::26 113::65 25::132 40::168 0.442179653 1 IR

AT1G43890 chr1:16646906-16646950 2 309::15 337::4 346::334 359::327 0.442578555 1 A3SS

AT5G53360 chr5:21648478-21648820 2 45::12 42::44 14::44 38::59 0.443585283 1 Cassette

AT5G37370 chr5:14815172-14815283 4 110::36 97::56 45::152 63::146 0.44410718 1 Cassette

AT5G19030 chr5:6357584-6357598 4 18::156 42::137 138::62 126::61 0.444114509 1 A3SS

AT5G65050 chr5:25985089-25985122 4 45::37 59::17 95::169 162::233 0.446165218 1 A3SS

AT1G70410 chr1:26536604-26536926 3 1554::201 956::179 28::1632 24::1418 0.447059243 1 AltStart

AT2G26210 chr2:11158314-11158371 4 15::889 2::907 435::404 455::416 0.447394879 1 Cassette

AT1G76730 chr1:28804138-28804155 2 268::53 247::64 258::277 255::295 0.447576875 1 A5SS

AT3G59280 chr3:21909594-21909620 3 18::631 11::650 589::629 613::608 0.44763046 1 A3SS

AT5G46390 chr5:18818500-18818516 8 82::62 84::97 79::158 99::188 0.448083576 1 A5SS

AT5G44920 chr5:18139730-18140183 3 41::77 52::59 71::68 82::65 0.448539636 1 AltEnd

AT5G21930 chr5:7244650-7244657 5 50::80 75::82 45::156 55::175 0.44901215 1 A3SS

AT2G43810 chr2:18149494-18149574 2 447::6 415::20 6::320 20::366 0.449069424 1 AltStart

AT2G24250 chr2:10314568-10314591 2 1::9 4::12 14::32 27::40 0.449333317 1 A3SS

AT5G47455 chr5:19251935-19252204 4 62::30 93::93 33::20 45::52 0.449395853 1 A3SS

AT4G01940 chr4:842015-842097 3 183::216 266::352 116::557 204::727 0.449945961 1 IR

AT5G46420 chr5:18831293-18831298 5 271::19 254::55 182::310 201::309 0.45011332 1 A5SS

AT2G05380 chr2:1967610-1967674 2 17::23596 22::33753 11719::11304 16882::17002 0.451594814 1 IR

AT3G61080 chr3:22608166-22608176 6 331::13 275::26 344::304 304::266 0.451774703 1 A3SS

AT5G63460 chr5:25414413-25414448 3 216::21 262::29 40::237 56::295 0.452100601 1 Cassette

AT2G37050 chr2:15572537-15572613 12 226::10 277::35 18::258 33::343 0.45230464 1 IR

AT5G58220 chr5:23554940-23554979 4 264::72 266::134 127::390 183::438 0.452449802 1 A3SS

AT2G22250 chr2:9460285-9460338 2 6::10 17::0 150::193 129::146 0.45302138 1 A3SS

AT2G32690 chr2:13864136-13864167 1 0::57 0::88 33::3463 46::2663 0.453095587 1 IR

AT1G19396 chr1:6710115-6710345 2 46::30 79::21 32::47 33::85 0.453231769 1 AltEnd

AT5G05610 chr5:1677223-1677319 5 171::136 284::122 87::276 75::366 0.453268095 1 IR

AT5G44030 chr5:17716300-17716351 6 3::32 12::9 40::38 27::53 0.454104814 1 A5SS

AT1G53230 chr1:19850202-19850221 1 220::20 323::22 220::317 333::352 0.454415869 1 A5SS

AT5G08130 chr5:2608370-2608382 5 8::197 5::197 201::230 202::242 0.454563178 1 A3SS

AT2G27760 chr2:11826410-11826415 7 16::7 14::12 22::27 20::33 0.454582041 1 A3SS

AT2G39550 chr2:16503281-16503289 8 45::66 70::79 50::133 73::205 0.455287371 1 A3SS

AT1G49510 chr1:18328276-18328291 4 13::351 6::539 37::190 22::253 0.455685958 1 A3SS

AT3G60240 chr3:22262372-22262378 3 6::68 13::36 47::80 35::59 0.455721738 1 A3SS

AT2G30120 chr2:12861200-12861336 1 59::11 31::38 12::54 23::71 0.456615951 1 IR

AT2G17150 chr2:7469148-7469222 4 16::18 4::24 39::51 37::72 0.456741218 1 A3SS

AT1G06570 chr1:2012297-2012304 1 477::2 497::3 485::563 527::468 0.457074965 1 A5SS

AT5G56500 chr5:22873871-22873908 1 103::24 84::10 36::79 21::62 0.4576066 1 A5SS

AT5G57150 chr5:23154539-23154696 6 99::9 203::3 13::75 4::87 0.458442932 1 AltEnd

AT5G61910 chr5:24862440-24862555 4 2::56 13::33 7::53 16::52 0.460074813 1 A5SS

AT5G22700 chr5:7546405-7546528 2 34::15 94::8 53::67 127::92 0.460141297 1 A5SS

AT5G26240 chr5:9194101-9194106 21 66::60 113::109 35::132 59::218 0.460880263 1 A5SS

AT2G21660 chr2:9265649-9265740 2 2::12308 76::18837 7033::2805 10817::4230 0.461199614 1 IR

AT4G04880 chr4:2465769-2465779 8 67::11 56::19 40::72 55::112 0.46182723 1 A3SS

AT4G33520 chr4:16120502-16120935 4 28::671 19::700 263::225 359::216 0.462130778 1 AltEnd

AT5G55490 chr5:22479175-22479319 5 18::5 6::16 25::38 46::47 0.462197089 1 Cassette

AT3G54620 chr3:20218536-20218609 6 23::170 13::256 209::268 270::334 0.462235615 1 A3SS

AT1G65230 chr1:24230040-24230049 3 69::606 92::560 357::691 372::747 0.462295395 1 A3SS

AT1G79880 chr1:30048049-30048125 2 54::39 26::62 27::85 39::78 0.463042864 1 IR

AT1G65980 chr1:24559649-24559702 3 0::7107 0::8038 3736::2769 4163::3366 0.463351471 1 IR

AT5G06130 chr5:1854168-1854191 5 683::13 847::3 671::618 815::710 0.464178573 1 A5SS

AT4G25450 chr4:13010287-13010373 14 256::9 362::42 26::286 54::366 0.464868017 1 IR

AT3G43540 chr3:15432198-15432203 6 1::328 20::361 168::269 190::338 0.464991424 1 A5SS

AT4G27870 chr4:13879605-13879664 1-2 97::36 73::17 24::120 10::90 0.465103693 1 Cassette

AT3G16260 chr3:5510366-5510394 5 42::14 41::9 22::64 23::68 0.465556182 1 A5SS

AT5G64000 chr5:25617066-25617095 3 3::35 14::62 38::41 72::80 0.465709753 1 A5SS

AT5G60170 chr5:24229700-24229706 9 64::7 50::19 70::103 64::152 0.466120156 1 A3SS

AT1G72050 chr1:27115642-27115816 3 21::210 9::244 112::135 134::194 0.466211582 1 Cassette

AT3G51950 chr3:19278151-19278240 9 693::159 926::138 99::358 88::462 0.466497147 1 IR

AT3G13882 chr3:4574622-4574681 1 0::2036 0::1712 1075::815 891::803 0.467619949 1 IR

AT3G26510 chr3:9711400-9711459 3 0::28 1::53 72::1755 85::767 0.467804252 1 Cassette

AT4G32320 chr4:15604423-15604445 7 8::299 19::431 52::276 80::392 0.467904021 1 A3SS

AT4G32320 chr4:15603967-15604013 4 139::17 219::27 39::167 49::223 0.467981444 1 A5SS

AT3G29160 chr3:11129794-11129799 10 16::290 29::315 194::311 224::365 0.468728015 1 A3SS

AT2G20585 chr2:8866348-8866403 3 285::106 362::130 84::231 108::283 0.469090027 1 Cassette

AT5G17070 chr5:5615176-5615186 3 8::148 22::110 54::149 61::121 0.469196382 1 A5SS

AT2G31290 chr2:13344056-13344130 1 13::37 1::12 19::37 7::38 0.469690768 1 IR

AT2G46500 chr2:19088710-19088853 1 49::135 45::56 77::213 52::214 0.469846333 1 IR

AT5G08185 chr5:2634974-2635046 3 54::39 108::104 39::122 90::238 0.469985594 1 Cassette

AT2G23790 chr2:10126150-10126660 2 80::16 76::65 58::125 114::225 0.470942391 1 A3SS

AT5G08650 chr5:2808818-2808826 14 702::30 685::47 368::656 359::689 0.470997907 1 A5SS

AT4G36690 chr4:17296388-17296445 4-5 540::18 588::27 15::492 25::563 0.47130135 1 Cassette

AT1G08110 chr1:2535585-2535675 1 524::17 407::35 28::546 32::468 0.471968476 1 IR

AT2G31900 chr2:13560991-13561045 39 7::13 17::18 16::29 24::42 0.472395291 1 A3SS

AT2G38995 chr2:16282631-16283200 2 19::0 5::5 3::20 9::15 0.472396054 1 Cassette

AT5G06280 chr5:1918025-1918101 2 19::68 36::85 81::36 115::68 0.472409885 1 A3SS

AT1G18735 chr1:6460767-6460776 3 1026::23 1111::27 735::876 795::964 0.473555518 1 A5SS

AT3G28100 chr3:10456625-10456645 3 102::31 98::46 105::141 102::155 0.473629581 1 A3SS

AT3G54500 chr3:20178804-20178941 1 181::406 216::584 196::540 311::719 0.473729472 1 IR

AT1G54350 chr1:20288396-20288541 5 197::9 154::20 21::277 34::206 0.473755036 1 A5SS

AT1G19000 chr1:6561142-6561321 3 417::1489 440::1610 941::2421 990::2310 0.474992727 1 IR

AT5G14920 chr5:4827209-4827309 3 10::692 27::1027 366::314 545::603 0.475167251 1 IR

AT1G34200 chr1:12456078-12456260 1 14::37 2::75 42::75 60::72 0.475287952 1 A5SS

AT1G42430 chr1:15892643-15892682 4 15::150 3::171 21::156 15::197 0.475916723 1 A3SS

AT4G14210 chr4:8194804-8194817 2 303::25 211::2 103::403 61::269 0.47644663 1 A3SS

AT2G41060 chr2:17130030-17130110 4 27::17 11::25 38::98 89::129 0.476593832 1 AltEnd

AT5G03240 chr5:772520-772674 2 14::26 33::0 4772::3983 5435::4667 0.476842542 1 A5SS

AT3G47560 chr3:17525952-17526031 4 211::108 269::75 67::242 56::294 0.477775926 1 IR

AT3G62940 chr3:23264238-23264254 2 63::28 36::29 68::171 64::151 0.478936834 1 A3SS

AT5G14550 chr5:4691464-4691473 9 29::72 52::18 2346::816 2018::730 0.479415366 1 A5SS

AT1G10590 chr1:3503004-3503049 2 1095::4 963::20 124::1767 131::1720 0.481400007 1 A3SS

AT1G20890 chr1:7266737-7266768 3 72::30 84::18 82::93 89::99 0.481676655 1 A5SS

AT1G35340 chr1:12979228-12979264 6 26::307 12::424 65::388 71::534 0.482581264 1 A3SS

AT3G59780 chr3:22088408-22088418 5 331::632 461::663 318::811 431::1068 0.483545199 1 A3SS

AT1G78690 chr1:29596983-29597210 2 16::85 1::117 61::60 67::73 0.483853075 1 Cassette

AT1G28960 chr1:10109862-10109926 5 108::133 104::96 118::231 141::258 0.484795686 1 A5SS

AT4G39280 chr4:18281444-18281691 18 17::171 22::179 101::220 140::218 0.48518067 1 AltEnd

AT4G39260 chr4:18274314-18274320 3 17::8 26::22 2685::2796 5150::5322 0.486247734 1 A3SS

AT3G26690 chr3:9805408-9805473 1 16::107 41::97 63::46 56::63 0.486256206 1 IR

AT3G52060 chr3:19310879-19310967 1 1602::111 1449::44 155::1001 105::718 0.486318053 1 IR

AT3G51880 chr3:19247436-19247457 8 23::503 47::431 97::464 105::426 0.486598672 1 A5SS

AT1G73930 chr1:27797680-27797864 1 4::156 11::73 70::98 42::114 0.487360534 1 IR

AT2G38330 chr2:16065926-16065943 6 27::70 47::53 92::187 76::186 0.487447101 1 A5SS

AT1G10865 chr1:3616081-3616089 1 8::349 11::405 164::160 201::192 0.487847718 1 A5SS

AT5G05740 chr5:1725920-1726008 4 306::25 248::35 32::339 30::311 0.487883561 1 IR

AT2G04690 chr2:1646031-1646066 5 191::107 332::171 211::374 300::507 0.487946957 1 A5SS

AT1G30690 chr1:10887827-10887850 1 163::32 130::10 44::70 23::49 0.488190054 1 A5SS

AT2G13975 chr2:5865236-5865319 1 183::208 219::155 145::232 110::256 0.488295141 1 IR

AT1G72560 chr1:27324728-27324817 14 53::46 64::131 27::43 62::87 0.48870455 1 IR

AT1G31500 chr1:11276166-11276181 2 26::22 35::21 30::51 31::63 0.48884716 1 A3SS

AT4G30000 chr4:14670168-14670321 3 1::42 22::46 14::51 20::91 0.489233624 1 IR

AT1G20540 chr1:7114021-7114113 7 134::12 242::5 34::165 36::257 0.489592308 1 A5SS

AT3G62830 chr3:23232400-23232510 1 0::280 6::191 223::334 136::302 0.489861869 1 IR

AT2G04800 chr2:1687722-1687789 1 8::107 27::103 22::97 44::97 0.490989875 1 A5SS

AT3G11560 chr3:3641937-3641965 6 6::208 10::172 38::249 38::201 0.491556188 1 A3SS

AT4G19070 chr4:10447116-10447224 3 35::318 8::221 157::169 109::110 0.49205928 1 IR

AT1G17370 chr1:5954218-5954279 2 17::1189 32::1191 609::341 610::327 0.49231425 1 Cassette

AT3G22380 chr3:7918871-7918945 12 10::1294 17::1755 684::512 923::569 0.492791015 1 IR

AT1G43670 chr1:16469861-16469899 10 1020::21 1421::23 1043::1180 1427::1634 0.493070675 1 A3SS

AT3G12100 chr3:3855028-3855117 8 75::14 138::13 17::101 14::162 0.494372875 1 IR

AT1G77480 chr1:29114834-29114974 8 111::16 95::51 14::87 27::107 0.494786928 1 IR

AT1G60850 chr1:22400167-22400178 2 41::39 55::18 59::95 57::113 0.495601992 1 A3SS

AT4G26555 chr4:13405465-13405472 3 316::182 257::214 360::425 339::383 0.495758225 1 A3SS

AT2G44798 chr2:18468076-18468100 3 6::20 5::23 13::27 20::42 0.497027069 1 A5SS

AT2G13440 chr2:5597490-5597495 4 39::8 70::8 14::65 21::78 0.497158079 1 A3SS

AT3G10420 chr3:3241547-3241641 7 807::21 1012::81 57::1040 99::1331 0.498087315 1 IR

AT1G48490 chr1:17928633-17928719 1 0::23 0::24 15::27 19::21 0.498332871 1 IR

AT3G05345 chr3:1523758-1523860 1 12::201 29::205 100::231 108::222 0.498855309 1 IR

AT1G21410 chr1:7498143-7498223 2-3 177::20 123::0 4::278 3::206 0.498995262 1 AltEnd

AT3G01850 chr3:301494-301498 2 28::0 25::18 4::215 20::262 0.499070085 1 Cassette

AT4G00590 chr4:253426-253431 4 10::15 33::11 23::31 50::62 0.49931266 1 A3SS

AT1G70100 chr1:26403368-26403627 2 15::19 24::6 5::63 4::65 0.499818238 1 AltStart

AT5G66210 chr5:26457108-26457214 11 105::39 123::9 29::138 10::138 0.500421301 1 IR

AT2G33550 chr2:14210801-14210810 2 19::147 39::157 115::151 119::160 0.501204791 1 A3SS

AT5G27360 chr5:9661770-9661835 17 107::26 151::16 118::145 157::181 0.501264507 1 A3SS

AT3G14910 chr3:5016509-5016534 4 41::9 29::22 44::59 39::64 0.50144114 1 A3SS

AT3G61530 chr3:22772538-22772546 3 7::98 9::133 51::105 70::143 0.50186195 1 A5SS

AT4G24100 chr4:12516737-12516747 7 11::17 5::23 16::38 14::45 0.502042834 1 A3SS

AT4G09760 chr4:6149691-6149735 4 191::11 127::24 182::243 133::210 0.503046409 1 A5SS

AT4G26555 chr4:13405139-13405152 5 393::56 403::80 109::431 132::500 0.503211426 1 A3SS

AT2G46880 chr2:19265216-19265289 2 611::54 682::111 90::356 132::355 0.503382616 1 IR

AT3G17340 chr3:5921548-5921561 18 60::0 132::0 64::92 114::111 0.503404511 1 A5SS

AT2G17190 chr2:7478586-7478592 7 25::597 25::673 466::598 517::675 0.503432656 1 A3SS

AT2G46330 chr2:19018836-19018994 1 2554::182 2589::266 197::1554 230::1623 0.5039428 1 IR

AT1G32200 chr1:11602114-11602209 12 135::73 133::148 62::136 106::162 0.504417871 1 IR

AT1G53250 chr1:19858177-19858187 3 3::20 18::20 23::21 31::44 0.504709992 1 A5SS

AT1G19800 chr1:6846540-6846628 2 195::141 112::129 64::202 69::150 0.504909804 1 Cassette

AT5G37850 chr5:15065479-15065512 1 106::37 115::55 56::85 118::113 0.505367299 1 A5SS

AT1G09840 chr1:3199623-3199694 4 86::6 78::12 21::183 27::147 0.505392701 1 A3SS

AT1G66160 chr1:24637364-24637413 1 0::33 0::38 18::21 22::26 0.505545893 1 IR

AT5G24680 chr5:8454585-8454681 3 20::29 10::44 16::33 23::41 0.505981317 1 IR

AT2G31600 chr2:13449159-13449214 2 40::1 40::18 4::39 12::41 0.506460562 1 Cassette

AT3G03020 chr3:681529-681569 2 15::26 0::43 61::61 67::97 0.50683379 1 A3SS

AT4G20070 chr4:10863562-10863572 9 18::229 34::212 46::247 48::239 0.506846006 1 A3SS

AT5G04480 chr5:1273856-1273891 8 3::22 14::27 5::43 15::38 0.507262725 1 A5SS

AT1G54180 chr1:20228800-20228877 4 37::6 13::2 16::49 40::45 0.507691971 1 Cassette

AT5G08480 chr5:2744747-2744841 2 0::21 0::30 13::14 21::15 0.508402878 1 IR

AT1G22860 chr1:8086466-8086503 6 26::19 40::26 24::52 30::67 0.509278425 1 A5SS

AT4G23910 chr4:12427734-12428594 5 127::2 119::13 21::147 53::161 0.509308451 1 A5SS

AT2G05520 chr2:2026610-2026628 2 88::413 109::793 8711::15174 10618::18245 0.51014176 1 A5SS

AT4G21660 chr4:11508631-11508668 5-6 240::116 214::140 59::291 60::287 0.510276129 1 Cassette

AT1G78040 chr1:29345897-29345983 1 0::4685 0::4133 1884::4829 1631::4542 0.510389082 1 IR

AT5G24350 chr5:8311442-8311465 15 9::24 10::6 22::38 5::45 0.510456709 1 Cassette

AT1G27920 chr1:9729600-9729621 11 11::7 18::11 24::29 25::40 0.510688135 1 A3SS

AT4G36400 chr4:17198612-17198629 6 12::8 29::7 15::40 29::36 0.510960354 1 A3SS

AT1G45150 chr1:17080517-17080689 7 13::13 2::13 9::25 34::41 0.510964433 1 A3SS

AT4G00165 chr4:69370-69391 1 0::647 0::810 376::863 463::867 0.511220979 1 IR

AT5G27950 chr5:9985402-9985453 4 8::39 23::41 22::101 23::84 0.511399215 1 A3SS

AT3G01690 chr3:257040-257054 1 7::863 15::1147 938::840 1250::983 0.51179331 1 A5SS

AT3G59330 chr3:21927914-21927976 2 8::4 12::11 22::33 43::74 0.511982345 1 A3SS

AT1G33270 chr1:12068654-12068829 5 132::15 148::53 21::146 33::170 0.51315192 1 IR

AT1G34370 chr1:12551419-12551536 3 20::654 64::612 348::397 290::423 0.513349231 1 IR

AT2G02870 chr2:838290-838366 2 0::195 0::194 117::169 108::188 0.513517467 1 IR

AT1G61010 chr1:22474630-22474669 6 24::12 19::29 9::158 18::155 0.513939579 1 Cassette

AT2G28370 chr2:12133033-12133047 3 706::7 630::24 700::926 604::873 0.514012474 1 A3SS

AT1G22160 chr1:7823430-7823533 1 21::681 1::652 638::543 652::529 0.514160191 1 A5SS

AT1G21720 chr1:7627427-7627511 5 14::1482 12::1707 756::842 893::910 0.514238654 1 Cassette

AT1G57540 chr1:21311413-21311441 1 143::26 193::17 134::72 159::80 0.514265278 1 A5SS

AT3G15950 chr3:5398151-5398236 28 5::2613 13::3246 1402::1368 1710::1853 0.514287453 1 IR

AT3G01980 chr3:328025-328099 3 394::55 354::97 64::255 92::243 0.514438471 1 IR

AT5G66040 chr5:26410805-26410869 2 15775::194 13709::225 1899::13980 1695::12394 0.514877278 1 IR

AT1G61660 chr1:22755214-22755223 3 0::255 10::187 226::227 174::185 0.515093134 1 A3SS

AT4G24520 chr4:12663862-12663869 14 5::757 13::854 758::795 856::1009 0.515134912 1 A3SS

AT5G41140 chr5:16472117-16472138 7 3::23 3::46 26::31 49::37 0.51596628 1 A5SS

AT1G23960 chr1:8480017-8480201 1 265::8 264::37 18::215 26::194 0.516018948 1 IR

AT5G23450 chr5:7909050-7909126 2 15::155 6::169 89::190 97::165 0.516217997 1 IR

AT5G24670 chr5:8449653-8449690 7 30::139 32::265 141::188 268::314 0.516655236 1 A5SS

AT5G35840 chr5:14010167-14010203 2 54::14 52::55 66::106 70::152 0.516720939 1 A3SS

AT5G20450 chr5:6912811-6913127 1 6::19 10::37 7::29 17::40 0.517929398 1 IR

AT4G03415 chr4:1505524-1505692 3 29::99 3::117 160::270 180::248 0.518313683 1 A3SS

AT4G30900 chr4:15039566-15039642 1 24::11 25::37 10::42 25::51 0.518462381 1 IR

AT3G02460 chr3:507690-507696 8 89::9 98::30 103::121 111::131 0.519048142 1 A3SS

AT1G24793 chr1:8768280-8768302 3 46::52 50::35 8::46 3::3 0.520056994 1 A3SS

AT1G52500 chr1:19560835-19560892 3 22::72 17::103 38::311 50::439 0.520626882 1 Cassette

AT3G58710 chr3:21717777-21717785 4 45::2 21::9 50::65 42::64 0.520927295 1 A3SS

AT3G62750 chr3:23216663-23216724 13 182::18 313::9 203::126 318::197 0.521067208 1 A3SS

AT2G41840 chr2:17461317-17461326 1 6::6 317::9 12064::5588 11462::5375 0.521215202 1 A5SS

AT1G36310 chr1:13669542-13669625 1 69::48 66::18 35::252 19::303 0.521536332 1 IR

AT4G27760 chr4:13846034-13846080 6 209::11 246::18 229::253 284::331 0.521736369 1 A3SS

AT2G38040 chr2:15920848-15920856 12 229::867 255::1278 605::485 752::663 0.521938184 1 A3SS

AT5G42920 chr5:17207713-17207729 5 14::110 9::116 33::118 26::122 0.522193745 1 A5SS

AT1G25098 chr1:8812956-8813048 1 42::1817 8::2019 10::39 11::31 0.522215919 1 IR

AT1G34210 chr1:12460490-12460495 5 13::118 14::164 61::109 80::152 0.522764585 1 A3SS

AT1G31300 chr1:11193838-11193980 1 181::56 123::63 64::115 53::79 0.522798868 1 A5SS

AT4G15560 chr4:8884082-8884313 1 38::1981 4::1234 730::616 438::379 0.523481746 1 IR

AT1G79790 chr1:30017905-30017972 1 301::85 311::157 68::278 119::320 0.524083825 1 IR

AT1G15020 chr1:5175281-5175286 4 90::15 98::1 90::135 84::139 0.524304371 1 A3SS

AT3G01120 chr3:41482-41573 1 13::9762 25::9294 5360::4077 5121::3767 0.524307363 1 IR

AT1G74960 chr1:28152241-28152273 15 68::755 51::985 716::342 973::391 0.524344864 1 A3SS

AT1G23360 chr1:8296342-8296419 3 115::31 67::75 29::124 43::139 0.524407589 1 IR

AT5G20590 chr5:6965344-6965349 4 12::95 15::139 58::134 84::187 0.524996814 1 A3SS

AT2G05380 chr2:1967610-1967635 2 17::17 34::4 13705::17931 19301::24347 0.525316852 1 A5SS

AT3G19820 chr3:6882096-6882121 2 959::82 653::40 88::3099 70::2995 0.526084637 1 AltStart

AT5G14060 chr5:4538087-4538092 9 81::1206 108::1378 595::1416 672::1527 0.526495014 1 A5SS

AT1G04820 chr1:1357426-1357724 2 20::7473 0::6641 4106::4232 3539::3658 0.5266196 1 IR

AT4G12030 chr4:7211612-7211691 4 96::139 213::151 76::287 96::469 0.526737844 1 IR

AT4G12030 chr4:7211691-7211885 5 96::17 213::3 225::365 403::615 0.526804475 1 A3SS

AT3G46040 chr3:16914873-16914890 2 1748::23 1753::24 1649::1835 1679::1971 0.527003538 1 A3SS

AT2G21970 chr2:9355556-9355561 3 1021::9 1036::25 803::924 840::991 0.527462986 1 A3SS

AT5G19090 chr5:6388965-6389332 4 0::604 0::623 303::411 290::457 0.527686656 1 IR

AT5G26740 chr5:9292142-9292222 1 61::2 36::15 7::83 14::62 0.527951291 1 IR

AT3G55080 chr3:20414651-20414664 4 25::62 38::85 66::98 78::110 0.529110536 1 A3SS

AT5G18240 chr5:6029282-6029327 7 14::17 41::6 25::35 23::51 0.529417049 1 A3SS

AT2G21195 chr2:9083261-9083339 1 612::218 683::305 153::456 223::520 0.529731474 1 IR

AT2G47940 chr2:19620905-19620931 7 5::847 16::743 8::878 25::793 0.529928252 1 A5SS

AT5G67030 chr5:26755124-26755147 8 1991::8 1597::18 1871::1760 1498::1403 0.530095412 1 A5SS

AT4G29890 chr4:14610604-14610637 9 17::65 12::108 29::73 34::127 0.530428811 1 A3SS

AT4G38260 chr4:17938050-17938058 2 17::222 14::203 157::279 158::301 0.530653694 1 A3SS

AT5G09230 chr5:2871609-2871691 3 28::51 26::54 29::80 35::99 0.530672743 1 Cassette

AT3G59060 chr3:21829698-21829703 3 8::552 7::339 335::1167 205::886 0.530745033 1 A3SS

AT3G20930 chr3:7333427-7333442 8 10::137 10::153 24::175 21::182 0.530831711 1 A3SS

AT2G26800 chr2:11432202-11432313 2 245::117 151::108 174::503 174::353 0.530841912 1 A3SS

AT2G20550 chr2:8847154-8847252 1 13::28 20::16 23::45 14::46 0.531036916 1 IR

AT5G16480 chr5:5381829-5381886 3 13::240 6::261 128::147 117::167 0.531184932 1 Cassette

AT5G57360 chr5:23244215-23244324 2 0::776 0::1047 449::375 556::358 0.532331495 1 IR

AT2G35820 chr2:15051482-15051588 2 622::10 660::35 729::631 714::678 0.532504729 1 A3SS

AT1G73210 chr1:27529628-27529634 2 10::13 12::13 21::38 25::56 0.532513658 1 A3SS

AT1G25054 chr1:8798578-8798670 1 57::2366 87::2688 6::17 7::28 0.533062219 1 IR

AT4G02540 chr4:1118897-1119007 1 17::115 86::168 71::66 92::127 0.533170634 1 IR

AT3G16910 chr3:5773997-5774086 3 12::1784 3::1301 944::1107 685::965 0.533739737 1 IR

AT5G63370 chr5:25385077-25385151 2 41::376 60::345 213::138 187::183 0.533874665 1 IR

AT5G61420 chr5:24690582-24690663 1 797::34 896::75 71::752 99::869 0.533919137 1 IR

AT1G67600 chr1:25337008-25337025 3 113::3 160::13 21::135 42::207 0.533928426 1 A3SS

AT3G03560 chr3:855127-855140 8 13::26 11::54 17::36 23::71 0.534568395 1 A5SS

AT2G39470 chr2:16477002-16477016 5 1447::128 1325::120 1288::1748 1410::1982 0.534933691 1 A3SS

AT5G12860 chr5:4059850-4059931 3 0::3847 1::5455 2007::2683 2915::3451 0.535185998 1 IR

AT3G59950 chr3:22146796-22146891 2 27::128 4::134 70::123 73::126 0.536032245 1 IR

AT2G23680 chr2:10066360-10066453 1 25::0 9::10 3::22 6::21 0.536130097 1 IR

AT5G38895 chr5:15571937-15571954 3 32::9 19::21 39::60 25::49 0.536491494 1 A3SS

AT2G27730 chr2:11821652-11821732 1-2 1583::27 1948::20 30::1676 30::2080 0.536777798 1 AltStart

AT3G07180 chr3:2283341-2283471 8 0::385 0::292 202::150 154::165 0.536891922 1 Cassette

AT4G29890 chr4:14610510-14610551 8 17::65 12::109 67::78 112::114 0.537411677 1 A5SS

AT1G54355 chr1:20288461-20288541 4 192::9 152::20 17::322 28::348 0.537433434 1 AltEnd

AT2G27110 chr2:11580462-11580610 1 14::33 40::17 19::64 13::64 0.538069976 1 IR

AT3G13080 chr3:4196520-4196617 6 281::24 426::10 24::187 28::315 0.5385776 1 IR

AT3G17090 chr3:5829106-5829111 4 378::1 701::0 431::204 813::280 0.539161749 1 A3SS

AT5G54520 chr5:22148357-22148370 3 5::16 1::20 20::32 22::31 0.539385644 1 A3SS

AT2G03667 chr2:1115632-1115643 6 23::14 32::4 20::43 26::38 0.539397594 1 A3SS

AT5G40380 chr5:16154326-16154358 4 83::18 64::5 24::89 14::74 0.540125867 1 A5SS

AT3G14290 chr3:4765466-4765510 6 551::16 645::0 57::541 53::605 0.5402293 1 A5SS

AT1G78810 chr1:29628636-29628693 3 7::5 5::24 14::35 20::55 0.540856877 1 A5SS

AT4G24350 chr4:12610363-12610371 3 9::133 24::180 71::157 102::191 0.541093324 1 A3SS

AT1G12800 chr1:4364033-4364041 3 17::421 3::362 491::597 398::396 0.54193724 1 A3SS

AT5G10745 chr5:3396811-3397645 2 39::389 18::493 99::282 158::319 0.542087767 1 IR

AT1G11650 chr1:3916146-3916232 2-3 1585::148 1542::180 90::1791 120::1628 0.542168102 1 Cassette

AT3G01980 chr3:328848-328959 1 89::213 45::203 130::219 121::190 0.542422606 1 IR

AT2G43210 chr2:17960923-17960942 2 7::64 7::16 13::87 12::62 0.542870391 1 A3SS

AT3G59090 chr3:21842325-21842408 13 219::49 323::32 267::152 393::214 0.543013309 1 A3SS

AT1G25500 chr1:8956576-8956584 4 9::26 21::29 26::39 43::70 0.543039583 1 A5SS

AT5G10540 chr5:3333459-3334907 18 443::0 473::0 357::236 333::204 0.543123486 1 A3SS

AT5G22340 chr5:7395499-7395517 4 581::22 386::28 135::640 103::433 0.543427406 1 A5SS

AT4G10120 chr4:6315007-6315017 2 9::48 13::14 72::168 46::90 0.543727912 1 A3SS

AT1G62640 chr1:23192302-23192388 1 9::218 24::164 131::157 112::142 0.543846063 1 IR

AT2G04039 chr2:1333506-1333565 1 34::1015 13::1067 1190::621 1326::613 0.544276735 1 A5SS

AT4G29020 chr4:14305287-14305327 1 0::11711 179::9742 6092::2891 4968::2293 0.544501374 1 IR

AT3G19580 chr3:6803246-6803272 1 0::19 0::19 14::84 16::46 0.54513423 1 IR

AT1G15290 chr1:5258137-5258208 21 49::1477 121::1870 806::850 980::1184 0.546094286 1 IR

AT5G24340 chr5:8298181-8298196 3 13::18 8::28 15::37 11::63 0.546560014 1 A3SS

AT5G57960 chr5:23465807-23465827 2 104::4 90::14 107::105 99::95 0.546670103 1 A5SS

AT5G35180 chr5:13430626-13430632 19 136::16 222::5 161::172 226::217 0.546772414 1 A3SS

AT2G05440 chr2:1993671-1993723 2 69::689 74::945 7894::15734 8340::18325 0.546777166 1 A5SS

AT1G63250 chr1:23465023-23465042 4 5::10 14::5 16::31 12::34 0.546784501 1 A3SS

AT5G35210 chr5:13475970-13476227 10 28::15 58::13 132::227 167::314 0.547230539 1 A5SS

AT5G58950 chr5:23801433-23801441 4 1::1467 18::1591 1143::840 1275::1095 0.54828293 1 A3SS

AT5G47455 chr5:19251935-19251979 4 27::70 89::132 56::112 96::190 0.54835603 1 Cassette

AT2G02450 chr2:648666-648746 2 14::12 8::4 25::37 16::31 0.548434133 1 A3SS

AT4G31870 chr4:15410870-15410884 3 65::23 37::11 37::73 20::37 0.548701066 1 A5SS

AT4G34460 chr4:16479073-16479088 2 12::583 2::652 546::599 597::613 0.549318112 1 A3SS

AT2G14285 chr2:6053488-6053537 2 16::793 35::945 408::214 492::268 0.549504528 1 Cassette_multi

AT2G46225 chr2:18983309-18983403 5 255::13 245::28 15::238 24::263 0.549876032 1 IR

AT5G43710 chr5:17552693-17552775 16 8::36 9::98 11::64 22::118 0.549888252 1 A5SS

AT3G21610 chr3:7610197-7610205 2 168::44 210::38 133::250 147::261 0.550395304 1 A3SS

AT5G11380 chr5:3632581-3632653 8 1::114 20::113 149::135 139::156 0.55094585 1 A5SS

AT1G01540 chr1:197904-197974 5 356::32 382::72 62::456 77::537 0.551082502 1 IR

AT4G22350 chr4:11805926-11805964 4 20::5 35::28 4::6 19::8 0.551571046 1 A3SS

AT1G59610 chr1:21898930-21898960 16 12::147 6::153 33::168 38::198 0.551769316 1 A3SS

AT3G61420 chr3:22728733-22728759 14 35::34 54::61 58::104 79::155 0.551792254 1 A3SS

AT4G22120 chr4:11719399-11719462 1 14::34 1::22 26::13 5::10 0.552081487 1 A5SS

AT1G60530 chr1:22301012-22301109 2 29::22 65::8 14::64 11::98 0.552472772 1 IR

AT4G24770 chr4:12767559-12767589 1 53::38 33::6 9865::10246 8713::9505 0.552748169 1 A3SS

AT5G35560 chr5:13745271-13745350 8 18::97 4::121 54::72 66::70 0.552831919 1 IR

AT5G49210 chr5:19950074-19950080 2 294::6 255::8 227::480 212::451 0.552920015 1 A3SS

AT1G77840 chr1:29268867-29268978 2 149::14 169::3 149::316 208::426 0.553032741 1 A3SS

AT4G36980 chr4:17434142-17434320 11 166::154 160::274 75::266 135::368 0.553747142 1 IR

AT4G01450 chr4:609857-609955 4 45::167 12::160 171::166 169::151 0.55398619 1 A5SS

AT1G30550 chr1:10823315-10823340 2 19::0 22::0 23::25 28::32 0.553988612 1 A5SS

AT5G14910 chr5:4824660-4824687 3 1702::2 2029::21 1660::1840 1942::2165 0.55415098 1 A3SS

AT2G17670 chr2:7675464-7675571 1 0::146 0::172 63::56 76::62 0.554192303 1 IR

AT4G19985 chr4:10831068-10831078 3 177::33 242::50 98::241 153::319 0.554410187 1 A3SS

AT5G19550 chr5:6600183-6600237 8 762::13 1383::6 51::673 79::1230 0.554549568 1 A5SS

AT5G27830 chr5:9862607-9862616 6 30::85 20::152 57::52 74::80 0.554691188 1 A3SS

AT2G46550 chr2:19113175-19113259 2 2::633 10::417 353::295 226::246 0.554813299 1 IR

AT1G04140 chr1:1076119-1076136 13 54::576 95::746 167::554 240::737 0.554930479 1 A5SS

AT4G02450 chr4:1074668-1074677 4 27::1955 41::1907 1738::1110 1704::1040 0.555235529 1 A3SS

AT5G04420 chr5:1249484-1249756 1 65::11 76::29 6::53 14::65 0.55528571 1 IR

AT1G17440 chr1:5984253-5984353 8 97::58 160::50 41::101 37::134 0.555353456 1 IR

AT1G79750 chr1:30010637-30010654 2 11::1051 10::928 199::1314 223::1081 0.555478231 1 A5SS

AT1G58030 chr1:21464904-21464913 12 16::535 10::545 497::554 497::609 0.555859341 1 A3SS

AT3G16800 chr3:5722653-5722750 5 380::75 451::34 64::259 46::285 0.556813479 1 IR

AT3G50240 chr3:18626008-18626023 12 16::8 4::8 13::21 16::20 0.556957876 1 A3SS

AT1G18020 chr1:6203037-6203042 4 196::9 224::6 7::39 6::17 0.557111038 1 A3SS

AT4G09970 chr4:6246853-6246881 5 7::62 27::65 23::111 41::168 0.557826469 1 A3SS

AT3G59320 chr3:21926135-21926248 3 22::22 51::45 14::34 25::82 0.559835228 1 IR

AT1G21560 chr1:7554975-7555007 1 25::8 35::13 15::45 13::25 0.56039628 1 A5SS

AT2G21940 chr2:9352569-9352669 10 96::35 135::81 25::99 57::163 0.560983131 1 IR

AT1G03280 chr1:803543-803565 2 15::121 1::103 26::179 8::151 0.561050694 1 A3SS

AT3G15352 chr3:5180126-5180181 1-2 182::10 237::13 10::146 16::176 0.561065436 1 Cassette

AT1G78910 chr1:29665589-29665602 1 9::11 12::15 14::38 19::35 0.56171665 1 A5SS

AT1G56230 chr1:21046819-21046824 6 118::10 108::15 44::102 50::124 0.561780583 1 A3SS

AT3G55760 chr3:20700628-20700633 2 33::12 11::9 36::69 15::61 0.562185006 1 A3SS

AT1G19200 chr1:6625720-6625844 1 10::496 18::558 287::321 283::444 0.562863115 1 IR

AT1G18700 chr1:6441803-6441814 17 156::7 133::14 113::153 114::203 0.563570783 1 A3SS

AT4G37550 chr4:17645228-17645264 5 381::12 468::24 91::489 111::583 0.563971202 1 A5SS

AT5G42660 chr5:17104548-17104559 2 13::29 14::37 25::49 21::64 0.564035924 1 A5SS

AT4G31120 chr4:15132447-15132542 21 288::8 375::24 18::345 29::459 0.564065651 1 IR

AT2G38185 chr2:16000377-16000477 2 37::0 15::10 4::28 9::23 0.564625411 1 IR

AT1G62920 chr1:23305096-23305209 3 16::6 51::8 4::27 6::55 0.564630558 1 IR

AT2G43430 chr2:18037138-18037151 3 77::18 92::2 67::116 86::115 0.564703515 1 A5SS

AT1G44000 chr1:16709180-16709260 1-2 1994::22 2115::14 14::1548 13::1770 0.565119957 1 AltStart

AT1G14120 chr1:4834836-4834878 2 24::180 11::109 223::241 137::190 0.565369369 1 A3SS

AT5G55896 chr5:22631783-22631899 2-3 17::28 18::39 20::110 31::124 0.565448947 1 Cassette

AT5G38510 chr5:15419080-15419100 5 51::213 58::139 71::312 72::216 0.565537099 1 A5SS

AT3G29185 chr3:11156072-11156082 4 228::32 257::78 78::320 131::474 0.56630177 1 A3SS

AT1G15950 chr1:5480865-5480911 4-5 3162::25 2681::1 33::2528 26::2207 0.567314476 1 Cassette

AT3G61080 chr3:22607976-22607983 5 26::265 17::236 95::318 109::296 0.568162967 1 A3SS

AT2G29340 chr2:12598300-12598306 5 48::7 37::26 673::1138 725::1202 0.568335596 1 A5SS

AT3G24430 chr3:8869952-8869997 9 698::7 656::18 45::726 54::666 0.568585287 1 A3SS

AT2G19620 chr2:8487496-8487507 5 28::1 35::15 10::61 30::67 0.568683381 1 A3SS

AT1G71860 chr1:27028354-27028359 7 528::11 461::28 462::535 379::509 0.568839269 1 A3SS

AT3G04485 chr3:1199678-1199714 4 6::17 16::13 14::24 27::29 0.568957648 1 A3SS

AT3G55920 chr3:20744224-20744232 5 12::65 18::72 42::74 49::90 0.569036015 1 A5SS

AT5G53220 chr5:21596154-21596711 1 22::1 10::6 5::16 8::10 0.569468525 1 A5SS

AT2G26340 chr2:11215748-11215759 3 41::733 22::675 221::883 191::877 0.569861537 1 A3SS

AT5G08370 chr5:2691523-2691534 13 122::7 145::11 89::117 124::174 0.570286693 1 A5SS

AT1G71480 chr1:26932691-26932702 3 19::780 24::750 299::884 296::879 0.570699019 1 A3SS

AT1G27920 chr1:9728644-9728759 6 22::15 47::11 7::26 9::56 0.570804342 1 IR

AT4G37560 chr4:17647765-17647799 5 149::11 118::17 133::140 111::175 0.570899733 1 A3SS

AT1G58848 chr1:21796218-21796450 6 55::31 158::71 3::3 3::12 0.571356129 1 Cassette

AT5G18245 chr5:6029692-6029767 3 36::32 26::75 24::30 40::41 0.571367662 1 IR

AT1G10470 chr1:3443152-3443169 4 18::803 6::851 727::966 817::1098 0.571379548 1 A3SS

AT3G03270 chr3:761911-762211 4 0::1070 2::1809 439::641 697::985 0.571683718 1 AltEnd

AT2G21530 chr2:9220058-9220147 4 16::1376 2::1487 84::1383 91::1617 0.571703479 1 A3SS

AT3G49430 chr3:18333918-18333930 10 14::539 20::712 49::556 62::667 0.571786678 1 A3SS

AT4G33060 chr4:15949818-15949904 5-6 73::21 84::38 18::74 28::97 0.572338761 1 Cassette

AT2G17150 chr2:7469242-7469302 3 16::18 4::25 24::31 9::28 0.57239986 1 A5SS

AT2G18750 chr2:8125594-8125710 1 31::8 25::17 10::11 16::6 0.573338091 1 A5SS

AT5G66420 chr5:26524708-26524861 1 102::6 75::27 7::128 12::94 0.573546188 1 IR

AT2G28470 chr2:12173259-12173298 2 16::115 9::43 132::128 55::68 0.573830052 1 Cassette

AT2G01450 chr2:201184-201224 6 764::13 761::3 744::725 739::743 0.574031011 1 A5SS

AT5G59400 chr5:23958669-23958674 4 26::82 37::86 29::143 40::198 0.574070881 1 A3SS

AT5G41100 chr5:16450575-16450664 10 46::21 52::9 11::61 5::74 0.574582644 1 IR

AT3G10260 chr3:3172776-3172812 1 233::67 258::52 297::179 317::197 0.5748517 1 A5SS

AT4G13850 chr4:8021973-8022016 5 0::355 2::498 145::756 225::956 0.574895352 1 IR

AT5G47500 chr5:19271916-19271939 3 688::55 709::45 668::884 693::977 0.574987912 1 A5SS

AT1G61970 chr1:22907329-22907435 2 1::44 2::68 26::11 33::20 0.575661237 1 IR

AT3G28150 chr3:10471844-10472218 3 31::17 23::12 11::33 15::23 0.575922776 1 IR

AT2G40190 chr2:16786515-16786526 5 11::32 36::27 32::70 54::95 0.576141394 1 A3SS

AT4G19550 chr4:10660485-10660563 4 4::16 6::35 12::13 20::28 0.576303763 1 Cassette

AT4G16710 chr4:9398775-9398788 2 12::18 9::30 47::74 76::117 0.576468244 1 A3SS

AT1G13770 chr1:4723611-4723628 12 41::23 70::27 58::92 74::122 0.576712427 1 A3SS

AT1G34760 chr1:12743986-12744077 6 35::14 30::38 12::22 20::30 0.577036818 1 IR

AT1G43620 chr1:16429643-16429754 2 2::31 5::12 19::19 13::20 0.577327775 1 Cassette

AT5G46630 chr5:18922970-18923172 12 632::6 773::39 31::224 47::270 0.577331074 1 IR

AT2G45630 chr2:18796494-18796568 1 126::39 121::9 37::123 18::112 0.577561216 1 IR

AT1G63420 chr1:23517362-23517394 3-4 34::8 25::25 15::74 20::71 0.577721944 1 Cassette

AT2G37840 chr2:15852738-15852999 3 29::18 16::33 11::32 18::39 0.579170993 1 IR

AT5G50740 chr5:20637957-20637962 1 5::38 1::35 16::38 13::34 0.579584422 1 A5SS

AT5G43745 chr5:17572255-17572260 10 56::13 58::18 52::52 62::80 0.579643208 1 A3SS

AT4G21280 chr4:11334558-11334690 1 20::55840 0::48261 28232::17566 24381::14751 0.57975634 1 IR

AT5G16610 chr5:5445587-5445592 11 12::53 20::81 34::89 50::136 0.580245511 1 A3SS

AT3G02510 chr3:522428-522539 9 77::8 110::32 6::84 18::102 0.58045622 1 IR

AT1G70620 chr1:26629002-26629053 9 64::22 33::42 63::93 56::122 0.580582083 1 A3SS

AT1G72740 chr1:27381147-27381165 4 69::182 114::182 75::246 109::288 0.580724727 1 A5SS

AT3G61690 chr3:22833690-22833806 9 16::165 4::271 96::106 152::126 0.581117703 1 IR

AT4G33180 chr4:16001121-16001155 2 70::15 121::9 82::125 132::164 0.581478322 1 A3SS

AT5G48335 chr5:19588609-19588740 3 35::1010 27::1308 449::547 608::687 0.581624351 1 Cassette

AT3G19650 chr3:6824403-6824467 3 19::25 12::42 30::49 28::78 0.581828737 1 A3SS

AT5G20165 chr5:6805963-6806023 6 480::73 538::75 43::338 56::365 0.581846899 1 Cassette

AT3G56860 chr3:21049754-21050017 3 86::84 76::80 55::71 55::115 0.58190862 1 A3SS

AT4G08920 chr4:5725411-5725506 3 8::1877 16::1819 1024::1000 992::1007 0.582103713 1 IR

AT1G05540 chr1:1639823-1639921 2 42::193 36::78 114::153 44::94 0.58292386 1 IR

AT1G75180 chr1:28218147-28218330 2 78::101 31::58 47::269 43::251 0.583689476 1 AltStart

AT4G14910 chr4:8528478-8528643 7 356::10 353::39 20::215 35::237 0.583974209 1 IR

AT4G37553 chr4:17644342-17644355 5 8::390 24::359 114::443 115::425 0.58418794 1 A5SS

AT1G23080 chr1:8181002-8181083 3 1172::21 1555::90 92::402 156::537 0.584227655 1 IR

AT5G51620 chr5:20967695-20967765 3 49::205 68::288 133::174 197::241 0.584605553 1 Cassette

AT5G09250 chr5:2876081-2876121 3 311::51 399::36 68::105 82::141 0.584609989 1 A3SS

AT5G66550 chr5:26560863-26560870 3 15::292 3::257 214::289 170::220 0.585119258 1 A3SS

AT3G60300 chr3:22286579-22286586 6 26::488 71::697 258::513 381::719 0.585144745 1 A3SS

AT1G27630 chr1:9612349-9612429 2-3 28::7 26::16 17::53 22::73 0.585394803 1 AltStart

AT5G65685 chr5:26275329-26275407 4 9::88 21::178 44::83 83::120 0.585772208 1 Cassette

AT5G20790 chr5:7039984-7040229 1 0::1949 0::3604 1093::1992 2179::3534 0.5860894 1 IR

AT5G06770 chr5:2092106-2092166 2 60::104 88::101 63::112 59::128 0.586199694 1 Cassette

AT4G27745 chr4:13840078-13840196 1 7::30 10::15 29::38 33::46 0.586481526 1 A5SS

AT5G64600 chr5:25826746-25826784 7 8::35 16::35 12::53 25::51 0.586493872 1 A3SS

AT1G15750 chr1:5420280-5420431 3 17::331 0::257 167::184 128::115 0.586870669 1 Cassette_multi

AT5G05550 chr5:1639454-1639551 2 32::139 55::131 87::131 103::151 0.587224861 1 Cassette

AT5G16800 chr5:5524701-5524811 4 149::58 123::106 37::144 58::150 0.58767301 1 IR

AT5G64460 chr5:25773799-25773879 9 574::18 614::2 94::623 120::627 0.58774086 1 A3SS

AT3G56940 chr3:21077828-21077846 5 23219::203 19740::204 19557::25238 16674::22429 0.587819703 1 A3SS

AT3G19080 chr3:6598298-6598304 10 24::2 20::7 22::34 17::45 0.587925343 1 A3SS

AT4G25650 chr4:13082090-13082160 3 387::16 249::26 34::364 31::291 0.588037196 1 IR

AT2G18300 chr2:7953310-7953316 5 38::449 32::319 506::632 351::469 0.588125569 1 A3SS

AT2G28470 chr2:12173109-12173151 3 16::20 9::5 108::136 57::89 0.588383703 1 A3SS

AT5G02680 chr5:606045-606110 3 13::19 31::35 15::31 32::52 0.588720414 1 Cassette

AT2G47900 chr2:19612828-19613031 1 10::388 12::272 150::233 90::191 0.588814792 1 IR

AT5G10080 chr5:3151628-3151663 3 12::4 7::15 15::36 9::48 0.589103411 1 A3SS

AT4G25290 chr4:12943871-12943899 5 113::82 59::74 115::212 101::196 0.589377598 1 A3SS

AT4G32850 chr4:15853892-15853960 12 124::7 181::4 27::57 47::78 0.589919046 1 Cassette

AT4G30720 chr4:14974077-14974085 5 96::15 158::7 82::133 114::182 0.589963006 1 A5SS

AT3G04940 chr3:1366942-1367004 8 12::609 11::650 549::460 565::435 0.590269236 1 A5SS

AT5G04710 chr5:1359085-1359090 4 32::218 42::157 262::242 198::212 0.590330611 1 A3SS

AT1G64750 chr1:24053752-24053843 1 1378::162 1458::123 212::1650 200::1863 0.590656036 1 IR

AT3G56830 chr3:21043889-21044098 1 9::14 10::43 8::33 21::38 0.590825193 1 IR

AT5G58140 chr5:23529218-23529317 19 1128::47 1323::99 80::964 124::1128 0.591327416 1 IR

AT5G22720 chr5:7549056-7549137 4 45::0 89::18 4::57 20::84 0.591457349 1 Cassette

AT3G28900 chr3:10904150-10904229 2 76::4309 38::5910 2054::1932 2846::2478 0.591549337 1 Cassette

AT5G44200 chr5:17803900-17803996 1 195::70 195::32 53::124 28::113 0.591817776 1 IR

AT5G59710 chr5:24060234-24060245 7 7::112 12::78 41::121 34::120 0.591995266 1 A3SS

AT3G46440 chr3:17091635-17091725 1 87::5 65::16 11::69 15::57 0.592729437 1 IR

AT2G44850 chr2:18502148-18502158 7 22::677 45::720 555::720 598::839 0.59273284 1 A5SS

AT1G17730 chr1:6099650-6099867 2 8::289 14::310 32::266 56::265 0.593289028 1 A3SS

AT5G64572 chr5:25810804-25810816 1 1::415 1::805 549::628 994::1410 0.59424226 1 A5SS

AT2G38880 chr2:16240360-16240422 6 181::14 207::30 31::238 64::260 0.59533812 1 A5SS

AT5G53460 chr5:21701875-21701897 5 30::181 29::89 206::207 110::135 0.595511299 1 A5SS

AT1G62920 chr1:23304673-23304680 2 23::3 53::0 27::31 50::60 0.595607574 1 A3SS

AT4G20430 chr4:11019054-11019127 7 0::85 0::65 50::30 32::50 0.595996417 1 IR

AT4G05320 chr4:2718784-2718834 2 0::607 280::354 12472::12606 10837::10572 0.596058018 1 A5SS

AT5G49740 chr5:20206503-20206599 7 77::1021 77::805 428::527 311::400 0.597262002 1 IR

AT1G72930 chr1:27439930-27439973 1 956::5 1002::7 148::909 116::1016 0.597329421 1 A5SS

AT3G57470 chr3:21275500-21275592 2 24::12 28::4 16::19 21::38 0.597394458 1 A5SS

AT1G79090 chr1:29749366-29749457 6 62::355 136::461 179::223 249::242 0.598031163 1 IR

AT1G08110 chr1:2535570-2535585 2 323::760 304::584 485::940 399::801 0.598138554 1 Cassette

AT2G28305 chr2:12083285-12083292 4 435::5 350::15 370::546 307::531 0.598607323 1 A3SS

AT5G04690 chr5:1351351-1351393 3 14::0 21::0 20::27 24::16 0.598841258 1 A5SS

AT3G48110 chr3:17768995-17769120 24 11::304 4::399 177::158 202::216 0.599467053 1 IR

AT3G51260 chr3:19032636-19032658 3 0::2697 0::3063 1402::923 1554::1123 0.600249281 1 IR

AT5G05310 chr5:1574158-1574255 12 59::32 78::54 21::41 36::80 0.600356523 1 IR

AT3G63370 chr3:23405245-23405288 2 19::16 19::23 32::27 31::45 0.600709292 1 A5SS

AT3G57170 chr3:21161156-21161182 7 11::87 4::91 76::77 95::98 0.600934303 1 A3SS

AT5G61310 chr5:24654770-24654796 1 132::269 99::259 115::228 89::208 0.600981282 1 A5SS

AT5G17670 chr5:5821424-5821443 2 758::12 706::28 823::821 808::871 0.601053484 1 A3SS

AT3G07030 chr3:2223221-2223246 8 31::485 5::624 244::186 318::243 0.601744568 1 IR

AT3G29160 chr3:11130893-11130909 5 9::87 6::94 97::144 105::147 0.60214973 1 A3SS

AT1G68190 chr1:25559885-25559942 2-3 133::12 158::15 55::167 61::164 0.60331851 1 Cassette

AT1G29060 chr1:10148042-10148071 2 97::31 103::14 108::161 100::101 0.603645194 1 A3SS

AT1G80420 chr1:30237205-30237319 3 5::6 12::5 37::77 25::82 0.604603621 1 A3SS

AT5G02200 chr5:437563-438205 1 3::24 42::21 9::31 18::68 0.604782411 1 IR

AT4G34000 chr4:16297498-16297590 5 567::53 496::78 47::557 59::491 0.604876516 1 IR

AT4G24380 chr4:12613290-12613378 4 80::11 76::23 9::62 20::66 0.605191623 1 IR

AT5G07440 chr5:2356015-2356127 1 47::61 36::58 38::154 31::175 0.605303618 1 IR

AT1G05030 chr1:1439491-1439502 7 11::37 25::31 25::62 48::68 0.606051758 1 A5SS

AT2G45990 chr2:18920686-18920788 7 471::145 663::168 91::570 99::777 0.606290041 1 IR

AT2G32440 chr2:13775078-13775158 1-2 108::3 74::12 3::142 10::152 0.606396462 1 AltStart

AT3G12680 chr3:4027597-4027844 2 12::202 28::188 107::96 113::109 0.606408425 1 Cassette

AT2G19620 chr2:8488000-8488020 2 29::6 61::1 6::52 3::76 0.607097415 1 AltStart

AT1G07420 chr1:2279224-2279258 4 9::83 12::126 23::109 35::162 0.607844324 1 A3SS

AT1G48920 chr1:18101171-18101217 14 380::4896 558::6974 2427::2548 3437::3248 0.608205152 1 IR

AT5G04440 chr5:1255834-1255842 4 92::744 127::706 285::695 310::762 0.60862671 1 A3SS

AT3G17750 chr3:6077382-6077390 5 13::41 35::51 37::84 49::103 0.608665798 1 A3SS

AT5G06060 chr5:1825680-1825760 1-2 488::7 438::13 68::532 111::593 0.609256332 1 AltStart

AT4G00660 chr4:277444-277487 9 23::243 13::376 319::367 434::459 0.609387702 1 A5SS

AT2G27710 chr2:11817428-11817471 3 57::19022 0::21842 9942::9064 11410::10684 0.609388119 1 IR

AT1G10870 chr1:3621242-3621249 9 27::12 21::13 13::40 15::37 0.609693956 1 A3SS

AT1G31170 chr1:11134213-11134291 5 8::419 27::440 330::440 316::429 0.609853999 1 Cassette

AT4G33240 chr4:16037898-16037944 1 4::32 11::18 10::18 23::16 0.610067263 1 A5SS

AT4G30310 chr4:14833696-14833702 9 283::11 341::14 332::331 382::370 0.610476365 1 A3SS

AT3G22190 chr3:7833723-7833785 1 14::66 3::70 29::33 21::33 0.610484942 1 A5SS

AT4G06701 chr4:3940466-3940510 2 95::94 70::150 47::107 102::140 0.61053331 1 Cassette

AT3G16800 chr3:5721501-5721542 2 16::103 15::89 171::138 115::120 0.610987707 1 A5SS

AT2G07050 chr2:2928314-2928327 9 8::417 21::336 110::454 102::329 0.611497822 1 A3SS

AT3G29160 chr3:11131809-11131957 1 73::37 86::15 67::59 65::42 0.611882682 1 A5SS

AT2G48140 chr2:19687109-19687114 3 290::28 229::14 209::346 158::252 0.611906606 1 A3SS

AT1G08910 chr1:2858653-2858704 14 21::56 12::66 34::53 40::63 0.611981482 1 Cassette

AT4G31390 chr4:15234651-15234726 6 242::0 123::0 287::266 161::208 0.612917931 1 A3SS

AT5G28020 chr5:10028179-10028185 3 8::35 5::24 139::296 150::351 0.613313838 1 A3SS

AT5G45030 chr5:18172932-18172943 2 22::2 7::5 19::37 12::44 0.613334706 1 A3SS

AT5G22440 chr5:7436515-7436626 1 352::124 439::53 55::359 52::334 0.613459614 1 IR

AT3G06550 chr3:2039322-2039483 15 451::28 544::65 41::307 65::382 0.613831199 1 IR

AT1G69740 chr1:26232051-26232107 2 532::82 408::87 61::633 64::519 0.614108595 1 Cassette

AT5G15330 chr5:4980841-4980930 1 18::36 3::56 73::61 71::61 0.614146594 1 A5SS

AT3G26450 chr3:9682654-9682734 1-2 2383::27 1747::3 66::1432 49::1168 0.614282954 1 AltEnd

AT1G32370 chr1:11678024-11678058 5 57::16 28::26 91::113 116::148 0.614340519 1 A3SS

AT4G37895 chr4:17814633-17814646 2 46::0 27::2 44::31 32::28 0.614676703 1 A3SS

AT1G79820 chr1:30023689-30023727 11 10::70 33::72 63::88 82::134 0.614728764 1 A3SS

AT3G63190 chr3:23344403-23344475 2 16::1050 4::951 1222::1092 1066::922 0.614831193 1 A3SS

AT4G09550 chr4:6039687-6039695 2 16::280 41::297 150::309 192::401 0.614935094 1 A3SS

AT4G28080 chr4:13954162-13954213 13 10::489 20::474 41::500 58::443 0.615022681 1 A5SS

AT2G40160 chr2:16778371-16778396 3 14::2 23::2 9::29 17::43 0.615498243 1 A5SS

AT3G48380 chr3:17917701-17917708 4 76::8 110::13 71::115 86::137 0.615743093 1 A3SS

AT5G19140 chr5:6424793-6424873 1-2 3172::18 2711::18 36::2398 34::2274 0.615769955 1 AltStart

AT4G20320 chr4:10976054-10976103 7 82::4 87::21 110::126 99::118 0.615840443 1 A3SS

AT1G09530 chr1:3076748-3076808 1 75::32 72::53 34::96 62::101 0.615860556 1 A5SS

AT5G06280 chr5:1918108-1918368 1 19::65 36::79 37::340 43::316 0.615889971 1 A5SS

AT5G45030 chr5:18176018-18176106 7 361::21 343::21 50::260 46::262 0.615954582 1 IR

AT5G22730 chr5:7552465-7552471 1 17::31 6::4 105::93 88::95 0.615975268 1 A3SS

AT1G50300 chr1:18631101-18631119 3 34::110 60::116 53::97 71::114 0.616068113 1 A5SS

AT3G25560 chr3:9279596-9279693 11 0::158 0::150 75::149 79::160 0.616468563 1 IR

AT2G46450 chr2:19065943-19065948 2 120::32 148::40 87::88 96::98 0.617140506 1 A3SS

AT5G41370 chr5:16552363-16552572 6 11::23 34::18 19::37 18::63 0.617169198 1 A5SS

AT1G02100 chr1:391003-391026 6 270::36 241::41 241::287 248::370 0.617700166 1 A3SS

AT1G79500 chr1:29906105-29906288 15 1::39 20::38 47::43 85::53 0.617909647 1 A3SS

AT4G38670 chr4:18069740-18069815 3 18::160 32::133 100::84 78::99 0.617946628 1 IR

AT3G59110 chr3:21856437-21856444 3 88::6 118::14 55::83 80::118 0.617948267 1 A3SS

AT3G55080 chr3:20413205-20413218 9 17::49 21::102 54::90 84::133 0.618297438 1 A3SS

AT4G38240 chr4:17934465-17934482 4 12::91 3::97 18::118 13::112 0.618323836 1 A5SS

AT3G09920 chr3:3043812-3043868 2 18::34 23::9 32::88 15::78 0.618412656 1 A3SS

AT1G43850 chr1:16617231-16617492 1 25::29 21::10 16::24 13::16 0.618553431 1 A5SS

AT3G03340 chr3:788956-788993 9-10 68::54 95::60 36::104 36::138 0.61884949 1 Cassette

AT1G19000 chr1:6561316-6561321 3 18::422 24::442 1712::3617 1782::3231 0.618900655 1 A5SS

AT1G31410 chr1:11248342-11248356 4 23::104 30::79 91::146 77::159 0.619188194 1 A3SS

AT4G10970 chr4:6722029-6722138 1 41::53 51::30 39::16 69::14 0.619266005 1 A5SS

AT3G06290 chr3:1905655-1905870 6 20::8 12::13 5::19 13::21 0.619465479 1 A3SS

AT3G27470 chr3:10163170-10163270 1 22::19 38::7 19::17 18::27 0.619720986 1 A5SS

AT4G20380 chr4:11004896-11005022 2 273::3 269::21 16::374 23::364 0.619977348 1 IR

AT5G63200 chr5:25351210-25351239 8 67::14 81::23 31::80 43::104 0.62043331 1 A5SS

AT3G08950 chr3:2727926-2727939 3 60::7 50::17 22::54 28::63 0.621397895 1 A5SS

AT5G41040 chr5:16431450-16431537 1 50::0 18::5 3::49 4::33 0.621558016 1 IR

AT5G03190 chr5:758660-758843 1 75::406 13::258 201::306 127::205 0.621595298 1 IR

AT1G16490 chr1:5630314-5630389 3 4::14 19::8 11::8 6::16 0.622026557 1 IR

AT3G26890 chr3:9910873-9910944 4 61::9 46::3 24::132 26::137 0.622214827 1 Cassette

AT5G48150 chr5:19524072-19524083 3 17::233 18::162 174::487 124::501 0.622441041 1 A3SS

AT3G17880 chr3:6123961-6123982 3 111::0 73::0 111::108 88::134 0.623560052 1 A3SS

AT1G75020 chr1:28171125-28172347 2 46::80 15::90 34::85 33::72 0.623781658 1 Cassette

AT1G56190 chr1:21028332-21028428 1 20::1953 30::1210 815::1244 522::855 0.6242964 1 IR

AT2G21195 chr2:9083513-9083583 3 508::70 564::101 81::250 104::276 0.62492622 1 IR

AT5G03240 chr5:772291-772445 2 14::26 33::0 4924::4617 5862::6120 0.625035557 1 A3SS

AT5G55600 chr5:22522121-22522192 6 8::120 7::116 56::80 57::104 0.626223232 1 IR

AT4G25500 chr4:13024765-13024831 2 241::6 260::21 6::152 16::188 0.626390854 1 Cassette

AT2G34900 chr2:14725545-14725584 1 106::1 107::14 104::57 97::61 0.626906794 1 A5SS

AT1G13860 chr1:4743501-4743530 10 14::22 25::42 34::17 56::18 0.627122672 1 A3SS

AT4G33580 chr4:16140357-16140379 6 768::17 892::35 705::994 783::1028 0.627205309 1 A3SS

AT1G53780 chr1:20077745-20077988 2 50::76 62::46 49::90 43::108 0.627447941 1 A3SS

AT3G63530 chr3:23457014-23457072 6 174::15 155::20 63::220 51::204 0.627910654 1 A3SS

AT1G68680 chr1:25786581-25786592 2 20::430 5::549 69::152 77::172 0.628396585 1 A3SS

AT5G05800 chr5:1744940-1745137 1 16::62 19::53 53::50 65::42 0.628413125 1 A5SS

AT3G44310 chr3:15988894-15988977 5 0::12197 0::16009 6393::10171 8315::12918 0.628440922 1 IR

AT4G03260 chr4:1428171-1428328 1 10::322 7::368 142::542 164::639 0.628626949 1 AltStart

AT5G18640 chr5:6213067-6213160 1 13::36 12::26 29::37 28::49 0.629227573 1 A5SS

AT3G45590 chr3:16732670-16732748 1 24::7 27::16 8::22 14::31 0.629412785 1 IR

AT1G05230 chr1:1513825-1513831 12 128::23 95::33 53::135 43::133 0.630792777 1 A5SS

AT3G48200 chr3:17835608-17835624 7 156::4 119::13 145::248 118::206 0.631009059 1 A5SS

AT5G42940 chr5:17220362-17220386 1 111::14 79::5 107::39 72::27 0.63157257 1 A5SS

AT4G32420 chr4:15652483-15652530 2 17::8 23::9 8::52 6::59 0.631613132 1 Cassette

AT1G72130 chr1:27137650-27137736 2 90::88 62::137 59::182 82::186 0.631782135 1 IR

AT5G27540 chr5:9722581-9722622 1 89::10 69::21 14::38 25::40 0.632214649 1 A5SS

AT3G61440 chr3:22736370-22736427 3 86::5459 48::7255 2691::2862 3534::3884 0.632504336 1 Cassette

AT4G24830 chr4:12793344-12793477 10 22::1688 6::2092 975::696 1184::891 0.632944906 1 IR

AT4G22830 chr4:11991243-11991302 1 0::572 0::406 327::285 239::248 0.632988302 1 IR

AT2G47850 chr2:19596947-19597025 6 65::15 42::27 91::134 66::119 0.633001108 1 A3SS

AT5G07350 chr5:2324901-2324906 2 199::28 179::11 116::319 88::241 0.633287252 1 A3SS

AT1G01920 chr1:317760-317765 9 9::86 6::107 68::101 78::125 0.633856695 1 A3SS

AT5G41350 chr5:16543024-16543164 2 16::56 3::69 24::57 27::69 0.6338658 1 Cassette

AT2G24420 chr2:10383151-10383163 15 33::48 18::5 258::65 287::78 0.633905812 1 A3SS

AT2G25420 chr2:10819531-10819552 10 1::16 19::16 8::21 27::32 0.634634097 1 A3SS

AT1G28060 chr1:9779825-9779905 2 9::54 28::52 27::52 32::65 0.635231493 1 IR

AT5G11580 chr5:3719533-3719543 3 35::102 39::74 95::159 94::161 0.636113353 1 A3SS

AT1G15200 chr1:5229128-5229231 9-10 98::49 88::64 40::165 67::189 0.636168749 1 Cassette

AT5G58260 chr5:23561296-23561362 2 1085::16 1263::37 86::1020 109::1211 0.636380866 1 IR

AT5G02830 chr5:646771-646781 6 15::208 5::164 175::240 132::265 0.636466686 1 A3SS

AT5G46280 chr5:18770049-18770078 14 66::33 80::18 83::154 109::153 0.636819658 1 A5SS

AT1G64750 chr1:24053702-24053752 2 15::1378 39::1458 2037::2226 2206::2611 0.636989107 1 A3SS

AT5G53460 chr5:21702183-21702251 5-6 11::19 17::47 32::160 47::136 0.637322863 1 Cassette

AT5G13020 chr5:4131090-4131099 6 22::329 24::373 233::366 269::411 0.637401725 1 A3SS

AT1G70740 chr1:26675237-26675242 3 5::54 11::41 30::61 23::53 0.637487884 1 A3SS

AT1G58100 chr1:21513539-21513612 1 0::726 5::647 357::365 308::406 0.637545449 1 IR

AT5G03995 chr5:1077755-1077788 2 4::11 0::39 17::22 42::32 0.637675858 1 A3SS

AT2G48160 chr2:19691316-19691332 8 3::139 22::167 115::200 140::243 0.638236254 1 A3SS

AT1G18415 chr1:6344772-6344785 2 8::13 11::14 13::20 17::30 0.638242948 1 A5SS

AT2G46225 chr2:18983403-18983439 6 255::10 245::21 261::230 270::290 0.638365061 1 A3SS

AT2G27340 chr2:11697163-11697449 2 14::15 17::62 14::35 28::64 0.638728509 1 IR

AT1G78800 chr1:29626729-29626734 5 24::59 22::86 53::84 70::115 0.639118926 1 A5SS

AT5G05930 chr5:1781048-1781090 7 10::121 5::216 29::157 34::207 0.640257924 1 A5SS

AT1G02880 chr1:644256-644261 3 46::16 32::34 36::76 45::91 0.640381697 1 A3SS

AT2G37585 chr2:15766940-15766959 2 61::7 57::22 25::62 37::70 0.640400323 1 A5SS

AT2G24060 chr2:10229928-10229947 3 9::486 24::541 116::462 135::577 0.640524688 1 A3SS

AT5G22730 chr5:7552471-7553374 1 31::156 4::133 65::96 56::86 0.640943497 1 IR

AT4G01915 chr4:827717-827801 2 46::89 43::56 48::118 28::94 0.641242356 1 IR

AT1G77080 chr1:28958745-28958783 5 160::27 161::10 180::192 292::353 0.641477612 1 A3SS

AT2G02470 chr2:654337-654364 5 108::95 86::117 150::194 127::213 0.64150827 1 A3SS

AT2G38280 chr2:16033727-16033742 19 88::196 131::304 119::298 155::436 0.641515831 1 A5SS

AT1G72280 chr1:27212425-27212449 8 14::15 18::9 22::24 17::40 0.64156319 1 A3SS

AT1G16080 chr1:5515631-5515648 5 11::1444 6::1514 297::606 337::686 0.641871926 1 A3SS

AT1G52500 chr1:19561687-19561796 7 22::634 17::1000 685::311 964::439 0.642587841 1 Cassette

AT2G38810 chr2:16220681-16220826 2 88::37 88::66 27::201 47::225 0.642639017 1 IR

AT1G33060 chr1:11975703-11975715 7 1::56 22::60 60::30 80::43 0.642676236 1 A3SS

AT5G58510 chr5:23650408-23650421 15 29::46 31::52 36::67 44::86 0.642807747 1 A5SS

AT1G27920 chr1:9727027-9727161 1 39::20 117::20 29::61 47::140 0.642886143 1 A5SS

AT5G64500 chr5:25782218-25782240 8 7::292 21::283 314::340 299::293 0.643061083 1 A5SS

AT4G13150 chr4:7650704-7650717 8 14::147 5::147 135::143 181::181 0.643168179 1 A3SS

AT3G26440 chr3:9679449-9679568 14 45::22 36::11 19::40 10::45 0.643208728 1 IR

AT4G40040 chr4:18558565-18558646 1 31::5931 11::5488 62::2224 29::2027 0.643217819 1 A5SS

AT5G42770 chr5:17152682-17153297 5 69::14 95::17 101::88 129::109 0.643728475 1 MXE

AT4G28880 chr4:14251224-14251328 1 24::221 29::153 48::385 37::276 0.644003371 1 IR

AT5G24060 chr5:8132787-8132826 3 23::133 3::81 141::121 81::86 0.644162238 1 A3SS

AT3G17590 chr3:6018268-6018306 7 10::355 13::405 174::183 180::196 0.6442189 1 Cassette

AT3G03180 chr3:733666-733757 2 12::92 5::110 46::59 62::64 0.644481854 1 Cassette

AT1G20540 chr1:7113293-7113306 4 17::107 26::155 109::136 132::174 0.645014119 1 A5SS

AT1G07780 chr1:2412194-2412219 2 27::173 30::246 73::132 97::143 0.645069348 1 Cassette

AT2G30424 chr2:12964633-12964647 3 15::42 11::52 52::74 58::83 0.645093303 1 A3SS

AT3G12990 chr3:4156206-4156331 1 14::17 23::23 10::71 15::88 0.645112631 1 IR

AT4G19490 chr4:10623043-10623267 18 79::87 129::108 51::128 60::163 0.645134342 1 IR

AT2G44140 chr2:18257008-18257083 3 69::6 65::10 33::138 66::141 0.645208402 1 A3SS

AT3G53500 chr3:19835912-19836130 3 520::84 620::89 142::629 191::764 0.645487478 1 A3SS

AT5G20300 chr5:6856439-6856518 2 23::5 19::9 7::57 13::39 0.645681522 1 Cassette

AT5G27730 chr5:9821282-9821293 6 14::65 31::56 40::87 48::127 0.645752752 1 A3SS

AT3G03040 chr3:684680-684685 2 40::12 61::5 39::70 54::106 0.645779159 1 A3SS

AT3G52070 chr3:19313093-19313125 1 0::14 2::28 85::177 89::152 0.647162629 1 A5SS

AT2G24270 chr2:10329607-10329618 2 72::164 39::144 4103::5989 2340::3718 0.64749471 1 A3SS

AT5G58140 chr5:23528510-23528726 16 863::21 914::51 49::885 74::913 0.647533506 1 IR

AT3G61880 chr3:22906047-22906120 2 0::25 0::38 16::27 22::24 0.64769351 1 IR

AT5G18310 chr5:6062261-6062346 2 48::416 100::504 231::251 272::314 0.647729133 1 IR

AT1G34320 chr1:12521691-12521709 5 7::130 19::143 34::133 47::148 0.648309673 1 A3SS

AT3G02830 chr3:615271-615277 6 805::28 788::15 1109::1148 1168::1343 0.648513547 1 A3SS

AT4G00975 chr4:422656-422779 2 2::30 2::86 20::26 37::36 0.648551413 1 IR

AT5G48760 chr5:19772700-19772714 1 0::658 0::551 383::2108 313::2081 0.649001511 1 IR

AT1G53040 chr1:19767221-19767326 1 10::20 9::17 9::22 9::15 0.649016413 1 A5SS

AT4G19990 chr4:10835371-10835476 6 3::9 11::19 7::10 16::17 0.649039085 1 IR

AT4G03115 chr4:1384896-1384976 2-3 53::14 44::11 13::30 14::40 0.649541104 1 AltStart

AT4G26070 chr4:13217685-13217795 1 1::31 10::29 22::28 16::41 0.649662242 1 IR

AT4G24740 chr4:12755037-12755048 11 9::405 6::740 373::559 657::927 0.649773006 1 A3SS

AT3G58670 chr3:21704957-21704992 3 17::182 6::169 176::184 167::218 0.650000715 1 A3SS

AT2G15480 chr2:6762926-6763246 3 40::0 33::0 54::17 57::37 0.650318272 1 A3SS

AT1G13450 chr1:4613516-4613755 3 99::36 103::87 30::134 44::172 0.650637995 1 IR

AT1G68310 chr1:25599617-25599669 2 28::179 34::142 120::230 99::256 0.651015297 1 AltStart

AT2G45850 chr2:18873521-18873620 1 7::66 20::38 39::107 18::93 0.651048141 1 IR

AT1G01750 chr1:277137-277153 4 8::15 11::27 15::27 29::35 0.651278347 1 A5SS

AT2G21385 chr2:9150092-9150112 3 311::12 257::18 349::371 320::337 0.651312943 1 A3SS

AT5G16715 chr5:5486300-5486314 4 10::135 17::105 116::111 96::114 0.651365863 1 A5SS

AT1G62620 chr1:23182437-23182470 before first 50::2 68::16 13::80 45::98 0.651514709 1 Cassette

AT3G28130 chr3:10467732-10467978 4 14::216 3::281 141::173 171::203 0.651612306 1 Cassette_multi

AT5G14640 chr5:4721656-4721805 2 19::794 0::545 484::333 336::243 0.652092563 1 Cassette

AT5G65060 chr5:25990084-25990122 4 2::52 8::59 10::58 42::101 0.652308799 1 A3SS

AT5G41370 chr5:16552660-16552695 7 10::24 6::45 20::90 33::101 0.652622652 1 A3SS

AT2G21520 chr2:9216208-9216220 3 50::13 45::9 51::107 47::103 0.653193094 1 A3SS

AT1G62540 chr1:23154534-23154588 5 22::210 32::153 215::208 196::193 0.653284649 1 A5SS

AT3G59330 chr3:21926961-21927053 6 4::8 0::27 10::33 21::53 0.654237254 1 AltEnd

AT2G39250 chr2:16389677-16389694 6 7::108 13::111 112::118 99::129 0.654340668 1 A3SS

AT3G54350 chr3:20126623-20126764 1 71::9 59::11 9::64 11::45 0.654355902 1 IR

AT3G01790 chr3:284360-284450 5-6 727::17 977::28 17::642 22::868 0.655057413 1 Cassette

AT3G06720 chr3:2123557-2123567 10 601::12 837::9 287::554 389::703 0.655262921 1 A5SS

AT2G34080 chr2:14394324-14394348 1 15::9 9::12 27::25 22::40 0.655464913 1 A5SS

AT5G14610 chr5:4712582-4712725 5 68::10 86::13 17::116 14::140 0.655654812 1 Cassette_multi

AT1G02205 chr1:419529-419675 3 246::13 50::6 19::276 7::61 0.656554254 1 IR

AT1G76170 chr1:28585958-28585966 2 3::21 11::19 24::35 26::30 0.656674413 1 A3SS

AT4G31150 chr4:15145446-15145519 1 36::28 36::36 17::59 25::62 0.65697799 1 IR

AT2G26670 chr2:11342095-11342239 1 51::646 15::595 611::529 520::396 0.657566487 1 A5SS

AT5G22760 chr5:7575479-7575484 6 20::6 47::10 21::52 43::68 0.657954844 1 A3SS

AT3G52120 chr3:19331347-19331455 7 312::66 407::134 64::373 102::490 0.657961795 1 IR

AT1G75295 chr1:28256642-28256716 2 26::27 11::47 13::52 24::66 0.658321792 1 IR

AT3G01150 chr3:53911-53958 8 44::155 34::193 176::200 204::198 0.658443937 1 A5SS

AT1G66760 chr1:24904036-24904148 6 520::59 540::29 49::292 39::275 0.658572546 1 IR

AT1G70410 chr1:26536517-26536604 3 201::14 179::32 51::360 45::248 0.658899798 1 IR

AT1G34150 chr1:12436696-12436703 3 8::35 11::45 22::39 28::55 0.658904357 1 A5SS

AT4G12790 chr4:7518955-7519081 2 95::153 100::112 176::313 129::309 0.659015425 1 A3SS

AT2G25850 chr2:11026081-11026196 12 8::11 13::38 7::15 24::19 0.659372017 1 IR

AT3G27990 chr3:10395644-10395724 5-6 51::9 64::7 19::70 20::88 0.659393114 1 AltEnd

AT2G32960 chr2:13988673-13988768 3 12::220 4::341 115::147 167::210 0.659555355 1 Cassette

AT3G21710 chr3:7649060-7649348 2 88::22 102::71 9::85 22::107 0.659612951 1 IR

AT3G53100 chr3:19684686-19685182 5 55::392 78::314 241::156 258::147 0.659861023 1 AltEnd

AT5G43130 chr5:17315750-17315837 15 180::0 277::0 102::108 57::107 0.659986409 1 A3SS

AT5G02020 chr5:386721-387041 3 759::131 386::41 88::752 30::353 0.660005318 1 IR

AT3G62310 chr3:23058402-23058520 4 197::44 187::53 87::313 154::392 0.660188213 1 A3SS

AT1G56610 chr1:21212368-21212396 3 18::0 27::0 38::46 33::60 0.660263543 1 A3SS

AT3G53610 chr3:19878266-19878302 2 415::14 351::5 78::337 61::287 0.660306594 1 A3SS

AT3G52360 chr3:19412556-19412564 2 1140::25 967::5 863::724 729::624 0.660688281 1 A3SS

AT1G09280 chr1:2999023-2999028 8 12::107 19::182 53::146 80::180 0.660745138 1 A5SS

AT5G17620 chr5:5807102-5807113 6 109::12 105::14 35::170 37::162 0.661645789 1 A3SS

AT1G80030 chr1:30108122-30108141 2 21::310 18::289 79::323 76::305 0.661659685 1 A5SS

AT5G19221 chr5:6465659-6465743 5 981::7 815::26 72::608 72::533 0.661881133 1 IR

AT5G38030 chr5:15172593-15172634 3-4 121::20 106::8 13::131 15::121 0.662067995 1 Cassette

AT2G48070 chr2:19663657-19663669 3 712::22 855::9 319::823 368::988 0.662104324 1 A5SS

AT5G26280 chr5:9210240-9210246 7 1027::12 1748::5 901::519 1549::898 0.662192006 1 A3SS

AT2G28550 chr2:12226383-12226456 8 936::44 1108::102 59::575 101::790 0.662395153 1 IR

AT5G07150 chr5:2216381-2216416 4 0::18 2::20 23::43 21::51 0.662481843 1 A3SS

AT5G51620 chr5:20967875-20967951 4 85::81 100::151 110::132 141::207 0.662636543 1 A3SS

AT4G38520 chr4:18017682-18017948 1 338::30 430::57 22::494 50::581 0.663501878 1 IR

AT2G24040 chr2:10224394-10224443 2 210::34 252::28 207::148 284::210 0.663506123 1 A3SS

AT4G31390 chr4:15233460-15233476 1 11::233 10::133 262::247 178::161 0.66385788 1 A5SS

AT2G20180 chr2:8704280-8704359 9 39::210 28::241 170::90 224::102 0.664022992 1 A3SS

AT3G05350 chr3:1529805-1529812 4 86::21 92::38 28::144 38::128 0.664254716 1 A3SS

AT4G02120 chr4:942816-942827 13 15::146 8::224 129::115 213::239 0.664450512 1 A5SS

AT1G20880 chr1:7262757-7262780 6 18::60 20::85 65::95 100::120 0.664718637 1 A3SS

AT1G08390 chr1:2642541-2642599 2 11::164 15::190 168::214 206::270 0.664944633 1 A5SS

AT5G17530 chr5:5779880-5779885 9 159::8 120::8 103::120 93::105 0.665654254 1 A3SS

AT1G72860 chr1:27417724-27417841 3 10::25 52::31 14::18 18::35 0.66584885 1 IR

AT2G01450 chr2:201051-201097 7 13::764 3::761 790::875 803::881 0.666086115 1 A3SS

AT4G17310 chr4:9685977-9686024 2-3 69::34 72::23 50::52 47::54 0.666172662 1 Cassette

AT5G53140 chr5:21549208-21549511 2 14::839 1::676 739::578 584::510 0.666174741 1 A5SS

AT3G23640 chr3:8502307-8502327 2 72::36 73::36 71::130 82::91 0.666740581 1 A3SS

AT4G18120 chr4:10038415-10038460 2 13::3 43::1 6::81 4::140 0.666740728 1 AltStart

AT5G19430 chr5:6554072-6554214 1 232::19 295::8 31::126 36::152 0.66706098 1 A5SS

AT2G05440 chr2:1993694-1993746 2 14::13 5::6 4531::1763 4537::1787 0.667643127 1 A3SS

AT1G07745 chr1:2402620-2402700 1-2 21::11 26::26 15::33 31::49 0.667703759 1 AltStart

AT3G47675 chr3:17576463-17576537 1 19::12 28::47 38::71 137::125 0.667938516 1 AltStart

AT5G26240 chr5:9190405-9190420 4 8::66 15::47 27::70 27::83 0.667939613 1 A3SS

AT2G30460 chr2:12978677-12978843 1 28::10 21::21 16::41 26::44 0.668154845 1 IR

AT5G45300 chr5:18354286-18354292 2 36::13 21::4 48::43 29::42 0.668351222 1 A3SS

AT3G56950 chr3:21078483-21078590 3 0::405 0::511 238::8537 288::9139 0.66852449 1 IR

AT3G59680 chr3:22045191-22045256 2 23::44 15::38 32::79 29::86 0.66860409 1 A3SS

AT4G05050 chr4:2588401-2588858 3 104::18586 109::17200 10888::4465 9999::4329 0.668753202 1 IR

AT1G05630 chr1:1685484-1685587 6 35::18 37::20 16::44 15::52 0.66882223 1 IR

AT3G18520 chr3:6363413-6363475 9 11::278 10::369 279::255 336::279 0.669065195 1 A5SS

AT4G20020 chr4:10844824-10844870 4 11::711 35::1068 361::349 570::436 0.669158871 1 IR

AT2G23930 chr2:10182365-10182387 1 18::2014 41::2394 1913::926 2218::1054 0.669205525 1 A5SS

AT3G11470 chr3:3611724-3611729 3 36::55 15::35 28::75 14::58 0.669273588 1 A5SS

AT2G24060 chr2:10231304-10231335 9 40::1404 28::1917 731::420 1035::581 0.670297422 1 IR

AT5G25560 chr5:8899746-8899761 3 191::17 271::38 177::238 227::328 0.670390781 1 A3SS

AT1G23490 chr1:8337218-8337223 2 2325::19 1886::16 1269::2156 1027::1910 0.670412633 1 A3SS

AT1G04960 chr1:1410318-1410619 1 173::6 126::16 9::97 10::80 0.670830667 1 IR

AT1G07830 chr1:2422352-2422432 1-2 415::16 465::28 31::411 58::532 0.671090207 1 AltStart

AT1G78070 chr1:29356665-29356708 4-5 142::103 66::64 66::226 57::126 0.671298509 1 Cassette

AT4G14930 chr4:8540946-8540951 8 461::16 498::4 371::437 382::487 0.672006738 1 A3SS

AT5G57300 chr5:23210875-23210955 2 204::14 181::6 13::169 5::187 0.672109769 1 AltStart

AT5G46280 chr5:18769697-18769958 15 33::66 18::80 17::65 25::63 0.672223173 1 AltEnd

AT4G10430 chr4:6455326-6455443 10 18::307 22::442 42::196 57::211 0.672241366 1 MXE

AT1G63940 chr1:23732903-23732991 14 669::6 860::12 64::705 87::929 0.672312682 1 IR

AT5G63160 chr5:25334614-25334687 3 10::363 13::131 484::496 169::197 0.672400532 1 A3SS

AT5G05140 chr5:1521360-1521398 5 10::77 8::70 21::90 21::87 0.672552572 1 A3SS

AT4G28706 chr4:14169327-14169332 8 24::149 40::148 46::192 49::189 0.672739136 1 A3SS

AT2G01420 chr2:182005-182017 1 236::403 233::263 309::426 269::316 0.6729789 1 A5SS

AT3G53990 chr3:19990567-19990654 2 813::23 906::6 77::711 77::756 0.672980317 1 IR

AT5G01010 chr5:4237-4258 4 21::613 7::581 564::624 560::592 0.673064455 1 A3SS

AT3G10915 chr3:3417036-3417377 1 148::25 145::55 26::107 42::123 0.673139304 1 IR

AT4G29270 chr4:14424251-14424269 2 20::895 5::662 880::867 686::754 0.673165979 1 A3SS

AT1G14310 chr1:4885605-4885619 3 78::3 82::15 13::107 26::85 0.673536987 1 A5SS

AT1G56280 chr1:21073616-21073711 4 0::17115 17::19926 9743::7403 11320::8735 0.673559524 1 IR

AT5G16800 chr5:5524811-5524836 4 149::25 123::23 172::201 160::187 0.673849119 1 A5SS

AT1G75380 chr1:28281708-28281784 10 787::58 1128::44 87::328 104::444 0.673942661 1 IR

AT1G02850 chr1:631928-632009 8 27::132 8::105 52::181 63::165 0.674352614 1 A3SS

AT3G18500 chr3:6354697-6354709 10 26::83 24::117 41::123 62::200 0.674622467 1 A3SS

AT3G03690 chr3:912270-912385 3 24::128 9::155 60::98 77::98 0.674685073 1 Cassette

AT5G35160 chr5:13414873-13414943 2 24::15 11::24 177::195 117::135 0.674742767 1 A3SS

AT5G62790 chr5:25216417-25216484 4 537::0 454::0 11::535 25::448 0.674814918 1 Cassette

AT1G24260 chr1:8594201-8594242 6 0::26 1::72 17::22 34::49 0.674859221 1 Cassette

AT1G80940 chr1:30411680-30411749 2 201::149 395::159 106::279 108::331 0.675114626 1 IR

AT2G15000 chr2:6481828-6481871 2 28::553 61::710 81::558 136::755 0.675862723 1 A5SS

AT1G23110 chr1:8197848-8198052 2 23::40 74::59 67::68 132::109 0.675984613 1 A5SS

AT1G04420 chr1:1192713-1192718 5 37::630 51::555 404::641 373::614 0.676225262 1 A3SS

AT1G14685 chr1:5042588-5042855 2 139::15 133::3 25::223 45::253 0.676603784 1 AltStart

AT2G20570 chr2:8855944-8855950 1 92::1375 67::1789 948::1290 1219::1643 0.676720693 1 A5SS

AT5G22090 chr5:7316543-7316651 2 0::680 0::558 397::834 318::858 0.676865854 1 AltStart

AT3G55740 chr3:20696496-20696510 2 32::11 42::1 52::72 52::85 0.677004546 1 A3SS

AT2G43560 chr2:18074544-18074578 4 8::1121 28::1379 1292::1077 1588::1337 0.677019349 1 A3SS

AT4G31877 chr4:15413759-15413822 2-3 148::45 103::30 80::160 63::94 0.677069624 1 Cassette

AT1G14570 chr1:4985428-4985491 5 238::8 168::17 279::367 233::371 0.677276088 1 A3SS

AT3G05840 chr3:1740700-1740729 2 269::27 185::19 341::653 266::553 0.677620793 1 A3SS

AT3G17030 chr3:5805792-5805800 9 22::7 18::18 14::28 21::38 0.678497251 1 A3SS

AT1G17350 chr1:5942348-5942381 2 19::3 19::20 34::67 32::96 0.678667308 1 A3SS

AT1G01448 chr1:164103-164135 2 15::5 40::3 12::35 8::54 0.679167617 1 A5SS

AT5G06530 chr5:1992851-1992921 3 20::443 3::348 222::257 185::183 0.679587998 1 Cassette

AT5G49215 chr5:19953675-19953681 1 3::14 0::10 7::6 7::11 0.679866372 1 A5SS

AT3G17365 chr3:5948063-5948158 3 62::12 90::5 12::56 11::78 0.68078258 1 IR

AT1G27752 chr1:9664128-9664208 4-5 49::17 53::3 12::84 9::84 0.681054889 1 AltEnd

AT1G08660 chr1:2759406-2759413 2 64::14 92::12 45::69 70::99 0.681173593 1 A5SS

AT4G33000 chr4:15926040-15926141 8 48::159 95::227 84::187 112::237 0.681251897 1 IR

AT3G59360 chr3:21935804-21935812 14 221::113 268::139 208::275 259::353 0.681818123 1 A5SS

AT1G08810 chr1:2819858-2819970 2 25::19 12::41 10::63 18::70 0.682203791 1 IR

AT5G57770 chr5:23400919-23401110 8 12::259 11::731 135::116 388::292 0.682270672 1 IR

AT3G59080 chr3:21837436-21837545 1 2::60 0::44 40::33 23::22 0.682465252 1 IR

AT1G28280 chr1:9886310-9886678 1 0::232 26::296 45::359 53::364 0.682515223 1 IR

AT4G16695 chr4:9395061-9395142 3 487::90 590::148 52::366 73::438 0.682523489 1 Cassette

AT5G36700 chr5:14422035-14422137 10 1203::45 1318::75 3::9 3::7 0.68264541 1 IR

AT2G34410 chr2:14523214-14523220 17 72::33 83::0 218344::227259 200665::208402 0.68273107 1 A3SS

AT3G16520 chr3:5618826-5618851 3 21::15 10::32 23::20 44::28 0.682831106 1 A3SS

AT2G31900 chr2:13562887-13563057 30 8::4 10::8 7::18 8::22 0.682882446 1 Cassette

AT5G49470 chr5:20064096-20064110 3 18::36 7::37 22::40 15::49 0.683203333 1 A3SS

AT4G26690 chr4:13456646-13456747 8 16::979 4::978 505::402 491::405 0.683404563 1 IR

AT5G07370 chr5:2331227-2331233 4 207::14 215::15 282::343 295::405 0.683473242 1 A3SS

AT1G55690 chr1:20808645-20808724 15 83::15 99::5 13::52 8::66 0.683631927 1 IR

AT5G63050 chr5:25293066-25293135 2 15::559 9::457 292::241 257::201 0.683929219 1 Cassette

AT1G57540 chr1:21311221-21311413 1 143::18 193::7 7::139 6::192 0.684042376 1 IR

AT5G47080 chr5:19125610-19125625 3 11::91 18::87 102::126 106::155 0.684278075 1 A3SS

AT5G28770 chr5:10797620-10797641 2 22::251 34::190 252::301 204::280 0.684519471 1 A3SS

AT5G26742 chr5:9285000-9285114 10 0::47 0::33 22::2069 15::2522 0.684565244 1 IR

AT5G54650 chr5:22201713-22201853 1 0::31 0::34 19::12 18::11 0.684921189 1 IR

AT5G26622 chr5:9356305-9356428 2 25::2 15::0 118::105 184::139 0.685092431 1 A3SS

AT3G16950 chr3:5786690-5786785 15 40::706 42::752 364::231 401::229 0.68529882 1 IR

AT1G55870 chr1:20896344-20896363 3 32::2 61::19 26::44 57::69 0.685614838 1 A3SS

AT1G71240 chr1:26857122-26857127 6 14::47 9::26 15::64 11::64 0.685627124 1 A3SS

AT1G70620 chr1:26630582-26630701 11 92::78 96::136 51::63 70::104 0.686281296 1 IR

AT1G60995 chr1:22466995-22467000 11 8::52 13::71 71::67 79::78 0.686455321 1 A3SS

AT2G04700 chr2:1647553-1647570 4 1234::16 1408::35 1050::1203 1166::1301 0.686767733 1 A3SS

AT4G33000 chr4:15925657-15925725 6 140::19 192::18 141::137 197::187 0.686857329 1 A3SS

AT1G78070 chr1:29357408-29357413 6 10::233 7::192 225::255 174::191 0.687031394 1 A5SS

AT2G27720 chr2:11819138-11819187 4 54::17590 17::19568 9169::9038 10300::10104 0.687347757 1 IR

AT2G23560 chr2:10029478-10029564 3 5::23 17::43 14::18 25::24 0.687510004 1 IR

AT3G16220 chr3:5498155-5498428 3 12::184 4::227 132::148 159::199 0.687624591 1 Cassette_multi

AT1G80270 chr1:30181053-30181077 1 38::67 27::58 69::46 65::41 0.687769375 1 A5SS

AT1G59750 chr1:21983072-21983081 13 237::96 249::93 263::305 273::399 0.687802984 1 A3SS

AT1G70650 chr1:26641075-26641098 7 22::1160 29::1054 1106::1086 1003::978 0.6879269 1 A5SS

AT2G01100 chr2:81800-82186 2 139::15 183::46 28::242 58::321 0.687996917 1 IR

AT5G66240 chr5:26466274-26466364 1 288::808 257::900 509::439 559::440 0.688007661 1 IR

AT1G31600 chr1:11314994-11315569 2 47::96 27::133 39::136 70::193 0.688327747 1 IR

AT2G29290 chr2:12587000-12587108 4 798::20 710::4 1019::1058 997::1074 0.68854979 1 A3SS

AT1G05570 chr1:1655162-1655286 16 0::43 0::57 34::33 36::26 0.688716496 1 IR

AT4G21860 chr4:11600109-11600202 3 1::802 20::1069 469::1719 599::1978 0.688804279 1 IR

AT3G28070 chr3:10448446-10448451 3 6::158 1::171 135::229 156::237 0.689037976 1 A3SS

AT2G43920 chr2:18190230-18190254 4 14::398 15::727 84::420 137::726 0.689055022 1 A5SS

AT3G47250 chr3:17401594-17401609 2 125::245 106::185 311::531 241::544 0.689419999 1 A3SS

AT4G21760 chr4:11565823-11565834 13 11::88 9::69 72::102 55::72 0.689612404 1 A5SS

AT3G16220 chr3:5498831-5498862 7 13::220 3::327 37::162 43::227 0.689834693 1 A3SS

AT3G27770 chr3:10287280-10287397 2 17::602 8::745 312::345 404::446 0.689971939 1 IR

AT3G15430 chr3:5208818-5208925 1 13::59 23::35 30::42 21::37 0.690066147 1 IR

AT3G56650 chr3:20985307-20985318 2 106::515 137::423 154::558 169::587 0.690645255 1 A3SS

AT1G53380 chr1:19914868-19915012 3 130::1 90::9 7::182 7::208 0.690752567 1 AltStart

AT5G48150 chr5:19524309-19524388 2 86::259 48::210 136::435 101::445 0.691031487 1 Cassette

AT3G15010 chr3:5054062-5054606 1 239::132 376::208 81::243 124::303 0.691060018 1 IR

AT1G61180 chr1:22554110-22554286 1 24::30 84::43 14::48 23::47 0.691573764 1 IR

AT1G33470 chr1:12145353-12145362 3 295::10 356::11 275::321 348::460 0.692265341 1 A3SS

AT3G16700 chr3:5689266-5689331 5 3::31 5::149 20::23 89::74 0.692398142 1 IR

AT3G57520 chr3:21289307-21289341 14 0::444 4::500 459::426 494::537 0.692549079 1 A3SS

AT3G62190 chr3:23022037-23022169 4 455::68 453::86 79::206 124::235 0.692669097 1 AltEnd

AT2G34310 chr2:14478360-14478487 4 359::20 339::34 21::385 38::381 0.692687479 1 Cassette

AT1G01020 chr1:8571-8594 1 80::16 117::55 80::49 107::62 0.692798746 1 A5SS

AT1G54100 chr1:20198855-20198962 1 167::63 101::39 50::190 37::124 0.692920775 1 IR

AT2G42365 chr2:17642685-17642771 2 2::28 1::57 21::8 23::17 0.693038975 1 IR

AT5G23550 chr5:7940480-7940485 4 6::96 22::100 67::115 74::118 0.693163406 1 A3SS

AT2G46830 chr2:19247828-19247921 8 4::2036 24::2082 1062::991 1075::986 0.69362322 1 IR

AT1G06010 chr1:1823385-1823410 2 18::351 21::356 359::403 357::378 0.693858556 1 A5SS

AT4G23510 chr4:12268212-12268292 3-4 0::10 0::21 11::13 71::22 0.694117096 1 AltEnd

AT4G31430 chr4:15249755-15249785 5 262::15 201::19 232::198 186::191 0.694246061 1 A3SS

AT4G24770 chr4:12767559-12767590 1 53::18189 32::15999 9877::8076 8726::6910 0.694281668 1 IR

AT5G45030 chr5:18175700-18175794 6 395::81 401::96 86::189 93::210 0.694446727 1 IR

AT2G16940 chr2:7345079-7345197 9 55::267 23::245 337::434 373::510 0.694573465 1 A3SS

AT1G50300 chr1:18631542-18631558 2 26::73 43::70 37::96 54::108 0.694622718 1 A5SS

AT3G50670 chr3:18827553-18828349 6 81::143 102::175 111::306 135::303 0.694860625 1 A5SS

AT1G05870 chr1:1772141-1772309 2 87::182 152::187 54::592 68::580 0.695362131 1 IR

AT2G01520 chr2:236021-236178 1 137::14425 2::7508 13704::6106 7312::2946 0.695498922 1 A5SS

AT5G64460 chr5:25775106-25775115 4 39::199 41::195 295::450 282::416 0.695803588 1 A3SS

AT1G01060 chr1:37302-37373 2 31::1019 20::764 138::741 130::574 0.695827713 1 A5SS

AT1G63770 chr1:23664166-23664272 2 114::174 63::100 141::231 82::128 0.695837562 1 Cassette

AT5G61020 chr5:24559241-24559247 3 163::350 138::376 424::637 418::593 0.696106572 1 A3SS

AT5G36180 chr5:14241447-14241496 12 9::114 19::124 70::53 71::55 0.696346201 1 Cassette

AT4G19670 chr4:10701365-10701424 2 20::20 12::13 48::142 31::118 0.696407127 1 A3SS

AT2G43490 chr2:18058045-18058057 2 33::3 9::2 25::27 10::16 0.696430486 1 A5SS

AT1G78870 chr1:29651845-29651949 6 586::100 764::175 97::608 136::825 0.696510115 1 IR

AT5G14565 chr5:4695628-4695707 2 132::766 118::618 398::676 320::659 0.696528362 1 IR

AT2G26800 chr2:11430792-11430799 5 20::479 25::461 361::507 352::467 0.696773343 1 A3SS

AT5G43630 chr5:17527241-17527256 2 33::39 14::35 42::115 35::119 0.697017731 1 A3SS

AT4G38200 chr4:17916902-17916912 3 7::39 20::25 19::60 32::68 0.697059337 1 A3SS

AT1G13820 chr1:4736214-4736311 3 15::570 11::993 253::322 513::498 0.697123484 1 Cassette

AT3G58180 chr3:21545525-21545546 5 252::1 265::14 209::262 258::322 0.697430432 1 A3SS

AT2G17640 chr2:7669064-7669130 6 80::9 99::8 45::151 62::166 0.697440765 1 A3SS

AT4G05440 chr4:2757870-2758076 1 17::110 3::177 62::74 100::104 0.697535991 1 IR

AT1G24070 chr1:8518615-8518629 4 12::30 8::19 21::32 17::35 0.697689439 1 A5SS

AT2G31900 chr2:13563179-13563238 29 8::4 10::9 5::18 8::22 0.698882577 1 Cassette

AT2G05510 chr2:2016386-2016408 2 9::2249 6::2711 1175::1244 1353::1465 0.69901125 1 IR

AT1G64430 chr1:23936363-23936695 12 148::34 282::114 21::130 64::219 0.699598458 1 IR

AT3G54380 chr3:20135847-20135855 4 7::94 30::105 76::128 94::154 0.699691988 1 A3SS

AT1G07880 chr1:2434621-2434727 4 20::9 20::30 5::35 17::49 0.699702676 1 IR

AT5G45680 chr5:18531413-18531418 2 14::1291 25::1177 1101::1258 1056::1237 0.69981543 1 A5SS

AT3G59330 chr3:21926248-21926305 7 4::53 0::117 33::34 75::56 0.699863179 1 Cassette

AT1G76020 chr1:28533204-28533284 1-2 274::8 272::22 25::213 46::240 0.699984787 1 AltStart

AT1G27000 chr1:9376401-9376412 13 637::11 843::4 436::364 601::559 0.699997876 1 A3SS

AT5G08185 chr5:2635215-2635226 2 22::57 24::90 119::120 195::233 0.700112939 1 A3SS

AT1G22430 chr1:7919749-7919832 3 40::454 43::488 243::281 270::334 0.700318899 1 IR

AT5G66050 chr5:26413838-26414155 1 129::17 149::25 12::162 23::153 0.700362284 1 IR

AT1G76570 chr1:28730342-28730380 5 10::479 6::461 423::397 461::441 0.700654707 1 A5SS

AT3G45100 chr3:16505991-16506002 5 61::25 129::45 58::118 107::175 0.700689924 1 A3SS

AT1G29120 chr1:10177782-10177861 9 329::6 411::32 19::298 35::440 0.700738178 1 IR

AT1G60505 chr1:22295134-22295139 3 22::15 21::49 45::30 72::61 0.701061469 1 A3SS

AT5G66070 chr5:26422518-26422591 3 57::3 51::18 5::62 12::77 0.701757079 1 IR

AT5G43920 chr5:17674807-17674902 3 2::164 22::242 96::84 134::141 0.701786367 1 IR

AT1G04425 chr1:1195053-1195439 2 20::123 21::41 29::149 37::114 0.702159549 1 A3SS

AT5G45050 chr5:18179284-18179369 4 0::70 1::55 43::38 32::58 0.702460991 1 IR

AT2G43465 chr2:18048187-18048194 8 8::11 24::18 26::34 43::69 0.702621743 1 A3SS

AT1G70850 chr1:26715536-26715706 3 203::13584 161::10432 11325::6222 9185::5546 0.703220996 1 A3SS

AT5G65770 chr5:26315745-26315826 9 2::58 11::81 29::37 39::44 0.703537904 1 IR

AT5G04810 chr5:1391379-1391399 3 295::44 245::53 255::321 219::313 0.703673278 1 A3SS

AT4G31170 chr4:15155467-15155576 1 23::163 17::107 45::87 56::62 0.703714688 1 A5SS

AT3G59490 chr3:21984811-21984819 7 30::127 38::129 106::84 105::91 0.703861089 1 A3SS

AT3G28070 chr3:10448169-10448282 1 138::22 78::33 17::143 20::148 0.70391224 1 IR

AT3G51040 chr3:18953094-18953642 1 93::10 83::36 14::94 25::97 0.704168314 1 IR

AT2G33620 chr2:14234220-14234462 2 64::44 73::29 63::315 57::330 0.704858115 1 AltStart

AT5G35570 chr5:13751862-13751869 5 46::15 60::9 37::69 41::56 0.70521931 1 A3SS

AT4G34020 chr4:16300445-16300550 2 7::124 3::103 130::141 118::139 0.705321707 1 A3SS

AT1G16840 chr1:5762958-5762965 3 305::232 279::288 256::235 265::285 0.705370623 1 A3SS

AT2G43780 chr2:18137410-18137571 1 316::7 488::0 236::42 309::29 0.706001797 1 A5SS

AT4G09760 chr4:6149501-6149691 4 188::33 127::46 29::244 40::224 0.706180276 1 IR

AT2G31370 chr2:13379035-13379222 1 28::58 10::46 45::46 36::27 0.706963179 1 IR

AT2G05440 chr2:1994987-1995067 4 0::119 4::216 23::7168 45::8061 0.706967289 1 AltEnd

AT1G75150 chr1:28204738-28204774 11 6::14 5::25 10::13 30::19 0.70712033 1 A3SS

AT3G14880 chr3:5007362-5007383 2 2::15 9::5 6::21 12::15 0.707127369 1 A3SS

AT3G56940 chr3:21077702-21077750 4 203::23211 204::19738 23909::19740 20031::14523 0.707358397 1 A5SS

AT4G26110 chr4:13235010-13235027 11 3::2023 21::2458 923::551 1123::740 0.707359599 1 Cassette

AT1G19400 chr1:6714086-6714166 1-2 164::19 66::11 27::365 29::304 0.707531401 1 AltStart

AT3G62800 chr3:23227169-23227174 2 13::49 20::47 36::77 42::76 0.707623088 1 A3SS

AT5G03240 chr5:772291-772520 2 14::9560 33::11421 4927::3487 5865::4388 0.707998917 1 IR

AT4G31580 chr4:15307482-15307516 4 13::2271 33::2423 1186::1418 1271::1475 0.708009793 1 IR

AT2G05830 chr2:2229830-2229911 1 246::16 236::35 34::136 42::139 0.708195761 1 IR

AT1G49760 chr1:18419871-18419935 9 0::412 0::534 137::753 173::1146 0.708409376 1 IR

AT4G27050 chr4:13577652-13577660 3 10::39 9::67 40::61 58::86 0.708770137 1 A3SS

AT2G46340 chr2:19023087-19023221 6 16::520 6::633 312::342 364::405 0.709097441 1 IR

AT2G44920 chr2:18526165-18526278 6 868::71 982::98 77::842 107::940 0.709326522 1 IR

AT4G01150 chr4:494358-494449 3 12268::24 13393::57 735::12156 818::12710 0.709499187 1 IR

AT4G21800 chr4:11572833-11572927 1 75::0 50::3 11::35 7::20 0.709646143 1 A5SS

AT1G04300 chr1:1153124-1153234 3 46::0 35::12 3::40 11::46 0.709814094 1 IR

AT4G11570 chr4:7004412-7004657 1 17::749 3::611 431::762 338::713 0.709820496 1 IR

AT3G26740 chr3:9828148-9828159 2 1667::13 670::5 1615::2179 639::970 0.71020972 1 A3SS

AT3G15351 chr3:5177293-5177386 4 131::22 139::25 20::101 21::120 0.710244046 1 IR

AT2G39800 chr2:16602269-16602372 3 33::6064 18::1351 3039::2848 677::691 0.710464613 1 Cassette

AT4G36690 chr4:17294430-17294603 12 261::132 417::177 198::535 267::807 0.710569514 1 A3SS

AT3G13062 chr3:4185971-4186008 4 37::353 20::318 396::433 394::386 0.710583965 1 A5SS

AT1G80000 chr1:30093078-30093161 1 25::109 41::62 66::107 43::106 0.710884562 1 IR

AT3G13570 chr3:4430483-4430643 3-4 74::7 512::159 18::135 156::817 0.710953477 1 Cassette

AT2G29525 chr2:12640230-12640531 11 21::17 41::44 11::31 15::43 0.711014474 1 IR

AT5G24350 chr5:8311708-8311788 15 2::13 2::19 12::37 34::45 0.711093412 1 AltEnd

AT1G79870 chr1:30045193-30045216 1 33::706 40::534 682::695 537::560 0.711298119 1 A5SS

AT5G24735 chr5:8469015-8469020 1 452::1033 504::1049 1342::1187 1363::1165 0.711466535 1 A5SS

AT2G01620 chr2:278918-278924 3 282::65 308::41 202::334 211::304 0.711605884 1 A3SS

AT3G56930 chr3:21075988-21075993 6 39::11 98::12 28::12 57::28 0.711732187 1 A3SS

AT2G46572 chr2:19127377-19127457 2-3 6::5 9::21 17::23 22::41 0.711844734 1 AltStart

AT3G29185 chr3:11156072-11156184 3 228::64 257::128 25::294 35::406 0.712068919 1 IR

AT5G64240 chr5:25696860-25696948 3 561::60 531::51 59::577 53::535 0.712145199 1 IR

AT3G47680 chr3:17577154-17577183 3 43::35 59::28 20::86 24::110 0.712646953 1 Cassette

AT5G24810 chr5:8519452-8519469 11 78::16 73::6 30::63 26::80 0.712758002 1 A3SS

AT5G12170 chr5:3934161-3934175 5 7::85 33::99 85::111 96::135 0.713573095 1 A5SS

AT1G04910 chr1:1385647-1385765 10 0::107 1::154 53::67 98::68 0.713613376 1 Cassette

AT5G24670 chr5:8450186-8450202 9 144::6 280::30 136::201 263::388 0.713693579 1 A3SS

AT1G76930 chr1:28895998-28896027 1-2 114::224 92::146 1723::1603 1811::1982 0.714034976 1 Cassette

AT4G25770 chr4:13119032-13119096 4 67::16 53::8 76::73 68::89 0.714123664 1 A5SS

AT5G20520 chr5:6945378-6945398 4 405::31 475::18 398::396 475::451 0.714128342 1 A5SS

AT1G77580 chr1:29144568-29144662 4 10::21 31::9 13::35 10::44 0.714538205 1 IR

AT2G02960 chr2:863995-864020 2 18::23 42::21 89::156 158::246 0.714600922 1 A3SS

AT3G15395 chr3:5200994-5201005 3 80::123 74::150 196::409 214::490 0.714687511 1 A3SS

AT1G79529 chr1:29916286-29916370 3 1212::23 1557::14 79::1075 96::1467 0.714788271 1 IR

AT5G46390 chr5:18818500-18818592 8 82::75 84::99 24::151 31::182 0.71481807 1 IR

AT4G36690 chr4:17295297-17295398 10 65::830 48::1062 481::499 569::637 0.715052552 1 IR

AT4G13100 chr4:7636846-7636855 5 37::448 73::498 322::192 356::215 0.715185368 1 A3SS

AT2G35660 chr2:14989002-14989010 3 104::158 111::104 148::258 125::223 0.715324335 1 A3SS

AT1G16000 chr1:5497101-5497117 3 215::94 208::72 144::247 130::294 0.715334175 1 A5SS

AT5G19030 chr5:6357877-6357896 3 241::26 221::10 83::244 64::218 0.715585032 1 A3SS

AT1G30330 chr1:10689901-10689907 3 13::16 11::21 12::55 14::75 0.715758596 1 A3SS

AT1G27370 chr1:9507023-9508147 1 15::26 13::103 15::45 28::92 0.715893693 1 IR

AT5G17230 chr5:5662456-5662466 2 1026::10 825::6 31::1831 24::1859 0.715951741 1 AltStart

AT2G14530 chr2:6188024-6188029 2 4::56 27::73 45::89 56::124 0.716004683 1 A3SS

AT5G09230 chr5:2873271-2873344 10 110::15 131::41 17::114 32::146 0.716687555 1 IR

AT1G67080 chr1:25046200-25046213 2 329::11 350::17 248::311 267::338 0.716813912 1 A5SS

AT4G24900 chr4:12816504-12816513 5 10::7 30::10 18::39 41::64 0.716847302 1 A3SS

AT1G19400 chr1:6713787-6714169 1 164::119 66::78 73::323 59::247 0.716964528 1 IR

AT4G39030 chr4:18189616-18189622 13 807::11 825::8 78::956 84::980 0.716994068 1 A5SS

AT5G38360 chr5:15332298-15332305 7 7::72 24::103 51::98 80::152 0.717251812 1 A3SS

AT3G04485 chr3:1199567-1199632 3-4 5::31 11::24 21::25 23::30 0.717263955 1 Cassette

AT3G56040 chr3:20796377-20796387 3 28::313 34::285 267::277 250::294 0.717399755 1 A3SS

AT1G62610 chr1:23180330-23180346 4 104::12 90::22 101::99 113::156 0.71754675 1 A5SS

AT4G02430 chr4:1071274-1071285 11 25::5 20::12 55::75 90::131 0.717720516 1 A3SS

AT5G35560 chr5:13744780-13744787 9 6::59 9::65 56::59 60::68 0.71827343 1 A3SS

AT3G26430 chr3:9672874-9672896 3 49::3189 110::3150 1630::1424 1571::1350 0.718325799 1 IR

AT2G39470 chr2:16476534-16476558 2 257::1435 279::1462 1488::1671 1496::1611 0.718724344 1 A5SS

AT4G24972 chr4:12838307-12838330 2 380::7 357::8 39::413 37::372 0.718820931 1 A5SS

AT5G55060 chr5:22342491-22342531 2 10::103 3::126 55::58 81::62 0.719318718 1 Cassette

AT5G58140 chr5:23525667-23525674 4 692::16 496::8 438::688 328::524 0.719540889 1 A3SS

AT3G21390 chr3:7532872-7532906 4 31::243 48::331 277::300 359::340 0.719645297 1 A5SS

AT4G10120 chr4:6316320-6316375 6 15::340 8::178 364::373 203::215 0.719658996 1 A5SS

AT4G27000 chr4:13556560-13556637 2-3 1579::79 1579::108 38::1720 66::1667 0.719916069 1 Cassette

AT2G24755 chr2:10548338-10548370 3 44::46 61::34 44::81 49::113 0.720324783 1 A3SS

AT1G27650 chr1:9615137-9615196 2 650::0 655::0 755::662 741::760 0.720412378 1 A3SS

AT5G16140 chr5:5271078-5271083 4 90::18 90::13 40::128 45::134 0.720737414 1 A3SS

AT4G00770 chr4:331515-331715 1 26::30 46::13 14::47 12::54 0.720891924 1 IR

AT1G66940 chr1:24973602-24973730 2 127::15 291::79 18::182 44::372 0.720898147 1 IR

AT3G09440 chr3:2903245-2903336 2 0::135 0::202 53::1368 82::1631 0.720954413 1 IR

AT4G32590 chr4:15721506-15721608 1 26::848 12::892 858::595 892::607 0.720989193 1 A5SS

AT4G14716 chr4:8431686-8431766 1-2 473::7 447::16 6::391 13::454 0.721291389 1 AltStart

AT4G04750 chr4:2421246-2421289 13 9::135 13::181 78::68 93::92 0.721361235 1 Cassette

AT5G42870 chr5:17187282-17187297 3 68::24 102::28 66::115 98::116 0.721795034 1 A5SS

AT2G38810 chr2:16220826-16220906 2 7::88 1::88 34::248 36::280 0.721912942 1 AltStart

AT1G30200 chr1:10626650-10626661 2 95::112 158::152 84::167 157::272 0.722188425 1 A3SS

AT5G51080 chr5:20770625-20770686 2 3::16 5::17 20::28 32::28 0.722188997 1 A3SS

AT5G21222 chr5:7212007-7212053 11 139::11 129::39 167::185 133::201 0.722332976 1 A3SS

AT1G23110 chr1:8197747-8197752 4 35::73 41::159 98::281 202::441 0.72241373 1 A3SS

AT4G32420 chr4:15651496-15651603 6 67::14 45::13 9::140 18::188 0.722521105 1 AltStart

AT3G48820 chr3:18102632-18102659 2 19::21 11::20 45::70 41::64 0.722606293 1 A3SS

AT1G78510 chr1:29536372-29536386 4 543::15 452::22 189::580 175::515 0.722874469 1 A3SS

AT2G27030 chr2:11532144-11532687 1 1311::38 1293::82 35::407 39::459 0.723488485 1 IR

AT5G60120 chr5:24210074-24210095 7 6::127 18::145 129::130 149::160 0.72349111 1 A5SS

AT1G69010 chr1:25943424-25943429 6 26::191 15::237 220::230 251::264 0.723573722 1 A5SS

AT5G53350 chr5:21646803-21646822 12 6::555 10::533 110::487 101::470 0.723789621 1 A3SS

AT1G06500 chr1:1989526-1989668 1 59::48 62::45 33::53 46::47 0.723798133 1 IR

AT1G70610 chr1:26622786-26622797 2 178::30 91::15 90::190 51::119 0.723987464 1 A5SS

AT1G19350 chr1:6688644-6688775 1 768::1071 817::922 396::2277 360::2139 0.723987823 1 IR

AT2G44050 chr2:18224542-18224560 2 847::20 841::10 666::754 645::700 0.724029575 1 A3SS

AT1G56350 chr1:21096080-21096114 2 21::1 25::14 8::19 17::46 0.724130455 1 A5SS

AT5G45430 chr5:18411500-18411506 13 1224::21 1527::21 941::878 1181::1149 0.724255484 1 A3SS

AT5G47310 chr5:19201262-19201270 2 228::23 245::8 248::361 249::374 0.724300768 1 A3SS

AT5G13360 chr5:4283694-4283699 1 21::71 26::48 81::25 68::26 0.724568785 1 A5SS

AT5G49730 chr5:20203094-20203193 5 30::1320 7::968 712::739 534::547 0.724773006 1 IR

AT3G22170 chr3:7825421-7825455 2 1::57 1::39 73::97 51::97 0.724779876 1 A3SS

AT2G24755 chr2:10547911-10547916 4 21::74 10::103 33::81 47::104 0.724790186 1 A3SS

AT1G54390 chr1:20305458-20305555 5 10::149 8::381 82::106 216::212 0.725069518 1 IR

AT1G02910 chr1:656495-656505 6 5::333 13::403 349::353 425::448 0.725320017 1 A3SS

AT5G66160 chr5:26445796-26445801 3 175::21 175::13 203::225 190::280 0.725333841 1 A3SS

AT2G43180 chr2:17953957-17954032 5 68::10 96::17 16::73 22::93 0.725359141 1 IR

AT1G15410 chr1:5298339-5298386 1 6::10 20::7 12::11 7::22 0.725463829 1 A5SS

AT5G66250 chr5:26468711-26469044 1 41::128 64::111 55::130 62::203 0.725615427 1 IR

AT4G08850 chr4:5637649-5640178 1 140::21 309::18 89::46 137::57 0.725713791 1 A5SS

AT2G42240 chr2:17597954-17598029 5 2::193 15::204 104::96 112::109 0.726311586 1 Cassette

AT1G15750 chr1:5420431-5420539 2 23::16 33::16 10::66 15::53 0.726384691 1 IR

AT4G39260 chr4:18274363-18274393 2 15::8 19::22 3972::4882 7360::8815 0.726493557 1 A5SS

AT4G26510 chr4:13384062-13384106 1 42::20 46::31 16::93 24::114 0.727036667 1 AltStart

AT2G01070 chr2:77363-77400 4-5 192::22 224::18 24::161 25::177 0.727254653 1 Cassette

AT2G38860 chr2:16234914-16234941 2 2::419 3::378 408::479 391::485 0.727274948 1 A3SS

AT5G58350 chr5:23584827-23584995 1 4::26 12::22 12::61 14::41 0.727328988 1 IR

AT3G09600 chr3:2947522-2947539 5 412::44 559::43 380::439 566::751 0.727430767 1 A3SS

AT2G44140 chr2:18257008-18257169 2 69::46 65::85 28::126 46::130 0.72772075 1 IR

AT2G01400 chr2:174988-175158 2 5::138 11::212 62::211 98::231 0.727802605 1 IR

AT3G63445 chr3:23424843-23424899 2-3 11::14 1::18 92::100 104::107 0.727857864 1 A3SS

AT5G25475 chr5:8869619-8869651 2 174::14 251::17 16::143 22::181 0.728419448 1 AltStart

AT4G12770 chr4:7507811-7507817 4 17::118 5::110 132::167 113::147 0.728500709 1 A3SS

AT5G42020 chr5:16808008-16808174 6 0::2503 0::3036 1195::979 1402::1294 0.728626904 1 IR

AT1G79930 chr1:30063940-30063957 10 938::12 1309::6 223::403 276::555 0.728700885 1 A3SS

AT4G27860 chr4:13875112-13875157 5 7::42 22::52 21::72 35::78 0.728941596 1 A3SS

AT4G27050 chr4:13577743-13577906 1 39::11 68::8 36::34 65::57 0.729184574 1 A5SS

AT5G51040 chr5:20751085-20751097 3 775::3 783::11 543::734 541::671 0.729357184 1 A3SS

AT2G36000 chr2:15118116-15118220 1 197::265 248::307 291::646 360::645 0.729712769 1 A5SS

AT5G54080 chr5:21950810-21950818 11 17::3 22::1 11::18 16::35 0.729801958 1 A5SS

AT5G43750 chr5:17580234-17580330 5 9::1341 25::1172 1555::636 1454::623 0.729910426 1 A3SS

AT1G07700 chr1:2379798-2380104 1 439::14 453::36 27::498 37::540 0.730032805 1 IR

AT1G04300 chr1:1152947-1152971 5 30::0 39::0 4::53 16::59 0.730277619 1 A3SS

AT1G07700 chr1:2379729-2379798 1 229::439 307::453 401::212 448::271 0.730288067 1 A5SS

AT2G26590 chr2:11314985-11315020 1 84::57 77::79 71::42 69::56 0.730509511 1 A5SS

AT2G43560 chr2:18074820-18074825 2 109::968 180::1359 686::1004 940::1159 0.730602448 1 A5SS

AT3G18780 chr3:6476680-6476767 3 8065::19 10044::22 749::5114 929::5667 0.730769548 1 IR

AT5G60580 chr5:24356205-24356226 8 32::125 32::156 57::151 61::186 0.731092788 1 A3SS

AT2G36230 chr2:15194938-15194952 3 114::20 109::38 44::114 58::121 0.731248057 1 A3SS

AT3G20720 chr3:7242630-7242702 11 16::64 4::58 35::41 28::34 0.731339798 1 Cassette

AT1G78290 chr1:29459012-29459092 2 7::32 0::21 115::246 94::240 0.731418015 1 AltStart

AT3G20430 chr3:7122083-7122161 3-4 59::20 54::13 15::95 27::109 0.731529498 1 Cassette

AT4G36270 chr4:17161621-17161702 15 27::0 30::0 26::15 34::34 0.731603106 1 A5SS

AT2G30170 chr2:12881100-12881116 3 254::46 314::66 69::262 110::333 0.731609064 1 A3SS

AT3G28660 chr3:10739244-10739347 1 12::16 6::34 8::10 18::11 0.731657242 1 IR

AT1G09060 chr1:2925248-2925264 2 56::14 30::6 32::86 17::75 0.731950099 1 A3SS

AT1G72830 chr1:27406250-27406326 5 5::85 7::139 51::39 78::79 0.732111427 1 IR

AT1G50140 chr1:18575740-18575813 7 23::76 14::78 38::65 43::52 0.732516748 1 Cassette

AT5G39510 chr5:15823425-15823439 6 25::590 28::694 107::333 120::418 0.732538541 1 A3SS

AT1G11840 chr1:3997239-3997255 8 4404::0 4704::29 3465::4192 3663::4666 0.732673804 1 A3SS

AT5G08230 chr5:2644196-2644228 9 13::32 2::50 16::41 9::45 0.73268503 1 A5SS

AT3G25150 chr3:9158588-9158594 7 35::263 69::338 291::312 377::374 0.732740364 1 A3SS

AT1G28210 chr1:9859857-9859893 19 4::22 6::60 11::20 10::37 0.732846589 1 A3SS

AT5G02580 chr5:580239-580327 2 60::12 74::12 14::33 14::39 0.732853158 1 IR

AT1G48420 chr1:17897770-17897816 5 565::4 520::13 60::599 64::564 0.73316618 1 A3SS

AT2G31960 chr2:13589233-13589270 2 46::4 20::7 6::130 8::154 0.73392681 1 AltStart

AT2G20290 chr2:8745581-8745625 27 6::6 12::14 6::10 11::22 0.734027992 1 Cassette

AT4G20070 chr4:10863910-10863950 10 264::8 289::13 243::273 255::287 0.734654428 1 A3SS

AT5G07370 chr5:2330497-2330844 1 207::17 205::25 23::93 24::88 0.734722646 1 A5SS

AT3G27925 chr3:10366726-10366736 8 52::1055 36::1324 802::271 1025::342 0.734839781 1 A3SS

AT1G17280 chr1:5917417-5917422 10 46::443 30::479 396::346 437::404 0.735221406 1 A3SS

AT1G70280 chr1:26468133-26468222 2 164::8 115::13 12::148 14::117 0.735869305 1 IR

AT5G16540 chr5:5403494-5403593 1 243::54 258::91 43::205 60::240 0.73597491 1 IR

AT3G51950 chr3:19280538-19280619 3 31::333 14::227 151::676 109::607 0.73619281 1 IR

AT3G10250 chr3:3170842-3170892 1 67::22 45::23 50::24 36::21 0.736208031 1 A5SS

AT2G05520 chr2:2026650-2026670 3 1153::447 1572::665 6233::9534 8006::13635 0.736223793 1 Cassette

AT4G19200 chr4:10499355-10499428 2 30::5218 0::5208 2685::2323 2661::2287 0.736237336 1 IR

AT3G22950 chr3:8137525-8137542 3 17::153 26::120 145::241 118::199 0.736249023 1 A3SS

AT4G05320 chr4:2719240-2719345 3 2135::66 2093::56 16391::14977 16690::14024 0.73631533 1 A5SS

AT1G28100 chr1:9803970-9804026 11 5::17 25::24 81::132 153::203 0.736396516 1 A5SS

AT1G28670 chr1:10075253-10075269 4 14::362 5::390 309::402 354::554 0.736590726 1 A3SS

AT3G60600 chr3:22401575-22401913 6 1390::27 1498::61 54::1162 74::1327 0.736646373 1 IR

AT5G18400 chr5:6094919-6094991 1 8::71 17::67 33::46 29::51 0.736811606 1 IR

AT5G55550 chr5:22503846-22503927 1 15::3 14::6 4::38 7::36 0.736847956 1 IR

AT5G11450 chr5:3654616-3654622 2 1096::14 1028::1 1142::1027 1100::990 0.737400968 1 A3SS

AT2G23985 chr2:10206104-10206113 4 65::49 108::44 95::66 139::99 0.737471207 1 A3SS

AT3G51830 chr3:19223137-19223151 14 73::28 73::61 92::156 83::160 0.73750706 1 A3SS

AT4G34610 chr4:16532549-16532573 2 2::51 0::35 84::145 60::173 0.737625161 1 A3SS

AT2G22720 chr2:9659075-9659095 7 43::112 70::113 76::367 102::418 0.737634209 1 A3SS

AT3G30390 chr3:11978927-11979037 2 700::187 618::205 148::990 166::880 0.737731822 1 Cassette

AT1G10910 chr1:3641241-3641251 4 98::23 116::21 73::121 85::146 0.737828301 1 A3SS

AT4G15955 chr4:9044498-9044699 3 18::120 0::111 52::69 48::72 0.737839069 1 Cassette_multi

AT1G69750 chr1:26235855-26236254 2 15::174 7::190 89::138 115::152 0.737854682 1 IR

AT2G32040 chr2:13636420-13636434 6 16::85 34::121 58::159 99::217 0.737881759 1 A3SS

AT5G26980 chr5:9488361-9488446 8 5::39 24::70 24::41 32::58 0.737898795 1 IR

AT1G04550 chr1:1240998-1241057 2 1::390 16::493 15::392 38::475 0.738108892 1 A3SS

AT2G46572 chr2:19128859-19128939 1 20::12 58::19 10::26 16::48 0.738319211 1 IR

AT1G34570 chr1:12656236-12656295 2 28::138 18::138 84::115 96::112 0.738722524 1 Cassette

AT3G58170 chr3:21542915-21542927 4 460::28 434::17 201::495 170::453 0.739012512 1 A3SS

AT4G38260 chr4:17939168-17939176 before first 19::21 15::35 42::68 58::78 0.739012834 1 A3SS

AT2G46540 chr2:19111158-19111182 2 924::25 1177::18 993::1157 1191::1283 0.739324446 1 A5SS

AT4G04350 chr4:2129664-2129687 6 24::34 29::15 28::83 30::73 0.739442148 1 A3SS

AT5G19400 chr5:6544362-6544381 7 34::376 32::491 33::471 37::603 0.739601382 1 A3SS

AT1G74960 chr1:28156132-28156192 1 0::440 0::250 241::664 126::474 0.739776757 1 IR

AT2G32760 chr2:13892578-13892600 2 18::24 25::15 33::42 42::51 0.739839252 1 A3SS

AT1G76460 chr1:28684846-28684851 9 958::34 892::56 95::1050 100::919 0.740121874 1 A3SS

AT1G55500 chr1:20722292-20722308 8 171::1 274::17 35::241 83::324 0.74020566 1 A5SS

AT4G14880 chr4:8520244-8520332 1 4::1869 9::1378 22::467 23::343 0.740353517 1 A5SS

AT4G18740 chr4:10304268-10304275 2 131::33 107::63 65::169 72::203 0.740504736 1 A3SS

AT2G22990 chr2:9787071-9787190 4 46::2711 27::2375 1477::1306 1254::1121 0.741029068 1 Cassette

AT5G53460 chr5:21709630-21709710 20 55::2646 56::3577 1386::916 1921::1138 0.741092278 1 IR

AT5G10140 chr5:3173825-3173891 8 66::0 44::0 5::50 4::14 0.74109419 1 A3SS

AT3G59330 chr3:21928036-21928068 1 12::5 23::11 26::13 38::16 0.741116089 1 A5SS

AT1G14030 chr1:4806886-4806891 2 56::15 57::20 26::97 48::124 0.741138551 1 A3SS

AT1G72280 chr1:27212524-27212562 7 15::15 19::9 19::24 24::36 0.741352536 1 A5SS

AT1G18180 chr1:6257695-6257701 10 17::42 14::27 9::24 12::25 0.741524644 1 A3SS

AT4G01897 chr4:820767-820847 2-3 211::17 284::17 21::149 18::197 0.741572773 1 AltStart

AT2G20950 chr2:9004308-9004314 7 35::111 59::97 116::180 128::190 0.741575774 1 A3SS

AT4G08685 chr4:5550934-5550940 2 1459::12 1327::11 1219::1157 1122::1237 0.741611936 1 A3SS

AT3G07215 chr3:2296814-2297717 1 3::63 18::99 30::38 50::85 0.741675729 1 IR

AT2G22650 chr2:9626101-9626124 6 32::36 32::24 41::74 45::136 0.741680536 1 A3SS

AT4G29930 chr4:14644982-14645070 3 3::89 6::59 52::73 29::47 0.741683662 1 IR

AT5G60410 chr5:24300667-24300753 17 28::536 26::864 296::254 478::384 0.741712241 1 IR

AT1G02120 chr1:398905-398966 15 5::298 12::509 139::125 246::208 0.742013507 1 Cassette

AT4G26140 chr4:13243615-13243722 16 52::19 39::31 11::72 12::60 0.74227742 1 IR

AT5G46110 chr5:18697883-18697890 1 8563::237 5789::293 4995::5518 3585::3829 0.742292423 1 A5SS

AT5G16110 chr5:5261792-5261877 2 1506::8 1374::15 1621::1618 1587::1563 0.742331134 1 A5SS

AT4G24230 chr4:12567138-12567144 4 227::47 304::35 100::89 109::117 0.743030841 1 A3SS

AT2G39950 chr2:16679173-16679426 3-4 23::64 19::32 44::123 33::107 0.743161496 1 Cassette_multi

AT3G55280 chr3:20501435-20501451 5 1906::14 2259::3 1699::932 1944::1030 0.743328983 1 A3SS

AT3G48115 chr3:17772842-17772926 1 14::20 43::30 15::377 25::420 0.743712337 1 AltStart

AT4G08980 chr4:5758590-5758690 3 189::0 222::5 73::497 69::648 0.743858574 1 AltStart

AT5G34850 chr5:13110300-13110321 4 985::16 895::4 1193::1166 1009::861 0.744787849 1 A5SS

AT4G18740 chr4:10303631-10303725 3 153::98 145::214 59::116 113::159 0.744897225 1 IR

AT5G20165 chr5:6806366-6806434 4 572::0 634::0 15::491 38::500 0.744988271 1 Cassette

AT1G10670 chr1:3535578-3535774 1 606::23 565::16 30::342 39::306 0.74529992 1 IR

AT1G64720 chr1:24048368-24048390 1 0::25153 25::24119 13093::11667 12485::11299 0.745324083 1 IR

AT4G13575 chr4:7892973-7892987 5 264::2 435::24 63::234 118::394 0.74547311 1 A3SS

AT4G32920 chr4:15893311-15893324 7 8::53 15::42 20::62 26::63 0.745613196 1 A5SS

AT5G59610 chr5:24014318-24014350 3 106::47 143::35 23::169 24::205 0.745757334 1 Cassette

AT2G31900 chr2:13561874-13561924 35 9::25 24::31 25::19 33::40 0.745829798 1 A3SS

AT5G03345 chr5:814584-814624 3 11::650 5::792 633::611 786::734 0.745924533 1 A5SS

AT4G30910 chr4:15045018-15045315 1 13::67 7::100 53::94 75::146 0.746094385 1 AltStart

AT3G15980 chr3:5411965-5412008 24 147::86 192::115 177::255 247::326 0.746303702 1 A5SS

AT3G58900 chr3:21773965-21774055 1 21::63 43::104 30::52 54::66 0.746447779 1 IR

AT5G54130 chr5:21964063-21964098 6 106::48 122::59 60::188 81::175 0.746478901 1 A5SS

AT5G46470 chr5:18844911-18844921 4 36::7 67::9 26::57 48::68 0.74653651 1 A3SS

AT1G49140 chr1:18177672-18177730 2 17::3063 0::3299 1512::1201 1620::1292 0.746807386 1 Cassette

AT1G41830 chr1:15604719-15604733 6 10::807 10::660 686::890 564::747 0.746906689 1 A3SS

AT1G23080 chr1:8182206-8182218 1 180::375 176::442 196::404 201::368 0.747236154 1 A5SS

AT4G34240 chr4:16391981-16392068 9 435::48 520::28 64::512 64::533 0.747395904 1 IR

AT4G24500 chr4:12658904-12658992 2 14::153 8::177 75::84 94::77 0.747417767 1 IR

AT5G58960 chr5:23806778-23806863 2 122::32 116::23 28::240 20::230 0.747645797 1 IR

AT5G27970 chr5:10013405-10013441 39 84::17 114::41 26::107 58::146 0.747736844 1 A5SS

AT5G51150 chr5:20789817-20789827 3 3::79 13::70 63::145 75::136 0.747869514 1 A3SS

AT1G31870 chr1:11437252-11437269 2 15::5 14::8 19::33 15::35 0.748109641 1 A3SS

AT3G44750 chr3:16298779-16298788 4 766::0 1070::45 646::651 872::919 0.748152176 1 A3SS

AT1G76320 chr1:28633891-28634000 3 2::94 13::107 55::60 62::80 0.748523836 1 IR

AT5G10490 chr5:3303981-3304104 2 5::78 3::52 74::63 65::67 0.748579845 1 A3SS

AT4G18370 chr4:10149877-10149882 3 25::281 26::289 238::283 257::272 0.748592262 1 A5SS

AT1G55000 chr1:20516205-20516272 2 29::534 21::733 286::366 385::407 0.749254207 1 IR

AT1G18720 chr1:6459296-6459751 1 59::979 26::915 502::555 513::602 0.749595318 1 AltStart

AT4G38545 chr4:18024286-18024342 3 154::0 167::0 199::215 208::307 0.749925019 1 A5SS

AT4G39300 chr4:18287804-18288111 3 60::476 108::570 103::346 132::411 0.749985441 1 AltEnd

AT3G18380 chr3:6312247-6312256 4 136::30 139::16 89::155 91::135 0.750176501 1 A5SS

AT1G70780 chr1:26695741-26695810 1 1774::19 2578::0 1827::1323 2616::1762 0.750184367 1 A5SS

AT4G30100 chr4:14715511-14715612 1-2 42::70 44::88 44::92 42::87 0.750213875 1 Cassette

AT2G22990 chr2:9786941-9786951 3 1385::21 1150::24 558::1224 455::997 0.750392514 1 A5SS

AT5G47455 chr5:19251196-19251251 2 149::15 235::17 41::212 61::334 0.750393994 1 A5SS

AT2G29400 chr2:12614361-12614452 2 14::1593 1::2122 806::972 1068::1338 0.750416738 1 IR

AT3G59300 chr3:21920405-21920503 7 20::253 24::428 133::193 189::242 0.750666344 1 Cassette

AT2G30710 chr2:13087814-13087836 5 10::100 7::101 84::111 92::125 0.750947246 1 A3SS

AT1G23860 chr1:8428605-8428617 4 747::9 881::2 739::671 877::778 0.751145359 1 A5SS

AT2G47630 chr2:19534889-19534905 2 5::200 10::117 203::203 139::166 0.751248372 1 A5SS

AT1G76440 chr1:28683009-28683044 2 65::172 68::113 203::210 133::188 0.751277536 1 A3SS

AT3G61850 chr3:22896704-22896789 2 19::60 49::34 29::36 26::38 0.751413939 1 IR

AT2G20570 chr2:8857143-8857151 6 1808::25 2771::26 1700::2027 2642::3273 0.751701527 1 A3SS

AT4G00830 chr4:352196-352379 1 300::13 254::18 19::146 23::144 0.751710016 1 A5SS

AT5G23860 chr5:8044575-8044736 3 0::377 0::376 284::748 255::673 0.751827421 1 IR

AT1G08650 chr1:2753183-2753477 3 831::0 1419::0 624::145 1070::240 0.752273105 1 A3SS

AT4G23470 chr4:12249491-12249818 2 626::10 781::40 25::666 43::838 0.752353318 1 IR

AT1G44920 chr1:16983574-16983593 4 1103::6 925::19 218::1162 189::1022 0.752456359 1 A3SS

AT3G45600 chr3:16734849-16734882 1-2 689::13 710::21 14::498 18::447 0.752481559 1 Cassette

AT4G24480 chr4:12652344-12652375 4-5 20::13 31::11 11::44 16::37 0.752559804 1 Cassette

AT2G24270 chr2:10327134-10327330 10 121::1848 107::2027 1593::2233 1748::2397 0.752855956 1 IR

AT1G15340 chr1:5276725-5276882 2 0::954 0::741 512::584 397::573 0.752964583 1 IR

AT5G02680 chr5:605914-605995 2 14::13 13::31 27::26 64::46 0.753207446 1 A5SS

AT5G11170 chr5:3554101-3554237 4 113::952 153::895 146::819 165::783 0.753316178 1 A3SS

AT5G18420 chr5:6108803-6108812 2 0::153 3::70 135::123 71::91 0.753456397 1 A3SS

AT3G63460 chr3:23434944-23434950 10 123::21 134::8 67::163 69::141 0.754067069 1 A5SS

AT2G31900 chr2:13561957-13562021 34 9::25 24::31 11::20 24::30 0.754443024 1 A5SS

AT2G11910 chr2:4807166-4807277 1 366::985 367::912 498::1248 447::1208 0.754708852 1 IR

AT1G09195 chr1:2970721-2971078 1 1::21 8::23 12::18 13::21 0.754792724 1 IR

AT1G27300 chr1:9483702-9483733 2 13::131 5::168 31::195 29::267 0.755100673 1 A3SS

AT5G13220 chr5:4219992-4220045 4 20::8 31::13 14::42 15::42 0.755104877 1 A5SS

AT1G63610 chr1:23585005-23585018 3 16::284 25::217 99::280 83::232 0.755381624 1 A5SS

AT5G43990 chr5:17698504-17698518 2 15::11 3::17 33::53 18::24 0.756075116 1 A3SS

AT3G62620 chr3:23161266-23161387 6 23::7 69::8 7::38 9::57 0.756554433 1 IR

AT1G22140 chr1:7814937-7815129 3 325::2 363::2 147::239 187::257 0.756683849 1 A3SS

AT5G19670 chr5:6648241-6648254 3 5::31 13::26 16::35 18::52 0.756842109 1 A3SS

AT1G53090 chr1:19783570-19783684 1 5::36 5::13 14::51 7::40 0.756967315 1 IR

AT1G52510 chr1:19561527-19561687 12 32::570 93::790 222::94 334::133 0.757055327 1 IR

AT2G47020 chr2:19319816-19320042 11 64::838 41::1259 421::581 643::783 0.757282414 1 IR

AT4G18890 chr4:10353415-10353495 1-2 57::7 55::8 9::83 8::99 0.757623783 1 AltStart

AT4G15393 chr4:8806711-8806718 7 83::3 193::1 104::82 204::203 0.757625529 1 A3SS

AT2G03680 chr2:1120922-1121195 2 2201::4 1668::11 6::2451 15::2518 0.757758776 1 AltStart

AT5G16880 chr5:5549614-5549638 2 30::239 13::244 68::424 54::383 0.758286458 1 A3SS

AT5G66480 chr5:26545156-26545242 3 9::115 22::103 72::62 63::76 0.758541328 1 IR

AT3G16430 chr3:5582692-5582708 4 33::895 51::841 165::596 164::672 0.758698595 1 A3SS

AT4G36050 chr4:17054850-17054937 1 29::6 25::2 5::31 4::27 0.758890994 1 IR

AT1G20920 chr1:7289288-7289309 3 6::46 11::55 111::51 154::68 0.759267363 1 A5SS

AT5G17710 chr5:5841388-5841394 1 933::5 981::12 666::925 711::890 0.759343261 1 A5SS

AT3G02600 chr3:552437-552529 4 67::172 103::179 126::110 162::152 0.759684057 1 Cassette

AT4G27050 chr4:13577660-13577743 1 39::9 67::10 9::42 11::67 0.75970932 1 IR

AT4G14520 chr4:8341936-8342045 2 15::27 9::14 25::57 26::50 0.76001084 1 Cassette

AT2G20920 chr2:8999195-8999228 2 947::15 908::1 928::986 844::999 0.760601579 1 A3SS

AT3G10915 chr3:3416514-3416618 5 195::0 279::19 18::216 35::267 0.761154637 1 IR

AT4G14970 chr4:8561111-8561167 28-29 0::25 0::15 12::17 9::13 0.761165041 1 Cassette

AT3G19980 chr3:6963108-6963188 4-5 249::6 180::12 15::181 16::178 0.761191468 1 AltStart

AT4G02890 chr4:1279389-1279417 2 85::104 32::55 4338::4140 4026::3725 0.76144814 1 A5SS

AT1G75560 chr1:28372563-28372748 2 29::641 59::690 466::412 524::462 0.761483791 1 Cassette

AT3G01550 chr3:217794-217809 5 18::499 10::425 406::500 357::439 0.761635022 1 A3SS

AT4G09970 chr4:6247200-6247210 6 101::15 172::15 115::123 186::205 0.761639395 1 A3SS

AT5G61830 chr5:24837651-24837700 2-3 48::8 63::10 17::72 17::91 0.761752125 1 Cassette

AT4G34460 chr4:16478754-16478994 2 553::8 528::23 25::482 29::499 0.761758312 1 IR

AT1G21920 chr1:7705526-7705642 1 12::766 16::712 384::449 395::422 0.761906012 1 IR

AT1G22180 chr1:7829671-7829751 2 3::38 5::36 29::36 25::50 0.761942251 1 Cassette

AT3G63445 chr3:23425754-23425861 2 52::76 60::123 97::87 117::94 0.762203899 1 A3SS

AT1G28680 chr1:10079444-10079498 3 1::301 21::388 48::285 61::343 0.762428523 1 A3SS

AT3G13030 chr3:4171779-4171909 2 17::0 31::0 15::60 13::94 0.763183106 1 AltStart

AT1G67350 chr1:25235813-25235820 2 43::1686 24::1673 142::1700 133::1768 0.763262496 1 A3SS

AT5G40500 chr5:16228022-16228053 1 90::49 102::62 83::139 139::151 0.76346576 1 A5SS

AT4G02600 chr4:1144052-1144137 1 40::103 44::78 63::85 58::84 0.763469257 1 IR

AT5G22040 chr5:7298381-7298386 2 0::23 1::12 62::82 38::69 0.763512184 1 A3SS

AT1G77490 chr1:29119587-29119592 10 707::5 897::16 505::803 677::1027 0.764107795 1 A5SS

AT3G16310 chr3:5527139-5527150 3 10::461 29::594 348::451 474::622 0.764204565 1 A3SS

AT5G53580 chr5:21766299-21766308 3 35::459 25::476 391::552 389::566 0.764279519 1 A3SS

AT3G06125 chr3:1849141-1849332 2 44::435 40::321 1005::505 952::528 0.764283903 1 AltEnd

AT2G28290 chr2:12070737-12070840 34 19::117 16::111 25::67 19::83 0.764354893 1 IR

AT1G06190 chr1:1893867-1893947 1-2 489::15 409::10 29::450 16::486 0.764478018 1 AltEnd

AT1G22310 chr1:7883125-7883197 1 10::70 2::86 40::39 47::48 0.764529977 1 IR

AT3G63260 chr3:23373296-23373393 5 648::93 778::165 87::584 130::803 0.764751804 1 IR

AT2G20050 chr2:8652112-8652121 6 12::107 18::86 123::136 98::121 0.764812368 1 A3SS

AT4G13100 chr4:7637658-7637663 3 20::126 10::127 92::112 82::102 0.764967068 1 A5SS

AT2G42540 chr2:17711308-17711378 3 0::1491 0::221 818::419 120::61 0.765055397 1 IR

AT2G42130 chr2:17566475-17566811 1 542::11 432::19 17::410 15::343 0.76531486 1 IR

AT1G11750 chr1:3969300-3969382 9 1189::5 1363::17 30::1282 41::1405 0.765331878 1 A5SS

AT3G57020 chr3:21099446-21099488 2 129::603 51::373 678::767 417::423 0.765463619 1 A3SS

AT1G07705 chr1:2385278-2385570 13 99::11 199::15 8::96 10::168 0.76559526 1 IR

AT4G25500 chr4:13025600-13025856 4 107::77 209::86 51::203 78::280 0.765631843 1 Cassette

AT4G03240 chr4:1424438-1424451 2 21::137 15::167 121::160 140::167 0.765760326 1 A3SS

AT3G08720 chr3:2650722-2650985 2 12::14 7::26 18::105 22::127 0.765891407 1 AltStart

AT5G14250 chr5:4599938-4600031 8 230::9 285::27 27::220 39::286 0.765954097 1 IR

AT2G30620 chr2:13045989-13046013 2 77::0 0::20 2092::1982 2357::1979 0.766682182 1 A5SS

AT3G11960 chr3:3793249-3793361 15 110::12 184::9 14::106 17::202 0.766787215 1 IR

AT2G28880 chr2:12401263-12401274 9 5::27 17::18 24::48 20::53 0.766854305 1 A3SS

AT3G22425 chr3:7952237-7952347 5 231::4 207::11 15::221 18::208 0.767105009 1 IR

AT4G15510 chr4:8861324-8861334 3 362::114 271::122 364::454 303::425 0.767336501 1 A3SS

AT5G51170 chr5:20794985-20795040 2 37::8 53::7 46::64 74::72 0.767389443 1 A5SS

AT2G04270 chr2:1475950-1475973 4 39::4 35::4 45::40 41::48 0.767419515 1 A3SS

AT3G11890 chr3:3757006-3757088 2 77::82 102::59 47::88 34::96 0.767450785 1 IR

AT1G76010 chr1:28529255-28529305 7 552::16 693::8 369::713 452::794 0.767611141 1 A5SS

AT5G65380 chr5:26123852-26123944 6 90::1573 88::1390 885::1047 799::921 0.767689631 1 IR

AT1G69530 chr1:26143008-26143025 5 395::21 773::23 7230::7768 9608::10462 0.767710781 1 A3SS

AT2G25970 chr2:11073061-11074353 5 48::386 74::320 67::528 89::536 0.767804651 1 A5SS

AT2G37080 chr2:15583109-15583115 4 41::63 34::43 55::132 36::160 0.767891641 1 A3SS

AT1G62800 chr1:23256695-23256701 4 18::94 7::119 114::150 119::157 0.768223925 1 A3SS

AT3G26085 chr3:9531042-9531130 3 169::193 131::190 120::264 113::278 0.768271708 1 IR

AT1G01060 chr1:35164-35212 9 23::89 2::11 1830::1982 1633::2070 0.768457214 1 A3SS

AT2G01070 chr2:77281-77363 4 20::3 18::18 7::172 18::202 0.768508451 1 IR

AT5G35180 chr5:13432693-13432782 25 201::23 377::64 30::142 64::277 0.768746223 1 IR

AT3G59330 chr3:21924787-21924822 7 2::54 0::139 48::58 109::99 0.768775725 1 A3SS

AT3G24740 chr3:9033475-9033554 3 89::62 149::58 37::151 48::205 0.769134667 1 IR

AT5G54840 chr5:22277016-22277397 4 81::6 82::6 5::96 5::113 0.76924183 1 IR

AT5G63440 chr5:25407738-25407867 5 83::6 145::14 8::118 18::195 0.769399223 1 IR

AT4G19020 chr4:10417873-10417886 9 46::4 49::21 22::75 31::68 0.769488201 1 A5SS

AT5G24320 chr5:8285536-8285548 7 99::7 139::7 17::102 25::164 0.769526438 1 A3SS

AT3G06700 chr3:2117802-2117905 1 7365::125 9383::192 512::5634 655::7141 0.769804794 1 IR

AT2G45380 chr2:18701098-18701145 6 18::181 14::184 97::163 112::171 0.769823995 1 Cassette

AT4G39680 chr4:18417126-18417184 2 21::600 13::636 305::353 310::409 0.769856078 1 Cassette

AT1G01170 chr1:74449-74455 2 39::1730 48::1424 1278::1975 1090::1744 0.769898206 1 A3SS

AT3G59660 chr3:22035986-22036023 11 4::100 16::124 101::105 139::157 0.769973259 1 A3SS

AT2G43465 chr2:18049303-18049353 1 5::6 12::16 13::16 18::26 0.769985763 1 A5SS

AT5G64470 chr5:25776814-25776821 2 17::24 34::35 24::47 48::87 0.770135403 1 A3SS

AT4G26140 chr4:13244785-13244816 11 20::25 23::30 25::53 36::63 0.770148048 1 A5SS

AT5G47900 chr5:19392659-19392796 3 0::50 0::58 25::24 36::20 0.770155631 1 Cassette

AT3G09410 chr3:2898350-2898437 11 156::17 181::18 14::150 22::187 0.770383744 1 IR

AT4G32360 chr4:15623505-15623533 8 36::7 32::22 15::50 31::55 0.770886677 1 A5SS

AT2G43680 chr2:18109048-18109069 3 7::377 9::397 60::448 59::407 0.770944759 1 A3SS

AT3G05210 chr3:1481426-1481431 8 6::137 30::202 160::132 218::182 0.77104428 1 A5SS

AT1G58602 chr1:21758509-21758658 3 124::40 132::27 21::151 18::175 0.771091359 1 Cassette

AT2G28380 chr2:12135844-12135934 1 7::461 20::401 240::234 218::219 0.771290299 1 IR

AT1G25054 chr1:8805188-8805210 6 10::37 17::44 3::3 3::3 0.771567739 1 A3SS

AT1G67190 chr1:25133039-25133044 2 17::18 7::16 23::55 17::62 0.771681982 1 A3SS

AT2G36720 chr2:15393287-15393405 1 18::100 14::71 54::68 41::65 0.771728921 1 IR

AT5G07150 chr5:2216106-2216136 2 15::0 17::2 6::17 8::25 0.77180464 1 A5SS

AT2G26930 chr2:11493003-11493008 6 202::11 261::38 194::235 252::297 0.771833901 1 A5SS

AT1G65270 chr1:24244646-24244795 9 42::87 70::202 69::60 101::115 0.771869499 1 A3SS

AT3G08690 chr3:2641225-2641365 1 648::22 508::21 34::151 41::115 0.772118332 1 A5SS

AT1G45688 chr1:17192392-17192517 2 594::2 590::25 38::760 49::840 0.772215701 1 IR

AT5G01470 chr5:192296-192302 7 153::33 110::33 83::162 64::165 0.772216344 1 A3SS

AT1G12750 chr1:4347537-4347790 2 41::24 47::32 38::105 53::144 0.772562298 1 A3SS

AT4G00335 chr4:146773-146791 2 10::133 8::120 14::136 11::120 0.772608507 1 A3SS

AT1G71920 chr1:27067706-27067716 4 39::0 41::0 9::23 7::8 0.772611868 1 A3SS

AT4G24460 chr4:12644151-12644165 5 73::13 102::6 57::81 80::120 0.772614998 1 A3SS

AT3G09470 chr3:2911106-2911167 11-12 284::33 408::29 25::182 32::238 0.772943144 1 Cassette

AT5G43210 chr5:17347802-17347833 1 24::17 22::20 23::39 26::56 0.773097253 1 A5SS

AT1G64150 chr1:23811764-23811775 3 128::29 89::44 147::174 101::145 0.773213941 1 A5SS

AT4G01690 chr4:732033-732127 9 0::2854 0::3391 1484::719 1719::934 0.773242733 1 IR

AT3G28070 chr3:10450623-10450707 6 142::17 191::14 19::89 22::116 0.773254563 1 IR

AT2G38185 chr2:16001721-16001739 7 45::0 14::0 13::38 8::39 0.773323055 1 A5SS

AT1G05790 chr1:1733762-1733778 3 3::55 9::53 11::44 15::60 0.773650797 1 A3SS

AT4G34670 chr4:16550124-16550134 7 6105::40 7736::0 3884::1708 4907::2144 0.773683131 1 A3SS

AT3G61480 chr3:22753018-22753028 9 42::8 36::17 44::64 45::66 0.773715161 1 A3SS

AT4G22350 chr4:11806749-11806837 1 3::39 13::34 23::23 20::26 0.773847299 1 IR

AT1G07840 chr1:2426103-2426192 7 139::18 192::10 19::148 14::194 0.774426224 1 IR

AT5G66850 chr5:26696142-26696171 11 224::15 261::16 43::156 54::194 0.774620858 1 A3SS

AT4G39330 chr4:18292590-18292706 5 0::7192 0::6306 3809::1173 3305::1098 0.775209595 1 IR

AT2G40280 chr2:16827840-16827869 1 6::57 16::45 17::77 24::55 0.775217797 1 A5SS

AT3G45100 chr3:16505741-16505754 4 70::10 159::20 60::96 134::208 0.775219293 1 A3SS

AT3G57970 chr3:21465938-21466026 4 15::9 49::8 7::19 11::46 0.775332635 1 IR

AT1G72970 chr1:27455209-27455291 5 29::1826 10::1465 1021::1172 839::979 0.775635143 1 IR

AT1G26560 chr1:9180066-9180083 5 17::185 9::121 59::202 45::164 0.775910359 1 A3SS

AT1G28110 chr1:9804126-9804167 10 5::10 30::12 198::243 265::337 0.776176099 1 A5SS

AT5G58790 chr5:23745448-23745468 3 11::2 16::11 35::38 36::68 0.776248964 1 A3SS

AT3G63440 chr3:23425944-23425993 5 76::57 122::77 109::100 162::124 0.776523558 1 A3SS

AT5G22440 chr5:7436232-7436242 3 6::1788 12::1909 1070::2114 1029::2371 0.776906377 1 A3SS

AT5G38290 chr5:15303498-15303812 2 12::187 9::190 80::123 92::111 0.776910111 1 Cassette

AT1G76570 chr1:28730049-28730087 4 244::26 315::23 294::376 383::441 0.777194445 1 A3SS

AT4G13350 chr4:7773349-7773358 2 69::14 56::14 33::81 24::70 0.777256087 1 A3SS

AT1G20620 chr1:7144025-7144105 1-2 7893::32 4860::46 40::8294 44::7993 0.777280233 1 AltStart

AT4G14713 chr4:8427362-8427428 7 36::13 89::10 12::64 15::115 0.777374283 1 IR

AT1G68080 chr1:25518512-25518603 6 11::82 5::115 39::54 63::78 0.777398854 1 IR

AT1G25420 chr1:8916493-8916571 2 212::25 190::46 24::228 32::243 0.777594282 1 IR

AT2G31725 chr2:13486624-13486629 3 13::131 7::132 97::87 90::108 0.777627488 1 A3SS

AT5G08130 chr5:2609354-2609391 3 9::109 18::68 119::149 99::147 0.777670699 1 A3SS

AT3G48780 chr3:18091837-18091873 11 44::378 66::370 92::419 115::480 0.777695215 1 A3SS

AT1G30590 chr1:10837560-10837588 11 17::89 14::147 106::139 143::208 0.777929497 1 A3SS

AT3G02180 chr3:404828-404978 1 752::124 760::82 88::1213 83::1232 0.777946122 1 IR

AT4G17190 chr4:9650565-9650645 2 215::1 167::5 13::207 15::187 0.777952296 1 IR

AT1G13920 chr1:4759560-4759623 7 9::8 13::16 48::42 39::71 0.777976635 1 A3SS

AT1G02100 chr1:390037-390053 2 245::25 299::36 234::270 275::341 0.778014281 1 A3SS

AT2G01090 chr2:82186-82220 6 139::29 183::34 202::247 244::309 0.778053482 1 A3SS

AT1G31360 chr1:11233087-11233136 3 7::7 20::4 9::20 24::29 0.778207263 1 A3SS

AT3G52720 chr3:19541044-19541152 2 19::718 3::477 644::982 440::723 0.778215993 1 Cassette

AT4G28660 chr4:14150432-14150452 2 27::3469 14::2828 685::2805 541::2705 0.778228648 1 A3SS

AT4G10100 chr4:6308816-6308849 1 333::78 371::70 307::321 329::347 0.778580547 1 A5SS

AT4G07410 chr4:4202772-4202859 8 0::365 19::450 189::217 243::323 0.778737512 1 IR

AT5G47710 chr5:19329903-19330097 2 113::59 151::70 59::194 49::254 0.778951893 1 AltStart

AT5G18620 chr5:6196327-6196336 24 50::101 53::137 37::158 39::212 0.779065044 1 A5SS

AT3G32980 chr3:13526660-13526739 4 7::7086 13::7178 3639::1890 3704::2031 0.779081916 1 IR

AT5G42220 chr5:16875560-16875569 13 200::16 189::12 156::229 151::234 0.779266474 1 A3SS

AT3G59650 chr3:22033674-22033756 2 368::11 483::12 28::349 35::470 0.779366005 1 IR

AT4G38120 chr4:17893849-17893922 9 14::9 14::15 20::37 18::31 0.77937502 1 A3SS

AT5G38212 chr5:15264745-15264798 1 38::10 38::12 45::58 45::87 0.77945285 1 A5SS

AT5G39740 chr5:15903426-15903486 2 2024::26 1729::11 25::1629 10::1412 0.779569441 1 Cassette

AT1G28580 chr1:10044823-10044862 5 5::802 11::1128 784::421 1086::554 0.779593765 1 A3SS

AT5G43100 chr5:17300272-17300288 4 23::98 37::87 48::122 52::114 0.779619602 1 A3SS

AT5G13980 chr5:4512309-4512315 19 20::261 15::280 31::265 31::271 0.779768446 1 A3SS

AT1G10390 chr1:3412608-3412708 1 64::16 45::19 14::57 12::37 0.779807974 1 IR

AT1G49340 chr1:18253848-18253869 2 15::53 20::25 57::55 30::41 0.779927979 1 A5SS

AT1G07700 chr1:2380924-2381014 4 658::2 822::22 47::383 69::475 0.779999741 1 IR

AT1G43770 chr1:16549897-16549981 4 21::11 25::17 9::35 12::41 0.78036283 1 IR

AT3G16240 chr3:5506414-5506511 3 15455::14 11400::7 16075::10719 11908::9303 0.780405736 1 A3SS

AT4G32420 chr4:15651375-15651496 6 14::6 13::14 6::17 13::24 0.780499675 1 IR

AT5G19221 chr5:6466283-6466328 6 2::893 9::652 83::764 73::560 0.781049896 1 A3SS

AT1G10890 chr1:3630255-3630279 5 86::34 125::39 100::97 138::147 0.782161464 1 A3SS

AT5G57290 chr5:23207152-23207198 2 120::18695 53::19587 9399::2741 9935::2961 0.782289781 1 IR

AT4G13495 chr4:7843376-7843383 2 449::6 611::7 684::1014 1152::1784 0.782572419 1 A3SS

AT1G12930 chr1:4401687-4401695 14 14::43 13::69 40::66 56::77 0.782990665 1 A3SS

AT1G06470 chr1:1970580-1970676 2 0::52 0::101 26::48 49::60 0.783026393 1 IR

AT4G17330 chr4:9697323-9697350 2 30::18 13::20 34::56 15::38 0.783053695 1 A5SS

AT2G39280 chr2:16403205-16403220 12 15::12 14::14 47::59 69::103 0.783149305 1 A3SS

AT3G18900 chr3:6519009-6519095 9 0::21 0::32 16::16 22::29 0.783476308 1 Cassette

AT2G25670 chr2:10930719-10930828 1 187::199 170::207 124::382 141::407 0.783615858 1 IR

AT2G32250 chr2:13693270-13693382 1 0::13 5::15 10::25 10::34 0.783731242 1 IR

AT3G01060 chr3:17108-17133 6 2::1144 5::1263 1066::641 1186::833 0.78400175 1 A3SS

AT4G01070 chr4:461881-462283 1 0::1078 0::1104 813::392 832::328 0.784190906 1 IR

AT1G51740 chr1:19190044-19190061 4 42::11 71::14 21::72 32::82 0.784224431 1 A5SS

AT1G55830 chr1:20869680-20869703 2 25::9 17::22 12::37 22::41 0.784261144 1 A3SS

AT4G18975 chr4:10393314-10393321 4 8::69 9::71 89::101 86::90 0.784402256 1 A3SS

AT3G14740 chr3:4952232-4952238 2 29::47 20::20 53::30 27::18 0.78445044 1 A3SS

AT3G05270 chr3:1502960-1503006 2 4::9 5::7 22::28 10::25 0.784854839 1 A3SS

AT3G44630 chr3:16198487-16198749 5 45::13 57::4 88::118 81::134 0.785086252 1 A3SS

AT1G68890 chr1:25903768-25903875 21 11::147 22::196 82::86 103::114 0.785187419 1 IR

AT3G12640 chr3:4014333-4014450 1 25::95 5::67 45::52 34::41 0.785294254 1 IR

AT2G14260 chr2:6042387-6042446 7 291::10 297::6 287::266 298::268 0.785405109 1 A5SS

AT1G01320 chr1:127935-127952 7 181::8 153::10 18::160 27::156 0.785415333 1 A3SS

AT1G69680 chr1:26205337-26205369 3 105::14 68::8 36::97 22::74 0.785617591 1 A5SS

AT2G21660 chr2:9265649-9265664 3 2::15 121::56 5802::1681 8816::2688 0.785797401 1 A3SS

AT3G05400 chr3:1550944-1551003 4 0::122 0::49 64::53 29::25 0.785806085 1 Cassette

AT1G67700 chr1:25375712-25375726 5 1689::1191 1902::1413 1493::3202 1789::3307 0.785894672 1 A5SS

AT4G34900 chr4:16626466-16626530 12 8::21 22::35 23::30 33::61 0.785902844 1 A3SS

AT2G42190 chr2:17582869-17583141 1 15::247 4::236 196::320 170::335 0.786174379 1 Cassette

AT5G56900 chr5:23021953-23022041 3 80::0 72::15 8::82 12::95 0.786333376 1 IR

AT4G01595 chr4:691897-691987 1 59::5 146::6 10::71 17::147 0.786542227 1 IR

AT2G29290 chr2:12586522-12586562 2 21::676 16::559 525::117 423::130 0.786560495 1 A5SS

AT1G18710 chr1:6452410-6452446 3 20::182 3::82 29::267 10::101 0.786782756 1 A3SS

AT1G17450 chr1:5993875-5993966 7 0::16 0::14 11::13 8::14 0.786869347 1 IR

AT4G02195 chr4:971225-971241 3 20::32 22::16 33::51 29::51 0.787035326 1 A3SS

AT5G49470 chr5:20064636-20064645 4 20::0 45::1 27::49 45::72 0.787082981 1 A3SS

AT1G09640 chr1:3121516-3121544 7 22::4842 0::5316 2603::2417 2839::2592 0.787188069 1 IR

AT3G13120 chr3:4220273-4220289 3 56::3071 31::4115 814::3589 1056::4206 0.787214264 1 A5SS

AT2G15280 chr2:6641242-6641289 5 206::19 149::10 186::196 149::167 0.787366041 1 A3SS

AT5G13450 chr5:4310638-4310686 6 15::2786 48::3312 2909::1602 3469::1913 0.787632831 1 A3SS

AT1G30475 chr1:10788682-10788913 5 51::6 50::27 7::30 18::38 0.787702658 1 IR

AT4G05000 chr4:2563109-2563346 1 46::0 34::9 9::26 10::19 0.787759591 1 A5SS

AT1G75300 chr1:28256642-28256658 4 26::7 11::13 22::42 40::70 0.787925039 1 A5SS

AT3G23830 chr3:8607687-8607699 2 1020::343 1232::500 539::1408 768::1996 0.788575111 1 A3SS

AT5G28050 chr5:10045412-10045484 2 1196::8 1109::10 23::542 23::525 0.788796959 1 Cassette

AT4G17560 chr4:9781249-9781281 4 1646::13 1674::29 219::1778 241::1714 0.788816969 1 A5SS

AT5G51410 chr5:20881595-20881699 7 28::240 45::214 132::107 111::121 0.788923963 1 IR

AT5G60870 chr5:24487093-24487100 2 18::5 20::11 14::48 22::79 0.789131751 1 A3SS

AT5G07150 chr5:2216224-2216295 3 15::0 17::4 9::38 12::48 0.789139103 1 Cassette

AT2G22010 chr2:9364572-9364581 10 103::24 112::12 62::126 54::154 0.789195567 1 A3SS

AT2G33480 chr2:14181879-14181885 3 51::6 94::10 40::61 88::126 0.789278576 1 A3SS

AT4G15540 chr4:8874673-8874701 6 289::19 444::48 319::429 474::693 0.789559204 1 A3SS

AT1G06590 chr1:2021901-2021906 8 8::29 16::23 32::43 27::40 0.789611956 1 A3SS

AT4G16990 chr4:9564596-9564692 8 159::220 308::417 123::303 226::615 0.789623988 1 IR

AT3G13677 chr3:4476271-4476277 2 137::7 176::9 163::154 200::163 0.789655753 1 A3SS

AT5G58200 chr5:23549649-23549738 1 73::6 78::14 12::52 13::64 0.789750378 1 IR

AT4G01700 chr4:730681-730688 3 36::877 52::708 583::1087 542::890 0.789904716 1 A5SS

AT4G38960 chr4:18162056-18162168 3 204::125 190::151 60::196 69::187 0.789977982 1 Cassette_multi

AT5G14320 chr5:4618006-4618234 2 5391::30 5571::53 115::3996 133::4071 0.790119441 1 IR

AT1G44740 chr1:16888579-16888885 6 38::0 27::0 17::41 41::66 0.790314089 1 Cassette_multi

AT5G49550 chr5:20106982-20107001 2 10::131 11::193 193::195 243::223 0.790593004 1 A3SS

AT1G19700 chr1:6812501-6812540 1 73::47 39::31 68::42 34::28 0.790752736 1 A5SS

AT5G02070 chr5:407601-407623 2 0::11 1::24 14::13 23::21 0.79088739 1 A3SS

AT4G15540 chr4:8873934-8874138 4 47::117 42::129 107::278 174::423 0.791043589 1 A3SS

AT1G49480 chr1:18316034-18316309 1 9::248 12::171 14::80 18::59 0.791178243 1 A5SS

AT2G40160 chr2:16777931-16778102 2 11::9 30::5 15::22 20::35 0.791227943 1 Cassette

AT3G16430 chr3:5581716-5581798 1 91::22 68::8 17::383 12::401 0.791349399 1 IR

AT5G41610 chr5:16639210-16639300 4 12::31 34::29 22::31 21::31 0.791615229 1 IR

AT4G39200 chr4:18258460-18258487 4 0::14612 181::16536 8152::5523 9125::6031 0.791784821 1 IR

AT3G23255 chr3:8312157-8312391 3 18::98 10::161 49::101 82::114 0.791805739 1 Cassette_multi

AT5G01580 chr5:223460-223488 2 6::5 8::15 8::19 11::24 0.791913038 1 A5SS

AT2G36250 chr2:15200131-15200469 1 1::226 0::137 16::74 28::52 0.792220648 1 A5SS

AT2G48110 chr2:19676092-19676216 9 67::0 52::0 51::22 54::47 0.79262106 1 A3SS

AT1G18460 chr1:6353287-6353292 2 7::119 19::97 41::200 41::206 0.792857399 1 A3SS

AT5G60410 chr5:24300626-24300667 17 17::30 14::31 306::343 481::520 0.793041511 1 A5SS

AT3G25840 chr3:9455027-9455084 5 10::92 10::116 10::82 9::108 0.793055198 1 A5SS

AT2G33820 chr2:14307056-14307061 5 15::57 26::64 72::68 83::94 0.793209205 1 A3SS

AT3G55480 chr3:20570720-20570793 2 29::10 32::18 10::44 14::43 0.793294515 1 IR

AT1G14570 chr1:4986664-4986679 9 334::16 470::9 288::418 416::510 0.793430997 1 A3SS

AT3G56730 chr3:21014187-21014235 2 19::2 11::1 7::20 3::23 0.793602879 1 AltStart

AT3G56260 chr3:20868081-20868276 1 12::77 6::106 39::49 63::49 0.793668913 1 IR

AT2G15050 chr2:6519225-6519246 1 277::272 287::373 767::1770 907::1809 0.793868673 1 A5SS

AT2G42610 chr2:17750095-17750105 2 143::35 308::81 103::75 224::171 0.793975503 1 A3SS

AT1G26880 chr1:9315733-9315820 3 4662::11 4926::33 345::2287 377::2591 0.79412132 1 IR

AT3G60500 chr3:22354225-22354242 2 9::51 8::50 23::75 20::66 0.794316007 1 A3SS

AT3G56460 chr3:20931269-20931349 6 4::9 16::10 22::318 38::376 0.794387095 1 AltEnd

AT4G25720 chr4:13100023-13100133 10 108::20 204::45 19::85 32::134 0.794447587 1 IR

AT5G24520 chr5:8371000-8371006 3 68::251 79::352 362::105 481::134 0.794651147 1 A3SS

AT3G10060 chr3:3103072-3103077 5 620::299 706::403 586::931 756::1060 0.794742771 1 A5SS

AT4G32030 chr4:15492715-15492819 3 381::46 367::18 53::247 37::238 0.794899312 1 IR

AT3G54500 chr3:20178454-20178491 3 99::1072 94::1438 998::1229 1330::1627 0.79496762 1 A3SS

AT1G05890 chr1:1780174-1780212 3 207::14 227::3 216::193 229::232 0.795058359 1 A3SS

AT5G18640 chr5:6213927-6213937 5 8::68 12::86 36::94 40::88 0.795126992 1 A3SS

AT3G03330 chr3:784046-784147 10 288::12 339::5 25::290 23::346 0.795245576 1 A3SS

AT5G13550 chr5:4357492-4357503 7 119::3 117::16 49::128 61::121 0.795344622 1 A3SS

AT5G59560 chr5:24001675-24001792 1 12::102 1::126 51::76 57::93 0.795405122 1 IR

AT3G46590 chr3:17154831-17154836 6 424::9 474::11 301::437 337::535 0.795580863 1 A3SS

AT4G06634 chr4:3766140-3766218 2 28::18 24::19 13::43 14::55 0.795797151 1 IR

AT2G28930 chr2:12424593-12424602 1 42::5 34::27 21::29 34::30 0.795932929 1 A5SS

AT3G60260 chr3:22274024-22274108 1 117::2 104::5 8::43 12::47 0.796040612 1 A5SS

AT4G24090 chr4:12513578-12513585 2 335::85 355::86 214::442 216::519 0.796108975 1 A3SS

AT1G19485 chr1:6747134-6747147 2 28::3 22::3 33::40 27::26 0.796262029 1 A3SS

AT2G24600 chr2:10452749-10452909 3 8::20 12::20 10::12 9::21 0.79629057 1 IR

AT3G14230 chr3:4738700-4738712 1 16::468 6::605 62::416 75::470 0.796323153 1 A5SS

AT5G13630 chr5:4388032-4388140 4 15328::0 14337::254 12313::12299 11238::10028 0.796388331 1 A5SS

AT5G01230 chr5:94531-94708 5 71::0 56::0 3::78 8::64 0.796492496 1 A3SS

AT2G17340 chr2:7542536-7542567 7 9::431 22::442 374::424 427::530 0.796522733 1 A3SS

AT5G26610 chr5:9375406-9375438 2 0::22 5::22 16::63 41::85 0.79684362 1 A3SS

AT3G11570 chr3:3645042-3645247 5 663::7 862::31 29::372 41::489 0.796910891 1 IR

AT1G75190 chr1:28219804-28219916 1 0::71 0::80 29::19 44::17 0.79696199 1 IR

AT1G76460 chr1:28688076-28688130 2 120::22 107::11 35::180 30::192 0.797038963 1 A3SS

AT3G08710 chr3:2645308-2645409 1 64::25 59::23 44::69 39::56 0.797210969 1 A5SS

AT1G54920 chr1:20474346-20474351 8 218::0 268::0 155::259 193::330 0.797219504 1 A3SS

AT1G60670 chr1:22346540-22346601 7 7::160 1::229 80::128 112::146 0.797360222 1 Cassette

AT4G02890 chr4:1278877-1279083 3 47::570 31::395 5097::3654 5715::4399 0.797584006 1 A3SS

AT1G34065 chr1:12399242-12399247 10 42::2 59::20 9::56 18::92 0.797641329 1 A3SS

AT1G65540 chr1:24366065-24366089 1 21::21 33::13 26::72 22::68 0.797671759 1 A3SS

AT4G25315 chr4:12952396-12952452 2 65::129 141::189 70::128 107::213 0.797828194 1 Cassette

AT3G52150 chr3:19343138-19343245 4 5::822 1::979 385::427 401::540 0.797983522 1 IR

AT5G12040 chr5:3887151-3887360 7 512::35 551::29 33::510 34::576 0.798133131 1 IR

AT5G44920 chr5:18139440-18140183 2 41::0 52::0 48::68 56::65 0.798274305 1 AltEnd

AT1G76010 chr1:28528742-28528803 8 20::4704 17::5823 2335::1861 2930::2398 0.798400385 1 IR

AT4G14145 chr4:8152786-8152791 3 177::25 155::38 40::179 70::237 0.798580984 1 A3SS

AT1G05900 chr1:1789025-1789162 9 66::13 149::16 9::88 17::157 0.798661587 1 IR

AT4G16950 chr4:9539044-9539174 8 122::33 198::57 23::116 42::173 0.79880627 1 IR

AT4G39100 chr4:18218733-18218753 4 121::427 168::538 383::638 498::816 0.798880766 1 A3SS

AT5G37370 chr5:14815843-14815938 2 44::181 19::164 97::160 91::136 0.798886354 1 IR

AT4G27490 chr4:13739923-13739946 2 13::65 10::65 47::171 51::185 0.798887405 1 A3SS

AT3G15354 chr3:5170172-5170190 4 163::155 225::148 203::314 282::443 0.798893554 1 A5SS

AT2G15280 chr2:6641030-6641099 4 19::318 10::240 162::178 125::168 0.799179489 1 Cassette

AT1G11330 chr1:3811979-3811985 3 10::0 29::0 17::40 34::53 0.799185218 1 A3SS

AT1G09980 chr1:3258865-3258888 7 51::10 89::6 52::77 73::81 0.799297369 1 A5SS

AT3G07565 chr3:2415354-2415381 5 181::20 258::17 46::158 59::203 0.799355765 1 A5SS

AT1G28395 chr1:9969772-9969836 4 106::297 159::365 263::107 317::144 0.799779045 1 Cassette

AT5G11650 chr5:3745811-3745845 4 19::86 5::80 34::139 13::108 0.800099903 1 A3SS

AT1G32120 chr1:11555604-11555817 2 41::0 44::0 16::57 7::57 0.800188314 1 A5SS

AT2G41670 chr2:17375123-17375128 5 28::6 19::13 18::40 17::45 0.800243804 1 A3SS

AT5G06120 chr5:1844737-1844803 2 9::76 6::67 56::41 52::44 0.800301531 1 Cassette

AT4G24770 chr4:12767590-12767635 1 54::43 32::9 9989::7061 8943::5722 0.800308051 1 A5SS

AT3G13062 chr3:4185358-4185382 2 458::10 388::3 70::412 61::356 0.800472051 1 A5SS

AT2G38180 chr2:15998220-15998278 5 12::69 11::101 83::68 97::91 0.800565768 1 A5SS

AT3G01350 chr3:136842-137057 4 13::145 5::76 85::75 33::46 0.800609487 1 IR

AT3G49160 chr3:18222491-18222841 3 20::85 23::215 41::68 104::109 0.800727438 1 IR

AT5G18130 chr5:5996359-5997049 1 170::19 147::5 10::111 8::98 0.800762403 1 IR

AT2G02020 chr2:480235-480242 4 6::7 17::10 44::39 65::69 0.801425659 1 A5SS

AT3G27130 chr3:10000031-10000042 5 134::20 143::7 101::34 114::49 0.801452519 1 A5SS

AT3G57140 chr3:21153395-21153477 1 1::15 2::23 9::10 15::12 0.801471781 1 IR

AT1G06690 chr1:2051132-2051154 5 20::615 13::572 285::262 279::224 0.802437729 1 Cassette

AT5G48655 chr5:19732878-19732916 1 279::8 195::17 289::194 238::167 0.802638 1 A5SS

AT3G17090 chr3:5827844-5827861 2 18::390 36::534 22::455 38::552 0.802755971 1 A3SS

AT5G55630 chr5:22531436-22531687 1 77::56 87::27 32::187 21::191 0.803085761 1 IR

AT2G18690 chr2:8097754-8097861 1 0::32 0::39 24::32 23::60 0.803143758 1 IR

AT5G50240 chr5:20451632-20451641 3 31::55 13::57 64::111 42::107 0.803301128 1 A3SS

AT4G18070 chr4:10032424-10032495 5 307::0 441::11 6::158 35::236 0.80330489 1 Cassette

AT2G48100 chr2:19670808-19670828 1-2 165::15 148::14 49::171 62::182 0.80346014 1 Cassette

AT5G60170 chr5:24229276-24229302 7 2::57 15::74 15::73 39::105 0.803473541 1 A3SS

AT2G32000 chr2:13620752-13620757 4 1::22 8::21 19::35 17::30 0.803665555 1 A3SS

AT1G20410 chr1:7080155-7080174 9 6::52 11::56 48::68 54::67 0.80374273 1 A3SS

AT4G38240 chr4:17931921-17931996 18 75::129 216::219 81::106 141::190 0.80377269 1 IR

AT1G76510 chr1:28712734-28712757 1 74::46 47::44 83::74 60::69 0.804093248 1 A5SS

AT2G21187 chr2:9076747-9076827 1 8::3634 8::2660 2081::1058 1523::756 0.804623198 1 IR

AT4G17520 chr4:9772170-9772176 2 11::2231 5::2221 1197::2140 1179::1925 0.805120073 1 A5SS

AT5G04830 chr5:1403673-1403771 1 669::22 671::29 51::362 59::381 0.805272948 1 IR

AT2G41430 chr2:17269402-17269490 1 2554::1 1488::8 238::989 127::524 0.805488108 1 A5SS

AT1G10670 chr1:3535578-3535645 1 606::8 565::10 38::95 44::89 0.805557673 1 A5SS

AT4G30580 chr4:14933400-14933405 5 259::63 249::63 141::481 151::604 0.805836983 1 A3SS

AT5G13590 chr5:4373617-4373622 7 15::56 13::93 73::53 97::111 0.805895934 1 A3SS

AT1G21350 chr1:7478682-7478694 2 368::15 359::7 133::321 119::291 0.806135022 1 A3SS

AT4G17310 chr4:9685454-9685710 3 73::10 66::19 15::47 18::38 0.8066943 1 A3SS

AT1G14700 chr1:5059621-5059629 2 41::447 23::328 342::436 235::364 0.806889531 1 A5SS

AT3G51950 chr3:19281037-19281043 2 214::41 132::34 184::242 128::183 0.806957067 1 A3SS

AT1G25540 chr1:8973807-8973837 2 0::110 3::49 47::47 23::24 0.806977128 1 Cassette

AT4G33080 chr4:15963990-15964025 12 17::931 16::1117 436::395 554::483 0.807025874 1 Cassette

AT2G21500 chr2:9207886-9207961 4 35::34 53::19 18::58 12::62 0.807151486 1 IR

AT5G47730 chr5:19334005-19334027 13 120::99 146::131 136::274 185::345 0.807187215 1 A3SS

AT1G08820 chr1:2824792-2824915 2 104::19 83::4 16::120 5::83 0.807208728 1 Cassette

AT2G35800 chr2:15046881-15046954 4 11::239 4::303 22::262 18::327 0.807240817 1 A3SS

AT5G53010 chr5:21490786-21490853 24 6::14 0::21 28::33 43::52 0.807272575 1 A3SS

AT3G54480 chr3:20171217-20171261 5 24::562 13::486 646::392 623::440 0.807417187 1 A3SS

AT1G73770 chr1:27742583-27742665 2 27::10 49::5 36::36 60::47 0.807462632 1 A3SS

AT5G50550 chr5:20577518-20577523 3 9::40 23::34 3::4 4::4 0.807648736 1 A3SS

AT1G57860 chr1:21430291-21430337 2 2214::19 2809::26 79::1396 119::1686 0.80769432 1 A3SS

AT1G09840 chr1:3200103-3200183 1 0::35 20::45 18::30 25::40 0.807725341 1 IR

AT1G13340 chr1:4569448-4570269 3 5::33 11::44 43::29 42::26 0.807777964 1 AltEnd

AT1G23380 chr1:8302027-8302033 2 66::15 61::3 48::57 33::51 0.807925182 1 A3SS

AT5G17230 chr5:5661908-5662013 3 5::3291 18::2762 1838::1740 1535::1474 0.808063748 1 IR

AT3G20870 chr3:7312505-7312618 1 16::280 10::220 155::119 142::92 0.808535652 1 IR

AT5G24000 chr5:8111782-8111835 3 32::51 43::47 44::139 61::111 0.808694673 1 A5SS

AT1G01448 chr1:165769-165983 5 9::8 20::13 44::30 78::57 0.808776522 1 A3SS

AT2G29310 chr2:12590228-12590233 2 138::425 94::244 201::825 118::477 0.808878527 1 A3SS

AT4G27270 chr4:13662895-13662931 2 65::19 66::40 40::95 71::138 0.809189096 1 A3SS

AT2G31902 chr2:13561602-13561621 2 7::12 17::10 9::19 22::36 0.809197014 1 A3SS

AT2G02710 chr2:759154-759160 6 13::80 33::125 69::119 95::153 0.809448681 1 A3SS

AT5G65380 chr5:26125299-26125380 3 26::1852 16::1454 1078::973 826::731 0.809683376 1 IR

AT1G09570 chr1:3098940-3099032 2 0::404 0::301 224::229 165::201 0.809756109 1 IR

AT1G06230 chr1:1910222-1910227 5 25::197 25::278 111::269 154::335 0.809779467 1 A5SS

AT2G32710 chr2:13874945-13874954 2 100::117 121::100 97::306 106::302 0.810014604 1 A3SS

AT3G52340 chr3:19407119-19407205 1 12::23 19::39 17::52 28::57 0.810095389 1 IR

AT5G22875 chr5:7650681-7650710 1 149::4 169::18 36::54 49::54 0.810223925 1 A5SS

AT4G13220 chr4:7674664-7674928 2 15::770 9::942 409::251 562::319 0.810547142 1 Cassette_multi

AT5G26980 chr5:9490215-9490220 2 10::59 16::77 34::65 41::67 0.810774785 1 A3SS

AT3G49260 chr3:18262560-18262716 1 120::5 91::10 9::120 7::84 0.810791921 1 IR

AT5G65440 chr5:26152251-26152375 3 17::19 13::30 8::32 20::36 0.810818524 1 IR

AT2G29910 chr2:12751808-12751895 2 65::23 92::34 21::87 28::98 0.811198986 1 IR

AT3G19460 chr3:6748498-6748601 3 75::51 106::94 31::122 52::139 0.811638426 1 IR

AT3G55370 chr3:20528568-20528662 3 115::33 90::21 27::94 21::91 0.811818599 1 IR

AT2G46280 chr2:19004106-19004200 6 1532::17 2266::10 142::1518 205::2273 0.811960213 1 IR

AT4G31020 chr4:15108127-15108258 7 84::12 117::4 7::96 9::114 0.812009121 1 IR

AT2G42230 chr2:17593748-17593833 8 143::35 219::32 30::133 36::191 0.812128767 1 IR

AT2G47410 chr2:19451509-19451539 9 7::8 8::18 10::22 15::32 0.812207561 1 A3SS

AT5G25760 chr5:8967718-8967952 1 145::30 165::62 25::102 41::128 0.812316452 1 IR

AT3G58110 chr3:21517752-21517843 1 0::125 0::132 71::78 71::94 0.812393472 1 IR

AT1G07745 chr1:2401344-2401356 6 32::29 37::14 31::54 32::52 0.81250912 1 A5SS

AT5G27860 chr5:9875445-9875539 4 80::4 96::18 361::420 594::650 0.812695991 1 A3SS

AT1G08220 chr1:2582860-2582865 3 30::15 70::15 45::52 68::80 0.813043685 1 A3SS

AT5G62090 chr5:24938622-24938920 2 25::13 18::17 7::85 8::66 0.813050692 1 IR

AT1G72770 chr1:27392393-27392517 4 311::182 323::144 109::234 104::230 0.813101093 1 IR

AT3G12600 chr3:4004684-4004845 1 12::773 6::965 318::221 407::239 0.813133148 1 IR

AT1G07940 chr1:2464913-2465076 2 3623::90 2597::53 65::2047 45::1507 0.813224762 1 Cassette

AT5G21222 chr5:7211235-7211315 9-10 134::13 139::4 19::167 10::186 0.813258948 1 AltEnd

AT4G25100 chr4:12886516-12886612 3 210::36 224::50 225::14369 219::13853 0.813532298 1 IR

AT1G08550 chr1:2708187-2708192 5 120::26 130::15 64::165 70::182 0.81354374 1 A3SS

AT1G55840 chr1:20874058-20874086 2 15::660 6::566 300::226 251::211 0.813556156 1 Cassette

AT1G07350 chr1:2258747-2258845 5 118::361 108::467 312::369 376::445 0.813923907 1 Cassette

AT1G72560 chr1:27324983-27324992 13 28::13 64::13 25::58 49::98 0.814041755 1 A5SS

AT5G07890 chr5:2519112-2519211 4 8::11 28::11 9::20 8::46 0.814198628 1 IR

AT2G43530 chr2:18070120-18070170 1 14::291 28::334 293::104 389::142 0.814552896 1 A5SS

AT5G57790 chr5:23412239-23412676 3 21::48 18::32 24::44 21::41 0.814582245 1 IR

AT1G05950 chr1:1804952-1804963 9 11::9 25::8 16::29 33::38 0.814811141 1 A5SS

AT1G68140 chr1:25540794-25540840 1-2 258::16 277::13 13::309 17::359 0.814843362 1 Cassette

AT2G30970 chr2:13178905-13178984 1 0::222 0::182 127::296 104::269 0.814892288 1 IR

AT2G19580 chr2:8474714-8474742 2 4::181 13::153 177::221 148::180 0.815029634 1 A3SS

AT5G48000 chr5:19447091-19447171 2 30::42 10::18 33::401 14::276 0.815201981 1 AltStart

AT3G18850 chr3:6501174-6501307 2 8::18 5::10 14::70 7::70 0.815520481 1 AltStart

AT2G43910 chr2:18186254-18186320 3 2657::16 2710::4 16::2147 12::2514 0.815579956 1 AltStart

AT3G01380 chr3:149065-149109 3 9::13 7::33 25::38 38::50 0.815744156 1 A3SS

AT4G25450 chr4:13011080-13011085 11 274::11 219::6 106::258 89::248 0.81580462 1 A3SS

AT2G27000 chr2:11524091-11524107 2 7::29 15::30 35::53 40::65 0.816004308 1 A3SS

AT4G08170 chr4:5166183-5166213 3 17::18 18::41 18::29 26::41 0.816088253 1 Cassette

AT1G51070 chr1:18928693-18928755 3 31::1253 26::1596 660::584 866::709 0.816116744 1 Cassette_multi

AT3G63180 chr3:23338073-23338097 10 39::35 24::20 47::88 32::84 0.816329948 1 A3SS

AT5G59290 chr5:23916090-23916130 11 3::1802 12::1818 839::753 898::802 0.81643014 1 Cassette

AT4G29670 chr4:14535882-14536097 3 610::50 870::45 38::435 37::571 0.816436191 1 IR

AT5G06230 chr5:1885161-1885250 5 594::14 672::39 59::209 77::250 0.816752995 1 IR

AT4G28030 chr4:13937893-13937926 5 18::876 13::1102 966::632 1215::803 0.817101372 1 A3SS

AT1G51590 chr1:19130779-19130792 5 13::75 22::64 23::81 28::80 0.817480355 1 A5SS

AT3G49720 chr3:18441688-18441702 2 479::21 469::9 114::539 97::485 0.817485528 1 A3SS

AT1G31580 chr1:11311523-11311531 2 20::11740 24::14391 123::9995 311::13864 0.817609629 1 A3SS

AT1G17200 chr1:5879397-5879429 2 1218::28 1126::11 1103::1188 1013::1079 0.817727354 1 A3SS

AT3G16460 chr3:5593743-5593771 3 52::3387 13::2913 1736::1496 1556::1241 0.817995799 1 IR

AT3G01390 chr3:151380-151460 1-2 1089::7 1008::20 20::1314 21::1358 0.818162232 1 AltStart

AT5G47650 chr5:19312379-19312438 1 11::77 9::44 33::220 21::173 0.818188568 1 AltStart

AT4G28680 chr4:14157474-14157480 9 27::4 36::0 36::50 32::41 0.818192234 1 A5SS

AT4G28910 chr4:14264703-14264715 4 20::446 26::514 520::475 620::637 0.818384419 1 A3SS

AT5G11630 chr5:3740877-3740942 2 89::149 124::246 193::357 308::449 0.818574808 1 A5SS

AT1G58807 chr1:21783289-21783378 4 38::157 125::271 3::3 3::3 0.818846127 1 IR

AT1G03160 chr1:764831-764866 8 11::136 10::205 144::126 200::173 0.818941377 1 A5SS

AT3G55630 chr3:20638283-20638295 11 308::33 364::21 236::278 268::350 0.81896427 1 A3SS

AT5G16290 chr5:5337478-5337493 13 15::287 13::503 60::157 106::264 0.819051776 1 A3SS

AT1G02120 chr1:399011-399068 16 5::162 12::272 34::177 49::286 0.819092956 1 A3SS

AT1G54360 chr1:20291357-20291378 5 16::127 21::155 99::123 121::147 0.819633376 1 A5SS

AT2G26600 chr2:11316613-11316793 1 10::30 7::25 33::32 28::22 0.819701682 1 A5SS

AT1G23380 chr1:8297811-8297885 4 11::159 30::152 80::104 90::111 0.81994634 1 IR

AT5G55000 chr5:22321405-22321546 11 206::10 243::3 12::131 11::172 0.820079771 1 IR

AT1G62920 chr1:23304365-23304444 1 26::0 53::0 11::37 8::42 0.820129187 1 A5SS

AT1G19680 chr1:6808607-6808632 6 23::58 32::48 63::73 62::83 0.820222884 1 A5SS

AT1G16020 chr1:5499475-5499513 5 56::10 75::23 8::90 31::94 0.820516713 1 Cassette

AT1G43620 chr1:16429558-16429643 2 15::10 3::13 7::20 10::20 0.820549251 1 IR

AT4G27120 chr4:13604234-13604332 1 31::22 29::21 20::62 16::58 0.820560175 1 IR

AT1G52360 chr1:19505365-19505447 25 40::255 31::374 260::261 392::348 0.82057463 1 A5SS

AT2G01350 chr2:166067-166072 6 201::10 204::20 33::246 45::329 0.820770819 1 A3SS

AT1G67040 chr1:25019703-25019709 5 45::30 34::27 30::78 27::80 0.820876049 1 A3SS

AT2G20080 chr2:8664064-8664144 2 0::53 4::48 28::30 32::20 0.82091691 1 Cassette

AT1G67210 chr1:25143434-25143440 10 17::0 35::1 10::25 24::31 0.821041701 1 A3SS

AT3G16470 chr3:5597972-5597994 2 15::310 4::296 65::16 52::14 0.821065281 1 A5SS

AT1G02960 chr1:667281-667373 12 27::94 50::84 55::74 47::91 0.821170693 1 IR

AT1G27920 chr1:9729293-9729366 9 11::14 6::20 20::24 36::44 0.821213463 1 Cassette

AT1G08640 chr1:2748766-2748776 10 7::924 8::1408 700::511 1058::777 0.821379035 1 A3SS

AT1G55260 chr1:20615810-20615864 2 845::21 729::7 951::1055 879::1029 0.821533316 1 A3SS

AT2G19580 chr2:8474828-8474834 1 181::5 154::14 101::127 87::105 0.821630958 1 A5SS

AT4G21580 chr4:11475849-11475949 1 30::515 6::370 284::227 221::144 0.821829904 1 IR

AT3G05380 chr3:1540660-1540747 4 15::6 27::0 3::23 4::25 0.82189359 1 IR

AT1G19860 chr1:6893818-6893898 1-2 82::5 49::10 20::91 21::113 0.82196545 1 AltEnd

AT1G68890 chr1:25901357-25901479 13 22::18 25::0 50::65 49::75 0.822101107 1 A3SS

AT3G26360 chr3:9656389-9656405 2 262::6 318::12 124::374 124::444 0.822253254 1 A3SS

AT3G01690 chr3:257771-257788 4 25::883 22::970 273::1122 307::1268 0.822285332 1 A3SS

AT2G26170 chr2:11141696-11141703 2 123::7 98::10 65::89 55::76 0.822297526 1 A3SS

AT3G58900 chr3:21774055-21774066 3 21::3 43::2 64::103 108::173 0.822325423 1 A3SS

AT5G01600 chr5:229287-229318 1 3481::35 4845::10 3824::2191 5204::2764 0.822379078 1 A5SS

AT2G28310 chr2:12085689-12085698 2 88::9 109::9 60::129 70::102 0.822386293 1 A3SS

AT4G15510 chr4:8861036-8861050 2 324::71 251::45 305::477 218::363 0.822467513 1 A3SS

AT3G63450 chr3:23427059-23427080 8 42::13 53::11 44::26 35::35 0.822540637 1 A3SS

AT1G34418 chr1:12582469-12582507 2 572::168 725::195 572::545 798::718 0.822903684 1 A3SS

AT3G14930 chr3:5021309-5021404 3 21::1343 5::1294 759::694 717::694 0.822971357 1 IR

AT2G21270 chr2:9112085-9112093 11 58::365 66::317 126::433 118::353 0.823393852 1 A3SS

AT5G02830 chr5:645993-645998 7 226::44 250::48 85::308 87::349 0.823573437 1 A3SS

AT4G21580 chr4:11476961-11476979 5 335::0 225::4 320::407 241::377 0.82357851 1 A3SS

AT3G03380 chr3:805015-805115 15 64::308 48::348 160::221 196::254 0.824049728 1 IR

AT4G37560 chr4:17647406-17647641 4 11::262 17::246 138::143 142::175 0.824062655 1 Cassette

AT4G29160 chr4:14380876-14380966 1 476::59 340::24 40::377 19::282 0.824195693 1 IR

AT2G39770 chr2:16589376-16589382 2 1641::8 1622::13 1049::1379 1071::1455 0.824624484 1 A3SS

AT4G25450 chr4:13012818-13012844 4 10::93 9::53 17::117 15::90 0.824701054 1 A5SS

AT1G78370 chr1:29484714-29484766 2 4::9366 101::10172 8804::3813 9604::4533 0.824885962 1 A3SS

AT5G04130 chr5:1127482-1127488 2 43::68 25::35 83::126 49::75 0.824925883 1 A3SS

AT2G01460 chr2:211065-211093 23 52::5 81::13 60::71 87::96 0.824985036 1 A3SS

AT3G57340 chr3:21219036-21219143 1 76::108 40::86 88::287 67::283 0.82526404 1 IR

AT1G78200 chr1:29420380-29420477 1 107::85 135::73 59::83 52::84 0.825547236 1 IR

AT1G07120 chr1:2185034-2185050 4 0::30 6::18 3::39 9::38 0.825620525 1 A5SS

AT4G35940 chr4:17023031-17023199 3 11::7 30::2 41::44 70::63 0.825672154 1 A3SS

AT2G38570 chr2:16137688-16137698 4 17::12 25::5 16::43 12::40 0.825875108 1 A3SS

AT2G35330 chr2:14869060-14869226 1 71::34 29::37 20::115 23::110 0.826091431 1 IR

AT5G64960 chr5:25956087-25956186 2 146::35 139::55 20::186 32::193 0.826192895 1 IR

AT1G09700 chr1:3138114-3138199 3 81::45 99::85 6::51 7::58 0.826200629 1 IR

AT5G61920 chr5:24864493-24864540 1 19::1 18::2 13::18 9::15 0.826405008 1 A5SS

AT2G17970 chr2:7820932-7821126 1 16::9 8::24 8::29 11::29 0.826413838 1 A5SS

AT3G18430 chr3:6326122-6326163 2 5::202 6::143 43::245 23::225 0.826783225 1 A3SS

AT1G08970 chr1:2882686-2883141 1 449::5 407::27 13::535 12::540 0.826789663 1 IR

AT3G21160 chr3:7416844-7416858 5 13::60 8::67 21::77 20::81 0.826828067 1 A5SS

AT2G29350 chr2:12601935-12602034 4 21::4 36::3 5::14 5::26 0.826835988 1 IR

AT1G79790 chr1:30017442-30017518 4 313::108 333::162 83::341 112::403 0.827142305 1 IR

AT1G32900 chr1:11923595-11923618 1 16::1273 6::667 150::236 79::145 0.827818573 1 A5SS

AT1G79630 chr1:29964622-29964750 3 46::18 38::24 35::81 23::77 0.828114093 1 Cassette

AT2G46090 chr2:18952182-18952193 8 8::194 19::224 84::189 98::220 0.828259763 1 A3SS

AT3G59350 chr3:21932685-21932971 2 12::81 8::70 35::106 34::119 0.828366989 1 AltStart

AT3G22420 chr3:7947559-7947656 4 135::23 127::30 22::135 27::144 0.82851076 1 IR

AT4G08035 chr4:4867187-4867719 1 207::13 405::30 20::87 22::154 0.828625672 1 IR

AT1G14685 chr1:5042855-5043077 2 15::26 3::49 9::212 24::246 0.82864419 1 IR

AT2G40690 chr2:16975463-16975474 6 59::18 51::13 61::113 50::112 0.829051038 1 A3SS

AT2G35390 chr2:14895708-14895812 6 536::4 707::25 22::340 36::464 0.829069134 1 IR

AT1G03330 chr1:818273-818280 3 7::618 13::659 716::398 754::441 0.829123394 1 A3SS

AT2G46640 chr2:19149187-19149193 3 85::32 44::27 57::142 39::112 0.829128028 1 A3SS

AT1G52905 chr1:19706111-19706260 1 12::243 5::342 194::44 213::38 0.829279648 1 A5SS

AT1G80640 chr1:30312533-30312551 2 7::182 11::286 176::193 259::276 0.829314463 1 A5SS

AT5G14850 chr5:4802464-4802469 8 4::59 12::80 39::63 58::90 0.829399724 1 A3SS

AT5G48790 chr5:19781034-19781054 5 546::12 523::28 133::558 153::596 0.829602427 1 A3SS

AT5G27300 chr5:9621075-9621101 2 38::13 35::22 57::73 52::86 0.829714063 1 A3SS

AT3G61690 chr3:22833086-22833240 8 17::207 40::270 104::127 135::188 0.829741505 1 IR

AT2G32060 chr2:13640258-13640314 1 41::744 41::840 44::171 51::206 0.829806681 1 A5SS

AT3G21250 chr3:7461603-7461693 2 9::126 6::122 67::77 79::68 0.829954227 1 IR

AT4G37680 chr4:17700986-17701044 2 9::819 15::617 132::260 107::198 0.83008509 1 Cassette

AT3G09660 chr3:2962322-2962333 12 11::0 28::0 12::22 33::32 0.83033714 1 A5SS

AT3G48780 chr3:18091610-18091776 10 44::655 66::691 324::275 329::326 0.830536368 1 Cassette

AT4G23430 chr4:12230527-12230557 4 263::76 220::76 272::364 233::303 0.830604093 1 A3SS

AT5G36790 chr5:14481091-14481225 11 40::1341 35::1624 7::9 8::11 0.830607702 1 IR

AT1G35580 chr1:13124815-13124827 2 13::443 2::381 345::529 304::489 0.830703907 1 A3SS

AT4G17040 chr4:9587374-9587451 5 20::3210 0::3382 1503::1721 1591::1679 0.830724917 1 Cassette

AT3G12570 chr3:3989266-3989357 2 28::298 11::351 159::292 192::351 0.83076165 1 IR

AT5G51630 chr5:20970810-20970955 3 0::22 0::32 11::12 17::14 0.830826674 1 IR

AT2G20820 chr2:8964978-8965022 3 4162::17 4965::27 4040::3573 4757::4294 0.830912173 1 A3SS

AT2G35840 chr2:15053647-15053719 1 89::34 44::31 21::55 19::26 0.830949256 1 A5SS

AT5G54080 chr5:21948443-21948529 9 31::4 78::12 6::84 15::104 0.831019509 1 IR

AT1G13330 chr1:4570469-4570582 7 40::9 32::10 6::50 7::54 0.83111195 1 IR

AT3G18100 chr3:6201959-6202025 3 0::13 0::12 9::16 9::8 0.831209203 1 IR

AT1G60600 chr1:22326582-22326590 3 10::169 11::175 112::205 110::211 0.831216762 1 A3SS

AT3G59950 chr3:22144852-22144943 7 200::166 253::141 75::137 89::168 0.831376191 1 IR

AT5G60570 chr5:24348368-24348380 2 42::168 30::185 75::373 75::465 0.831381541 1 A3SS

AT1G26210 chr1:9067301-9067381 1 52::69 64::109 34::77 50::99 0.831578011 1 IR

AT2G29630 chr2:12669987-12670093 7 58::0 75::0 68::27 101::53 0.831774673 1 A3SS

AT1G06515 chr1:1991970-1991984 2 41::207 40::243 230::258 287::336 0.831775109 1 A3SS

AT1G80245 chr1:30174975-30175061 4 18::112 38::182 63::76 96::148 0.831793059 1 IR

AT1G18620 chr1:6410059-6410216 1 50::0 30::0 13::47 17::32 0.831842696 1 A5SS

AT3G23480 chr3:8422552-8422646 14 47::12 44::16 12::44 13::37 0.831949691 1 IR

AT1G78680 chr1:29594907-29594912 5 4::543 12::479 107::530 102::463 0.832001848 1 A3SS

AT5G22620 chr5:7518903-7518914 5 144::8 172::18 73::219 95::247 0.832108458 1 A3SS

AT5G38210 chr5:15263599-15263679 1-2 1::13 7::7 24::30 20::48 0.832238799 1 AltStart

AT5G25040 chr5:8623620-8623706 5 13::142 10::230 91::77 136::120 0.832288209 1 IR

AT5G28840 chr5:10863615-10863874 2 1899::18 1395::0 1938::2395 1396::1730 0.832327123 1 A5SS

AT4G19160 chr4:10478321-10478347 4 533::38 561::57 199::563 205::552 0.832520859 1 A5SS

AT3G04630 chr3:1258798-1258820 2 340::10 316::4 8::383 4::398 0.832757033 1 AltStart

AT1G78265 chr1:29451595-29451643 1 4::8 1::12 89::124 89::159 0.832816693 1 A5SS

AT5G27840 chr5:9863413-9863506 4 318::10 334::2 17::193 29::214 0.832827236 1 Cassette

AT5G44572 chr5:17969011-17969106 2 22::365 5::283 195::123 156::92 0.832849557 1 Cassette

AT4G35760 chr4:16942611-16942705 7 12::467 7::522 243::184 274::202 0.832858817 1 IR

AT2G28290 chr2:12072334-12072376 35 103::54 159::48 113::182 184::261 0.833412868 1 A3SS

AT2G26260 chr2:11178557-11178565 2 66::9 57::22 64::67 63::81 0.833622141 1 A3SS

AT2G41020 chr2:17119930-17120276 9 76::8 105::22 7::82 13::119 0.833745214 1 IR

AT1G09300 chr1:3004435-3004515 2 43::12 52::5 12::52 6::47 0.833766248 1 IR

AT2G37060 chr2:15576312-15576336 2 40::4 29::14 47::85 52::94 0.833943541 1 A3SS

AT1G11360 chr1:3821780-3821795 2 17::337 13::350 329::139 360::184 0.834038275 1 A3SS

AT4G38545 chr4:18023266-18023335 4 188::159 138::162 92::112 90::91 0.834138616 1 IR

AT1G66940 chr1:24973346-24973352 5 32::182 81::389 202::145 419::346 0.834168704 1 A3SS

AT5G58240 chr5:23558890-23558977 3 294::38 396::66 50::336 90::521 0.834544755 1 A3SS

AT5G58210 chr5:23552261-23552271 1 11::9 6::13 40::43 43::40 0.834548987 1 A5SS

AT4G19510 chr4:10637927-10637966 9 8::137 8::161 24::110 23::171 0.834753446 1 A3SS

AT5G65740 chr5:26302904-26302973 3 21::23 14::40 17::43 27::53 0.834809521 1 IR

AT3G51290 chr3:19043215-19043295 4-5 0::25 0::22 24::44 20::44 0.834884968 1 AltStart

AT4G31780 chr4:15375183-15375196 2 11::472 22::456 14::453 27::400 0.835114863 1 A3SS

AT4G05320 chr4:2719697-2719802 3 2111::19 2307::84 24293::21318 29168::26806 0.835293552 1 A3SS

AT4G39680 chr4:18416175-18416267 3 9::443 28::475 231::381 223::445 0.835359451 1 IR

AT1G20920 chr1:7289309-7289386 3 46::111 55::177 65::81 109::120 0.835444152 1 IR

AT1G29170 chr1:10190646-10190747 7 73::15 90::35 14::90 24::122 0.83554443 1 IR

AT1G30300 chr1:10675065-10675154 9 20::59 36::99 33::44 48::72 0.835605611 1 IR

AT5G64340 chr5:25733044-25733124 1-2 110::9 91::6 17::268 6::348 0.835651886 1 AltStart

AT2G20990 chr2:9017052-9017125 10 1297::13 1023::12 136::1530 108::1472 0.835701815 1 IR

AT1G51200 chr1:18984554-18984724 1 5810::154 5776::145 236::2810 223::2718 0.835760319 1 IR

AT2G03640 chr2:1106420-1106431 2 40::114 36::99 167::209 142::181 0.835901101 1 A3SS

AT1G28410 chr1:9977970-9977985 4 128::11 127::22 34::138 52::168 0.835993222 1 A3SS

AT2G37760 chr2:15831894-15831971 1 32::138 29::66 56::282 32::179 0.836169436 1 IR

AT4G19110 chr4:10455011-10455020 16 641::355 820::363 602::718 709::865 0.83627321 1 A3SS

AT3G01490 chr3:191989-191996 3 3::317 13::302 245::329 259::405 0.836291419 1 A3SS

AT5G15390 chr5:4996013-4996026 2 187::43 268::59 100::244 136::249 0.836307716 1 A5SS

AT5G50650 chr5:20610860-20610865 3 8::25 12::37 3::3 3::3 0.836374255 1 A3SS

AT5G23090 chr5:7749342-7749383 2 0::95 11::119 14::100 28::126 0.836562462 1 A3SS

AT1G72850 chr1:27414780-27414925 2 24::36 35::25 19::29 13::40 0.836698841 1 IR

AT1G11400 chr1:3838708-3838715 3 299::15 354::26 216::314 257::343 0.83677294 1 A3SS

AT3G13300 chr3:4304100-4304206 1 39::125 24::79 66::123 53::80 0.836844817 1 IR

AT5G51690 chr5:20998514-20998648 4 20::136 19::177 65::100 91::136 0.836880363 1 IR

AT2G26810 chr2:11434254-11434433 7 57::16 48::30 12::80 24::94 0.836991098 1 IR

AT1G77180 chr1:29001664-29001840 2 305::29 254::25 33::527 37::524 0.83699118 1 A3SS

AT1G28290 chr1:9890492-9890513 1 239::345 236::254 1868::1333 1739::1248 0.83700123 1 A5SS

AT1G66330 chr1:24732266-24732360 2 116::35 64::25 38::627 43::616 0.837275764 1 AltStart

AT2G42280 chr2:17611795-17611909 3 63::19 110::10 8::78 12::121 0.837335642 1 IR

AT5G25270 chr5:8757711-8757791 15-16 0::188 0::348 88::231 176::297 0.837787821 1 AltEnd

AT1G20693 chr1:7177522-7177626 3 1522::15 1821::9 85::1570 109::1784 0.837811506 1 A5SS

AT3G06820 chr3:2152661-2152794 2 89::11 67::20 13::116 12::114 0.837846496 1 IR

AT2G31955 chr2:13585344-13585349 4 15::75 43::93 19::91 34::133 0.83788667 1 A5SS

AT5G61030 chr5:24560500-24560560 1 81::5 112::10 14::43 18::53 0.838374245 1 A5SS

AT4G35310 chr4:16802347-16802365 2 100::6 67::11 98::213 73::164 0.838402067 1 A3SS

AT3G01540 chr3:214883-215134 4-5 1037::57 1027::68 142::1196 180::1282 0.838466102 1 Cassette

AT2G38610 chr2:16149675-16149759 1 0::109 9::95 54::99 48::86 0.838693703 1 IR

AT5G20950 chr5:7110797-7110819 2 395::97 356::55 128::475 102::428 0.83871204 1 A3SS

AT4G38660 chr4:18067113-18067139 2 834::3 927::12 856::790 913::756 0.838735485 1 A5SS

AT1G78922 chr1:29676831-29677050 3 4::190 12::258 51::157 73::233 0.838816971 1 IR

AT3G54840 chr3:20320723-20320741 8 16::398 24::526 16::193 22::248 0.838897478 1 A3SS

AT3G54500 chr3:20178064-20178158 3 750::59 993::131 112::1293 182::1691 0.838922152 1 IR

AT5G62000 chr5:24910610-24910713 1 436::61 261::40 51::416 37::266 0.839057504 1 IR

AT3G09600 chr3:2948156-2948178 8 125::344 233::492 201::673 367::1021 0.839202008 1 A3SS

AT5G64360 chr5:25736550-25736689 3 19::28 45::59 12::30 33::61 0.839240889 1 IR

AT2G45135 chr2:18609217-18609306 1 9::23 5::42 14::21 23::36 0.839401045 1 IR

AT1G55960 chr1:20929031-20929107 3 38::4165 24::3618 2211::2197 1922::1940 0.839439853 1 IR

AT4G18070 chr4:10031118-10031189 2 194::27 194::17 20::195 11::243 0.83967612 1 AltStart

AT1G03055 chr1:710801-710955 4 37::33 44::28 16::58 19::63 0.839691729 1 IR

AT4G28790 chr4:14219369-14219406 3 18::10 16::10 17::26 27::39 0.839856357 1 A5SS

AT4G32000 chr4:15474555-15474618 8 13::32 11::54 34::65 58::73 0.839877407 1 A3SS

AT2G40205 chr2:16792420-16792445 2 2479::47 2689::83 481::2447 560::2566 0.839958003 1 A3SS

AT2G23390 chr2:9962696-9962703 4 3::216 13::265 187::273 216::265 0.840299121 1 A3SS

AT3G59060 chr3:21828302-21828308 6 270::1565 235::1698 939::631 1023::640 0.840348867 1 A3SS

AT3G22380 chr3:7913911-7913919 2 124::11 117::18 19::221 23::149 0.840399924 1 A5SS

AT3G24010 chr3:8676836-8676853 4 96::20 115::40 57::166 75::176 0.840518789 1 A3SS

AT5G40490 chr5:16225907-16225947 2 21::670 18::680 325::349 334::333 0.840596302 1 Cassette

AT2G31902 chr2:13560892-13560963 1 14::30 23::33 17::25 22::37 0.840614338 1 IR

AT5G55040 chr5:22327458-22327538 9 11::134 18::282 85::97 155::147 0.840872127 1 IR

AT2G22970 chr2:9778144-9778149 14 225::0 260::0 197::122 224::169 0.841043479 1 A3SS

AT4G15530 chr4:8870287-8870382 3 305::0 144::3 31::296 22::172 0.84115899 1 Cassette

AT1G29800 chr1:10435042-10435132 5 81::17 127::16 18::84 21::115 0.841159325 1 IR

AT3G55000 chr3:20383274-20383283 7 12::238 7::251 129::298 139::364 0.841212932 1 A3SS

AT1G15020 chr1:5173267-5173441 11 30::168 14::227 68::158 90::209 0.84130011 1 IR

AT5G44562 chr5:17948145-17948162 1 21::220 15::190 40::124 28::106 0.841496953 1 A5SS

AT4G16990 chr4:9560816-9560992 2 179::3 184::12 15::177 35::227 0.841715195 1 Cassette

AT5G64816 chr5:25913063-25913348 1 274::34 233::42 45::79 57::70 0.841732192 1 A5SS

AT2G15240 chr2:6615865-6615987 10 24::330 9::393 184::84 215::96 0.841737607 1 IR

AT3G60410 chr3:22332886-22333305 3 79::22 100::52 21::103 27::138 0.841812115 1 IR

AT5G27950 chr5:9987146-9987229 10 6::367 15::355 188::136 191::159 0.841850979 1 IR

AT1G49350 chr1:18266163-18266172 6 47::16 49::13 48::62 57::80 0.841912664 1 A3SS

AT4G10925 chr4:6704351-6704376 2 102::11 96::23 55::173 56::169 0.841963562 1 A3SS

AT1G31910 chr1:11458042-11458122 1-2 684::30 786::37 45::864 57::1025 0.842154423 1 AltEnd

AT2G17560 chr2:7642600-7642612 7 860::33 902::22 693::953 730::1027 0.842190535 1 A5SS

AT2G23140 chr2:9848940-9849104 1 12::25 6::31 20::23 12::29 0.842375393 1 A5SS

AT3G26910 chr3:9915329-9915345 11 28::43 58::42 54::26 56::35 0.842401716 1 A3SS

AT5G66720 chr5:26639970-26639979 3 14::452 32::437 345::628 330::634 0.842402467 1 A3SS

AT3G01340 chr3:128880-128892 1 16::287 30::240 87::100 90::83 0.842431486 1 A5SS

AT2G29190 chr2:12544089-12544197 10 3::630 12::791 297::175 390::258 0.842520459 1 IR

AT2G48110 chr2:19675212-19675268 6 40::0 17::0 5::41 12::36 0.842529616 1 Cassette

AT1G64960 chr1:24132613-24132693 3-4 0::18 0::42 27::26 41::34 0.842623938 1 AltStart

AT1G01060 chr1:37398-37569 1 1139::65 895::75 64::326 64::241 0.842637692 1 IR

AT3G19790 chr3:6875424-6875453 3 9::201 9::161 209::150 159::155 0.842762298 1 A3SS

AT5G21040 chr5:7146784-7146789 2 42::12 30::13 39::164 31::183 0.843063904 1 A3SS

AT3G56450 chr3:20931260-20931360 2 4::9 16::20 22::21 35::39 0.84318985 1 Cassette

AT1G14650 chr1:5030602-5030719 3 6::254 4::365 137::191 207::223 0.843208026 1 IR

AT3G22320 chr3:7892111-7892117 2 779::28 627::27 504::733 421::660 0.843365174 1 A3SS

AT1G28960 chr1:10109785-10109811 6 105::131 100::95 154::74 128::76 0.843477593 1 A3SS

AT5G43880 chr5:17643073-17643153 1-2 52::11 36::5 10::118 6::127 0.843775286 1 AltStart

AT4G38930 chr4:18151101-18151113 8 107::84 155::76 108::150 120::186 0.843903488 1 A5SS

AT5G58140 chr5:23529781-23529786 22 11::1441 22::2056 619::1397 888::1972 0.843915951 1 A5SS

AT3G52190 chr3:19355649-19355663 4 1::156 14::229 126::154 198::215 0.84393105 1 A3SS

AT1G66510 chr1:24816419-24816572 15 108::49 139::68 25::129 37::163 0.844130298 1 IR

AT3G20390 chr3:7111512-7111581 1 2911::19 3233::28 3320::2292 3621::2252 0.844192899 1 A5SS

AT1G53910 chr1:20135800-20135806 2 723::12 830::34 287::835 329::1025 0.844583676 1 A3SS

AT4G00050 chr4:19296-19324 5 394::24 338::7 447::666 388::659 0.844605835 1 A3SS

AT2G33740 chr2:14271322-14271442 5 380::68 432::56 66::287 59::332 0.84467894 1 IR

AT1G09195 chr1:2968653-2968758 8 0::29 0::39 28::19 26::32 0.844766475 1 Cassette

AT1G48460 chr1:17912002-17912113 3 17::180 6::153 194::180 167::190 0.845384356 1 A5SS

AT3G23610 chr3:8479496-8479607 6 2::29 3::28 9::19 16::22 0.845447504 1 A3SS

AT1G16610 chr1:5677355-5677376 7 81::371 74::358 370::563 373::585 0.845596427 1 A3SS

AT1G01740 chr1:272531-272555 9 1::19 15::21 20::25 27::44 0.845754126 1 A3SS

AT3G29375 chr3:11281495-11281584 5 107::16 165::25 20::110 30::155 0.845848734 1 IR

AT3G33530 chr3:14087259-14087275 3 80::0 43::0 8::68 20::73 0.845992576 1 Cassette

AT3G53130 chr3:19694228-19694233 4 18::241 18::210 244::246 203::202 0.846156178 1 A5SS

AT5G57150 chr5:23154156-23154274 5 99::0 203::0 10::78 3::129 0.846244946 1 Cassette

AT2G02570 chr2:698326-698340 9 29::0 79::0 113::176 261::317 0.84624859 1 A5SS

AT5G66360 chr5:26511206-26511291 2 11::100 11::130 56::58 66::75 0.846389033 1 IR

AT3G60370 chr3:22315920-22315925 4 126::37 170::44 51::204 60::289 0.846692079 1 A3SS

AT3G62970 chr3:23272548-23272561 12 19::132 12::190 22::137 16::180 0.846825853 1 A3SS

AT1G79830 chr1:30033584-30033590 2 48::80 49::55 73::159 53::106 0.846863949 1 A3SS

AT1G17890 chr1:6155442-6155575 1 106::14 102::37 17::204 22::245 0.846874856 1 IR

AT1G75300 chr1:28256716-28256770 5 26::7 11::13 38::25 32::27 0.846941497 1 A3SS

AT1G18360 chr1:6317138-6317247 7 12::307 8::324 179::121 170::119 0.847103958 1 IR

AT3G12570 chr3:3989357-3989386 3 28::28 11::18 230::298 251::362 0.847470125 1 A3SS

AT5G44750 chr5:18055027-18055044 7 29::14 61::28 23::61 41::113 0.847596832 1 A5SS

AT5G24170 chr5:8190281-8190286 4 34::79 57::122 131::160 182::191 0.847866663 1 A3SS

AT5G03690 chr5:964853-964955 1 2::62 6::58 20::27 14::23 0.847875213 1 A5SS

AT3G06850 chr3:2160482-2160591 1 0::58 0::52 38::130 24::101 0.847907248 1 IR

AT5G21060 chr5:7152160-7152166 3 89::75 73::69 143::173 131::160 0.848238119 1 A3SS

AT4G13885 chr4:8029404-8029517 5 34::0 39::0 20::20 16::7 0.848513308 1 A5SS

AT3G05930 chr3:1771410-1771533 3 11::62 9::99 41::85 82::125 0.84871339 1 Cassette

AT2G25670 chr2:10930635-10930719 2 20::191 18::179 358::459 359::510 0.848778174 1 A3SS

AT1G29120 chr1:10178146-10178231 11 17::507 40::692 293::130 435::196 0.848846697 1 IR

AT1G80970 chr1:30421514-30421705 3 8::4 11::20 7::16 8::23 0.848909399 1 IR

AT3G47675 chr3:17578381-17578461 before first 56::22 65::22 45::71 91::125 0.849048747 1 AltEnd

AT5G36290 chr5:14302852-14302953 1 382::214 390::263 151::375 167::427 0.849188109 1 IR

AT2G31900 chr2:13562638-13562793 31 8::5 10::10 3::18 8::22 0.84930235 1 Cassette

AT4G19510 chr4:10636621-10636702 5 1::146 19::145 76::78 79::99 0.849391228 1 IR

AT2G26310 chr2:11203402-11203476 9 0::32 0::77 21::25 43::40 0.849415318 1 Cassette

AT3G29130 chr3:11103400-11103589 2 19::0 57::0 17::37 20::91 0.849470893 1 Cassette_multi

AT3G49430 chr3:18334538-18334650 12 986::7 1233::15 55::1040 69::1325 0.849570819 1 IR

AT2G29960 chr2:12769367-12769397 6 2::972 2::1079 1021::541 1162::709 0.849583214 1 A3SS

AT1G02930 chr1:662253-662334 1 0::20 0::10 7::104 5::236 0.849728444 1 IR

AT1G31500 chr1:11274848-11274853 7 21::52 23::91 67::83 97::95 0.849839272 1 A3SS

AT1G10740 chr1:3569261-3569266 6 15::696 8::607 739::796 639::726 0.849914052 1 A3SS

AT1G13980 chr1:4789461-4789492 2 45::28 42::22 53::91 48::62 0.85036508 1 A3SS

AT2G31560 chr2:13437422-13437618 2 12::170 35::204 52::283 75::391 0.850453459 1 IR

AT4G40060 chr4:18572125-18572138 3 1342::28 1107::20 488::1409 411::1397 0.850481253 1 A3SS

AT2G24360 chr2:10366591-10366755 1 296::13 278::4 281::138 263::113 0.850496149 1 A5SS

AT1G73990 chr1:27826027-27826037 5 20::104 30::80 33::127 31::112 0.850586966 1 A3SS

AT1G33050 chr1:11968004-11968127 6 141::64 186::111 14::132 21::203 0.850612213 1 IR

AT4G35070 chr4:16694593-16694671 1 1::53 1::58 27::20 25::18 0.85065887 1 IR

AT1G15290 chr1:5259707-5259765 17 443::17 515::10 409::454 479::452 0.850789107 1 A5SS

AT3G59330 chr3:21927379-21927386 4 13::8 38::13 12::30 28::66 0.850823748 1 A3SS

AT4G35450 chr4:16839702-16839746 1 645::458 558::363 298::131 249::114 0.850956763 1 A5SS

AT1G09270 chr1:2997838-2997920 10 34::605 24::807 351::313 461::365 0.850964497 1 IR

AT3G13190 chr3:4240735-4240740 3 9::7 17::18 41::73 46::89 0.850981211 1 A3SS

AT1G53670 chr1:20037642-20037663 4 1134::0 1065::0 1023::1172 967::1260 0.851059113 1 A3SS

AT4G27610 chr4:13787859-13787937 2 46::45 48::28 40::96 38::101 0.851071822 1 Cassette

AT5G60430 chr5:24302239-24302332 2 10::18 11::21 27::34 58::54 0.851113664 1 A3SS

AT4G24100 chr4:12518376-12518432 15 16::136 5::125 65::69 62::90 0.851424776 1 Cassette

AT3G48870 chr3:18125958-18126024 2 15::476 5::276 481::486 288::321 0.851827711 1 A3SS

AT1G52500 chr1:19560692-19560743 2 22::91 17::111 36::311 57::439 0.851927227 1 Cassette

AT1G74910 chr1:28138591-28138719 1 11::227 19::169 78::312 66::248 0.852009324 1 IR

AT5G13760 chr5:4442759-4442853 3 86::15 79::14 76::77 69::104 0.852117335 1 A3SS

AT2G46020 chr2:18923120-18923200 before first 38::28 17::6 22::112 9::128 0.852280149 1 AltStart

AT5G06130 chr5:1855346-1855561 1 780::31 743::51 31::527 34::494 0.852289929 1 IR

AT5G28020 chr5:10028185-10028486 2 12::68 7::82 32::164 40::190 0.852401575 1 IR

AT2G42780 chr2:17802272-17802391 8 0::353 0::480 222::145 263::224 0.852601446 1 Cassette

AT3G02740 chr3:590808-590834 1 140::15 120::10 50::136 57::168 0.852632775 1 A5SS

AT5G01350 chr5:146918-146923 2 86::1335 68::1395 1532::2881 1563::3084 0.852685986 1 A3SS

AT1G67900 chr1:25466852-25467159 2 36::35 21::23 18::61 18::38 0.852759244 1 Cassette

AT2G25660 chr2:10924906-10924911 3 25::6 21::18 41::48 44::45 0.852847409 1 A5SS

AT4G16280 chr4:9211511-9213587 4 10::62 28::60 13::51 16::73 0.852858996 1 IR

AT1G60900 chr1:22425366-22425674 4-5 56::47 82::93 75::147 148::249 0.852925675 1 Cassette_multi

AT4G34265 chr4:16402735-16402780 2 1387::65 1501::73 212::1805 240::1970 0.852936657 1 A5SS

AT3G13030 chr3:4171449-4171558 3 29::15 40::20 9::60 13::89 0.852939861 1 IR

AT2G34670 chr2:14613205-14613305 4 17::7 35::6 5::28 6::41 0.852985281 1 IR

AT5G57300 chr5:23210654-23210660 3 45::173 32::155 113::204 99::157 0.853009671 1 A3SS

AT3G29390 chr3:11293316-11294033 11 24::80 54::162 73::202 71::264 0.853012918 1 A3SS

AT5G22220 chr5:7363978-7363987 14 48::1 54::0 45::24 52::38 0.853116554 1 A3SS

AT4G39140 chr4:18231907-18231914 1 4::85 9::68 15::57 18::42 0.853146868 1 A5SS

AT2G30620 chr2:13045989-13046014 2 77::4061 0::4628 2099::1507 2365::1642 0.853229966 1 IR

AT5G05930 chr5:1781485-1781490 6 6::104 15::140 131::156 166::181 0.853794975 1 A3SS

AT3G01130 chr3:45475-45495 1 2175::10 2886::5 1936::1315 2576::1612 0.853948625 1 A5SS

AT2G23820 chr2:10142517-10142602 6 176::9 270::2 20::90 26::112 0.854109882 1 IR

AT5G47900 chr5:19392309-19392394 1 6::19 0::14 12::23 7::20 0.854468125 1 IR

AT5G25460 chr5:8864449-8864527 2 18::7577 17::5030 4022::3945 2700::2615 0.854499727 1 IR

AT3G63445 chr3:23423457-23423621 3 5::7 36::19 3::25 11::45 0.854568262 1 IR

AT1G55620 chr1:20790445-20790459 2 46::9 65::4 21::72 26::81 0.85473272 1 A3SS

AT3G13800 chr3:4540113-4540130 5 54::14 72::15 32::75 34::97 0.854890449 1 A5SS

AT5G20450 chr5:6912004-6912135 4 0::49 0::55 35::37 39::35 0.85494741 1 Cassette

AT5G08760 chr5:2855043-2855048 1 237::11 408::18 89::149 131::241 0.855024871 1 A5SS

AT1G11870 chr1:4004556-4004563 3 3::145 13::112 34::137 35::115 0.855110393 1 A3SS

AT1G28330 chr1:9934288-9934336 4 788::0 873::0 157::420 101::377 0.855264304 1 A3SS

AT4G31398 chr4:15239013-15239113 1 26::19 22::7 15::36 6::28 0.855358824 1 IR

AT4G05590 chr4:2908003-2908017 4 2::155 16::218 48::169 82::227 0.855406446 1 A3SS

AT2G14910 chr2:6408579-6408676 1 1590::3 1521::15 1748::1452 1640::1352 0.855440913 1 A5SS

AT1G53250 chr1:19857843-19857948 2 12::6 5::15 6::19 8::22 0.855848896 1 IR

AT2G41070 chr2:17131096-17131240 1 24::42 43::42 25::66 27::79 0.855921057 1 IR

AT2G09795 chr2:3671960-3672125 2 8::97 0::88 131::120 123::140 0.856009246 1 AltEnd

AT1G70480 chr1:26562178-26562297 1 167::21 155::28 17::133 21::121 0.856080698 1 IR

AT2G02770 chr2:776100-776201 13-14 8::12 36::12 14::25 46::47 0.856105526 1 A5SS

AT1G50570 chr1:18727828-18728077 2 95::65 34::42 44::212 31::212 0.856128712 1 AltStart

AT5G23120 chr5:7779660-7779668 5 10::2442 7::2737 1350::2460 1555::2641 0.856200521 1 A5SS

AT4G29810 chr4:14593415-14593494 7 399::4 540::24 44::169 66::233 0.857043212 1 IR

AT4G32970 chr4:15912329-15912413 6-7 0::11 0::54 10::11 28::31 0.857142134 1 Cassette

AT1G53650 chr1:20030944-20030962 1 47::12 45::22 52::35 45::38 0.85716922 1 A5SS

AT3G15395 chr3:5200851-5200880 2 201::12 218::12 23::353 30::420 0.857246394 1 Cassette

AT1G31190 chr1:11146497-11146510 8 11::399 9::500 133::402 174::516 0.857292646 1 A3SS

AT2G04845 chr2:1702824-1702829 4 70::22 80::20 75::100 80::123 0.857327759 1 A3SS

AT3G12250 chr3:3906077-3906150 2 34::49 23::14 28::32 12::25 0.857540858 1 Cassette

AT1G03290 chr1:807510-807631 1 31::19 18::10 22::26 13::19 0.857618602 1 A5SS

AT5G27350 chr5:9654037-9654073 18 632::3 842::12 615::137 810::187 0.857689856 1 A3SS

AT5G47435 chr5:19243811-19243892 8 20::12 33::23 8::35 14::56 0.857729405 1 IR

AT5G64170 chr5:25672666-25672693 8 17::576 35::865 319::370 488::495 0.857771661 1 IR

AT1G78870 chr1:29651949-29651981 7 586::12 764::5 595::708 806::993 0.857780048 1 A3SS

AT5G09240 chr5:2872350-2872361 5 73::37 84::48 40::107 50::138 0.857805684 1 A3SS

AT2G02570 chr2:699656-699812 3 63::14 69::9 36::127 54::159 0.857910389 1 A3SS

AT4G28880 chr4:14252858-14252871 9 401::5 355::10 277::373 260::385 0.857962428 1 A3SS

AT5G59480 chr5:23981174-23981187 2 177::11 142::4 41::195 29::157 0.858061093 1 A3SS

AT2G30600 chr2:13039259-13039283 7 22::47 31::97 32::69 53::112 0.858147734 1 Cassette

AT4G18593 chr4:10238140-10238205 2 108::21 109::9 189::239 177::255 0.858155665 1 A3SS

AT2G38130 chr2:15980177-15980234 3 80::104 80::108 118::250 133::296 0.858252806 1 A3SS

AT1G04945 chr1:1399423-1399465 2 534::34 596::15 27::509 22::499 0.858304344 1 Cassette

AT5G42850 chr5:17181977-17182050 2 729::24 857::53 46::877 68::965 0.858304618 1 IR

AT4G11820 chr4:7111839-7112030 2 0::251 0::215 181::320 139::332 0.858398936 1 AltStart

AT3G54910 chr3:20344636-20344641 2 19::8 29::4 15::48 37::61 0.858631427 1 A3SS

AT4G38930 chr4:18149691-18149771 1-2 72::4 60::12 7::128 24::160 0.85871955 1 AltStart

AT3G26100 chr3:9538281-9538377 3 44::173 64::200 90::211 97::254 0.858953354 1 IR

AT5G55290 chr5:22424114-22424206 1 365::137 430::113 89::432 74::480 0.858953731 1 IR

AT2G26430 chr2:11245511-11245528 2 8::352 12::329 317::233 305::243 0.859142787 1 A3SS

AT2G31900 chr2:13568236-13568256 7 10::0 20::0 14::12 25::13 0.859163936 1 A5SS

AT3G46600 chr3:17158209-17158298 1 22::448 14::171 231::440 90::164 0.859168691 1 IR

AT4G27710 chr4:13829871-13829918 4 2::168 19::216 179::196 223::211 0.859221623 1 A5SS

AT5G01260 chr5:106453-106476 3 21::113 17::122 145::181 154::150 0.859250453 1 A5SS

AT4G32440 chr4:15658272-15658397 3 33::121 41::158 79::52 74::74 0.859266969 1 IR

AT1G75330 chr1:28267607-28267615 2 17::764 30::692 355::851 355::794 0.859347011 1 A5SS

AT2G23550 chr2:10028547-10028635 1 0::41 0::32 20::17 15::22 0.859347195 1 IR

AT2G16900 chr2:7325029-7325159 3 102::8 73::10 17::114 25::128 0.859364726 1 A3SS

AT5G16800 chr5:5524701-5524735 5 149::30 123::23 65::116 92::132 0.859430151 1 A3SS

AT5G24210 chr5:8218102-8218182 2-3 134::39 112::45 35::226 34::278 0.859482023 1 AltEnd

AT1G54920 chr1:20473952-20473982 6 196::0 206::0 19::167 39::176 0.859639158 1 A5SS

AT3G48090 chr3:17757375-17757455 1 37::0 32::0 28::26 37::23 0.859962916 1 A5SS

AT5G55290 chr5:22424099-22424114 2 25::365 52::430 470::636 552::715 0.860180073 1 A3SS

AT3G04930 chr3:1364187-1364620 1 0::91 0::106 225::276 227::225 0.860212834 1 IR

AT1G80770 chr1:30355424-30355461 2 121::20 91::22 157::194 125::174 0.860725345 1 A3SS

AT2G28470 chr2:12173151-12173259 2 20::155 5::89 81::130 50::76 0.860759685 1 IR

AT1G80325 chr1:30197594-30197672 1 7::21 35::24 13::22 16::42 0.861043766 1 IR

AT4G21620 chr4:11491624-11491724 1 0::3599 0::3077 1978::897 1665::797 0.861103729 1 IR

AT2G11000 chr2:4345756-4345761 6 39::4 43::17 44::47 66::68 0.861313638 1 A5SS

AT1G33970 chr1:12350970-12351207 1 4::7 12::13 5::47 6::58 0.861364734 1 IR

AT5G45670 chr5:18528929-18528934 2 519::11 232::9 392::555 197::306 0.861616842 1 A3SS

AT4G13100 chr4:7637726-7637901 2 28::38 7::43 17::66 14::54 0.861671343 1 IR

AT4G31060 chr4:15116732-15116979 3 12::15 24::64 10::11 25::33 0.861717716 1 IR

AT3G61480 chr3:22750632-22750752 3 36::0 17::0 30::36 23::19 0.862040942 1 A3SS

AT3G10260 chr3:3172512-3172776 1 232::23 258::56 19::262 30::294 0.862053113 1 IR

AT3G20060 chr3:7003826-7003845 2 1018::0 919::0 16::713 30::629 0.862062713 1 AltStart

AT1G30510 chr1:10807353-10807435 5 9::1209 6::1228 653::375 659::437 0.862116227 1 IR

AT2G33490 chr2:14184408-14184425 3 9::22 11::42 17::34 21::49 0.862123016 1 A3SS

AT3G63510 chr3:23450988-23450993 3 43::15 44::10 56::77 62::107 0.862189675 1 A3SS

AT1G64770 chr1:24058038-24058049 2 1218::22 1069::23 1213::1275 1097::1259 0.862361164 1 A3SS

AT1G27370 chr1:9507212-9507328 2 16::2 14::19 26::60 53::113 0.862364011 1 AltStart

AT4G33500 chr4:16114000-16114010 5 8::139 16::141 120::138 123::138 0.862481044 1 A3SS

AT2G46450 chr2:19067096-19067113 6 113::82 126::78 110::109 216::140 0.862706743 1 A3SS

AT1G52630 chr1:19607907-19607941 3 11::11 13::17 22::34 21::39 0.862714214 1 A3SS

AT5G61150 chr5:24604075-24604081 10 115::21 176::17 54::207 83::262 0.862770145 1 A5SS

AT2G34660 chr2:14602710-14603206 1 66::0 41::7 3::62 4::32 0.862885739 1 IR

AT4G35300 chr4:16799425-16799456 2 34::32 18::23 54::54 36::32 0.862995168 1 A5SS

AT1G22880 chr1:8095964-8096046 1 12::253 2::395 130::106 207::153 0.863005185 1 IR

AT1G78680 chr1:29594725-29594730 4 402::11 321::16 174::489 142::429 0.863014705 1 A3SS

AT3G20440 chr3:7128162-7128251 6 0::41 0::51 26::23 31::29 0.8630526 1 Cassette

AT3G29010 chr3:11006932-11007123 2 19::20 4::16 18::34 14::26 0.863134685 1 Cassette

AT5G20620 chr5:6973732-6973960 2 7::15 15::3 441::363 536::405 0.863215864 1 A5SS

AT1G28580 chr1:10045846-10046109 1 529::63 557::105 34::477 46::512 0.86337595 1 IR

AT1G79520 chr1:29913736-29913913 2 362::35 133::14 46::438 30::188 0.863385722 1 A3SS

AT2G02860 chr2:831083-831108 4 3::54 11::45 59::70 52::65 0.863425814 1 A3SS

AT1G03740 chr1:936572-936655 6 61::0 87::15 5::50 16::76 0.86342733 1 IR

AT4G19490 chr4:10619426-10619435 8 19::94 20::70 45::108 37::128 0.863655359 1 A3SS

AT3G26510 chr3:9711338-9711400 3 10::91 8::138 49::33 75::52 0.863674382 1 IR

AT1G52370 chr1:19506522-19507018 1 9::11 20::12 4::52 11::55 0.863736683 1 IR

AT4G00440 chr4:198112-198212 7 16::136 30::243 77::121 126::141 0.863814304 1 IR

AT4G01610 chr4:695701-695705 5 449::8 301::0 7::614 3::422 0.864089618 1 Cassette

AT2G22730 chr2:9663871-9663961 14 6::5 16::14 4::12 9::14 0.864133273 1 IR

AT2G26540 chr2:11288719-11288766 5 192::3 260::13 182::170 274::247 0.86415554 1 A5SS

AT1G26230 chr1:9075105-9075307 2 0::38 0::31 40::36 45::50 0.864481448 1 AltStart

AT1G04310 chr1:1157546-1157651 1 17::14 20::14 19::38 36::47 0.864511554 1 A5SS

AT4G17098 chr4:9609100-9609180 1-2 22::2 25::14 4::164 21::203 0.864699718 1 AltStart

AT1G80270 chr1:30181227-30181232 2 22::83 7::78 25::96 24::95 0.86472438 1 A3SS

AT1G69840 chr1:26295590-26295703 1 15::69 12::29 7::25 4::18 0.864764442 1 A5SS

AT3G55010 chr3:20386690-20386816 1 219::93 208::78 64::248 55::251 0.864802658 1 IR

AT5G17250 chr5:5671434-5671441 11 17::16 25::13 10::34 9::28 0.864807411 1 A5SS

AT3G12520 chr3:3969617-3969630 11 8::18 8::28 27::37 40::58 0.864826006 1 A3SS

AT1G12800 chr1:4363819-4363824 4 11::660 12::438 399::608 270::446 0.864892143 1 A3SS

AT1G55260 chr1:20616046-20616055 3 967::4 950::10 953::207 937::212 0.864906852 1 A3SS

AT1G29951 chr1:10493238-10493323 4 21::830 26::978 489::415 565::520 0.865103633 1 IR

AT3G26670 chr3:9800615-9800661 2 14::25 4::30 43::84 57::74 0.865280561 1 A3SS

AT3G51370 chr3:19069439-19069653 1 17::528 19::509 327::230 288::211 0.865371608 1 IR

AT4G36050 chr4:17054156-17054167 5 9::5 13::11 9::34 20::33 0.865434601 1 A3SS

AT2G45950 chr2:18904624-18904717 9 103::40 128::77 35::99 53::142 0.865445345 1 IR

AT1G51160 chr1:18950007-18950014 2 194::218 176::155 248::680 264::676 0.86546648 1 A3SS

AT2G01450 chr2:201605-201684 4 313::42 261::63 427::342 423::334 0.865494437 1 A5SS

AT5G45500 chr5:18434986-18434995 2 71::18 43::27 29::159 30::177 0.865604961 1 A3SS

AT4G27700 chr4:13827393-13827400 2 1597::365 1269::324 664::1765 566::1453 0.86561358 1 A3SS

AT1G49590 chr1:18355610-18355708 4 203::0 153::0 11::163 32::168 0.86581401 1 AltEnd

AT1G07910 chr1:2446086-2446358 2 2::14 1::10 14::36 10::42 0.865983966 1 AltStart

AT5G58320 chr5:23579429-23579524 4 0::62 0::63 35::26 31::32 0.865993338 1 IR

AT3G52050 chr3:19305034-19305088 1 25::6 20::17 33::15 36::17 0.866169733 1 A5SS

AT2G30620 chr2:13046014-13046041 3 77::0 0::20 2325::1205 2632::1410 0.866216608 1 A3SS

AT2G16405 chr2:7108308-7108333 3 39::4 42::18 15::55 24::64 0.866559042 1 A3SS

AT5G16480 chr5:5382207-5382259 2 84::13 111::10 44::132 45::165 0.866617689 1 A3SS

AT2G46570 chr2:19127215-19127285 2 6::5 9::21 10::25 26::35 0.866634475 1 A5SS

AT1G49950 chr1:18496251-18496256 3 223::9 205::7 157::277 141::237 0.867103251 1 A5SS

AT3G61740 chr3:22852048-22852160 17 15::5 36::7 3::22 6::45 0.867177801 1 IR

AT1G52510 chr1:19564982-19565000 1 503::20 497::18 586::587 531::514 0.867338291 1 A5SS

AT2G16430 chr2:7121296-7121301 5 12::378 5::553 314::527 450::662 0.867363256 1 A5SS

AT3G24760 chr3:9041269-9041280 1 40::27 36::13 44::58 31::61 0.867488502 1 A5SS

AT3G04130 chr3:1085923-1086007 2 0::22 4::39 14::25 26::31 0.867522318 1 IR

AT3G16450 chr3:5588486-5588570 1 1406::78 978::73 154::3072 109::2426 0.867559992 1 IR

AT1G68530 chr1:25713329-25713689 1 3100::157 2385::101 79::2403 55::1916 0.867653765 1 IR

AT2G22990 chr2:9788915-9788925 11 2202::5 2125::17 1681::2343 1600::2348 0.867670162 1 A3SS

AT2G29640 chr2:12672076-12672221 3 10::0 36::0 4::29 8::42 0.867833046 1 Cassette

AT2G42500 chr2:17700253-17700524 2 5::1456 3::1445 713::608 700::525 0.867899172 1 Cassette_multi

AT3G56040 chr3:20795122-20795148 7 3::482 16::550 455::374 523::467 0.868126794 1 A3SS

AT3G55760 chr3:20700513-20700628 1 33::17 11::5 12::62 5::53 0.868136901 1 IR

AT1G28290 chr1:9890275-9890296 2 312::238 287::186 2855::3028 3161::3644 0.868278402 1 A3SS

AT4G38225 chr4:17928196-17928285 2 479::45 514::45 36::718 38::829 0.868518148 1 IR

AT4G01710 chr4:736090-736116 3 100::41 102::54 98::156 103::165 0.868533237 1 A3SS

AT2G27490 chr2:11749961-11749975 1 51::27 63::12 50::28 53::24 0.868580241 1 A5SS

AT1G21750 chr1:7648411-7648635 9 0::1609 0::2211 838::476 1257::653 0.868631057 1 IR

AT3G06880 chr3:2170355-2170533 16 0::65 0::128 53::32 88::29 0.868722531 1 AltEnd

AT3G27700 chr3:10257516-10257680 5 0::75 0::97 51::58 63::80 0.868857465 1 IR

AT1G05200 chr1:1505317-1505462 2 36::0 28::6 4::64 3::113 0.869080869 1 AltStart

AT4G21560 chr4:11469779-11470115 2 101::18 130::36 15::150 23::169 0.869097241 1 IR

AT5G61010 chr5:24554051-24554156 1 4::19 6::17 10::62 10::94 0.869179131 1 IR

AT3G21175 chr3:7423328-7423334 3 114::51 99::57 115::130 112::126 0.869378274 1 A5SS

AT1G11360 chr1:3822147-3822164 1 49::294 46::304 105::305 107::326 0.869449438 1 A5SS

AT4G03280 chr4:1440346-1440438 1 15496::77 12787::72 503::9607 423::7983 0.869503179 1 IR

AT4G30190 chr4:14772544-14772644 8 1092::0 930::8 60::1315 56::1173 0.869531669 1 IR

AT2G30880 chr2:13143298-13143379 9 117::15 105::24 18::128 20::126 0.869610075 1 IR

AT3G26612 chr3:9782973-9783059 3 11::51 15::99 55::101 97::108 0.869683728 1 A5SS

AT5G26110 chr5:9119290-9119370 2 22::207 30::258 123::159 137::166 0.870043033 1 Cassette

AT5G01960 chr5:372467-372487 8 8::277 7::389 63::287 79::385 0.870094312 1 A3SS

AT1G78010 chr1:29335372-29335379 7 28::73 56::79 36::102 60::172 0.870268725 1 A3SS

AT1G70590 chr1:26619046-26619134 2 17::143 16::121 24::173 26::157 0.870275219 1 A3SS

AT5G58600 chr5:23684280-23684376 4 58::5 87::1 6::37 7::59 0.87028883 1 IR

AT4G27900 chr4:13890726-13890819 1 9::47 10::70 25::69 33::104 0.87035146 1 IR

AT5G19070 chr5:6376358-6376373 4 4::41 11::69 18::66 34::98 0.87037054 1 A3SS

AT3G46980 chr3:17307809-17307886 6 100::4 186::2 4::101 6::142 0.870560735 1 IR

AT5G67110 chr5:26785762-26785827 3 0::149 5::103 73::75 67::85 0.870588811 1 Cassette

AT4G32900 chr4:15879538-15879546 5 76::263 102::244 170::318 173::328 0.870978679 1 A5SS

AT1G03687 chr1:919995-920087 7 241::3 313::24 9::182 18::227 0.870999179 1 IR

AT1G78865 chr1:29648196-29648430 2 63::19 18::11 13::16 11::6 0.871032533 1 A3SS

AT3G52950 chr3:19634794-19634837 2 59::0 44::0 72::100 55::123 0.871094849 1 A3SS

AT5G44780 chr5:18069186-18069298 4 37::54 14::33 31::57 14::49 0.871249374 1 IR

AT3G47630 chr3:17564959-17565055 2 28::5 63::3 6::35 8::69 0.871289205 1 IR

AT2G27340 chr2:11697648-11697653 4 14::0 51::0 20::26 52::54 0.87134611 1 A3SS

AT4G28760 chr4:14208170-14208243 2 51::70 26::60 49::90 37::62 0.871599744 1 Cassette

AT4G00840 chr4:355916-355936 5 88::31 79::39 53::108 58::111 0.87171916 1 A3SS

AT1G60530 chr1:22300999-22301012 2 23::29 22::65 45::64 71::102 0.871746638 1 A5SS

AT2G46100 chr2:18954055-18954209 2 473::4 525::16 22::350 25::363 0.871834888 1 IR

AT2G40160 chr2:16778481-16778645 4 14::13 23::5 14::23 24::26 0.871871741 1 Cassette

AT2G43010 chr2:17888716-17888722 7 772::91 790::64 874::309 928::345 0.871929949 1 A3SS

AT4G32440 chr4:15659357-15659449 5 23::99 30::157 57::44 101::70 0.872088126 1 IR

AT5G06340 chr5:1937268-1937295 3 910::29 1159::32 123::993 150::1124 0.872216737 1 A5SS

AT1G10060 chr1:3286358-3286399 8 5::65 4::93 71::65 87::86 0.872316844 1 A5SS

AT5G36880 chr5:14540139-14540237 1 6::80 0::91 5::40 4::44 0.872329552 1 A5SS

AT2G17770 chr2:7723537-7723645 1 10::6 9::5 4::24 5::17 0.872446698 1 IR

AT2G03140 chr2:944634-944645 6 6::22 24::23 12::43 23::45 0.872456176 1 A3SS

AT1G35340 chr1:12978080-12978127 2 221::4 228::10 210::192 234::226 0.872503757 1 A3SS

AT1G52370 chr1:19506548-19506571 2 9::13 20::8 16::109 13::139 0.87258832 1 AltStart

AT2G40316 chr2:16836777-16836794 2 229::14 198::23 57::219 60::199 0.87258956 1 A5SS

AT3G46460 chr3:17096484-17096515 4 6::375 22::440 61::365 81::423 0.872667016 1 A5SS

AT3G52340 chr3:19407900-19407953 4 28::196 17::178 372::285 348::267 0.872711026 1 A5SS

AT3G04580 chr3:1238190-1238300 1 2::26 2::20 17::48 11::53 0.872819364 1 IR

AT3G63220 chr3:23358587-23359058 1 97::3 63::10 5::113 6::111 0.872907457 1 IR

AT2G02020 chr2:480391-480484 5 0::93 0::150 49::46 77::72 0.873106572 1 IR

AT2G36880 chr2:15481189-15481256 1 6::2501 0::1778 175::2066 138::1459 0.873252819 1 A5SS

AT4G31990 chr4:15470911-15471083 12 1::1159 18::1630 614::457 827::597 0.873302588 1 IR

AT5G24740 chr5:8486432-8486528 12 0::21 0::13 8::17 6::14 0.873484366 1 Cassette

AT4G32850 chr4:15854318-15854327 14 44::65 63::64 59::82 118::118 0.87354052 1 A3SS

AT3G06040 chr3:1825089-1825166 2 20::456 19::500 560::534 645::721 0.873540948 1 A3SS

AT5G06210 chr5:1879359-1879369 4 24::540 39::713 162::334 206::435 0.873548273 1 A3SS

AT5G26850 chr5:9445936-9445944 2 78::13 34::8 92::138 58::106 0.873651598 1 A3SS

AT5G13410 chr5:4301412-4301424 3 306::14 267::2 93::296 77::281 0.873759598 1 A3SS

AT5G07370 chr5:2330957-2331043 3 220::0 230::0 51::321 70::376 0.873876831 1 Cassette

AT2G43410 chr2:18026259-18026359 6 10::52 3::82 28::94 48::109 0.874196474 1 IR

AT2G31900 chr2:13567318-13567410 11 9::8 2::10 10::14 14::12 0.874230432 1 A3SS

AT5G43810 chr5:17611005-17611361 1 27::7 14::3 6::27 3::15 0.874313458 1 A5SS

AT1G21190 chr1:7420069-7420112 3 624::8 781::15 65::358 86::418 0.87434297 1 A3SS

AT2G47890 chr2:19608955-19609039 2 105::0 113::10 10::101 18::115 0.874450643 1 IR

AT1G67360 chr1:25237913-25237996 1 150::27 131::7 24::202 14::164 0.874663664 1 IR

AT5G02940 chr5:687280-687285 10 63::23 31::26 69::115 55::82 0.874691815 1 A3SS

AT2G26280 chr2:11188034-11188129 7 9::485 6::701 249::296 356::421 0.874814169 1 IR

AT1G78670 chr1:29592085-29592090 4 376::13 306::20 165::429 141::400 0.874824209 1 A3SS

AT2G41670 chr2:17378296-17378301 9 620::23 599::39 688::606 643::546 0.874888229 1 A3SS

AT2G29650 chr2:12674248-12674267 7 729::10 915::22 148::843 215::1071 0.874937179 1 A3SS

AT1G48490 chr1:17922225-17922310 18 85::10 92::2 12::61 8::94 0.875131726 1 IR

AT3G08590 chr3:2611239-2611330 1 935::64 687::43 116::692 87::525 0.875472732 1 IR

AT1G55000 chr1:20516178-20516205 2 16::29 14::23 372::485 449::528 0.875615029 1 A5SS

AT1G07960 chr1:2467212-2467638 1 178::28 168::45 9::236 25::272 0.875683022 1 IR

AT4G19003 chr4:10408689-10408918 1 0::112 9::151 12::44 12::43 0.875925591 1 A5SS

AT2G33620 chr2:14234462-14234739 2 44::64 29::77 38::227 37::225 0.876102335 1 IR

AT3G45050 chr3:16477128-16477165 4 68::0 193::0 215::516 217::668 0.876278005 1 A5SS

AT1G21370 chr1:7483997-7484178 1 0::121 0::108 68::61 46::68 0.876317907 1 IR

AT5G03970 chr5:1072960-1072985 2 6::37 3::26 53::80 38::93 0.876384187 1 A3SS

AT4G23660 chr4:12331378-12331638 1 20::23 3::23 16::47 15::45 0.876439727 1 IR

AT5G67030 chr5:26754935-26754942 9 1993::12 1897::7 1524::2130 1409::1777 0.876477081 1 A5SS

AT5G19221 chr5:6464695-6464725 2 1038::3 1192::9 932::733 1095::932 0.876616152 1 A3SS

AT4G34840 chr4:16607440-16607470 7 58::8 85::16 28::85 31::89 0.87670166 1 A5SS

AT3G58640 chr3:21692685-21692693 3 11::146 6::127 102::181 107::145 0.876711251 1 A3SS

AT1G08980 chr1:2885840-2885853 7 250::27 288::25 236::367 254::381 0.876837383 1 A3SS

AT3G28270 chr3:10538171-10538714 1 302::25 103::20 6::610 5::303 0.876984898 1 IR

AT1G49540 chr1:18336569-18336575 4 5::34 5::26 21::54 20::36 0.877183994 1 A3SS

AT2G14890 chr2:6400188-6400690 1 4258::52 4443::77 94::2792 103::2549 0.877391655 1 IR

AT1G60545 chr1:22303894-22303979 1 3::30 15::101 20::30 55::47 0.877559797 1 IR

AT1G68410 chr1:25652517-25652743 1 9::22 7::36 15::19 21::18 0.877746617 1 IR

AT3G49900 chr3:18501509-18501518 3 1::34 0::58 18::48 31::47 0.877748121 1 A5SS

AT2G27710 chr2:11816840-11816926 1 7383::163 7838::210 413::3368 475::3792 0.877866974 1 IR

AT5G03905 chr5:1053573-1053586 3 183::11 161::18 179::158 170::198 0.877877306 1 A3SS

AT2G47940 chr2:19620266-19620273 10 926::17 920::31 500::779 487::762 0.87796571 1 A3SS

AT1G79000 chr1:29716775-29716938 18 0::208 0::319 119::83 188::123 0.878025721 1 IR

AT1G76990 chr1:28932929-28933242 2 325::5 362::7 19::685 48::966 0.87809247 1 AltStart

AT3G18535 chr3:6376695-6376709 2 129::61 116::37 74::189 49::142 0.878242826 1 A3SS

AT4G10970 chr4:6720676-6720714 6 169::0 215::0 15::197 24::207 0.878265844 1 Cassette

AT1G63855 chr1:23699919-23700015 5 10::54 10::71 31::54 38::60 0.878315528 1 IR

AT1G30300 chr1:10675154-10675161 9 20::25 36::23 64::35 100::61 0.878729996 1 A3SS

AT5G18830 chr5:6277761-6277786 4 105::4 93::14 27::132 33::115 0.878772627 1 A5SS

AT5G26760 chr5:9405754-9405965 3 43::11 45::34 7::88 16::88 0.879079637 1 IR

AT4G38552 chr4:18027708-18027809 1 130::58 267::68 34::159 48::269 0.879255268 1 IR

AT4G16540 chr4:9316577-9316618 3 32::0 44::0 8::30 6::46 0.879315196 1 A3SS

AT3G29390 chr3:11291362-11291368 6 121::8 98::12 78::116 67::143 0.879468106 1 A3SS

AT1G15220 chr1:5241121-5241246 1 117::4 96::17 9::95 15::95 0.879488514 1 IR

AT2G39950 chr2:16678967-16679015 3-4 67::149 43::92 87::164 60::145 0.879658319 1 Cassette_multi

AT4G32010 chr4:15482415-15482427 8 56::9 58::16 22::84 28::93 0.879670383 1 A5SS

AT2G34050 chr2:14383147-14383161 4 122::7 131::21 51::109 72::149 0.87971305 1 A3SS

AT4G25300 chr4:12945918-12945998 2 83::18 97::7 12::96 14::107 0.879757408 1 IR

AT2G46910 chr2:19272407-19272419 2 14::146 4::139 50::410 44::362 0.879886447 1 A3SS

AT5G64460 chr5:25775478-25775483 1 139::71 134::73 136::68 138::67 0.879908248 1 A5SS

AT3G46490 chr3:17116866-17116900 4-5 22::10 43::15 8::109 12::161 0.880032781 1 Cassette

AT1G03860 chr1:980894-980987 3 700::19 800::40 34::726 49::740 0.880136068 1 IR

AT5G55730 chr5:22558266-22558317 2 0::86 0::82 51::652 45::667 0.880305557 1 IR

AT1G67310 chr1:25202827-25202907 1-2 0::42 0::25 44::68 24::84 0.880849303 1 AltStart

AT1G07840 chr1:2424153-2424274 1 128::2 145::5 6::60 7::56 0.880965781 1 A5SS

AT3G47810 chr3:17638886-17639043 2 10::1366 6::1339 750::763 689::780 0.881075152 1 Cassette

AT3G56160 chr3:20839889-20839906 6 229::22 206::11 200::224 206::212 0.881181227 1 A5SS

AT2G16940 chr2:7347068-7347075 2 22::252 15::269 289::322 289::300 0.881216533 1 A3SS

AT5G10940 chr5:3452076-3452085 9 19::82 7::71 20::90 13::101 0.88126925 1 A5SS

AT2G20270 chr2:8737863-8737980 5 16::304 8::446 133::266 206::347 0.881272508 1 IR

AT1G72416 chr1:27259831-27259915 2 0::11 0::28 9::6 18::11 0.881442245 1 Cassette

AT1G80650 chr1:30315441-30315482 4 71::35 97::23 115::101 143::119 0.881468982 1 A3SS

AT5G04750 chr5:1372371-1372381 1 28::1483 16::1510 1512::1226 1539::1265 0.881530511 1 A5SS

AT5G01910 chr5:358084-358164 2 54::29 70::36 19::53 26::57 0.881668773 1 IR

AT3G27260 chr3:10068344-10068436 2 63::0 57::2 72::97 66::111 0.881738669 1 A3SS

AT5G06440 chr5:1966364-1966378 4 65::5 50::15 21::73 41::95 0.881800051 1 A3SS

AT4G38950 chr4:18159529-18159889 1 10::71 6::95 61::82 74::92 0.881807906 1 IR

AT5G47430 chr5:19240431-19240609 2 1::87 0::78 56::260 52::232 0.881809562 1 Cassette

AT5G37850 chr5:15067113-15067196 5 0::359 0::465 168::216 213::241 0.881918492 1 Cassette

AT1G11280 chr1:3790647-3790725 1 23::53 17::37 37::60 20::57 0.881967017 1 IR

AT4G23630 chr4:12317980-12318080 2 12::2252 21::2119 1212::825 1098::784 0.882100606 1 IR

AT3G61490 chr3:22758113-22758154 1 55::4 28::2 17::23 8::14 0.882223434 1 A5SS

AT2G41290 chr2:17211578-17211583 2 15::300 19::201 288::306 183::193 0.882338872 1 A3SS

AT1G80000 chr1:30096669-30096715 10 0::265 15::329 25::213 44::291 0.882454289 1 A3SS

AT5G46410 chr5:18826287-18826296 3 6::83 8::88 38::111 39::93 0.882471121 1 A3SS

AT4G23770 chr4:12384078-12384311 1 24::10 42::31 14::47 19::73 0.882541673 1 IR

AT4G33000 chr4:15924756-15924838 2 0::218 0::229 118::171 116::233 0.882630774 1 AltStart

AT2G23600 chr2:10043407-10043490 1 28::1605 22::2468 863::815 1306::1195 0.882792365 1 IR

AT5G61410 chr5:24683852-24683858 10 794::5551 832::6809 3267::3148 4011::3772 0.883040487 1 A3SS

AT1G76990 chr1:28935187-28935272 8 7::1469 9::2341 813::669 1251::1066 0.88331537 1 IR

AT1G48380 chr1:17878415-17878509 3 81::5 118::0 9::118 12::144 0.883316867 1 IR

AT5G37740 chr5:14992337-14992366 2 356::0 425::0 16::277 7::309 0.8833832 1 Cassette

AT4G28260 chr4:14004914-14005004 1 0::658 1::647 353::500 363::560 0.883467636 1 IR

AT1G07728 chr1:2396434-2396529 1 54::100 74::77 61::138 47::157 0.883620784 1 IR

AT1G18660 chr1:6422883-6422908 8 121::7 130::16 133::178 158::194 0.883654376 1 A3SS

AT1G07780 chr1:2411929-2412194 2 89::35 129::47 12::152 22::163 0.883685877 1 IR

AT3G07180 chr3:2283132-2283213 9 0::407 0::325 199::150 153::165 0.883714567 1 Cassette

AT5G24600 chr5:8421760-8421869 2 0::15 0::30 11::20 20::22 0.883898927 1 IR

AT5G03560 chr5:901323-902235 2 276::55 274::66 46::193 64::216 0.883924515 1 AltEnd

AT5G04740 chr5:1370441-1370514 3 274::9 313::20 41::350 53::480 0.883975586 1 A3SS

AT3G60590 chr3:22398732-22398737 2 15::31 9::36 19::83 16::72 0.884025344 1 A3SS

AT5G26622 chr5:9356305-9356977 1 17::178 14::329 100::102 167::138 0.884053252 1 IR

AT3G50210 chr3:18616275-18616370 1 2::57 4::46 34::34 25::43 0.884094536 1 IR

AT1G04390 chr1:1181938-1182013 5-6 0::24 0::53 28::24 36::34 0.884146192 1 Cassette

AT1G15790 chr1:5439837-5439852 2 42::4 23::9 28::64 21::42 0.884189292 1 A3SS

AT3G10300 chr3:3187734-3187826 3 94::7 115::1 12::94 11::98 0.88431951 1 IR

AT2G31900 chr2:13563546-13563556 27 6::7 3::11 15::15 18::18 0.884382699 1 A3SS

AT4G18120 chr4:10040891-10040982 8 15::187 20::371 103::113 194::189 0.884461924 1 IR

AT1G16840 chr1:5762958-5763091 2 305::276 279::318 51::299 41::361 0.884514901 1 IR

AT2G31900 chr2:13561169-13561536 37 7::25 17::28 18::23 17::39 0.884630952 1 Cassette_multi

AT1G01650 chr1:236285-236296 5 3::128 15::125 127::142 131::151 0.884800403 1 A3SS

AT1G24880 chr1:8782660-8782682 8 30::67 89::166 3::5 5::3 0.884961432 1 A3SS

AT5G08570 chr5:2778121-2778324 1 346::53 324::54 42::154 39::149 0.88501101 1 A5SS

AT5G03440 chr5:857902-857985 2 70::42 63::66 62::116 73::123 0.885076239 1 A3SS

AT5G64940 chr5:25953427-25953482 20 1379::408 1747::422 1640::1163 2145::1630 0.885336559 1 A3SS

AT5G08270 chr5:2664977-2664982 5 1413::23 1124::11 218::1579 174::1444 0.885543777 1 A5SS

AT5G23200 chr5:7808184-7808189 4 16::65 22::79 27::101 29::130 0.885764435 1 A3SS

AT5G18830 chr5:6279593-6279671 9 15::166 4::189 151::154 171::148 0.885769999 1 A5SS

AT3G17609 chr3:6024034-6024108 1 209::79 308::151 63::404 108::512 0.885833051 1 IR

AT5G62130 chr5:24952053-24952509 2 264::0 216::0 19::269 25::202 0.885834828 1 Cassette_multi

AT2G22980 chr2:9782998-9783003 14 27::2 80::0 32::16 66::34 0.885873183 1 A3SS

AT1G77080 chr1:28959578-28959627 8 199::0 345::1 10::131 17::233 0.885898046 1 Cassette

AT5G42080 chr5:16821465-16821570 12 1116::20 1140::32 30::1205 35::1205 0.885954518 1 IR

AT1G60505 chr1:22295139-22295634 2 9::39 36::61 15::33 23::69 0.886024937 1 IR

AT1G18773 chr1:6475381-6475613 3 44::14 42::24 59::38 89::59 0.886071697 1 A3SS

AT5G02810 chr5:640085-640202 5-6 23::23 59::35 15::67 29::102 0.886182438 1 Cassette

AT4G17420 chr4:9722536-9722616 10 0::168 0::177 74::67 99::56 0.8862027 1 IR

AT5G17210 chr5:5657343-5657352 2 58::0 58::0 52::81 54::71 0.886497118 1 A3SS

AT3G09250 chr3:2840254-2840356 3 0::258 0::293 139::1029 167::1137 0.886592661 1 IR

AT5G65670 chr5:26256091-26256097 6 1935::46 2478::95 2015::431 2595::511 0.886776323 1 A3SS

AT3G05280 chr3:1505397-1505418 2 282::14 207::21 62::285 52::228 0.88685927 1 A3SS

AT4G00590 chr4:253839-253931 6 11::63 7::116 35::59 63::74 0.887000115 1 IR

AT5G11910 chr5:3837083-3837143 5 80::13 146::11 89::143 147::171 0.887184183 1 A5SS

AT2G18440 chr2:7995974-7996357 2 27::1317 95::1935 353::1089 498::1447 0.887201699 1 IR

AT3G46660 chr3:17189845-17190004 2 12::22 26::25 12::17 13::26 0.887233835 1 IR

AT4G16150 chr4:9149869-9149878 7 113::16 85::8 86::107 78::97 0.887258449 1 A3SS

AT2G16700 chr2:7244798-7244815 2 2::215 3::162 71::340 52::309 0.887276638 1 A3SS

AT5G19940 chr5:6740132-6740140 2 6316::103 6032::160 1185::7090 1340::6786 0.887406532 1 A5SS

AT4G25170 chr4:12910413-12910457 2 488::0 483::0 19::485 14::494 0.887464978 1 Cassette

AT5G02680 chr5:607289-607436 6-7 0::68 0::156 46::55 102::115 0.887537342 1 Cassette

AT3G55070 chr3:20410293-20410436 5 160::19 181::27 17::202 23::229 0.887554174 1 IR

AT4G27610 chr4:13787373-13788084 1 46::37 48::32 18::96 17::101 0.887632634 1 IR

AT5G65210 chr5:26057880-26057983 1 4::170 6::177 99::34 107::37 0.887653804 1 IR

AT3G14310 chr3:4773628-4773756 2 16::1008 15::1266 492::519 587::634 0.887765355 1 IR

AT1G30282 chr1:10663338-10663424 1 8::3 9::8 4::19 6::19 0.88791279 1 IR

AT3G59210 chr3:21889781-21889910 2 16::10 7::11 22::51 42::61 0.887918215 1 A3SS

AT1G60550 chr1:22307268-22307278 3 10::807 7::794 736::823 726::849 0.888115184 1 A3SS

AT3G63445 chr3:23425861-23425944 1 76::34 122::46 22::109 33::128 0.888170294 1 IR

AT3G55770 chr3:20703415-20703441 1 1554::9 1380::5 261::364 223::324 0.888187489 1 A5SS

AT3G27260 chr3:10070646-10070669 7 54::16 78::12 75::99 130::161 0.888195164 1 A3SS

AT3G06778 chr3:2140594-2140674 4 9::10 6::15 26::30 33::40 0.888262667 1 A3SS

AT5G39940 chr5:15988279-15988392 4 12::39 8::78 23::36 50::56 0.888575747 1 Cassette

AT2G20440 chr2:8813466-8813523 2 18::0 40::0 3::40 3::53 0.888616844 1 AltStart

AT1G45248 chr1:17163346-17163420 5 10::47 4::53 25::35 30::37 0.889040184 1 Cassette

AT3G25800 chr3:9424310-9424315 6 257::9 269::7 203::327 204::286 0.889106992 1 A5SS

AT4G27520 chr4:13750924-13750991 2 85::9090 0::7224 4861::3541 3870::2756 0.889180437 1 IR

AT1G10500 chr1:3460261-3460302 3 30::1131 35::1258 1202::400 1329::451 0.889264609 1 A3SS

AT2G20950 chr2:9004544-9004626 7 153::37 162::42 35::168 33::216 0.889374074 1 IR

AT2G46610 chr2:19137831-19138387 2 9::29 5::34 29::70 31::61 0.889518045 1 A5SS

AT2G47860 chr2:19600194-19600203 2 7::38 8::44 47::50 41::59 0.889523654 1 A5SS

AT1G10360 chr1:3396080-3396100 2 29::1007 19::781 1055::804 925::797 0.889536423 1 A3SS

AT2G01140 chr2:95637-95645 4 3::3095 19::3239 2169::3083 2353::3833 0.889618395 1 A3SS

AT5G59160 chr5:23881111-23881387 3 621::17 814::40 21::354 33::423 0.889690744 1 IR

AT1G17710 chr1:6091704-6091829 1 593::57 661::50 58::510 61::614 0.889747608 1 IR

AT1G52000 chr1:19335028-19335112 3 116::70 47::1 1100::1116 362::322 0.889826876 1 A5SS

AT2G46450 chr2:19066626-19066631 4 7::193 19::289 100::53 150::76 0.889862643 1 A5SS

AT2G26810 chr2:11436084-11436214 1 14::10 27::8 9::13 11::24 0.889882594 1 IR

AT3G05210 chr3:1479857-1479874 1 7::80 14::136 93::117 142::127 0.889914109 1 A5SS

AT1G26440 chr1:9145696-9145985 1 5::8 29::25 7::32 23::68 0.889954178 1 IR

AT2G35380 chr2:14892860-14893070 1 60::19 38::23 12::73 9::43 0.890064856 1 IR

AT1G78580 chr1:29557661-29557964 1-2 50::20 53::13 7::147 6::148 0.890142008 1 AltStart

AT4G08480 chr4:5388655-5388687 7 17::50 7::53 45::61 64::89 0.890157965 1 A3SS

AT1G08310 chr1:2619397-2619406 3 6::39 18::51 14::76 48::126 0.890174138 1 A3SS

AT2G21660 chr2:9265716-9265740 2 19::2 62::76 7600::5839 11687::8396 0.890292082 1 A5SS

AT5G20120 chr5:6795762-6795865 2 18::286 9::311 136::193 155::180 0.89037413 1 IR

AT2G04780 chr2:1677039-1677469 1 619::12 532::16 18::777 22::744 0.890567269 1 IR

AT2G36410 chr2:15280190-15280199 4 113::213 113::216 124::76 131::83 0.89056914 1 A3SS

AT1G31950 chr1:11477932-11478040 7 16::114 19::130 67::64 66::78 0.890604949 1 IR

AT5G10350 chr5:3256847-3256927 3-4 465::8 696::14 24::367 34::580 0.890752605 1 AltEnd

AT1G71310 chr1:26878852-26878860 4 11::139 9::151 173::190 193::246 0.890753334 1 A3SS

AT5G03500 chr5:877666-877686 2 116::6 159::15 34::179 35::191 0.890835298 1 A3SS

AT4G31010 chr4:15107913-15108000 3 78::1 90::14 4::89 12::105 0.890851903 1 IR

AT3G46830 chr3:17248518-17248615 1 160::19 202::12 140::44 150::33 0.891089628 1 A5SS

AT5G61530 chr5:24742364-24742690 3 77::0 91::0 115::145 121::169 0.891259212 1 MXE

AT2G44980 chr2:18554175-18554206 10 0::31 0::42 16::20 22::25 0.891297005 1 IR

AT4G39460 chr4:18358800-18358844 1 307::4 308::3 249::15 238::15 0.891572743 1 A5SS

AT1G26850 chr1:9303845-9303909 1 290::156 195::101 322::196 220::143 0.89159302 1 A5SS

AT5G14430 chr5:4654198-4654227 4 14::399 24::437 69::518 82::575 0.891624255 1 A3SS

AT5G05730 chr5:1720612-1720700 6 191::1 146::10 19::160 19::161 0.891630788 1 IR

AT3G16470 chr3:5596747-5596884 5 45::935 51::862 1120::1710 1008::1726 0.891697997 1 A3SS

AT4G15540 chr4:8873848-8873933 3 47::117 47::124 187::174 261::268 0.891741083 1 A5SS

AT5G11910 chr5:3836993-3837061 6 81::12 146::10 23::94 32::204 0.891823344 1 A3SS

AT5G11710 chr5:3775468-3775542 10 12::733 5::940 386::450 508::588 0.891885538 1 IR

AT5G13790 chr5:4450310-4450319 3 14::14 8::10 22::30 16::22 0.891912049 1 A3SS

AT1G21930 chr1:7713453-7713515 2 7::611 10::721 364::224 418::333 0.89194903 1 Cassette

AT1G73430 chr1:27604450-27604463 4 11::85 9::65 35::68 30::72 0.891960616 1 A3SS

AT2G19280 chr2:8365104-8365349 3 21::7 24::17 3::25 8::33 0.891988787 1 IR

AT2G03890 chr2:1186498-1186859 2 0::1267 0::930 649::643 486::565 0.892389238 1 IR

AT4G16920 chr4:9519606-9519629 7 24::0 23::0 12::29 29::40 0.892528117 1 A3SS

AT1G13080 chr1:4459569-4459643 1 1::667 7::540 395::300 305::244 0.892574578 1 IR

AT1G80690 chr1:30329881-30329893 2 34::179 25::230 90::194 99::239 0.892659749 1 A5SS

AT3G46490 chr3:17116984-17117285 4-5 58::54 77::95 43::101 59::160 0.892735699 1 Cassette_multi

AT1G22910 chr1:8107593-8107613 6 117::143 112::131 157::165 147::192 0.892740296 1 A3SS

AT2G24150 chr2:10266909-10266924 2 13::556 24::529 213::620 214::590 0.892804177 1 A5SS

AT5G08710 chr5:2836188-2836205 7 38::19 73::19 28::61 39::88 0.892849877 1 A3SS

AT5G04460 chr5:1263013-1263023 5 15::103 12::117 81::76 96::105 0.892908368 1 A5SS

AT1G28685 chr1:10079033-10079092 3 264::5 239::5 29::208 32::198 0.892923901 1 A3SS

AT1G71528 chr1:26942018-26942100 1 9::19 9::35 15::16 24::23 0.892963559 1 IR

AT3G05560 chr3:1614268-1614491 1 4772::4 4312::24 65::1900 82::1666 0.893022042 1 A5SS

AT1G69935 chr1:26342175-26342191 4 21::336 40::375 391::215 423::273 0.893165231 1 A3SS

AT4G38290 chr4:17942617-17942695 before first 292::26 314::31 13::272 24::268 0.893270131 1 Cassette

AT2G40830 chr2:17042881-17042892 2 271::9 191::4 13::399 9::390 0.893286269 1 Cassette

AT5G04930 chr5:1448260-1448269 4 125::13 169::22 90::151 117::217 0.893483028 1 A3SS

AT2G06050 chr2:2361983-2362127 1 110::31 94::32 19::111 19::99 0.893685667 1 IR

AT2G28470 chr2:12173403-12173422 1 95::16 38::9 130::73 56::33 0.893768327 1 A5SS

AT3G04500 chr3:1212566-1212735 4 228::21 265::33 42::286 50::294 0.893837355 1 A3SS

AT1G52000 chr1:19334943-19335028 3 116::2170 47::734 1157::1228 391::412 0.893837622 1 IR

AT3G62720 chr3:23201197-23201296 1 0::21 2::46 11::133 22::147 0.893891629 1 IR

AT3G17100 chr3:5831383-5831466 1 705::342 446::273 228::794 179::663 0.893916819 1 IR

AT2G01260 chr2:136170-136255 4 6::308 1::424 174::157 210::212 0.893918832 1 Cassette

AT1G08650 chr1:2753125-2753175 2 831::0 1419::0 23::755 41::1133 0.893970119 1 Cassette

AT2G24020 chr2:10218979-10218985 2 10::894 6::867 673::1003 653::960 0.893977791 1 A3SS

AT3G43520 chr3:15407256-15407271 2 32::968 54::1132 263::1035 316::1252 0.894032772 1 A3SS

AT3G17185 chr3:5861605-5861998 1 941::22 1038::45 22::459 29::529 0.894071126 1 IR

AT3G53610 chr3:19878266-19878390 1 406::96 349::72 49::252 44::209 0.894135822 1 IR

AT1G54700 chr1:20417023-20417128 3 156::6 160::5 13::152 13::178 0.894338736 1 IR

AT1G68568 chr1:25747214-25747224 2 2::250 6::118 227::273 94::109 0.894378108 1 A3SS

AT5G20150 chr5:6803032-6803116 3 3::3596 15::5879 1833::1904 3015::3375 0.894642344 1 IR

AT1G19230 chr1:6646476-6646555 5 0::23 0::23 13::15 16::10 0.894656959 1 IR

AT4G11090 chr4:6765523-6765528 2 13::26 5::37 19::59 19::56 0.8946576 1 A5SS

AT4G08290 chr4:5240329-5240404 5 250::18 260::11 29::177 29::197 0.894713934 1 IR

AT5G60230 chr5:24250946-24251319 1 42::9 19::6 6::46 5::47 0.894772134 1 IR

AT1G64650 chr1:24026020-24026186 1 290::11 333::13 21::299 25::287 0.894980207 1 IR

AT2G45670 chr2:18817206-18817220 4 361::81 299::88 188::506 178::451 0.895087728 1 A3SS

AT4G38260 chr4:17937221-17937238 5 13::207 3::241 226::152 255::178 0.895150938 1 A3SS

AT2G46230 chr2:18984651-18984742 7 506::13 679::16 51::514 67::711 0.895246397 1 IR

AT2G44280 chr2:18303985-18304071 1 6::10 2::13 8::11 10::12 0.895372871 1 IR

AT4G24160 chr4:12541793-12541866 7 187::38 180::29 39::193 36::184 0.895473133 1 IR

AT3G51100 chr3:18981618-18981648 2 5::171 9::179 158::144 187::221 0.89547944 1 A3SS

AT2G44920 chr2:18525608-18525646 3 865::23 848::25 103::710 103::721 0.895538273 1 A5SS

AT3G22420 chr3:7948263-7948345 6 128::58 127::101 45::181 71::229 0.895714434 1 IR

AT4G12040 chr4:7214921-7215315 1 567::20 415::27 23::759 21::752 0.895784423 1 IR

AT3G13224 chr3:4255855-4256082 5 52::52 74::87 24::73 48::127 0.895838916 1 IR

AT5G61910 chr5:24861674-24861708 5 8::224 10::216 110::98 111::105 0.895914557 1 IR

AT3G20070 chr3:7006706-7006734 1 15::23 24::21 42::33 40::26 0.895973127 1 A5SS

AT2G43910 chr2:18185018-18185023 8 3154::14 4424::9 1899::3939 2638::5392 0.895989198 1 A5SS

AT5G47840 chr5:19375730-19375738 2 15::851 19::788 217::796 233::794 0.896141921 1 A3SS

AT3G63400 chr3:23412428-23412447 2 53::87 31::74 69::144 42::131 0.896211766 1 A3SS

AT4G11830 chr4:7117294-7117343 8 12::54 16::126 55::63 124::109 0.896242855 1 A3SS

AT3G26744 chr3:9834840-9834888 2 1::78 1::74 147::327 159::316 0.896292662 1 A3SS

AT4G37630 chr4:17679991-17679997 3 26::12 26::23 41::34 49::64 0.896373606 1 A3SS

AT1G77810 chr1:29261450-29261468 9 22::0 45::4 7::46 15::46 0.896394412 1 A3SS

AT3G29290 chr3:11240126-11240223 2 9::114 12::216 68::113 116::149 0.896401147 1 IR

AT5G12840 chr5:4053354-4053442 1 59::149 35::156 85::123 80::122 0.896439717 1 IR

AT4G38600 chr4:18045272-18045326 8 240::0 175::0 263::177 195::144 0.896626483 1 A5SS

AT3G22550 chr3:7992078-7992084 2 469::8 556::15 466::647 542::658 0.896630136 1 A5SS

AT2G45245 chr2:18659583-18659693 4 4::160 11::168 90::52 88::50 0.896670971 1 IR

AT5G47880 chr5:19386347-19386425 2 531::0 632::16 35::326 50::365 0.896753479 1 IR

AT5G19430 chr5:6554906-6554933 3 16::404 8::384 102::428 120::479 0.896803362 1 A3SS

AT3G13560 chr3:4427313-4427324 2 48::11 45::8 24::76 21::65 0.896804273 1 A3SS

AT2G31370 chr2:13381092-13381103 6 199::11 247::22 88::246 114::289 0.896919106 1 A5SS

AT2G09795 chr2:3672125-3672206 1 97::286 88::357 172::212 206::239 0.896944142 1 IR

AT1G06240 chr1:1912148-1912156 4 13::180 25::208 13::181 18::215 0.897009188 1 A3SS

AT1G12440 chr1:4242359-4242808 1 1183::107 1039::103 39::1275 32::1265 0.897013016 1 IR

AT1G60270 chr1:22223396-22223483 6 8::57 11::129 37::56 78::106 0.897038304 1 Cassette

AT1G71860 chr1:27028763-27028769 9 428::9 520::7 243::149 309::196 0.89706885 1 A3SS

AT4G36090 chr4:17078949-17079046 4 40::2 59::25 5::42 14::69 0.897085077 1 IR

AT2G11890 chr2:4803296-4803408 1 9::1252 1::1290 682::794 728::827 0.897160476 1 IR

AT4G35000 chr4:16665905-16665910 7 14::2594 3::2443 1885::2853 1786::2690 0.89723046 1 A3SS

AT2G24060 chr2:10229821-10229826 2 480::13 551::7 513::485 570::533 0.897364586 1 A3SS

AT1G50000 chr1:18516410-18516415 3 17::49 14::34 50::84 37::83 0.897383186 1 A3SS

AT2G40460 chr2:16897310-16897344 2 20::93 2::56 38::189 23::112 0.897481396 1 A3SS

AT1G79270 chr1:29815873-29816190 1 63::55 18::22 15::6 12::3 0.897508693 1 A5SS

AT4G31410 chr4:15245474-15245551 3 134::92 155::79 55::83 53::118 0.897552289 1 IR

AT1G19330 chr1:6681500-6681515 3 146::65 159::72 104::244 140::272 0.897554237 1 A3SS

AT5G57930 chr5:23454820-23454829 2 1002::4 892::3 624::777 539::669 0.897641094 1 A3SS

AT2G38880 chr2:16238811-16238884 2 9::280 12::269 297::338 245::313 0.897647559 1 A5SS

AT5G59780 chr5:24082386-24082493 3 22::595 17::1038 371::405 633::693 0.897658394 1 IR

AT1G06410 chr1:1954951-1955031 1-2 88::9 51::9 10::267 11::251 0.89766487 1 AltStart

AT4G09680 chr4:6118564-6118572 5 29::6 15::4 12::38 9::23 0.897692892 1 A5SS

AT4G35920 chr4:17014761-17014818 2 191::11 132::15 9::256 14::268 0.897796937 1 AltStart

AT3G05165 chr3:1458136-1458408 19 0::576 0::774 265::253 346::255 0.897814059 1 AltEnd

AT5G44920 chr5:18139421-18139467 1 41::69 52::59 83::90 83::78 0.897913178 1 A5SS

AT1G78865 chr1:29648196-29649385 2 63::14 18::13 8::30 6::14 0.898127313 1 IR

AT2G02560 chr2:690463-690469 3 27::22 18::10 26::65 19::39 0.898216128 1 A3SS

AT3G58050 chr3:21494049-21494074 6 16::11 9::23 17::37 14::40 0.898222443 1 A3SS

AT4G35350 chr4:16811563-16811660 3 204::9 202::18 20::200 27::228 0.898255448 1 IR

AT3G13225 chr3:4262166-4262448 2 21::3 17::3 21::34 21::37 0.898299985 1 A5SS

AT2G45830 chr2:18866503-18866607 1 7::24 31::23 15::30 17::49 0.898331819 1 IR

AT4G26555 chr4:13404849-13404859 7 35::442 33::510 473::564 527::623 0.898361986 1 A3SS

AT3G06720 chr3:2123047-2123069 9 8::392 8::438 390::402 443::446 0.898437588 1 A5SS

AT5G02740 chr5:617717-618135 5 605::6 539::8 11::351 8::340 0.898530502 1 IR

AT1G19400 chr1:6712536-6712981 2 46::703 39::606 34::503 20::391 0.898546236 1 A5SS

AT3G04810 chr3:1320129-1320214 15 17::284 24::447 169::154 253::240 0.898834863 1 IR

AT4G39270 chr4:18277300-18277386 1 14::112 8::187 61::64 98::93 0.899116296 1 IR

AT3G58620 chr3:21681906-21681917 4 13::45 20::43 21::120 31::97 0.899389751 1 A3SS

AT5G57565 chr5:23310346-23310792 1 0::12 0::23 42::12 53::19 0.899558878 1 IR

AT4G14300 chr4:8232877-8233100 2 25::352 52::372 139::545 138::571 0.899561712 1 IR

AT4G12720 chr4:7487584-7487594 2 5::114 8::108 116::114 120::112 0.899709699 1 A5SS

AT5G66180 chr5:26450425-26450524 7 140::35 174::44 28::135 33::171 0.899733391 1 IR

AT3G52570 chr3:19501865-19501870 3 8::39 23::48 27::70 44::75 0.899759361 1 A3SS

AT2G32320 chr2:13730181-13730252 11 5::16 19::36 15::36 31::61 0.899882624 1 A3SS

AT5G11790 chr5:3800978-3800989 4 43::296 45::299 105::422 109::420 0.899971774 1 A3SS

AT1G06690 chr1:2051190-2051232 4 20::297 13::280 52::294 51::244 0.900009434 1 A5SS

AT5G40850 chr5:16368555-16368575 6 19::586 21::764 135::386 188::537 0.900029155 1 A3SS

AT5G15270 chr5:4960974-4961135 7 62::59 145::82 30::67 49::101 0.900240158 1 IR

AT3G46210 chr3:16978782-16978865 1 93::13 86::24 89::19 92::20 0.900354186 1 A5SS

AT1G79500 chr1:29906013-29906288 14 39::145 38::224 64::75 99::89 0.900508279 1 IR

AT4G01210 chr4:510434-510444 6 7::74 8::36 35::81 26::68 0.900542121 1 A5SS

AT1G75850 chr1:28482709-28482774 6 56::24 44::25 31::68 31::62 0.900656351 1 A3SS

AT4G27010 chr4:13565184-13565251 7 20::0 34::0 20::18 40::33 0.900666207 1 A3SS

AT2G31110 chr2:13258894-13258916 5 144::9 143::10 146::148 144::155 0.900876715 1 A5SS

AT5G61540 chr5:24745782-24745881 3 19::15 17::42 9::40 19::64 0.900979538 1 IR

AT5G16300 chr5:5342034-5342039 6 167::0 229::0 87::108 115::159 0.901024022 1 A3SS

AT1G66730 chr1:24889194-24889291 9 0::18 19::33 10::16 25::40 0.901090334 1 Cassette

AT5G65260 chr5:26081317-26081755 3-4 877::29 898::22 18::898 22::964 0.901515049 1 Cassette

AT5G19660 chr5:6644129-6644134 7 15::29 11::27 26::65 28::93 0.901580316 1 A3SS

AT5G06450 chr5:1968404-1968503 2 9::81 11::104 109::136 134::155 0.90165179 1 A3SS

AT5G19221 chr5:6466215-6466237 5-6 1::894 7::657 746::492 574::426 0.901887044 1 Cassette

AT4G19420 chr4:10588972-10588992 6 104::281 122::290 342::522 356::535 0.901939068 1 A3SS

AT5G38590 chr5:15452628-15453021 2 19::23 32::21 24::63 33::64 0.901970055 1 A5SS

AT1G12230 chr1:4148910-4148975 4 558::0 505::2 7::524 15::470 0.902034964 1 Cassette

AT5G58140 chr5:23527727-23527737 12 876::14 765::25 977::1033 861::982 0.902059819 1 A3SS

AT1G57610 chr1:21338480-21338627 1 50::132 77::132 71::114 73::146 0.902077953 1 IR

AT1G26850 chr1:9301631-9301672 5 1314::0 1555::0 1201::1203 1401::1240 0.902281059 1 A5SS

AT5G13000 chr5:4113473-4113527 34 165::0 212::0 173::240 213::217 0.902307122 1 A5SS

AT4G33050 chr4:15945161-15945247 7 36::3 129::5 7::48 18::115 0.902511611 1 IR

AT3G46450 chr3:17093112-17093286 7 10::575 9::607 257::300 303::287 0.90262914 1 IR

AT4G29940 chr4:14649235-14649315 12 0::44 0::31 25::18 17::24 0.902672612 1 Cassette

AT1G23960 chr1:8479983-8480017 2 18::265 11::264 256::257 247::230 0.902685057 1 A3SS

AT5G64360 chr5:25736775-25736890 3 4::20 3::38 46::37 77::70 0.902790597 1 A3SS

AT2G32540 chr2:13816939-13816944 7 278::14 280::11 242::291 251::327 0.902845387 1 A3SS

AT2G18230 chr2:7934120-7934200 1-2 364::16 305::4 18::474 12::412 0.90289085 1 AltStart

AT3G58510 chr3:21641620-21641906 4 21::239 11::305 28::300 27::355 0.902892934 1 A3SS

AT1G15380 chr1:5290819-5290937 1 0::32 0::67 20::20 35::48 0.902916181 1 IR

AT5G55300 chr5:22426466-22426591 8 0::124 0::136 64::54 87::40 0.902923413 1 Cassette

AT3G01100 chr3:35555-35852 9 105::19 119::27 6::100 15::126 0.903202908 1 IR

AT2G44798 chr2:18467971-18468014 4 6::48 5::35 29::18 22::25 0.903217286 1 Cassette

AT4G18020 chr4:10007393-10007398 1 41::40 37::60 36::27 35::27 0.903232694 1 A5SS

AT2G41600 chr2:17345768-17345993 2 159::61 265::113 24::189 55::258 0.903505819 1 IR

AT5G43470 chr5:17462997-17463003 6 82::147 136::224 149::137 234::253 0.903599999 1 A3SS

AT1G34030 chr1:12371037-12371197 2 14::5056 38::5647 2667::2298 3171::2785 0.903605459 1 Cassette

AT1G55250 chr1:20609957-20609985 11 23::0 33::0 3::32 8::47 0.903644318 1 Cassette

AT5G27770 chr5:9835994-9836074 1-2 3370::24 3482::12 41::2425 22::2681 0.903701303 1 AltStart

AT2G37440 chr2:15720550-15720599 7 0::16 0::29 9::9 15::13 0.903764651 1 IR

AT1G65950 chr1:24549310-24549376 7 24::3 33::17 5::25 15::46 0.903803458 1 Cassette

AT5G50810 chr5:20676060-20676088 2 1098::30 1356::51 89::682 149::894 0.903804076 1 A3SS

AT1G14560 chr1:4981841-4981925 3 18::110 20::185 60::90 100::122 0.90400542 1 IR

AT4G09010 chr4:5778031-5778048 8 20::2494 10::2252 2403::2311 2179::2269 0.904026645 1 A3SS

AT5G04510 chr5:1288311-1288355 6 10::82 22::77 30::104 48::115 0.904043107 1 A3SS

AT4G26110 chr4:13235027-13235431 11 1030::9 1248::33 11::410 28::588 0.904045572 1 IR

AT2G42770 chr2:17799296-17799321 2 10::622 12::602 570::711 563::735 0.904096228 1 A3SS

AT1G31160 chr1:11123146-11123193 5 394::40 500::30 63::409 68::568 0.904376659 1 A3SS

AT4G22233 chr4:11763884-11763914 1 93::0 146::13 107::149 151::193 0.904550712 1 A5SS

AT5G13220 chr5:4219629-4219684 3 4::43 4::32 39::34 43::36 0.90473449 1 A5SS

AT4G12917 chr4:7563621-7563632 2 11::116 5::152 167::113 194::111 0.904769558 1 A3SS

AT4G31200 chr4:15164617-15164832 1 7::6 13::12 6::71 7::59 0.904839879 1 IR

AT1G10820 chr1:3601971-3601977 8 18::97 28::142 88::104 136::165 0.90484445 1 A3SS

AT3G27340 chr3:10122539-10122568 4 1061::30 878::25 982::400 813::348 0.904968656 1 A3SS

AT4G03820 chr4:1772127-1772225 4 41::7 60::23 6::23 14::36 0.905198715 1 IR

AT2G43190 chr2:17956062-17956199 1 40::7 50::3 5::29 4::39 0.90530891 1 IR

AT3G14830 chr3:4983174-4983299 1 48::6 28::7 10::69 7::71 0.905380186 1 IR

AT3G02020 chr3:340970-340981 12 999::11 1502::9 751::932 1162::1410 0.905529909 1 A5SS

AT3G27740 chr3:10282876-10283082 4 0::1359 0::1251 648::573 584::525 0.905600885 1 Cassette_multi

AT1G70850 chr1:26715929-26716051 2 269::15 182::0 16407::7238 12329::5670 0.905618675 1 Cassette

AT5G06960 chr5:2155661-2155677 2 35::2 35::4 37::50 33::44 0.905793903 1 A3SS

AT1G21560 chr1:7555183-7555370 2 32::1 48::0 41::86 68::100 0.905891688 1 A3SS

AT5G51460 chr5:20902906-20902919 4 137::13 171::15 129::133 167::166 0.905899951 1 A3SS

AT1G45249 chr1:17165935-17166069 3 279::57 251::60 49::250 61::219 0.90591426 1 Cassette

AT5G19660 chr5:6645037-6645047 4 26::22 42::13 38::53 34::53 0.905935892 1 A3SS

AT5G57180 chr5:23170124-23170129 2 405::21 431::12 283::203 310::213 0.905945879 1 A3SS

AT5G45428 chr5:18408984-18409059 3 14::961 4::972 511::514 515::472 0.906331439 1 IR

AT5G65180 chr5:26045700-26045887 1 49::4 39::20 5::41 7::38 0.906377245 1 IR

AT4G11845 chr4:7127136-7127179 4 3::21 4::46 29::41 47::41 0.90649043 1 A3SS

AT3G21100 chr3:7402074-7402089 9 7::33 20::42 23::35 33::61 0.906567274 1 A3SS

AT1G68568 chr1:25747366-25747383 1 250::2 118::6 272::236 133::122 0.906582222 1 A5SS

AT2G32640 chr2:13848443-13848507 11 82::61 77::81 75::139 126::190 0.906686396 1 A3SS

AT1G08970 chr1:2883848-2883916 2 289::5 429::1 253::693 234::716 0.907099752 1 A5SS

AT3G15030 chr3:5063681-5063692 3 183::0 161::2 168::147 154::181 0.907206699 1 A3SS

AT2G33051 chr2:14023807-14023911 3 4::169 19::233 82::91 130::179 0.907332246 1 IR

AT3G29575 chr3:11383675-11383924 1 22::141 22::56 41::320 17::201 0.907356917 1 IR

AT1G55500 chr1:20720570-20720609 5 48::34 29::32 54::103 44::107 0.907414526 1 A3SS

AT4G27620 chr4:13790750-13790908 1 7::52 7::45 13::25 7::27 0.907539452 1 A5SS

AT2G34410 chr2:14522718-14522727 16 13::17 10::0 156903::12855 198332::15692 0.9075508 1 A5SS

AT4G11570 chr4:7004657-7004686 2 17::6 3::9 550::801 484::752 0.907565351 1 A3SS

AT5G03440 chr5:857544-857552 3 94::9 131::6 62::123 75::123 0.907599526 1 A5SS

AT4G18375 chr4:10154805-10154830 7 13::0 35::5 20::17 42::38 0.907602439 1 A3SS

AT5G19130 chr5:6418246-6418251 4 17::31 10::45 17::74 22::90 0.907612166 1 A3SS

AT3G56210 chr3:20854143-20854149 6 170::15 197::30 49::196 66::202 0.907645978 1 A3SS

AT1G79090 chr1:29753004-29753083 1 68::22 82::12 19::68 14::62 0.907678962 1 IR

AT1G30620 chr1:10854990-10855050 2 24::7 31::1 5::38 3::24 0.907827717 1 Cassette

AT5G20450 chr5:6912770-6912811 1-2 0::13 0::28 13::31 33::39 0.907984622 1 Cassette

AT2G47650 chr2:19540736-19540741 1 158::14 194::7 66::320 79::326 0.908034199 1 A5SS

AT5G64520 chr5:25790090-25790219 8 3::13 13::18 6::14 11::22 0.908217016 1 IR

AT3G63440 chr3:23425043-23425170 2 14::94 18::99 58::72 58::94 0.908283286 1 Cassette

AT3G08990 chr3:2742970-2742996 1 4::11 14::10 6::44 13::60 0.908339696 1 AltStart

AT5G57940 chr5:23456667-23456897 2 98::13 88::10 19::201 15::312 0.908347445 1 AltStart

AT3G06760 chr3:2133901-2133912 6 211::0 235::0 246::302 294::344 0.908378382 1 A3SS

AT3G58510 chr3:21640171-21640277 1 84::4 97::11 12::51 13::46 0.908389764 1 A5SS

AT3G24120 chr3:8706335-8706344 6 253::45 273::54 138::252 163::277 0.908406645 1 A3SS

AT3G59330 chr3:21925941-21926135 7 4::40 0::88 35::34 83::56 0.908447613 1 Cassette

AT3G25570 chr3:9288882-9288915 3 22::41 33::50 52::101 77::118 0.908450354 1 A3SS

AT3G45240 chr3:16573243-16573618 1 6::25 23::19 14::42 11::55 0.908485528 1 IR

AT5G43430 chr5:17454866-17454886 5 6::110 2::172 13::86 9::124 0.908608093 1 A3SS

AT2G14740 chr2:6312348-6312442 12 43::263 78::310 135::202 144::263 0.908656502 1 IR

AT5G60940 chr5:24523687-24523780 3 37::24 32::22 13::48 12::43 0.908886085 1 IR

AT1G42480 chr1:15937170-15937219 3 161::0 223::0 180::189 217::198 0.908987846 1 A3SS

AT3G52120 chr3:19329982-19330107 3 24::172 7::118 64::249 85::245 0.909030578 1 A3SS

AT5G64330 chr5:25729495-25729576 4 258::6 294::25 17::199 29::247 0.909105952 1 IR

AT1G69410 chr1:26089867-26089923 3 22::3075 7::2759 1429::1440 1319::1230 0.909121702 1 Cassette

AT2G45270 chr2:18668632-18668646 10 4::91 18::123 30::89 50::150 0.909142714 1 A3SS

AT2G02570 chr2:699137-699149 5 90::12 143::29 30::152 50::178 0.90921998 1 A3SS

AT2G26692 chr2:11350914-11350984 2 786::5 374::6 430::13 206::10 0.90927667 1 A3SS

AT1G30160 chr1:10606467-10606480 2 7::20 6::5 17::39 8::20 0.909485358 1 A3SS

AT2G33470 chr2:14177969-14177988 2 359::247 349::259 305::685 307::686 0.90949587 1 A3SS

AT4G37330 chr4:17563133-17563149 3 28::382 7::320 351::318 295::324 0.909632522 1 A3SS

AT1G08660 chr1:2757514-2757602 10 86::84 120::147 52::160 86::269 0.909678514 1 IR

AT2G01630 chr2:279936-280092 2 87::0 136::0 107::121 202::169 0.909708285 1 A5SS

AT2G44850 chr2:18499745-18499874 5 0::35 0::55 23::29 36::39 0.909768958 1 Cassette

AT5G35170 chr5:13423377-13423455 18 667::16 948::34 50::321 76::437 0.909834193 1 IR

AT5G53130 chr5:21540722-21540727 1 20::34 17::19 59::50 38::36 0.909889426 1 A5SS

AT2G45150 chr2:18614973-18615058 4 60::10 91::9 8::67 10::84 0.909984022 1 IR

AT3G56450 chr3:20931490-20931683 2-3 0::44 0::63 30::36 49::59 0.910037947 1 Cassette

AT3G13060 chr3:4181951-4182031 5 6::331 13::314 192::195 169::183 0.91004145 1 IR

AT1G17720 chr1:6094079-6094085 14 1::428 14::589 261::222 366::338 0.910075565 1 A3SS

AT1G15240 chr1:5248384-5248467 14 80::3 125::3 8::83 11::126 0.910133338 1 IR

AT3G16340 chr3:5541632-5541647 7 31::0 29::0 33::22 32::33 0.91016814 1 A3SS

AT1G24040 chr1:8505691-8505788 1 172::5 206::24 15::133 22::146 0.910216648 1 IR

AT3G47680 chr3:17578461-17578688 2 22::38 22::89 33::63 47::110 0.910257876 1 IR

AT4G02260 chr4:990662-990749 21 190::30 286::56 26::219 43::267 0.910273788 1 IR

AT4G32285 chr4:15585871-15585974 1 219::120 180::115 67::424 63::433 0.910365494 1 IR

AT3G07610 chr3:2431099-2431127 9 19::0 32::0 19::15 30::34 0.910602161 1 A3SS

AT4G16845 chr4:9479103-9479109 13 10::63 22::73 39::71 57::94 0.910726847 1 A3SS

AT5G03430 chr5:850862-850867 9 194::33 234::51 53::236 77::283 0.910734267 1 A3SS

AT5G05540 chr5:1638250-1638337 10 52::6 77::23 11::70 21::86 0.910839044 1 IR

AT3G19330 chr3:6700222-6700319 2 15::6 10::3 4::16 4::27 0.910846282 1 IR

AT1G60850 chr1:22398564-22398678 7 120::5 184::4 8::113 12::183 0.910919796 1 IR

AT5G15550 chr5:5059266-5059351 11 305::16 501::13 22::285 30::473 0.911071057 1 IR

AT2G41310 chr2:17223573-17223691 5 32::338 46::419 213::163 226::182 0.911098513 1 IR

AT2G02410 chr2:632874-632904 1 11::9 30::32 12::20 36::50 0.911148796 1 A5SS

AT5G10140 chr5:3174036-3174107 7 57::0 44::0 3::54 3::17 0.911183057 1 Cassette

AT5G10350 chr5:3256503-3256583 4 465::10 696::25 34::367 71::580 0.911241836 1 AltStart

AT3G46060 chr3:16917786-16917903 1 1024::47 948::23 71::612 59::552 0.911342297 1 IR

AT3G27280 chr3:10078063-10078264 2 392::1 493::18 16::379 28::453 0.911371552 1 IR

AT5G19855 chr5:6712279-6712291 5 1543::13 1415::9 452::938 404::958 0.911402039 1 A3SS

AT4G17250 chr4:9672977-9673106 1 0::117 0::169 64::53 86::41 0.91158587 1 IR

AT5G04690 chr5:1351202-1351261 3-4 0::18 0::34 12::22 18::17 0.911615669 1 Cassette

AT3G05640 chr3:1642849-1642890 1 26::27 8::12 30::30 13::12 0.911745169 1 A5SS

AT1G80490 chr1:30266508-30266540 2 52::0 43::0 10::44 4::49 0.911766421 1 A3SS

AT5G16940 chr5:5572216-5572223 2 3::49 4::38 40::78 35::91 0.911794503 1 A3SS

AT1G54680 chr1:20413319-20413360 2 43::0 59::0 51::55 49::30 0.911798684 1 A3SS

AT1G11400 chr1:3838016-3838083 2 295::14 364::15 13::263 29::312 0.911869392 1 AltStart

AT2G24590 chr2:10451067-10451180 6 238::145 267::133 67::509 67::684 0.911889191 1 IR

AT1G60590 chr1:22314821-22314838 7 573::6 303::10 153::500 99::305 0.911994512 1 A3SS

AT3G03780 chr3:957235-957289 2 882::2 379::4 6::1591 7::710 0.9120002 1 Cassette

AT5G63760 chr5:25516074-25516185 2 0::43 0::63 21::157 31::137 0.912057937 1 IR

AT1G29170 chr1:10190632-10190646 8 3::73 1::90 78::97 98::143 0.912238888 1 A3SS

AT2G40540 chr2:16931084-16931164 1-2 73::66 62::32 50::340 37::344 0.912245296 1 AltStart

AT4G34000 chr4:16295431-16295469 1 15::34 7::3 11::488 3::410 0.912311841 1 AltStart

AT2G21830 chr2:9305408-9305492 3 16::153 12::187 87::81 110::99 0.912316657 1 IR

AT1G75420 chr1:28305371-28305542 1 12::69 12::75 45::51 43::60 0.912351468 1 IR

AT2G33380 chr2:14145270-14145395 5 41::1296 7::576 638::538 268::253 0.912371654 1 Cassette

AT4G04850 chr4:2456846-2456940 16 638::40 751::33 54::620 58::721 0.912472993 1 IR

AT3G47390 chr3:17461562-17461642 3-4 12::5 24::14 7::64 15::97 0.91249395 1 AltEnd

AT5G56850 chr5:22985779-22985784 9 109::10 197::16 114::126 207::226 0.912802206 1 A5SS

AT5G08270 chr5:2662699-2662704 4 6::17 17::11 17::412 29::514 0.912813122 1 A3SS

AT1G05670 chr1:1697832-1697939 2 9::2 40::2 3::15 4::37 0.912848668 1 IR

AT5G08640 chr5:2805227-2805399 3 0::254 0::331 97::260 109::314 0.912946244 1 IR

AT3G19800 chr3:6876387-6876396 2 647::58 641::77 500::676 496::685 0.912979839 1 A3SS

AT1G67930 chr1:25475504-25475584 2 22::283 7::216 148::101 118::91 0.913024405 1 IR

AT5G57860 chr5:23438459-23438606 3 274::3 279::15 13::256 19::291 0.913064422 1 IR

AT4G34000 chr4:16295558-16295845 2 49::48 10::20 90::488 58::410 0.913210268 1 AltStart

AT4G27410 chr4:13708723-13708833 1 123::10 34::9 10::126 6::52 0.913330281 1 IR

AT3G05932 chr3:1771410-1771716 1 11::62 9::99 35::69 60::98 0.913358029 1 Cassette

AT3G56630 chr3:20979804-20979908 2 6::243 17::261 141::183 134::197 0.913413763 1 IR

AT4G26370 chr4:13334941-13335079 3 268::14 275::26 17::274 22::284 0.913490941 1 IR

AT5G64400 chr5:25749794-25750084 4 6976::71 7366::100 142::4767 167::5141 0.913556062 1 IR

AT1G26210 chr1:9067841-9068057 2 27::294 56::365 156::180 204::227 0.913657145 1 IR

AT2G40935 chr2:17083379-17083444 3 60::40 83::38 39::87 50::106 0.913689068 1 A5SS

AT4G29170 chr4:14383457-14383552 2 7::14 4::13 11::29 13::22 0.913801477 1 A3SS

AT5G15610 chr5:5081587-5081675 8 540::77 679::129 83::556 111::712 0.91389864 1 IR

AT1G03630 chr1:908033-908039 3 1353::37 825::24 772::1817 492::1288 0.91396191 1 A3SS

AT1G73350 chr1:27576479-27576519 3 67::59 63::78 83::142 100::189 0.913983992 1 A3SS

AT3G28690 chr3:10755619-10755910 1 133::32 130::12 20::137 17::131 0.914139951 1 IR

AT4G37440 chr4:17603664-17603669 6 195::0 286::0 115::106 165::166 0.914292834 1 A3SS

AT4G01995 chr4:873489-873501 3 53::157 77::147 91::217 101::232 0.914318254 1 A3SS

AT5G05360 chr5:1589030-1589135 2 319::12 377::20 30::171 41::184 0.914359655 1 IR

AT1G16260 chr1:5562029-5562081 1 0::59 0::42 37::70 24::69 0.914361843 1 IR

AT5G41700 chr5:16676886-16677080 4 11455::31 12778::26 426::5744 477::6323 0.914436143 1 IR

AT3G58610 chr3:21671460-21671539 1 1::1273 0::723 450::3028 270::1962 0.914488905 1 IR

AT5G11790 chr5:3801420-3801431 6 364::18 460::10 299::382 409::481 0.914596358 1 A3SS

AT2G05310 chr2:1933983-1933988 1 2905::37 3041::46 2686::1834 2815::1851 0.914653255 1 A5SS

AT5G59730 chr5:24065294-24065393 1 5::244 10::275 121::260 146::281 0.914663802 1 IR

AT4G37670 chr4:17696396-17696480 8 34::5 40::16 7::39 13::49 0.914684519 1 IR

AT5G66558 chr5:26566010-26566093 1 209::21 221::41 28::195 35::183 0.914711851 1 IR

AT2G27950 chr2:11903186-11903288 2 23::78 10::54 37::106 26::83 0.914769081 1 IR

AT1G74088 chr1:27862095-27862273 2 0::55 0::85 30::29 47::46 0.914816118 1 IR

AT5G65110 chr5:26009976-26010078 6 189::9 294::12 17::162 25::229 0.914899548 1 IR

AT3G15358 chr3:5178199-5178290 2 16::60 6::48 32::26 25::26 0.914926405 1 IR

AT5G42560 chr5:17016661-17016728 7 13::695 3::809 367::247 416::286 0.914926522 1 IR

AT3G57410 chr3:21244988-21245161 21 346::10 347::16 26::368 42::410 0.915054136 1 A3SS

AT3G44670 chr3:16217057-16217237 1 27::77 21::77 25::66 24::68 0.915116549 1 IR

AT5G53486 chr5:21722229-21722235 3 159::10 203::12 86::204 104::199 0.915215214 1 A3SS

AT2G31900 chr2:13562356-13563353 28 8::9 10::7 7::18 8::22 0.915255254 1 Cassette_multi

AT3G08680 chr3:2637854-2637935 1 0::314 4::189 166::154 104::96 0.915369249 1 IR

AT1G50630 chr1:18751790-18751875 3 71::13 74::17 9::74 16::67 0.915533806 1 IR

AT3G10915 chr3:3417456-3417489 1 15::8 11::10 64::104 82::108 0.915576919 1 A3SS

AT1G27760 chr1:9669342-9669354 3 355::6 411::12 289::414 329::449 0.915661379 1 A3SS

AT5G09240 chr5:2873958-2874043 3 103::33 173::44 28::61 34::96 0.915738461 1 IR

AT3G02710 chr3:584255-584260 1 10::150 8::121 109::129 92::108 0.915758063 1 A5SS

AT5G06710 chr5:2069042-2069165 2 53::4 54::6 4::59 7::56 0.915874528 1 IR

AT2G36010 chr2:15121390-15121396 8 22::0 31::9 15::31 15::52 0.916126425 1 A3SS

AT2G32320 chr2:13729206-13729242 8 27::0 25::0 11::28 11::34 0.916156449 1 A5SS

AT4G02210 chr4:976037-976055 2 25::7 23::5 10::73 8::92 0.916228693 1 AltStart

AT5G61030 chr5:24561715-24561740 5 0::1352 39::1785 691::476 910::632 0.916408083 1 IR

AT5G11060 chr5:3512934-3512942 6 223::6 226::14 135::145 131::165 0.916411551 1 A3SS

AT2G15910 chr2:6935087-6935097 1 503::0 621::0 500::542 632::658 0.9165468 1 A5SS

AT3G05685 chr3:1674079-1674143 3 30::0 57::0 47::35 63::59 0.916548924 1 A5SS

AT4G37430 chr4:17597776-17598126 1 71::38 109::51 36::132 50::153 0.916627858 1 A5SS

AT1G22630 chr1:8003418-8003513 1 78::1214 82::1219 568::652 558::734 0.916799813 1 IR

AT1G07745 chr1:2402458-2402755 1 21::13 26::21 10::27 17::38 0.91683622 1 IR

AT2G14285 chr2:6053244-6053404 2-3 484::24 591::21 37::424 66::535 0.916867369 1 Cassette

AT5G54080 chr5:21949925-21950014 11 6::10 5::27 19::15 32::30 0.916974652 1 A5SS

AT5G29000 chr5:11022924-11023060 6 55::9 99::7 8::41 9::58 0.917023773 1 IR

AT1G27360 chr1:9501973-9502077 2 4::28 8::18 17::53 11::78 0.917045566 1 IR

AT4G25690 chr4:13091233-13091703 1 79::16 92::10 18::176 17::229 0.917049025 1 IR

AT3G59310 chr3:21922917-21923011 7 57::17 87::23 13::62 17::91 0.917356324 1 IR

AT4G18120 chr4:10039057-10039073 4 48::6 98::13 42::58 84::103 0.917360693 1 A3SS

AT5G56730 chr5:22950873-22950957 6 24::0 17::0 24::30 19::19 0.917459447 1 A5SS

AT4G38545 chr4:18023875-18023956 3 160::75 187::69 49::178 46::221 0.917517651 1 IR

AT2G28540 chr2:12223333-12223420 13 4::311 3::467 186::132 271::185 0.917532533 1 IR

AT2G29600 chr2:12654983-12654988 before first 16::113 30::150 126::125 184::180 0.917577136 1 A5SS

AT1G56170 chr1:21024796-21025011 1 126::31 88::12 50::75 44::54 0.917710136 1 A5SS

AT3G28070 chr3:10449265-10449336 4 169::74 147::72 190::301 158::285 0.917743507 1 A3SS

AT3G11560 chr3:3645422-3645534 18 2::1107 15::1418 528::505 683::704 0.917837332 1 IR

AT2G03810 chr2:1161724-1161729 2 8::13 11::3 14::10 8::5 0.918035586 1 A5SS

AT4G04692 chr4:2375978-2376115 1 147::2852 103::2896 1580::1332 1589::1368 0.918044407 1 IR

AT3G21110 chr3:7405407-7405417 1 100::15 77::23 83::75 67::72 0.918072469 1 A5SS

AT1G15040 chr1:5180304-5180395 1 80::9 87::17 14::78 16::91 0.918087088 1 IR

AT5G16810 chr5:5529105-5529116 3 102::11 80::11 24::132 27::130 0.918105747 1 A3SS

AT3G18240 chr3:6257710-6257792 4 7::237 23::264 117::81 141::103 0.918209329 1 IR

AT2G41720 chr2:17403808-17403905 9 89::102 97::106 56::90 65::113 0.918321442 1 IR

AT5G58470 chr5:23638295-23638448 6 115::98 122::152 64::127 104::168 0.918345673 1 IR

AT4G37070 chr4:17465130-17465171 6 5::57 0::60 17::84 14::56 0.918584102 1 A5SS

AT1G09660 chr1:3128187-3128259 7 511::40 653::58 73::300 99::402 0.918624819 1 IR

AT5G05670 chr5:1698023-1698067 1 131::185 95::146 128::118 91::101 0.918655274 1 A5SS

AT5G20010 chr5:6760858-6760866 3 15::2846 4::2048 1821::2814 1265::1989 0.918679905 1 A5SS

AT5G66470 chr5:26543048-26543155 5 226::5 236::13 18::279 34::294 0.918689709 1 A3SS

AT2G04039 chr2:1333703-1333778 2 1183::55 1287::115 167::1410 219::1658 0.918760026 1 IR

AT1G18690 chr1:6433670-6433688 1 10::86 18::83 74::45 80::61 0.918834273 1 A5SS

AT1G80040 chr1:30109866-30110115 4 681::7 779::17 20::744 26::837 0.918874279 1 A3SS

AT2G43920 chr2:18190429-18190549 3 283::19 443::17 20::199 20::335 0.918922002 1 AltStart

AT3G12700 chr3:4037997-4038058 2 78::4 41::8 9::100 6::84 0.918971472 1 Cassette

AT3G59780 chr3:22086558-22086566 1 293::14 307::14 85::217 76::212 0.919052683 1 A5SS

AT4G32250 chr4:15573184-15573294 1 0::55 0::43 29::68 23::55 0.91905431 1 IR

AT4G38600 chr4:18041298-18041381 17 163::340 277::536 219::216 336::356 0.919203265 1 IR

AT4G32060 chr4:15502412-15502446 4-5 165::131 173::138 403::589 456::709 0.919503941 1 Cassette

AT1G13700 chr1:4695954-4696023 1 19::24 6::22 22::92 16::70 0.919545973 1 AltStart

AT5G65210 chr5:26058542-26058659 3 111::4 109::5 8::151 17::195 0.919589313 1 AltStart

AT5G59780 chr5:24083198-24083230 1 113::41 149::32 296::273 413::383 0.919652003 1 A5SS

AT5G56600 chr5:22910220-22910243 2 1318::14 1437::12 73::1351 85::1416 0.919664044 1 A3SS

AT4G23330 chr4:12193486-12193620 5 0::18 0::45 13::35 18::63 0.91968889 1 IR

AT2G30740 chr2:13095620-13095700 before first 64::10 45::9 17::135 13::174 0.919703731 1 AltStart

AT1G48540 chr1:17950226-17950262 11 79::3 159::2 85::74 166::127 0.919706016 1 A3SS

AT4G10170 chr4:6344452-6344587 1 12::87 9::71 45::84 34::95 0.919835363 1 IR

AT5G62720 chr5:25193925-25193953 3 360::17 317::31 402::130 352::141 0.919916257 1 A3SS

AT3G58220 chr3:21565081-21565272 3 35::43 67::86 28::60 47::100 0.919916819 1 IR

AT4G28025 chr4:13937258-13937285 1 966::87 1172::93 1103::327 1316::383 0.920004221 1 A5SS

AT3G05675 chr3:1659893-1660163 1 15::4 22::6 4::13 3::25 0.920117584 1 IR

AT2G18410 chr2:7992131-7992241 7 84::4 96::15 6::98 13::121 0.920270254 1 IR

AT2G41700 chr2:17384994-17385001 36 10::31 6::61 8::52 6::85 0.920303924 1 A5SS

AT1G07930 chr1:2459870-2460102 3 0::10342 0::10455 2111::2799 2436::3334 0.920335537 1 IR

AT3G44620 chr3:16194982-16195052 3 331::17 437::36 35::347 48::430 0.920379233 1 IR

AT2G22920 chr2:9757089-9757111 13 118::12 140::6 41::111 38::137 0.920380571 1 A5SS

AT4G22540 chr4:11864253-11864378 2 53::8 38::14 7::127 8::149 0.920381545 1 AltStart

AT5G14060 chr5:4536067-4536073 2 324::629 281::647 343::895 320::738 0.920630871 1 A3SS

AT4G36800 chr4:17342584-17342679 1 889::7 854::0 910::171 895::162 0.920698674 1 A5SS

AT3G63445 chr3:23425267-23425754 2 4::63 11::85 99::50 109::72 0.921034185 1 Cassette_multi

AT1G55310 chr1:20632477-20632551 7 0::255 0::1641 130::84 845::501 0.921050282 1 IR

AT5G64572 chr5:25810875-25810885 2 1::415 1::805 212::450 437::785 0.921272126 1 A3SS

AT1G44900 chr1:16970827-16970833 2 30::0 19::0 21::34 14::39 0.921274694 1 A3SS

AT4G01610 chr4:695594-695599 4 0::449 2::301 326::624 218::438 0.921291072 1 A5SS

AT1G49180 chr1:18184820-18184943 11 5::44 12::48 28::26 27::45 0.921318576 1 IR

AT2G26692 chr2:11350828-11350888 1 786::4 374::6 54::918 33::448 0.921422167 1 A5SS

AT5G05560 chr5:1647253-1647345 13-14 0::39 0::52 21::27 32::27 0.921532194 1 Cassette

AT5G66240 chr5:26467907-26467913 10 909::40 1112::52 827::828 1010::946 0.921593173 1 A3SS

AT5G64470 chr5:25777435-25777458 4 79::19 77::21 74::74 88::106 0.921698479 1 A3SS

AT1G45248 chr1:17164616-17164671 1 3::26 1::46 9::20 5::26 0.921810174 1 A5SS

AT5G52530 chr5:21317901-21318009 2 90::10 98::9 29::150 22::178 0.921885435 1 AltStart

AT1G31500 chr1:11274126-11274236 9 76::23 79::24 23::91 26::123 0.921897278 1 IR

AT3G08840 chr3:2682478-2682620 13 50::31 54::31 14::64 17::76 0.922037514 1 IR

AT1G51110 chr1:18936547-18936552 6 48::181 73::249 118::281 179::373 0.922040882 1 A3SS

AT5G56380 chr5:22840679-22840817 1 12::47 7::74 28::50 43::59 0.922047944 1 IR

AT5G54090 chr5:21951080-21951088 8 11::8 10::4 14::24 10::29 0.922081928 1 A3SS

AT3G14075 chr3:4666823-4666849 1 36::0 43::4 45::16 44::24 0.922271433 1 A5SS

AT2G03730 chr2:1139948-1140082 1 64::6 71::14 10::58 11::69 0.922324674 1 IR

AT1G25500 chr1:8957162-8957243 2 0::75 0::84 46::32 45::50 0.9224068 1 IR

AT3G01710 chr3:260537-260546 5 18::25 13::34 28::48 27::49 0.922517031 1 A3SS

AT5G50950 chr5:20732455-20732521 11 16::347 12::398 166::197 207::198 0.922591571 1 Cassette

AT5G18550 chr5:6162203-6162242 6 14::155 8::190 167::149 210::165 0.922643983 1 A5SS

AT1G26930 chr1:9336850-9337007 2 9::217 7::182 134::127 113::146 0.922771675 1 IR

AT1G32120 chr1:11554693-11554781 1 30::109 17::58 62::29 34::26 0.922983776 1 IR

AT1G54390 chr1:20306635-20306727 2 61::27 76::27 22::85 22::105 0.923012671 1 IR

AT5G58210 chr5:23552271-23552359 1 7::71 11::80 37::48 42::48 0.923066086 1 IR

AT1G11790 chr1:3984508-3984595 8 241::5 188::3 21::218 17::184 0.923091881 1 IR

AT5G39570 chr5:15844633-15844772 1 70::4045 26::3527 1940::2120 1750::1802 0.923110194 1 IR

AT4G30820 chr4:15007073-15007211 3 15::92 23::88 40::49 61::46 0.923181934 1 Cassette

AT3G27820 chr3:10316801-10316814 6 9::752 14::708 235::602 224::588 0.923232797 1 A3SS

AT5G24350 chr5:8311524-8311554 15 9::18 10::19 15::26 18::34 0.92324154 1 A3SS

AT5G18190 chr5:6013783-6013862 2 57::14 53::1 31::133 19::113 0.923387514 1 A3SS

AT5G11200 chr5:3568142-3568278 4 40::528 32::509 49::527 62::526 0.92339171 1 A3SS

AT3G26700 chr3:9809950-9810425 1 24::10 19::15 14::49 15::73 0.923517465 1 AltStart

AT2G43410 chr2:18025922-18026259 7 80::10 109::3 11::97 18::113 0.92357042 1 AltEnd

AT1G08360 chr1:2636053-2636109 1 995::16 825::7 47::178 33::142 0.92357769 1 A5SS

AT5G59780 chr5:24083118-24083198 1 112::420 147::600 245::321 329::455 0.923697144 1 IR

AT2G25910 chr2:11050529-11050594 5-6 442::10 489::17 10::432 26::486 0.923883591 1 Cassette

AT5G53470 chr5:21711654-21711659 3 151::4 221::16 97::153 153::217 0.923895904 1 A5SS

AT5G28400 chr5:10344245-10344325 8-9 0::18 0::31 13::7 18::9 0.923969902 1 AltStart

AT3G54500 chr3:20177349-20177408 6 9::1092 13::1639 144::1558 181::2145 0.924096211 1 A5SS

AT5G13480 chr5:4331531-4331628 1 19::48 22::69 26::19 34::29 0.924390411 1 IR

AT1G02910 chr1:656745-656755 5 298::38 257::55 61::343 77::376 0.924393227 1 A3SS

AT5G13360 chr5:4284342-4284347 3 78::6 81::9 38::75 40::83 0.924503809 1 A3SS

AT1G11450 chr1:3853820-3853995 2 46::15 41::14 7::59 10::63 0.924507497 1 IR

AT1G04080 chr1:1053096-1053187 5 219::46 229::35 31::280 35::284 0.924618372 1 Cassette

AT5G57350 chr5:23236388-23236394 2 49::105 29::70 191::337 132::236 0.925028281 1 A3SS

AT4G04620 chr4:2329943-2329974 2 77::9 67::1 90::207 101::230 0.925061704 1 A3SS

AT1G13860 chr1:4743530-4743669 9 22::4 42::5 5::31 7::47 0.925167962 1 IR

AT1G08680 chr1:2765200-2765327 6 10::217 0::208 127::116 131::108 0.925275756 1 IR

AT3G17611 chr3:6025161-6025385 1 59::5 80::11 5::67 10::96 0.925318771 1 IR

AT1G62250 chr1:22995919-22996058 5 379::25 460::41 34::440 40::501 0.925415027 1 IR

AT2G30695 chr2:13081131-13081296 1 198::26 161::32 26::156 26::128 0.925458839 1 IR

AT5G35680 chr5:13858594-13858623 2 531::78 527::86 148::578 174::743 0.925474946 1 A3SS

AT1G21130 chr1:7399469-7399558 2 342::43 288::19 37::291 24::247 0.925823661 1 IR

AT3G57410 chr3:21244719-21244725 22 11::383 7::434 450::475 505::591 0.92590349 1 A3SS

AT3G59470 chr3:21980132-21980528 1 29::9 27::24 16::59 24::60 0.925904977 1 IR

AT4G31860 chr4:15407034-15407120 9 244::14 227::22 29::232 30::232 0.925935004 1 IR

AT1G49130 chr1:18175798-18175903 1 2::42 1::23 23::35 12::32 0.925998594 1 IR

AT5G44530 chr5:17940778-17940913 10 97::9 113::14 177::102 238::179 0.926018946 1 A3SS

AT1G68580 chr1:25755241-25755331 7 13::368 8::474 185::146 266::200 0.926029416 1 IR

AT1G71080 chr1:26810456-26810465 4 346::7 414::11 154::316 186::392 0.926078841 1 A3SS

AT1G07120 chr1:2186044-2186103 1 2::33 7::37 9::40 9::34 0.926229246 1 A5SS

AT1G03545 chr1:886244-886393 1 42::12 75::26 7::28 14::38 0.926288238 1 IR

AT4G16700 chr4:9397133-9397140 6 9::11 9::15 8::29 8::29 0.926422989 1 A5SS

AT1G54440 chr1:20327614-20327620 14 9::48 12::57 31::50 52::93 0.926431536 1 A3SS

AT3G15400 chr3:5201829-5201970 1 0::57 0::62 27::29 35::30 0.926507925 1 IR

AT2G31800 chr2:13521821-13521828 8 79::45 101::50 83::113 105::157 0.926510806 1 A3SS

AT3G53190 chr3:19714310-19714372 1 416::8 326::8 35::217 30::171 0.926601984 1 A5SS

AT2G39725 chr2:16569515-16569583 1 23::3 33::14 7::9 21::12 0.926851764 1 A5SS

AT1G24030 chr1:8504087-8504106 3 148::10 219::10 33::153 40::213 0.926879961 1 A5SS

AT5G51970 chr5:21111658-21111742 1 11::89 7::124 22::264 37::297 0.927130728 1 IR

AT1G05410 chr1:1587124-1587206 2 4::219 4::164 123::100 87::90 0.927610767 1 IR

AT1G13340 chr1:4570340-4570350 2 5::33 11::44 11::55 14::58 0.927927372 1 A5SS

AT4G29020 chr4:14305287-14305308 1 40::17 231::2 6232::4005 5041::2918 0.927978697 1 A5SS

AT3G20890 chr3:7320349-7320445 2 5::166 11::157 89::99 81::117 0.927992458 1 IR

AT2G44830 chr2:18490129-18490153 1 69::2 48::2 4::116 6::117 0.928013799 1 AltStart

AT1G15175 chr1:5223733-5223841 1-2 59::76 56::46 72::53 44::42 0.928126141 1 Cassette

AT5G66675 chr5:26616686-26616813 1 14::125 9::109 64::98 53::109 0.928166951 1 IR

AT2G21150 chr2:9066873-9066886 6 18::150 23::124 41::231 52::214 0.928226923 1 A3SS

AT3G48110 chr3:17767860-17767902 19 4::104 17::151 107::97 141::114 0.928260686 1 A5SS

AT5G07910 chr5:2522407-2522415 7 127::19 102::19 85::138 77::137 0.928279421 1 A3SS

AT1G52500 chr1:19560692-19561796 2 22::654 17::969 198::311 279::439 0.928445431 1 Cassette_multi

AT3G59910 chr3:22132928-22133021 2 10::550 18::437 276::305 235::266 0.928524277 1 IR

AT1G03160 chr1:762578-762628 3 47::7 68::12 20::89 26::91 0.928548705 1 A5SS

AT3G01480 chr3:189298-189311 2 1010::8 1150::7 239::1069 282::1119 0.928615988 1 A5SS

AT5G13500 chr5:4338312-4338361 1 0::81 9::81 73::15 79::26 0.928861195 1 A5SS

AT5G65430 chr5:26148566-26148687 3 1742::10 2306::19 112::931 152::1224 0.928875024 1 IR

AT1G72360 chr1:27242090-27242176 1 48::8 77::7 7::55 10::89 0.928954761 1 IR

AT5G58300 chr5:23572341-23572427 2 36::4 28::2 5::158 3::149 0.928971095 1 AltStart

AT1G18470 chr1:6356935-6357080 11 263::6 225::9 20::235 16::219 0.928975528 1 IR

AT1G73470 chr1:27625294-27625407 1 117::37 120::34 28::116 23::104 0.929051966 1 IR

AT4G38540 chr4:18023624-18023640 3 104::11 103::23 28::134 52::143 0.929200164 1 A5SS

AT1G28090 chr1:9797975-9798034 11 4::158 17::221 82::88 109::128 0.929272794 1 Cassette

AT3G16760 chr3:5704311-5704369 1 0::351 0::395 187::144 210::158 0.929389319 1 IR

AT3G11930 chr3:3777224-3777303 3 2283::72 2672::114 131::1182 167::1357 0.929523443 1 IR

AT1G25520 chr1:8964280-8964285 13 7::136 20::238 15::39 26::63 0.929525921 1 A3SS

AT1G53530 chr1:19978383-19978393 1 153::6 158::26 46::76 61::97 0.92990058 1 A5SS

AT5G48880 chr5:19816923-19817204 2 43::71 24::52 38::61 49::43 0.929940466 1 Cassette

AT3G20270 chr3:7067494-7067544 2 25::18 15::24 11::55 19::79 0.9299699 1 Cassette

AT4G18975 chr4:10392714-10392726 7 126::7 152::18 56::123 85::192 0.929975967 1 A3SS

AT5G54570 chr5:22168146-22168167 10 2::11 1::17 5::16 7::38 0.93001399 1 A5SS

AT3G04440 chr3:1180320-1180340 3 21::13 6::12 3::3 3::3 0.930049492 1 A3SS

AT3G17120 chr3:5841824-5842067 2 141::13 130::5 7::183 5::202 0.930220259 1 Cassette

AT4G04740 chr4:2404968-2405095 8 0::34 0::87 33::24 42::44 0.930227241 1 IR

AT3G17330 chr3:5919389-5919517 2 3::95 3::69 54::74 38::51 0.93033572 1 Cassette

AT1G13630 chr1:4670051-4670135 3-4 0::13 0::43 10::17 23::17 0.93037892 1 Cassette

AT2G26890 chr2:11471967-11471977 5 22::14 12::3 22::45 9::24 0.93041759 1 A3SS

AT5G25570 chr5:8902897-8902986 4 50::8 50::7 10::39 8::42 0.930419117 1 IR

AT3G54500 chr3:20175665-20175782 9 1171::203 1895::266 148::1095 205::1792 0.930441935 1 IR

AT3G25805 chr3:9427131-9427136 3 106::27 119::31 73::145 90::171 0.930542383 1 A3SS

AT3G17910 chr3:6134718-6134724 3 68::8 94::13 20::73 30::85 0.930593399 1 A5SS

AT5G36170 chr5:14236387-14236406 7 384::10 533::35 102::225 160::361 0.930599377 1 A3SS

AT2G33800 chr2:14301169-14301174 2 8::4199 10::4211 2590::2657 2627::2886 0.930736849 1 A3SS

AT3G28130 chr3:10465780-10465881 1 81::8 90::24 13::110 18::102 0.930862504 1 IR

AT1G27921 chr1:9729134-9729366 5 11::11 6::12 13::26 37::52 0.930885278 1 A3SS

AT5G19221 chr5:6464234-6464266 1 1081::3 1443::9 24::699 34::776 0.931005258 1 AltStart

AT4G00420 chr4:184052-184132 1-2 86::12 89::15 13::121 23::158 0.931018982 1 AltStart

AT1G62610 chr1:23182355-23182361 3 15::47 10::80 61::92 96::129 0.93102937 1 A3SS

AT4G25570 chr4:13054202-13054213 4 58::1738 46::1744 1554::1509 1595::1561 0.931092773 1 A3SS

AT3G14110 chr3:4677001-4677016 3 1329::15 1267::9 127::1212 123::1295 0.931213424 1 A3SS

AT3G01050 chr3:14553-14620 4 20::226 21::327 50::256 59::324 0.931213828 1 A3SS

AT4G12560 chr4:7443193-7443524 2 149::70 159::92 63::114 96::142 0.931214549 1 IR

AT5G05460 chr5:1618798-1618893 11 2::17 13::22 9::54 15::79 0.931260264 1 IR

AT3G24080 chr3:8695280-8695305 1 0::92 0::204 59::48 128::69 0.93132208 1 IR

AT1G15400 chr1:5296046-5296051 2 8::74 4::118 91::44 127::55 0.931341734 1 A3SS

AT4G05040 chr4:2578712-2578937 1 29::1 23::6 4::11 5::19 0.931459794 1 A5SS

AT1G17410 chr1:5969479-5969487 2 23::21 59::28 35::93 47::136 0.931478008 1 A3SS

AT5G15790 chr5:5148844-5148950 1 1::73 5::79 28::33 39::37 0.931485281 1 IR

AT3G61440 chr3:22736869-22736949 5 2976::12 4587::7 2900::3466 4658::5814 0.931523756 1 A3SS

AT2G36360 chr2:15242921-15243022 17 22::81 17::113 44::74 49::111 0.9316916 1 IR

AT1G52230 chr1:19455329-19455402 3 16059::59 15253::0 20646::12368 20720::13927 0.9317834 1 A3SS

AT1G32810 chr1:11884737-11884745 5 64::5 65::15 49::103 55::122 0.931818212 1 A3SS

AT1G17890 chr1:6155442-6155470 2 106::5 102::16 28::227 46::272 0.93186837 1 A3SS

AT1G04290 chr1:1148072-1148091 2 18::453 10::447 434::395 452::458 0.931888583 1 A3SS

AT3G59770 chr3:22084967-22085071 3 74::2 67::1 68::88 64::64 0.932029675 1 A5SS

AT5G14930 chr5:4829868-4830159 3 0::48 0::86 26::22 35::32 0.932112663 1 IR

AT5G03900 chr5:1050993-1051226 11 243::22 210::36 17::250 25::247 0.932172965 1 IR

AT1G13350 chr1:4575915-4575995 2 0::28 3::38 21::50 29::66 0.932215734 1 Cassette

AT1G78780 chr1:29622303-29622447 2 1::12 0::35 17::39 37::50 0.932366123 1 AltStart

AT1G30282 chr1:10663994-10664089 2 8::3 21::15 3::21 9::24 0.932493723 1 IR

AT2G30120 chr2:12860541-12860678 4 10::0 33::0 8::46 21::64 0.932511591 1 AltEnd

AT3G24150 chr3:8725625-8725750 1 8::80 15::99 36::47 51::62 0.932649173 1 IR

AT1G04910 chr1:1390426-1390451 3 23::38 32::43 36::106 43::73 0.932751579 1 A5SS

AT3G58030 chr3:21484599-21484775 1 34::110 16::97 64::80 47::79 0.93281845 1 IR

AT1G33415 chr1:12119473-12119521 2 25::2 23::10 31::30 25::40 0.933020781 1 A3SS

AT3G13060 chr3:4183207-4183294 7 255::132 275::194 85::314 117::349 0.933109563 1 IR

AT1G17970 chr1:6185324-6185483 1 53::132 35::154 52::124 65::122 0.933212104 1 IR

AT5G63460 chr5:25414013-25414326 4 0::547 0::676 240::283 305::350 0.933263007 1 Cassette_multi

AT3G52110 chr3:19324169-19324195 6 71::75 105::83 132::183 133::186 0.933285175 1 A5SS

AT2G15080 chr2:6536719-6536823 1 7::3 26::2 4::15 3::22 0.933308378 1 IR

AT1G15180 chr1:5225788-5225882 5 9::700 22::747 377::456 375::446 0.93331454 1 IR

AT4G28250 chr4:14000752-14000822 3-4 2541::34 2534::23 81::2137 89::2280 0.933416176 1 Cassette

AT5G01215 chr5:85261-85357 1 5::464 12::528 249::312 274::351 0.933427459 1 IR

AT1G17110 chr1:5848200-5848212 6 26::46 36::52 44::102 50::111 0.933449843 1 A5SS

AT1G32070 chr1:11535444-11535458 5 5::800 16::1022 268::814 354::934 0.933647553 1 A5SS

AT4G35440 chr4:16837816-16837821 3 91::28 88::47 127::117 145::133 0.93374909 1 A5SS

AT1G61150 chr1:22543266-22543368 4 187::11 150::19 17::214 20::188 0.933751876 1 IR

AT3G54000 chr3:19997805-19997895 3 48::19 75::17 14::63 15::79 0.933774648 1 IR

AT2G18915 chr2:8197011-8197021 1 83::0 57::0 62::100 46::69 0.933793039 1 A5SS

AT3G29644 chr3:11500667-11500771 2 59::116 17::24 47::124 13::62 0.933941048 1 Cassette

AT3G10915 chr3:3417171-3417206 2 148::0 145::0 37::112 53::128 0.934066444 1 Cassette_multi

AT5G05310 chr5:1571875-1571882 4 14::32 23::33 12::42 18::64 0.934104033 1 A3SS

AT5G45370 chr5:18389359-18389432 4 0::74 0::82 45::26 52::43 0.934145076 1 IR

AT2G47850 chr2:19595826-19595950 1 9::108 0::81 45::51 39::43 0.934147365 1 IR

AT4G38360 chr4:17969108-17969113 7 8::170 9::142 42::198 38::197 0.934257614 1 A3SS

AT3G54740 chr3:20262845-20263037 2 24::55 26::49 38::80 30::108 0.934339502 1 IR

AT3G20270 chr3:7067116-7067772 1 25::11 15::25 7::55 7::79 0.934568095 1 IR

AT1G02145 chr1:407468-407473 16 110::19 89::29 68::194 59::192 0.934581112 1 A3SS

AT1G08520 chr1:2697872-2697877 5 549::26 351::24 543::603 349::453 0.934639154 1 A3SS

AT2G03390 chr2:1032225-1032381 2 20::198 18::194 245::331 187::271 0.934694317 1 A3SS

AT1G66270 chr1:24701881-24701887 6 0::672 1::831 323::660 319::770 0.934712614 1 A3SS

AT2G34410 chr2:14522883-14523057 17 53::78 16::91 189511::163573 196152::188165 0.934722024 1 A5SS

AT5G07360 chr5:2328145-2328166 8 0::117 8::150 127::117 153::151 0.93484748 1 A3SS

AT1G02350 chr1:469971-470009 1 4::52 8::38 76::79 75::81 0.934863339 1 A5SS

AT5G46490 chr5:18851966-18852374 2 4::243 8::264 133::124 153::105 0.934880485 1 IR

AT1G68660 chr1:25778138-25778229 3 85::3759 82::4263 1998::638 2281::700 0.934941615 1 IR

AT3G49390 chr3:18317725-18317744 2 98::0 55::5 37::187 23::165 0.934981792 1 A3SS

AT1G30500 chr1:10805141-10805153 5 17::364 12::450 277::371 349::452 0.934995631 1 A3SS

AT5G47900 chr5:19393606-19393615 8 36::6 47::5 63::70 64::67 0.935008593 1 A3SS

AT4G17890 chr4:9938967-9939039 6 304::18 411::47 40::156 66::206 0.935033836 1 IR

AT3G63445 chr3:23424663-23424743 2-3 15::11 36::19 72::66 129::86 0.935040389 1 AltEnd

AT2G31280 chr2:13340896-13341015 7 0::88 0::144 60::95 84::129 0.935087354 1 Cassette

AT2G14835 chr2:6366974-6367175 1 99::32 101::28 17::110 18::113 0.935122305 1 IR

AT5G64130 chr5:25665029-25665105 3 2680::6 2938::4 107::2706 117::2955 0.935212292 1 IR

AT3G12020 chr3:3832971-3833209 21 33::0 30::2 10::43 4::43 0.935284648 1 IR

AT5G59440 chr5:23971809-23971816 3 95::106 157::156 53::328 85::383 0.935337057 1 A3SS

AT1G19650 chr1:6798953-6799099 2 20::14 23::8 6::31 7::31 0.935463179 1 IR

AT2G36360 chr2:15244477-15244498 13 94::3 89::8 25::114 32::93 0.935535452 1 A3SS

AT3G45090 chr3:16493806-16493811 3 2::16 1::38 11::33 16::38 0.935555388 1 A3SS

AT5G01470 chr5:191465-191523 3 170::13 109::2 194::189 125::132 0.935627459 1 A3SS

AT4G15233 chr4:8694037-8694109 22 16::0 35::0 3::9 3::26 0.93568109 1 A5SS

AT1G55350 chr1:20654235-20654256 32 25::162 78::285 169::182 282::274 0.93569966 1 A3SS

AT4G17098 chr4:9610400-9610502 2 214::59 257::84 43::168 57::216 0.935763706 1 IR

AT2G38020 chr2:15910247-15910350 15 180::10 254::18 10::132 16::188 0.93582034 1 IR

AT5G58005 chr5:23482775-23482963 3 243::34 371::27 18::243 26::270 0.935832436 1 IR

AT2G26980 chr2:11518401-11518505 2 0::15 0::29 18::148 20::191 0.935880637 1 AltStart

AT1G05785 chr1:1730922-1730959 2 18::52 14::57 24::73 19::75 0.935947036 1 A3SS

AT1G27370 chr1:9507023-9507051 3 22::1 27::7 32::65 93::127 0.935978665 1 A3SS

AT1G75300 chr1:28255947-28255966 2 1::26 0::23 41::50 51::68 0.936007239 1 A5SS

AT3G27190 chr3:10040353-10040367 10 19::167 26::254 56::168 77::239 0.936012524 1 A5SS

AT4G20260 chr4:10941548-10941567 2 771::3 589::5 722::1031 573::835 0.936017905 1 A3SS

AT4G34750 chr4:16578052-16578178 1 5::63 36::121 30::62 58::127 0.936144728 1 IR

AT5G10030 chr5:3140008-3140252 2 0::34 0::28 28::64 31::109 0.936265199 1 AltStart

AT2G34860 chr2:14709491-14709512 4 1676::39 1859::29 1442::1536 1649::1834 0.936307339 1 A3SS

AT2G01470 chr2:213546-213551 4 193::33 206::57 108::245 128::285 0.936332535 1 A3SS

AT5G38590 chr5:15452551-15452628 2 19::4 32::17 5::45 10::53 0.936333918 1 IR

AT4G15510 chr4:8861752-8862314 3 522::4 565::19 13::392 15::386 0.936363089 1 IR

AT3G43790 chr3:15658632-15658650 16 26::0 26::0 8::36 7::26 0.936643177 1 A5SS

AT3G47680 chr3:17577398-17578461 2 56::22 65::22 60::75 110::124 0.936773564 1 AltEnd

AT4G23980 chr4:12453720-12453726 12 72::2 93::0 63::113 91::127 0.936855425 1 A3SS

AT2G37340 chr2:15671600-15671818 3 1600::57 1781::88 113::1493 147::1799 0.936871787 1 A3SS

AT4G19985 chr4:10831166-10831196 2 6::204 17::275 54::192 87::245 0.936890725 1 A5SS

AT2G41600 chr2:17345760-17345768 3 3::188 0::283 141::169 229::249 0.936891443 1 A3SS

AT3G48780 chr3:18091519-18091601 9 277::44 323::66 64::346 88::415 0.937057597 1 A5SS

AT5G04000 chr5:1079621-1079627 2 20::0 15::4 18::26 15::19 0.937191219 1 A3SS

AT2G44800 chr2:18468014-18468100 3 20::11 20::17 7::19 9::28 0.93722067 1 IR

AT3G44510 chr3:16110615-16110627 2 9::9 26::29 17::56 31::72 0.937528254 1 A3SS

AT3G57050 chr3:21114162-21114207 2 428::80 342::77 454::633 400::567 0.937555504 1 A5SS

AT5G28770 chr5:10796920-10796988 5 355::102 355::107 85::367 88::415 0.937626526 1 IR

AT2G45770 chr2:18852407-18852423 6 306::11 217::15 310::363 230::284 0.937629378 1 A3SS

AT5G47570 chr5:19294185-19294194 3 2333::7 2451::8 2109::2478 2262::2636 0.937631451 1 A5SS

AT1G53430 chr1:19937688-19937712 12 24::0 29::0 27::29 28::26 0.937693144 1 A3SS

AT3G17300 chr3:5907449-5907476 1-2 266::11 355::21 13::251 28::308 0.937709792 1 Cassette

AT1G29690 chr1:10380433-10380443 4 9::47 8::43 37::72 37::75 0.937799952 1 A3SS

AT3G11945 chr3:3782674-3782717 1 250::0 196::0 238::271 205::179 0.937801091 1 A5SS

AT3G54010 chr3:20001313-20001384 2 81::6 45::10 11::71 10::47 0.937815777 1 IR

AT3G59765 chr3:22077755-22077765 2 21::244 7::125 253::232 135::136 0.937930516 1 A3SS

AT5G41150 chr5:16474121-16474384 5 19::6 26::4 4::27 4::42 0.937940163 1 IR

AT5G44240 chr5:17817535-17817625 2 24::0 36::0 27::37 34::35 0.937943405 1 A3SS

AT5G64170 chr5:25674007-25674123 5 10::885 11::1119 461::472 583::524 0.938019483 1 IR

AT2G15000 chr2:6481358-6481656 3 566::15 741::33 8::353 24::519 0.938132222 1 Cassette

AT2G01180 chr2:108114-108505 1 54::5 43::11 11::102 16::92 0.938165935 1 IR

AT3G62090 chr3:22989726-22989791 4 8::8 5::21 9::16 13::23 0.938269386 1 Cassette

AT1G21690 chr1:7616604-7616625 4 214::0 205::0 186::219 180::174 0.938354387 1 A3SS

AT1G05890 chr1:1779786-1779869 1 221::0 230::0 33::146 52::150 0.938397655 1 A5SS

AT3G24560 chr3:8965366-8965451 7 15::1 39::3 3::20 3::38 0.938405895 1 IR

AT3G18160 chr3:6221244-6221336 2 82::1 87::13 9::83 14::105 0.938557832 1 IR

AT5G65080 chr5:26000267-26000324 2 0::88 0::22 37::30 12::16 0.938574165 1 Cassette

AT3G46100 chr3:16930107-16930155 4 169::7 146::11 24::206 35::186 0.938589606 1 A3SS

AT5G40370 chr5:16148769-16148997 1 2835::9 2668::1 96::1657 87::1497 0.938652862 1 IR

AT5G44710 chr5:18041594-18041603 2 208::9 305::6 213::283 288::407 0.938660452 1 A3SS

AT1G17350 chr1:5944233-5944289 8 0::179 0::198 102::98 110::117 0.938681368 1 IR

AT3G09770 chr3:2996557-2996644 2 388::19 427::16 44::361 43::328 0.938682252 1 IR

AT2G42600 chr2:17738711-17738809 2 26::202 9::130 513::977 357::668 0.938688904 1 A3SS

AT4G24840 chr4:12799534-12799539 3 102::11 84::16 38::128 37::113 0.938706866 1 A3SS

AT5G03180 chr5:755162-755174 3 30::10 43::14 35::40 57::52 0.938738103 1 A5SS

AT4G01430 chr4:586158-586509 2 0::22 0::43 26::18 40::30 0.938872965 1 Cassette_multi

AT4G38950 chr4:18157248-18157253 8 6::120 14::137 72::145 83::149 0.938927811 1 A3SS

AT5G66010 chr5:26400003-26400080 2 16::72 28::104 121::202 145::293 0.938932208 1 A3SS

AT3G53270 chr3:19750629-19750635 10 3::92 1::129 105::65 138::89 0.93893407 1 A3SS

AT5G45300 chr5:18355618-18355629 6 3::64 18::88 51::76 76::94 0.938981335 1 A5SS

AT1G75630 chr1:28400950-28401056 2 14::5375 5::5305 2789::2834 2774::2939 0.939028882 1 IR

AT4G03205 chr4:1414125-1414210 3 60::13 102::24 12::45 18::81 0.939044933 1 IR

AT4G16540 chr4:9316390-9316413 3-4 0::27 0::47 35::63 51::112 0.939177657 1 Cassette

AT5G59660 chr5:24038544-24038619 13 16::0 21::0 20::21 23::22 0.939220742 1 A3SS

AT5G63050 chr5:25293781-25293866 4 10::536 10::511 270::358 251::362 0.939261305 1 IR

AT2G35620 chr2:14964749-14964772 2 27::31 14::25 36::71 34::77 0.939414885 1 A3SS

AT3G03690 chr3:913146-913151 1 82::33 50::19 70::75 46::68 0.939469285 1 A5SS

AT4G35750 chr4:16941254-16941339 2 12::6876 5::7258 3830::3157 4043::3330 0.939499025 1 IR

AT5G47110 chr5:19134832-19134854 1 11::3703 15::3702 353::4158 366::3969 0.939528368 1 A5SS

AT5G28530 chr5:10525656-10525668 3 22::0 26::1 9::29 11::48 0.939542534 1 A3SS

AT1G55325 chr1:20644144-20644160 21 65::0 54::0 59::56 49::56 0.939565586 1 A3SS

AT2G01220 chr2:124269-124296 6 199::27 363::49 56::203 100::331 0.939569069 1 A5SS

AT1G01490 chr1:182135-182253 1 90::339 68::238 169::272 117::225 0.939618745 1 IR

AT1G52000 chr1:19334943-19335027 3 140::46 47::1 1157::1293 393::458 0.939714873 1 A3SS

AT3G01790 chr3:285593-285624 2 428::22 504::34 93::521 114::658 0.93976151 1 A3SS

AT4G10550 chr4:6520138-6520215 1 0::12 0::50 8::6 30::20 0.939855465 1 IR

AT4G12720 chr4:7487449-7487541 1 30::107 18::109 53::48 52::47 0.939884521 1 IR

AT2G48100 chr2:19671176-19671183 2 162::12 148::9 128::221 131::263 0.93997579 1 A3SS

AT3G29100 chr3:11076506-11076606 4 38::3 32::6 7::42 8::31 0.939997646 1 IR

AT5G23420 chr5:7888959-7889533 4 0::300 0::299 163::149 147::151 0.940048763 1 Cassette_multi

AT2G28940 chr2:12427952-12427959 2 14::8 21::5 12::44 27::71 0.940143959 1 A3SS

AT3G10130 chr3:3132912-3132918 1 4::145 11::111 54::180 48::148 0.940189354 1 A5SS

AT4G24900 chr4:12815702-12815812 3 16::14 28::32 11::56 17::53 0.940205938 1 IR

AT2G46572 chr2:19127918-19127956 2 9::6 14::9 11::19 19::37 0.940222717 1 A5SS

AT3G05520 chr3:1601130-1601212 10 0::259 0::375 132::96 194::112 0.940276967 1 IR

AT1G79650 chr1:29974309-29974327 4 76::154 56::133 163::248 136::208 0.940291526 1 A3SS

AT3G23590 chr3:8470601-8470670 8 18::19 23::22 45::84 44::120 0.940307397 1 A3SS

AT5G10030 chr5:3139305-3139338 3 37::0 29::0 7::56 3::74 0.94036927 1 A3SS

AT5G40580 chr5:16250624-16250756 2 250::6 285::2 12::370 4::412 0.940377539 1 AltStart

AT5G62165 chr5:24964959-24965050 1 9::7 5::9 5::14 8::11 0.940458942 1 IR

AT2G29210 chr2:12560456-12560487 9 11::399 18::484 198::183 256::251 0.940471236 1 IR

AT2G20790 chr2:8950526-8950601 5 216::35 215::26 38::180 33::190 0.940516104 1 IR

AT4G03120 chr4:1387140-1387180 5 9::517 24::565 292::143 312::159 0.940571574 1 IR

AT1G30590 chr1:10838450-10838457 7 7::105 15::167 101::116 153::190 0.940644571 1 A3SS

AT1G33050 chr1:11968675-11968744 4 5::133 11::153 165::208 250::292 0.940684173 1 A3SS

AT2G43490 chr2:18054724-18054731 12 4::23 7::34 21::17 29::26 0.94088955 1 A3SS

AT5G35995 chr5:14138245-14138479 1 18::21 20::38 11::51 15::67 0.940917298 1 IR

AT3G56410 chr3:20916596-20919495 3 9::13 10::26 10::20 13::45 0.94103676 1 IR

AT5G16450 chr5:5374334-5374344 2 56::95 68::97 106::248 122::321 0.941141643 1 A3SS

AT4G02850 chr4:1267862-1267942 1-2 36::22 32::17 13::100 14::109 0.941222942 1 AltStart

AT1G71340 chr1:26887582-26887679 1 13::1374 2::1186 825::359 714::282 0.941342948 1 IR

AT1G03960 chr1:1014237-1014334 1 18::18 9::9 12::20 6::17 0.941448596 1 IR

AT5G24320 chr5:8286305-8286346 3 37::45 24::47 48::77 57::106 0.941804994 1 A3SS

AT5G45410 chr5:18403512-18403599 2 611::1 453::3 16::577 21::445 0.941848275 1 Cassette

AT2G31810 chr2:13524596-13524661 2 331::0 352::0 289::277 315::305 0.941880083 1 A3SS

AT2G46570 chr2:19127377-19127828 3 6::14 9::35 15::21 23::41 0.941904365 1 Cassette_multi

AT2G44650 chr2:18420128-18420182 3 31::3680 12::4132 1560::1703 1814::1985 0.941933714 1 Cassette

AT3G25013 chr3:9115462-9115527 6 22::0 11::0 23::15 17::16 0.94198247 1 A3SS

AT5G10350 chr5:3257636-3257642 1 512::29 754::41 249::317 373::491 0.942046126 1 A5SS

AT3G08890 chr3:2707405-2707416 3 151::10 130::20 127::121 112::126 0.942146682 1 A3SS

AT5G10900 chr5:3436336-3436419 9 24::5 36::1 6::29 4::34 0.942151905 1 IR

AT5G41150 chr5:16475556-16475568 3 9::3 15::20 9::21 16::36 0.94218061 1 A5SS

AT3G07750 chr3:2473478-2473484 2 97::40 108::28 58::136 71::177 0.942383614 1 A3SS

AT5G36790 chr5:14481190-14481225 11 13::52 12::46 10::17 10::18 0.942626071 1 A5SS

AT2G43910 chr2:18186352-18186540 2 45::4214 27::4343 1550::2006 1672::2047 0.942626694 1 Cassette

AT5G49970 chr5:20330719-20330752 6 344::0 295::0 379::395 307::380 0.942669911 1 A3SS

AT3G20320 chr3:7089701-7089807 1 96::10 103::11 12::233 16::232 0.942720524 1 IR

AT4G17410 chr4:9716189-9716350 1 32::56 21::41 36::109 27::115 0.942723704 1 IR

AT1G29950 chr1:10492590-10492724 3 240::12 236::11 10::252 9::243 0.942756496 1 IR

AT4G24740 chr4:12755932-12756193 5 26::436 58::600 235::291 330::406 0.942812129 1 Cassette_multi

AT4G17310 chr4:9686444-9686498 2 13::94 21::85 42::142 48::165 0.942943442 1 A5SS

AT1G24265 chr1:8600512-8600584 1 13::9 21::16 7::21 13::30 0.942957483 1 IR

AT5G53420 chr5:21674389-21674489 2 127::0 165::2 12::181 41::279 0.942993118 1 AltStart

AT1G54310 chr1:20274535-20274561 4 86::33 107::67 45::96 82::116 0.94302474 1 A5SS

AT5G35210 chr5:13474507-13474592 16 93::142 168::177 81::119 106::197 0.943145051 1 IR

AT1G17050 chr1:5830259-5830274 4 8::500 13::438 78::532 77::467 0.943165038 1 A3SS

AT1G60940 chr1:22441934-22442108 1 72::140 67::114 57::142 38::134 0.943274959 1 IR

AT1G31550 chr1:11295991-11296000 4 14::34 32::50 33::60 56::75 0.943338492 1 A5SS

AT5G53580 chr5:21765372-21765400 7 2::711 14::828 657::332 768::399 0.943418971 1 A3SS

AT5G64220 chr5:25686258-25686395 1 0::26 0::30 13::16 22::18 0.943460757 1 IR

AT1G52500 chr1:19562052-19562781 8 553::75 823::102 94::582 149::831 0.943513531 1 Cassette_multi

AT2G20190 chr2:8715187-8715192 14 87::8 115::14 50::191 74::203 0.943533895 1 A3SS

AT3G63440 chr3:23424157-23425170 1 15::43 36::37 57::96 77::115 0.943564044 1 AltStart

AT1G80040 chr1:30110697-30110725 2 472::4 469::2 54::545 41::540 0.943588776 1 Cassette

AT2G44800 chr2:18467230-18467255 1 37::0 33::0 9::23 14::26 0.943691045 1 A5SS

AT1G07320 chr1:2250173-2250179 2 3248::112 4038::109 1831::1049 2211::1296 0.943702594 1 A3SS

AT5G59830 chr5:24105305-24105358 2 32::19 31::16 54::72 52::67 0.943785313 1 A3SS

AT5G43500 chr5:17470021-17470077 3 3::33 0::48 8::34 5::54 0.943884706 1 A3SS

AT4G35500 chr4:16858110-16858139 3 12::0 25::0 19::34 31::43 0.944058388 1 A3SS

AT1G60200 chr1:22203749-22204553 2 27::17 26::22 21::88 31::78 0.944063492 1 A3SS

AT2G33820 chr2:14306885-14306892 6 6::45 16::63 42::64 67::91 0.944093979 1 A3SS

AT5G11330 chr5:3617450-3617522 4 2::585 2::905 305::219 475::309 0.944157053 1 IR

AT2G34460 chr2:14530145-14530236 3 15::2836 10::2446 1581::1727 1395::1625 0.944200348 1 IR

AT5G06250 chr5:1892735-1892766 2 11::4 19::11 15::23 21::36 0.944219532 1 A5SS

AT5G64200 chr5:25681542-25681733 8 20::67 180::434 27::34 136::246 0.944255852 1 IR

AT3G47540 chr3:17521977-17522241 2 0::186 0::193 90::116 87::92 0.944398993 1 IR

AT2G39340 chr2:16425150-16425246 3 19::65 40::80 37::66 45::85 0.944468035 1 IR

AT3G47550 chr3:17524894-17524965 6 246::41 267::43 34::243 42::269 0.944573798 1 IR

AT2G40360 chr2:16856006-16856057 3 10::219 11::290 227::173 315::241 0.944596288 1 A3SS

AT1G08845 chr1:2839840-2839850 3 80::11 58::8 66::115 44::82 0.944599944 1 A3SS

AT1G69800 chr1:26276144-26276278 1 0::81 0::73 44::111 36::101 0.944649982 1 IR

AT5G05660 chr5:1694208-1694213 2 98::5 97::13 26::91 35::127 0.944681547 1 A3SS

AT4G11410 chr4:6945782-6945866 6 12::363 5::447 185::208 232::267 0.944729905 1 Cassette

AT4G02005 chr4:878510-878579 4 21::14 26::16 11::9 13::13 0.944772792 1 IR

AT3G27320 chr3:10090592-10090689 1 68::170 113::180 92::156 98::176 0.944778714 1 IR

AT3G23670 chr3:8523443-8523784 13 0::36 0::61 25::26 24::26 0.944793261 1 Cassette_multi

AT5G67580 chr5:26957524-26957565 1 76::5 78::5 93::39 94::32 0.944798144 1 A5SS

AT3G04050 chr3:1050998-1051101 3 11::37 11::26 21::25 17::25 0.944852322 1 IR

AT5G62520 chr5:25098861-25098945 2 13::4 29::2 5::33 5::41 0.944863311 1 IR

AT4G23570 chr4:12299923-12300008 1 23::99 33::96 54::113 50::139 0.944868388 1 IR

AT5G22050 chr5:7303240-7303261 7 0::93 0::158 51::114 88::193 0.944930943 1 IR

AT3G18930 chr3:6523742-6523949 1 17::15 48::14 9::78 5::74 0.944949198 1 IR

AT1G30130 chr1:10589452-10589462 6 144::34 237::53 137::57 175::77 0.944955436 1 A3SS

AT5G06580 chr5:2014543-2014550 8 108::15 83::12 48::96 38::79 0.945015386 1 A3SS

AT1G51430 chr1:19068526-19068612 3 49::13 77::23 72::106 63::146 0.945024007 1 Cassette

AT1G60850 chr1:22400167-22400285 1 41::49 55::43 14::79 22::93 0.945085357 1 IR

AT2G35660 chr2:14988924-14989010 2 158::143 104::130 59::334 37::254 0.945087982 1 IR

AT2G46700 chr2:19184891-19184941 3 42::0 53::0 11::53 4::58 0.945089105 1 AltStart

AT4G02480 chr4:1085205-1085212 12 230::32 200::21 61::315 46::249 0.945131836 1 A3SS

AT4G26140 chr4:13243914-13243920 15 20::27 22::21 16::72 18::71 0.945206446 1 A5SS

AT1G06230 chr1:1907276-1907281 2 3::23 9::19 43::149 25::137 0.945229376 1 A3SS

AT3G60070 chr3:22186001-22186012 2 13::32 8::46 33::48 41::56 0.94546172 1 A3SS

AT2G22670 chr2:9638416-9638422 7 1861::69 1872::79 1791::275 1816::282 0.945496699 1 A3SS

AT3G02470 chr3:509201-509305 2 10041::9 8899::17 748::7908 661::6881 0.94550296 1 IR

AT3G04500 chr3:1213702-1213774 1 38::575 38::467 343::315 256::279 0.945585281 1 IR

AT3G59770 chr3:22084602-22084607 5 97::13 62::13 74::87 55::68 0.945637167 1 A3SS

AT1G32230 chr1:11615739-11615832 7 1065::9 1727::35 90::575 154::956 0.945850353 1 IR

AT3G06080 chr3:1835349-1835484 3 300::105 358::109 70::288 65::344 0.945908113 1 IR

AT4G03260 chr4:1428954-1429050 2 0::693 0::607 333::435 294::435 0.945919846 1 IR

AT1G07010 chr1:2153391-2153423 3 753::0 785::0 4::831 16::800 0.945932109 1 Cassette

AT3G26020 chr3:9513854-9513943 1 23::0 14::0 4::18 6::10 0.945950067 1 A5SS

AT4G37560 chr4:17647184-17647333 3 11::113 17::128 145::158 155::178 0.945957347 1 A5SS

AT4G12310 chr4:7310796-7311221 2 11::182 9::238 234::62 324::113 0.946002864 1 A3SS

AT3G61110 chr3:22612203-22612238 2-3 6838::53 8120::27 93::7582 97::8670 0.9460046 1 Cassette

AT1G34010 chr1:12361091-12361113 1 119::70 175::68 117::114 148::118 0.946043251 1 A5SS

AT2G32320 chr2:13729286-13729306 9 27::0 25::0 10::30 10::52 0.946122496 1 Cassette

AT1G15750 chr1:5420539-5420630 2 124::56 88::42 16::173 19::114 0.946166938 1 Cassette

AT3G06530 chr3:2024721-2024727 10 39::0 19::2 27::38 15::23 0.94628176 1 A3SS

AT1G47290 chr1:17338822-17338993 10 64::6 119::16 102::54 151::90 0.946387322 1 A3SS

AT5G13220 chr5:4219992-4220463 4 20::11 31::9 5::18 4::23 0.94638775 1 IR

AT1G13860 chr1:4745793-4745873 2 19::0 40::0 34::34 41::34 0.946415293 1 A5SS

AT4G25610 chr4:13066763-13066819 10 0::80 0::121 47::24 70::29 0.946735524 1 IR

AT1G15020 chr1:5173850-5173872 11 8::157 15::202 157::202 215::267 0.946909579 1 A3SS

AT5G22820 chr5:7624041-7624137 9 31::5 31::11 6::20 10::43 0.947023023 1 IR

AT1G67090 chr1:25048680-25048701 3 1026::246800 665::217941 238074::138552 213104::138147 0.94707758 1 A3SS

AT5G57870 chr5:23440859-23440871 3 13::746 2::603 611::785 496::619 0.947137635 1 A5SS

AT1G07480 chr1:2299275-2299370 1 259::36 214::37 32::124 28::106 0.947142423 1 IR

AT4G09040 chr4:5794974-5794986 6 43::737 27::710 587::464 545::446 0.947148939 1 A3SS

AT1G35530 chr1:13096441-13096520 23 10::1 15::0 3::16 3::15 0.947162502 1 IR

AT3G12290 chr3:3920336-3920349 2 697::10 702::18 226::791 240::784 0.947168156 1 A5SS

AT2G31751 chr2:13499866-13500162 7 15::144 8::213 29::319 44::333 0.947187194 1 IR

AT3G50050 chr3:18556018-18556035 4 5::8 2::22 15::21 30::31 0.947212256 1 A3SS

AT2G36360 chr2:15246850-15246858 4 2::82 5::64 22::48 18::39 0.947227675 1 A5SS

AT5G14105 chr5:4552239-4552244 2 14::555 8::652 616::251 703::267 0.947257625 1 A3SS

AT2G47070 chr2:19336879-19337111 1 16::517 8::330 233::290 148::200 0.947316363 1 IR

AT5G23380 chr5:7869628-7869708 5-6 142::14 213::16 8::110 14::169 0.947396246 1 AltStart

AT5G35410 chr5:13636342-13636347 9 8::12 11::16 10::33 15::33 0.947426375 1 A3SS

AT3G58720 chr3:21717630-21717697 2 6::89 0::72 65::79 61::93 0.947434535 1 Cassette

AT1G01650 chr1:236784-236865 2 84::2 70::8 8::107 10::121 0.947579328 1 IR

AT2G47900 chr2:19613031-19613128 1 11::0 26::0 225::170 184::105 0.94775517 1 A5SS

AT5G40810 chr5:16340259-16340589 2 1010::2 864::8 33::1336 34::1137 0.947791402 1 IR

AT3G15095 chr3:5082038-5082290 1 0::353 0::286 227::301 195::299 0.947911714 1 IR

AT4G32660 chr4:15757019-15757039 4 65::14 76::18 56::125 101::160 0.947920707 1 A5SS

AT5G46470 chr5:18848159-18848228 9 7::140 22::151 78::57 87::87 0.948091423 1 IR

AT1G02020 chr1:353162-353238 1 178::10 216::12 15::93 17::109 0.94809629 1 IR

AT3G54320 chr3:20118467-20118570 7 0::91 0::113 50::99 70::103 0.948172426 1 IR

AT4G26140 chr4:13243615-13243630 17 52::11 39::17 28::81 33::67 0.948195162 1 A3SS

AT1G13920 chr1:4759202-4759302 5 10::17 16::12 33::42 27::41 0.948212072 1 A5SS

AT5G26800 chr5:9426408-9426443 3 512::15 700::21 520::306 692::377 0.948254925 1 A3SS

AT1G56612 chr1:21214607-21214720 1 38::22 66::34 14::49 27::80 0.948273472 1 IR

AT4G28790 chr4:14219858-14219925 6 5::12 6::32 17::23 38::54 0.94838222 1 A3SS

AT4G30160 chr4:14753502-14753726 1 4::48 6::36 29::14 17::12 0.948440631 1 IR

AT3G49140 chr3:18214384-18214398 4 82::17 90::22 37::117 56::119 0.948447733 1 A3SS

AT3G26510 chr3:9712331-9712444 1 3244::366 1294::188 281::2418 138::1007 0.948597901 1 IR

AT3G02180 chr3:405359-405364 3 1419::41 1526::27 1034::724 1107::762 0.948698133 1 A3SS

AT3G13225 chr3:4261995-4262166 2 21::4 17::12 4::25 8::25 0.948713952 1 IR

AT3G10185 chr3:3145727-3145937 1-2 0::99 0::27 57::43 16::11 0.948758562 1 Cassette_multi

AT2G39290 chr2:16407927-16407943 2 240::16 267::13 237::316 257::321 0.948907027 1 A3SS

AT4G10430 chr4:6454927-6455101 11 2::236 4::326 127::128 159::195 0.948917449 1 IR

AT3G52660 chr3:19521823-19522564 1 56::35 77::46 18::74 24::88 0.949032202 1 IR

AT1G79830 chr1:30032895-30032901 3 16::133 5::105 76::114 59::89 0.949037991 1 A3SS

AT1G07120 chr1:2184759-2184954 5 30::0 18::6 5::29 7::28 0.949056514 1 AltEnd

AT1G16260 chr1:5560902-5560915 4 4::59 13::50 60::98 76::133 0.949111242 1 A3SS

AT5G50850 chr5:20689613-20689666 2 35::734 34::663 43::787 35::647 0.949168786 1 A3SS

AT5G04080 chr5:1104926-1104935 2 591::20 522::9 426::617 369::578 0.949205394 1 A3SS

AT4G30410 chr4:14871859-14872060 1 99::4 68::8 7::117 7::110 0.949293299 1 IR

AT4G26140 chr4:13244689-13244744 12 16::25 15::30 38::46 33::55 0.949330819 1 A3SS

AT3G30380 chr3:11976432-11976499 5 78::0 102::10 8::75 15::111 0.949359916 1 IR

AT1G78290 chr1:29458920-29459012 2 32::171 21::149 82::133 65::105 0.949397794 1 IR

AT5G47080 chr5:19125979-19125988 2 10::108 7::119 113::116 127::120 0.949402459 1 A3SS

AT2G47470 chr2:19483509-19483546 10 1692::0 1833::0 1601::662 1772::833 0.94943466 1 A3SS

AT1G29970 chr1:10499665-10500011 7 0::643 0::610 147::405 139::485 0.949454453 1 AltEnd

AT4G32520 chr4:15692342-15692348 2 11::196 3::170 114::342 100::247 0.94947106 1 A3SS

AT2G38280 chr2:16033647-16033654 20 29::255 23::412 219::141 321::205 0.949512814 1 A3SS

AT2G39000 chr2:16287770-16287877 1 327::1 369::2 380::290 386::249 0.949536881 1 A5SS

AT5G54900 chr5:22296569-22296650 2-3 764::45 846::59 28::800 43::920 0.949584355 1 Cassette

AT3G24440 chr3:8877375-8877381 2 38::24 77::37 66::103 99::115 0.949625329 1 A5SS

AT3G44740 chr3:16296153-16296330 6 5::10 11::20 16::13 45::41 0.949650641 1 A3SS

AT4G31120 chr4:15134654-15134660 10 4::153 11::190 116::231 135::281 0.949675254 1 A3SS

AT3G53460 chr3:19819582-19819600 5 8::2885 11::3709 2547::1279 3261::1777 0.949820182 1 A3SS

AT2G26150 chr2:11136246-11136276 2 3::28 1::12 17::25 6::13 0.949892634 1 Cassette

AT4G35450 chr4:16839702-16839845 1 645::240 558::244 170::406 166::364 0.949936735 1 IR

AT1G01320 chr1:123495-123501 24 1::453 0::528 241::838 279::1071 0.949992734 1 A3SS

AT5G19500 chr5:6580793-6580798 10 8::218 11::319 194::189 284::284 0.950118115 1 A5SS

AT1G18660 chr1:6421382-6421398 2 2::52 3::28 70::135 59::115 0.950263663 1 A3SS

AT3G16400 chr3:5566367-5566479 1 288::27 151::20 32::908 27::664 0.950316975 1 IR

AT1G53310 chr1:19888229-19888276 2 176::14 153::5 12::726 11::839 0.950326575 1 AltStart

AT3G47390 chr3:17462548-17462553 5 29::30 63::35 34::53 50::83 0.950344274 1 A3SS

AT2G26910 chr2:11483168-11483175 8 154::14 89::4 82::175 51::84 0.950368391 1 A3SS

AT4G01560 chr4:678630-678667 3 9::162 8::205 154::174 217::251 0.950387891 1 A3SS

AT2G37300 chr2:15663569-15663689 2 48::11 32::3 5::72 3::53 0.950396187 1 IR

AT3G07215 chr3:2296385-2296529 3 26::45 66::88 20::40 46::76 0.950417731 1 IR

AT3G25572 chr3:9288443-9288543 4 4::162 12::217 80::128 106::183 0.950439984 1 IR

AT1G56220 chr1:21044345-21044357 3 1695::2687 1356::2309 2640::3779 2016::3242 0.950505825 1 A3SS

AT2G42590 chr2:17732120-17732206 6 1947::2 2467::12 86::1194 113::1494 0.950523152 1 IR

AT3G19180 chr3:6636484-6636592 10 54::3 111::4 6::75 12::109 0.950574548 1 IR

AT2G17800 chr2:7740170-7740269 1 2::143 11::167 55::200 57::198 0.950610532 1 IR

AT4G37070 chr4:17466921-17466954 1 63::1 37::0 73::72 41::27 0.950639674 1 A5SS

AT5G53860 chr5:21868048-21868203 11 0::1448 0::1775 828::702 1013::763 0.950719979 1 Cassette

AT2G37450 chr2:15723804-15723921 2 13::61 23::52 94::127 152::215 0.950724387 1 A3SS

AT2G45700 chr2:18828498-18828503 5 102::6 153::25 98::89 149::153 0.9508472 1 A5SS

AT4G01040 chr4:455151-455157 8 186::12 248::19 129::92 179::173 0.950865254 1 A3SS

AT1G71310 chr1:26878860-26879290 3 139::21 151::38 16::121 27::138 0.95089561 1 IR

AT1G56090 chr1:20978432-20978437 3 52::28 25::22 32::67 19::76 0.950911038 1 A3SS

AT5G42470 chr5:16983437-16983442 6 7::78 10::88 59::85 68::100 0.951048624 1 A3SS

AT1G25350 chr1:8892124-8892150 9 223::0 157::0 201::185 155::146 0.951109491 1 A5SS

AT2G40270 chr2:16823694-16823715 3 0::60 8::66 79::97 83::104 0.951136475 1 A3SS

AT1G18420 chr1:6344692-6344715 5 21::0 25::10 6::20 17::43 0.951146889 1 A5SS

AT1G63990 chr1:23741492-23741498 12 428::13 458::21 412::434 442::471 0.951212499 1 A5SS

AT1G68580 chr1:25753368-25753422 2 81::32 77::35 24::84 20::91 0.951238346 1 Cassette

AT3G48115 chr3:17771564-17772700 2 20::17 28::44 366::765 399::873 0.951250242 1 A3SS

AT3G02570 chr3:545015-545048 2 283::5 234::12 41::288 38::259 0.951313654 1 A3SS

AT1G70980 chr1:26763215-26763418 1 18::110 12::97 93::105 103::82 0.951348454 1 A5SS

AT4G01593 chr4:691478-691631 2 71::11 126::55 11::80 25::146 0.951474605 1 IR

AT3G62080 chr3:22986304-22986380 1 0::58 0::32 34::32 17::27 0.951567074 1 IR

AT1G48650 chr1:17989581-17989708 19 0::570 0::661 285::239 313::324 0.951584009 1 IR

AT4G28040 chr4:13940850-13940859 2 18::44 13::22 68::260 33::128 0.951640928 1 A3SS

AT3G14420 chr3:4821788-4821849 2 7321::24 4464::8 12747::16950 8118::11272 0.951745272 1 A3SS

AT4G28030 chr4:13938625-13938634 2 827::146 843::130 522::947 525::959 0.95175034 1 A3SS

AT2G31900 chr2:13567945-13567965 9 1::11 1::20 14::7 20::8 0.951774529 1 A3SS

AT3G10490 chr3:3269937-3269946 5 149::13 167::2 109::161 117::210 0.951777345 1 A3SS

AT5G66600 chr5:26578426-26578519 1 2::11 0::18 7::9 12::10 0.951861378 1 IR

AT2G21940 chr2:9350674-9350955 2 7::36 18::32 12::74 11::92 0.951991728 1 AltStart

AT1G07120 chr1:2185600-2185916 2 20::8 10::8 5::39 9::33 0.952024322 1 Cassette

AT5G04740 chr5:1369616-1370514 3 0::671 0::823 303::361 384::443 0.952043217 1 Cassette_multi

AT5G12330 chr5:3989330-3989690 1 10::7 11::18 8::82 16::98 0.95205818 1 IR

AT4G14905 chr4:8526615-8526852 1 72::31 117::25 20::100 25::114 0.952175666 1 IR

AT1G31175 chr1:11141961-11142056 2 13::30 37::91 22::42 56::69 0.952178165 1 IR

AT5G59830 chr5:24105163-24105305 1 32::14 31::15 8::55 10::52 0.952205181 1 IR

AT5G19480 chr5:6571698-6571804 1 20::33 7::20 17::36 12::17 0.952213157 1 IR

AT5G50110 chr5:20375059-20375064 4 10::107 7::131 64::131 76::164 0.952250742 1 A3SS

AT2G35020 chr2:14759583-14759588 11 7::154 10::241 93::181 143::230 0.952279237 1 A5SS

AT3G26420 chr3:9674729-9674938 5-6 0::68 0::77 41::38 53::59 0.952424259 1 Cassette

AT2G47960 chr2:19627789-19627869 7-8 310::15 391::15 15::318 20::363 0.952498193 1 AltEnd

AT4G20310 chr4:10962216-10962238 3 11::24 13::32 32::50 39::56 0.952580313 1 A3SS

AT5G12150 chr5:3924561-3924578 22 11::1091 8::1282 997::555 1145::682 0.95258838 1 A3SS

AT2G04560 chr2:1591663-1591694 4 0::10 2::16 17::22 17::25 0.952635358 1 A3SS

AT4G08690 chr4:5552878-5552956 2 62::3 94::13 4::118 9::173 0.952697302 1 AltStart

AT5G19390 chr5:6538012-6538019 23 268::6 379::1 181::190 250::264 0.952762757 1 A3SS

AT5G10710 chr5:3380208-3380342 6 65::0 87::0 71::65 78::74 0.952789929 1 MXE

AT1G51950 chr1:19305949-19306029 1-2 469::13 381::2 12::233 7::198 0.952796632 1 AltStart

AT3G63445 chr3:23424663-23424899 2-3 15::14 36::18 80::66 112::86 0.952898857 1 AltEnd

AT3G07300 chr3:2327406-2327424 2 66::75 46::67 83::156 76::115 0.952906285 1 A3SS

AT4G24290 chr4:12596498-12596809 6 293::24 258::20 18::293 19::291 0.953014381 1 IR

AT5G09880 chr5:3083739-3083814 4-5 46::100 60::175 95::186 145::313 0.953046542 1 Cassette_multi

AT4G30490 chr4:14900047-14900122 10 18::301 27::320 162::166 169::184 0.95312271 1 IR

AT5G10710 chr5:3379209-3379214 1 1::59 5::67 70::64 73::41 0.953127883 1 A5SS

AT1G11870 chr1:4006281-4006306 10 280::15 291::20 290::205 328::274 0.953130389 1 A3SS

AT1G24350 chr1:8639285-8639422 6 312::0 327::1 19::162 44::211 0.953148904 1 Cassette

AT1G74590 chr1:28024097-28024207 2 10::70 32::101 42::31 48::49 0.953181007 1 IR

AT1G70580 chr1:26612760-26612791 1 13::245 12::122 226::40 114::29 0.953291747 1 A5SS

AT5G28500 chr5:10478595-10478922 1 0::2969 0::3264 1632::990 1803::1015 0.953308142 1 IR

AT4G37550 chr4:17645504-17645555 6-7 392::14 525::13 18::345 22::433 0.953378546 1 Cassette

AT5G43990 chr5:17698020-17698094 1 12::22 17::11 20::11 15::14 0.953423015 1 A5SS

AT2G43640 chr2:18098617-18098688 2 10::518 5::517 276::246 278::225 0.953438068 1 Cassette

AT3G08650 chr3:2626903-2626977 2 5::207 2::165 235::442 206::376 0.953472306 1 A3SS

AT1G61415 chr1:22660850-22660941 4 8::4 16::0 4::11 3::21 0.953498775 1 IR

AT1G10740 chr1:3568547-3568616 7 537::66 545::64 78::445 77::472 0.953498958 1 IR

AT4G35800 chr4:16961914-16961936 11 2::819 20::941 448::204 490::201 0.953577572 1 IR

AT5G12260 chr5:3967072-3967083 1 14::20 14::43 30::51 46::59 0.953588674 1 A5SS

AT1G60000 chr1:22094181-22094273 1 26::2665 38::3068 1576::1234 1805::1363 0.953608574 1 IR

AT1G64355 chr1:23886672-23886788 2 635::27 685::51 53::255 68::271 0.953610167 1 IR

AT2G31955 chr2:13585062-13585086 5 110::0 145::0 108::130 146::160 0.953646659 1 A5SS

AT5G47040 chr5:19096694-19096715 7 21::403 6::282 355::392 255::272 0.953749442 1 A3SS

AT5G52890 chr5:21445425-21445510 2 26::15 46::12 13::43 11::44 0.953860956 1 IR

AT1G03160 chr1:762391-762414 2 79::16 58::20 21::110 22::97 0.953880611 1 A5SS

AT2G40160 chr2:16777842-16777857 1 5::14 4::31 31::20 49::31 0.954149739 1 A5SS

AT3G62940 chr3:23264510-23264639 1 6::87 4::62 22::19 21::19 0.954149775 1 A5SS

AT4G15010 chr4:8574919-8575069 1 37::7 52::20 7::49 11::79 0.95419041 1 IR

AT2G29390 chr2:12612140-12612294 2 11::36 5::37 21::99 25::103 0.9542019 1 IR

AT3G10700 chr3:3348673-3348684 7 41::10 51::27 24::71 36::92 0.95421134 1 A3SS

AT1G75450 chr1:28314768-28314865 5 322::0 218::5 25::86 20::76 0.9542787 1 IR

AT1G69252 chr1:26037400-26037406 2 153::52 36::23 2306::1458 1939::1167 0.954294008 1 A3SS

AT5G57250 chr5:23194976-23195052 2 173::174 200::207 105::209 129::235 0.954299814 1 IR

AT2G46680 chr2:19166187-19166193 2 21::606 13::299 483::360 236::207 0.954304028 1 A3SS

AT5G51710 chr5:21005630-21005635 15 5::31 15::53 25::53 46::73 0.954399729 1 A3SS

AT1G29310 chr1:10252648-10252654 2 110::14 77::13 87::218 73::179 0.954449185 1 A3SS

AT1G59600 chr1:21890737-21890769 3 19::51 17::74 55::91 83::143 0.954521484 1 A3SS

AT2G41540 chr2:17326606-17326793 2 19::78 20::72 36::126 36::107 0.954625809 1 IR

AT5G57630 chr5:23342489-23342533 3 38::17 24::2 40::42 26::20 0.954655503 1 A5SS

AT3G51380 chr3:19074151-19074194 2 12::21 20::16 36::49 42::45 0.954674956 1 A3SS

AT5G10140 chr5:3174818-3174859 6 9::154 0::99 73::60 44::20 0.954684555 1 Cassette_multi

AT4G31210 chr4:15166295-15166307 3 5::27 1::17 8::54 7::40 0.954739527 1 A3SS

AT4G28290 chr4:14012151-14012168 2 649::22 406::7 208::337 116::198 0.954758188 1 A3SS

AT1G76630 chr1:28762906-28762991 15 0::145 0::137 81::61 73::78 0.954791184 1 IR

AT3G53090 chr3:19681053-19681063 5 17::9 17::16 18::20 21::26 0.954826499 1 A3SS

AT4G12970 chr4:7586514-7586526 2 707::8 751::13 510::654 550::688 0.954869095 1 A5SS

AT5G63440 chr5:25407021-25407029 2 101::11 144::27 85::156 107::168 0.954870787 1 A3SS

AT3G53710 chr3:19903642-19903651 4 8::306 10::480 365::189 552::263 0.954895167 1 A3SS

AT1G48635 chr1:17984899-17985110 5 107::0 182::0 11::132 15::187 0.954952896 1 Cassette_multi

AT4G13495 chr4:7843138-7843166 1 556::0 988::3 125::494 209::942 0.955001977 1 A5SS

AT3G49920 chr3:18506526-18506611 4 0::20 0::59 13::16 40::36 0.955043483 1 Cassette

AT4G36690 chr4:17294430-17294704 11 261::264 417::358 156::524 212::775 0.955060615 1 IR

AT2G29525 chr2:12638397-12638505 1 1::33 0::37 20::16 21::21 0.955109367 1 IR

AT4G22850 chr4:11995815-11995896 4 0::124 0::218 64::50 117::64 0.955283382 1 IR

AT5G01780 chr5:303725-303854 2 36::6 37::12 11::55 26::83 0.955342792 1 A3SS

AT3G61070 chr3:22606597-22606712 1 5::140 2::137 76::43 72::38 0.955345096 1 IR

AT3G59330 chr3:21925941-21927379 4 4::31 0::75 32::34 70::56 0.955359353 1 Cassette_multi

AT4G17570 chr4:9785984-9785996 3 26::45 15::48 39::78 37::63 0.955513708 1 A5SS

AT5G03540 chr5:889983-890104 2 106::2 85::8 8::146 7::118 0.955630029 1 IR

AT1G70620 chr1:26627512-26627540 3 4::19 13::9 76::93 83::105 0.955716334 1 A5SS

AT2G37860 chr2:15858347-15858442 6 475::0 562::9 29::508 39::595 0.955763661 1 IR

AT4G31115 chr4:15130346-15130419 1 158::23 143::37 32::145 38::158 0.955798291 1 IR

AT2G39580 chr2:16512364-16512403 5 42::0 31::0 5::31 4::35 0.955801999 1 A5SS

AT1G15810 chr1:5445526-5445531 2 135::13 124::10 158::164 148::167 0.955898029 1 A3SS

AT3G48810 chr3:18099427-18099537 2 11::0 35::0 3::14 4::36 0.955898629 1 IR

AT1G74970 chr1:28158864-28158912 1 3146::126 3013::97 3108::4625 2919::3979 0.955900999 1 A5SS

AT3G53780 chr3:19926327-19926398 1 0::114 1::134 64::51 73::42 0.955938233 1 IR

AT2G06510 chr2:2585220-2585290 2 0::28 0::29 20::13 17::17 0.955955843 1 IR

AT3G55630 chr3:20637570-20637575 6 57::139 72::195 131::200 186::262 0.955978346 1 A3SS

AT1G29357 chr1:10275147-10275219 2 18::15 43::18 28::67 36::111 0.95609186 1 A3SS

AT5G44562 chr5:17947671-17947776 2 214::26 214::45 31::158 38::163 0.956149182 1 IR

AT5G09330 chr5:2892775-2892854 5 50::738 74::989 263::253 409::334 0.95617279 1 IR

AT4G26140 chr4:13243722-13243732 16 52::15 39::20 52::56 41::47 0.956208281 1 A5SS

AT1G33680 chr1:12206359-12206431 6-7 144::84 138::87 52::373 79::450 0.956243168 1 Cassette

AT2G01220 chr2:124794-124811 8 17::177 37::245 51::217 83::302 0.956258001 1 A3SS

AT4G26640 chr4:13439763-13439807 2 93::23 81::17 36::162 37::186 0.95631201 1 AltStart

AT1G01100 chr1:50447-50496 4 53::33899 69::35613 18768::14455 19737::14889 0.956458048 1 IR

AT5G50240 chr5:20451533-20451544 2 74::12 66::4 15::119 6::109 0.956468317 1 AltStart

AT4G38540 chr4:18023713-18023736 4 104::11 103::23 105::144 107::158 0.956521104 1 A3SS

AT5G23480 chr5:7917868-7917938 4-5 0::10 0::17 7::9 10::18 0.956530954 1 Cassette

AT1G08490 chr1:2687351-2687367 5 13::196 21::194 185::266 186::267 0.956673503 1 A3SS

AT1G19715 chr1:6819313-6819430 1 91::6 117::21 12::86 20::138 0.956710104 1 IR

AT3G01202 chr3:70711-70738 2 193::0 164::1 25::192 35::202 0.956777153 1 A5SS

AT5G52230 chr5:21211678-21211782 1 5::9 0::14 7::15 10::11 0.956808785 1 IR

AT1G05710 chr1:1716064-1716102 3 99::1 80::3 11::65 13::76 0.956835216 1 Cassette

AT2G27150 chr2:11602330-11602430 2 5::9 6::6 8::24 10::16 0.957093724 1 A3SS

AT1G05710 chr1:1715780-1715842 2 99::1 80::3 3::115 14::134 0.957123734 1 AltStart

AT4G38470 chr4:18000016-18000044 3 6::202 12::168 26::210 28::194 0.957158104 1 A3SS

AT3G14420 chr3:4821638-4821753 1 7320::24 4461::8 339::819 234::501 0.957188379 1 A5SS

AT1G67580 chr1:25327511-25327624 6 0::331 3::513 159::168 251::210 0.957233931 1 IR

AT1G21590 chr1:7567759-7567764 6 9::253 8::177 179::309 137::300 0.957378808 1 A3SS

AT1G78265 chr1:29451682-29451707 2 4::8 1::12 104::129 105::158 0.957388602 1 A3SS

AT5G16715 chr5:5485996-5486036 3 74::15 98::15 21::128 22::133 0.95741364 1 A5SS

AT2G34780 chr2:14669035-14669080 3 7::9 4::6 10::21 5::23 0.957433628 1 Cassette

AT3G63340 chr3:23391947-23391959 21 10::101 12::202 110::31 225::60 0.957436348 1 A3SS

AT1G12810 chr1:4368020-4368118 3 606::0 758::0 30::457 25::497 0.957456067 1 Cassette

AT1G69935 chr1:26342826-26342834 2 48::244 97::337 366::406 474::505 0.95751337 1 A3SS

AT1G13920 chr1:4759374-4759455 6 8::26 16::25 27::42 26::51 0.957513396 1 Cassette

AT4G22233 chr4:11763788-11763794 2 0::97 13::153 104::36 157::39 0.957524728 1 A3SS

AT5G51970 chr5:21111594-21111658 1 14::11 3::7 13::821 15::1027 0.9575566 1 AltStart

AT2G26310 chr2:11201968-11202094 2 0::22 0::40 13::12 27::20 0.957606534 1 IR

AT2G23600 chr2:10042813-10042824 2 14::916 15::1225 687::808 955::1289 0.957617527 1 A3SS

AT2G26030 chr2:11091783-11091798 4 77::21 87::20 37::57 39::72 0.957676117 1 A3SS

AT3G16170 chr3:5478942-5478988 10 52::14 64::6 55::66 80::87 0.957845721 1 A3SS

AT1G57610 chr1:21337626-21337704 3 4::318 12::444 187::124 255::162 0.957904269 1 IR

AT1G03310 chr1:813581-813604 1 57::27 35::25 50::65 38::41 0.957905018 1 A5SS

AT2G41670 chr2:17377565-17377588 9 609::146 576::163 240::852 274::952 0.957931783 1 A5SS

AT1G10900 chr1:3636998-3637023 1 13::84 6::77 18::76 16::84 0.957942662 1 A5SS

AT1G27920 chr1:9729134-9729180 8 11::4 6::8 16::25 39::56 0.957978841 1 A5SS

AT3G06330 chr3:1917423-1917507 1 127::9 107::3 11::54 7::39 0.958173935 1 A5SS

AT5G44750 chr5:18056573-18056585 13 13::2 7::3 37::67 93::113 0.95818369 1 A5SS

AT5G65380 chr5:26125638-26125702 2 14::948 9::655 73::720 54::489 0.958266214 1 A5SS

AT1G58590 chr1:21743298-21743483 2 2::17 12::69 5::17 22::48 0.958315696 1 IR

AT1G16560 chr1:5668573-5668739 2 279::3 284::8 7::222 17::239 0.958391676 1 Cassette

AT2G06925 chr2:2842748-2843011 2 8::378 7::528 177::95 254::146 0.958394382 1 Cassette_multi

AT2G13290 chr2:5511999-5512016 2 7::74 3::35 24::139 13::123 0.95839735 1 A3SS

AT5G66055 chr5:26417734-26417814 5 357::78 434::79 71::435 77::466 0.958482429 1 IR

AT1G07700 chr1:2380354-2380385 3 666::8 665::10 690::796 719::913 0.95851598 1 A3SS

AT4G30910 chr4:15043338-15043346 7 92::6 151::14 27::133 69::246 0.958552118 1 A3SS

AT4G02530 chr4:1112936-1112943 2 1691::11 1719::23 989::1958 1018::2010 0.958630724 1 A3SS

AT2G43090 chr2:17919329-17919417 1 2::3797 0::4288 2182::1788 2504::1926 0.95868109 1 IR

AT5G03730 chr5:974708-974850 15 36::180 53::313 119::143 220::270 0.958693835 1 IR

AT1G13990 chr1:4795282-4795565 2 306::0 247::0 8::258 15::230 0.958732778 1 Cassette_multi

AT3G22260 chr3:7873335-7873350 11 20::25 17::29 22::19 22::30 0.958742172 1 A3SS

AT4G30470 chr4:14894646-14894653 2 13::288 5::155 287::294 151::159 0.958759706 1 A5SS

AT2G34720 chr2:14651447-14651527 1-2 291::15 275::11 18::556 13::691 0.958797356 1 AltStart

AT1G16560 chr1:5668919-5668945 3 151::129 143::145 203::335 192::370 0.958855761 1 A3SS

AT5G53620 chr5:21781161-21781174 2 2::148 5::73 40::185 24::121 0.958864546 1 A3SS

AT5G63370 chr5:25385151-25385156 2 41::3 63::18 234::179 230::208 0.95890356 1 A5SS

AT5G49230 chr5:19959565-19959574 3 160::16 152::11 129::224 146::218 0.958929459 1 A5SS

AT5G52580 chr5:21338670-21338688 4 28::112 17::95 43::145 28::109 0.958946829 1 A3SS

AT1G62130 chr1:22966484-22966506 10 23::0 12::0 32::24 18::20 0.95896077 1 A5SS

AT4G04340 chr4:2122895-2123194 1 177::10 106::10 12::183 8::102 0.958984431 1 IR

AT2G43430 chr2:18037621-18038002 1 54::5 56::8 4::81 7::66 0.958996201 1 IR

AT2G28760 chr2:12338740-12338854 2 15::16 8::5 15::88 6::88 0.959032365 1 AltStart

AT1G05220 chr1:1513213-1513293 2-3 0::169 1::208 132::76 154::73 0.959048667 1 AltStart

AT3G61610 chr3:22799040-22799499 1 37::27 23::23 15::68 14::62 0.95907599 1 IR

AT2G39435 chr2:16466646-16466691 2 0::32 0::11 16::20 12::22 0.959122657 1 Cassette

AT5G05980 chr5:1800172-1800264 15 10::180 7::282 177::192 278::299 0.959230171 1 A3SS

AT1G78480 chr1:29525785-29525860 2 98::0 103::0 9::74 17::74 0.959299685 1 Cassette

AT3G47675 chr3:17576716-17576755 before first 16::42 22::74 78::82 118::183 0.959310195 1 Cassette

AT5G60640 chr5:24371347-24371440 11 466::23 639::32 53::414 78::608 0.959363578 1 IR

AT3G06810 chr3:2149802-2149901 14 5::368 10::460 178::193 213::234 0.959495207 1 IR

AT5G39830 chr5:15943411-15943422 2 27::92 37::92 80::125 87::156 0.959645975 1 A5SS

AT5G62640 chr5:25150893-25150911 8 7::75 0::50 76::142 48::102 0.959711817 1 A3SS

AT2G17033 chr2:7401633-7401688 2 132::22 100::10 205::243 144::185 0.959765279 1 A3SS

AT5G39600 chr5:15854003-15854118 3 11::502 26::592 277::211 328::268 0.959778372 1 IR

AT2G45320 chr2:18685125-18685153 3 9::4 33::6 12::22 37::53 0.959786935 1 A5SS

AT5G49010 chr5:19867670-19867806 4 63::3 87::12 6::82 10::105 0.959888435 1 IR

AT2G31840 chr2:13539724-13539737 1 122::12 80::12 99::85 64::61 0.959999585 1 A5SS

AT5G22000 chr5:7279556-7279583 8 4::595 4::701 559::841 663::891 0.960027982 1 A5SS

AT1G07650 chr1:2364691-2364709 3 44::2 77::0 6::64 6::85 0.960087646 1 A3SS

AT5G17230 chr5:5662615-5662773 1 1002::24 821::17 925::171 674::119 0.960125102 1 A5SS

AT5G43930 chr5:17678672-17678678 9 1::55 3::48 36::72 37::104 0.960137349 1 A3SS

AT1G24300 chr1:8617437-8617452 7 11::20 8::29 22::93 32::103 0.960165242 1 A3SS

AT5G43100 chr5:17301017-17301028 6 109::13 104::6 27::129 20::119 0.960298374 1 A5SS

AT3G04870 chr3:1342720-1342832 1 18::225 4::114 89::179 44::93 0.960309105 1 IR

AT2G43080 chr2:17916112-17916308 3 13::75 24::100 23::113 23::150 0.960430346 1 A3SS

AT5G04520 chr5:1290770-1290828 1 39::8 49::9 48::77 65::83 0.960463937 1 A5SS

AT3G08030 chr3:2564269-2564470 1 1377::17 953::8 56::1580 44::1109 0.960500766 1 IR

AT1G18730 chr1:6461844-6461910 6 1167::6 1612::10 31::593 46::814 0.96051747 1 Cassette

AT5G48230 chr5:19555011-19555089 2 307::14 233::10 41::302 30::231 0.960530521 1 IR

AT5G52210 chr5:21205078-21205225 2 40::1 37::4 4::74 7::81 0.960550029 1 AltStart

AT3G52180 chr3:19350346-19350613 10 139::6 132::16 6::163 15::195 0.960584464 1 IR

AT5G48950 chr5:19847063-19847132 2 5::11 17::17 6::9 24::16 0.960597633 1 A3SS

AT5G55670 chr5:22545207-22545583 1 0::159 0::187 127::115 131::122 0.960618762 1 IR

AT5G02650 chr5:598426-598467 4 78::0 79::0 3::71 8::78 0.960649615 1 Cassette

AT3G50670 chr3:18827439-18828349 6 143::174 175::194 103::317 129::385 0.960697523 1 IR

AT5G35100 chr5:13360867-13360976 1 0::1488 0::1340 809::1014 702::787 0.960698527 1 IR

AT1G77290 chr1:29038543-29038877 1 32::15 25::28 10::39 16::41 0.960837613 1 IR

AT5G67440 chr5:26914625-26914704 3 65::8 63::9 14::83 14::103 0.960838893 1 IR

AT3G61690 chr3:22830014-22832346 7 11::153 8::186 66::103 78::143 0.960864472 1 Cassette

AT2G41100 chr2:17138307-17138573 3 0::241 5::210 173::231 120::171 0.960910541 1 Cassette

AT2G04270 chr2:1476286-1476297 5 13::44 6::30 29::55 20::69 0.960929151 1 A3SS

AT3G01202 chr3:69932-70072 1 187::7 236::9 11::203 18::254 0.96099066 1 IR

AT2G40020 chr2:16712509-16712597 5 422::59 408::82 48::336 58::329 0.960993299 1 IR

AT2G43640 chr2:18099028-18099074 1 253::15 252::9 218::51 197::36 0.961008538 1 A5SS

AT4G21450 chr4:11427430-11427692 5 586::27 670::48 28::576 45::659 0.961012125 1 IR

AT5G58240 chr5:23559057-23559082 2 332::13 463::9 332::321 487::439 0.96102894 1 A5SS

AT1G19485 chr1:6748158-6748163 5 23::13 16::17 13::31 15::37 0.961105512 1 A5SS

AT1G06670 chr1:2046215-2046324 14 14::217 11::284 128::101 153::161 0.961213896 1 IR

AT4G31210 chr4:15166866-15166878 4 13::34 3::30 16::51 8::46 0.961228061 1 A3SS

AT1G11592 chr1:3896090-3896212 3 84::3 123::3 7::108 9::131 0.961230107 1 IR

AT1G09140 chr1:2944960-2944966 6 577::6 724::14 377::498 486::630 0.961248457 1 A3SS

AT1G79110 chr1:29759999-29760008 3 12::73 23::159 80::150 138::276 0.96137019 1 A3SS

AT2G21160 chr2:9070154-9070294 8 1904::0 2247::0 1537::318 1791::389 0.961483614 1 A3SS

AT3G07930 chr3:2527737-2527745 1 1::61 4::48 52::27 50::33 0.961586267 1 A5SS

AT2G45330 chr2:18686604-18686613 2 103::3 133::1 85::139 90::154 0.961659549 1 A3SS

AT1G16060 chr1:5509303-5509383 3-4 3::13 4::21 9::98 11::138 0.96171993 1 AltStart

AT5G15845 chr5:5176647-5176757 8 1053::50 823::62 72::829 60::620 0.961751032 1 IR

AT3G50790 chr3:18881180-18881202 3 23::288 34::300 300::324 301::369 0.961766077 1 A3SS

AT2G41150 chr2:17154995-17155026 4 12::0 39::0 7::25 16::59 0.961767284 1 A5SS

AT2G31900 chr2:13565067-13565272 21 8::3 7::9 5::18 11::16 0.961769008 1 Cassette

AT2G21250 chr2:9103638-9103721 5 601::10 676::18 46::361 57::425 0.961796318 1 IR

AT1G52670 chr1:19615950-19615975 5 6::426 9::415 394::487 394::487 0.961805954 1 A3SS

AT4G16890 chr4:9501911-9502382 5 39::30 50::24 192::166 258::249 0.961818157 1 A5SS

AT1G73430 chr1:27603848-27603949 1 5::72 7::66 52::46 39::35 0.961925447 1 IR

AT3G24180 chr3:8741167-8741172 3 1::69 4::41 60::75 38::49 0.961937238 1 A3SS

AT1G14240 chr1:4865810-4865822 4 39::0 18::0 31::43 17::41 0.961939861 1 A3SS

AT3G28130 chr3:10465946-10465988 2 125::10 153::9 24::116 25::138 0.962046026 1 A5SS

AT4G22890 chr4:12007782-12007788 2 3751::187 3008::151 1956::3354 1566::2797 0.962073951 1 A3SS

AT3G23900 chr3:8634305-8634616 3 11::33 11::38 42::50 48::75 0.962131447 1 A3SS

AT1G73670 chr1:27701832-27701837 6 155::10 144::14 69::161 65::178 0.962327799 1 A3SS

AT5G22620 chr5:7518569-7518634 6 65::192 69::220 144::212 184::255 0.962328419 1 Cassette

AT2G47860 chr2:19600273-19600336 3 38::8 44::8 47::66 45::84 0.962443364 1 A3SS

AT1G21440 chr1:7503174-7503179 4 1487::20 1267::29 798::1518 698::1324 0.962453637 1 A5SS

AT2G32179 chr2:13672808-13672895 3 115::0 109::0 37::351 64::398 0.962522668 1 A5SS

AT4G38960 chr4:18161120-18161233 1 8::5 13::7 5::113 5::103 0.962554666 1 IR

AT4G04220 chr4:2034088-2034106 2 21::2 21::1 23::21 21::29 0.962570536 1 A3SS

AT2G33120 chr2:14044530-14044582 2 594::0 590::0 625::640 613::596 0.962638518 1 A5SS

AT3G60510 chr3:22357762-22357838 10 21::2 54::2 6::29 9::59 0.962648816 1 IR

AT5G06210 chr5:1879180-1879189 3 465::3 496::12 287::528 323::645 0.962669343 1 A3SS

AT1G63855 chr1:23700726-23700767 2 10::57 5::50 76::92 72::95 0.962704213 1 A3SS

AT5G54570 chr5:22170056-22170089 1 13::0 16::2 14::22 29::49 0.962764046 1 A5SS

AT4G39870 chr4:18502092-18502206 1 4::42 8::53 31::27 35::30 0.962772063 1 IR

AT3G06480 chr3:1988133-1988194 3-4 47::50 45::62 29::76 35::101 0.962855002 1 Cassette

AT4G32530 chr4:15693564-15693655 3 700::27 762::40 79::558 96::640 0.962865807 1 IR

AT3G08990 chr3:2742530-2742610 before first 4::13 14::13 6::44 10::60 0.962977571 1 AltEnd

AT3G52340 chr3:19407953-19408021 4 196::12 178::3 127::382 124::373 0.962980887 1 A5SS

AT2G20830 chr2:8969811-8970018 1 36::191 51::200 60::227 60::272 0.963041026 1 IR

AT5G62480 chr5:25089295-25089374 1 6::48 4::56 28::31 28::30 0.963080785 1 IR

AT4G14400 chr4:8296213-8296226 3 81::0 130::0 74::104 126::149 0.963117631 1 A3SS

AT4G39560 chr4:18382506-18382593 1 56::34 92::61 22::71 39::77 0.963121017 1 IR

AT2G25840 chr2:11022707-11022743 4 263::3 270::3 301::334 286::279 0.963138201 1 A3SS

AT5G65685 chr5:26275686-26275722 2 49::20 64::28 53::98 45::131 0.963149699 1 Cassette

AT4G19160 chr4:10477530-10477821 1 2::984 6::853 446::387 392::351 0.963194835 1 IR

AT3G52050 chr3:19305034-19305212 1 25::64 20::70 41::92 43::67 0.96327041 1 IR

AT2G43950 chr2:18200684-18200932 5 430::10 504::19 16::235 30::277 0.963283156 1 IR

AT5G10190 chr5:3200708-3200722 3 100::10 111::7 82::74 89::106 0.963301902 1 A3SS

AT1G17455 chr1:5997886-5997894 2 62::0 70::0 74::55 83::84 0.963481211 1 A3SS

AT2G33360 chr2:14141195-14141661 2 0::127 0::136 76::88 70::87 0.96348577 1 IR

AT5G59890 chr5:24122601-24122674 2 1723::6 1467::4 15::1248 18::1193 0.96353061 1 AltStart

AT5G04220 chr5:1157352-1157378 6 10::4 9::7 12::20 11::16 0.963537109 1 A5SS

AT5G23630 chr5:7963669-7963679 16 12::117 8::102 128::118 102::116 0.963543616 1 A3SS

AT1G68920 chr1:25915292-25915519 1 2::81 9::62 37::60 38::55 0.963573301 1 IR

AT2G43490 chr2:18055273-18055279 11 12::31 4::50 30::45 40::66 0.963623098 1 A3SS

AT1G23750 chr1:8400622-8400645 2 194::13 140::4 179::221 129::173 0.963796923 1 A3SS

AT1G56720 chr1:21265565-21265570 3 48::27 49::22 48::98 35::80 0.963850425 1 A3SS

AT5G18440 chr5:6112926-6113023 1 3::20 10::27 9::17 17::26 0.963878345 1 IR

AT1G09195 chr1:2968199-2968254 11 0::16 2::19 25::14 36::20 0.963908426 1 A3SS

AT1G70550 chr1:26599066-26599082 6 34::115 33::95 58::170 52::147 0.963919802 1 A3SS

AT5G26290 chr5:9227069-9227078 5 41::0 93::0 55::67 98::107 0.963975929 1 A3SS

AT5G38410 chr5:15377719-15377740 3 477::128786 134::119010 87582::24173 80706::23579 0.964051115 1 A3SS

AT4G01060 chr4:460696-460702 2 106::240 61::149 262::356 162::216 0.964069971 1 A3SS

AT5G15860 chr5:5179029-5179106 7 190::10 235::3 27::154 28::153 0.964075044 1 IR

AT3G44110 chr3:15869216-15869384 6 0::6673 0::7698 3716::1737 4345::2150 0.964101536 1 IR

AT1G31580 chr1:11311405-11311437 1 11736::16 14367::29 1552::4289 1867::4630 0.964283673 1 A5SS

AT1G21190 chr1:7420697-7420740 1 21::703 33::876 67::469 92::583 0.964340331 1 A5SS

AT1G15860 chr1:5454896-5455055 1 137::59 104::63 29::132 26::114 0.964407828 1 IR

AT4G38150 chr4:17900850-17901176 1 1::109 0::120 31::125 33::162 0.964426922 1 IR

AT5G22770 chr5:7588100-7588194 1 20::157 18::106 94::105 59::89 0.96443742 1 IR

AT2G29650 chr2:12675644-12675699 1 489::11 502::9 483::434 527::403 0.964451301 1 A5SS

AT3G21300 chr3:7495100-7495109 4 15::89 25::73 24::127 32::112 0.96445641 1 A5SS

AT3G23580 chr3:8460735-8460770 2 199::9 137::8 211::263 152::232 0.964477437 1 A3SS

AT4G02480 chr4:1085287-1085293 11 232::32 204::21 181::285 151::211 0.964512417 1 A5SS

AT5G26622 chr5:9356569-9356891 1 17::5 14::0 104::102 184::138 0.964513741 1 AltStart

AT4G36650 chr4:17283809-17283895 3 7::85 0::110 46::77 64::95 0.964550054 1 IR

AT1G61600 chr1:22730321-22730453 2 17::111 10::109 64::55 70::57 0.964569901 1 IR

AT2G40460 chr2:16899548-16899582 3 22::157 10::119 186::220 123::124 0.964571923 1 A5SS

AT4G28080 chr4:13951128-13951218 22 775::26 680::14 54::839 50::775 0.964579857 1 A3SS

AT1G68890 chr1:25899959-25899972 10 34::24 25::18 45::73 33::54 0.964855248 1 A3SS

AT1G24510 chr1:8687689-8687903 1 804::1 751::10 31::709 33::619 0.964870684 1 IR

AT2G29350 chr2:12601230-12601235 2 13::0 18::0 12::20 15::25 0.964878628 1 A3SS

AT4G15180 chr4:8654942-8654995 5 28::0 25::0 30::30 27::29 0.964917985 1 A3SS

AT5G48010 chr5:19460512-19460558 12 0::608 0::463 288::458 223::341 0.964931096 1 Cassette

AT5G02470 chr5:544715-544844 1 33::8 20::6 13::6 16::6 0.964964328 1 A5SS

AT3G01480 chr3:189713-189741 4 519::665 595::702 596::1546 725::1915 0.965001638 1 A3SS

AT3G07180 chr3:2283002-2283889 6 0::246 0::261 174::150 156::165 0.965011597 1 Cassette_multi

AT5G28850 chr5:10879359-10879451 5 88::5 102::1 14::79 11::86 0.965053556 1 IR

AT2G26000 chr2:11084239-11084292 9 0::48 0::55 32::32 32::42 0.965177828 1 Cassette

AT1G34065 chr1:12400965-12400986 1 16::9 39::13 14::6 34::12 0.965191219 1 A5SS

AT5G20680 chr5:6998779-6998998 2 3::17 4::26 11::22 18::29 0.965210364 1 Cassette

AT3G59040 chr3:21823842-21824178 1 134::1 63::5 5::107 4::94 0.965234813 1 IR

AT5G51290 chr5:20845206-20845211 10 44::13 44::15 40::58 51::80 0.96533208 1 A3SS

AT2G17320 chr2:7534387-7534418 7 10::112 6::156 105::124 145::151 0.965339049 1 A3SS

AT4G17020 chr4:9577972-9578156 15 2::58 5::58 29::34 42::40 0.9653944 1 IR

AT5G52547 chr5:21327319-21327404 2 5::13 3::7 7::53 9::53 0.96541887 1 AltStart

AT2G28670 chr2:12301155-12301189 2 14::370 18::387 187::206 203::217 0.965421359 1 IR

AT1G70180 chr1:26428946-26429047 7 171::17 255::26 21::147 37::191 0.965440217 1 IR

AT5G11030 chr5:3491225-3491237 13 1::108 11::105 115::64 115::77 0.965500035 1 A3SS

AT2G40160 chr2:16778715-16778913 5 11::15 3::25 16::21 22::21 0.965563124 1 A3SS

AT2G44920 chr2:18526278-18526338 7 12::1805 6::2139 905::439 1050::475 0.965624172 1 Cassette

AT3G05510 chr3:1595451-1595604 1 1::40 5::37 31::28 32::31 0.965626919 1 IR

AT4G09010 chr4:5778912-5778931 3 2081::36 1668::31 392::1996 346::1599 0.965630769 1 A3SS

AT5G62165 chr5:24966818-24966879 4 5::18 6::31 10::22 16::27 0.965641866 1 Cassette

AT3G09470 chr3:2912360-2912366 8 286::9 362::18 236::333 297::439 0.965692734 1 A5SS

AT1G80030 chr1:30105341-30105377 11 88::81 111::136 315::408 335::500 0.965705106 1 A5SS

AT2G41090 chr2:17136480-17136488 3 1836::4 4384::12 1946::911 4788::2419 0.965775908 1 A3SS

AT5G45710 chr5:18541791-18541885 2 29::2 28::10 5::57 10::64 0.965822235 1 IR

AT1G18900 chr1:6532336-6532440 3 0::142 0::191 55::106 82::114 0.965845641 1 IR

AT1G02090 chr1:387672-388268 7 374::32 392::21 23::457 28::482 0.965876128 1 IR

AT1G50970 chr1:18899558-18899584 13 18::0 33::0 6::23 7::55 0.965941472 1 Cassette

AT1G64860 chr1:24100638-24100643 10 1077::76 1347::78 998::566 1277::768 0.965997146 1 A3SS

AT1G50440 chr1:18685966-18686061 1 13::24 41::38 15::81 25::134 0.966079795 1 IR

AT4G28706 chr4:14167969-14167975 2 125::12 110::18 122::142 109::146 0.966140033 1 A3SS

AT1G59750 chr1:21979423-21979619 1 14::157 21::137 67::13 68::12 0.966153135 1 A5SS

AT1G78265 chr1:29451643-29451707 1 8::176 12::170 93::122 92::151 0.966356847 1 IR

AT5G40830 chr5:16355098-16355201 2 6::315 9::453 171::125 253::163 0.966390095 1 IR

AT4G01590 chr4:690246-690385 1 15::247 9::215 184::233 169::217 0.966440579 1 IR

AT3G05910 chr3:1764718-1765059 10 0::1055 0::1377 517::217 695::274 0.966476617 1 Cassette_multi

AT1G80210 chr1:30163479-30163564 9 102::24 135::52 23::56 44::71 0.966486443 1 IR

AT3G16630 chr3:5667301-5667315 3 17::112 11::107 123::197 119::149 0.966492297 1 A3SS

AT1G14570 chr1:4983735-4983754 2 194::4 178::3 234::313 245::341 0.966499697 1 A3SS

AT1G02205 chr1:421720-421735 9 1::523 0::187 143::715 48::253 0.966587746 1 A3SS

AT3G19460 chr3:6748904-6748909 5 78::63 109::80 73::139 99::182 0.966616565 1 A5SS

AT3G23600 chr3:8474130-8474149 2 10::2534 8::2362 333::1859 310::1719 0.966663886 1 A3SS

AT1G05970 chr1:1816402-1816408 3 60::116 82::118 151::133 156::138 0.966769931 1 A3SS

AT3G22690 chr3:8021908-8022010 1 0::33 0::42 22::7 23::11 0.96684116 1 IR

AT1G54730 chr1:20428049-20428111 13 26::188 25::156 105::107 89::111 0.966844614 1 Cassette

AT3G09150 chr3:2803886-2804070 1 40::1 56::5 4::36 5::46 0.966897618 1 IR

AT5G47900 chr5:19394629-19394634 13 65::0 65::2 19::68 34::80 0.96692283 1 A5SS

AT5G07890 chr5:2519669-2519798 2 19::2 20::4 5::21 7::20 0.966927748 1 Cassette

AT4G37430 chr4:17598188-17598212 2 77::38 113::51 80::121 112::140 0.966945128 1 A3SS

AT5G22580 chr5:7502927-7502935 2 2505::10 2806::8 2609::1811 2973::2235 0.966947171 1 A3SS

AT4G39925 chr4:18517972-18518052 1 21::2 25::8 24::44 30::64 0.966998765 1 AltStart

AT5G35620 chr5:13825133-13825576 3 867::2 1209::16 11::879 20::1108 0.967004516 1 IR

AT1G29730 chr1:10402160-10402306 19-20 0::14 0::17 10::7 9::8 0.967021171 1 Cassette_multi

AT1G10050 chr1:3281440-3281447 6 7::6 11::11 8::27 9::22 0.967080427 1 A3SS

AT5G04280 chr5:1192953-1194495 3 13::272 27::331 49::462 67::598 0.967098849 1 A3SS

AT1G20810 chr1:7232289-7232320 1 353::80 401::113 136::359 173::363 0.967259837 1 A5SS

AT2G28810 chr2:12363734-12363936 1 58::12 16::3 5::18 3::7 0.967269532 1 A5SS

AT1G68660 chr1:25778545-25778560 2 28::2358 28::2018 2051::2132 1816::2010 0.967332266 1 A3SS

AT1G75210 chr1:28224593-28224673 1-2 101::12 65::16 13::146 14::172 0.967332885 1 AltStart

AT3G12350 chr3:3932456-3932530 1 253::9 302::4 41::290 45::286 0.967406088 1 A5SS

AT5G37380 chr5:14819035-14819105 2 21::35 9::29 24::85 26::93 0.967426044 1 Cassette

AT2G30990 chr2:13189249-13189340 3 5::15 3::21 14::20 12::27 0.967435472 1 IR

AT5G23870 chr5:8047775-8047795 5 15::21 27::34 23::38 41::67 0.967443684 1 A3SS

AT3G58640 chr3:21690776-21690781 7 6::202 18::230 210::261 245::256 0.96749793 1 A3SS

AT5G15700 chr5:5115721-5115823 19 0::81 0::114 41::28 63::55 0.967502316 1 IR

AT5G28350 chr5:10323327-10323337 9 58::9 59::14 54::70 49::73 0.967522334 1 A3SS

AT5G07370 chr5:2330497-2331227 1 192::75 190::72 36::321 47::376 0.967529822 1 IR

AT5G19950 chr5:6741312-6741318 6 5::127 0::170 101::197 118::248 0.967575509 1 A3SS

AT5G07630 chr5:2413182-2413193 11 13::13 9::19 42::57 51::63 0.96762953 1 A5SS

AT5G26770 chr5:9409770-9409961 1 26::7 26::12 8::29 7::28 0.967631954 1 IR

AT2G34410 chr2:14525451-14525522 17 35::52 67::16 27143::26219 21575::22546 0.967739986 1 A3SS

AT1G08125 chr1:2541103-2541116 5 12::74 24::71 35::90 42::103 0.967758237 1 A5SS

AT3G04610 chr3:1252841-1253018 2-3 144::18 143::23 20::181 22::180 0.967773198 1 Cassette

AT3G12915 chr3:4113988-4114050 3 0::42 0::33 25::23 21::32 0.967791977 1 IR

AT5G15860 chr5:5180719-5180724 2 11::157 7::106 104::178 72::106 0.967822459 1 A3SS

AT1G67690 chr1:25370471-25370477 5 7::7 11::17 5::14 10::22 0.967857795 1 A3SS

AT3G04640 chr3:1260949-1261034 1 0::134 0::125 74::113 70::104 0.967963842 1 IR

AT3G27460 chr3:10160670-10160679 4 8::17 10::6 17::35 10::39 0.967992413 1 A3SS

AT5G64460 chr5:25775306-25775354 3 209::23 202::27 19::221 30::207 0.967998843 1 Cassette

AT1G22850 chr1:8081025-8081201 6 2168::23 3017::19 68::2092 85::3026 0.968020678 1 A3SS

AT4G35080 chr4:16699801-16699889 3 4::1039 6::1243 556::429 673::483 0.968029319 1 IR

AT1G28570 chr1:10043048-10043263 2 4::245 0::180 110::112 96::89 0.968041775 1 Cassette

AT2G27060 chr2:11553696-11553768 2 15::300 10::272 156::102 150::124 0.968060695 1 IR

AT1G03780 chr1:948595-948640 14 46::0 16::0 5::38 3::25 0.968070382 1 A3SS

AT1G06150 chr1:1870003-1870009 9 78::62 135::103 78::165 128::260 0.9680983 1 A3SS

AT4G21850 chr4:11591533-11591638 2 742::10 1060::16 43::492 64::696 0.968143594 1 IR

AT3G59780 chr3:22086750-22086870 2 10::685 12::697 359::437 337::461 0.968145056 1 IR

AT2G21870 chr2:9322196-9322202 2 67::3558 49::3263 2878::2981 2621::2866 0.968338073 1 A3SS

AT5G67580 chr5:26956846-26956855 3 113::9 108::13 16::120 28::162 0.968360305 1 A3SS

AT4G20310 chr4:10963133-10963167 6 52::0 74::2 50::48 77::91 0.968360446 1 A3SS

AT1G29790 chr1:10431528-10431637 2 2::150 0::224 19::107 25::150 0.968384783 1 A3SS

AT1G10657 chr1:3531193-3531265 3 10::257 7::295 48::261 37::317 0.968390592 1 A3SS

AT2G41720 chr2:17406304-17406327 3 7::91 10::75 21::95 20::73 0.968434362 1 A5SS

AT5G14270 chr5:4604896-4605112 1 3::44 14::44 28::54 23::52 0.968440322 1 IR

AT5G51620 chr5:20967606-20967650 2 88::49 105::69 100::180 140::221 0.96844116 1 A5SS

AT4G19500 chr4:10627381-10627468 2 7::154 23::163 85::84 87::88 0.968528993 1 IR

AT3G55920 chr3:20744495-20744575 3-4 90::18 96::16 15::82 10::123 0.968576123 1 AltStart

AT1G13450 chr1:4613613-4613889 3 0::207 3::245 74::137 79::193 0.968580367 1 Cassette

AT5G01260 chr5:106476-106797 3 113::52 122::40 28::139 33::144 0.968703882 1 IR

AT1G07110 chr1:2181456-2181462 11 204::6 279::11 105::244 133::307 0.968720826 1 A3SS

AT2G47710 chr2:19555670-19555694 2 2624::43 2648::33 461::2600 471::2683 0.968788517 1 A3SS

AT3G09050 chr3:2765196-2765207 1 11::268 13::220 255::172 239::164 0.968805426 1 A5SS

AT1G63010 chr1:23348998-23349004 10 1::177 4::231 123::192 152::211 0.968818891 1 A3SS

AT2G01060 chr2:74585-74785 2 386::6 399::15 21::404 23::466 0.968821694 1 IR

AT3G56580 chr3:20962379-20962483 2 9::239 1::290 119::218 157::252 0.968858539 1 IR

AT3G01850 chr3:301025-301051 5 2::258 14::344 305::313 406::420 0.968870747 1 A3SS

AT1G25175 chr1:8828298-8828544 3 198::156 120::37 3::3 3::3 0.968935119 1 Cassette

AT3G17900 chr3:6130302-6130309 5 24::73 14::57 42::107 36::117 0.968938427 1 A3SS

AT3G58490 chr3:21633558-21633572 3 296::6 345::14 258::328 331::438 0.968945551 1 A3SS

AT1G70570 chr1:26610293-26610326 4 177::27 176::24 56::193 50::201 0.969025661 1 A5SS

AT1G19680 chr1:6808999-6809007 6 25::0 53::3 34::37 65::74 0.969029143 1 A3SS

AT5G05560 chr5:1653889-1654185 32-33 0::44 0::122 33::23 58::52 0.969049991 1 Cassette_multi

AT3G07810 chr3:2495039-2495086 4 265::19 325::38 268::117 336::139 0.969070475 1 A3SS

AT3G54350 chr3:20126442-20126468 3 15::59 18::42 65::89 43::63 0.96911958 1 A3SS

AT1G61140 chr1:22539750-22539777 3 3::17 5::16 12::24 11::17 0.969161387 1 A5SS

AT1G19394 chr1:6710115-6710468 3 30::14 21::2 31::27 30::23 0.969188793 1 A3SS

AT4G27040 chr4:13575081-13575095 3 75::6 65::10 47::135 42::135 0.969267703 1 A3SS

AT4G14520 chr4:8342198-8342238 3 44::55 48::38 37::105 26::107 0.969299269 1 Cassette_multi

AT3G01120 chr3:41227-41239 2 53::4822 48::4686 4141::4134 4005::4039 0.969343539 1 A3SS

AT2G38820 chr2:16222795-16222862 2 17::300 7::259 158::193 138::150 0.969362485 1 IR

AT3G27100 chr3:9994816-9994914 4 158::0 164::8 9::82 15::101 0.969401828 1 IR

AT5G52530 chr5:21317498-21317514 1 9::81 4::94 93::76 101::72 0.969416912 1 A5SS

AT4G35830 chr4:16977793-16977946 1 533::6 420::3 15::409 12::308 0.969447458 1 IR

AT5G12840 chr5:4053413-4053442 1 6::61 0::35 101::123 93::108 0.969447496 1 A5SS

AT1G28290 chr1:9890275-9890492 1 218::5003 220::5113 1696::1604 1728::1731 0.96945612 1 IR

AT3G60310 chr3:22288647-22288684 4 14::1 12::8 15::14 16::19 0.969456513 1 A3SS

AT5G17010 chr5:5591721-5591818 2 16::355 12::236 162::120 118::84 0.969509543 1 Cassette

AT2G31350 chr2:13369265-13369278 3 30::274 33::366 245::274 334::306 0.969615027 1 A5SS

AT5G35180 chr5:13427820-13427832 13 4::132 15::156 44::155 60::193 0.969655688 1 A3SS

AT3G55080 chr3:20412862-20412877 10 64::2 119::0 27::67 59::136 0.969742149 1 A3SS

AT4G30200 chr4:14790052-14790145 2 1::311 0::294 169::227 167::201 0.969745054 1 Cassette

AT5G57960 chr5:23466029-23466109 1-2 89::6 68::10 7::138 19::156 0.969814832 1 AltStart

AT4G18270 chr4:10100341-10100383 5 13::3 18::5 25::36 39::52 0.969822842 1 A5SS

AT1G35515 chr1:13078915-13078929 2 283::498 225::313 1119::516 1120::500 0.969864162 1 A5SS

AT2G27980 chr2:11918060-11918079 3 7::19 12::39 15::51 21::49 0.969905671 1 A5SS

AT4G22540 chr4:11862337-11862377 7 14::144 12::177 150::165 175::156 0.969913346 1 A3SS

AT1G02350 chr1:470347-470371 3 50::6 42::4 61::73 55::80 0.969915785 1 A3SS

AT3G63500 chr3:23449230-23449754 2 0::244 0::231 153::175 126::186 0.969977092 1 IR

AT3G06330 chr3:1919304-1919406 6 222::11 262::12 24::233 30::273 0.969986989 1 IR

AT1G58602 chr1:21764770-21764969 9 57::0 439::7 3::80 30::214 0.969990837 1 AltEnd

AT5G43990 chr5:17699285-17699345 5 15::15 6::18 19::39 21::25 0.969994119 1 A5SS

AT1G34315 chr1:12514659-12515037 5 19::0 45::0 4::27 5::59 0.970059015 1 Cassette_multi

AT5G40520 chr5:16233822-16233909 2 12::3 6::6 5::13 5::12 0.970067927 1 IR

AT2G45250 chr2:18662471-18662565 4 1::806 0::825 418::230 420::272 0.970088328 1 Cassette

AT1G24030 chr1:8504175-8505460 4 146::9 218::10 12::80 16::107 0.970141641 1 AltEnd

AT5G10480 chr5:3299056-3299082 6 957::0 724::0 6::921 11::673 0.970161018 1 Cassette

AT1G53310 chr1:19888088-19888229 2 14::7 5::12 15::282 16::201 0.970181095 1 IR

AT4G02210 chr4:973990-974086 6 144::14 240::23 13::98 20::139 0.970188983 1 IR

AT5G62890 chr5:25243538-25243695 1 475::2 308::7 19::288 15::212 0.970198256 1 IR

AT3G47680 chr3:17576463-17576755 3 16::54 22::121 67::80 112::173 0.970206724 1 Cassette

AT1G02750 chr1:603775-603784 4 98::0 84::0 76::117 59::125 0.970296927 1 A3SS

AT4G38230 chr4:17928764-17928771 12 6::13 2::11 24::41 60::81 0.970368642 1 A5SS

AT1G77600 chr1:29156671-29156698 18 17::0 27::0 3::20 3::25 0.970384376 1 A3SS

AT3G54480 chr3:20172403-20172481 3 50::861 52::998 444::498 518::524 0.970543006 1 Cassette

AT5G39660 chr5:15880779-15880988 2 0::122 0::137 53::268 61::235 0.970573377 1 IR

AT1G74970 chr1:28158042-28158048 2 126::3142 97::3011 2603::2961 2469::3055 0.970580876 1 A3SS

AT5G53220 chr5:21596236-21596711 2 22::1 10::6 5::26 8::25 0.970585518 1 Cassette

AT5G24360 chr5:8316829-8316877 2 23::0 33::0 3::25 3::27 0.970722687 1 Cassette

AT2G31900 chr2:13567566-13567583 10 10::9 10::2 13::18 11::17 0.970757073 1 A5SS

AT4G36960 chr4:17427062-17427235 1 49::39 50::37 23::52 20::61 0.970767354 1 IR

AT2G26430 chr2:11245261-11245268 3 0::236 4::170 135::237 115::218 0.97076741 1 A3SS

AT1G43670 chr1:16469169-16469218 6 1002::7 1142::17 1108::1163 1226::1285 0.970769085 1 A3SS

AT5G45410 chr5:18403289-18403303 3 6::606 8::453 492::677 341::541 0.97077257 1 A3SS

AT4G10610 chr4:6558988-6559083 8 695::38 812::69 52::306 71::394 0.970835039 1 IR

AT5G22460 chr5:7445286-7445295 2 49::5 28::0 35::105 18::67 0.970837704 1 A3SS

AT1G63940 chr1:23730104-23730195 1 89::315 84::255 177::286 128::245 0.970924691 1 IR

AT5G09220 chr5:2867523-2867528 4 2168::37 2033::40 503::2467 474::2221 0.970963429 1 A5SS

AT3G04970 chr3:1377476-1377486 7 4::20 20::48 14::32 30::72 0.970977142 1 A3SS

AT5G39785 chr5:15931879-15931885 3 10::0 23::0 8::18 11::25 0.971050686 1 A5SS

AT2G31530 chr2:13429361-13429366 9 40::1 77::16 26::80 36::117 0.971063074 1 A3SS

AT1G28960 chr1:10110022-10110162 4 129::184 165::187 109::146 100::153 0.971263251 1 IR

AT4G26210 chr4:13282354-13282359 2 325::3 385::4 296::341 357::436 0.97127317 1 A3SS

AT3G61080 chr3:22607823-22607832 4 22::316 17::252 108::316 93::258 0.97127986 1 A3SS

AT3G12390 chr3:3942658-3942742 2 11::2883 6::3415 1526::1409 1876::1562 0.971340354 1 Cassette

AT2G35920 chr2:15075930-15075958 2 6::103 12::100 17::98 24::87 0.971349447 1 A3SS

AT1G66880 chr1:24952952-24953677 5 9::9 16::21 35::19 48::19 0.971368067 1 Cassette

AT2G37860 chr2:15856726-15856942 1 517::59 495::55 34::418 32::442 0.971377083 1 IR

AT4G32140 chr4:15523988-15524005 4 126::20 137::28 52::143 62::179 0.971378792 1 A3SS

AT5G47970 chr5:19423558-19423563 4 82::5 75::10 48::88 46::90 0.971383624 1 A3SS

AT1G18840 chr1:6504071-6504081 1 43::74 33::34 54::18 38::12 0.971432331 1 A5SS

AT2G24390 chr2:10373994-10374000 2 204::5 177::2 105::179 96::182 0.971495208 1 A3SS

AT2G04840 chr2:1700185-1700202 3 289::52 233::46 137::406 106::337 0.971514786 1 A5SS

AT3G15353 chr3:5181276-5181282 1 55944::285 51717::76 43393::16165 39594::14040 0.971537178 1 A5SS

AT1G75420 chr1:28306149-28306247 3 26::110 18::135 58::83 79::94 0.971563697 1 IR

AT4G00560 chr4:240941-240953 6 280::4 434::3 91::166 135::259 0.97162926 1 A3SS

AT4G14400 chr4:8294661-8294743 2 0::149 3::250 70::96 133::142 0.971661367 1 Cassette

AT5G01030 chr5:10574-10620 2 24::2 18::2 34::65 30::76 0.971684571 1 A3SS

AT2G32090 chr2:13644627-13644722 2 811::12 695::5 17::451 10::453 0.971703412 1 AltStart

AT3G53190 chr3:19716456-19716469 5 16::416 10::437 103::627 98::700 0.971750805 1 A3SS

AT5G07950 chr5:2541404-2541414 4 79::11 77::20 27::106 30::107 0.971773051 1 A3SS

AT1G45110 chr1:17053120-17053128 4 8::10 13::41 9::32 21::57 0.971842556 1 A3SS

AT4G13940 chr4:8055242-8055378 1 0::27795 0::27948 16869::14374 16952::14205 0.9719343 1 IR

AT5G28020 chr5:10028486-10028524 2 29::16 22::7 10::517 5::714 0.971941834 1 AltStart

AT2G16060 chr2:6983117-6983133 3 4::364 25::856 352::432 827::1040 0.97199089 1 A3SS

AT3G12050 chr3:3839448-3839566 1 0::947 2::741 499::543 418::517 0.972003435 1 IR

AT3G51380 chr3:19074237-19074274 1 12::21 20::17 43::76 50::79 0.972068307 1 A5SS

AT1G55325 chr1:20640284-20640343 12 0::32 0::43 24::29 26::24 0.972085435 1 Cassette

AT3G02750 chr3:594363-594469 5 341::1 478::10 18::325 27::437 0.972139809 1 IR

AT5G11720 chr5:3779039-3779047 5 8::113 17::100 64::155 72::183 0.972145648 1 A3SS

AT5G02680 chr5:607592-607666 7 62::0 137::0 63::77 142::151 0.972231324 1 A3SS

AT1G58080 chr1:21504941-21504960 10 5::1082 11::1521 1094::1070 1608::1691 0.97223259 1 A3SS

AT2G45010 chr2:18568155-18568755 1 86::20 130::54 21::108 28::153 0.972259615 1 IR

AT2G36010 chr2:15120719-15120854 5 16::0 18::0 19::31 15::41 0.972265616 1 MXE

AT3G57230 chr3:21179929-21179988 5 7::145 6::166 18::131 19::143 0.972276206 1 A3SS

AT5G51230 chr5:20827584-20827599 16 118::0 128::6 97::140 107::144 0.972327704 1 A3SS

AT4G16420 chr4:9263889-9263901 9 25::56 35::47 66::131 59::139 0.972381873 1 A3SS

AT5G19170 chr5:6446865-6446878 7 141::5 155::10 149::120 156::135 0.972394797 1 A3SS

AT1G60525 chr1:22300267-22300662 2 33::44 34::48 17::62 25::92 0.972432508 1 IR

AT3G53370 chr3:19788248-19788362 2 0::179 6::193 244::154 236::167 0.972510544 1 A3SS

AT1G62130 chr1:22966388-22966431 11 23::0 12::0 5::27 3::17 0.972528642 1 A3SS

AT5G04090 chr5:1107061-1107276 3 71::34 100::73 16::120 29::156 0.972528742 1 IR

AT2G16790 chr2:7284804-7284810 3 1::79 10::91 90::71 101::87 0.972580296 1 A3SS

AT5G42530 chr5:17005057-17005105 2 3777::26 9012::28 495::2553 1100::6557 0.97258149 1 A3SS

AT4G36730 chr4:17310673-17310679 8 52::359 86::413 290::505 357::653 0.972583022 1 A3SS

AT3G17950 chr3:6146379-6146603 1 123::37 58::30 15::199 11::214 0.972601712 1 IR

AT3G26165 chr3:9571146-9571199 1 34::0 68::0 41::43 78::67 0.972603576 1 A5SS

AT4G17330 chr4:9690979-9690985 12 5::105 11::163 93::161 150::237 0.972617875 1 A3SS

AT2G28320 chr2:12095637-12095642 3 50::19 39::21 50::68 45::54 0.972618783 1 A5SS

AT5G07940 chr5:2536468-2536592 4 18::18 31::24 11::47 16::43 0.972634283 1 IR

AT1G15290 chr1:5258128-5258137 21 1::49 18::121 819::927 1072::1341 0.972660984 1 A3SS

AT4G31790 chr4:15377320-15377466 1 124::1 115::5 9::102 9::107 0.972702044 1 IR

AT2G14910 chr2:6407062-6407241 4 2495::2 2668::12 114::1616 124::1816 0.972722949 1 IR

AT3G02030 chr3:347661-347668 11 16::0 25::0 17::36 37::51 0.972762737 1 A3SS

AT4G18020 chr4:10006755-10006775 2 33::58 32::69 79::226 91::204 0.972790645 1 A3SS

AT5G37130 chr5:14683671-14683676 7 20::40 24::53 32::84 41::85 0.972812757 1 A3SS

AT2G41260 chr2:17206885-17207051 2 10::27 3::9 7::16 3::8 0.97282055 1 IR

AT3G19000 chr3:6553922-6554037 2 243::123 319::126 64::265 78::321 0.972826157 1 IR

AT1G56180 chr1:21026649-21026659 6 165::13 192::21 84::183 99::251 0.972834177 1 A3SS

AT5G27970 chr5:10014826-10014832 43 107::11 165::34 128::126 200::168 0.972888622 1 A3SS

AT3G21350 chr3:7517981-7518103 4 223::0 214::0 12::246 21::233 0.97290515 1 Cassette

AT1G52230 chr1:19455126-19455252 2 59::40804 0::37526 22553::13009 20277::13324 0.973048667 1 Cassette

AT4G23020 chr4:12067369-12067449 2 30::0 18::0 7::51 7::29 0.973059901 1 Cassette

AT2G21170 chr2:9071183-9071189 8 14::4558 10::5697 677::4270 850::5318 0.973059942 1 A5SS

AT1G16340 chr1:5590524-5590611 1 0::65 4::90 43::54 60::66 0.973064381 1 IR

AT2G16365 chr2:7074527-7074602 2 15::62 9::25 45::83 26::89 0.97311107 1 AltStart

AT5G67030 chr5:26754189-26754198 14 7::2143 13::2313 1915::2373 2095::2664 0.973133986 1 A3SS

AT3G06760 chr3:2133516-2133581 4 120::78 171::101 51::181 56::229 0.973151445 1 Cassette_multi

AT3G01930 chr3:319222-319311 3 18::259 18::318 152::142 174::190 0.973166298 1 IR

AT3G33530 chr3:14087188-14087211 2 80::0 43::0 11::62 21::73 0.973167327 1 A5SS

AT3G61240 chr3:22669320-22669332 10 424::3 513::6 442::149 539::206 0.97316962 1 A3SS

AT1G18660 chr1:6425414-6425506 18 282::4 362::5 10::177 12::233 0.973173583 1 IR

AT5G22640 chr5:7530756-7530763 3 12::289 4::235 269::243 218::200 0.973198073 1 A5SS

AT3G51620 chr3:19147873-19147887 9 60::42 79::73 66::49 95::79 0.973205715 1 A3SS

AT5G10480 chr5:3299873-3299880 2 0::908 3::636 1063::879 779::697 0.973219452 1 A3SS

AT3G55020 chr3:20391742-20391747 9 8::133 12::135 46::142 53::141 0.973224367 1 A5SS

AT5G35930 chr5:14069816-14069861 9 27::8 25::11 11::33 23::54 0.973260819 1 A3SS

AT1G60000 chr1:22094094-22094181 1-2 0::1547 0::1967 1378::1092 1750::1215 0.973268285 1 Cassette

AT1G70505 chr1:26571179-26571205 2 18::15 31::19 21::44 23::48 0.973312627 1 A5SS

AT5G10020 chr5:3134300-3134444 1 3::38 3::53 59::72 61::66 0.973329633 1 A5SS

AT5G59660 chr5:24037749-24037758 10 19::0 22::0 14::20 16::19 0.973395025 1 A3SS

AT4G38800 chr4:18113729-18113737 7 8::2202 10::2933 1326::2227 1745::3024 0.973419695 1 A3SS

AT2G36010 chr2:15120000-15120149 2 12::0 24::0 12::32 9::31 0.97342292 1 Cassette

AT5G43850 chr5:17628487-17628581 3 19::5389 2::3745 2577::3342 1853::2447 0.973434986 1 Cassette

AT5G48880 chr5:19813557-19813680 16 0::1363 3::1445 568::597 654::633 0.973449664 1 Cassette

AT3G61710 chr3:22840179-22840284 7 69::15 90::15 14::90 18::103 0.973482823 1 IR

AT5G40570 chr5:16247486-16247506 2 9::2 25::1 4::14 5::21 0.973494552 1 Cassette

AT2G20100 chr2:8680853-8681612 7 36::3 44::14 3::36 6::32 0.973527735 1 IR

AT1G04400 chr1:1188006-1188073 2 274::7 188::1 34::362 19::262 0.97372129 1 A3SS

AT2G35658 chr2:14990539-14990651 1 9::6 21::4 5::16 3::23 0.973724669 1 IR

AT1G76570 chr1:28729687-28729694 3 42::179 44::171 108::154 122::188 0.973745042 1 A3SS

AT3G60340 chr3:22304258-22304388 1 52::355 33::324 202::181 210::148 0.973754456 1 IR

AT1G04010 chr1:1034338-1034345 6 10::8 17::12 10::32 17::33 0.973848241 1 A3SS

AT3G04770 chr3:1309855-1309933 3 103::2 220::11 13::121 27::223 0.973870625 1 IR

AT2G17695 chr2:7684841-7684846 3 13::496 4::376 512::441 401::376 0.973881682 1 A3SS

AT2G42580 chr2:17730379-17730390 4 7::152 14::185 21::193 28::289 0.973962115 1 A3SS

AT5G20060 chr5:6776275-6776441 2 134::0 132::0 3::527 10::542 0.974041563 1 AltStart

AT2G01270 chr2:140195-140200 4 11::176 18::201 87::218 104::242 0.974057296 1 A3SS

AT2G27350 chr2:11701523-11702526 2 16::272 9::310 190::114 182::114 0.974069741 1 Cassette_multi

AT2G34840 chr2:14702776-14702869 1 36::5 33::7 5::34 3::27 0.974097589 1 IR

AT3G09940 chr3:3057877-3057901 5 0::32 1::64 31::22 66::66 0.974129614 1 A3SS

AT2G02960 chr2:863995-864248 1 18::104 42::202 44::116 81::180 0.974140571 1 IR

AT5G52240 chr5:21214215-21214232 2 1817::11 2107::16 1854::1209 2194::1474 0.974150959 1 A3SS

AT1G35250 chr1:12933348-12933359 4 6::137 12::102 147::171 126::130 0.974158681 1 A3SS

AT3G04440 chr3:1180171-1180254 2 19::13 6::12 3::3 3::4 0.974222614 1 A5SS

AT2G42810 chr2:17814558-17814719 6 292::66 259::53 37::288 46::249 0.974223539 1 Cassette

AT5G46560 chr5:18889221-18889236 6 16::83 21::97 37::94 48::130 0.97425825 1 A3SS

AT1G59750 chr1:21982989-21982995 12 49::284 49::293 316::277 338::310 0.974364709 1 A5SS

AT3G02700 chr3:580449-580480 3 492::8 436::7 571::623 635::754 0.974418804 1 A5SS

AT5G08440 chr5:2723609-2723622 10 49::7 59::8 17::25 19::22 0.97447057 1 A5SS

AT1G45545 chr1:17179610-17179615 3 2231::46 1907::43 1804::2623 1553::2102 0.974472348 1 A5SS

AT2G38880 chr2:16240219-16240308 5 269::9 255::3 19::115 17::123 0.974473527 1 IR

AT5G62880 chr5:25238700-25238713 7 228::18 320::23 243::283 331::369 0.974477023 1 A3SS

AT4G33950 chr4:16272526-16272534 1 10::57 3::39 41::45 26::45 0.974533266 1 A5SS

AT1G14180 chr1:4848189-4848194 3 50::2 40::6 27::67 22::75 0.974608167 1 A3SS

AT1G04750 chr1:1333237-1333351 1 876::0 898::0 1000::527 891::456 0.974609406 1 A5SS

AT4G07410 chr4:4201535-4201632 11 0::457 15::855 255::207 456::388 0.974684484 1 IR

AT5G09820 chr5:3056168-3056256 5 92::42 162::79 38::33 61::65 0.974721078 1 IR

AT1G27520 chr1:9560597-9560620 9 34::19 74::65 24::61 65::107 0.974721294 1 A5SS

AT3G04890 chr3:1348958-1348977 8 14::169 16::242 45::113 61::154 0.974778511 1 A3SS

AT1G02960 chr1:667437-667464 12 10::24 17::37 66::91 83::104 0.974890098 1 A3SS

AT1G06820 chr1:2094819-2094833 6 7::144 18::167 125::129 139::171 0.974957227 1 A3SS

AT2G13650 chr2:5689117-5689222 4-5 72::17 86::35 12::85 17::113 0.974962963 1 Cassette

AT1G22660 chr1:8021434-8021440 15 82::2 166::0 78::35 137::56 0.974977797 1 A3SS

AT1G49720 chr1:18402128-18402153 4 12::0 26::0 4::12 6::24 0.975000554 1 A5SS

AT4G25670 chr4:13086366-13086386 2 360::6 405::2 6::525 7::587 0.975007552 1 AltStart

AT4G33000 chr4:15925556-15925579 5 29::129 35::174 136::175 180::241 0.975083357 1 A5SS

AT5G23760 chr5:8013793-8013873 2-3 248::3 326::12 21::109 18::125 0.975104835 1 AltEnd

AT4G28980 chr4:14290116-14290439 2 160::32 193::53 16::187 24::198 0.975156772 1 IR

AT5G49230 chr5:19959467-19959473 4 16::160 11::152 117::247 128::264 0.975260883 1 A3SS

AT4G14360 chr4:8270315-8270320 2 49::57 37::46 63::157 50::144 0.975272388 1 A3SS

AT1G53710 chr1:20054629-20054634 8 23::9 39::8 25::29 40::45 0.975273704 1 A5SS

AT1G68540 chr1:25720830-25720912 3 2::151 1::202 84::74 119::81 0.975276161 1 IR

AT5G60590 chr5:24357945-24357957 4 17::64 22::95 38::106 60::146 0.975284811 1 A3SS

AT3G27120 chr3:9999850-9999941 12 13::154 14::150 201::153 216::156 0.975301462 1 A3SS

AT5G67610 chr5:26963335-26963371 2 44::2 82::2 10::61 13::73 0.975318251 1 A3SS

AT5G63870 chr5:25562913-25562924 2 89::12 95::8 33::141 32::117 0.975331825 1 A3SS

AT5G52110 chr5:21170642-21170681 8 2::310 2::351 260::261 313::310 0.975333493 1 A3SS

AT1G17850 chr1:6147084-6147100 7 80::5 114::19 47::150 85::196 0.975342267 1 A3SS

AT5G57790 chr5:23412239-23412250 4 21::3 18::3 16::13 18::12 0.975350921 1 A3SS

AT1G58080 chr1:21505852-21505879 5 81::923 114::1080 177::835 212::943 0.97537405 1 A5SS

AT1G14700 chr1:5059726-5059732 3 501::16 350::10 551::483 374::375 0.975396934 1 A3SS

AT5G57290 chr5:23207168-23207213 2 27::9337 1::9936 8617::8622 9227::8945 0.975423619 1 Cassette

AT2G15910 chr2:6934205-6934503 3 0::495 0::618 313::114 344::149 0.975456826 1 AltEnd

AT1G33410 chr1:12117813-12117825 4 30::7 33::10 18::51 21::54 0.975508983 1 A3SS

AT4G14240 chr4:8207027-8207054 1 100::0 94::0 68::73 80::81 0.975568498 1 A5SS

AT3G21260 chr3:7465019-7465094 3 16::7 47::6 11::163 16::197 0.975581137 1 AltStart

AT5G09410 chr5:2923508-2923572 9 0::110 2::92 70::74 76::89 0.975719565 1 Cassette

AT5G23510 chr5:7928947-7929061 10 51::19 90::29 9::54 19::86 0.975732647 1 IR

AT2G15490 chr2:6762926-6762935 2 40::0 33::0 27::38 26::49 0.975734287 1 A3SS

AT3G47000 chr3:17315779-17315787 3 13::259 10::154 214::243 143::157 0.975764129 1 A3SS

AT3G03770 chr3:948736-948795 2 0::52 1::19 77::83 45::62 0.975773784 1 A3SS

AT4G04040 chr4:1941341-1941378 9 342::10 438::6 315::360 418::479 0.975783321 1 A3SS

AT5G42760 chr5:17150057-17150257 2 0::438 0::549 226::253 313::287 0.975794071 1 IR

AT3G56720 chr3:21013087-21013118 7 10::90 22::105 31::45 51::79 0.975826826 1 A3SS

AT3G03350 chr3:792698-792733 4 21::0 24::0 10::38 15::42 0.975836831 1 Cassette

AT3G47550 chr3:17525153-17525160 8 209::6 274::2 119::148 163::180 0.975854193 1 A3SS

AT3G14570 chr3:4898590-4898669 27 0::36 0::24 19::20 12::14 0.975865415 1 IR

AT5G35790 chr5:13957622-13957629 7 15::508 8::478 391::815 371::850 0.975903283 1 A3SS

AT3G13180 chr3:4238500-4238510 7 90::31 97::26 65::173 71::186 0.975925591 1 A3SS

AT1G01448 chr1:165656-165769 5 9::46 20::105 28::41 65::73 0.975959102 1 IR

AT2G25510 chr2:10856754-10856760 2 2076::1 5488::7 1025::1802 2729::4910 0.975997807 1 A3SS

AT3G09050 chr3:2765322-2765402 1-2 268::11 220::15 13::189 17::198 0.97602974 1 AltEnd

AT4G28300 chr4:14015015-14015396 1 300::57 182::43 40::297 29::162 0.976035601 1 IR
[truncated: 422,519 more chars]
