# Supplementary material for: Depletion of Arabidopsis SC35 and SC35-like serine/arginine-rich proteins affects the transcription and splicing of a subset of genes
Source: PLoS Genet. 2017 Mar 8;13(3):e1006663. doi: 10.1371/journal.pgen.1006663 (PMC5362245; doi:10.1371/journal.pgen.1006663)
Supplement: S4 Table — The alternatively spliced genes (213 (p<0.05)) overlapped to the differentially expressed genes (1249). Data were analyzed using the formula VLOOKUP. (DOCX) [file pgen.1006663.s020.docx]

**Table S3 Alternatively spliced genes overlap to the differentially expressed genes**

| AccID | alternative splicing | #N/A |
| --- | --- | --- |
| AT2G11410 | AT5G36170 | #N/A |
| AT1G53480 | AT2G38870 | #N/A |
| AT2G42540 | AT5G64170 | #N/A |
| AT1G53490 | AT4G16510 | #N/A |
| AT5G52300 | AT1G08520 | #N/A |
| AT4G08093 | AT3G29130 | #N/A |
| AT3G27860 | AT3G53830 | #N/A |
| AT2G37870 | AT5G13800 | #N/A |
| AT1G60190 | AT1G02840 | #N/A |
| AT3G14440 | AT2G43010 | #N/A |
| AT5G35777 | AT4G34265 | #N/A |
| AT2G16367 | AT5G36170 | AT1G55310 |
| AT3G48360 | AT1G55310 | #N/A |
| AT2G40000 | AT2G04378 | #N/A |
| AT3G27250 | AT1G07820 | #N/A |
| AT5G59320 | AT4G33770 | #N/A |
| AT5G56550 | AT3G51420 | #N/A |
| AT3G55980 | AT4G35770 | #N/A |
| AT4G33550 | AT3G10420 | #N/A |
| AT2G39800 | AT3G22121 | #N/A |
| AT4G11280 | AT2G04378 | #N/A |
| AT1G80440 | AT5G56140 | #N/A |
| AT1G23390 | AT1G04830 | #N/A |
| AT2G20670 | AT5G62575 | #N/A |
| AT1G02205 | AT5G11170 | #N/A |
| AT2G44130 | AT5G18255 | #N/A |
| AT2G41190 | AT1G51110 | #N/A |
| AT3G46600 | AT4G13500 | #N/A |
| AT3G15450 | AT5G14550 | #N/A |
| AT5G35190 | AT4G08390 | #N/A |
| AT5G21940 | AT3G19720 | #N/A |
| AT4G12480 | AT5G58800 | #N/A |
| AT2G16060 | AT2G26980 | #N/A |
| AT3G05730 | AT2G39730 | #N/A |
| AT1G65190 | AT1G07110 | AT1G73480 |
| AT4G02970 | AT1G73480 | #N/A |
| AT5G64200 | AT1G19350 | #N/A |
| AT2G21640 | AT5G66570 | #N/A |
| AT3G55460 | AT5G53860 | #N/A |
| AT3G16670 | AT1G11840 | #N/A |
| AT3G13570 | AT3G05160 | #N/A |
| AT2G01422 | AT3G49645 | #N/A |
| AT5G09570 | AT1G34470 | #N/A |
| AT5G51190 | AT5G37055 | #N/A |
| AT2G38470 | AT2G20890 | #N/A |
| AT5G15500 | AT1G55130 | #N/A |
| AT2G11405 | AT5G60390 | #N/A |
| AT5G13170 | AT3G18500 | #N/A |
| AT2G25900 | AT1G69460 | #N/A |
| AT3G06435 | AT3G47460 | #N/A |
| AT4G12490 | AT1G18735 | #N/A |
| AT1G56660 | AT4G08280 | #N/A |
| AT5G02020 | AT2G18440 | #N/A |
| AT3G50480 | AT2G42500 | #N/A |
| AT1G72910 | AT1G69530 | #N/A |
| AT1G55310 | AT1G50000 | #N/A |
| AT3G19620 | AT1G64430 | #N/A |
| AT3G18773 | AT5G18260 | #N/A |
| AT5G24850 | AT3G49290 | #N/A |
| AT4G39675 | AT1G19120 | #N/A |
| AT5G37260 | AT1G37130 | #N/A |
| AT5G63160 | AT1G23360 | #N/A |
| AT3G28220 | AT5G14550 | #N/A |
| AT2G18700 | AT2G28550 | #N/A |
| AT5G52310 | AT3G52340 | #N/A |
| AT1G18710 | AT4G22670 | #N/A |
| AT2G11880 | AT3G13470 | #N/A |
| AT1G67105 | AT4G00710 | #N/A |
| AT3G10020 | AT2G04690 | #N/A |
| AT5G49080 | AT4G09760 | #N/A |
| AT1G60590 | AT3G63340 | #N/A |
| AT3G07350 | AT1G78790 | #N/A |
| AT2G30770 | AT4G38330 | #N/A |
| AT5G47450 | AT5G20510 | #N/A |
| AT1G72920 | AT1G26670 | #N/A |
| AT3G26510 | AT1G52220 | #N/A |
| AT4G33720 | AT5G58100 | #N/A |
| AT5G59220 | AT5G54270 | #N/A |
| AT4G02280 | AT3G52050 | #N/A |
| AT5G24150 | AT5G23020 | #N/A |
| AT5G17890 | AT5G27390 | #N/A |
| AT3G16530 | AT3G24520 | AT4G02725 |
| AT2G01008 | AT4G02725 | #N/A |
| AT2G43140 | AT3G05160 | #N/A |
| AT1G80840 | AT1G76280 | #N/A |
| AT1G27020 | AT1G28395 | AT4G34710 |
| AT1G69480 | AT4G34710 | #N/A |
| AT3G26740 | AT3G28130 | #N/A |
| AT4G27280 | AT5G51960 | #N/A |
| AT5G60250 | AT1G14710 | #N/A |
| AT2G40330 | AT4G05320 | #N/A |
| AT3G01420 | AT2G45070 | #N/A |
| AT5G57560 | AT5G05435 | #N/A |
| AT1G69490 | AT3G53890 | #N/A |
| AT3G44450 | AT1G12370 | #N/A |
| AT2G01860 | AT5G05270 | #N/A |
| AT1G52000 | AT2G45140 | #N/A |
| AT2G15090 | AT4G31810 | #N/A |
| AT1G16090 | AT1G05230 | #N/A |
| AT5G09930 | AT4G08460 | #N/A |
| AT4G36040 | AT3G66654 | AT1G05900 |
| AT2G27830 | AT1G05900 | #N/A |
| AT5G17300 | AT4G18440 | #N/A |
| AT3G54580 | AT4G05320 | #N/A |
| AT1G13260 | AT2G46915 | #N/A |
| AT5G66400 | AT5G26110 | #N/A |
| AT4G37610 | AT3G26570 | #N/A |
| AT5G61600 | AT4G31720 | #N/A |
| AT1G72360 | AT5G04430 | #N/A |
| AT1G69260 | AT5G52030 | #N/A |
| AT3G21720 | AT3G61420 | #N/A |
| AT1G77120 | AT4G38520 | #N/A |
| AT4G04760 | AT2G43235 | #N/A |
| AT1G26390 | AT3G56160 | #N/A |
| AT2G44180 | AT5G55580 | #N/A |
| AT1G31290 | AT1G02840 | #N/A |
| AT2G42530 | AT2G21330 | #N/A |
| AT1G62440 | AT1G73490 | #N/A |
| AT2G30766 | AT3G56210 | #N/A |
| AT1G54040 | AT2G19480 | AT4G13495 |
| AT1G75750 | AT4G13495 | #N/A |
| AT2G33380 | AT2G39760 | AT3G26510 |
| AT5G05440 | AT3G26510 | #N/A |
| AT2G39510 | AT1G14710 | #N/A |
| AT2G38310 | AT4G33770 | #N/A |
| AT3G54590 | AT1G54390 | #N/A |
| AT2G19590 | AT1G31160 | #N/A |
| AT1G49450 | AT3G17300 | #N/A |
| AT3G12580 | AT3G08940 | #N/A |
| AT4G21650 | AT4G27040 | #N/A |
| AT5G46900 | AT2G05100 | #N/A |
| AT1G79520 | AT5G55896 | #N/A |
| AT1G11440 | AT3G01500 | #N/A |
| AT1G18870 | AT3G15770 | #N/A |
| AT5G15970 | AT5G21105 | #N/A |
| AT4G12550 | AT5G01650 | #N/A |
| AT1G49860 | AT5G41670 | #N/A |
| AT2G17850 | AT4G19095 | #N/A |
| AT2G18660 | AT3G26900 | #N/A |
| AT5G57760 | AT3G09600 | #N/A |
| AT1G15380 | AT1G73650 | #N/A |
| AT5G62020 | AT1G07119 | #N/A |
| AT3G56880 | AT2G30860 | #N/A |
| AT5G35940 | AT5G02500 | AT3G55460 |
| AT1G33960 | AT3G55460 | #N/A |
| AT3G53980 | AT2G39780 | #N/A |
| AT3G59940 | AT1G55690 | #N/A |
| AT4G03230 | AT3G17950 | #N/A |
| AT5G01520 | AT3G13480 | #N/A |
| AT3G28550 | AT3G01690 | #N/A |
| AT1G73480 | AT3G26020 | AT3G28950 |
| AT2G33790 | AT3G28950 | #N/A |
| AT3G16660 | AT5G26180 | #N/A |
| AT2G32800 | AT5G09225 | #N/A |
| AT5G04120 | AT4G03415 | #N/A |
| AT5G25280 | AT3G18890 | #N/A |
| AT3G30775 | AT1G29040 | #N/A |
| AT1G27730 | AT3G10230 | #N/A |
| AT1G65970 | AT1G79600 | #N/A |
| AT1G70290 | AT4G24800 | #N/A |
| AT3G62680 | AT2G06025 | #N/A |
| AT1G26800 | AT5G53850 | AT3G17609 |
| AT5G59310 | AT3G17609 | #N/A |
| AT1G13609 | AT1G36160 | #N/A |
| AT3G09940 | AT1G70490 | AT5G17300 |
| AT5G46890 | AT5G17300 | #N/A |
| AT3G62070 | AT5G53048 | #N/A |
| AT3G24460 | AT5G53048 | #N/A |
| AT3G15310 | AT2G05310 | #N/A |
| AT1G13430 | AT1G49590 | #N/A |
| AT1G53830 | AT4G26650 | #N/A |
| AT2G41730 | AT1G23310 | #N/A |
| AT1G68570 | AT3G61420 | #N/A |
| AT5G64190 | AT1G07010 | #N/A |
| AT1G36180 | AT1G36390 | #N/A |
| AT4G21870 | AT1G02305 | #N/A |
| AT1G23720 | AT3G16230 | #N/A |
| AT5G05640 | AT1G19396 | #N/A |
| AT4G19060 | AT1G01790 | #N/A |
| AT3G56360 | AT3G45050 | #N/A |
| AT5G02590 | AT2G43160 | #N/A |
| AT5G66270 | AT1G69252 | #N/A |
| AT5G42060 | AT5G38480 | #N/A |
| AT3G44900 | AT1G80940 | #N/A |
| AT3G49970 | AT4G14385 | #N/A |
| AT4G07510 | AT1G19350 | #N/A |
| AT1G19530 | AT1G20810 | #N/A |
| AT2G41090 | AT5G01600 | #N/A |
| AT5G55450 | AT2G35500 | #N/A |
| AT1G75335 | AT4G00420 | #N/A |
| AT1G12040 | AT4G13940 | #N/A |
| AT4G29990 | AT2G21240 | #N/A |
| AT5G66985 | AT1G47530 | #N/A |
| AT2G18328 | AT2G02390 | #N/A |
| AT4G01950 | AT4G20850 | #N/A |
| AT5G14920 | AT1G66980 | #N/A |
| AT2G34390 | AT3G46130 | #N/A |
| AT3G04720 | AT1G47490 | #N/A |
| AT5G19120 | AT3G13920 | #N/A |
| AT1G49570 | AT4G04880 | #N/A |
| AT4G19100 | AT3G61420 | #N/A |
| AT4G31870 | AT5G12240 | #N/A |
| AT4G10270 | AT1G76970 | #N/A |
| AT4G15910 | AT3G28080 | #N/A |
| AT4G19680 | AT5G53850 | #N/A |
| AT5G43840 | AT3G25680 | #N/A |
| AT4G08410 | AT3G49430 | #N/A |
| AT5G23750 | AT3G54790 | AT1G33230 |
| AT1G69760 | AT1G33230 | #N/A |
| AT2G39681 | AT2G34410 | #N/A |
| AT3G29970 | AT4G35785 | #N/A |
| AT3G10340 | AT1G72510 | #N/A |
| AT1G54970 |  | #N/A |
| AT1G21320 |  | #N/A |
| AT4G20820 |  | #N/A |
| AT3G03270 |  | #N/A |
| AT1G36370 |  | #N/A |
| AT5G52780 |  | #N/A |
| AT1G02820 |  | #N/A |
| AT5G42530 |  | #N/A |
| AT5G55930 |  | #N/A |
| AT2G47770 |  | #N/A |
| AT4G11460 |  | #N/A |
| AT1G49210 |  | #N/A |
| AT3G26200 |  | #N/A |
| AT5G64120 |  | #N/A |
| AT2G25200 |  | #N/A |
| AT2G42610 |  | #N/A |
| AT1G02660 |  | #N/A |
| AT4G05070 |  | #N/A |
| AT2G22470 |  | #N/A |
| AT1G32920 |  | #N/A |
| AT3G09220 |  | #N/A |
| AT2G23000 |  | #N/A |
| AT1G06100 |  | #N/A |
| AT5G28300 |  | #N/A |
| AT1G18400 |  | #N/A |
| AT5G06640 |  | #N/A |
| AT3G42806 |  | #N/A |
| AT1G14200 |  | #N/A |
| AT5G03190 |  | #N/A |
| AT3G28270 |  | #N/A |
| AT1G28660 |  | #N/A |
| AT2G47800 |  | #N/A |
| AT4G21440 |  | #N/A |
| AT2G21660 |  | #N/A |
| AT4G21930 |  | #N/A |
| AT3G13277 |  | #N/A |
| AT4G34250 |  | #N/A |
| AT4G24700 |  | #N/A |
| AT5G66650 |  | #N/A |
| AT2G46680 |  | #N/A |
| AT5G10040 |  | #N/A |
| AT4G33560 |  | #N/A |
| AT5G02502 |  | #N/A |
| AT4G13390 |  | #N/A |
| AT1G62510 |  | #N/A |
| AT2G47520 |  | #N/A |
| AT1G20430 |  | #N/A |
| AT2G29630 |  | #N/A |
| AT4G13310 |  | #N/A |
| AT2G46390 |  | #N/A |
| AT1G21250 |  | #N/A |
| AT1G58360 |  | #N/A |
| AT2G16650 |  | #N/A |
| AT2G04032 |  | #N/A |
| AT3G22235 |  | #N/A |
| AT5G25610 |  | #N/A |
| AT2G20750 |  | #N/A |
| AT1G49390 |  | #N/A |
| AT1G07135 |  | #N/A |
| AT2G28470 |  | #N/A |
| AT3G28345 |  | #N/A |
| AT2G44230 |  | #N/A |
| AT5G05400 |  | #N/A |
| AT3G62550 |  | #N/A |
| AT3G45140 |  | #N/A |
| AT1G15405 |  | #N/A |
| AT2G05510 |  | #N/A |
| AT5G45630 |  | #N/A |
| AT3G23800 |  | #N/A |
| AT4G28800 |  | #N/A |
| AT2G01023 |  | #N/A |
| AT4G29180 |  | #N/A |
| AT5G41020 |  | #N/A |
| AT2G43050 |  | #N/A |
| AT4G31351 |  | #N/A |
| AT5G65207 |  | #N/A |
| AT5G40800 |  | #N/A |
| AT4G02380 |  | #N/A |
| AT2G43920 |  | #N/A |
| AT4G36648 |  | #N/A |
| AT4G39260 |  | #N/A |
| AT2G40140 |  | #N/A |
| AT1G07430 |  | #N/A |
| AT4G02520 |  | #N/A |
| AT3G49320 |  | #N/A |
| AT3G56080 |  | #N/A |
| AT1G43800 |  | #N/A |
| AT5G50915 |  | #N/A |
| AT1G15125 |  | #N/A |
| AT2G27775 |  | #N/A |
| ATCG00170 |  | #N/A |
| AT2G22100 |  | #N/A |
| AT3G50840 |  | #N/A |
| AT3G62700 |  | #N/A |
| AT5G18700 |  | #N/A |
| AT3G11410 |  | #N/A |
| AT2G24850 |  | #N/A |
| AT1G65500 |  | #N/A |
| AT5G17270 |  | #N/A |
| AT3G05370 |  | #N/A |
| AT1G21460 |  | #N/A |
| AT1G13820 |  | #N/A |
| AT3G50060 |  | #N/A |
| AT4G36570 |  | #N/A |
| AT2G22200 |  | #N/A |
| AT2G31230 |  | #N/A |
| AT2G27385 |  | #N/A |
| AT3G01790 |  | #N/A |
| AT3G28950 |  | #N/A |
| AT4G18550 |  | #N/A |
| AT2G24980 |  | #N/A |
| AT5G13630 |  | #N/A |
| AT4G17250 |  | #N/A |
| AT1G18810 |  | #N/A |
| AT5G22320 |  | #N/A |
| AT4G15210 |  | #N/A |
| AT3G22910 |  | #N/A |
| AT2G40765 |  | #N/A |
| AT2G19900 |  | #N/A |
| AT4G08400 |  | #N/A |
| AT2G41150 |  | #N/A |
| AT1G06160 |  | #N/A |
| AT5G24200 |  | #N/A |
| AT2G27400 |  | #N/A |
| AT1G19050 |  | #N/A |
| AT4G38840 |  | #N/A |
| AT1G79770 |  | #N/A |
| AT1G59960 |  | #N/A |
| AT2G17880 |  | #N/A |
| AT1G65450 |  | #N/A |
| AT3G26210 |  | #N/A |
| AT4G29780 |  | #N/A |
| AT2G43510 |  | #N/A |
| AT3G01820 |  | #N/A |
| AT1G48090 |  | #N/A |
| AT3G43510 |  | #N/A |
| ATMG00030 |  | #N/A |
| AT3G55710 |  | #N/A |
| AT5G47230 |  | #N/A |
| AT5G06320 |  | #N/A |
| AT2G46440 |  | #N/A |
| AT5G26800 |  | #N/A |
| AT5G65610 |  | #N/A |
| AT3G58980 |  | #N/A |
| AT4G33070 |  | #N/A |
| AT5G60680 |  | #N/A |
| AT1G35240 |  | #N/A |
| AT2G32487 |  | #N/A |
| AT5G46330 |  | #N/A |
| AT5G55970 |  | #N/A |
| AT3G45160 |  | #N/A |
| AT2G39310 |  | #N/A |
| AT1G14640 |  | #N/A |
| AT4G13575 |  | #N/A |
| AT2G26440 |  | #N/A |
| AT2G32220 |  | #N/A |
| AT3G18170 |  | #N/A |
| AT5G03350 |  | #N/A |
| AT1G58590 |  | #N/A |
| AT1G75580 |  | #N/A |
| AT1G64490 |  | #N/A |
| AT1G19510 |  | #N/A |
| AT4G15393 |  | #N/A |
| AT3G25670 |  | #N/A |
| AT4G31354 |  | #N/A |
| AT2G05380 |  | #N/A |
| AT5G09820 |  | #N/A |
| AT3G16770 |  | #N/A |
| AT4G12735 |  | #N/A |
| AT4G13280 |  | #N/A |
| AT1G51140 |  | #N/A |
| AT5G38340 |  | #N/A |
| AT5G54880 |  | #N/A |
| AT1G21520 |  | #N/A |
| AT3G44990 |  | #N/A |
| AT2G14510 |  | #N/A |
| AT2G43590 |  | #N/A |
| AT4G19550 |  | #N/A |
| AT3G21500 |  | #N/A |
| AT3G45680 |  | #N/A |
| AT1G05340 |  | #N/A |
| AT5G10140 |  | #N/A |
| AT1G32960 |  | #N/A |
| AT1G02340 |  | #N/A |
| AT2G20520 |  | #N/A |
| AT1G72940 |  | #N/A |
| AT4G10910 |  | #N/A |
| AT1G24530 |  | #N/A |
| AT1G75830 |  | #N/A |
| AT5G45950 |  | #N/A |
| AT3G21040 |  | #N/A |
| AT5G15960 |  | #N/A |
| AT2G03760 |  | #N/A |
| AT1G56600 |  | #N/A |
| AT3G07860 |  | #N/A |
| AT1G22890 |  | #N/A |
| AT3G20380 |  | #N/A |
| AT4G31290 |  | #N/A |
| AT3G13224 |  | #N/A |
| AT1G24260 |  | #N/A |
| AT1G60660 |  | #N/A |
| AT1G64660 |  | #N/A |
| AT1G73500 |  | #N/A |
| AT4G13900 |  | #N/A |
| AT4G27520 |  | #N/A |
| AT1G32460 |  | #N/A |
| AT2G28780 |  | #N/A |
| AT5G21020 |  | #N/A |
| AT5G45340 |  | #N/A |
| AT2G18390 |  | #N/A |
| AT3G15357 |  | #N/A |
| AT1G71400 |  | #N/A |
| AT3G04210 |  | #N/A |
| AT5G19890 |  | #N/A |
| AT2G32300 |  | #N/A |
| AT4G10600 |  | #N/A |
| AT3G43190 |  | #N/A |
| AT1G72140 |  | #N/A |
| AT3G44970 |  | #N/A |
| AT1G03410 |  | #N/A |
| AT3G49570 |  | #N/A |
| AT1G06380 |  | #N/A |
| AT2G19810 |  | #N/A |
| AT4G11521 |  | #N/A |
| AT4G16500 |  | #N/A |
| AT4G34150 |  | #N/A |
| AT3G58850 |  | #N/A |
| AT1G58400 |  | #N/A |
| AT2G27402 |  | #N/A |
| AT5G24140 |  | #N/A |
| AT1G02390 |  | #N/A |
| AT1G68650 |  | #N/A |
| AT5G60670 |  | #N/A |
| AT4G03180 |  | #N/A |
| AT1G03060 |  | #N/A |
| AT3G23830 |  | #N/A |
| AT4G31877 |  | #N/A |
| AT3G13940 |  | #N/A |
| AT5G42200 |  | #N/A |
| AT1G04700 |  | #N/A |
| AT2G43520 |  | #N/A |
| AT2G35950 |  | #N/A |
| AT2G19800 |  | #N/A |
| AT4G31398 |  | #N/A |
| AT3G54880 |  | #N/A |
| AT3G05727 |  | #N/A |
| AT1G24265 |  | #N/A |
| AT5G10800 |  | #N/A |
| AT1G76960 |  | #N/A |
| AT5G09970 |  | #N/A |
| AT1G18570 |  | #N/A |
| AT3G60330 |  | #N/A |
| AT1G26380 |  | #N/A |
| AT3G51600 |  | #N/A |
| AT5G55400 |  | #N/A |
| AT3G56070 |  | #N/A |
| AT5G43720 |  | #N/A |
| AT4G28780 |  | #N/A |
| AT1G34510 |  | #N/A |
| AT2G01870 |  | #N/A |
| AT1G31690 |  | #N/A |
| AT5G27830 |  | #N/A |
| AT5G37300 |  | #N/A |
| AT4G24570 |  | #N/A |
| AT5G15310 |  | #N/A |
| AT5G58240 |  | #N/A |
| AT3G10185 |  | #N/A |
| AT3G21340 |  | #N/A |
| AT4G12520 |  | #N/A |
| AT3G20470 |  | #N/A |
| AT2G07709 |  | #N/A |
| AT5G02760 |  | #N/A |
| AT1G08580 |  | #N/A |
| AT3G59140 |  | #N/A |
| AT3G46560 |  | #N/A |
| AT4G37520 |  | #N/A |
| AT3G53230 |  | #N/A |
| AT2G43120 |  | #N/A |
| AT4G27380 |  | #N/A |
| AT5G44580 |  | #N/A |
| AT1G70320 |  | #N/A |
| AT1G11700 |  | #N/A |
| AT5G18670 |  | #N/A |
| AT5G42110 |  | #N/A |
| AT4G34710 |  | #N/A |
| AT3G20395 |  | #N/A |
| AT2G43800 |  | #N/A |
| AT5G10930 |  | #N/A |
| AT5G22250 |  | #N/A |
| AT4G02725 |  | #N/A |
| AT1G04900 |  | #N/A |
| AT2G27840 |  | #N/A |
| AT5G54190 |  | #N/A |
| AT1G66940 |  | #N/A |
| AT1G51860 |  | #N/A |
| AT3G48460 |  | #N/A |
| AT1G23205 |  | #N/A |
| ATCG00180 |  | #N/A |
| AT2G41210 |  | #N/A |
| AT1G70460 |  | #N/A |
| AT2G25000 |  | #N/A |
| AT2G07671 |  | #N/A |
| AT3G46700 |  | #N/A |
| AT2G29090 |  | #N/A |
| AT3G44205 |  | #N/A |
| AT4G25630 |  | #N/A |
| AT3G57730 |  | #N/A |
| AT3G02260 |  | #N/A |
| AT1G72750 |  | #N/A |
| AT5G38720 |  | #N/A |
| AT4G39800 |  | #N/A |
| AT5G43370 |  | #N/A |
| AT1G51080 |  | #N/A |
| AT1G78820 |  | #N/A |
| AT1G66390 |  | #N/A |
| AT3G01260 |  | #N/A |
| AT2G25210 |  | #N/A |
| AT1G18730 |  | #N/A |
| AT2G19970 |  | #N/A |
| AT5G48412 |  | #N/A |
| AT3G09450 |  | #N/A |
| AT4G27950 |  | #N/A |
| AT1G06830 |  | #N/A |
| AT3G47720 |  | #N/A |
| AT2G35420 |  | #N/A |
| AT3G25717 |  | #N/A |
| AT3G10710 |  | #N/A |
| AT3G06320 |  | #N/A |
| AT5G38780 |  | #N/A |
| ATMG01080 |  | #N/A |
| AT4G37070 |  | #N/A |
| AT2G44670 |  | #N/A |
| AT1G66660 |  | #N/A |
| AT1G12805 |  | #N/A |
| AT4G12510 |  | #N/A |
| AT3G05640 |  | #N/A |
| AT3G28900 |  | #N/A |
| AT2G22122 |  | #N/A |
| AT5G15230 |  | #N/A |
| AT2G26190 |  | #N/A |
| AT3G26483 |  | #N/A |
| AT4G30930 |  | #N/A |
| AT3G32030 |  | #N/A |
| AT5G05340 |  | #N/A |
| AT4G15230 |  | #N/A |
| AT4G32480 |  | #N/A |
| AT2G33480 |  | #N/A |
| AT4G37220 |  | #N/A |
| AT1G65570 |  | #N/A |
| AT2G39855 |  | #N/A |
| AT2G15970 |  | #N/A |
| AT3G10570 |  | #N/A |
| AT2G20800 |  | #N/A |
| AT1G31200 |  | #N/A |
| AT2G31490 |  | #N/A |
| AT2G44110 |  | #N/A |
| AT1G05135 |  | #N/A |
| AT1G58370 |  | #N/A |
| AT1G05900 |  | #N/A |
| AT4G11210 |  | #N/A |
| AT1G52240 |  | #N/A |
| AT1G23090 |  | #N/A |
| AT2G14900 |  | #N/A |
| AT2G14878 |  | #N/A |
| AT5G20790 |  | #N/A |
| AT1G10990 |  | #N/A |
| AT2G21140 |  | #N/A |
| AT5G47640 |  | #N/A |
| AT2G33060 |  | #N/A |
| AT5G49560 |  | #N/A |
| AT5G61220 |  | #N/A |
| AT3G22231 |  | #N/A |
| AT4G31790 |  | #N/A |
| AT1G68238 |  | #N/A |
| AT3G08520 |  | #N/A |
| AT5G24770 |  | #N/A |
| AT5G23940 |  | #N/A |
| AT2G47690 |  | #N/A |
| AT1G66930 |  | #N/A |
| AT1G62380 |  | #N/A |
| AT3G51895 |  | #N/A |
| AT3G48350 |  | #N/A |
| AT2G47460 |  | #N/A |
| AT2G10410 |  | #N/A |
| AT3G21010 |  | #N/A |
| AT4G25250 |  | #N/A |
| AT1G09090 |  | #N/A |
| AT4G22666 |  | #N/A |
| AT1G14250 |  | #N/A |
| AT1G55010 |  | #N/A |
| AT3G21150 |  | #N/A |
| AT3G44590 |  | #N/A |
| AT5G28510 |  | #N/A |
| AT1G10640 |  | #N/A |
| AT2G34690 |  | #N/A |
| AT5G20160 |  | #N/A |
| ATCG00630 |  | #N/A |
| AT5G42600 |  | #N/A |
| AT2G17930 |  | #N/A |
| AT5G40380 |  | #N/A |
| AT3G45700 |  | #N/A |
| AT4G01985 |  | #N/A |
| AT3G13784 |  | #N/A |
| AT3G21030 |  | #N/A |
| AT3G02780 |  | #N/A |
| AT1G14210 |  | #N/A |
| AT2G29980 |  | #N/A |
| AT5G20710 |  | #N/A |
| AT5G18250 |  | #N/A |
| AT5G23740 |  | #N/A |
| AT1G16970 |  | #N/A |
| AT1G49520 |  | #N/A |
| AT4G39950 |  | #N/A |
| AT2G32060 |  | #N/A |
| AT2G40510 |  | #N/A |
| AT3G10720 |  | #N/A |
| AT1G52930 |  | #N/A |
| AT5G63000 |  | #N/A |
| AT2G02100 |  | #N/A |
| AT2G31141 |  | #N/A |
| AT5G45820 |  | #N/A |
| AT4G32460 |  | #N/A |
| AT1G07830 |  | #N/A |
| AT1G10760 |  | #N/A |
| AT3G25940 |  | #N/A |
| AT2G45860 |  | #N/A |
| AT3G59650 |  | #N/A |
| AT5G08185 |  | #N/A |
| AT2G04845 |  | #N/A |
| AT5G55720 |  | #N/A |
| AT2G30210 |  | #N/A |
| AT5G05250 |  | #N/A |
| AT1G63310 |  | #N/A |
| AT2G44140 |  | #N/A |
| AT2G32100 |  | #N/A |
| AT5G11260 |  | #N/A |
| AT2G25720 |  | #N/A |
| AT4G24110 |  | #N/A |
| AT5G62930 |  | #N/A |
| AT5G07322 |  | #N/A |
| AT3G22750 |  | #N/A |
| AT3G15500 |  | #N/A |
| AT1G08890 |  | #N/A |
| AT3G25730 |  | #N/A |
| AT1G21670 |  | #N/A |
| AT1G19610 |  | #N/A |
| AT1G61570 |  | #N/A |
| AT5G05370 |  | #N/A |
| AT1G34315 |  | #N/A |
| AT2G14610 |  | #N/A |
| AT3G46020 |  | #N/A |
| AT2G33850 |  | #N/A |
| AT4G22990 |  | #N/A |
| AT4G22070 |  | #N/A |
| AT5G48010 |  | #N/A |
| AT3G57710 |  | #N/A |
| AT1G11600 |  | #N/A |
| AT4G25810 |  | #N/A |
| AT3G46330 |  | #N/A |
| AT2G20870 |  | #N/A |
| AT4G25470 |  | #N/A |
| AT2G07711 |  | #N/A |
| AT5G04370 |  | #N/A |
| AT3G29034 |  | #N/A |
| AT5G59613 |  | #N/A |
| AT5G66740 |  | #N/A |
| AT1G77885 |  | #N/A |
| AT1G43790 |  | #N/A |
| AT1G58380 |  | #N/A |
| AT1G69880 |  | #N/A |
| AT5G62210 |  | #N/A |
| AT1G63360 |  | #N/A |
| AT2G19990 |  | #N/A |
| ATMG01380 |  | #N/A |
| AT3G02850 |  | #N/A |
| AT1G02800 |  | #N/A |
| AT3G14067 |  | #N/A |
| AT5G27100 |  | #N/A |
| AT1G14060 |  | #N/A |
| AT1G68840 |  | #N/A |
| AT5G60730 |  | #N/A |
| AT5G51220 |  | #N/A |
| AT3G10860 |  | #N/A |
| AT3G03770 |  | #N/A |
| AT1G26410 |  | #N/A |
| AT3G45100 |  | #N/A |
| AT2G45210 |  | #N/A |
| AT5G18540 |  | #N/A |
| AT4G28480 |  | #N/A |
| AT4G33610 |  | #N/A |
| AT5G65890 |  | #N/A |
| AT1G49310 |  | #N/A |
| AT4G08770 |  | #N/A |
| AT1G63880 |  | #N/A |
| AT2G21770 |  | #N/A |
| AT4G13520 |  | #N/A |
| AT5G35732 |  | #N/A |
| AT1G27370 |  | #N/A |
| AT3G56020 |  | #N/A |
| AT1G73680 |  | #N/A |
| AT2G23600 |  | #N/A |
| AT4G12600 |  | #N/A |
| AT5G11910 |  | #N/A |
| AT5G53980 |  | #N/A |
| AT4G02410 |  | #N/A |
| AT5G63600 |  | #N/A |
| AT1G53700 |  | #N/A |
| AT1G44830 |  | #N/A |
| AT1G20160 |  | #N/A |
| AT5G18840 |  | #N/A |
| ATMG00020 |  | #N/A |
| AT2G36170 |  | #N/A |
| AT1G21240 |  | #N/A |
| AT4G21585 |  | #N/A |
| AT4G33150 |  | #N/A |
| AT3G16720 |  | #N/A |
| AT2G25510 |  | #N/A |
| AT1G07090 |  | #N/A |
| AT5G51060 |  | #N/A |
| AT1G12160 |  | #N/A |
| AT5G41010 |  | #N/A |
| AT4G27410 |  | #N/A |
| AT5G37410 |  | #N/A |
| AT4G21610 |  | #N/A |
| AT3G10110 |  | #N/A |
| AT2G30750 |  | #N/A |
| AT2G31083 |  | #N/A |
| AT3G27831 |  | #N/A |
| AT3G45440 |  | #N/A |
| AT5G47990 |  | #N/A |
| AT5G20080 |  | #N/A |
| AT4G37260 |  | #N/A |
| AT3G53160 |  | #N/A |
| AT5G48240 |  | #N/A |
| AT4G27260 |  | #N/A |
| AT3G19580 |  | #N/A |
| AT5G57340 |  | #N/A |
| AT2G37450 |  | #N/A |
| AT5G09978 |  | #N/A |
| AT2G23180 |  | #N/A |
| AT3G54530 |  | #N/A |
| AT5G44980 |  | #N/A |
| AT4G13495 |  | #N/A |
| AT4G31985 |  | #N/A |
| AT3G44750 |  | #N/A |
| AT4G32720 |  | #N/A |
| AT1G75670 |  | #N/A |
| AT1G59660 |  | #N/A |
| AT5G57050 |  | #N/A |
| AT3G02170 |  | #N/A |
| AT4G04223 |  | #N/A |
| AT1G66090 |  | #N/A |
| AT2G23940 |  | #N/A |
| AT3G43980 |  | #N/A |
| AT3G19508 |  | #N/A |
| AT4G08780 |  | #N/A |
| AT1G15870 |  | #N/A |
| AT5G37500 |  | #N/A |
| AT5G59950 |  | #N/A |
| AT3G03010 |  | #N/A |
| AT4G16260 |  | #N/A |
| AT4G26950 |  | #N/A |
| AT3G59900 |  | #N/A |
| AT1G13145 |  | #N/A |
| AT1G17870 |  | #N/A |
| AT1G26970 |  | #N/A |
| AT4G11880 |  | #N/A |
| AT1G56630 |  | #N/A |
| AT5G64000 |  | #N/A |
| AT4G22305 |  | #N/A |
| AT5G23110 |  | #N/A |
| AT3G50520 |  | #N/A |
| AT3G24480 |  | #N/A |
| AT5G55960 |  | #N/A |
| AT3G49400 |  | #N/A |
| AT1G47580 |  | #N/A |
| AT2G39675 |  | #N/A |
| AT2G38080 |  | #N/A |
| AT2G17845 |  | #N/A |
| AT5G06550 |  | #N/A |
| AT1G22900 |  | #N/A |
| AT1G79075 |  | #N/A |
| AT3G52060 |  | #N/A |
| AT5G11460 |  | #N/A |
| AT1G16000 |  | #N/A |
| AT2G27330 |  | #N/A |
| AT1G55240 |  | #N/A |
| AT5G27990 |  | #N/A |
| AT4G23700 |  | #N/A |
| AT3G13437 |  | #N/A |
| AT1G60505 |  | #N/A |
| AT3G50800 |  | #N/A |
| AT5G23155 |  | #N/A |
| AT4G15290 |  | #N/A |
| AT4G26790 |  | #N/A |
| AT5G36910 |  | #N/A |
| AT1G26440 |  | #N/A |
| AT5G39680 |  | #N/A |
| AT2G39040 |  | #N/A |
| AT5G52640 |  | #N/A |
| AT5G65990 |  | #N/A |
| AT1G54450 |  | #N/A |
| AT5G23380 |  | #N/A |
| AT4G34760 |  | #N/A |
| AT3G02790 |  | #N/A |
| AT3G06700 |  | #N/A |
| AT4G26230 |  | #N/A |
| AT1G61960 |  | #N/A |
| AT2G11140 |  | #N/A |
| AT4G34750 |  | #N/A |
| AT1G25260 |  | #N/A |
| AT5G36220 |  | #N/A |
| AT4G27450 |  | #N/A |
| AT1G29395 |  | #N/A |
| AT1G69930 |  | #N/A |
| AT1G19240 |  | #N/A |
| AT1G32780 |  | #N/A |
| AT5G55200 |  | #N/A |
| AT5G46790 |  | #N/A |
| AT5G43190 |  | #N/A |
| AT3G15395 |  | #N/A |
| AT1G22190 |  | #N/A |
| AT5G39890 |  | #N/A |
| AT1G43910 |  | #N/A |
| AT5G06730 |  | #N/A |
| AT5G52380 |  | #N/A |
| AT3G17520 |  | #N/A |
| AT5G50800 |  | #N/A |
| AT4G15770 |  | #N/A |
| AT3G50460 |  | #N/A |
| AT1G32630 |  | #N/A |
| AT1G49160 |  | #N/A |
| AT5G57530 |  | #N/A |
| AT5G53120 |  | #N/A |
| AT4G38100 |  | #N/A |
| AT5G18810 |  | #N/A |
| AT2G26400 |  | #N/A |
| AT2G39030 |  | #N/A |
| AT2G44798 |  | #N/A |
| AT5G14690 |  | #N/A |
| AT2G40800 |  | #N/A |
| AT2G01940 |  | #N/A |
| AT5G41765 |  | #N/A |
| AT4G21830 |  | #N/A |
| AT3G17609 |  | #N/A |
| AT5G33370 |  | #N/A |
| AT3G61890 |  | #N/A |
| AT4G01390 |  | #N/A |
| AT3G10520 |  | #N/A |
| AT2G20450 |  | #N/A |
| AT5G01180 |  | #N/A |
| AT1G61420 |  | #N/A |
| AT2G29010 |  | #N/A |
| AT5G42580 |  | #N/A |
| AT3G23550 |  | #N/A |
| AT5G62920 |  | #N/A |
| AT3G62150 |  | #N/A |
| AT3G07910 |  | #N/A |
| AT3G49510 |  | #N/A |
| AT4G28300 |  | #N/A |
| AT5G23330 |  | #N/A |
| AT4G01897 |  | #N/A |
| AT5G04140 |  | #N/A |
| AT5G46500 |  | #N/A |
| AT1G67148 |  | #N/A |
| AT3G28750 |  | #N/A |
| AT3G28650 |  | #N/A |
| AT4G13340 |  | #N/A |
| AT3G19130 |  | #N/A |
| AT5G07040 |  | #N/A |
| AT4G28290 |  | #N/A |
| AT5G48580 |  | #N/A |
| AT5G57770 |  | #N/A |
| AT3G47010 |  | #N/A |
| AT4G29480 |  | #N/A |
| AT1G69270 |  | #N/A |
| AT3G28160 |  | #N/A |
| AT2G24240 |  | #N/A |
| AT1G20100 |  | #N/A |
| AT5G65740 |  | #N/A |
| AT2G29620 |  | #N/A |
| AT4G32800 |  | #N/A |
| AT1G22270 |  | #N/A |
| AT4G05100 |  | #N/A |
| AT5G20190 |  | #N/A |
| AT5G27400 |  | #N/A |
| AT4G18670 |  | #N/A |
| AT4G38780 |  | #N/A |
| AT4G04614 |  | #N/A |
| AT1G77750 |  | #N/A |
| AT4G26080 |  | #N/A |
| AT2G23430 |  | #N/A |
| AT3G50190 |  | #N/A |
| AT1G68550 |  | #N/A |
| AT4G29310 |  | #N/A |
| AT5G38840 |  | #N/A |
| AT1G52710 |  | #N/A |
| AT1G56045 |  | #N/A |
| AT3G22210 |  | #N/A |
| AT5G56940 |  | #N/A |
| AT1G25275 |  | #N/A |
| AT1G53760 |  | #N/A |
| AT4G01430 |  | #N/A |
| AT1G19380 |  | #N/A |
| AT1G23290 |  | #N/A |
| AT4G35560 |  | #N/A |
| AT2G29940 |  | #N/A |
| AT1G56570 |  | #N/A |
| AT5G03552 |  | #N/A |
| AT1G61370 |  | #N/A |
| AT2G26530 |  | #N/A |
| AT4G08115 |  | #N/A |
| AT4G02075 |  | #N/A |
| AT3G61620 |  | #N/A |
| AT1G67030 |  | #N/A |
| AT1G21270 |  | #N/A |
| AT1G70260 |  | #N/A |
| AT2G37040 |  | #N/A |
| AT3G49790 |  | #N/A |
| AT4G16960 |  | #N/A |
| AT1G27435 |  | #N/A |
| AT5G45600 |  | #N/A |
| AT1G29430 |  | #N/A |
| AT1G17450 |  | #N/A |
| AT4G28420 |  | #N/A |
| AT5G58760 |  | #N/A |
| AT3G25120 |  | #N/A |
| AT4G26690 |  | #N/A |
| AT4G08100 |  | #N/A |
| AT2G24110 |  | #N/A |
| AT5G14105 |  | #N/A |
| AT5G35935 |  | #N/A |
| AT2G45600 |  | #N/A |
| AT3G55700 |  | #N/A |
| AT2G23810 |  | #N/A |
| AT1G55860 |  | #N/A |
| AT5G54370 |  | #N/A |
| AT2G01730 |  | #N/A |
| AT2G33847 |  | #N/A |
| AT4G28040 |  | #N/A |
| AT5G23360 |  | #N/A |
| AT5G47980 |  | #N/A |
| AT1G74270 |  | #N/A |
| AT2G36160 |  | #N/A |
| AT4G13800 |  | #N/A |
| AT3G55170 |  | #N/A |
| AT3G19030 |  | #N/A |
| AT5G18920 |  | #N/A |
| AT2G44860 |  | #N/A |
| AT4G26670 |  | #N/A |
| AT3G13610 |  | #N/A |
| AT4G18250 |  | #N/A |
| AT3G25882 |  | #N/A |
| AT4G04700 |  | #N/A |
| AT2G33370 |  | #N/A |
| AT4G12090 |  | #N/A |
| AT1G69730 |  | #N/A |
| AT3G23880 |  | #N/A |
| AT1G77570 |  | #N/A |
| AT5G50790 |  | #N/A |
| AT5G44710 |  | #N/A |
| AT3G23920 |  | #N/A |
| AT1G50400 |  | #N/A |
| AT2G35480 |  | #N/A |
| AT3G52420 |  | #N/A |
| AT5G14730 |  | #N/A |
| AT1G52100 |  | #N/A |
| AT5G43740 |  | #N/A |
| AT3G50810 |  | #N/A |
| AT5G43540 |  | #N/A |
| AT5G42700 |  | #N/A |
| AT3G07070 |  | #N/A |
| AT3G30725 |  | #N/A |
| AT2G44360 |  | #N/A |
| AT4G00380 |  | #N/A |
| AT4G00310 |  | #N/A |
| AT1G21900 |  | #N/A |
| AT5G67510 |  | #N/A |
| AT2G28290 |  | #N/A |
| AT1G63530 |  | #N/A |
| AT3G45210 |  | #N/A |
| AT4G22380 |  | #N/A |
| AT4G16680 |  | #N/A |
| AT4G12545 |  | #N/A |
| AT1G15350 |  | #N/A |
| AT5G57120 |  | #N/A |
| AT3G55240 |  | #N/A |
| AT1G50180 |  | #N/A |
| AT3G19380 |  | #N/A |
| AT3G46210 |  | #N/A |
| AT1G47278 |  | #N/A |
| AT3G60490 |  | #N/A |
| AT1G51500 |  | #N/A |
| AT4G34770 |  | #N/A |
| AT1G58270 |  | #N/A |
| AT1G32870 |  | #N/A |
| AT1G52450 |  | #N/A |
| AT5G28630 |  | #N/A |
| AT1G31320 |  | #N/A |
| AT5G27850 |  | #N/A |
| AT5G53450 |  | #N/A |
| AT5G06860 |  | #N/A |
| AT1G67550 |  | #N/A |
| AT2G14890 |  | #N/A |
| AT5G40690 |  | #N/A |
| AT1G33230 |  | #N/A |
| AT4G22212 |  | #N/A |
| AT1G34540 |  | #N/A |
| AT5G06630 |  | #N/A |
| AT3G13760 |  | #N/A |
| AT1G55810 |  | #N/A |
| AT1G10682 |  | #N/A |
| AT5G65050 |  | #N/A |
| AT5G61160 |  | #N/A |
| AT2G26110 |  | #N/A |
| AT1G30110 |  | #N/A |
| AT1G62570 |  | #N/A |
| AT3G17210 |  | #N/A |
| AT2G47380 |  | #N/A |
| AT1G12020 |  | #N/A |
| AT1G73965 |  | #N/A |
| AT4G33730 |  | #N/A |
| AT2G37990 |  | #N/A |
| AT3G17715 |  | #N/A |
| AT3G60900 |  | #N/A |
| AT4G36060 |  | #N/A |
| AT1G68880 |  | #N/A |
| AT5G37540 |  | #N/A |
| AT5G03120 |  | #N/A |
| AT5G02490 |  | #N/A |
| AT5G12190 |  | #N/A |
| AT1G49500 |  | #N/A |
| AT5G19190 |  | #N/A |
| AT2G29460 |  | #N/A |
| AT5G15120 |  | #N/A |
| AT1G49430 |  | #N/A |
| AT4G35800 |  | #N/A |
| AT3G15760 |  | #N/A |
| AT3G21050 |  | #N/A |
| AT4G34580 |  | #N/A |
| AT1G11210 |  | #N/A |
| AT1G30760 |  | #N/A |
| AT2G30020 |  | #N/A |
| AT1G05540 |  | #N/A |
| AT1G55990 |  | #N/A |
| AT3G08990 |  | #N/A |
| AT4G28720 |  | #N/A |
| AT3G28940 |  | #N/A |
| AT3G06145 |  | #N/A |
| AT1G25560 |  | #N/A |
| AT4G06534 |  | #N/A |
| AT5G50335 |  | #N/A |
| AT1G17330 |  | #N/A |
| AT5G57280 |  | #N/A |
| AT3G48390 |  | #N/A |
| AT5G02170 |  | #N/A |
| AT2G17500 |  | #N/A |
| AT5G44730 |  | #N/A |
| AT1G53990 |  | #N/A |
| AT3G41762 |  | #N/A |
| AT3G11120 |  | #N/A |
| AT4G19170 |  | #N/A |
| AT5G53650 |  | #N/A |
| AT2G22425 |  | #N/A |
| AT3G02910 |  | #N/A |
| AT3G18950 |  | #N/A |
| AT2G43150 |  | #N/A |
| AT2G28630 |  | #N/A |
| AT3G20760 |  | #N/A |
| AT1G10050 |  | #N/A |
| AT3G13435 |  | #N/A |
| AT2G37120 |  | #N/A |
| AT3G27290 |  | #N/A |
| AT4G37660 |  | #N/A |
| AT1G76780 |  | #N/A |
| AT1G67140 |  | #N/A |
| AT3G53590 |  | #N/A |
| AT3G01050 |  | #N/A |
| AT1G70300 |  | #N/A |
| AT1G07985 |  | #N/A |
| AT4G27900 |  | #N/A |
| AT4G38850 |  | #N/A |
| AT3G44010 |  | #N/A |
| AT5G23930 |  | #N/A |
| AT5G47060 |  | #N/A |
| AT5G45790 |  | #N/A |
| AT5G05060 |  | #N/A |
| AT3G22230 |  | #N/A |
| AT3G49410 |  | #N/A |
| AT3G14270 |  | #N/A |
| AT2G40460 |  | #N/A |
| AT3G21370 |  | #N/A |
| AT3G47836 |  | #N/A |
| AT4G37040 |  | #N/A |
| AT4G24920 |  | #N/A |
| AT1G14860 |  | #N/A |
| AT2G02955 |  | #N/A |
| AT1G64110 |  | #N/A |
| AT4G12620 |  | #N/A |
| AT2G04050 |  | #N/A |
| AT4G01975 |  | #N/A |
| AT2G24550 |  | #N/A |
| AT1G23200 |  | #N/A |
| AT1G11410 |  | #N/A |
| AT2G21220 |  | #N/A |
| AT4G35490 |  | #N/A |
| AT2G14210 |  | #N/A |
| AT1G33480 |  | #N/A |
| AT1G22640 |  | #N/A |
| AT5G09290 |  | #N/A |
| AT2G39330 |  | #N/A |
| AT1G11475 |  | #N/A |
| AT1G52040 |  | #N/A |
| AT5G27770 |  | #N/A |
| AT5G49740 |  | #N/A |
| AT1G35320 |  | #N/A |
| AT3G58020 |  | #N/A |
| AT5G02050 |  | #N/A |
| AT4G18930 |  | #N/A |
| AT3G59540 |  | #N/A |
| AT4G01380 |  | #N/A |
| AT2G40205 |  | #N/A |
| AT5G16950 |  | #N/A |
| AT5G12050 |  | #N/A |
| AT3G47340 |  | #N/A |
| AT1G62420 |  | #N/A |
| ATMG01370 |  | #N/A |
| AT1G53200 |  | #N/A |
| AT1G74810 |  | #N/A |
| AT1G13810 |  | #N/A |
| AT5G23030 |  | #N/A |
| AT4G01790 |  | #N/A |
| AT1G54380 |  | #N/A |
| AT3G52670 |  | #N/A |
| AT1G32450 |  | #N/A |
| AT2G43530 |  | #N/A |
| AT1G08180 |  | #N/A |
| AT3G51860 |  | #N/A |
| AT1G51820 |  | #N/A |
| AT4G22830 |  | #N/A |
| AT5G09530 |  | #N/A |
| AT2G47990 |  | #N/A |
| AT2G15880 |  | #N/A |
| AT2G36930 |  | #N/A |
| AT4G14270 |  | #N/A |
| AT3G55340 |  | #N/A |
| AT3G14810 |  | #N/A |
| AT3G57860 |  | #N/A |
| AT3G59840 |  | #N/A |
| AT3G47370 |  | #N/A |
| AT1G14790 |  | #N/A |
| AT5G49270 |  | #N/A |
| AT4G16670 |  | #N/A |
| AT4G04780 |  | #N/A |
| AT1G64400 |  | #N/A |
| AT1G04290 |  | #N/A |
| AT5G57800 |  | #N/A |
| AT1G54000 |  | #N/A |
| AT1G13420 |  | #N/A |
| AT2G43870 |  | #N/A |
| AT5G27420 |  | #N/A |
| AT4G03210 |  | #N/A |
| AT3G24715 |  | #N/A |
| AT1G14980 |  | #N/A |
| AT3G24780 |  | #N/A |
| AT3G60910 |  | #N/A |
| AT3G49100 |  | #N/A |
| AT1G64563 |  | #N/A |
| AT5G61550 |  | #N/A |
| AT3G16080 |  | #N/A |
| AT1G04840 |  | #N/A |
| AT4G24275 |  | #N/A |
| AT5G57290 |  | #N/A |
| AT5G09585 |  | #N/A |
| AT3G08860 |  | #N/A |
| AT3G28150 |  | #N/A |
| AT4G21940 |  | #N/A |
| AT3G57720 |  | #N/A |
| AT1G15550 |  | #N/A |
| AT3G50845 |  | #N/A |
| AT1G19900 |  | #N/A |
| AT1G02730 |  | #N/A |
| AT3G23150 |  | #N/A |
| AT3G15356 |  | #N/A |
| AT4G17770 |  | #N/A |
| AT1G56150 |  | #N/A |
| AT5G06400 |  | #N/A |
| AT1G78070 |  | #N/A |
| AT3G02550 |  | #N/A |
| AT2G43200 |  | #N/A |
| AT1G57860 |  | #N/A |
| AT3G22100 |  | #N/A |
| AT5G67400 |  | #N/A |
| AT5G61820 |  | #N/A |
| AT1G79150 |  | #N/A |
| AT1G55020 |  | #N/A |
| AT2G18860 |  | #N/A |
| AT1G72210 |  | #N/A |
| AT2G35980 |  | #N/A |
| AT4G25315 |  | #N/A |
| AT5G34871 |  | #N/A |
| AT3G25890 |  | #N/A |
| AT2G23985 |  | #N/A |
| AT2G35736 |  | #N/A |
| AT3G59390 |  | #N/A |
| AT2G21290 |  | #N/A |
| AT2G27690 |  | #N/A |
| AT4G17490 |  | #N/A |
| AT5G41690 |  | #N/A |
| AT3G16700 |  | #N/A |
| AT1G18850 |  | #N/A |
| AT4G25760 |  | #N/A |
| AT4G25220 |  | #N/A |
| AT1G08930 |  | #N/A |
| AT1G50055 |  | #N/A |
| AT1G30250 |  | #N/A |
| AT1G49700 |  | #N/A |
| AT3G01600 |  | #N/A |
| AT5G51510 |  | #N/A |
| AT2G47880 |  | #N/A |
| AT5G53870 |  | #N/A |
| AT5G38020 |  | #N/A |
| AT5G65060 |  | #N/A |
| AT1G26470 |  | #N/A |
| AT5G60660 |  | #N/A |
| AT5G27250 |  | #N/A |
| AT1G48100 |  | #N/A |
| AT3G09735 |  | #N/A |
| AT1G51650 |  | #N/A |
| AT5G43970 |  | #N/A |
| AT5G27660 |  | #N/A |
| AT1G35250 |  | #N/A |
| AT5G05900 |  | #N/A |
| AT1G61210 |  | #N/A |
| AT1G80820 |  | #N/A |
| AT1G57660 |  | #N/A |
| AT5G51810 |  | #N/A |
