# Supplementary material for: Depletion of Arabidopsis SC35 and SC35-like serine/arginine-rich proteins affects the transcription and splicing of a subset of genes
Source: PLoS Genet. 2017 Mar 8;13(3):e1006663. doi: 10.1371/journal.pgen.1006663 (PMC5362245; doi:10.1371/journal.pgen.1006663)
Supplement: S5 Table — (DOCX) [file pgen.1006663.s021.docx]

**Table S5 Primers used in this study**

| Primers | Sequence | Purpose |
| --- | --- | --- |
| XBAT35-UNSKIP/S-5' | gatgcacagccacgcacagt | analysis of the splicing efficiency |
| XBAT35-UNSKIP-3' | tgcggcttgggattgtgga | analysis of the splicing efficiency |
| XBAT35-SKIP-3' | taagacgtgtttttgtggaG | analysis of the splicing efficiency |
| AGL70-UNSKIP/S-5' | aaagctcgacaactttcaat | analysis of the splicing efficiency |
| AGL70-UNSKIP-3' | gatctttgacatgttgtcac | analysis of the splicing efficiency |
| AGL70-SKIP-3' | tttctgcaagatcttgtcac | analysis of the splicing efficiency |
| BASS5-UNS/SP-5'-N | tcctcatggaattctgttaa | analysis of the splicing efficiency |
| BASS5-UNSP-3'-N | ttgatccaaaccttggcttg | analysis of the splicing efficiency |
| BASS5-SPL-3'-N | tacaaagtaccttggcttga | analysis of the splicing efficiency |
| AT4G02725-SP-5' | ggcatgtagctgggtaatac | analysis of the splicing efficiency |
| AT4G02725-SP-3' | ctgaatacgactttgggtag | analysis of the splicing efficiency |
| AT4G02725-UNS-5' | gacaatgctagtttctataa | analysis of the splicing efficiency |
| AT4G02725-UNS-3' | ctgaatacgactgaaccaaa | analysis of the splicing efficiency |
| AtSEN1-UN/SP-5' | atggcctcaactgatcttct | analysis of the splicing efficiency |
| AtSEN1-UN-3' | atcgccgtgaagccctgta | analysis of the splicing efficiency |
| AtSEN1-SPL-3' | agctcattctctgtccaagc | analysis of the splicing efficiency |
| AT5G47455-UNS/S-5' | agtaatttgtaaaagtgctc | analysis of the splicing efficiency |
| AT5G47455-UNS-3' | tgacagagcccacctttaa | analysis of the splicing efficiency |
| AT5G47455-SP-3' | gaaagtcccattttaaatac | analysis of the splicing efficiency |
| UMAMIT47-UNSKIP/S-5' | gttgtcatcgtcaaagtctg | analysis of the splicing efficiency |
| UMAMIT47-UNSKIP-3' | cattatatgtgtctggagaa | analysis of the splicing efficiency |
| UMAMIT47-SKIP-3' | agttattattccctggagaa | analysis of the splicing efficiency |
| AT1G53250-skip-RT-F | gaatacctaattcacttgct | analysis of the splicing efficiency |
| AT1G53250-skip-RT-R | tagctaatccaatcttcctt | analysis of the splicing efficiency |
| AT1G53250-unskip-RT-F | ggagttgaaatctgtaggtt | analysis of the splicing efficiency |
| AT1G53250-unskip-RT-R | atctttgttcatagctttca | analysis of the splicing efficiency |
| Actin-RT-F | ACGGTAACATTGTGCTCAGTGGTG | analysis of the splicing efficiency |
| Actin-RT-R | CTTGGAGATCCACATCTGCTGGA | analysis of the splicing efficiency |
|  |  |  |
| SCL28-ECORI-5' | NNNGAATTCatggctagagcgagaagccgga | constructs of pCambia1300-YFP/pCambia2300-CFP for analysis localization at cellular level |
| SCL28-SpeI-3' | NNACTAGTacgacttaaggatcgagaacgg | constructs of pCambia1300-YFP/pCambia2301-CFP for analysis localization at cellular level |
| SCL30-ECORI-5' | NNNGAATTCatgaggagatacagtccgcctt | constructs of pCambia1300-YFP/pCambia2302-CFP for analysis localization at cellular level |
| SCL30-SalI-3' | NNNGTCGACtcttggagatacctccacagac | constructs of pCambia1300-YFP/pCambia2303-CFP for analysis localization at cellular level |
| SCL30a-ECORI-5' | NNNGAATTCatgagaggaaggagctacacgc | constructs of pCambia1300-YFP/pCambia2304-CFP for analysis localization at cellular level |
| SCL30a-SalI-3' | NNNGTCGACatgagaggaaggagctacacgc | constructs of pCambia1300-YFP/pCambia2305-CFP for analysis localization at cellular level |
| SCL33-ECORI-5' | NNNGAATTCatgaggggaaggagctacactc | constructs of pCambia1300-YFP/pCambia2306-CFP for analysis localization at cellular level |
| SCL33-SalI-3' | NNNGTCGACctggcttggtgaacggtcttcg | constructs of pCambia1300-YFP/pCambia2307-CFP for analysis localization at cellular level |
| SC35-ECORI-5' | NNNGAATTCatgtcgcacttcggaaggtcag | constructs of pCambia1300-YFP/pCambia2308-CFP for analysis localization at cellular level |
| SC35-SalI-3' | NNNGTCGACttccgcagcataaggagattga | constructs of pCambia1300-YFP/pCambia2309-CFP for analysis localization at cellular level |
| CDKC1-ECOR1-5' | NNNGAATTCatggcgatggcatcattcgg | constructs of pCambia1300-YFP/pCambia2310-CFP for analysis localization at cellular level |
| CDKC1-Sal1-3' | NNNGTCGACctgttgccatccgtattgct | constructs of pCambia1300-YFP/pCambia2311-CFP for analysis localization at cellular level |
| CDKC2-ECOR1-5' | NNNGAATTCatggcggctgcggcttttgg | constructs of pCambia1300-YFP/pCambia2312-CFP for analysis localization at cellular level |
| CDKC2-Sal1-3' | NNNGTCGACcggttgccatccatattgtt | constructs of pCambia1300-YFP/pCambia2313-CFP for analysis localization at cellular level |
| U2AF65A-SalI-5' | NNNGTCGACatgtctgaattcgaagatcacg | constructs of pCambia1300-YFP/pCambia2314-CFP for analysis localization at cellular level |
| U2AF65A-SpeI-3' | NNNACTAGTggctccataatcaccctgttca | constructs of pCambia1300-YFP/pCambia2315-CFP for analysis localization at cellular level |
| U170K-SalI-5' | NNNGTCGACatgggagactccggcgatcctt | constructs of pCambia1300-YFP/pCambia2316-CFP for analysis localization at cellular level |
| U170K-SpeI-3' | NNNACTAGTacgaacatactctcgcgattct | constructs of pCambia1300-YFP/pCambia2317-CFP for analysis localization at cellular level |
|  |  |  |
| pSCL28-Sal1-5' | NNNGTCGACttcgggtgtctcctctcctccg | GUS staining |
| pSCL28-BamH1-3' | NNNGGATCCcgaagatttctcttccttaggt | GUS staining |
| pSCL30-Sal1-5' | NNNGTCGACctttctatcttttttcaggcca | GUS staining |
| pSCL30-BamH1-3' | NNNGGATCCcacctaccatagaaacaaaacg | GUS staining |
| pSCL30a-Sal1-5' | NNNGTCGACatcattttatagtatgagaaat | GUS staining |
| pSCL30a-BamH-3' | NNNGGATCCtgtgtctagactttaatatcta | GUS staining |
| pSCL33-Sal1-5' | NNNGTCGACtgtagataccaacagcttgact | GUS staining |
| pSCL33-BamH1-3' | NNNGGATCCtgagtcaagcttcaatctctct | GUS staining |
| pSC35-Sal1-5' | NNNGTCGACaccaatttcaaaagagtatcct | GUS staining |
| pSC35-BamH-3' | NNNGGATCCggttttcccgccggcggagaaa | GUS staining |
|  |  |  |
| SCL28-CLUC-XhoI-3' | NNNCTCGAGtcaacgacttaaggatcgagaa | Firefly Luciferase (LUC) Complementation Image Assay |
| SCL30-CLUC-SalI-3' | NNNGTCGACtcatcttggagatacctccaca | Firefly Luciferase (LUC) Complementation Image Assay |
| SCL30a-CLUC-SalI-3' | NNNGTCGACtcactggcttggagaacggtct | Firefly Luciferase (LUC) Complementation Image Assay |
| SCL33-CLUC-SalI-3' | NNNGTCGACtcactggcttggtgaacggtct | Firefly Luciferase (LUC) Complementation Image Assay |
| SC35-CLUC-SalI-3' | NNNGTCGACtcattccgcagcataaggagat | Firefly Luciferase (LUC) Complementation Image Assay |
| NRPB4-NLUC-KpnI-5' | NNNGGTACCatgtccggagaagaagaagaga | Firefly Luciferase (LUC) Complementation Image Assay |
| NRPB4-NLUC-SalI-3' | NNNGTCGACctcgaatctcttgacaagtgag | Firefly Luciferase (LUC) Complementation Image Assay |
| NRPB7-NLUC-BamHI-5' | NNNGGATCCatgtttttccacatagtattgg | Firefly Luciferase (LUC) Complementation Image Assay |
| NRPB7-NLUC-SalI-3' | NNNGTCGACtgccgctgcagggtcgtttatg | Firefly Luciferase (LUC) Complementation Image Assay |
|  |  |  |
| FLC-1-F | GACATATCCAGAAAAGGGCAAG | ChIP analysis |
| FLC-1-R | GGTGAATGTACGGCATGATTT | ChIP analysis |
| FLC-2-F | ATCCGTATCGTAGGGGAGGAAAGA | ChIP analysis |
| FLC-2-R | GAAGACAAGATTGCCACGTGTACC | ChIP analysis |
| FLC-3-F | AAACGTCGCAACGGTCTCAT | ChIP analysis |
| FLC-3-R | AAACCCAGGTAAGGAAAAGGCG | ChIP analysis |
| FLC-4-F | CACAGTAGTTTTGAATTTTGGTAGCTT | ChIP analysis |
| FLC-4-R | TGAAGTAGCATATGTGCGGTAAG | ChIP analysis |
| FLC-5-F | TTGACAATCCACAACCTCAATC | ChIP analysis |
| FLC-5-R | TCAATTTCCTAGAGGCACCAA | ChIP analysis |
| FLC-6-F | AGCCTTTTAGAACGTGGAACC | ChIP analysis |
| FLC-6-R | TCTTCCATAGAAGGAAGCGACT | ChIP analysis |
| FLC-7-F | CGATATGGGAAACAGCATGCTGA | ChIP analysis |
| FLC-7-R | GGGCTATGAAAATTGCGGTATGC | ChIP analysis |
|  |  |  |
| SC35-FLAG-Sac1-5' | NNNGAGCTCatgtcgcacttcggaaggtcag | Co-IP |
| SC35-FLAG-Kpn1-3' | NNNGGTACCttccgcagcataaggagattga | Co-IP |
| SCL28-FLAG-Sac1-5' | NNNGAGCTCatggctagagcgagaagccgga | Co-IP |
| SCL28-FLAG-Kpn1-3' | NNNGGTACCacgacttaaggatcgagaacgg | Co-IP |
| SCL30-FLAG-Kpn1-5' | NNNGGTACCatgaggagatacagtccgcctt | Co-IP |
| SCL30-FLAG-Kpn1-3' | NNNGGTACCtcttggagatacctccacagac | Co-IP |
| SCL30a-FLAG-Kpn1-5' | NNNGGTACCatgagaggaaggagctacacgc | Co-IP |
| SCL30a-FLAG-Kpn1-3' | NNNGGTACCctggcttggagaacggtctcca | Co-IP |
| SCL33-FLAG-Sac1-5' | NNNGAGCTCatgaggggaaggagctacactc | Co-IP |
| SCL33-FLAG-Kpn1-3' | NNNGGTACCctggcttggtgaacggtcttcg | Co-IP |
| NRPB4-ECORI-5' | NNNGAATTCatgtccggagaagaagaagaga | Co-IP |
| NRPB4-SalI-3' | NNNGTCGACctcgaatctcttgacaagtgag | Co-IP |
| NRPB7-ECORI-5' | NNNGAATTCatgtttttccacatagtattgg | Co-IP |
| NRPB7-SalI-3' | NNNGTCGACtgccgctgcagggtcgtttatg | Co-IP |
|  |  |  |
| SC35-AD-ECORI-5' | NNNGAATTCatgtcgcacttcggaaggtcag | Yeast Two-Hybrid |
| SC35-AD-BamH1-3' | NNNGGATCCtcattccgcagcataaggagat | Yeast Two-Hybrid |
| SCL28-AD-ECORI-5' | NNNGAATTCatggctagagcgagaagccgga | Yeast Two-Hybrid |
| SCL28-AD-BamH1-3' | NNNGGATCCtcaacgacttaaggatcgagaa | Yeast Two-Hybrid |
| SCL30-AD-ECORI-5' | NNNGAATTCatgaggagatacagtccgcctt | Yeast Two-Hybrid |
| SCL30-AD-Sal1-3' | NNNGTCGACtcatcttggagatacctccaca | Yeast Two-Hybrid |
| SCL30a-AD-ECOR1-5' | NNNGAATTCatgagaggaaggagctacacgc | Yeast Two-Hybrid |
| SCL30a-AD-Sal1-3' | NNNGTCGACtcactggcttggagaacggtct | Yeast Two-Hybrid |
| SCL33-AD-ECORI-5' | NNNGAATTCatgaggggaaggagctacactc | Yeast Two-Hybrid |
| SCL33-AD-BamH1-3' | NNNGGATCCtcactggcttggtgaacggtct | Yeast Two-Hybrid |
| U170K-BD-Sal1-5' | NNNGTCGACctatgggagactccggcgatcc | Yeast Two-Hybrid |
| U170K-BD-Sal1-3' | NNNGTCGACtcaacgaacatactctcgcgat | Yeast Two-Hybrid |
| U2AF65A-BD-BamH1-5' | NNNGGATCCgtatgtctgaattcgaagatca | Yeast Two-Hybrid |
| U2AF65A-BD-Sal1-3' | NNNGTCGACtcaggctccataatcaccctgt | Yeast Two-Hybrid |
| CDKC1-BD-ECORI-5' | NNNGAATTCatggcgatggcatcattcgggc | Yeast Two-Hybrid |
| CDKC1-BD-SalI-3' | NNNGTCGACttactgttgccatccgtattgct | Yeast Two-Hybrid |
| CDKC2-BD-ECORII-5' | NNNGAATTCatggcggctgcggcttttgggc | Yeast Two-Hybrid |
| CDKC2-BD-SalI-3' | NNNGTCGACttacggttgccatccatattgt | Yeast Two-Hybrid |
|  |  |  |
|  |  |  |
|  |  |  |
| CS853758-LP | TCACAGTCGTCGATTTCTTCC | T-DNA verification |
| CS852758-RP | GAAGTCCTCTTGTAGTCCCCG | T-DNA verification |
| CS805508-LP | AAGGAAAGAGGAGGCTTACCC | T-DNA verification |
| CS805508-RP | TAAGCCAAAATGGATCTCGAG | T-DNA verification |
| SALK_056672-LP | GTTGTCGAAACACACATACGG | T-DNA verification |
| SALK_056672-RP | GAGATCACTGTCACGACTCCC | T-DNA verification |
| SALK_058566-LP | TCTTCCTCGCCATTTTGTATG | T-DNA verification |
| SALK_058566-RP | AAAAGCTTCCTCCATGAGCTC | T-DNA verification |
| SALK_033824C-LP | GGCTTCTTCTGGGACTACGAC | T-DNA verification |
| SALK_033824C-RP | TGTGTCTTCGTCAAGGGGTAC | T-DNA verification |
| SALK_116747C-LP | GGTATCTTCTTCCCTGCAAGC | T-DNA verification |
| SALK_116747C-RP | ACGTCCATAAATTGCATCGTC | T-DNA verification |
| CS878689-LP | AATCATTCCTTCTGCCTCGAC | T-DNA verification |
| CS878689-RP | CCTTGGAGGAACCTTCAAATC | T-DNA verification |
| SALK_087841C-LP | TAATGTCACCGGGCAAGTTAC | T-DNA verification |
| SALK_087841C-RP | TTGTTGGCTTCAGACCAAATC | T-DNA verification |
| SALK_055412-LP | AGTTTGAGGATGCTCGTGATG | T-DNA verification |
| SALK_055412-RP | CTCGATGTAGATTTTGCAGGC | T-DNA verification |
| SALK_0146565C-LP | TTTGATTACCTGTGGGTCGAG | T-DNA verification |
| SALK_014656C-RP | ACTGCATAAACCCCCTACCAC | T-DNA verification |
| CS834516-LP | TGATTTCACGGCGTTTAAAAC | T-DNA verification |
| CS834516-RP | TCATAGTCGAAATTCCCAACG | T-DNA verification |
| SALK_118875-LP | AGAACAATCCACGAAAGCATG | T-DNA verification |
| SALK_118875-RP | TGTTTTGGGATTCTTCAGTGG | T-DNA verification |
| CS803022-LP | ACTCAACATTTGATTTTGGCG | T-DNA verification |
| CS803022-RP | TATCAGGTGACCTATCACGCC | T-DNA verification |
| SALK_114234-LP | GTGGCTGCAAATAAACGAATC | T-DNA verification |
| SALK_114234-RP | GCATCTCTTTCGTCATCGAAC | T-DNA verification |
| CS809215-LP | TCTTTTCATCCATGGATCTCG | T-DNA verification |
| CS809215-RP | CAATGGCACAATAAACCAAGG | T-DNA verification |
| SALK_031147C-LP | ATCATACGTCCTCGCTCACAC | T-DNA verification |
| SALK_031147C-RP | AAGGAGAGTGCAAATACGCAG | T-DNA verification |
| SALK_051525-LP | ACAGAACCAAAACTATGCCCC | T-DNA verification |
| SALK_051525-RP | GCAAATGTGATCAGAGTGTGC | T-DNA verification |
| LB1.3 | ATTTTGCCGATTTCGGAAC | T-DNA verification |
| LB2 | GCTTCCTATTATATCTTCCCAAATTACCAATACA | T-DNA verification |
| WisLB | AACGTCCGCAATGTGTTATTAAGTTGTC | T-DNA verification |
|  |  |  |
| SC35-RT-F | AACATCACCTTCCGTACGACTGCT | Real-time RT-PCR |
| SC35-RT-R | GCCTTTCCACTGCTTTGTGAGCTT | Real-time RT-PCR |
| SCL28-RT-F | CGGAAAGGCTACGACGATAAT | Real-time RT-PCR |
| SCL28-RT-R | TCGAGAGGGAGATTACGGATAA | Real-time RT-PCR |
| SCL30-RT-F | AAGAGCTTCGTGAGCCCTTTGAGA | Real-time RT-PCR |
| SCL30-RT-R | AGACTCTGAAGCAACAACCACCGT | Real-time RT-PCR |
| SCL30a-RT-F | TTTGGTGGGAGTCGTGACAGTGAT | Real-time RT-PCR |
| SCL30a-RT-R | AGATGTCCTTGACGGGACCAAACT | Real-time RT-PCR |
| SCL33-RT-F | CGCCATGATTGCAGGCAAGAAGAT | Real-time RT-PCR |
| SCL33-RT-R | AATTGAACGAACCCAAACCCTCGC | Real-time RT-PCR |
| AT2G32220-RT-F | GGCAAAGAAGACGGCTAAGA | Real-time RT-PCR |
| AT2G32220-RT-R | GAGACAACGTTCTTCAGATCCA | Real-time RT-PCR |
| AT3G01790-RT-F | CGTATCAATCTCGACGGTCTTC | Real-time RT-PCR |
| AT3G01790-RT-R | CGTCGCGGTTAGGACAATAA | Real-time RT-PCR |
| AT3G06320-RT-F | GACGTTCATGTTCATCCGTCTA | Real-time RT-PCR |
| AY3G06320-RT-R | TCCCTTGGTACTCTTCCTCTT | Real-time RT-PCR |
| AT5G19890-RT-F | GCTCGTGACTCCGTTGTATT | Real-time RT-PCR |
| AY5919890-RT-R | GGTAGATTGTTTGCACTGTTCTG | Real-time RT-PCR |
| AT1G49570-RT-F | TGGGACGAAGGGACTCATTA | Real-time RT-PCR |
| AT1G49570-RT-R | GGTCACGAACTTGGCTGTTA | Real-time RT-PCR |
| HAI2-RT-F | GTTGCGGCTAGGTGTAAAGA | Real-time RT-PCR |
| HAI2-RT-R | CTACGCTCCATCATCTTCTTCC | Real-time RT-PCR |
| TAT3-RT-F | CCCGAGAGTGATTGGGAAAT | Real-time RT-PCR |
| TAT3-RT-R | GTTGAGATGGTCGTAGGTGTAG | Real-time RT-PCR |
| PYL6-RT-F | GAACACCCTCAAGCGTACAA | Real-time RT-PCR |
| PYL6-RT-R | TCAAGCCGCTCTAAGCTAAAC | Real-time RT-PCR |
| FPA-RT-F | CCCGTCAAGAACGGCTATATG | Real-time RT-PCR |
| FPA-RT-R | GCAGAGGATTGGACTGAGATTC | Real-time RT-PCR |
| FVE-RT-F | TGGAGTTGGTTCGCCTATTT | Real-time RT-PCR |
| FVE-RT-R | CTCCCAAAGACGGATGACTTAT | Real-time RT-PCR |
| FY-RT-F | CCCTCAATGCCTATGTCTCATC | Real-time RT-PCR |
| FY-RT-R | CCTGAAGGAGCACCCATAAA | Real-time RT-PCR |
| VRN1-RT-F | CATCACCACTTCCTGAGTCTAC | Real-time RT-PCR |
| VRN1-RT-R | CGTTGGCTCTTCAGCTTTAAC | Real-time RT-PCR |
| VRN2-RT-F | GCTAGAAGCCACCTACTTCTTC | Real-time RT-PCR |
| VRN2-RT-R | CGCTATCCCGATCAGACATTAC | Real-time RT-PCR |
| VIL2-RT-F | GAGAGTGTGAAGGGAAGAAGAG | Real-time RT-PCR |
| VIL2-RT-R | CTGGAATCACATACCGAGAAGG | Real-time RT-PCR |
| VRN5-RT-F | AGTAGAACTGAGAGGAGGATGG | Real-time RT-PCR |
| VRN5-RT-R | GCGTTCGAATGGCCAAATATAC | Real-time RT-PCR |
| AT2G11880-RT-F | ACTGAAGTGTCCCAAATCCTG | Real-time RT-PCR |
| AT2G11880-RT-R | CGTCTCCACTAATTCACCCTG | Real-time RT-PCR |
| AT4G39675-RT-F | AGTTACGCACACCTACAAGTC | Real-time RT-PCR |
| AT4G39675-RT-R | CGGACGCAAAAGATGTTTCG | Real-time RT-PCR |
| AT1G56660-RT-F | AGCGATGTGAAAGTGGAAGAG | Real-time RT-PCR |
| AT1G56660-RT-R | CCCGACTCATCCTTCTCTTTT | Real-time RT-PCR |
| EMB975-RT-F | AAACCCGATAAACCGAGTCG | Real-time RT-PCR |
| EMB975-RT-R | TTGGAGTTGGAAGACCTAAAGG | Real-time RT-PCR |
| AT3G44450-RT-F | ACAAAACAAGACAGGTACAAAGAC | Real-time RT-PCR |
| AT3G44450-RT-R | TCCATCCCATAAGCAATCCTTC | Real-time RT-PCR |
| ATCAPE3-RT-F | ATGCCCGTAACTATGCTAACC | Real-time RT-PCR |
| ATCAPE3-RT-R | TCGTAGTCAAATTGCTCGTCC | Real-time RT-PCR |
| AT2G11410-RT-F | GCAAGATATGAAGACGCTGTTG | Real-time RT-PCR |
| AT2G11410-RT-R | CCCGAATCCAGAAAGTTAAATCC | Real-time RT-PCR |
| AT2G11880-RT-F | ACTGAAGTGTCCCAAATCCTG | Real-time RT-PCR |
| AT2G11880-RT-R | CGTCTCCACTAATTCACCCTG | Real-time RT-PCR |
| AT3G27860-RT-F | AGTTGCAGAGCGAGAAAGATC | Real-time RT-PCR |
| AT3G27860-RT-R | ACGGATTAGAGCCAGCATTTC | Real-time RT-PCR |
| AT3G27250-RT-F | TCGGAGAGGAAACACAAGAAAC | Real-time RT-PCR |
| AT3G27250-RT-R | GGAACTCAAACTACCCTCACAC | Real-time RT-PCR |
| AT1G80440-RT-F | AACTTATCCCCAATCTTCCCG | Real-time RT-PCR |
| AT1G80440-RT-R | GAGACTTCACGGTTCCAGG | Real-time RT-PCR |
| NCED3-RT-F | CCCAAAGCCAAAGAATCCAAC | Real-time RT-PCR |
| NCED3-RT-R | CACTAGGATCAGCCGTTTTAGG | Real-time RT-PCR |
| SZF1-RT-F | GAGTGTGCTTTTGTTCATCCG | Real-time RT-PCR |
| SZF1-RT-R | AGAATCTCCTTTCGGGCATG | Real-time RT-PCR |
| FT-RT-F | GGCCAAAGAGAGGTGACTAATG | Real-time RT-PCR |
| FT-RT-R | GGTCTTCTCCACCAATCTCAAC | Real-time RT-PCR |
| SOC1-RT-F | AAACTCTTGGGAGAAGGCATAG | Real-time RT-PCR |
| SOC1-RT-R | TGAGTCTTTCTTGCTCGAATACA | Real-time RT-PCR |
| FLC-RT-1-F | CTCGTCAGCTTTCTGTTCTCT | Real-time RT-PCR |
| FLC-RT-1-R | TCAAGGATCTTGACCAGGTTATC | Real-time RT-PCR |
| FLC-RT-2-F | tccggcaagctctacagcttc | Real-time RT-PCR |
| FLC-RT-2-R | agcatgctgtttcccatatcgatc | Real-time RT-PCR |
| FLC-RT-3-F | GCAAGCTTGTGGGATCAAATG | Real-time RT-PCR |
| FLC-RT-3-R | TCGGTCTTCTTGGCTCTAGT | Real-time RT-PCR |
| FLC-RT-4-F | GCCAAGAAGACCGAACTCAT | Real-time RT-PCR |
| FLC-RT-4-R | TCTGCTCCCACATGATGATTATT | Real-time RT-PCR |
| PIF3-F | TGCTTCTGCTACGCCTAAAG | Real-time RT-PCR |
| PIF3-R | CCGAGCCAACAGAAGAACATA | Real-time RT-PCR |
| PIF4-F | CAGATGCAATCGGTAACAAGTC | Real-time RT-PCR |
| PIF4-R | TCCTATCTCTCCTCCTCCTTTC | Real-time RT-PCR |
| PIF5-F | TCACTGCAGCAGAACAGATAAA | Real-time RT-PCR |
| PIF5-R | ATTCCACTTCCCATCCACATC | Real-time RT-PCR |
| RBCS-RT-F | GCAAGGCTAACAACGACATTAC | Real-time RT-PCR |
| RBCS-RT-R | AGGTCAGGAAGGTAAGAGAGAG | Real-time RT-PCR |
| CAB1-RT-F | GAAGGTGAAGGAGCTCAAGAA | Real-time RT-PCR |
| CAB1-RT-R | ATGGTCAGCAAGGTTCTCTATC | Real-time RT-PCR |
| APL3-RT-F | TCTGGCTGCTACACAAACTC | Real-time RT-PCR |
| APL3-RT-R | ATCCTCAAACACCCACAGAAA | Real-time RT-PCR |
| CHS-RT-F | GTCCCTAAGCTAGGCAAAGAAG | Real-time RT-PCR |
| CHS-RT-R | GTAGTGCAGAAGACGACATGAG | Real-time RT-PCR |
| ABI1-RT-F | GTCTCACATCTTCGTCGCTAAC | Real-time RT-PCR |
| ABI1-RT-R | CGCAGCTTCATCTTCTCTATCC | Real-time RT-PCR |
| ABI2-RT-F | GTGAGTTCGAGATGACCTTTGA | Real-time RT-PCR |
| ABI2-RT-R | CAACACCTCCCACACTACATAA | Real-time RT-PCR |
| ABI3-RT-F | CAGCAGAACCAAACCCAAATC | Real-time RT-PCR |
| ABI3-RT-R | CGGGACATTAGGCCAATACA | Real-time RT-PCR |
| ABI4-RT-F | TCCTCCACTCAAACCCTAAGA | Real-time RT-PCR |
| ABI4-RT-R | GGTCCCACCATTAAGGAAGATG | Real-time RT-PCR |
| ABI5-RT-F | GGAGAGATGACACTTGAGGATTT | Real-time RT-PCR |
| ABI5-RT-R | TGGTTCGGGTTTGGATTAGG | Real-time RT-PCR |
| PIF1-F | GATGTCAATGGGATGTGGAATG | Real-time RT-PCR |
| PIF1-R | CATGAAGGAAGGAGGAGGAATAG | Real-time RT-PCR |
| SPT-F | GTTTCTCCGGTGGTGGTAATAA | Real-time RT-PCR |
| SPT-R | CTCCAACGGAAGAAGACGATAC | Real-time RT-PCR |
| Actin-RT-F | ACGGTAACATTGTGCTCAGTGGTG | Real-time RT-PCR |
| Actin-RT-R | CTTGGAGATCCACATCTGCTGGA | Real-time RT-PCR |
|  |  |  |
| SR34-CRISPR-5' | GATTgtgtacgtcggaaaccttcc | CRISPR |
| SR34-CRISPR-3' | AAACggaaggtttccgacgtacac | CRISPR |
| RSZ22a-CRISPR-1-5' | GATTgcgtgtgtatgttggtaatttgg | CRISPR |
| RSZ22a-CRISPR-1-3' | AAACccaaattaccaacatacacacgc | CRISPR |
| RSZ22a-CRISPR-2-5' | GATTgtagagcagtctcataaccgtgg | CRISPR |
| RSZ22a-CRISPR-2-3' | AAACccacggttatgagactgctctac | CRISPR |
|  |  |  |
|  |  |  |
| eIF4A-F | TGACCACACAGTCTCTGCAA | mRNA decay analysis |
| eIF4A-R | ACCAGGGAGACTTGTTGGAC | mRNA decay analysis |
